# C–H Bonds as Functional Groups: Simultaneous Generation of Multiple Stereocenters by Enantioselective Hydroxylation at Unactivated Tertiary C–H Bonds

**DOI:** 10.1021/jacs.2c10148

**Published:** 2023-07-11

**Authors:** Andrea Palone, Guillem Casadevall, Sergi Ruiz-Barragan, Arnau Call, Sílvia Osuna, Massimo Bietti, Miquel Costas

**Affiliations:** † Institut de Química Computacional i Catàlisi (IQCC) and Departament de Química, Universitat de Girona, Campus Montilivi, Girona, Catalonia E-17071, Spain; ‡ Dipartimento di Scienze e Tecnologie Chimiche, Università “Tor Vergata”, Via della Ricerca Scientifica, 1, I-00133 Rome, Italy; § ICREA, Pg. Lluís Companys 23, Barcelona 08010, Spain

## Abstract

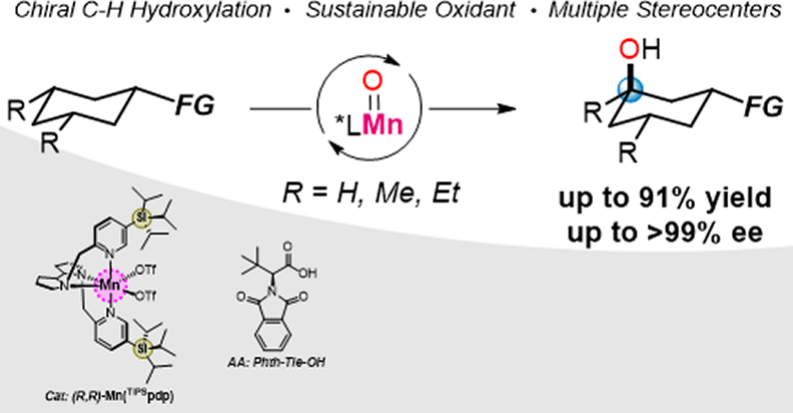

Enantioselective C–H oxidation is a standing chemical challenge foreseen as a powerful tool to transform readily available organic molecules into precious oxygenated building blocks. Here, we describe a catalytic enantioselective hydroxylation of tertiary C–H bonds in cyclohexane scaffolds with H_2_O_2_, an evolved manganese catalyst that provides structural complementary to the substrate similarly to the lock-and-key recognition operating in enzymatic active sites. Theoretical calculations unveil that enantioselectivity is governed by the precise fitting of the substrate scaffold into the catalytic site, through a network of complementary weak non-covalent interactions. Stereoretentive C(*sp*^3^)–H hydroxylation results in a single-step generation of multiple stereogenic centers (up to 4) that can be orthogonally manipulated by conventional methods providing rapid access, from a single precursor to a variety of chiral scaffolds.

## Introduction

**Figure 1:**
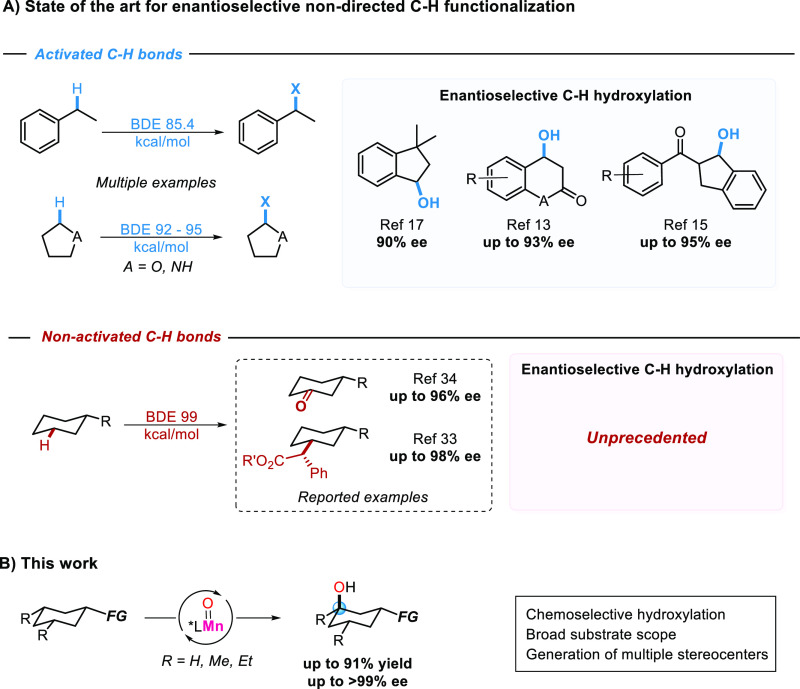
State of the art for non-enzymatic non-directed enantioselective C(*sp*^3^)–H functionalization reactions. (A) Selected precedents for enantioselective functionalization of activated (top) and non-activated (bottom) C–H bonds. (B) Features of the current work.

Given the ubiquity of chiral oxygenated aliphatic moieties in natural and bioactive products,^1^ reactions that construct C–O bonds in an enantioselective manner from readily available C(*sp*^3^)–H bond rich starting materials are coveted synthetic tools.^2−4^ Such reactions can expand the chiral pool with naturally inaccessible oxygenated skeletons.^5^ However, enantioselective C(*sp*^3^)–H bond oxidation remains an unsolved problem; chiral oxidants capable of cleaving strong unactivated C–H bonds are practically unknown outside the enzymatic world, and realization of such reaction needs to overcome major challenges regarding site-selectivity, product chemoselectivity, and enantioselectivity, because of the inherent reactivity of the oxidant, the higher reactivity of most common functional groups and of the first formed hydroxylation product when compared with unactivated C–H bonds and finally, the numerous non-equivalent C–H bonds displayed by organic molecules.^6^ All these issues have restricted the available examples of asymmetric C–H oxidation to weak, activated C(*sp*^3^)–H bonds (benzylic^7−19^ or α-to-heteroatom^20−25^) (Figure 1).

Herein we close this gap by describing the desymmetrization of dimethylcyclohexane derivatives via tertiary C(*sp*^3^)–H bond hydroxylation. We take advantage of the known ability of iron and manganese complexes to hydroxylate tertiary C–H bonds via powerful high-valent metal-oxo oxidants.^26−32^ The reaction constitutes a rather unique case of non-directed chiral functionalization of strong C–H bonds, preceded by carbene C–H insertions catalyzed by rhodium paddlewheel complexes and ketonization of monosubstituted cyclohexanes with manganese catalysts (Figure 1B).^33−35^ In the latter case, overoxidation of the first formed alcohol cannot be prevented, eliminating the corresponding hydroxylated stereocenter. The current reaction relies on a catalytic system composed of a chiral manganese complex and a phthalimido-protected amino acid coligand that activates hydrogen peroxide under mild reaction conditions.^36^ Asymmetric hydroxylation occurs in short reaction times and results in desymmetrization, generating up to four stable chiral centers in a single step, which are then susceptible to orthogonal chemical manipulation.^37−39^ Enantioselectivity is governed by the precise fitting of the cyclohexane scaffold into the active catalytic site, through a network of weak non-covalent interactions that define a lock and key interaction reminiscent of the highly ordered structures of substrate-bound enzymatic sites involved in chiral C–H hydroxylation. The broad utility of the resulting enantioenriched hydroxylated products is illustrated by their straightforward elaboration into chiral cyclohexenes, terpenoids, diol, lactone, amino acid, and macrolide precursors.^39−41^

## Results and Discussion

### Reaction Development

**Figure 2:**
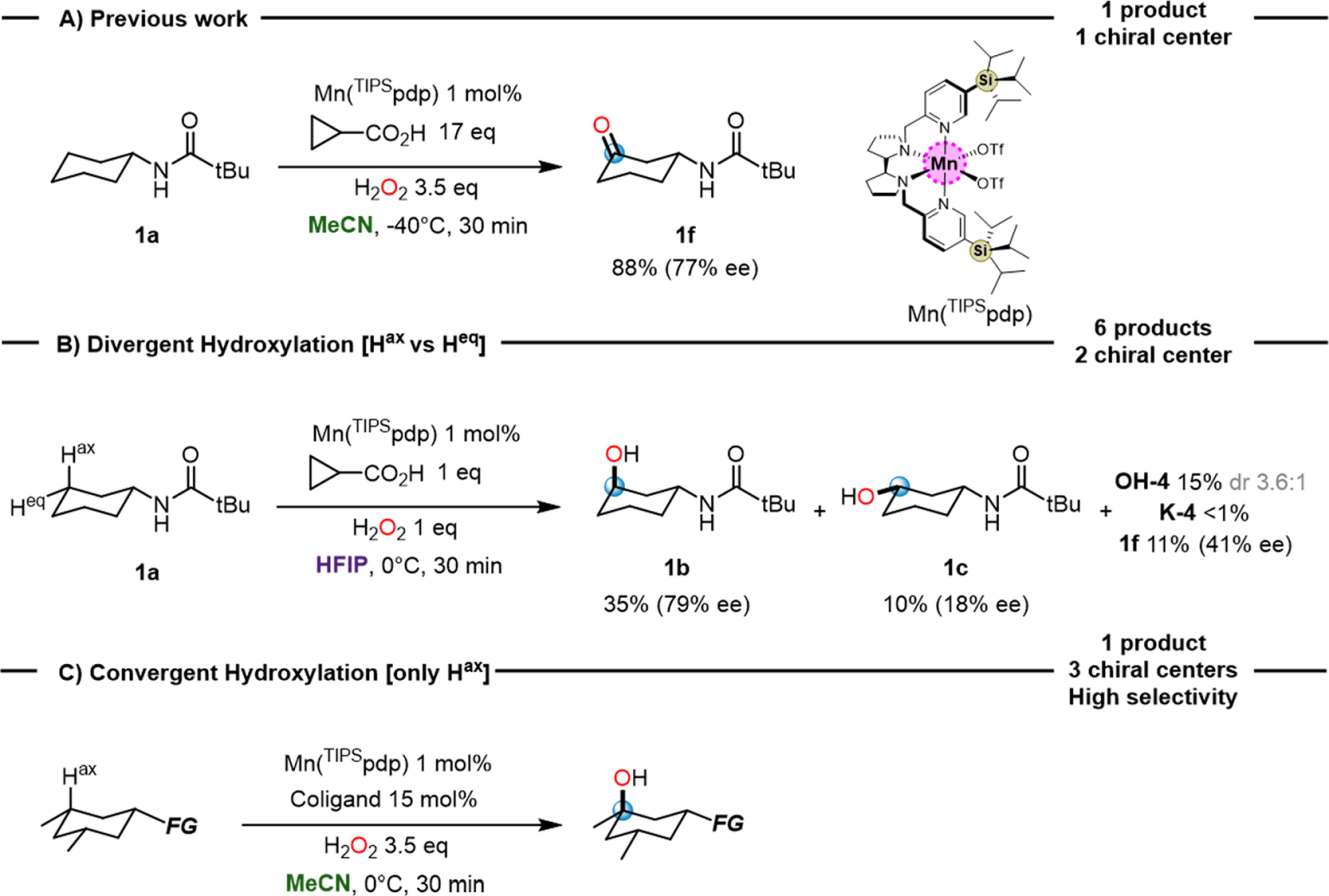
(A) Previously described chiral C3-ketonization of *N-*cyclohexylpivalamide (**1a**) in acetonitrile. (B) Enantioselective C3-hydroxylation of **1a** in HFIP. (C) Enantioselective tertiary C–H bond hydroxylation of all *cis*-3,5-dimethyl substituted cyclohexane derivatives.

In the absence of directing groups, enantioselective functionalization of unactivated C(*sp*^3^)–H bonds is rare.^7,42−46^ Previously described examples include carbene insertion reactions with rhodium catalysts,^33,47−53^ coupling of photocatalytically generated alkyl radicals with chiral Lewis acids/carboxylic acids^54−57^ and oxidation with bioinspired catalysts^34,35^ or enzymes.^58−63^ We initially considered that the site and enantioselective ketonization reaction we have recently described (Figure 2A)^34,35^ could represent a viable path toward enantioselective methylene hydroxylation, provided that overoxidation is prevented or minimized. Toward this end, we focused our attention in the use of fluorinated alcohol solvents because their strong hydrogen bond donor ability can exert a polarity reversal on the C_α_–H(OH) bonds preventing or limiting overoxidation, thus preserving the chirality obtained in the first step.^6^ Indeed, oxidation of *N*-cyclohexylpivalamide (**1a**) in 2,2,2-trifluoroethanol (TFE) and 1,1,1,3,3,3-hexafluoro-2-propanol (HFIP) using Mn(^TIPS^pdp) as the catalyst proceeds with the accumulation of the sought secondary hydroxylated products, but not surprisingly they are obtained as a complex mixture, which illustrates the challenges associated to the design of a selective C–H hydroxylation reaction in the absence of directing groups, even for relatively simple substrates where the number of non-equivalent C–H bonds is limited.^6^ When stoichiometric H_2_O_2_ was employed, four different hydroxyamides were obtained as major products, resulting from the stereoretentive axial and equatorial C–H bond hydroxylation at C3 (**1b**(OH-3 ax) and **1c**(OH-3 eq)) and C4 (**OH-4**), while the corresponding ketoamides **K-3** and **K-4** were formed in smaller amounts (Figure 2B). Most interestingly, hydroxylation at the axial C_3_–H to form **1b**(OH-3 ax) in 35% yield proceeds with high enantioselectivity (79% ee) and dominates over hydroxylation at the equatorial C_3_–H that delivers **1c**(OH-3 eq) in 10% yield and a modest 18% ee.

This analysis delineates a strategy for developing an enantioselective C–H hydroxylation reaction. Consequently, all *cis*-trisubstituted cyclohexane derivatives bearing methyl groups at C3 and C5 were selected. Besides its fundamental significance as a first example of non-directed chiral hydroxylation of a C(*sp*^3^)–H bond,^64^ such reactions will have features of important relevance in synthesis; since this class of substrates are *meso*-compounds with C1 prochiral and both C3 and C5 enantiotopic tertiary carbons, *meso*-desymmetrization by hydroxylation at these positions will provide three stereocenters in a single step. Moreover, product elaboration can provide an easy entry into different chiral motifs of interest in the construction of biologically relevant compounds.

### Optimization of the Oxidation of **3a**

**Figure 3:**
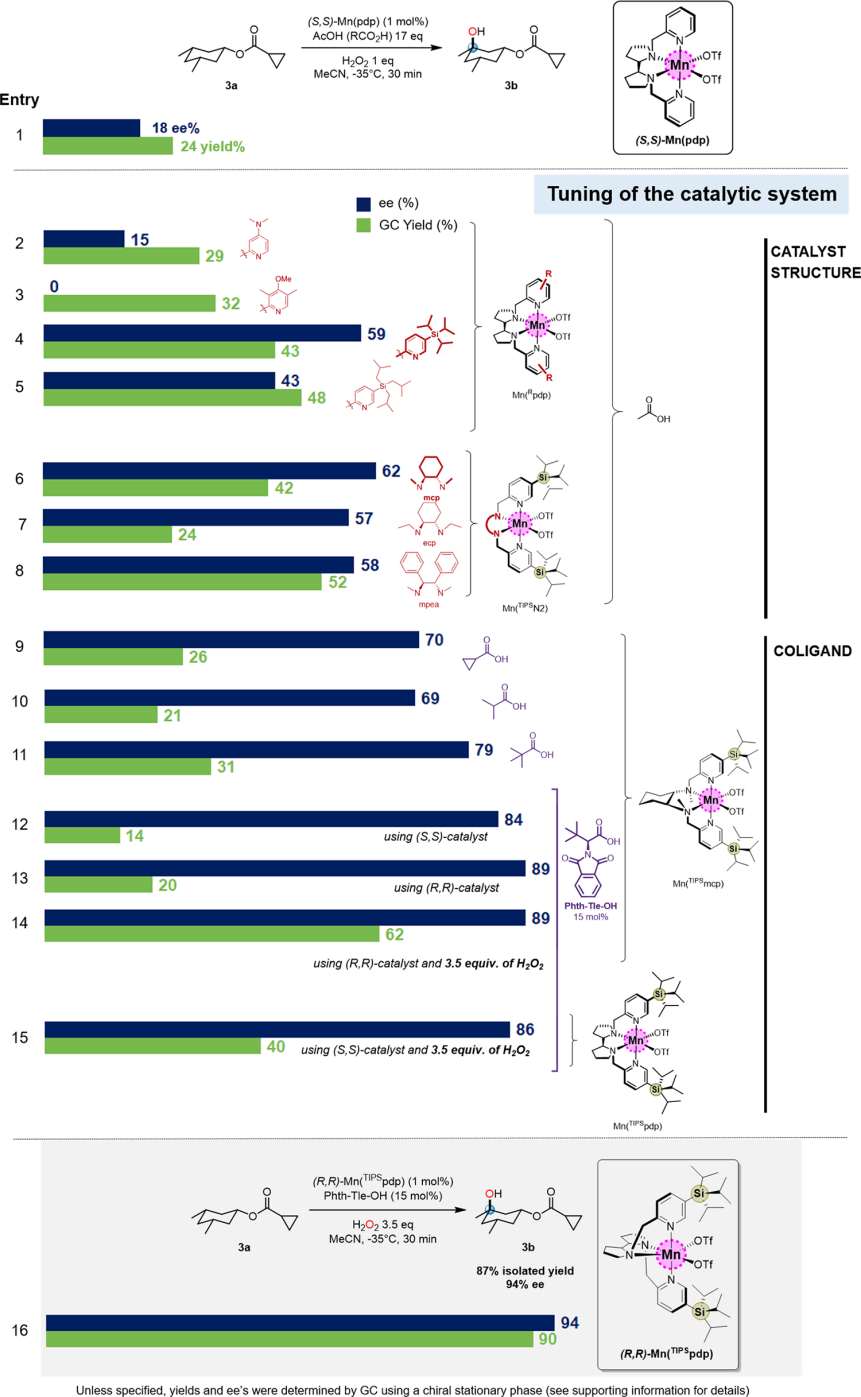
Catalyst and coligand optimization in the hydroxylation of ester **3a**.

Initial optimizations were performed oxidizing 1-(3,5-dimethylcyclohexyl)cyclopropanecarboxylate **3a** (250 mM) with 1 equiv of H_2_O_2_, delivered over 30 min by a syringe pump, in MeCN at −35 °C in vials opened to air, employing 1 mol % of a manganese catalyst and 17 equiv of a carboxylic acid or 15 mol % of an amino acid coligand. A series of catalysts in combination with different coligands and solvents were first screened (see the Supporting Information for full details). This class of manganese and iron complexes have been previously shown to be excellent C–H oxidation catalysts.^26−29,32^ Results are graphically displayed in Figure 3.

At first, we tested different manganese-based catalysts in combination with AcOH (17 equiv). Under these conditions, oxidation of **3a** using the Mn(pdp) catalyst provides the tertiary hydroxy ester **3b** in low yield and enantioselectivity (24% yield, 18% ee) and excellent mass balance. Of notice, **3b** was the only oxidation product detected, confirming that hydroxylation proceeds with stereoretention and site-selectivity. Screening entailed the use of catalysts with electron-donating substituents on the pyridine ligands (entries 2–3), and with bulky tris-(alkyl)silyl groups at position 3 of the pyridine rings (entries 4–5). The nature of the chiral diamine backbone (bipyrrolidine, *trans*-1,2-cyclohexanediamine, and 1,2-diphenylethane-1,2-diamine) and the metal (Fe vs Mn, see Supporting Information for details) was also explored. From this analysis, it was concluded that the best results in terms of yield and ee were obtained with sterically encumbered Mn(^TIPS^pdp) (entry 4)^65^ and Mn(^TIPS^mcp) (entry 6).

Different carboxylic acid coligands were then explored (entries 9–11). Interestingly, an increase in steric bulk systematically translates into an increase in enantioselectivity. Remarkably, a further increase in enantioselectivity (84% ee) was observed employing 15 mol % of *N*-phthalimido-L-*tert*-leucine (Phth-Tle-OH) and (*S,S*)-Mn(^TIPS^mcp) accompanied however by a low yield (14%) (entry 12). Since both the amino acid coligand and the catalyst are chiral, matching–mismatching effects resulting from the combination of the, respective, chiralities were also considered by using the two enantiomeric catalysts (*S,S* and *R,R*). Pleasantly, a slight increase in yield and enantioselectivity of product **3b** was observed using (*R,R*)-Mn(^TIPS^mcp), (20% yield, 89% ee) (entry 13).

Further screening involved the analysis of the nature of the amino acid side-chain and N-protecting group, which revealed Phth-Tle-OH as the leading coligand (see the Supporting Information). Employing (*R,R*)-Mn(^TIPS^mcp) and Phth-Tle-OH, the yield of **3b** could be improved to 62% using 3.5 equiv of H_2_O_2_ while retaining high enantioselectivity (89% ee) (entry 14). Most interestingly, reevaluation of the activity of (*R,R*)-Mn(^TIPS^pdp) under the new conditions provided the highest yield (90%) and enantioselectivity (94% ee) (entry 16). Of notice, employing the catalyst with the opposite absolute configuration ((*S,S*)-Mn(^TIPS^pdp)), a decrease in yield and enantioselectivity (40% yield, 86% ee, entry 15) was observed, revealing important match–mismatch effects between the respective chiralities of the catalyst and the amino acid. We remark that the unusually diverse structural versatility of both the manganese catalyst and the acid coligand are the key elements that enable the rapid evolution of the catalytic system during optimization.

### Substrate Scope

**Figure 4:**
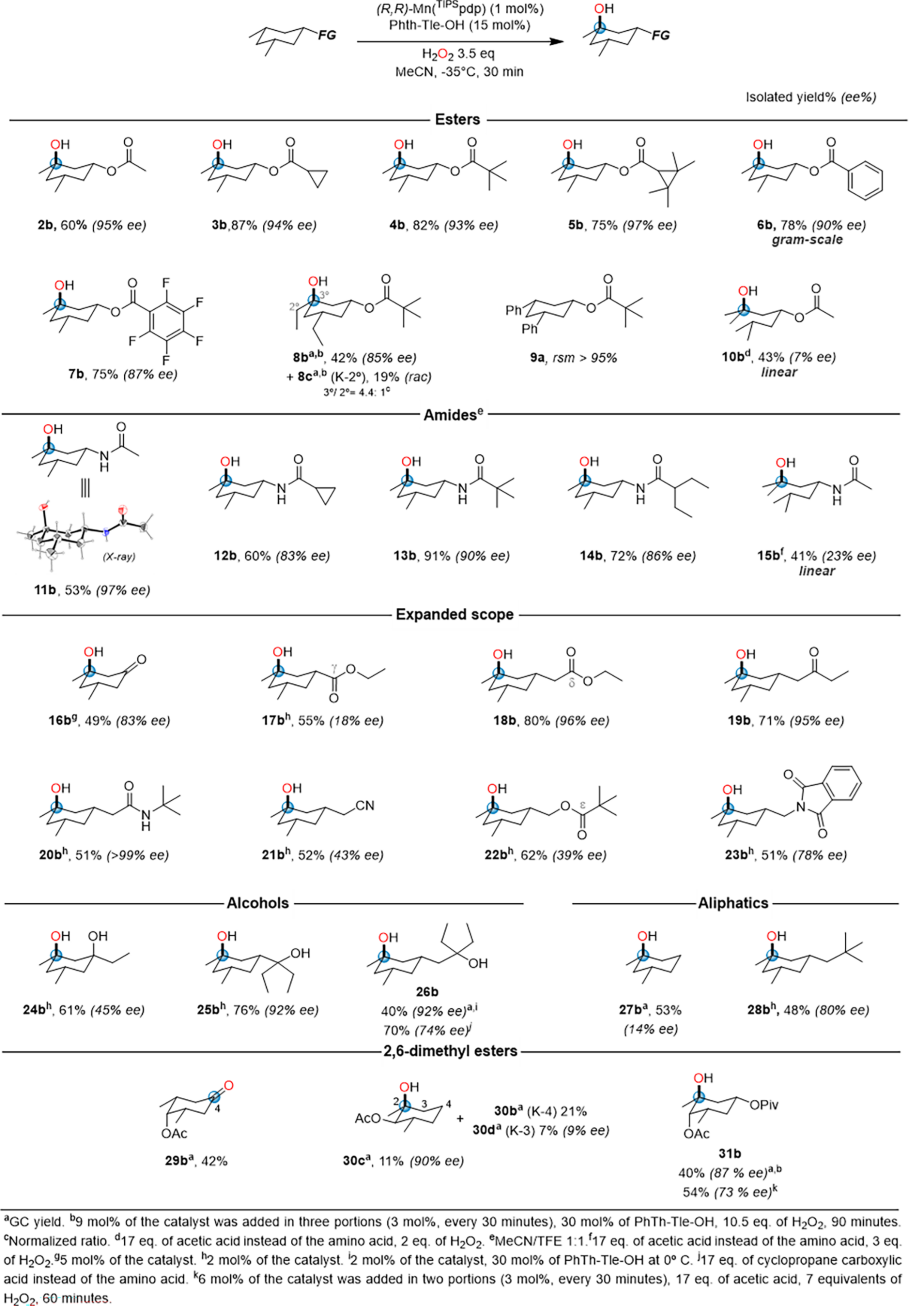
Substrate scope of the enantioselective hydroxylation.

With the optimum conditions in hand, we then extended our study to other *cis*-3,5-dimethyl cyclohexane derivatives bearing different groups at C1 (Figure 4). We were pleased to see that the reaction shows an unusually large substrate scope for a C–H functionalization reaction; esters, amides, imides, ketones, nitriles, and tertiary alcohols are all well tolerated, affording the corresponding hydroxylated products in good isolated yields and good to excellent enantioselectivities.

Oxidation of acetate ester **2a** proceeds with good isolated yield (60%) and excellent enantioselectivity (95% ee), and by increasing the steric hindrance of the acyl group (**3a**–**5a**), a pronounced improvement in yield up to 87% was obtained, while retaining high enantioselectivity (93–97% ee). The ester scope can be satisfactorily extended to benzoyl-substituted derivatives (**6a–7a**) demonstrating the tolerance of the system to aromatic groups. A gram-scale hydroxylation employing **6a** highlights the efficiency and practicality of the current methodology, affording **6b** in 78% isolated yield and 90% ee, with no trace of products deriving from aromatic oxidation. A decrease in electron density of the aryl ring as in **7a** also led to similar results (75% isolated yield and 87% ee).

The nature of the substituents in positions 3 and 5 of the cyclohexane motif was also explored. A more complex picture was observed in the oxidation of ester **8a** bearing *cis*-diethyls. We were pleased to see that tertiary alcohol **8b** is still the major product (42% yield), but oxidation of the methylenic site in the ethyl chain provides ketoester **8c** as a side product (19% yield), presumably because of the larger steric demand of the endocyclic tertiary C–H bond when compared with the exocyclic methylenic ones. Of interest, **8b** is obtained in high enantioselectivity (85% ee), which contrasts with the racemate obtained for **8c**. On the other hand, complete recovery of the starting material was observed in **9a** bearing *cis*-diphenyls in the cyclohexane ring, reasonably due to competitive aromatic oxidation that deactivates the catalytic system.^36,66^

Amide substrates (**11a**–**14a**) are also hydroxylated in high isolated yields (up to 91%) and enantioselectivies (83–97% ee). As for the ester derivatives, replacement of acetyl by bulkier acyl groups leads to improvements in reaction yield, while retaining high enantioselectivity.

The conformationally free and therefore more challenging for enantiodiscrimination linear substrates (ester **10a** and amide **15a**) were also tested. Site-selective tertiary C–H hydroxylation was observed in moderate yields (up to 43% yield), and low (7% ee) or moderate (23% ee) enantioselectivities were observed for hydroxyester **10b** and hydroxyamide **15b**, respectively.

We then moved to explore the role of different carbonyl-based groups. The simpler *cis*-3,5-dimethyl cyclohexanone (**16a**) is a particularly challenging substrate because of the electronic deactivation determined by the carbonyl group. In addition, it has a lower degree of structural complexity that makes chiral hydroxylation particularly challenging. Interestingly, oxidation of **16a** delivered hydroxy ketone **16b** in relatively high ee (83%) and moderate yield (49%). It is notable that this chiral motif makes **16b** particularly attractive as a versatile building block for subsequent synthetic elaboration.

In **17a**, the ethoxycarbonyl group places the carbonyl moiety in γ to the reactive tertiary C–H bonds, and rather unexpectedly a pronounced decrease in enantioselectivity is observed (18% ee). However, when a methylene is placed between the carbonyl and the ring as in **18a** high enantioselectivity is restored (96% ee), and the product is obtained in high isolated yield (80%). To complete the series, we also tested ketone **19a** and amide **20a**, both displaying a methylene spacer between the carbonyl group and the cyclohexane ring. Remarkably, in both cases the corresponding hydroxylated products were obtained in satisfactory yields (71 and 51%) and excellent enantioselectivities (95 and >99% ee).

Replacement of the δ-carbonyl moiety by a cyano group can be also tolerated but the corresponding hydroxy nitrile **21b** is obtained with moderate yield (52%) and enantioselectivity (43% ee). Furthermore, in ester **22a**, the pivaloyl group is shifted by an additional methylene unit from the cyclohexane core and, compared to **4a**, this leads to a decrease in enantioselectivity (39% ee). However, with a more rigid phthalimide group as in **23a**, enantiodiscrimination is substantially restored (78% ee).

Despite the presence of a δ-carbonyl functionality ensures high enantioselection, this can be also accomplished with substrates devoid of this group. Tertiary alcohols **25a** and **26a** are hydroxylated with moderate to high yields (up to 76%) to the corresponding chiral 1,4- and 1,5-diols **25b** and **26b**, respectively, with excellent enantioselectivities (92% ee). On the other hand, with **24a** the chiral diaxial 1,3-diol **24b** is obtained in 61% yield and 45% ee.

Particularly interesting is the application of the current system to hydrocarbons. The simplest *cis*-1,3-dimethyl cyclohexane **27a** is oxidized to **27b** in low 14% ee, highlighting the importance of the group at C1 in defining enantiodiscrimination. However, oxidation of the C1-neopentyl substituted **28a** proceeds with remarkably high enantioselectivity (80% ee) representing the only example reported so far of a non-enzymatic hydroxylation of a strong hydrocarbon C–H bond that proceeds with high enantioselectivity.

The oxidation of *cis*-2,6-dimethyl substituted cyclohexanes proved to be more challenging but was also shown to provide an entry into functionalized cyclohexanes with up to four stereocenters (Figure 4 bottom).^60^ Oxidation of **29a**, containing an axial acetyl group, occurs exclusively at the C4 methylenic site, delivering ketoester **29b** in 42% yield, leaving the tertiary C–H bonds intact. A more complex picture was observed in the oxidation of its diastereoisomer **30a**, bearing an equatorial ester moiety. C4 ketoester **30b** is still the major product, but C2 hydroxy ester **30c** and C3 ketoester **30d** are obtained as side products. Of interest, **30c** is obtained in high enantioselectivity (90% ee), which contrasts with the poor ee’s obtained for **30d**. Tentatively, this can be attributed to oxidation at C3 being initiated by hydrogen atom transfer (HAT) at the equatorial C–H, which appeared to be a low enantioselective path during the optimization studies on **1a** (Figure 2B).

Based on the results obtained with **29a**, we envisioned that installing a bulky equatorial ester group at C4 could block ketonization at C3 (and C4), without affecting hydroxylation enantioselectivity at the C2/C6 tertiary axial C–H bonds. In line with expectations, with 1,4-diester **31a**, selective tertiary axial C–H bond hydroxylation was observed delivering **31b** in moderate yield (40%) but high enantioselectivity (87% ee). Interestingly, **31b** is a complex and densely functionalized molecule, where four of the six carbons of the cyclohexane core are stereocenters. Furthermore, **31b** also represents an interesting molecule in medicinal chemistry because the removal of the ester groups provides the carbocyclic analogue^38^ of the rare deoxysugar α-axenose, present in the anticancer drug trioxacarcin A.^37^

### Computational Analysis of the Origin of Enantioselectivity

**Figure 5:**
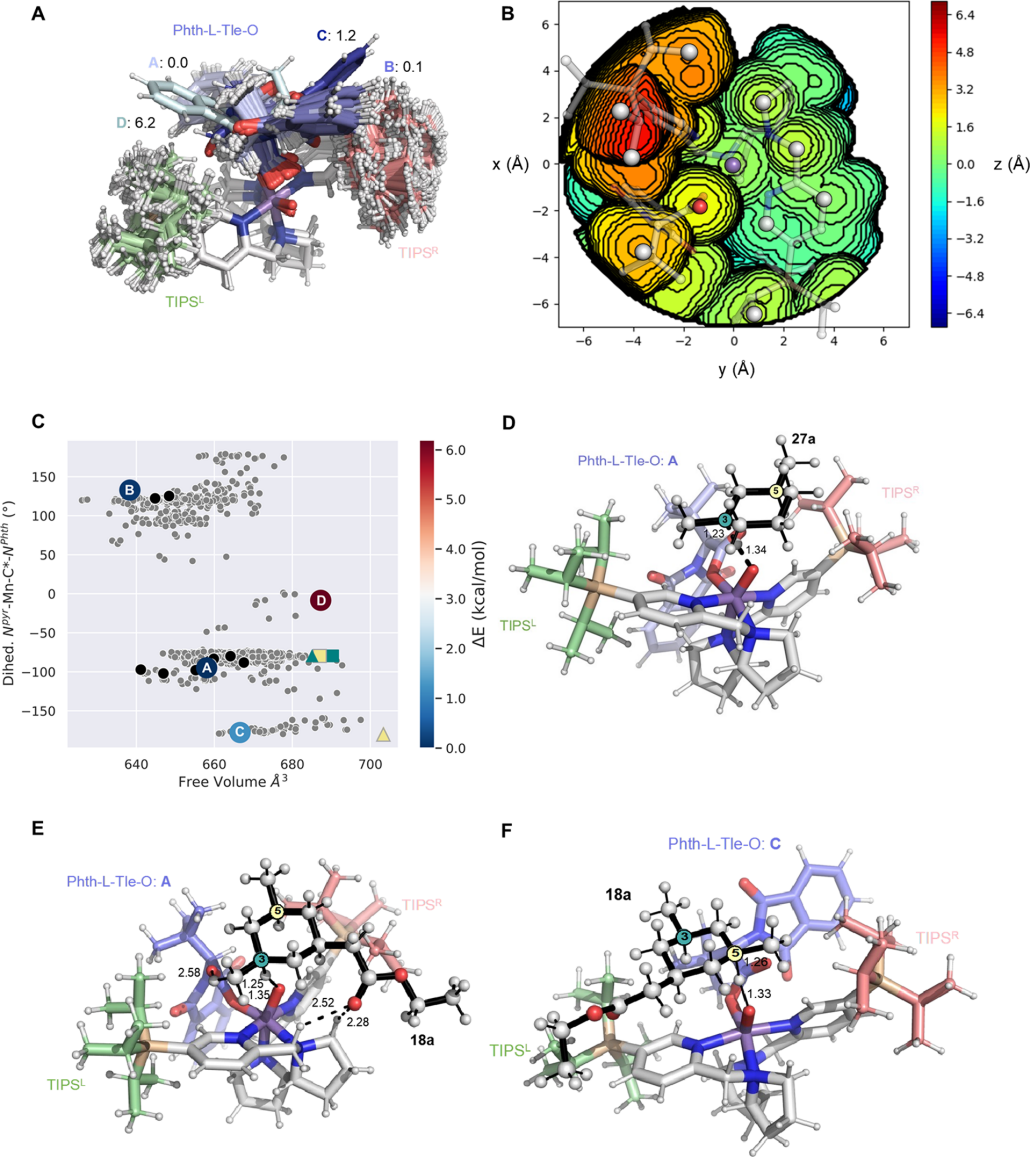
(A) Overlay of the CREST-generated conformational ensemble of the free (*R,R*)-Mn(^TIPS^pdp) catalyst. The most stable conformations A–D of the catalyst corresponding to the different positioning of the phthalimide group of the Phth-Tle-O coligand are highlighted using different scales of blue color. The relative stabilities are also indicated in kcal/mol. (B) Steric map of the lowest in energy conformation A of the free catalyst, showing the available volume for allowing substrate binding. (C) Representation of the 1000 structures (gray points) generated with CREST for the free catalyst based on a selected dihedral angle that differentiates the A–D conformations of the phthalimide group of the coligand (N^pyr^-Mn-C*-N^Phth^, *y* axis) and the available volume (in Å^3^) for substrate binding (*x* axis). Conformations A–D are marked in the plot and colored according to their relative energies. Black points correspond to the 14 structures obtained after the second clusterization procedure. Free catalysts for HAT TSs are represented with triangles for **18a** and squares for **27a**; C-3 hydroxylation is highlighted in teal, and C-5 hydroxylation in yellow. (D–F) Lowest in energy HAT TSs for substrates: (D) **27a** for C-3, (E) **18a** for C-3, and (F) **18a** for C-5 hydroxylation are shown. All distances are given in Å. The catalyst is represented in sticks using the following coloring scheme: coligand in blue, TIPS^R^ group in pink, and TIPS^L^ in green. Substrates **27a** and **18a** are displayed in spheres and black sticks, and the key C-3 and C-5 carbons are highlighted in teal and yellow, respectively.

The structural and dynamical evaluation of the optimized (*R,R*)-Mn(^TIPS^pdp) catalyst was performed by a combined computational analysis based on Conformer-Rotamer Ensemble Sampling Tool (CREST) software,^67^ geometry-aware clusterization schemes, and density functional theory (DFT, see the Supporting Information for details). The ensemble of structures generated by CREST revealed three different regions that define the catalytic site: the rigid main structural ligand skeleton bonded to Mn in the center; the two bulky tris-isopropylsilyl (TIPS, named TIPS^R^ and TIPS^L^ in Figure 5A) substituents that form two large steric barriers at the two sides of the catalyst; and finally, the bound Phth-Tle-O ligand, displaying a high level of flexibility that easily adapts for substrate binding (Figure 5A–C). It should be noted that four distinctive conformations (1–4 in Figure S3) of the catalyst could be obtained corresponding to the two possible rotations of the carbonyl and Phth groups, represented as Newman projections through the C_α_–CO_2_ bond of the Mn-bound Phth-L-Tle-O ligand. In 1–2, the two groups are eclipsed with Mn=O and in 3–4 are in anti. However, our CREST-based conformational analysis was restricted to eclipsed conformations 1–2, as we found that the difference in energy between syn and anti was higher than 11 kcal/mol at the UM06-L-D3 level of theory. Additionally, the transition state (TS) obtained for HAT when the carbonyl group of Phth-Tle-O and the Mn=O are in anti for substrate **18a** presents a substantially larger activation barrier (difference of more than 7 kcal/mol with respect to 2, see Table S11). This is in line with previous studies indicating a weak O–O interaction between the unpaired p electron of the Mn=O and the lone pair of the carbonyl group of the substrate in both a similar manganese- and iron-based catalyst,^68,69^ which we find to be crucial for effective HAT. The CREST-based ensemble of the free catalyst in 1–2 conformations revealed that the coligand can adopt four main conformations of the phthalimide group (as shown in Figures 5A–C, S4): the lowest in energy conformation A in which the *tert*-butyl group of the coligand is situated close to the carbonyl group, B that is only 0.1 kcal/mol higher in energy and instead presents the phthalimide group located close to the carbonyl, and finally C–D at 1.2 and 6.2 kcal/mol, respectively, in which Phth is establishing some C–H·· π interactions with either the TIPS^R^ group (C) or the TIPS^L^ (D) (see Table S10). Consequently, our analysis suggests that the shielding exerted by the TIPS groups and especially the conformation of the amino acid coligand shape the catalyst’s reaction site and determines the available volume for substrate access for asymmetric hydroxylation (see Figures 5B,C, S5).^70^

Intrigued by how the different conformations of Phth shape the catalyst active site and impact the recognition and interaction with the substrate for asymmetric hydroxylation, we located the HAT TSs and reaction complexes (RCs) corresponding to hydroxylation at positions C-3 and C-5 for the model substrates **18a** and **27a** (Figure 5D–F). The optimized TSs and RCs for both C-3 and C-5 were then subjected to CREST analysis for finding additional lower in energy conformations. Interestingly, despite starting from different initial structures for both C-3 and C-5 the lowest in energy RC found positions **18a** in such a way that C-3 is better oriented for hydroxylation (i.e., shorter distance and better angle). The proper positioning of **18a** for asymmetric hydroxylation is mostly attributed to the CH··π interaction observed between the methyl group at C-3 and the pyridine ring (see Figures S6, S7, S9). In this RC, the carbonyl group of the C-1 substituent of **18a** is interacting with the TIPS^R^ group. Both interactions contribute to properly position C-3 close to the Mn=O moiety for efficient hydroxylation. In this RC of **18a**, the methyl substituent of the cyclohexane scaffold at C-5 is located between the isopropyl group of TIPS^R^ and the *tert*-butyl of the coligand. It should be also highlighted that in this lowest in energy RC, the phthalimide group of the coligand adopts the most stable conformation A as described above.

The lowest in energy HAT TS for C-3 maintains this network of weak interactions, except for the functional group at C-1 that is establishing a hydrogen bond between the carbonyl group of the ester and both the hydrogen atom at C-2 of one of the pyrrolidine rings and the pyridin-2-ylmethyl hydrogen. This HAT TS for C-3 has an O–H–C distance of 1.35 and 1.25 Å for O–H, and H–C, respectively (see Figure 5E). The Mn–O–C angle is ca. 131°. The computed available volume at this C-3 HAT TS is 685.2 Å^3^ (see teal triangle in Figures 5C, and S13). The computed HAT activation barrier from the lowest in energy RC for asymmetric hydroxylation at C-3 is 3.8 kcal/mol. Interestingly, the lowest in energy TS found for C-5 presents a HAT activation barrier of 7.2, which is 3.4 kcal/mol higher than the one obtained for C-3 (see Table S11). The TS for C-5 asymmetric hydroxylation presents a completely different orientation of **18a** with respect to that for C-3, and a distinct conformation of the phthalimide group with respect to C-3 (see Figure 5F). In fact, the phthalimide group adopts conformation C (that is 1.2 kcal/mol higher than A for the free catalyst), thus establishing C–H·· π interactions with the TIPS^R^ group. The computed volume at this HAT TS for C-5 hydroxylation is substantially increased to 703.4 Å^3^ (see the yellow triangle in Figures 5C, S13). In this TS for C-5, none of the above-mentioned interactions observed for C-3 are possible: the carbonyl ester of the C-1 substituent of **18a** is not making hydrogen bond interactions with the pyrrolidine and pyridin-2-ylmethyl hydrogens, pointing instead toward the TIPS^L^ group; the C-3 methyl group is located in the space left by the conformational change of the phthalimide group of the coligand, and therefore can no longer establish a CH ··π interaction with the pyridine ring. These differences in the network of weak interactions between C-3 and C-5 indicate that the Mn=O catalyst has an active site shape and volume that is preorganized and optimal for enantioselective hydroxylation at C-3.

Our predicted substrate-bound catalyst pose is also in line with the experimental trends obtained for the different substrates tested. The study using the same methodology of the simplest compound **27a** lacking any substituent at C-1 indicates that the enantioselectivity of the process is reduced as both C-3 and C-5 can adopt a similar pose and establish the C–H·· π interaction between one of the methyl groups and the pyridine ring (see Figures 5D, S8, S10). The TS for hydroxylation at C-3 allows the methyl substituent at C-5 to establish weak dispersion interactions between the isopropyl group of TIPS^R^ and the *tert*-butyl of the coligand (which is not possible for the C-5 pose). The difference in the activation energy obtained for HAT at C-3 and C-5 for **27a** is reduced to 0.1 kcal/mol, in this case in favor of C-5 hydroxylation (at the UM06-L-D3/6-31G(d)(Mn-Def2SVP) level C-3 hydroxylation is favored by ca. 0.5 kcal/mol). Interestingly, the calculation of the available volume of the catalyst at the HAT TS for C-3 and C-5 of **27a** is in both cases ca. 690 Å^3^ (which is similar to the one found for the C-3 HAT TS for substrate **18a**, see squares in Figure 5C). Substrate **28a** that contains a neopentyl group in C-1 can still potentially adopt the same binding pose as **18a**, however, the lack of the ester group at C-1 avoids the potential hydrogen bond interaction, which is compensated by the dispersion interactions established with the TIPS^R^ and the catalyst scaffold (see Figure S12). The same explanation holds for substrate **17a** that, compared to **18a**, lacks the methylene spacer, thus preventing the establishment of the hydrogen bond interaction of the carbonyl ester group and the pyrrolidine methyl and pyridin-2-ylmethyl (see Figure S11). In both **28a** and **17a**, the favorable interaction of the C-3 methyl with the pyridine ring is maintained only in the C-3 pose. Altogether, our computational predictions indicate that despite the high conformational flexibility of the TIPS and Phth-Tle-O coligand of the (*R,R*)-Mn(^TIPS^pdp) catalyst that impacts the available volume for productive substrate binding, the most stable conformations present an active site that is highly complementary to the cyclohexane-based scaffold for enantioselective hydroxylation at C-3.

### Elaboration of the Hydroxylated Products

**Figure 6:**
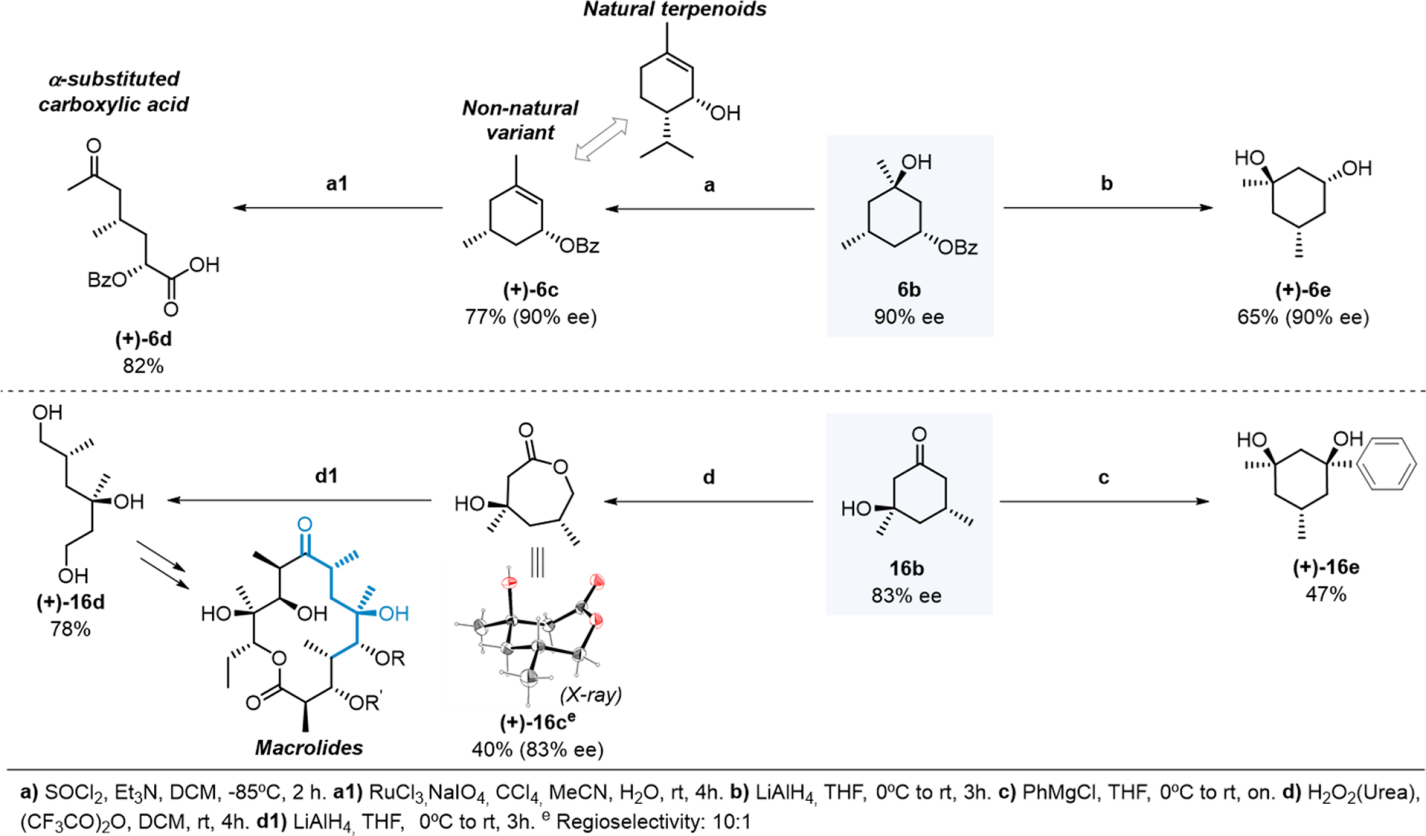
Illustrative examples of product elaboration.

Four different pathways were pursued for illustrating the straightforward chemical elaboration of the chiral hydroxylation products (Figure 6). Path **a** describes the manipulation of the tertiary alcohol functionality in product **6b**. Chiral trisubstituted cyclohexene **6c** (bearing a valuable allylic ester motif and two chiral centers)^71^ could be prepared in 87% yield by dehydration. In terms of step economy, this compares favorably with the six-step synthesis reported so far for the free alcohol (intermediate in the total synthesis of didemnaketals).^72^ Furthermore, chiral cyclohexenes such as **6c** are interesting because they may be considered as non-natural variants to naturally occurring terpenoids such as carene, pulegol, and piperitol, which find wide utility in the total synthesis of natural products and may thus represent an important addition to the chiral pool.^5,73^ Elaboration of the olefinic site opens numerous paths for straightforward diversification. Illustrating these possibilities, oxidative C=C bond cleavage in **6c** provides the linear α-acyloxy carboxylic acid **6d** in 82% yield. This synthetic route extended to cyclohexenes with different allylic ***O***, ***N***, or ***C*** motifs, provides general access to α-substituted carboxylic acids, including protected non-natural amino acids with two chiral centers and a terminal acetyl moiety.

On the other hand, paths **b**, **c**, and **d** exemplify manipulation of the C1 moiety. A chiral 1,3-diol could be prepared by removal of the *–Bz* group at C1 in **6b** providing **6e** in 65% yield (path **b**) bearing three chiral centers.

Moreover, by nucleophilic addition to the C1 carbonyl group in ketoalcohol **16b** (path **c**), diaxial 1,3-diol **16e** bearing two valuable chiral quaternary centers could be obtained in 47% yield.^74^ Ketoalcohol **16b** could also be converted through Baeyer–Villiger oxidation to the 7-membered ring lactone **16c** (path **d**), leading to increased molecular complexity in an oxidation fashion, keeping the stereogenic centers untouched. Manipulation of chiral 7-membered ring lactones offers numerous paths for diversification into interesting oxygenated chains. This was exemplified by the reduction of **16c**, to afford linear polyol **16d** in 78% yield, displaying a typical chiral 1,3 pattern present in numerous macrolides.^41^

## Conclusions

The current work describes the first example of non-directed and non-enzymatic catalytic enantioselective hydroxylation of unactivated tertiary C(*sp*^3^)–H bonds. Theoretical analysis of the origin of the enantioselectivity uncovers that chiral recognition relies on a synergistic interplay of weak interactions and structural complementary between the substrate and the catalyst that resemble lock and key recognition phenomena operating in enzymatic sites. Furthermore, the use of catalysts based on an earth-abundant metal singularizes the current system with respect to state-of-the-art asymmetric C(*sp*^3^)–H functionalization reactions that rely on precious metals. In addition, the use of hydrogen peroxide as an oxidant confers the system a high atom economy, which combined with the mild experimental conditions makes the reaction particularly appealing from a sustainability perspective.

Collectively, the work delivers proof of concept of the powerful reach of biomimetic oxidation catalysts as unique tools for manipulating C–H bonds as functional groups, valorizing simple organic molecules by means of their conversion into chiral, stereochemically rich, and versatile oxidized products, expanding in a virtually unlimited manner the available chiral pool. Finally, the high structural versatility of the catalytic system is a key aspect that enables the rapid evolution of the reaction from modest to outstanding yields and enantioselectivities. We foresee that such versatility will find utility in the development of novel enantioselective C–H functionalization reactions.

## Supporting Information Available

Materials and methods; catalysts used in this work; catalysts screening and optimization of reaction conditions; free volumes for the substrate; generated CREST ensembles; volume calculations; relative energies between the conformations; different optimized HAT transition states; CREST ensembles for the reactant complexes; non-covalent interactions; steric maps of the free catalyst; crystallographic data; NMR spectra; SFC and GC traces; and description of the computational approaches (PDF)
