# Supplementary material for: C–H Bonds as Functional Groups: Simultaneous Generation of Multiple Stereocenters by Enantioselective Hydroxylation at Unactivated Tertiary C–H Bonds
Source: J Am Chem Soc. 2023 Jul 11;145(29):15742–53. doi: 10.1021/jacs.2c10148 (PMC10651061; doi:10.1021/jacs.2c10148)
Supplement: Supplementary file 1 — ja2c10148_si_001.pdf [file ja2c10148_si_001.pdf]

Supplementary Materials for

C-H Bonds as Functional Groups. Simultaneous Generation of Multiple Stereocenters by Enantioselective Hydroxylation at Unactivated Tertiary C-H Bonds

Andrea Palone, Guillem Casadevall, Sergi Ruiz-Barragan, Arnau Call, Sílvia Osuna,* Massimo Bietti,* Miquel Costas.*

Corresponding author. Email: silvia.osuna@udg.edu, bietti@uniroma2.it, miquel.costas@udg.edu.

Table of Contents

1.1 Materials 6

1.2 Instrumentation 6

1.3 Synthesis of the substrates 7

1.3.1 Synthesis of ester substrates 7

Esters synthesis protocol A: 7

Esters synthesis protocol B: 8

Synthesis of 8a 11

Synthesis of 9a 13

Synthesis of 10a 14

Amides synthesis protocol 17

Synthesis of 15a 19

1.3.2 Synthesis of substrates 17a – 28a 21

Synthesis of 17a 21

Synthesis of 22a 23

Synthesis of 23a 23

Synthesis of 21a 24

Synthesis of 19a 24

Synthesis of 18a 25

Synthesis of 20a 26

Synthesis of 24a 27

Synthesis of 25a 27

Synthesis of 26a 28

Synthesis of 28a 28

1.3.3 Synthesis of substrates 29a – 31a 30

Synthesis of 29a 30

Synthesis of 30a 30

Synthesis of 31a 31

1.4 Synthesis of the complexes 34

Figure S1. *Schematic representation of the catalysts used in this work* 34

1.4.1 Synthesis of (*S,S*)-Mn(^{TIPS}mpea) 35

1.5 Oxidation reactions 36

1.5.1 Reaction protocol for catalysis method A 36

1.5.2 Reaction protocol for catalysis method B 38

1.5.3 General Procedure for product isolation 39

1.5.4 **Synthesis of oxidation products** 39
Synthesis of **1b(OH-3 ax) and 1c(OH-3 eq)** 39
Synthesis of **1e(OH-4 eq) and 1d(OH-4 ax)** 41
1.5.5 **Reaction oxidation of 1a** 44
Table S1. *Catalysts screening of oxidation of 1a in different solvents* 44
1.5.6 **Reaction optimization for oxidation of 3a** 45
Table S2. *Catalyst screening.* 45
Table S3. *Coligand screening.* 46
Table S4. *Catalyst screening using AAs as coligand.* 47
Table S5. *Optimization of Catalyst and AAs as coligand loading.* 48
Table S6. *Optimization of substrate concentration.* 49
Table S7. *Protecting group screening of L-tert-leucine coligand.* 50
Table S8. *Side-chain screening of Phthalimido-protected aminoacid.* 51
Table S9. *Oxidation of* ***3ax***[a] 52
1.5.7 **Characterization of oxidation products** 53
Oxidation of 8a 56
Oxidation of 9a 57
Oxidation of 10a 57
Solid state structure of ***(1R,3R,5S)-11b*** 58
Crystal data of *(+)*-***(1R,3R,5S)-11b.*** 59
Oxidation of 26a 66
Oxidation of 27a 66
Oxidation of 29a 67
Oxidation of 30a 68
Oxidation of 31a 69
1.6 **Elaboration of tertiary alcohol** 71
Synthesis of 6c 71
Synthesis of 6d 71
Synthesis of 6e 72
Synthesis of 16c 73
Solid state structure of ***16c*** 74
Crystal data of ***16c.*** 74
Synthesis of 16d 75
Synthesis of 16e 76
1.7 **NMR Spectra** 77
1.8 **SFC and GC traces** 197

Derivatization of product 15b 197

Derivatization of product 19b 198

Derivatization of product 21b 199

1.9 Computational Approaches 236

Figure S2. *Computational pipeline used based on the combination of the CREST program, geometry-based clusterization procedure, single point (SP) energy calculations and geometry optimizations at the UM06-L-D3 level of theory.* 238

1.10 Computational Analyses 239

Rationalization of the conformational flexibility and preorganization of the Mn-catalyst 239

Figure S3. *Newman projections through the Cα-CO2 bond of the Mn-bound Phth-Tle-O ligand.* 239

Figure S4. *Generated CREST ensembles for the free catalyst: the left image corresponds to the first 1000 structures generated from CREST at the xTB level, the middle figure shows the reduced number of conformations based on xTB relative energies and after applying the first clusterization protocol (136 structures), and the right panel shows the 14 lowest in energy conformations at the DFT level found in the second clusterization step.* 239

Figure S5. *Volume calculations of the A-F conformations of the free catalyst: (A) the lowest in energy conformation in which the tert-butyl group of the coligand is situated close to the carbonyl group, (B) that is only 0.1 kcal/mol higher in energy and instead presents the phthalimide group located close to the carbonyl, (C) at 1.2 kcal/mol in which the Phth is establishing C-H·· π interactions with the TIPS^R group, (D) at 6.2 kcal/mol where the Phth is establishing C-H·· π interactions with the TIPS,^{L} and (F) at 11.3 kcal/mol that presents the carbonyl group of the coligand in anti with respect to the Mn=O.* 241

Table S10. *Relative energies between the conformations for the catalyst in the absence of any substrate. Details of electronic method are described in the method section above. All energies are expressed in kcal/mol.* 241

Rationalization of the origins of the enantioselectivity in the catalyst substrate complexes and transitions states 241

Table S11. *Relative energy between the different optimized HAT transition states for substrates **18a** and **27a**. Details of electronic method described in the method section. Energies in kcal/mol.* 241

Figure S6*. CREST ensembles for the reactant complexes of substrate **18a** for (A) C-3 and (B) C-5. Right image conformations are obtained from the first clusterization step of the protocol. Left image conformations are obtained from the second clusterization step of the protocol.* 242

Figure S7*. CREST ensembles for the HAT transition states of substrate **18a** for (A) C-3 and (B) C-5. Right image conformations are obtained from the first clusterization step of the protocol. Left image conformations are obtained from the second clusterization step of the protocol.* 242

Figure S8*. CREST ensembles for the HAT transition states of substrate **27a** for (A) C-3 and (B) C-5. Right image conformations are obtained from the first clusterization step of the protocol. Left image conformations are obtained from the second clusterization step of the protocol.* 243

Figure S9. *Non-covalent interactions obtained by NCIPLOT for the HAT transition state of **18a** for (A) C-3, and (B) C-5.* 243

Figure S10. *Non-covalent interactions obtained by NCIPLOT for the HAT transition state of **27a** for (A) C-3, and (B) C-5.* 244

Figure S11. *CREST ensembles for the reactant complexes of substrates* ***17a*** *for (A) C-3 and (B) C-5. Right image conformations are obtained from the first clusterization step of the protocol. Left image conformations are obtained from the second clusterization step of the protocol.* 244

Figure S12. *CREST ensembles for the reactant complexes of substrates* ***28a*** *for (A) C-3 and (B) C-5. Right image conformations are obtained from the first clusterization step of the protocol. Left image conformations are obtained from the second clusterization step of the protocol.* 245

Figure S13. *Steric maps of the free catalyst for the HAT transition state superimposed with* ***18a*** *for (A) C-3 hydroxylation with the catalyst in conformation A, and (B) C-5 hydroxylation with the catalyst in conformation C.* 245

1.11 References.. 246

1.1 Materials

Reagents and solvents used were of commercially available reagent quality unless stated otherwise. Solvents were purchased from SDS, Scharlab, Fluorochem. Solvents were purified and dried by passing through an activated alumina purification system (M-Braun SPS-800) or by conventional distillation techniques.

1.2 Instrumentation

Oxidation products were identified by comparison of their GC retention times and GC/MS with those of authentic compounds, and/or by ^{1}H and ^{13}C-NMR analyses. Xray diffraction analysis were carried out on a BRUKER SMART APEX CCD diffractometer using graphite-monochromated MoKα radiation (λ = 0.71073 Å) from an X-ray Tube. NMR spectra were taken on BrukerDPX400 spectrometers using standard conditions. Electrospray ionization mass spectrometry (ESI-MS) experiments were performed on a Bruker Daltonics Esquire 3000 Spectrometer using a 1 mM solution of the analyzed compound. High resolution mass spectra (HRMS) were recorded on a Bruker MicroTOF-Q II (Q-TOF) instrument with a ESI source at Serveis Tècnics of the University of Girona. Samples were introduced into the mass spectrometer ion source by direct infusion through a syringe pump and were externally calibrated using sodium formate. Optical rotations were measured at room temperature (25° C) using a Jasco P-2000 iRM-800 polarimeter. Concentration is expressed in g/100 mL. The cell was 10 cm long with a 1 mL of capacity. Chromatographic analyses were performed on an AgilentGC-7820-A chromatograph using a HP5 column (30 m) while enantiomer resolution using HP-Chiral-20B, J&W CYCLOSIL-B columns and supercritical fluid chromatography (SFC) were performed on an Agilent 1260 Infinity II SFC System using CHIRALPAK IA-3, CHIRALPAK IB-3, CHIRALPAK IC-3, CHIRALPAK IG-3 columns.

1.3 Synthesis of the substrates

The following substrates were obtained from a commercially available mixture of isomers of 3,5-dimethylcyclohexan-1-ol, previously separated following a modified literature procedure, by flash chromatography over silica using hexane:ethyl acetate 10:1. Obtaining the two diastereoisomers (1α, 3α, 5α)-3,5-dimethylcyclohexan-1-ol (**A**) and (1β, 3α, 5α)-3,5-dimethylcyclohexan-1-ol (**B**).[1,2]

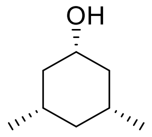

(1α,3α,5α)-
3,5-dimethylcyclohexan-1-ol
A

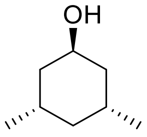

(1β,3α,5α)-
3,5-dimethylcyclohexan-1-ol
B

1.3.1 Synthesis of ester substrates

The following ester substrates were obtained starting from the alcohol **A**:

2a, **3a**, **4a**, **6a** and **7a** were obtained using *ester synthesis protocol A*, **5a** were obtained using *ester synthesis protocol B.*

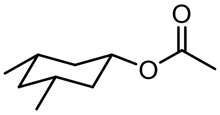

2a

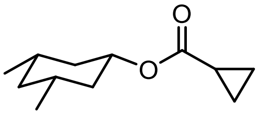

3a

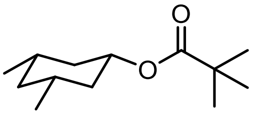

4a

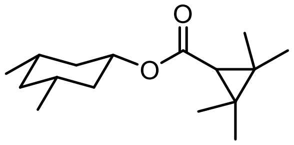

5a

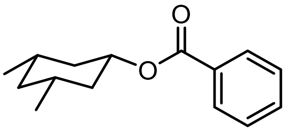

6a

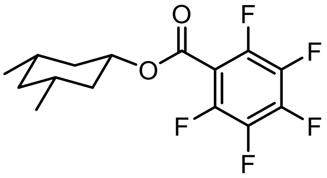

7a

Esters synthesis protocol A:

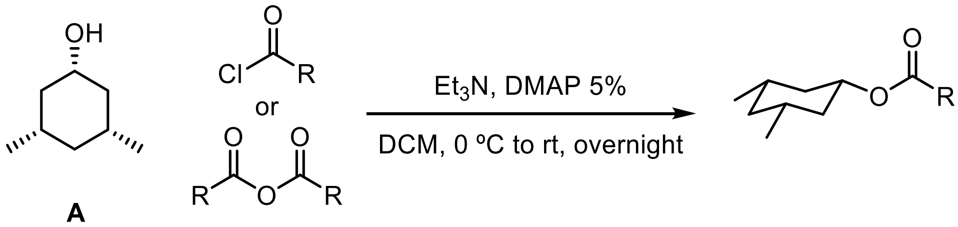

A

A round-bottom flask equipped with a septum and kept under nitrogen was charged with a 0.40 M solution of the alcohol **A** (1 equiv) in dry dichloromethane, triethylamine (1,5 equiv) and DMAP (5 mol %), then the reaction mixture was cooled to 0 °C. The acyl chloride or anhydride (1,5 equiv) was added dropwise over 10 minutes and the reaction was left stirring overnight, letting the temperature rise to room temperature. At this point, HCl 1M solution was added until pH~1 and then diluted with dichloromethane. The organic layer was separated from the acid aqueous layer. The aqueous layer was extracted with dichloromethane (2x) and the organic layers were combined. The organic layer was washed with saturated $NaHCO_3$ and dried over anhydrous sodium sulfate (Na_2SO_4), filtered and evaporated to dryness, then the crude ester was purified by flash chromatography over silica gel.

Esters synthesis protocol B:

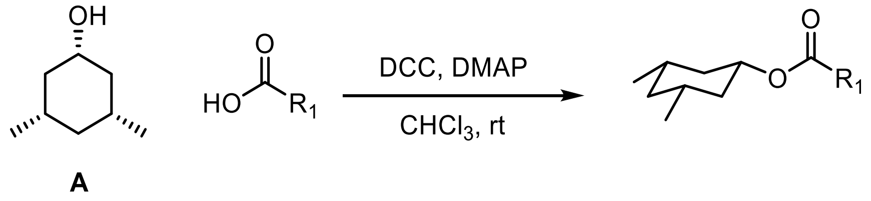

To a round-bottom flask charged with a 0.20 M solution of the alcohol **A** (1 equiv) in chloroform was added DCC (3.5 equiv), DMAP (2.5 equiv) and the corresponding carboxylic acid (4,5 equiv), and the mixture was left stirring until alcohol was consumed (monitored by TLC). Hexane was added to the rection mixture and the precipitate dicyclohexyl urea was removed by filtration throught a plug of silica gel, which was subsequently rinsed with ethyl acetate (2x). The organic phase was successively washed with the following solutions: 1M HCl, $NaHCO_{3\ (aq,\ sat)}$, H_2O. The organic layer was dried over anhydrous sodium sulfate (Na_2SO_4), filtered and evaporated to dryness, then the crude ester was purified by flash chromatography over silica gel.

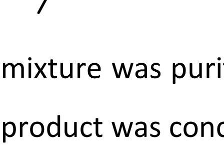

2a: Following the general conditions of *ester synthesis protocol A*, the crude mixture was purified by flash chromatography over silica using hexane:ethyl acetate 50:1 and the product was concentrated to dryness. The product was isolated as a yellow oil (0.21 g, 89% yield).

^{1}H NMR (400 MHz, $CDCl_3$) δ 4.72 (tt, *J* = 11.4, 4.4 Hz, 1H), 2.02 (s, 3H), 1.93 (ddq, *J* = 11.4, 3.5, 1.8 Hz, 2H), 1.61 (ddt, *J* = 12.5, 3.4, 1.7 Hz, 1H), 1.52 (dddd, *J* = 15.3, 8.8, 6.5, 3.3 Hz, 2H), 0.99 – 0.84 (m, 8H), 0.55 (q, *J* = 12.0 Hz, 1H). ^{13}C NMR (101 MHz, $CDCl_3$) δ 170.7, 73.0, 43.0, 39.9, 30.6, 22.1, 21.4. HRMS (ESI+) *m/z* calculated for $C_{10}H_{18}O_2$ $[M+Na]^+$ 193.1199, found 193.1204.

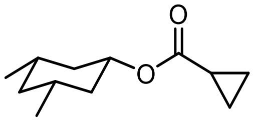

3a: Following the general conditions of *ester synthesis protocol A*, the crude mixture was purified by flash chromatography over silica using hexane: ethyl acetate 50:1 and the product was concentrated to dryness. The product was isolated as a yellow oil (0.61 g, 83% yield). ^{1}H NMR (400 MHz, $CDCl_3$) δ 4.72 (tt, *J* = 11.4, 4.4 Hz, 1H), 1.93 (ddq, *J* = 11.7, 3.9, 1.8 Hz, 2H), 1.61 – 1.44 (m, 4H), 0.98 – 0.95 (m, 2H), 0.95 – 0.88 (m, 8H), 0.82 (dt, *J* = 8.2, 3.4 Hz, 2H), 0.55 (q, *J* = 11.8 Hz, 1H). ^{13}C NMR (101 MHz, $CDCl_3$) δ 174.4, 72.9, 43.0, 40.0, 30.6, 22.1, 13.2, 8.2. HRMS (ESI+) *m/z* calculated for $C_{12}H_{20}O_2$ $[M+Na]^+$ 219.1356, found 219.1360.

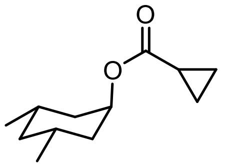

3ax: Following the general conditions of *ester synthesis protocol A* starting from (1β,3α,5α)-3,5-dimethylcyclohexan-1-ol (**B**), the crude mixture was purified by flash chromatography over silica using hexane: ethyl acetate 50:1 and the product was concentrated to dryness. The product was isolated as a colourless oil (0.12 g, 65% yield). ^{1}H NMR (400 MHz, $CDCl_3$) δ 5.07 (p, *J* = 3.0 Hz, 1H), 1.84 – 1.63 (m, 5H), 1.55 – 1.52 (m, 1H), 1.04 – 0.92 (m, 4H), 0.85 (d, *J* = 6.5 Hz, 6H), 0.81 (dt, *J* = 8.1, 3.4 Hz, 2H), 0.56 (q, *J* = 11.9 Hz, 1H). ^{13}C NMR (101 MHz, $CDCl_3$) δ 174.3, 70.6, 43.5, 38.1, 26.9, 22.4, 13.3, 8.2. HRMS (ESI+) *m/z* calculated for $C_{12}H_{20}O_2$ $[M+Na]^+$ 219.1356, found 219.1351.

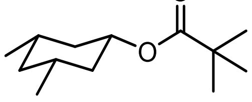

4a: Following the general conditions of *ester synthesis protocol A*, the crude mixture was purified by flash chromatography over silica using hexane:ethyl acetate 50:1 and the product was concentrated to dryness. The product was isolated as a yellow oil (0.71 g, 72% yield).

^{1}H NMR (400 MHz, $CDCl_3$) δ 4.69 (tt, *J* = 11.3, 4.4 Hz, 1H), 1.90 (ddt, *J* = 10.0, 4.0, 2.0 Hz, 2H), 1.61 (ddq, *J* = 14.4, 3.5, 1.7 Hz, 1H), 1.51 (dtd, *J* = 12.2, 6.0, 2.8 Hz, 2H), 1.17 (s, 9H), 1.00 – 0.84 (m, 8H), 0.55 (q, *J* = 12.0 Hz, 1H). ^{13}C NMR (101 MHz, $CDCl_3$) δ 178.1, 72.5, 43.1, 39.8, 38.6, 30.6, 27.1, 22.1. HRMS (ESI+) *m/z* calculated for $C_{13}H_{24}O_2$ $[M+Na]^+$ 235.1669, found 235.1669.

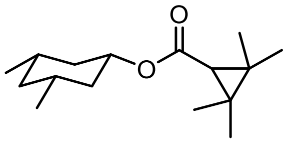

5a: Following the general conditions of *ester synthesis protocol B*, the crude mixture was purified by flash chromatography over silica using hexane:ethyl acetate 80:1 and the product was concentrated to dryness. The product was isolated as a colorless oil (0.30 g, 75% yield). ^{1}H NMR (400 MHz, $CDCl_3$) δ 4.70 (tt, *J* = 11.4, 4.3 Hz, 1H), 1.93 (ddt, *J* = 10.0, 4.1, 2.1 Hz, 2H), 1.60 (ddq, *J* = 12.7, 3.5, 1.8 Hz, 1H), 1.54 – 1.43 (m, 2H), 1.24 (s, 6H), 1.17 (s, 6H), 1.14 (s, 1H), 0.98 – 0.84 (m, 8H), 0.54 (q, *J* = 12.0 Hz, 1H). ^{13}C NMR (101 MHz, $CDCl_3$) δ 171.8, 72.2, 43.1, 40.3, 36.1, 30.7, 29.7, 23.6, 22.2, 16.6. HRMS (ESI+) *m/z* calculated for $C_{16}H_{28}O_2$ $[M+Na]^+$ 275,1987, found 275.1979.

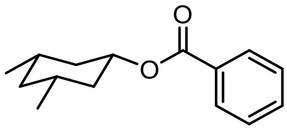

6a: Following the general conditions of *ester synthesis protocol A*, the crude mixture was purified by flash chromatography over silica using hexane:ethyl acetate 50:1 and the product was concentrated to dryness. The product was isolated as a colourless oil (1.24 g, 85% yield). ^{1}H NMR (400 MHz, $CDCl_3$) δ 8.07 – 8.00 (m, 2H), 7.58 – 7.51 (m, 1H), 7.47 – 7.38 (m, 2H), 4.98 (tt, *J* = 11.3, 4.4 Hz, 1H), 2.07 (dtd, *J* = 11.4, 3.8, 1.8 Hz, 2H), 1.70 – 1.57 (m, 3H), 1.07 (q, *J* = 11.9 Hz, 2H), 0.96 (d, *J* = 6.5 Hz, 6H), 0.68 – 0.54 (m, 1H). ^{13}C NMR (101 MHz, $CDCl_3$) δ 166.1, 132.7, 130.9, 129.5, 128.2, 73.6, 43.1, 40.0, 30.6, 22.2. HRMS (ESI+) *m/z* calculated for $C_{15}H_{20}O_2$ $[M+Na]^+$ 255.1356, found 255.1360.

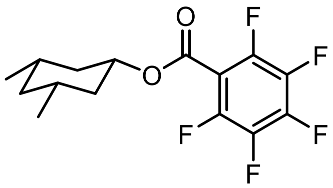

7a: Following the general conditions of *ester synthesis protocol A*, the crude mixture was purified by flash chromatography over silica using hexane: ethyl acetate 200:1

and the product was concentrated to dryness. The product was isolated as a colourless oil (0.12 g, 78% yield). ^{1}H NMR (400 MHz, $CDCl_3$) δ 5.03 (tt, *J* = 11.4, 4.4 Hz, 1H), 2.08 (dtd, *J* = 11.7, 3.7, 1.9 Hz, 2H), 1.70 – 1.56 (m, 3H), 1.06 (q, *J* = 11.9 Hz, 2H), 0.97 (d, *J* = 6.5 Hz, 6H), 0.61 (q, *J* = 12.0 Hz, 1H). ^{13}C NMR (101 MHz, $CDCl_3$) δ 158.5, 146.5- 146.2 (m), 144.3- 144.1 (m), 144.0- 143.7 (m), 141.8- 141.5 (m), 139.1- 138.7, 136.5- 136.2 (m), 109.2- 108.8 (m), 76.2, 42.8, 39.6, 30.6, 22.0. ^{19}F NMR (377 MHz, $CDCl_3$) δ -139.80 – -139.95 (m), -150.47 (tt, *J* = 21.0, 4.1 Hz), -161.58 (tt, *J* = 20.7, 5.9 Hz). HRMS (ESI+) *m/z* calculated for $C_{15}H_{15}F_5O_2$ $[M+Na]^+$ 345.0884, found 345.0888.

Synthesis of 8a

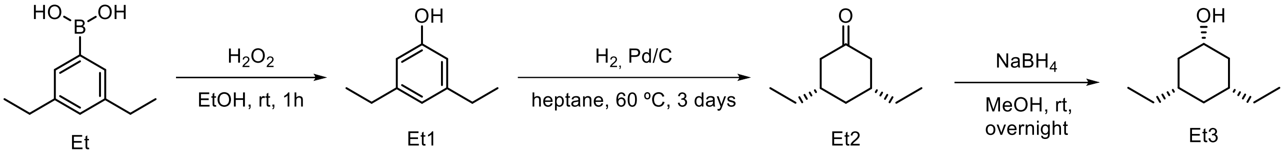

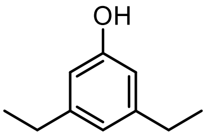

Synthesis of intermediate Et1

Et1 was synthesized using a modified reported methodology.[3]

A round-bottom flask was charged with the phenylboronic acid **Et**[4] (870 mg, 4.9 mmol, 1 equiv) in 5 ml of EtOH. Then, hydrogen peroxide (30% H_2O_2 solution in water, 1.5 ml, 14.7 mmol, 3 equiv) was added at room temperature and stirred for 1 minute. At this point, the reaction mixture was quenched with 10 mL of H_2O. The aqueous layer was extracted with ethyl acetate (3x) and the organic fractions were combined, dried over anhydrous sodium sulfate (Na_2SO_4), filtered and evaporated to dryness. Then the crude was purified by flash chromatography over silica using pentane: ethyl acetate 8:1 to obtain the phenol **Et1** (422mg, 58 % yield) as a yellow solid.^{1}H NMR (400 MHz, $CDCl_3$) δ 6.62 (t, *J* = 1.4 Hz, 1H), 6.50 (d, *J* = 1.5 Hz, 2H), 4.60 (s, 1H), 2.58 (q, *J* = 7.6 Hz, 4H), 1.22 (t, *J* = 7.7 Hz, 6H). NMR Spectral data match those previously reported.[5]

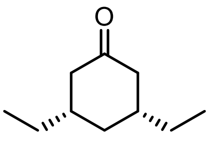

Synthesis of intermediate Et2

Et2 was synthesized using a modified reported methodology.[6]

A vial was charged with the phenol **Et1** (422 mg, 2.8 mmol, 1 equiv) and Pd/C (30% w/w), sealed with a septum and kept under nitrogen. Then, 4 ml of degassed heptane with freeze-pump technique was added under nitrogen flow. At this point, the vial was placed in a Parr reactor and the system was purged with hydrogen flow under stirring. After 5 minutes, the hydrogen pressure was set at 10 bar and left stirring at 60 ºC for 3 days. After carefully depressurization, the reaction mixture was filtered over acrodisc® and purified by flash chromatography over silica using pentane: ethyl acetate 24:1 to obtain the ketone **Et2** (200mg, 46 % yield) as a yellow oil (Due to the volatile nature of this compound, ethyl acetate was not completely removed). ^1H NMR (400 MHz, $CDCl_3$) δ 2.41 – 2.34 (m, 2H), 1.96 – 1.86 (m, 3H), 1.60 (dddt, *J* = 12.7, 9.5, 6.3, 3.2 Hz, 2H), 1.45 – 1.27 (m, 4H), 0.96 (dt, *J* = 13.2, 11.9 Hz, 1H), 0.89 (t, *J* = 7.5 Hz, 6H). ^{13}C NMR (101 MHz, $CDCl_3$) δ 212.1, 47.6, 39.7, 37.8, 29.8, 11.1. ESI-MS *m/z* found for $C_{10}H_{18}O$ $[M+H]^+$ 155.1

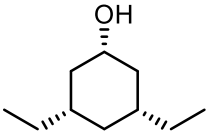

Synthesis of intermediate Et3

A round-bottom flask equipped with a septum and kept under nitrogen, was cooled to 0º C, then was charged with the ketone **Et2** (126mg, 0.8 mmol) and 5 ml of dry MeOH. At this point, $NaBH_4$ (61 mg, 1.6 mmol, 2 equiv) was added slowly as a solid under nitrogen flow. The reaction was left stirring overnight, letting to warm up to room temperature. After this, the reaction mixture was quenched with 2 ml of water and diluted with 10 ml of ether and water. The organic layer was separated from the aqueous layer. The aqueous layer was extracted with ether (2x) and the organic layers were combined. The organic layer was washed with brine and dried over anhydrous sodium sulfate (Na_2SO_4), filtered and evaporated to dryness, then the crude was purified by flash chromatography over silica using pentane: diethyl ether 1: 0 → 10: 1 →8: 1 to obtain **Et3** (56 mg, 44% yield) as a yellow oil. ^1H NMR (400 MHz, $CDCl_3$) δ 3.58 (ddd, *J* = 15.3, 10.9, 4.3 Hz, 1H), 2.00 (dd, *J* = 13.3, 9.6 Hz, 2H), 1.67 (d, *J* = 12.8 Hz, 1H), 1.39 – 1.15 (m, 7H), 0.93 – 0.68 (m, 8H), 0.45 (q, *J* = 11.6 Hz, 1H). ^{13}C NMR (101 MHz, $CDCl_3$) δ 70.8, 42.1, 38.3, 37.4, 29.6, 11.4. ESI-MS *m/z* found for $C_{10}H_{20}O$ $[M+Na]^+$ 179.2

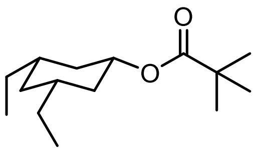

8a: Following the general conditions of *ester synthesis protocol A* starting from the alcohol **Et3**, the crude mixture was purified by flash chromatography over silica using pentane to obtain **8a** as a colourless oil (69 mg, 84% yield). ^{1}H NMR (400 MHz, $CDCl_3$) δ 4.67 (tt, *J* = 11.3, 4.4 Hz, 1H), 1.94 (d, *J* = 12.1 Hz, 2H), 1.70 (d, *J* = 13.0 Hz, 1H), 1.31 – 1.22 (m, 6H), 1.16 (s, 9H), 0.91 – 0.83 (m, 8H), 0.48 (q, *J* = 11.6 Hz, 1H). ^{13}C NMR (101 MHz, $CDCl_3$) δ 178.1, 72.8, 38.6, 38.2, 37.9, 37.1, 29.6, 27.2, 11.4. HRMS (ESI+) *m/z* calculated for $C_{15}H_{28}O_2$ $[M+Na]^+$ 263.1982, found 263.1982.

Synthesis of 9a

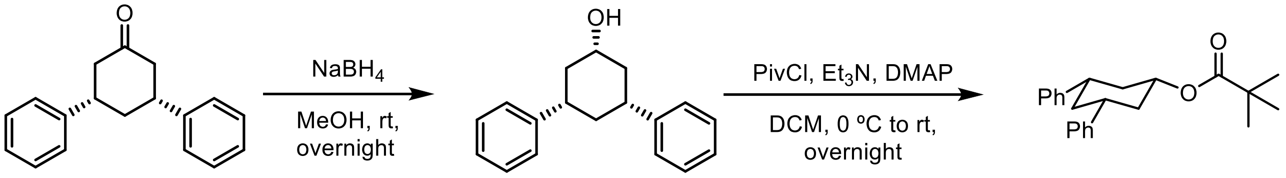

A round-bottom flask equipped with a septum and kept under nitrogen, was cooled to 0º C, then was charged with the ketone **Ph**[7] (1 g, 4.03 mmol, 1 equiv) and 100 ml of dry MeOH. At this point, $NaBH_4$ (228 mg, 6 mmol, 1.5 equiv) was added slowly as a solid under nitrogen flow. The reaction was left stirring overnight, letting to warm up to room temperature. After this, the reaction mixture was evaporated to remove almost all methanol and diluted with ethyl acetate and water. The organic layer was separated from the aqueous layer. The aqueous layer was extracted with acetate (2x) and the organic layers were combined. The organic layer was washed with brine and dried over anhydrous sodium sulfate (Na_2SO_4), filtered and evaporated to dryness. At this point, the crude solid was solubilized in 20 ml of hot MeOH/H_2O mixture (1:1) and left cool down to precipitate **Ph1**. After removing the solvent, **Ph1** intermediate was immediately used for the next step without further purification.

A round-bottom flask equipped with a septum and kept under nitrogen, was charged with the alcohol **Ph1** obtained in 50 ml of dry DCM, triethylamine (830 µl, 6 mmol, 1,5 equiv) and DMAP (25 mg, 5 mol %), then the reaction mixture was cooled to 0 °C. At this point, the pivaloyl chloride (730 µl, 6 mmol, 1,5 equiv) was added dropwise over 10 minutes and the reaction was left stirring overnight, letting the temperature rise to room temperature. At this point, HCl 1M solution was

added until pH~1 and then diluted with dichloromethane. The organic layer was separated from the acid aqueous layer. The aqueous layer was extracted with dichloromethane (2x) and the organic layers were combined. The organic layer was washed with saturated $NaHCO_3$ and dried over anhydrous sodium sulfate (Na_2SO_4), filtered and evaporated to dryness, then the crude was purified by flash chromatography over silica using hexane: ethyl acetate 10: 1 to obtain **9a** (470 mg, 35% yield, over 2 steps) as a white solid. ^{1}H NMR (400 MHz, CD_2Cl_2) δ 7.34 – 7.24 (m, 8H), 7.23 – 7.16 (m, 2H), 5.00 (tt, *J* = 11.2, 4.4 Hz, 1H), 2.88 (tt, *J* = 12.5, 3.3 Hz, 2H), 2.26 – 2.16 (m, 2H), 2.04 (dtt, *J* = 12.9, 3.4, 1.8 Hz, 1H), 1.63 (p, *J* = 12.5 Hz, 3H), 1.17 (s, 9H). ^{13}C NMR (101 MHz, CD_2Cl_2) δ 177.7, 145.7, 128.5, 126.9, 126.3, 72.4, 42.2, 41.0, 38.8, 38.5, 26.9. HRMS (ESI+) *m/z* calculated for $C_{23}H_{28}O_2$ $[M+Na]^+$ 359.1982, found 359.1982.

Synthesis of 10a

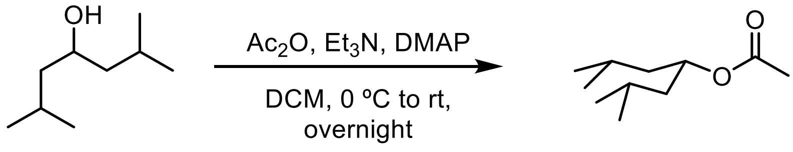

10 **10a**

A round-bottom flask equipped with a septum and kept under nitrogen, was charged with the alcohol **10** (580 mg, 4 mmol, 1 equiv) in 50 ml of dry DCM, triethylamine (830 µl, 6 mmol, 1,5 equiv) and DMAP (25 mg, 5 mol %), then the reaction mixture was cooled to 0 °C. At this point, the acetic anhydride (573 µl, 6 mmol, 1,5 equiv) was added dropwise over 10 minutes and the reaction was left stirring overnight, letting the temperature rise to room temperature. At this point, HCl 1M solution was added until pH~1 and then diluted with dichloromethane. The organic layer was separated from the acid aqueous layer. The aqueous layer was extracted with dichloromethane (2x) and the organic layers were combined. The organic layer was washed with saturated $NaHCO_3$ and dried over anhydrous sodium sulfate (Na_2SO_4), filtered and evaporated to dryness, then the crude was purified by flash chromatography over silica using hexane: ethyl acetate 50: 1 to obtain **10a** (648 mg, 58% yield) as a yellow liquid. ^{1}H NMR (400 MHz, $CDCl_3$) δ 5.06 (tt, *J* = 8.7, 4.6 Hz, 1H), 2.03 (s, 3H), 1.65 – 1.55 (m, 2H), 1.49 (ddd, *J* = 14.0, 8.5, 5.5 Hz, 2H), 1.27 (ddd, *J* = 13.9, 8.4, 4.6 Hz, 2H), 0.90 (d, *J* = 6.6 Hz, 12H). ^{13}C NMR (101 MHz, $CDCl_3$) δ 170.8, 71.2, 44.0, 24.7, 23.1, 22.3, 21.3. HRMS (ESI+) *m/z* calculated for $C_{11}H_{22}O_2$ $[M+Na]^+$ 209.1512, found 209.1516.

Synthesis of amide substrates

The following amide substrates **11a**, **12a**, **13a**, **14a** were obtained using *amides synthesis protocol* from the azide **B2:**

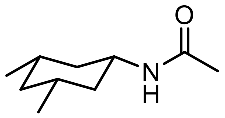

11a

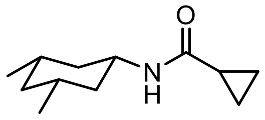

12a

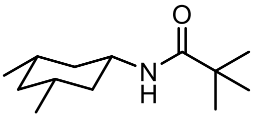

13a

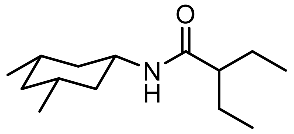

14a

Synthesis of B2

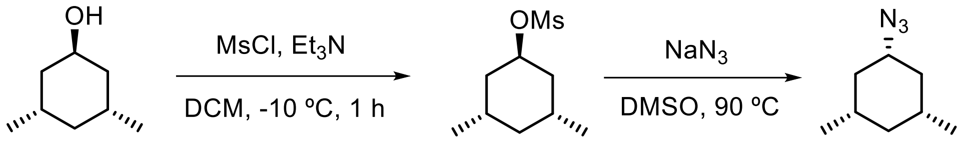

B **B1** **B2**

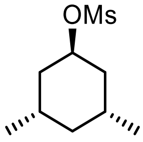

B1:

A round-bottom flask equipped with a septum and kept under nitrogen was charged with alcohol **B** (780 mg, 6.1 mmol, 1 equiv), triethylamine (1.2 ml, 8.5 mmol, 1.4 equiv) and 35 ml of dry dichloromethane, then the reaction mixture was cooled to -10 °C. At this point, methanesulfonyl chloride (566 μl, 7.3 mmol, 1.2 equiv) was delivered over a period of 40 minutes by syringe pump into the solution, and the reaction was left stirring at the same temperature for other 20 minutes. The reaction mixture was successively washed with the following solutions: H_2O, 1M HCl, $NaHCO_3$ $_{(aq,\ sat)}$, NaCl $_{(aq,\ sat)}$. The organic layer was dried over anhydrous sodium sulfate (Na_2SO_4), filtered and evaporated to dryness to obtain **B1** (1.093 g, 87% yield) as a yellow oil without further purification. ^{1}H NMR (400 MHz, $CDCl_3$) δ 5.06 (p, *J* = 2.9 Hz, 1H), 3.00 (s, 3H), 2.09 – 1.98 (m, 2H), 1.84 (tdp, *J* = 12.9, 6.7, 3.2 Hz, 2H), 1.72 (dtt, *J* = 13.1, 3.6, 2.0 Hz, 1H), 1.12 (ddd, *J* = 14.7, 12.6, 2.5 Hz, 2H), 0.90 (d, *J* = 6.6 Hz, 6H), 0.61 (q, *J* = 12.2 Hz, 1H). ^{13}C NMR (101 MHz, $CDCl_3$) δ 80.2, 43.0, 39.0, 38.6, 26.5, 22.1. HRMS (ESI+) *m/z* calculated for $C_9H_{18}O_3S$ $[M+Na]^+$ 229.0869, found 229.0877.

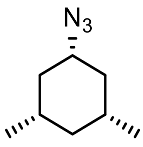 **B2**:

A two-neck round-bottom flask equipped with a septum and kept under nitrogen was charged with **B1** (413 mg, 2.0 mmol, 1 equiv) (attention, work with small quantities, azides are potentially explosive!!) and 15 ml of DMSO. At this point, sodium azide (650 mg, 10 mmol, 5 equiv) was added as a solid, and the reaction was heated slowly to 90 ºC and left stirring overnight. At this point, the reaction mixture was then cooled down to room temperature and diluite with ethyl acetate. The reaction mixture was washed with H_2O (3x) and NaCl $_{(aq,\ sat)}$. The organic layer was dried over anhydrous sodium sulfate (Na_2SO_4), filtered and evaporated to dryness to obtain **B2** (200 mg, 65% yield) as a yellow oil without further purification. ^{1}H NMR (400 MHz, $CDCl_3$) δ 3.27 (tt, *J* = 11.8, 4.1 Hz, 1H), 1.94 (ddq, *J* = 11.5, 3.6, 1.8 Hz, 2H), 1.63 (dtt, *J* = 12.9, 3.5, 1.8 Hz, 1H), 1.54 – 1.41 (m, 2H), 0.94 (d, *J* = 6.5 Hz, 6H), 0.93 – 0.84 (m, 2H), 0.55 (dt, *J* = 13.0, 11.8 Hz, 1H). ^{13}C NMR (101 MHz, $CDCl_3$) δ 59.8, 42.9, 39.8, 31.1, 22.1.

Amides synthesis protocol

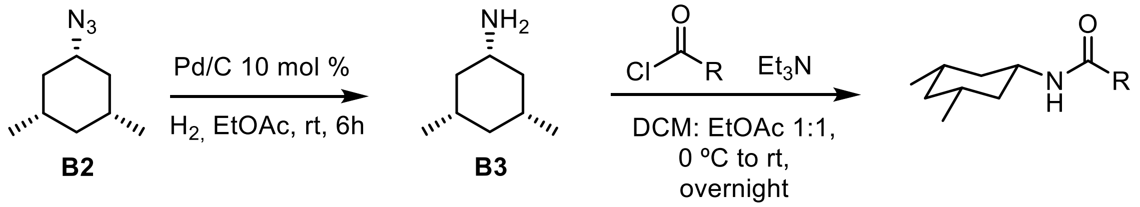

A round-bottom flask equipped with a septum and kept under nitrogen, was charged with a 0.30 M solution of the azide **B2** (1 equiv) in ethyl acetate. Palladium on carbon (10 %) was added as a solid under nitrogen flow. At this point the flask was sealed, evacuated, and backfilled with hydrogen gas (3 times), and kept under hydrogen (balloon) and the reaction was left stirring for 6h. After that, the reaction mixture was filtered over celite in a round-bottom flask and washed with dry dichloromethane. The crude amine **B3** in solution of CH_2Cl_2: EtOAc (1:1) was immediately acylated without further purification.

To a freshly prepared solution **B3** in CH_2Cl_2: EtOAc (1:1) was added triethylamine (1.0 equiv, respectively to the starting azide **B2**), then the reaction mixture was cooled to 0 °C. The acyl chloride (1.0 equiv) was added dropwise over 10 minutes and the reaction was left stirring overnight, letting to warm up to room temperature. At this point, a saturated aqueous Na_2CO_3 solution was added

until pH~10-11 and then diluted with dichloromethane. The organic layer was separated from the basic aqueous layer. The aqueous layer was extracted with dichloromethane (2x) and the organic layers were combined. The organic layer was washed with 1N HCl and dried over anhydrous sodium sulfate (Na_2SO_4). The organic layer was evaporated to dryness and the crude amide was purified by flash chromatography over silica gel.

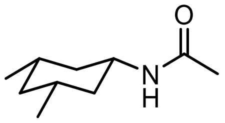

11a: Following the general conditions of a*mides synthesis protocol*, the crude mixture was purified by flash chromatography over silica using hexane: ethyl acetate 1:1 and the product was concentrated to dryness. The product was isolated as a white solid (0.27 g, 81% yield). ^{1}H NMR (400 MHz, $CDCl_3$) δ 5.28 (s, 1H), 3.79 (tdt, *J* = 12.0, 8.1, 4.0 Hz, 1H), 1.95 (s, 3H), 1.94 – 1.89 (m, 2H), 1.63 (dtt, *J* = 12.7, 3.7, 1.9 Hz, 1H), 1.53 (dddd, *J* = 15.2, 8.7, 6.6, 3.4 Hz, 2H), 0.90 (d, *J* = 6.5 Hz, 6H), 0.65 (q, *J* = 11.9 Hz, 2H), 0.51 (q, *J* = 11.8 Hz, 1H). ^{13}C NMR (101 MHz, $CDCl_3$) δ 169.1, 48.3, 43.2, 41.5, 31.2, 23.6, 22.2. HRMS (ESI+) *m/z* calculated for $C_{10}H_{19}NO$ $[M+Na]^+$ 192.1359, found 192.1360.

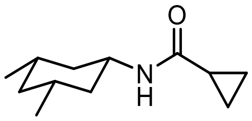

12a: Following the general conditions of a*mides synthesis protocol*, the crude mixture was purified by flash chromatography over silica using hexane: ethyl acetate 1:1 and the product was concentrated to dryness. The product was isolated as a white solid (0.23 g, 78% yield). ^{1}H NMR (400 MHz, $CDCl_3$) δ 5.39 (s, 1H), 3.81 (tdt, *J* = 12.0, 8.1, 4.0 Hz, 1H), 1.94 (ddt, *J* = 12.0, 3.9, 2.0 Hz, 2H), 1.62 (ddq, *J* = 12.7, 3.6, 1.8 Hz, 1H), 1.51 (dtq, *J* = 16.3, 6.6, 3.3 Hz, 2H), 1.29 – 1.23 (m, 2H), 0.98 – 0.92 (m, 2H), 0.90 (d, *J* = 6.6 Hz, 6H), 0.73 – 0.65 (m, 3H), 0.51 (q, *J* = 12.0 Hz, 1H). ^{13}C NMR (101 MHz, $CDCl_3$) δ 172.5, 48.4, 43.2, 41.6, 31.2, 22.3, 14.9, 7.0. HRMS (ESI+) *m/z* calculated for $C_{12}H_{21}NO$ $[M+Na]^+$ 218.1515, found 218.1516.

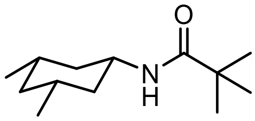

13a: Following the general conditions of a*mides synthesis protocol*, the crude mixture was purified by flash chromatography over silica using hexane: ethyl acetate 1:1 and the product was concentrated to dryness. The product was isolated as a white solid (0.25 g, 82% yield). ^{1}H NMR (400 MHz, $CDCl_3$) δ 5.37 (s, 1H), 3.77 (tdt, *J* = 11.9, 8.0, 4.0 Hz, 1H), 1.91 (ddt, *J* = 12.1, 4.0, 2.1 Hz, 2H), 1.65 (dq, *J* = 3.6, 1.8 Hz, 1H), 1.62 – 1.47 (m, 2H), 1.17 (s, 9H), 0.90 (d, *J* = 6.5 Hz, 6H),

0.65 (q, J = 11.9 Hz, 2H), 0.51 (q, J = 12.0 Hz, 1H). ^{13}C NMR (101 MHz, $CDCl_3$) δ 177.5, 48.0, 43.3, 41.5, 38.5, 31.2, 27.6, 22.3. HRMS (ESI+) *m/z* calculated for $C_{13}H_{25}NO$ $[M+Na]^+$ 234.1828, found 234.1830.

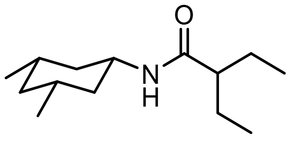

14a: Following the general conditions of a*mides synthesis protocol*, the crude mixture was purified by flash chromatography over silica using hexane: ethyl acetate 1:1 and the product was concentrated to dryness. The product was isolated as a white solid (0.22 g, 79% yield). ^1H NMR (400 MHz, $CDCl_3$) δ 5.21 (d, J = 8.2 Hz, 1H), 3.86 (tdt, J = 12.1, 8.2, 4.0 Hz, 1H), 1.93 (ddt, J = 12.0, 4.0, 2.1 Hz, 2H), 1.76 (tt, J = 9.4, 5.0 Hz, 1H), 1.67 – 1.58 (m, 3H), 1.58 – 1.49 (m, 3H), 1.44 (dtt, J = 12.3, 7.3, 3.7 Hz, 2H), 0.92 – 0.85 (m, 11H), 0.67 (q, J = 11.9 Hz, 2H), 0.51 (q, J = 12.0 Hz, 1H). ^{13}C NMR (101 MHz, $CDCl_3$) δ 174.6, 51.8, 48.0, 43.3, 41.7, 31.2, 25.9, 22.3, 12.1. HRMS (ESI+) *m/z* calculated for $C_{14}H_{27}NO$ $[M+Na]^+$ 248.1985, found 248.1987.

Synthesis of 15a

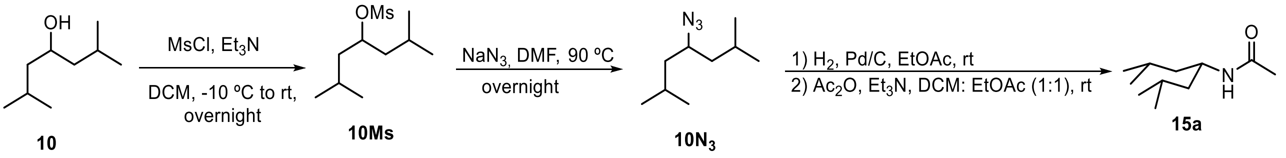

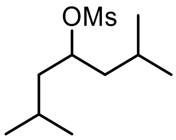

10Ms

A round-bottom flask equipped with a septum and kept under nitrogen was charged with alcohol **10** (1 g, 7.4 mmol, 1 equiv), triethylamine (2.6 ml, 18.5 mmol, 2.5 equiv) and 100 ml of dry dichloromethane, then the reaction mixture was cooled to -10 °C. At this point, methanesulfonyl chloride (1.4 ml, 18.5 mmol, 2.5 equiv) was delivered over a period of 40 minutes by syringe pump into the solution, and the reaction was left stirring overnight at room temperature. The reaction mixture was successively washed with the following solutions: H_2O, 1M HCl, $NaHCO_3$ $_{(aq,\ sat)}$, NaCl $_{(aq,\ sat)}$. The organic layer was dried over anhydrous sodium sulfate (Na_2SO_4), filtered and evaporated to dryness to obtain **10Ms** (0.93 g, 57% yield) as a yellow oil without further purification. ^1H NMR (400 MHz, $CDCl_3$) δ 4.86 (tt, J = 7.4, 5.3 Hz, 1H), 2.99 (s, 3H), 1.83 – 1.60 (m, 4H), 1.45 (ddd, J = 13.8, 7.9, 5.3 Hz, 2H), 0.95 (dd, J = 9.7, 6.5 Hz, 12H). ^{13}C NMR (101 MHz, $CDCl_3$) δ 81.6, 44.3, 39.1, 24.6, 23.0, 22.4. ESI-MS *m/z* found for $C_{10}H_{22}O_3S$ $[M+Na]^+$ 245.1

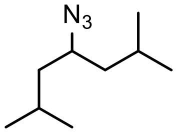

10N₃

A two-neck round-bottom flask equipped with a septum and kept under nitrogen was charged with **10Ms** (444 mg, 2.0 mmol, 1 equiv.) (attention, work with small quantities, azides are potentially explosive!!) and 15 ml of DMSO. At this point, sodium azide (650 mg, 10 mmol, 5 equiv) was added as a solid, and the reaction was heated slowly to 90 ºC and left stirring overnight. At this point, the reaction mixture was then cooled down to room temperature and diluite with ethyl acetate. The reaction mixture was washed with H_2O (3x) and NaCl $_{(aq,\ sat)}$. The organic layer was dried over anhydrous sodium sulfate (Na_2SO_4), filtered and evaporated to dryness to obtain the azide **$10N_3$** (235 mg, 70% yield) as a yellow oil without further purification. ^{1}H NMR (400 MHz, $CDCl_3$) δ 3.58 (tt, *J* = 9.3, 4.7 Hz, 1H), 2.04 (dddd, *J* = 15.2, 12.0, 7.7, 6.1 Hz, 2H), 1.73 (ddd, *J* = 14.5, 9.2, 5.4 Hz, 2H), 1.52 (ddd, *J* = 13.8, 8.6, 4.7 Hz, 2H), 1.19 (d, *J* = 6.6 Hz, 12H). ^{13}C NMR (101 MHz, $CDCl_3$) δ 59.1, 44.0, 25.2, 23.2, 22.1.

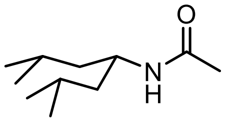

15a

A round-bottom flask equipped with a septum and kept under nitrogen, was charged with a 0.30 M solution of the azide **$10N_3$** (400mg, 2.4 mmol, 1 equiv.) in ethyl acetate. Palladium on carbon (10 %) was added as a solid under nitrogen flow. At this point the flask was sealed, evacuated, and backfilled with hydrogen gas (3 times), and kept under hydrogen (balloon) and the reaction was left stirring for 6h. After that, the reaction mixture was filtered over celite in a round-bottom flask and washed with dry dichloromethane. The crude amine in solution of CH_2Cl_2: EtOAc (1:1) was immediately acylated without further purification.

To a freshly prepared solution crude amine in CH_2Cl_2: EtOAc (1:1) was added triethylamine (1.5 equiv, respectively to the starting azide), then the reaction mixture was cooled to 0 °C. The acyl chloride (1.5 equiv) was added dropwise over 10 minutes and the reaction was left stirring overnight, letting to warm up to room temperature. At this point, a saturated aqueous Na_2CO_3 solution was added until pH~10-11 and then diluted with dichloromethane. The organic layer was separated from the basic aqueous layer. The aqueous layer was extracted with dichloromethane (2x) and the organic layers were combined. The organic layer was dried over anhydrous sodium sulfate (Na_2SO_4), filtered and evaporated to dryness, then the crude was purified by flash

chromatography over silica using hexane:ethyl acetate 1:1 to obtain **15a** (220mg, 49 % yield) as a white solid. ^1H NMR (400 MHz, $CDCl_3$) δ 5.02 (d, *J* = 9.5 Hz, 1H), 4.11 (dp, *J* = 9.5, 7.1 Hz, 1H), 1.98 (s, 3H), 1.61 (dd, *J* = 13.5, 6.7 Hz, 2H), 1.26 (t, *J* = 7.0 Hz, 4H), 0.93 (dd, *J* = 8.5, 6.6 Hz, 12H). ^{13}C NMR (101 MHz, $CDCl_3$) δ 169.4, 45.9, 45.8, 25.1, 23.7, 23.3, 22.5. HRMS (ESI+) *m/z* calculated for $C_{11}H_{23}NO$ $[M+Na]^+$ 208.1672, found 208.1671.

1.3.2 Synthesis of substrates 17a – 28a

Synthesis of 17a

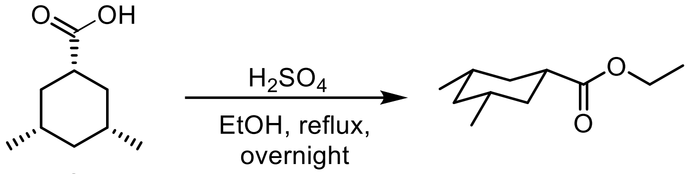

C **17a**

A round-bottom flask was charged with **C**[8] (500 mg, 3.2 mmol, 1 equiv), 10 ml of ethanol and one drop of sulfuric acid, then the reaction mixture was heated to reflux and left stirring overnight. At this point, the reaction mixture was cooled down to room temperature, evaporated almost the all solvent and diluite with ethyl acetate. The reaction mixture was washed with H_2O (3x) and NaCl $_{(aq, sat)}$. The organic layer was dried over anhydrous sodium sulfate (Na_2SO_4), filtered and evaporated to dryness, then the crude was purified by flash chromatography over silica using hexane:ethyl acetate 100 :1 to obtain **17a** (410mg, 69 % yield) as a yellow oil. ^1H NMR (400 MHz, $CDCl_3$) δ 4.11 (q, *J* = 7.1 Hz, 2H), 2.33 (tt, *J* = 12.3, 3.5 Hz, 1H), 1.89 (dtt, *J* = 12.5, 3.4, 1.5 Hz, 2H), 1.64 (dtt, *J* = 13.0, 3.7, 1.9 Hz, 1H), 1.44 (tdq, *J* = 13.0, 6.7, 3.3 Hz, 2H), 1.25 (t, *J* = 7.1 Hz, 3H), 1.05 – 0.93 (m, 2H), 0.92 (d, *J* = 6.6 Hz, 6H), 0.57 (dt, *J* = 13.0, 11.8 Hz, 1H). ^{13}C NMR (101 MHz, $CDCl_3$) δ 176.1, 60.1, 43.4, 43.4, 37.0, 31.9, 22.4, 14.3. HRMS (ESI+) *m/z* calculated for $C_{11}H_{20}O_2$ $[M+Na]^+$ 207.1356, found 207.1364.

Synthesis of intermediates C1 and C2

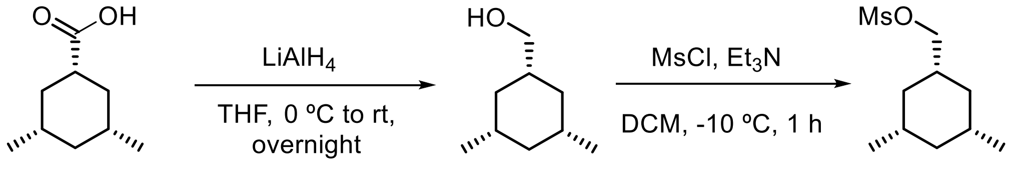

C **C1** **C2**

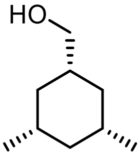

Synthesis of intermediate C1

A round-bottom flask equipped with a septum and kept under nitrogen, was cooled to 0º C, then was charged with lithium aluminium hydride (1.82 g, 48 mmol, 3 equiv) and 200 ml of dry THF. At this point, the carboxylic acid **C** (2.5 g, 16 mmol, 1 equiv) was added slowly as a solid under nitrogen flow. The reaction was left stirring overnight, letting to warm up to room temperature. After this, the reaction mixture was quenched with 1M NaOH solution at 5°C. After filtration through Celite© and extraction with ethyl acetate (2 x 50 mL) the organic fractions were dried over anhydrous Na_2SO_4, filtered and evaporated to dryness to obtain **C1** (2.3 g, 92% yield) as a yellow oil without further purification. ^{1}H NMR (400 MHz, $CDCl_3$) δ 3.43 (d, *J* = 6.3 Hz, 2H), 1.69 (dtt, *J* = 12.3, 3.6, 1.6 Hz, 2H), 1.63 (ddt, *J* = 12.9, 3.6, 1.7 Hz, 1H), 1.53 (ddq, *J* = 12.1, 6.3, 3.2 Hz, 1H), 1.42 (dddd, *J* = 15.1, 8.5, 6.7, 3.4 Hz, 2H), 0.88 (d, *J* = 6.6 Hz, 6H), 0.50 (td, *J* = 12.5, 11.2 Hz, 3H). ^{13}C NMR (101 MHz, $CDCl_3$) δ 68.9, 44.4, 40.6, 38.0, 32.2, 22.9. ESI-MS *m/z* found for $C_9H_{18}O$ $[M+Na]^+$ 165.1

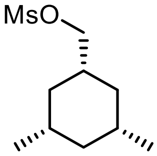

Synthesis of intermediate C2

A round-bottom flask equipped with a septum and kept under nitrogen was charged with alcohol **C1** (870 mg, 6.1 mmol, 1 equiv), triethylamine (1.2 ml, 8.5 mmol, 1.4 equiv) and 35 ml of dry dichloromethane, then the reaction mixture was cooled to -10 °C. At this point, methanesulfonyl chloride (566 μl, 7.3 mmol, 1.2 equiv) was delivered over a period of 40 minutes by syringe pump into the solution, and the reaction was left stirring at the same temperature for other 20 minutes. The reaction mixture was successively washed with the following solutions: H_2O, 1M HCl, $NaHCO_{3\,(aq,\,sat)}$, $NaCl_{(aq,\,sat)}$. The organic layer was dried over anhydrous sodium sulfate (Na_2SO_4), filtered and evaporated to dryness to obtain **C2** (1.093 g, 82% yield) as a yellow oil without further purification. ^{1}H NMR (400 MHz, $CDCl_3$) δ 4.02 (d, *J* = 6.3 Hz, 2H), 3.00 (s, 3H), 1.91 – 1.77 (m, 1H), 1.74 (ddt, *J* = 12.7, 3.9, 2.0 Hz, 2H), 1.67 (dtt, *J* = 13.0, 3.6, 1.8 Hz, 1H), 1.45 (tdq, *J* = 13.0, 6.6, 3.3 Hz, 2H), 0.91 (d, *J* = 6.6 Hz, 6H), 0.68 – 0.48 (m, 3H). ^{13}C NMR (101 MHz, $CDCl_3$) δ 75.0, 43.9, 37.5, 37.4, 37.3, 31.9, 22.7. HRMS (ESI+) *m/z* calculated for $C_{10}H_{20}O_3S$ $[M+Na]^+$ 243.1025, found 243.1042.

Synthesis of 22a

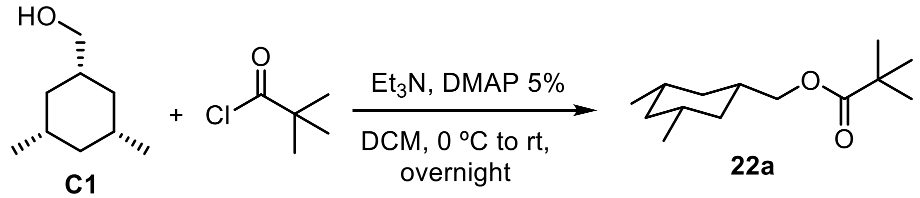

Following the general conditions of *ester synthesis protocol A*, the crude mixture was purified by flash chromatography over silica using hexane:ethyl acetate 50:1 and the product was concentrated to dryness. The product **22a** was isolated as a colourless oil (210 mg, 78% yield^{1}H NMR (400 MHz, $CDCl_3$) δ 3.86 (d, *J* = 6.1 Hz, 2H), 1.79 – 1.62 (m, 4H), 1.43 (dddd, *J* = 15.1, 8.5, 6.6, 3.4 Hz, 2H), 1.21 (s, 9H), 0.90 (d, *J* = 6.6 Hz, 6H), 0.62 – 0.47 (m, 3H). ^{13}C NMR (101 MHz, $CDCl_3$) δ 178.6, 69.4, 44.0, 37.9, 37.2, 31.9, 27.2, 22.7. HRMS (ESI+) *m/z* calculated for $C_{14}H_{26}O_2$ $[M+Na]^+$ 249.1825, found 249.1828.

Synthesis of 23a

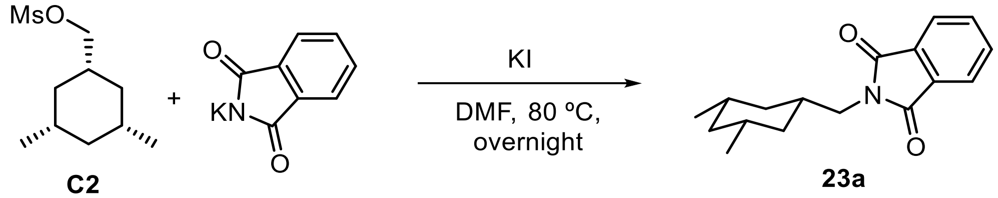

A round-bottom flask was charged with **C2** (188 mg, 0.90 mmol, 1 equiv), KI (14 mg, 0.09 mmol, 0.1 equiv, potassium phthalimide (250 mg, 1.35 mmol, 1.5 equiv) and 6 ml of N,N-Dimethylformamide, then the reaction mixture was heated slowly to 80 ºC and left stirring overnight. At this point, the reaction mixture was cooled down to 0 ºC, quenched with 10 ml of water and washed with EtOAc (3x). The combined organic layers were washed with H_2O and NaCl $_{(aq, sat)}$ and dried over anhydrous sodium sulfate (Na_2SO_4), filtered and evaporated to dryness, then the crude amide was purified by flash chromatography over silica using hexane:ethyl acetate 10:1 to obtain **23a** (166 mg, 70% yield) as a white solid. ^{1}H NMR (400 MHz, $CDCl_3$) δ 7.85 (dd, *J* = 5.4, 3.1 Hz, 2H), 7.71 (dd, *J* = 5.5, 3.0 Hz, 2H), 3.52 (d, *J* = 7.3 Hz, 2H), 1.87 (dddd, *J* = 15.7, 8.5, 6.2, 3.7 Hz, 1H), 1.64 – 1.58 (m, 2H), 1.48 – 1.28 (m, 3H), 0.87 (d, *J* = 6.6 Hz, 6H), 0.68 – 0.46 (m, 3H). ^{13}C NMR (101 MHz, $CDCl_3$) δ 168.7, 133.9, 132.1, 123.2, 44.1, 43.9, 39.0, 37.0, 32.0, 22.6. HRMS (ESI+) *m/z* calculated for $C_{17}H_{21}NO_2$ $[M+Na]^+$ 294.1465, found 294.1472.

Synthesis of 21a

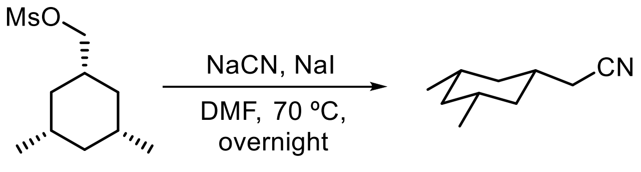

A round-bottom flask was charged with **C2** (600 mg, 2.7 mmol, 1 equiv), NaI (101 mg, 0.7 mmol, 0.25 equiv), NaCN (529 mg, 10.8 mmol, 4 equiv) and 8 ml of N,N-Dimethylformamide, then the reaction mixture was heated slowly to 70 ºC and left stirring overnight. At this point, the reaction mixture was then cooled down to room temperature and diluite with ethyl acetate. The reaction mixture was washed with H_2O (3x) and NaCl $_{(aq,\ sat)}$. The organic layer was dried over anhydrous sodium sulfate (Na_2SO_4), filtered and evaporated to dryness to obtain **21a** (285 mg, 70% yield) as a yellow oil without further purification. ^{1}H NMR (400 MHz, $CDCl_3$) δ 2.24 (d, *J* = 6.4 Hz, 2H), 1.82 – 1.76 (m, 2H), 1.76 – 1.70 (m, 1H), 1.66 (dtd, *J* = 14.6, 3.9, 2.2 Hz, 1H), 1.54 – 1.40 (m, 2H), 0.91 (d, *J* = 6.6 Hz, 6H), 0.74 – 0.61 (m, 2H), 0.54 (dt, *J* = 13.0, 11.8 Hz, 1H). ^{13}C NMR (101 MHz, $CDCl_3$) δ 119.0, 43.3, 40.6, 34.6, 32.0, 24.7, 22.4. HRMS (ESI+) *m/z* calculated for $C_{10}H_{17}N$ $[M+Na]^+$ 174.1253, found 174.1258.

Synthesis of 19a

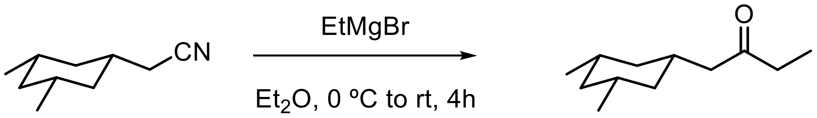

To a round-bottom flask equipped with a septum and kept under nitrogen charged with **21a** (222 mg, 1.47 mmol, 1 equiv) in diethyl ether (0.5 M) was added dropwisely 1.8 ml of ethylmagnesium bromide solution 1.0 M in THF (1.8 mmol, 1.2 equiv) at 0 ºC. The reaction was left stirring for 4 h, letting to warm up to room temperature. At this point, the reaction mixture was cooled down to 0 ºC, quenched with 6 ml of NH_4Cl $_{(aq,\ sat)}$ and washed with ether (3x). The combined organic layers was washed with H_2O and NaCl $_{(aq,\ sat)}$ and dried over anhydrous sodium sulfate (Na_2SO_4), filtered and evaporated to dryness, then the crude ketone was purified by flash chromatography over silica using hexane:ethyl acetate 100 :1 to obtain **19a** (132 mg, 50% yield) as a colourless oil. ^{1}H NMR (400

MHz, $CDCl_3$) δ 2.40 (q, *J* = 7.3 Hz, 2H), 2.27 (d, *J* = 6.9 Hz, 2H), 1.95 – 1.83 (m, 1H), 1.68 – 1.55 (m, 3H), 1.44 (tdq, *J* = 13.1, 6.6, 3.3 Hz, 2H), 1.04 (t, *J* = 7.3 Hz, 3H), 0.86 (d, *J* = 6.6 Hz, 6H), 0.49 (dtd, *J* = 13.2, 11.8, 5.3 Hz, 3H). ^{13}C NMR (101 MHz, $CDCl_3$) δ 211.7, 50.1, 43.8, 41.6, 36.7, 33.8, 32.2, 22.6, 7.8. HRMS (ESI+) *m/z* calculated for $C_{12}H_{22}O$ $[M+Na]^+$ 205.1563, found 205.1569.

Synthesis of intermediate C3

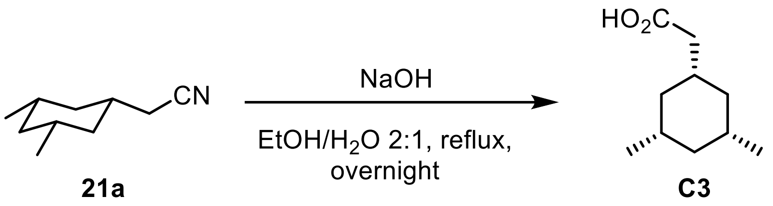

A round-bottom flask was charged with **21a** (200 mg, 1.3 mmol, 1 equiv), NaOH (524 mg, 13.1 mmol, 10 equiv) and 2.6 ml of a mixture of EtOH: water (2:1), then the reaction mixture was heated slowly to 80 ºC and left stirring overnight. At this point, the reaction mixture was cooled down to room temperature, evaporated almost the all solvent and diluite with ethyl acetate. The reaction mixture was washed with 1M HCl (3x) and NaCl $_{(aq,\ sat)}$. The organic layer was dried over anhydrous sodium sulfate (Na_2SO_4), filtered and evaporated to dryness to obtain **C3** (133 mg, 60% yield) as a white solid without further purification. ^1H NMR (400 MHz, $CDCl_3$) δ 2.23 (d, *J* = 7.0 Hz, 2H), 1.85 (dddd, *J* = 15.4, 8.2, 7.1, 3.6 Hz, 1H), 1.72 (ddd, *J* = 14.6, 4.0, 2.2 Hz, 2H), 1.64 (dtt, *J* = 12.8, 3.6, 1.8 Hz, 1H), 1.45 (tdq, *J* = 13.1, 6.7, 3.4 Hz, 2H), 0.88 (d, *J* = 6.6 Hz, 6H), 0.64 – 0.44 (m, 3H). ^{13}C NMR (101 MHz, $CDCl_3$) δ 179.1, 43.7, 41.8, 41.2, 34.5, 32.2, 22.5. ESI-MS *m/z* found for $C_{10}H_{18}O_2$ $[M-H]^-$ 169.1

Synthesis of 18a

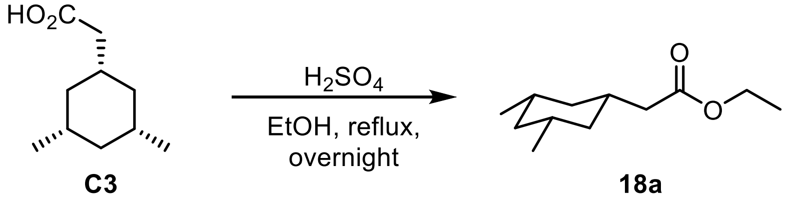

A round-bottom flask was charged with **C3** (200 mg, 1.2 mmol, 1 equiv), 3 ml of ethanol and one drop of sulfuric acid, then the reaction mixture was heated to reflux and left stirring overnight. At this point, the reaction mixture was cooled down to room temperature, evaporated almost the all solvent and diluite with ethyl acetate. The reaction mixture was washed with H_2O (3x) and NaCl $_{(aq, sat)}$. The organic layer was dried over anhydrous sodium sulfate (Na_2SO_4), filtered and evaporated to dryness, then the crude was purified by flash chromatography over silica using hexane:ethyl acetate 100 :1 to obtain **18a** (127 mg, 53% yield) as a yellow oil. ^{1}H NMR (400 MHz, $CDCl_3$) δ 4.13 (q, *J* = 7.2 Hz, 2H), 2.17 (d, *J* = 7.1 Hz, 2H), 1.83 (dddt, *J* = 15.6, 8.4, 7.1, 3.6 Hz, 1H), 1.72 – 1.55 (m, 3H), 1.44 (dddt, *J* = 14.9, 11.7, 6.6, 3.3 Hz, 2H), 1.26 (t, *J* = 7.1 Hz, 3H), 0.87 (d, *J* = 6.6 Hz, 6H), 0.62 – 0.43 (m, 3H). ^{13}C NMR (101 MHz, $CDCl_3$) δ 173.2, 60.1, 43.8, 42.2, 41.3, 34.7, 32.2, 22.6, 14.3. HRMS (ESI+) *m/z* calculated for $C_{11}H_{22}O_2$ $[M+Na]^+$ 221.1512, found 221.1504.

Synthesis of 20a

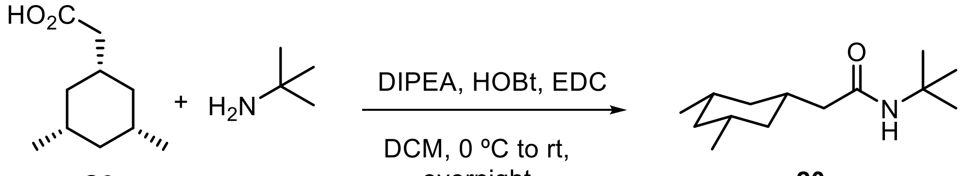

C3 **20a**

To a round-bottom flask equipped with a septum and kept under nitrogen charged with tert-Butylamine (93 μl, 1.0 equiv., 0.9 mmol) and dry CH_2Cl_2 (9 mL), was added Diisopropyl Ethylamine (166 μL, 1.0 equiv., 0.9 mmol) dropwise at 0 ºC. At this point, the carboxylic acid **C3** (171 mg, 1.0 equiv., 0.9 mmol), HOBt (132 mg, 1.1 equiv) and EDC (140 mg, 1.0 equiv, 0.9 mmol), were added in this order as solids. The reaction was left stirring overnight, letting to warm up to room temperature.The reaction mixture was successively washed with the following solutions: $NaHCO_3$ $_{(aq, sat)}$, Citric Acid (10 wt% Aq.), NaCl $_{(aq, sat)}$. The organic layer was dried over anhydrous sodium sulfate (Na_2SO_4), filtered and evaporated to dryness, then the crude was purified by flash chromatography over silica using hexane:ethyl acetate 1 :1 to obtain **18a** (180 mg, 89% yield) as a white solid. ^{1}H NMR (400 MHz, $CDCl_3$) δ 5.19 (s, 1H), 1.93 (d, *J* = 7.4 Hz, 2H), 1.84 (dddt, *J* = 14.8, 11.4, 7.6, 3.6 Hz, 1H), 1.72 – 1.59 (m, 3H), 1.50 – 1.39 (m, 2H), 1.35 (s, 9H), 0.87 (d, *J* = 6.6 Hz, 6H), 0.49 (qd, *J* = 12.0, 4.7 Hz, 3H). ^{13}C NMR (101 MHz, $CDCl_3$) δ 171.8, 51.1, 45.8, 43.9, 41.4, 35.1, 32.2, 28.9, 22.6. HRMS (ESI+) *m/z* calculated for $C_{14}H_{27}NO$ $[M+Na]^+$ 248.1985, found 248.1990.

Synthesis of 24a

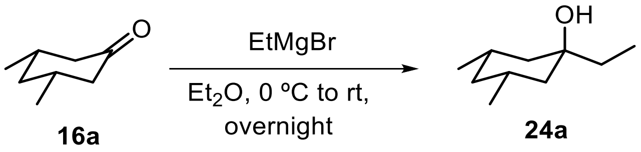

To a round-bottom flask equipped with a septum and kept under nitrogen charged with **16a** (126 mg, 1.0 mmol, 1 equiv) in diethyl ether (0.75 M) was added dropwisely 1.2 ml of ethylmagnesium bromide solution 1.0 M in THF (1.2 mmol, 1.2 equiv) at 0 ºC. The reaction was left stirring overnight, letting to warm up to room temperature. At this point, the reaction mixture was cooled down to 0 ºC, quenched with NH_4Cl $_{(aq, sat)}$ and washed with Et_2O (3x). The combined organic layers was washed with H_2O and NaCl $_{(aq, sat)}$ and dried over anhydrous sodium sulfate (Na_2SO_4), filtered and evaporated to dryness, then the crude alcohol was purified by flash chromatography over silica using hexane:ethyl acetate 10 :1 to **24a** (76 mg, 54% yield) as a white solid ^1H NMR (400 MHz, $CDCl_3$) δ 1.77 (tdq, *J* = 12.1, 6.5, 3.3 Hz, 2H), 1.66 (dtd, *J* = 14.6, 3.6, 1.9 Hz, 1H), 1.58 (q, *J* = 2.1 Hz, 1H), 1.56 – 1.53 (m, 1H), 1.45 (q, *J* = 7.5 Hz, 2H), 1.10 (s, 1H), 0.94 – 0.81 (m, 11H), 0.49 (q, *J* = 12.1 Hz, 1H).^{13}C NMR (101 MHz, $CDCl_3$) δ 72.6, 44.9, 43.8, 36.8, 27.8, 22.4, 7.4. HRMS (ESI+) *m/z* calculated for $C_{10}H_{20}O$ $[M+Na]^+$ 156.1514, found 156.1522.

Synthesis of 25a

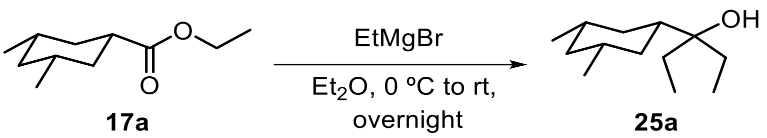

To a round-bottom flask equipped with a septum and kept under nitrogen charged with **17a** (306 mg, 1.7 mmol, 1 equiv) in diethyl ether (0.75 M) was added dropwisely 3.7 ml of ethylmagnesium bromide solution 1.0 M in THF (3.7 mmol, 2.2 equiv) at 0 ºC. The reaction was left stirring overnight, letting to warm up to room temperature. At this point, the reaction mixture was cooled down to 0 ºC, quenched with NH_4Cl $_{(aq, sat)}$ and washed with Et_2O (3x). The combined organic layers was washed with H_2O and NaCl $_{(aq, sat)}$ and dried over anhydrous sodium sulfate (Na_2SO_4), filtered and evaporated to dryness, then the crude alcohol was purified by flash chromatography over silica using hexane:ethyl acetate 10 :1 to obtain **25a** (230 mg, 70% yield) as a yellow oil. ^1H NMR (400 MHz,

$CDCl_3$) δ 1.64 (m, 3H), 1.59 – 1.54 (m, 1H), 1.52 – 1.31 (m, 6H), 1.04 (s, 1H), 0.90 (d, *J* = 6.6 Hz, 6H), 0.85 (t, *J* = 7.5 Hz, 6H), 0.73 – 0.61 (m, 2H), 0.50 (dt, *J* = 12.9, 11.7 Hz, 1H). ^{13}C NMR (101 MHz, $CDCl_3$) δ 75.6, 44.1, 43.7, 35.0, 32.7, 28.1, 22.9, 7.6. HRMS (ESI+) *m/z* calculated for $C_{13}H_{26}O$ $[M+Na]^+$ 221.1876, found 221.1876.

Synthesis of 26a

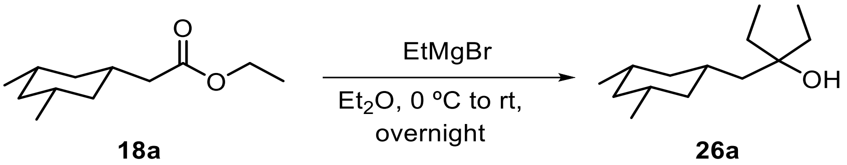

For the synthesis of **26a** was used the same procedure of **25a**, the crude mixture was purified by flash chromatography over silica using hexane:ethyl acetate 10:1 and the product was concentrated to dryness. The product was isolated as a yellow oil (86 mg, 52% yield). ^1H NMR (400 MHz, $CDCl_3$) δ 1.74 (ddt, *J* = 12.8, 4.0, 2.1 Hz, 2H), 1.61 (dtt, *J* = 12.6, 3.5, 1.9 Hz, 1H), 1.52 – 1.45 (m, 5H), 1.40 (tdd, *J* = 11.6, 5.9, 3.3 Hz, 2H), 1.32 (d, *J* = 5.5 Hz, 2H), 1.04 (s, 1H), 0.89 – 0.83 (m, 12H), 0.62 – 0.52 (m, 2H), 0.52 – 0.43 (m, 1H). ^{13}C NMR (101 MHz, $CDCl_3$) δ 75.6, 46.0, 44.0, 44.0, 33.1, 32.7, 31.6, 22.9, 8.1. HRMS (ESI+) *m/z* calculated for $C_{14}H_{28}O$ $[M+Na]^+$ 235.2032, found 235.2042.

Synthesis of 28a

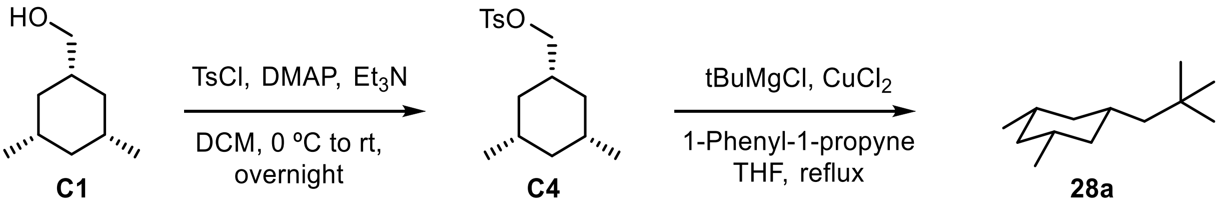

A round-bottom flask equipped with a septum and kept under nitrogen was charged with alcohol **C1** (980 mg, 6.9 mmol, 1 equiv), triethylamine (1.7 ml, 12.4 mmol, 1.8 equiv), DMAP (42 mg, 0.3 mmol, 0.05 eq) and 35 ml of dry dichloromethane, then the reaction mixture was cooled to 0 °C. At this point, TsCl (1.6 g, 8.3 mmol, 1.2 equiv) was added portion wise as a solid under nitrogen flow into the solution, and the reaction was left stirring overnight. The reaction mixture was successively washed with the following solutions: H_2O, 1M HCl, $NaHCO_3$ $_{(aq, sat)}$, NaCl $_{(aq, sat)}$. The organic layer was dried over anhydrous sodium sulfate (Na_2SO_4), filtered and evaporated to dryness to obtain **C4** (1.9

g, 97% yield). The crude intermediate **C4** was immediately used for the next step without further purification.

A round-bottom flask equipped with a septum and kept under nitrogen was charged with **C4** (1 g, 3.4 mmol, 1 equiv), phenyl-1-propyne (105 μl, 0.85 mmol, 0.25 equiv), $CuCl_2$ (45 mg, 0.33 mmol, 0.1 eq) and 7 ml of dry THF, then the reaction mixture was cooled to 0 °C. At this point, was added a solution of tBuMgCl (1.7 M in THF, 4.0 mL, 6.7 mmol, 2.0 eq) into the reaction mixture. Then the reaction was heated slowly up to reflux (from 0º C to 66ºC, increasing 10ºC every 5 min) and was left stirring overnight. The reaction mixture was cooled at 0ºC, quenched by the addition of $NH_4Cl_{(aq, sat)}$ and washed with pentane 3 times. The organic layer was dried over anhydrous sodium sulfate (Na_2SO_4), filtered and evaporated to dryness. The crude mixture was purified by flash chromatography over silica using pentane as eluent and the product **28a** was isolated as a colourless oil (240 mg, 40% yield). ^{1}H NMR (400 MHz, $CDCl_3$) δ 1.69 – 1.55 (m, 3H), 1.43 – 1.29 (m, 3H), 1.07 (d, *J* = 4.8 Hz, 2H), 0.88 (s, 9H), 0.86 – 0.84 (m, 6H), 0.58 – 0.39 (m, 3H). ^{13}C NMR (101 MHz, $CDCl_3$) δ 51.8, 44.7, 44.0, 34.2, 32.8, 31.2, 30.2, 22.9. GC-MS (CI) $[M + H]^+$ m/z = 184.1

1.3.3 Synthesis of substrates 29a – 31a

Synthesis of 29a

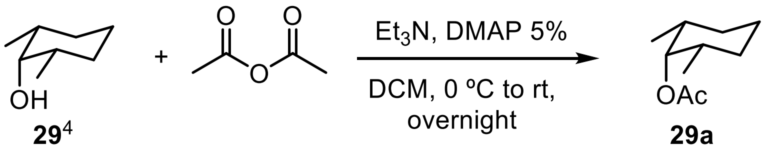

Following the general conditions of *ester synthesis protocol A*, the crude mixture was purified by flash chromatography over silica using hexane:ethyl acetate 50:1 and the product was concentrated to dryness. The product was isolated as a colourless oil (170 mg, 73% yield).

^{1}H NMR (400 MHz, $CDCl_3$) δ 4.31 (t, *J* = 10.3 Hz, 1H), 2.08 (s, 3H), 1.78 – 1.69 (m, 2H), 1.67 – 1.60 (m, 1H), 1.57 – 1.42 (m, 2H), 1.29 – 1.25 (m, 1H), 1.15 – 1.02 (m, 2H), 0.85 (d, *J* = 6.5 Hz, 6H). ^{13}C NMR (101 MHz, $CDCl_3$) δ 171.4, 83.1, 37.9, 34.0, 21.2, 18.7, 18.6. HRMS (ESI+) *m/z* calculated for $C_{10}H_{18}O_2$ $[M+Na]^+$ 193.1199, found 193.1205.

Synthesis of 30a

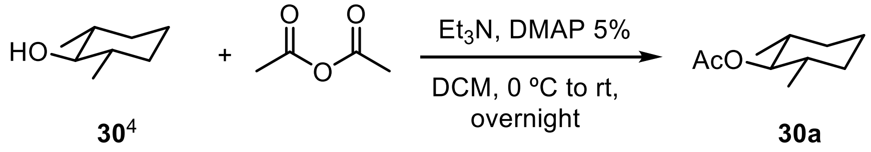

Following the general conditions of *ester synthesis protocol A*, the crude mixture was purified by flash chromatography over silica using hexane:ethyl acetate 50:1 and the product was concentrated to dryness. The product was isolated as a colourless oil (135 mg, 69% yield).

^{1}H NMR (400 MHz, $CDCl_3$) δ 5.03 (t, *J* = 2.5 Hz, 1H), 2.08 (s, 3H), 1.77 – 1.69 (m, 1H), 1.66 – 1.54 (m, 2H), 1.44 – 1.35 (m, 2H), 1.32 – 1.21 (m, 3H), 0.83 (d, *J* = 6.8 Hz, 6H). ^{13}C NMR (101 MHz, $CDCl_3$) δ 171.4, 76.7, 36.3, 28.6, 25.9, 21.0, 18.5. HRMS (ESI+) *m/z* calculated for $C_{10}H_{18}O_2$ $[M+Na]^+$ 193.1199, found 193.1202.

Synthesis of 31a

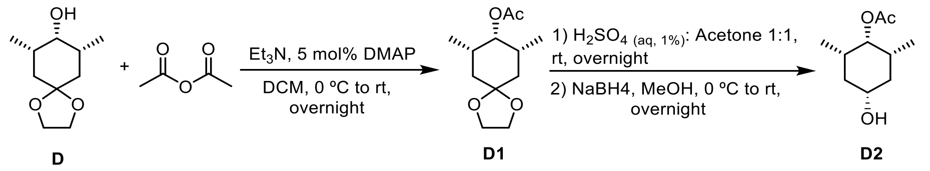

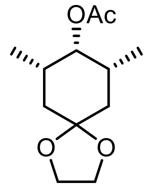

Synthesis of intermediate D1

A round-bottom flask equipped with a septum and kept under nitrogen was charged with a 0.40 M solution of the alcohol **D**[10] (980 mg, 5.3 mmol, 1 equiv) in dry dichloromethane, triethylamine (1.1 ml, 8 mmol, 1,5 equiv) and DMAP (32 mg, 5 mol %), then the reaction mixture was cooled to 0 °C. The acetic anhydride (750 µl, 8 mmol, 1,5 equiv) was added dropwise over 10 minutes and the reaction was left stirring overnight, letting the temperature rise to room temperature. At this point, HCl 1M solution was added until pH~1 and then diluted with dichloromethane. The organic layer was separated from the acid aqueous layer. The aqueous layer was extracted with dichloromethane (2x) and the organic layers were combined. The organic layer was washed with saturated $NaHCO_3$ and dried over anhydrous sodium sulfate (Na_2SO_4), filtered and evaporated to dryness to obtain the **D1** (1.1 g, 89% yield) as a yellow liquid without further purification. ^{1}H NMR (400 MHz, $CDCl_3$) δ 5.02 (t, *J* = 2.4 Hz, 1H), 4.00 – 3.90 (m, 4H), 2.08 (s, 3H), 1.97 (dddd, *J* = 14.1, 11.9, 7.1, 4.8 Hz, 2H), 1.60 – 1.49 (m, 4H), 0.86 (d, *J* = 6.8 Hz, 6H). ^{13}C NMR (101 MHz, $CDCl_3$) δ 171.3, 109.1, 75.3, 64.5, 64.4, 37.5, 33.7, 20.9, 17.8. ESI-MS *m/z* found for $C_{12}H_{20}O_4$ $[M+Na]^+$ 251.3.

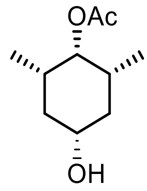

Synthesis of intermediate D2

A round-bottom flask was charged with **D1** (800 mg, 3.5 mmol, 1 equiv), 2 ml of acetone and 2 ml of H_2SO_4 1 % solution, then the reaction mixture was left stirring overnight at room temperature. At this point, saturated $NaHCO_3$ was added until pH~7 and then diluted with ether. The organic layer was separated from the aqueous layer. The aqueous layer was extracted with ether (2x) and the organic layers were combined. The organic layer was washed with brine and dried over anhydrous

sodium sulfate (Na_2SO_4), filtered and evaporated to dryness to obtain the crude ketone product, which was directly reduced without further purification.

A round-bottom flask equipped with a septum and kept under nitrogen, was cooled to 0º C, then was charged with the ketone obtained and 10 ml of dry MeOH. At this point, $NaBH_4$ (266 mg, 7 mmol, 2 equiv) was added slowly as a solid under nitrogen flow. The reaction was left stirring overnight, letting to warm up to room temperature. After this, the reaction mixture was evaporated to remove almost all methanol and diluted with ether and water. The organic layer was separated from the aqueous layer. The aqueous layer was extracted with ether (2x) and the organic layers were combined. The organic layer was washed with brine and dried over anhydrous sodium sulfate (Na_2SO_4), filtered and evaporated to dryness, then the crude was purified by flash chromatography over silica using hexane:ethyl acetate 3 :1 to obtain **D2** (300 mg, 46% yield, over 2 steps) as a white solid. ^{1}H NMR (400 MHz, $CDCl_3$) δ 4.97 (s, 1H), 3.70 (td, *J* = 11.1, 4.4 Hz, 1H), 2.10 (s, 3H), 1.70 (tq, *J* = 10.6, 3.6 Hz, 4H), 1.40 (d, *J* = 4.7 Hz, 1H), 1.37 – 1.23 (m, 2H), 0.89 (d, *J* = 6.6 Hz, 6H). ^{13}C NMR (101 MHz, $CDCl_3$) δ 171.2, 74.9, 70.0, 38.0, 34.5, 20.9, 17.8. ESI-MS *m/z* found for $C_{10}H_{18}O_3$ $[M+Na]^+$ 209.1.

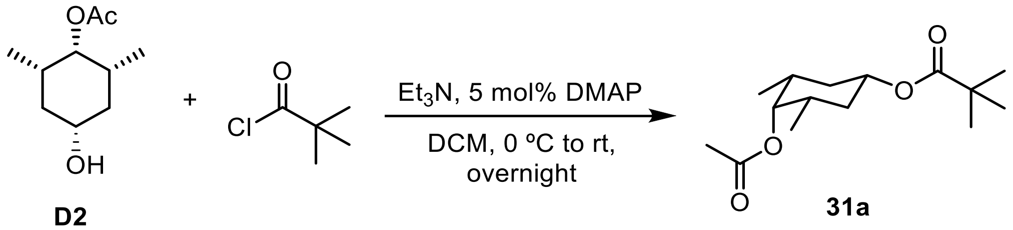

D2 31a

A round-bottom flask equipped with a septum and kept under nitrogen was charged with a 0.40 M solution of the alcohol **D2** (228 mg, 1.2 mmol, 1 equiv) in dry dichloromethane, triethylamine (250 μl, 1.8 mmol, 1,5 equiv) and DMAP (7.3 mg, 5 mol %), then the reaction mixture was cooled to 0 °C. The acetic anhydride (170 μl, 1.8 mmol, 1,5 equiv) was added dropwise over 10 minutes and the reaction was left stirring overnight, letting the temperature rise to room temperature. At this point, HCl 1M solution was added until pH~1 and then diluted with dichloromethane. The organic layer was separated from the acid aqueous layer. The aqueous layer was extracted with dichloromethane (2x) and the organic layers were combined. The organic layer was washed with saturated $NaHCO_3$ and dried over anhydrous sodium sulfate (Na_2SO_4), filtered and evaporated to dryness, then the crude was purified by flash chromatography over silica using hexane:ethyl acetate 50 :1 to obtain **31a** (300 mg, 91% yield) as a colourless solid. ^{1}H NMR (400 MHz, $CDCl_3$) δ 4.99 (t, *J* = 2.5 Hz, 1H),

4.75 (tt, J = 11.4, 4.5 Hz, 1H), 2.12 (s, 3H), 1.85 – 1.66 (m, 4H), 1.36 (q, J = 12.2 Hz, 2H), 1.19 (s, 9H), 0.88 (d, J = 6.7 Hz, 6H). ^{13}C NMR (101 MHz, $CDCl_3$) δ 178.3, 171.1, 74.8, 71.7, 38.8, 34.4, 33.8, 27.3, 21.0, 17.8. HRMS (ESI+) *m/z* calculated for $C_{15}H_{26}O_4$ $[M+Na]^+$ 293.1723, found 293.1734.

1.4 Synthesis of the complexes

Triflate complexes with (S,S) and (R,R) configuration, Mn(pdp), Mn(^{NMe2}pdp), Mn(^{DMM}pdp), Mn(^{TIPS}pdp), Fe(^{TIPS}pdp), Mn(^{TIBS}pdp), Mn(^{TIPS}mcp), Mn(^{TIPS}ecp) were syntehsized according to reported procedures. [11–15]

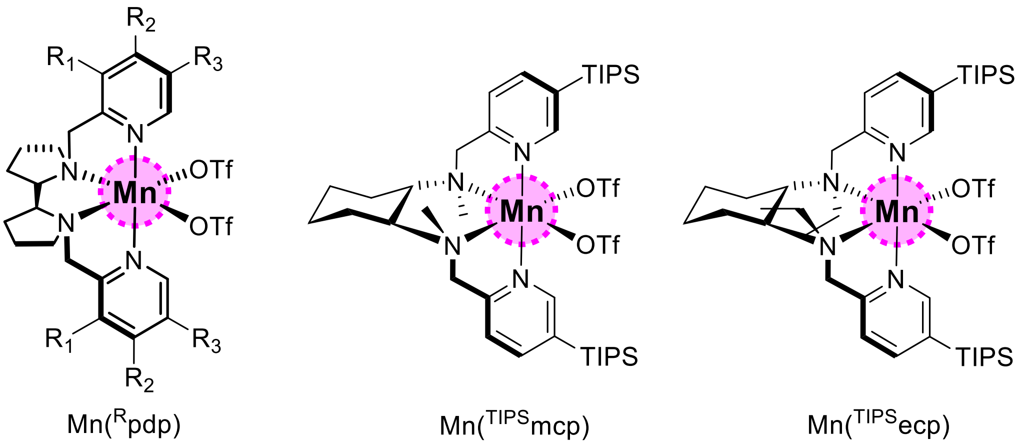

Catalyst	R1	R2	R3
Mn(pdp)	-H	-H	-H
Mn(^{NMe2}pdp)	-H	-NMe2	-H
Mn(^{DMM}pdp)	-Me	-OMe	-Me
Mn(^{TIPS}pdp)	-H	-H	-TIPS
Fe(^{TIPS}pdp)	-H	-H	-TIPS
Mn(^{TIBS}pdp)	-H	-H	-TIBS

Figure S1. *Schematic representation of the catalysts used in this work*

1.4.1 Synthesis of (*S,S*)-Mn(^{TIPS}mpea)

Synthesis of the ligand ^{TIPS}mpea

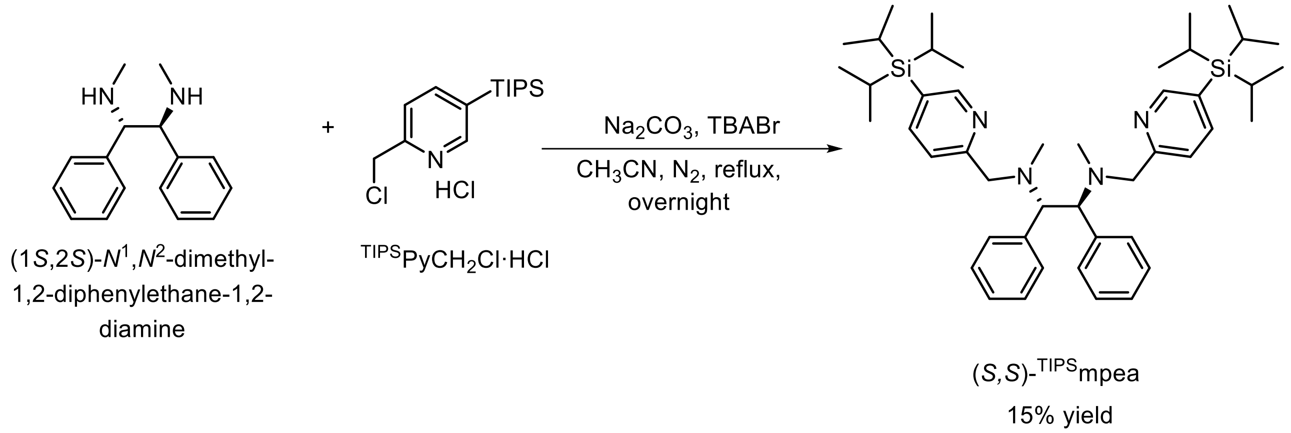

In a microwave crimper vial equipped with a magnetic stir bar was added (1S,2S)-*N^1*,*N^2*-dimethyl-1,2-diphenylethane-1,2-diamine[16] (60 mg, 0.25 mmol, 1 eq), ^{TIPS}PyCH$_2$Cl·HCl[11] (319 mg, 0.5 mmol, 2 eq), Na_2CO_3 (424 mg, 4 mmol, 16 eq), TBABr (5 mg) and 4 ml of anhydrous CH_3CN. The reaction mixture was refluxed overnight under N_2. At this point, the crude reaction was diluted with 15 ml of water and extracted with 30 ml of DCM (3x), the organic layer were combined and dried over Na_2SO_4. The crude mixture was purified by silica column (CH_2Cl_2:MeOH:NH_3 95:5:1) to provide **^{TIPS}mpea** (28 mg, yield 15%) as a-yellow oil. ^{1}H NMR (400 MHz, $CDCl_3$) δ 8.49 (s, 2H), 7.56 (t, *J* = 6.2 Hz, 4H), 7.12 – 6.97 (m, 10H), 4.40 (s, 2H), 3.80 (d, *J* = 14.3 Hz, 2H), 3.58 (d, *J* = 14.2 Hz, 2H), 2.19 (s, 6H), 1.36 – 1.29 (m, 6H), 1.01 (dd, *J* = 7.5, 2.3 Hz, 36H). ^{13}C NMR (101 MHz, $CDCl_3$) δ 160.9, 154.5, 143.3, 135.7, 129.7, 127.8, 127.4, 127.0, 122.8, 68.1, 60.1, 37.7, 18.6, 18.6, 10.8. HRMS (ESI+) *m/z* calculated for $C_{46}H_{70}N_4Si_2$ $[M+H]^+$ 735.5212, found 735.5211.

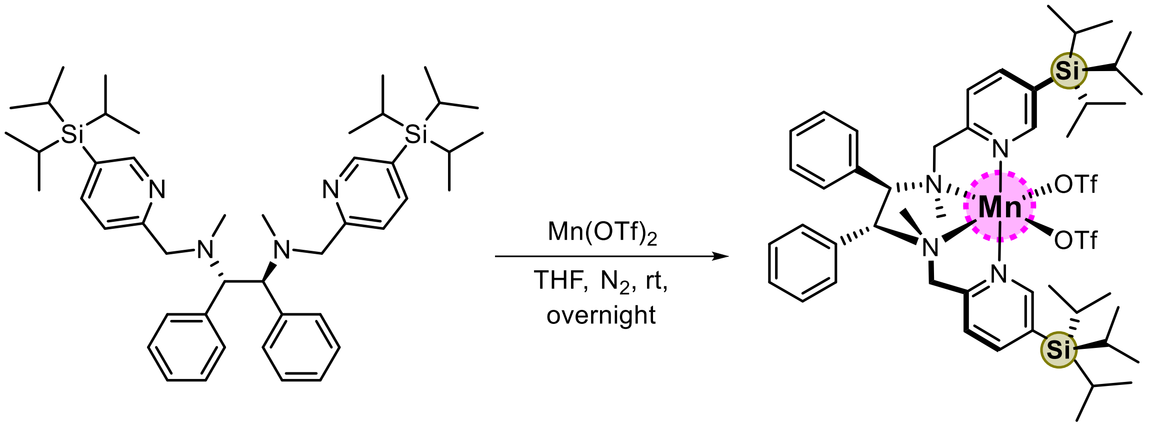

(*S*,*S*)-^{TIPS}mpea (*S*,*S*)-Mn(^{TIPS}mpea)

In glovebox, to a vial containing the ligand **(*S,S*)-^{TIPS}mpea** (25.6 mg, 0.035 mmol, 1 eq) in anhydrous THF (1 mL) was added as a solid Mn(OTf)$_2$ (12.3 mg, 0.035 mmol, 1 eq). After stirring overnight, the reaction mixture was dried under vacuum and dissolved in CH_2Cl_2 (1 mL). This solution was filtered through a short pad of Celite© and kept crystalizing by layering with hexane (2 mL). After one day, white crystals of **(*S,S*)-Mn(^{TIPS}mpea)** were obtained (29 mg, 76% yield). Anal. Calcd for $C_{48}H_{70}F_6MnN_4O_6S_2Si_2$: C, 52.97; H, 6.48; N, 5.15. Found: C, 52.80; H, 6.40; N, 5.45 %. FT-IR (ATR) ν, cm-1: 2944- 2867 (C-H)sp$_3$, 1593,1455,1312,1233,1214,1164,1038, 995, 882, 861, 759, 702, 683, 638, 573, 514. HRMS (ESI+) *m/z* calculated for $C_{48}H_{70}F_6MnN_4O_6S_2Si_2$, [M-OTf]$^+$: 938.4034, found: 938.4011.

1.5 Oxidation reactions

Hydrogen peroxide solutions employed in the oxidation reactions were prepared by diluting commercially available hydrogen peroxide (50% H_2O_2 solution in water, Aldrich) in acetonitrile to achieve a 0.9 M. The purity of the substrates synthesized as described above was in all cases >97%.

1.5.1 Reaction protocol for catalysis method A

Substrate (100 µmol, 1 eq) and the corresponding catalyst (1 µmol, 1 mol%) were dissolved in MeCN or fluorinated alcohol solvent (TFE or HFIP) to provide a concentration of the substrate to 0.25 M, in a 10 ml vial equipped with a stir bar cooled at 0 ºC for HFIP in a water/ice bath, or at -40 ºC for TFE and MeCN in a CH_3CN/N_2(liq) bath. Carboxylic acid (135 µl, 1.7 mmol, 17 eq) were added directly to the solution. Then, 0.9 M solution of H_2O_2 in the corresponding solvent (1 eq) diluted from commercially available H_2O_2 water solution was delivered over a period of 30 minutes by syringe

pump into the solution. Acetylation protocol of the alcohol products of the crude was performed to obtain GC analysis.

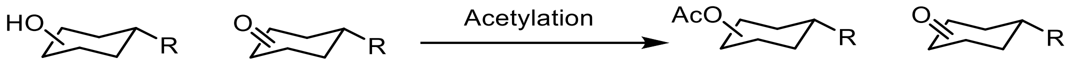

Acetylation protocol: 1ml of AcOEt was added to the reaction mixture, then the solution was quickly filtered through a silica plug, which was subsequently rinsed with 2 x 1 mL AcOEt. At this point the solvent was removed by rotatory evaporation, and to the crude was added 1 ml of CH_2Cl_2 and 0.1 ml of 1-methylimidazole. The solution was cooled at 0 ºC in a water/ice bath. Then, 1 ml of acetic anhydride was added to the solution over 1 minute, and the reaction was left stirring at the same temperature. After 20 minutes, 1 ml of water was added, and the mixture was left stirring for other 10 minutes. At this point, an internal standard (biphenyl) was added to the solution. After that, the organic layer was separated from the aqueous layer. The organic layer was successively washed with the following solutions: 1M H_2SO_4, $NaHCO_{3\ (aq,\ sat)}$, H_2O. After that, the organic phase was dried over anhydrous sodium sulfate (Na_2SO_4) plug, which was subsequently rinsed with 2 x 1 mL CH_2Cl_2.
GC analysis of the solution provided substrate conversions and product yields relative to the internal standard integration, the oxidized products were identified by comparison to the GC retention time of pure products.
Commercially unavailable products were synthesized. Ee's were determined by GC equipped with a chiral column, the oxidized products were identified by comparison to the GC retention time of racemate products.

1.5.2 Reaction protocol for catalysis method B

Substrate (100 µmol, 1 equiv.), amino acid (15 µmol, 15 mol%) and the corresponding catalyst (1 µmol, 1 mol%) were dissolved in 0.4 ml of acetonitrile (CH_3CN) [for some substrates the reaction was performed in 1:1 solution TFE:MeCN to solubilize them], in a 10 ml vial equipped with a stir bar cooled at -35 ºC in a CH_3CN/N_2(liq) bath. Then, 389 µL of a 0.9 M hydrogen peroxide solution in CH_3CN (3.5 equiv.) diluted from commercially available H_2O_2 (50% H_2O_2 solution in water, Aldrich) was delivered over a period of 30 minutes by syringe pump into the solution. The reaction mixture was left under stirring for further 5 minutes. At this point, an internal standard (biphenyl) was added and the solution was quickly filtered through a two layers plug of silica and basic alumina, which was subsequently rinsed with 2 x 1 mL AcOEt. GC analysis of the solution provided substrate conversions and product yields relative to the internal standard integration. Commercially

unavailable products were identified by a combination of ^1H, $^{13}C\{^1H\}$-NMR analysis, and HRMS. Enantiomeric excesses were determined by GC equipped with a chiral column, the oxidized products were identified by comparison to the GC retention time of racemate products.

1.5.3 General Procedure for product isolation

A 10 mL round bottom flask was charged with: substrate (500 µmol, 1 equiv.), amino acid (15 µmol, 15 mol%), catalyst (5 µmol, 1.0 mol%) and CH_3CN (2 mL). Then, the mixture was cooled at -35 ºC in an CH_3CN/N_2(liq) bath under magnetic stirring. At this point, 1.94 mL of a 0.9 M hydrogen peroxide solution in CH_3CN (3.5 equiv.) were added by syringe pump over a period of 30 min at -35°C. The reaction mixture was left under stirring for further 15 minutes. At this point, 10 mL of an aqueous $NaHCO_3$ saturated solution were added to the mixture. The resultant solution was extracted with CH_2Cl_2 (3 x 10 mL). Organic fractions were combined, dried over $MgSO_4$, and the solvent was evaporated to dryness. Then, the crude oxidized product was purified by flash chromatography over silica gel.

1.5.4 Synthesis of oxidation products

The following oxidation products of **1a** at C-3 were obtained from the ketone **1f**(K-3), that was prepared accordingly to the reported procedure.[11] The oxidation product at C-4 were prepared from commercially available cis-4-aminocyclohexan-1-ol and trans-4-aminocyclohexan-1-ol.

***Synthesis of* 1b(OH-3 ax) and 1c(OH-3 eq)**

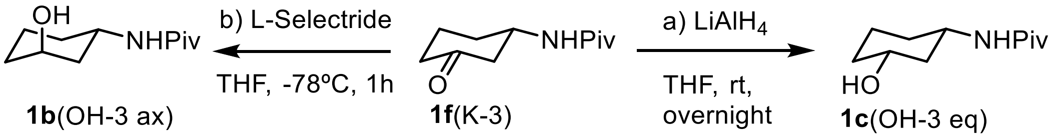

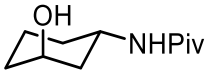

1b(OH-3 ax)

An oven-dried round bottom flask under N_2 cooled at –80 ºC was charged with L-selectride solution in anhydrous THF 1 M (1.5 equiv), then was added anhydrous THF to afford a concentration of 0.5 M of L-selectride. At this point, was added a solution of ketone **1f**(K-3) in anhydrous THF 1.5 M (1.0 equiv) drop wise. The temperature was maintained for 1 h at -78ºC. At this point, reaction was

careful quenched with NH_4Cl (aq, sat), and the aqueous layer was extracted with ethyl acetate three times. The combined organic layer was dried over anhydrous $MgSO_4$, filtered and evaporated to dryness, then the crude was purified by flash chromatography over silica using hexane:ethyl acetate 1 :1 to obtain **1b**(OH-3 ax) (43 mg, 65 % yield) as a white solid. ^1H NMR (400 MHz, $CDCl_3$) δ 5.46 (s, 1H), 4.17 (qt, *J* = 8.6, 3.8 Hz, 1H), 4.05 (s, 1H), 1.93 – 1.75 (m, 4H), 1.66 – 1.46 (m, 4H), 1.34 – 1.26 (m, 1H), 1.18 (s, 9H). ^{13}C NMR (101 MHz, $CDCl_3$) δ 177.8, 66.9, 44.3, 39.8, 38.7, 32.9, 32.0, 27.7, 19.6. HRMS (ESI+) *m/z* calculated for $C_{11}H_{21}NO_2$ $[M+Na]^+$ 222.1470, found 222.1472.

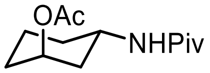

1b(OAc-3 ax), **1b**(OH-3 ax) was acetylated for GC and Chiral GC analysis were done using J&W CYCLOSIL-B. ^1H NMR (400 MHz, $CDCl_3$) δ 5.44 (s, 1H), 5.12 (s, 1H), 4.14 (dtd, *J* = 14.4, 6.9, 4.1 Hz, 1H), 2.08 (s, 4H), 1.94 (q, *J* = 5.7 Hz, 1H), 1.82 – 1.69 (m, 2H), 1.66 – 1.61 (m, 1H), 1.44 (ddd, *J* = 13.7, 10.6, 3.0 Hz, 2H), 1.20 (s, 9H). ^1H NMR (400 MHz, $CDCl_3$) δ 5.44 (s, 1H), 5.12 (s, 1H), 4.14 (dtd, *J* = 14.4, 6.9, 4.1 Hz, 1H), 2.07- 2.00 (m, 4H), 1.94 (q, *J* = 5.7 Hz, 1H), 1.82 – 1.69 (m, 2H), 1.66 – 1.61 (m, 1H), 1.44 (ddd, *J* = 13.7, 10.6, 3.0 Hz, 2H), 1.20 (s, 9H). ^{13}C NMR (101 MHz, $CDCl_3$) δ 177.8, 170.7, 44.3, 42.0, 38.7, 36.6, 32.2, 29.8, 29.7, 27.7, 21.5, 19.9.

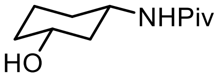

1c(OH-3 eq)

An oven-dried round bottom flask under N_2 was charged with sodium borohydride (3 equiv), and cooled at 0 ºC, then was added anhydrous MeOH to afford a concentration of 0.7 M of sodium borohydride. At this point, was added a solution of ketone **1f**(K-3) in anhydrous MeOH 1.5 M (1.0 equiv) drop wise. The reaction was allowed to warm to room temperature and stirring overnight. After this, the reaction mixture was careful quenched with NH_4Cl (aq, sat) solution at 0 ºC and the aqueous layer was extracted with DCM three times (2 x 10 mL) the organic fractions were dried over anhydrous $MgSO_4$, filtered and evaporated to dryness, then the crude was purified by flash chromatography over silica using hexane:ethyl acetate 1 :1 to obtain **1c**(OH-3 eq) (56 mg, 66 % yield) as a white solid. ^1H NMR (400 MHz, $CDCl_3$) δ 6.25 (s, 1H), 3.94 (dtd, *J* = 11.7, 8.0, 3.8 Hz, 2H), 2.10 – 1.93 (m, 1H), 1.88 – 1.66 (m, 3H), 1.57 (d, *J* = 3.6 Hz, 1H), 1.45 – 1.26 (m, 4H), 1.18 (s, 9H). ^{13}C NMR (101 MHz, $CDCl_3$) δ 177.5, 68.6, 45.6, 38.5, 34.1, 31.3, 27.6, 19.2. HRMS (ESI+) *m/z* calculated for $C_{11}H_{21}NO_2$ $[M+Na]^+$ 222.1470, found 222.1465.

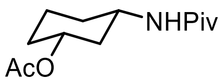 **1c**(OAc-3 eq), **1c**(OH-3 eq) was acetylated for GC and Chiral GC analysis were done using J&W CYCLOSIL-B. ^{1}H NMR (400 MHz, $CDCl_3$) δ 5.81 (s, 1H), 4.88 (tt, *J* = 8.6, 4.1 Hz, 1H), 4.01 – 3.88 (m, 1H), 2.14 (dtd, *J* = 13.5, 4.0, 1.2 Hz, 1H), 2.05 (s, 3H), 1.93 – 1.70 (m, 4H), 1.45 – 1.34 (m, 3H), 1.19 (s, 9H). ^{13}C NMR (101 MHz, $CDCl_3$) δ 177.4, 169.9, 71.1, 45.6, 38.5, 38.5, 37.1, 31.4, 30.6, 29.7, 27.6, 21.4, 20.0.

***Synthesis of* 1e(OH-4 eq) and 1d(OH-4 ax)**

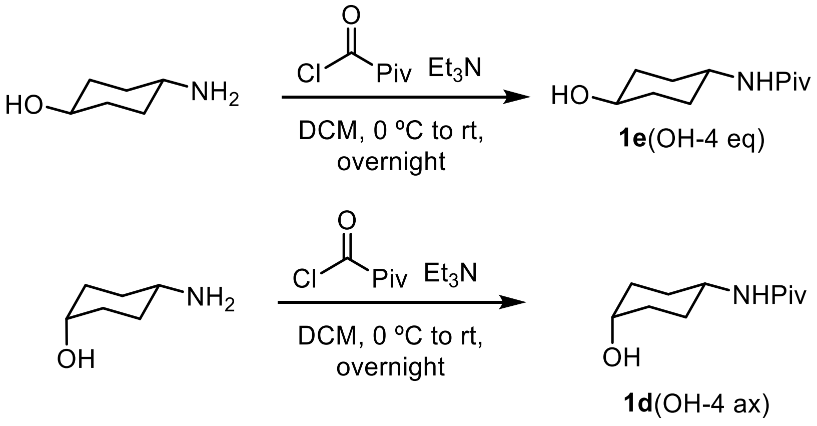

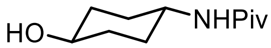 **1e**(OH-4 eq)

A round-bottom flask equipped with a septum and kept under nitrogen was charged with a 0.40 M solution of trans-4-aminocyclohexan-1-ol (1 equiv) in dry dichloromethane, triethylamine (1 equiv), then the reaction mixture was cooled to 0 °C. The pivaloyl chloride (0.9 equiv) was added dropwise over 10 minutes and the reaction was left stirring overnight, letting the temperature rise to room temperature. At this point, HCl 1M solution was added until pH~1 and then diluted with dichloromethane. The organic layer was separated from the acid aqueous layer. The aqueous layer was extracted with dichloromethane (2x) and the organic layers were combined. The organic layer was washed with saturated $NaHCO_3$ and dried over anhydrous sodium sulfate (Na_2SO_4), filtered and evaporated to dryness, obtaining **1e**(OH-4 eq) (33mg, 78% yield) without further purification. ^{1}H NMR (400 MHz, $CDCl_3$) δ 5.36 (s, 1H), 3.74 (tdt, *J* = 11.5, 7.7, 3.7 Hz, 1H), 3.61 (td, *J* = 10.6, 5.3 Hz, 1H), 2.07 – 1.90 (m, 4H), 1.50 – 1.32 (m, 3H), 1.25 (s, 1H), 1.17 (s, 10H). ^{13}C NMR (101 MHz, $CDCl_3$) δ 177.8, 70.0, 47.4, 38.6, 34.0, 30.9, 27.6. HRMS (ESI+) *m/z* calculated for $C_{11}H_{21}NO_2$ $[M+Na]^+$ 222.1470, found 222.1471.

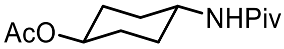 **1e**(OAc-4 eq), **1e**(OH-4 eq) was acetylated for GC analysis. ^{1}H NMR (400 MHz, $CDCl_3$) δ 5.39 (d, *J* = 7.8 Hz, 1H), 4.68 (tt, *J* = 10.8, 4.1 Hz, 1H), 3.77 (tdt, *J* = 11.5, 7.8, 3.7 Hz, 1H), 2.03 (s, 3H), 2.00 (t, *J* = 4.3 Hz, 3H), 1.56 – 1.42 (m, 2H), 1.18 (s, 10H). ^{13}C NMR (101 MHz, $CDCl_3$) δ 177.8, 170.7, 72.0, 47.1, 38.6, 30.6, 30.1, 27.6, 21.4.

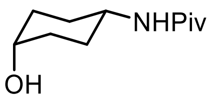 **1d**(OH-4 ax)

A round-bottom flask equipped with a septum and kept under nitrogen was charged with a 0.40 M solution of cis-4-aminocyclohexan-1-ol (1 equiv) in dry dichloromethane, triethylamine (1 equiv), then the reaction mixture was cooled to 0 °C. The pivaloyl chloride (0.9 equiv) was added dropwise over 10 minutes and the reaction was left stirring overnight, letting the temperature rise to room temperature. At this point, HCl 1M solution was added until pH~1 and then diluted with dichloromethane. The organic layer was separated from the acid aqueous layer. The aqueous layer was extracted with dichloromethane (2x) and the organic layers were combined. The organic layer was washed with saturated $NaHCO_3$ and dried over anhydrous sodium sulfate (Na_2SO_4), filtered and evaporated to dryness, obtaining **1d**(OH-4 ax) (45mg, 66% yield) without further purification.
Due to a probably conformational equilibrium in *cis*-N-(4-hydroxycyclohexyl)pivalamide, a peaks splitting was detected in ^{1}H and ^{13}C NMR. Whit acetylation, in **1d**(OAc-4 ax), this splitting disappear, probably due to the presence of a fixed conformation. ^{1}H NMR (400 MHz, $CDCl_3$) δ 5.77 – 5.33 (m, 1H), 3.94 (s, 1H), 3.83 (q, *J* = 6.2 Hz, 1H), 1.91 – 1.75 (m, 1H), 1.75 – 1.55 (m, 6H), 1.52 – 1.31 (m, 1H), 1.25 (s, 1H), 1.24 – 1.16 (m, 9H). ^{13}C NMR (101 MHz, $CDCl_3$) δ 177.7, 177.6, 68.2, 66.3, 46.9, 46.3, 38.9, 38.6, 38.6, 31.3, 29.7, 28.7, 27.8, 27.6, 27.3, 27.2. HRMS (ESI+) *m/z* calculated for $C_{11}H_{21}NO_2$ $[M+Na]^+$ 222.1470, found 222.1468.

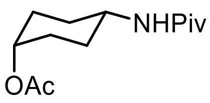 **1d**(OAc-4 ax), **1d**(OH-4 ax) was acetylated for GC analysis. ^{1}H NMR (400 MHz, $CDCl_3$) δ 5.51 (s, 1H), 4.94 (d, *J* = 3.5 Hz, 1H), 3.90 – 3.77 (m, 1H), 2.06 (s, 3H), 1.89 – 1.72 (m, 4H), 1.67 (ddd, *J* = 13.5, 11.1, 3.3 Hz, 2H), 1.50 (q, *J* = 9.7 Hz, 2H), 1.19 (s, 9H). ^{13}C NMR (101 MHz, $CDCl_3$) δ 177.6, 170.4, 69.0, 46.5, 38.6, 28.5, 27.6, 27.6, 21.4.

1.5.5 Reaction oxidation of 1a

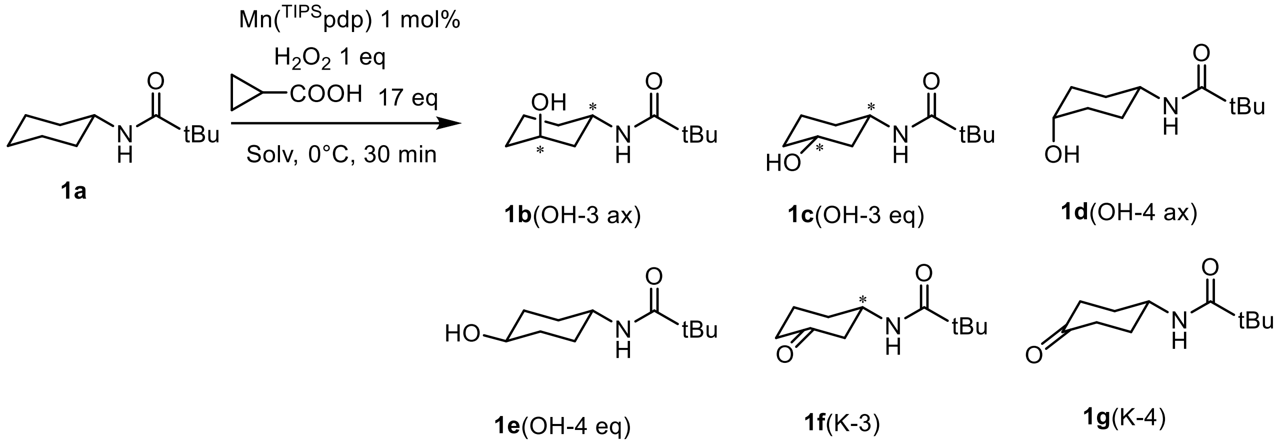

entry	cat	Solv	Conv %[a]	OH-3 (ee ax/ ee eq)%[a] [dr OH-3][b]	K-3 (ee) %[a]	OH-4/ K-4 % [dr OH-4][a,c]	C-3/C-4[d]
1	Mn(TIPSpdp)	MeCN[d]	99	-	88 (77)	-/ 7	6.4
2	Mn(TIPSpdp)	TFE	61	39 (93/40) [4.7]	18 (58)	7/1 [2.6]	4.4
1	Mn(TIPSpdp)	HFIP	67	45 (79/18) [3.3]	11 (41)	15/ <1 [3.6]	1.8
4	Mn(TIPSmcp)	MeCN[d]	99	-	90 (83)	-/6[e]	7.3
5	Mn(TIPSmcp)	TFE	50	28 (87/31) [2.8]	10 (59)	6/1 [2.3]	3.2
6	Mn(TIPSmcp)	HFIP	72	41 (85/22) [2.6]	9 (36)	19/<1 [3.8]	1.5

Table S1. *Catalysts screening of oxidation of 1a in different solvents* [a]Conversions and yields determined from crude reaction mixtures by GC. Ee's determined by Chiral GC analysis were done using J&W CYCLOSIL-B. [b]P1(OH-4)+P1(K4). [c]Normalized ratio. [d]H_2O_2 (3.5 equiv)

1.5.6 Reaction optimization for oxidation of 3a

Table S2. *Catalyst screening.*[a]

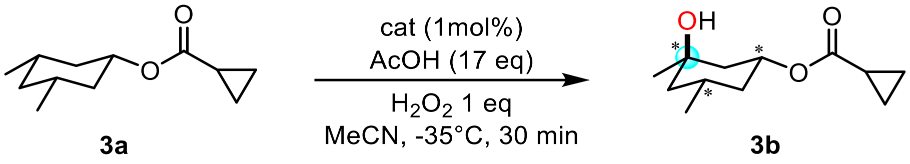

Entry	Catalyst	Modification	Conv. (%)	3b yield (%)	ee (%)[b]
1	Mn(pdp)		30	24	18
2	Mn(^{NMe2}pdp)		42	29	15
3	Mn(^{DMM}pdp)		39	32	rac
4	Mn(^{TIPS}pdp)		53	43	59
5	Mn(^{TIPS}pdp)	1 eq AcOH	13%	8%	57
6	Mn(^{TIPS}pdp)	1 eq AcOH, 3.5 eq H_2O_2	31%	25%	58
7	Mn(^{TIPS}pdp)	0.15 eq AcOH	5%	-	-
8	Mn(^{TIPS}pdp)	0.15 eq AcOH, 3.5 eq H_2O_2	5%	-	-
9	Fe(^{TIPS}pdp)		30	24	41
10	Mn(^{TIBS}pdp)		61	48	43
11	Mn(^{TIPS}mcp)		61	42	62
12	Mn(^{TIPS}mcp)[c]		67	31	57
13	Mn(^{TIPS}mcp)[d]		90	77	71
14	Mn(^{TIPS}mpea)		60	52	58
15	Mn(^{TIPS}ecp)		28	24	57

[a]Reaction conditions as described in general oxidation protocol A with (*S,S*)-catalysts using acetic acid as coligand. Conversions and yields determined by GC analysis of two or three different independent runs. [b]ee values determined by GC equipped with chiral column. [c]Reaction performed at 0º. [d]TFE instead of MeCN

***Table S3.** Coligand screening.*[a]

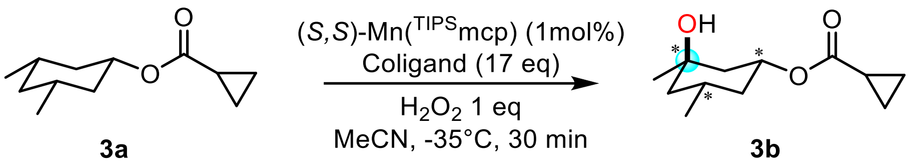

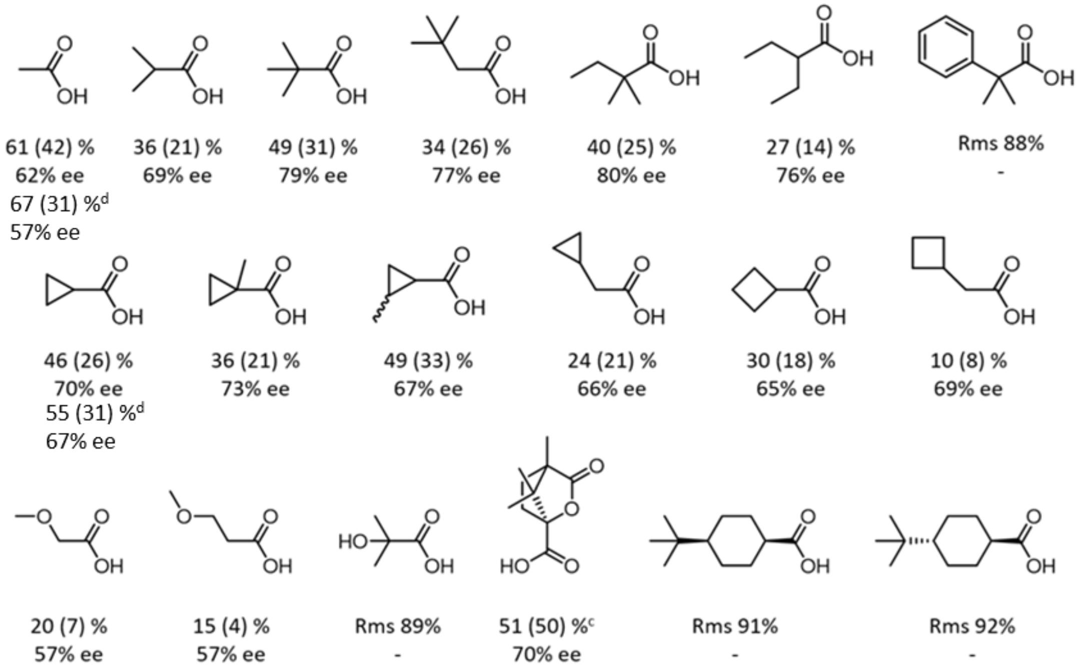

[a]Reaction conditions as described in general oxidation protocol A with (*S,S*)-Mn(^{TIPS}mcp) using different carboxylic acids as coligand. Conversions and yields determined by GC analysis of two or three different independent runs. [b]ee values determined by GC equipped with chiral column. [c]catalyst load 2.5 mol% [c]Reaction performed at 0º

Aminoacids coligand library was synthesized according to reported procedures.[17]

Table S4. *Catalyst screening using AAs as coligand.*[a]

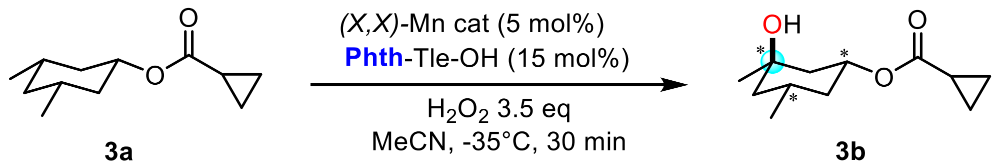

3a **3b**

Conv. (Yield) %
ee %[b]

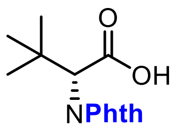

phthalimido-D-*tert*-leucine
Phth-D-Tle-OH

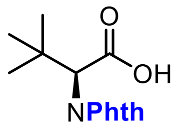

phthalimido-L-*tert*-leucine
Phth-L-Tle-OH

Entry	Catalyst	Chirality of AA	Conv. (%)[a]	3b yield (%)[a]	ee (%)[b]
1	(*S*,*S*)-Mn(^{TIPS}mcp)[c]	L	33	14	84
2	(*R*,*R*)-Mn(^{TIPS}mcp)[c]	L	39	20	89
3	(*R*,*R*)-Mn(^{TIPS}mcp)[c,e]	L	40	27	79
4	(*S*,*S*)-Mn(^{TIPS}mcp)[c,e]	L	51	36	82
5	(*R*,*R*)-Mn(^{TIPS}mcp)[d]	L	72	62	89
6	(*R*,*R*)-Mn(^{TIPS}mcp)	L	91	78	91
7	(*S*,*S*)-Mn(^{TIPS}mcp)	L	43	38	86
		D	92	82	90
8	(*R*,*R*)-Mn(^{TIPS}pdp)	L	91	88	93
8	(*R*,*R*)-Mn(^{TIPS}pdp)[d]	L	92	90	94
10	(*S*,*S*)-Mn(^{TIPS}pdp)[d]	L	52	40	86
		D	97	90	92
11	(*S*,*S*)-Fe(^{TIPS}pdp)	L	55	46	72
		D	48	30	62

12	(*S,S*)-Mn(^{TIBS}pdp)	L	67	61	84
		D	95	84	90
13	(*R,R*)-Mn(^{TIPS}ecp)	L	25	20	81
		D	23	16	79
14	(*S,S*)-Mn(^{TIPS}mpea)[f]	L	7	1	82
		D	10	8	87
15	(*R,R*)-Mn(^{DMM}pdp)	L	48	42	13
		D	48	43	20
16	(*Rac*)-Mn(^{TIPS}mcp)	L	58	57	41

[a]Reaction conditions as described in general oxidation protocol A with (*S,S*)-catalysts and (*R,R*)-catalysts. Conversions and yields determined by GC analysis of two or three different independent runs. [b]ee values determined by GC equipped with chiral column. [c]catalyst load 1 mol%, H_2O_2 load 1 eq. [d]catalyst load 1 mol%. [e]TFE instead of MeCN. [f]catalyst load 3 mol%.

Table S5. *Optimization of Catalyst and AAs as coligand loading.*[a]

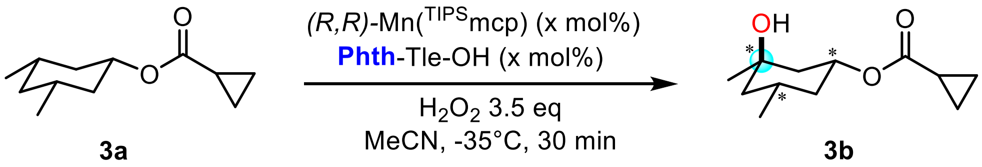

Entry	Catalyst Load	AA Load	Conv. (%)[a]	3b yield (%)[a]	ee (%)[b]
1	1	5	20	1	-
2	1	10	40	20	92
3	1	15	43	26	92
4	1	40	40	29	91
5	4	40	94	93	93
6	4	20	81	80	93

7	5	15	92	91	93
8[b]	1	15	92	90	94

[a]Reaction conditions as described in general oxidation protocol A with (*R,R*)-catalysts. Conversions and yields determined by GC analysis of two or three different independent runs. [b](*R,R*)-Mn(^{TIPS}pdp) instead of (*R,R*)-Mn(^{TIPS}mcp)

Table S6. *Optimization of substrate concentration.*[a]

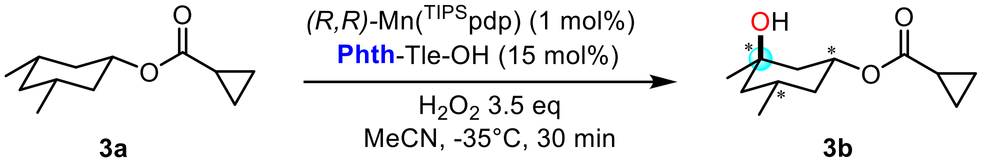

Entry	Conc 3a [M]	Conv. (%)[a]	3b yield (%)[a]	ee (%)[b]
1	1,25	88	85	94
2	0,5	97	93	94
3	0,25	97	94	94
4	0,05	71	70	94

[a]Reaction conditions as described in general oxidation protocol A with (*R,R*)-catalysts. Conversions and yields determined by GC analysis of two or three different independent runs.

Table S7. *Protecting group screening of L-tert-leucine coligand.*[a]

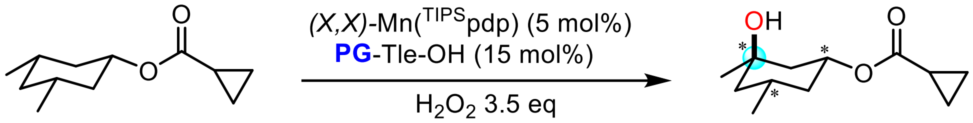

3a → **3b**

Yield% (Conv.)[a] %
ee %[b]

Entry	1	2	3	4
Chirality of catalyst (X,X)	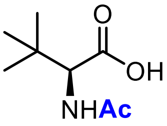	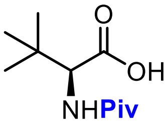	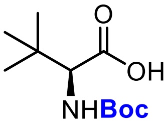	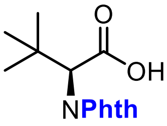
(S,S)	**26%** (30%) *77% ee*	**43%** (46%) *81% ee*	**72%** (75%) *87% ee*	**48%** (52%) *87% ee*
(R,R)	**36%** (42%) *90% ee*	**40%** (44%) *81% ee*	**37%** (46%) *85% ee*	**88%** (91%) *93% ee*

[a]Reaction conditions as described in general oxidation protocol A with (*R,R*)-catalysts. Conversions and yields determined by GC analysis of two or three different independent runs. [b]ee values determined by GC equipped with chiral column.

Table S8. *Side-chain screening of Phthalimido-protected aminoacid.*[a]

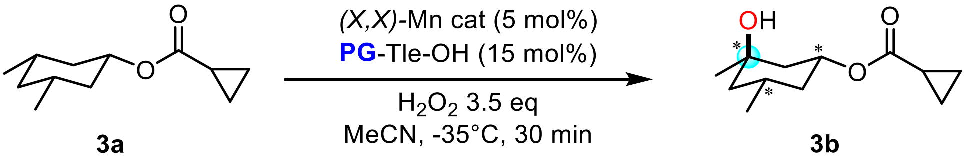

Yield% (Conv.) %
ee %[b]

Entry	1	2	3	4	5
Chirality of catalyst (X,X)	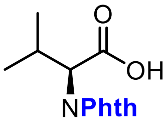	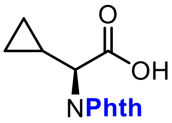	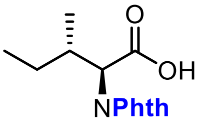	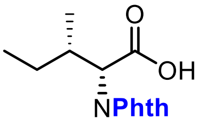	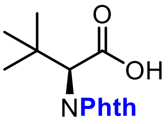
(S,S)	**56%** (64%) *81% ee*	**70%** (88%) *82% ee*	**60%** (70%) *81% ee*	**73%** (80%) *83% ee*	**48%** (52%) *87% ee*
(R,R)	**64%** (77%) *82% ee*	**73%** (90%) *80% ee*	**67%** (83%) *82% ee*	**46%** (56%) *85% ee*	**88%** (91%) *93% ee*

[a]Reaction conditions as described in general oxidation protocol A with (*R,R*)-catalysts. Conversions and yields determined by GC analysis of two or three different independent runs. [b]ee values determined by GC equipped with chiral column.

Table S9. *Oxidation of* ***3ax***[a]

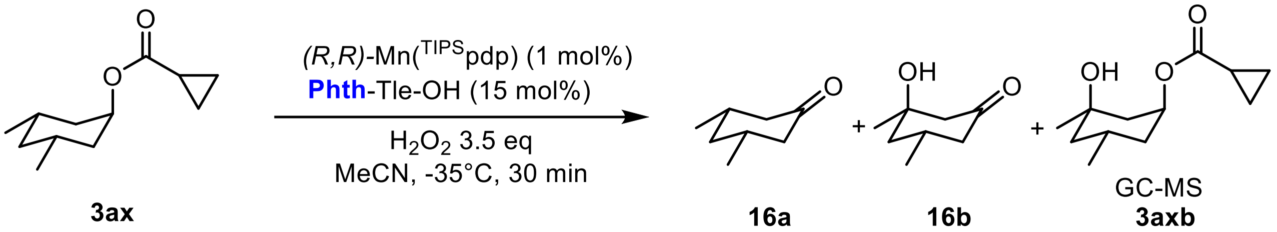

Entry	Modification	Conv. (%)[a]	16a yield (%)	16b yield [ee](%)[b]	3ax yield (%)[c]
1	-	10	4	4 [79]	1
2	0.5 eq H_2O_2	5	2	-	-
3	17 eq AcOH instead of AA	60	4	23 [60]	5

[a]Reaction conditions as described in general oxidation protocol A with (*R,R*)-catalysts. Conversions and yields determined by GC analysis of two or three different independent runs. [b]ee values determined by GC equipped with chiral column. [c] GC-MS (CI) $[M + NH_4]^+$ m/z = 230.2

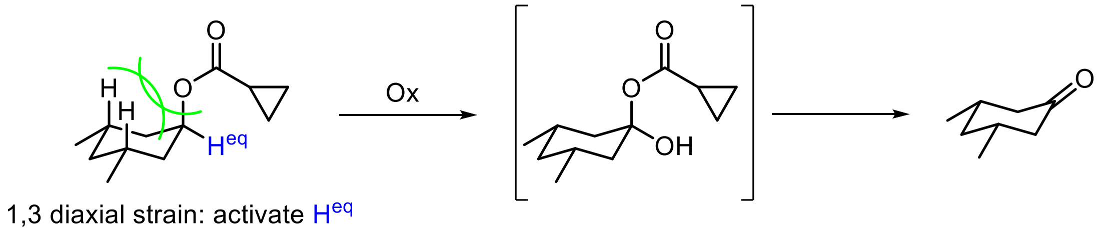

Oxidation of **3ax**: *A plausible explanation*[18]

1.5.7 Characterization of oxidation products

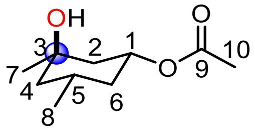

2b: Purified by flash chromatography over silica using hexane:ethyl acetate 5:1 and the product was concentrated to dryness. The product was isolated as a yellow oil (60% yield, 95% ee). ^{1}H NMR (400 MHz, $CDCl_3$) δ 5.05 (tt, *J* = 11.6, 4.4 Hz, 1H, *C1[1H]*), 2.02 (s, 3H, *C10[3H]*), 1.98 (ddt, *J* = 10.9, 4.4, 2.2 Hz, 2H, *C2[1H] C6[1H]*), 1.92 (ddd, *J* = 12.3, 6.8, 3.5 Hz, 1H, *C5[1H]*), 1.64 (dt, *J* = 4.0, 2.1 Hz, 1H, *C4[1H]*), 1.31 (dd, *J* = 12.8, 11.5 Hz, 1H, *C2[1H]*), 1.26 (s, 3H, *C7[3H]*), 1.22 (s, 1H, *O[H]*), 1.02 – 0.95 (m, 1H, *C4[1H]*), 0.94 (d, *J* = 6.6 Hz, 3H, *C8[3H]*), 0.88 (d, *J* = 11.9 Hz, 1H, *C6[1H]*). ^{13}C NMR (101 MHz, $CDCl_3$) δ 170.6 (*C9*), 71.3 (*C3*), 70.7 (*C1*), 46.6 (*C4*), 43.7 (*C2*), 39.7 (*C2*), 31.7 (*C7*), 26.3 (*C5*), 21.8 (*C10*), 21.4 (*C8*). HRMS (ESI+) *m/z* calculated for $C_{10}H_{18}O_3$ $[M+Na]^+$ 209.1148, found 209.1146. Chiral GC analysis were done using HP-CHIRAL-20B. $[\alpha]_D^{24}$ -5.881 ($CHCl_3$, c 0.160).

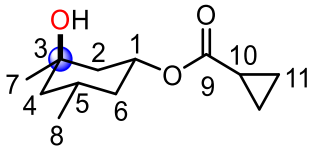

3b: Purified by flash chromatography over silica using hexane:ethyl acetate 5:1 and the product was concentrated to dryness. The product was isolated as a yellow oil (87% yield, 94% ee). ^{1}H NMR (400 MHz, $CDCl_3$) δ 5.04 (tt, *J* = 11.6, 4.4 Hz, 1H, *C1[1H]*), 2.05- 1.97 (m, 2H, *C2[1H] C6[1H]*), 1.96 – 1.86 (m, 1H, C5*[1H]*), 1.63 (ddt, *J* = 13.7, 4.2, 2.2 Hz, 1H, *C4[1H]*), 1.55 (tt, *J* = 8.0, 4.6 Hz, 1H, *C10[1H]*), 1.38 (bs, 1H, *O[H]*), 1.32 (dd, *J* = 12.9, 11.6 Hz, 1H, *C2[1H]*), 1.26 (s, 3H, *C7[3H]*), 1.02 – 0.87 (m, 7H, *C6[1H] C4[1H] C8[3H] C11[2H]*), 0.83 (dt, *J* = 8.2, 3.5 Hz, 2H, *C11[2H]*). ^{13}C NMR (101 MHz, $CDCl_3$) δ 174.4 (*C9*), 71.3 (*C3*), 70.6 (*C1*), 46.5 (*C4*), 43.8 (*C2*), 39.8 (*C6*), 31.7 (*C7*), 26.3 (*C5*), 21.8 (*C8*), 13.1 (*C10*), 8.3 (*C11*). HRMS (ESI+) *m/z* calculated for $C_{12}H_{20}O_3$ $[M+Na]^+$ 235.1305, found 235.1308. Chiral GC analysis were done using J&W CYCLOSIL-B. $[\alpha]_D^{24}$ -5.279 ($CHCl_3$, c 0.305).

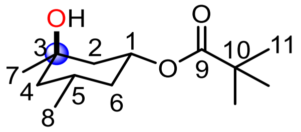

4b: Purified by flash chromatography over silica using hexane:ethyl acetate 5:1 and the product was concentrated to dryness. The product was isolated as a yellow oil (82% yield, 93% ee). ^{1}H NMR (400 MHz, $CDCl_3$) δ 5.01 (tt, *J* = 11.5, 4.4 Hz, 1H, *C1[1H]*), 1.99 (ddt, *J* = 12.9, 4.6, 2.1 Hz, 2H, *C2[1H] C6[1H]*), 1.96 – 1.88 (m, 1H, *C5[1H]*), 1.63 (dt, *J* = 15.8, 2.1 Hz, 1H, *C4[1H]*), 1.35

– 1.30 (m, 1H, *C_2[1H]*), 1.28 (s, 1H, *O[H]*), 1.26 (s, 3H, *C_7[3H]*), 1.17 (s, 9H, *C_{11}[3H] C_{11}[3H] C_{11}[3H]*), 1.02 – 0.96 (m, 1H, *C_4[1H]*), 0.94 (d, *J* = 6.5 Hz, 3H, *C_8[3H]*), 0.91 (d, *J* = 11.7 Hz, 1H, *C_2[1H]*). ^{13}C NMR (101 MHz, $CDCl_3$) δ 178.0 (*C_9*), 71.3 (*C_3*), 70.2 (*C_1*), 46.6 (*C_4*), 43.6 (*C_2*), 39.6 (*C_6*), 38.6 (*C_{10}*), 31.7 (*C_7*), 27.1 (*C_{11}*), 26.3 (*C_5*), 21.8 (*C_8*). HRMS (ESI+) *m/z* calculated for $C_{13}H_{24}O_3$ $[M+Na]^+$ 251.1618, found 251.1623. Chiral GC analysis were done using J&W CYCLOSIL-B. $[\alpha]_D^{24}$ -5.048 ($CHCl_3$, c 0.493).

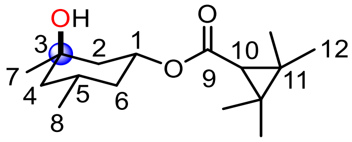

5b: Purified by flash chromatography over silica using hexane:ethyl acetate 5:1 and the product was concentrated to dryness. The product was isolated as a yellow oil (75% yield, 97% ee). was concentrated to dryness. ^{1}H NMR (400 MHz, $CDCl_3$) δ 5.02 (tt, *J* = 11.6, 4.4 Hz, 1H, *C_1[1H]*), 2.05 – 1.95 (m, 2H, *C_2[1H] C_6[1H]*), 1.92 (ddt, *J* = 12.3, 6.8, 3.5 Hz, 1H, *C_5[1H]*), 1.66 – 1.58 (m, 1H, *C_4[1H]*), 1.38 (bs, 1H, *O[H]*), 1.31 (dd, *J* = 13.0, 11.5 Hz, 1H, *C_2[1H]*), 1.25 (s, 3H, *C_7[3H]*), 1.24 (s, 3H, *C_{12}[3H]*), 1.23 (s, 3H, *C_{12}[3H]*), 1.17 (s, 6H, *C_{12}[3H] C_{12}[3H]*), 1.13 (s, 1H, *C_{10}[1H]*), 1.03 – 0.86 (m, 5H, *C_6[1H] C_4[1H] C_8[3H]*). ^{13}C NMR (101 MHz, $CDCl_3$) δ 171.7 (*C_9*), 71.3 (*C_3*), 69.9 (*C_1*), 46.6 (*C_4*), 44.0 (*C_2*), 40.0 (*C_6*), 36.0 (*C_{10}*), 31.7 (*C_7*), 29.8 (*C_{11}*), 26.4 (*C_5*), 23.6 (*C_{12}*), 21.8 (*C_8*), 16.6 (*C_{12}*), 16.6 (*C_{12}*). HRMS (ESI+) *m/z* calculated for $C_{16}H_{28}O_3$ $[M+Na]^+$ 291.1931, found 291.1930. Chiral GC analysis were done using J&W CYCLOSIL-B. $[\alpha]_D^{24}$ -3.055 ($CHCl_3$, c 0.266).

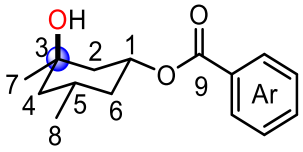

6b was obtained following grame-scale oxidation protocol. A 100 mL round bottom flask was charged with **6a** (1g, 4.30 mmol, 1 equiv.), Phth-Tle-OH (169 mg, 0.65 mmol, 15 mol%), the catalyst ***(R,R)*Mn(^{TIPS}pdp)** (43mg, 0.43 3mol, 1.0 mol%) and CH_3CN (17.2 mL). Then, the mixture was cooled at -35 ºC in an CH_3CN/N_2(liq) bath under magnetic stirring. At this point, 16.7 mL of a 0.9 M hydrogen peroxide solution in CH_3CN (3.5 equiv.) were added by syringe pump over a period of 30 min at -35°C. The reaction mixture was left under stirring for further 15 minutes. At this point, 10 mL of an aqueous $NaHCO_3$ saturated solution were added to the mixture. The resultant solution was extracted with CH_2Cl_2 (3 x 10 mL). Organic fractions were combined, dried over $MgSO_4$, and the solvent was evaporated to dryness. Then, the crude oxidized product was purified by flash chromatography over silica using hexane:ethyl acetate 3:1 to obtain **6b** (829mg, 78% yield, 90% ee) as a yellow oil. ^{1}H NMR (400 MHz, $CDCl_3$) δ 8.08 – 7.97 (m, 2H, *Ar*), 7.60 – 7.50 (m, 1H, *Ar*), 7.43 (dd,

J = 8.4, 7.1 Hz, 2H, *Ar*), 5.31 (tt, J = 11.5, 4.5 Hz, 1H, *C_1[1H]*), 2.14 (m, 2H, *C_2[1H] C_6[1H]*), 2.02 (qdd, J = 13.4, 6.7, 3.5 Hz, 1H, *C_5[1H]*), 1.68 (ddt, J = 13.7, 4.0, 2.1 Hz, 1H, *C_4[1H]*), 1.66 (bs, *O[H]*), 1.47 (dd, J = 12.9, 11.5 Hz, 1H, *C_2[1H]*), 1.30 (s, 3H, *C_7[3H]*), 1.14 – 1.00 (m, 2H, *C_4[1H] C_6[1H]*), 0.98 (d, J = 6.7 Hz, 3H, *C_8[3H]*). ^{13}C NMR (101 MHz, $CDCl_3$) δ 166.2 (*C_9*), 132.9 (*Ar*), 131.0 (*Ar*), 129.7 (*Ar*), 128.4 (*Ar*), 71.6(*C_1*), 71.5 (*C_3*), 46.8 (*C_4*), 43.9 (*C_2*), 39.9 (*C_6*), 31.9 (*C_7*), 26.5 (*C_5*), 22.0 (*C_8*). HRMS (ESI+) *m/z* calculated for $C_{15}H_{20}O_3$ [M+Na]$^+$ 271.1305, found 271.1314. Chiral SFC analysis were done using Chiralpack IC-3. $[\alpha]_D^{24}$ -6.881 ($CHCl_3$, c 0.123).

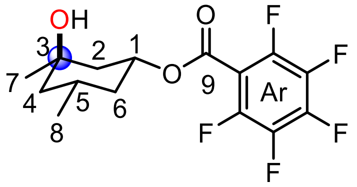

7b: Purified by flash chromatography over silica using hexane:ethyl acetate 5:1 and the product was concentrated to dryness.The product was isolated as a white solid (75% yield, 87% ee). ^{1}H NMR (400 MHz, $CDCl_3$) δ 5.37 (tt, J = 11.5, 4.5 Hz, 1H, *C_1[1H]*), 2.14 (tdt, J = 12.7, 4.5, 2.0 Hz, 2H, *C_2[1H] C_6[1H]*), 2.02 (ttd, J = 12.3, 6.8, 6.2, 3.5 Hz, 1H, *C_5[1H]*), 1.72 – 1.61 (m, 1H, *C_4[1H]*), 1.44 (dd, J = 12.8, 11.6 Hz, 1H, *C_2[1H]*), 1.41 (s, 1H, *O[H]*), 1.31 (s, 3H, *C_7[3H]*), 1.09 – 1.04 (m, 1H, *C_6[1H]*), 1.04 – 1.00 (m, 1H, *C_4[1H]*), 0.98 (d, J = 6.6 Hz, 3H, *C_8[3H]*). ^{13}C NMR (101 MHz, $CDCl_3$) δ 158.5 (*C_9*), 146.5- 146.3 (m, *Ar*), 144.4- 143.7 (m, *Ar*), 141.8- 141.5 (m, *Ar*), 139.0- 138.7 (m, *Ar*), 136.5- 136.2 (m, *Ar*), 109.1- 108.7 (m, *Ar*), 74.2 (*C_1*), 71.4 (*C_3*), 46.5 (*C_4*), 43.4 (*C_2*), 39.4 (*C_6*), 31.6 (*C_7*), 26.4 (*C_5*), 21.7 (*C_8*). ^{19}F NMR (377 MHz, $CDCl_3$) δ -139.76 - -139.87 (m), -150.33 (tt, J = 20.8, 4.3 Hz), -161.54 (tt, J = 20.7, 5.9 Hz). HRMS (ESI+) *m/z* calculated for $C_{15}H_{15}F_5O_3$ [M+Na]$^+$ 361.0834, found 361.0834. Chiral GC analysis were done using J&W CYCLOSIL-B. $[\alpha]_D^{24}$ -7.565 ($CHCl_3$, c 0.350).

Oxidation of 8a

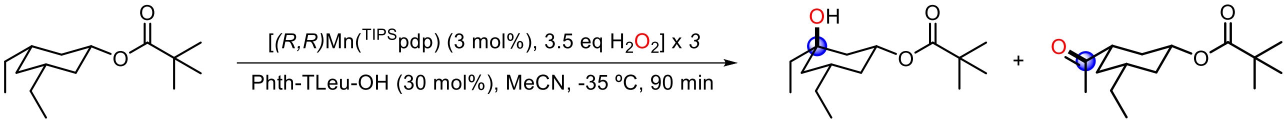

8a
conv 65%

8b
42% yield *(85 % ee)*

8c
19% yield *(rac)*

The yields of **8b** and **8c** were determined by GC analysis, using biphenyl as internal standard. **8a** was oxidized following oxidation protocol B with 30 mol% of the aminoacid. (Phth-Tle-OH) using 3 mol% of *(R,R)*-Mn(^{TIPS}pdp) as catalyst. The catalyst were added twice to the reaction mixture and re-exposed two times to 3.5 equivalent of H_2O_2.**8b** and **8c** were obtained following oxidation protocol A with 17 eq. of acetic acid (AcOH), 1 mol% of *(R,R)*-Mn(^{TIPS}pdp) and 2 eq of H_2O_2.

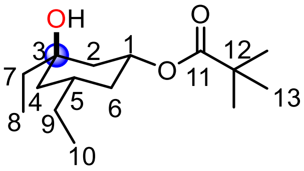

8b

Purified by flash chromatography over silica using pentane: diethyl ether (5:1). The product was isolated as a colorless oil (40% yield, 74 % ee). ^{1}H NMR (400 MHz, $CDCl_3$) δ 4.97 (tt, *J* = 11.4, 4.5 Hz, 1H, *C_1[1H]*), 2.02 – 1.95 (m, 1H, *C_6[1H]*), 1.84 (ddt, *J* = 12.8, 4.5, 2.2 Hz, 1H, *C_2[1H]*), 1.67 (m, 1H, *C_5[1H]*), 1.57 (ddt, *J* = 13.5, 4.0, 2.1 Hz, 1H, *C_4[1H]*), 1.43 (q, *J* = 7.5 Hz, 2H, *C_7[2H]*), 1.21 (ddd, *J* = 7.3, 5.8, 3.7 Hz, 3H, *C_2[1H] C_9[1H] O[H]*), 1.11 (s, 9H, *C_{13}[9H]*), 0.89 – 0.79 (m, 9H, *C_4[1H] C_6[1H] C_8[3H] C_9[1H] C_{10}[3H]*). ^{13}C NMR (101 MHz, $CDCl_3$) δ 178.4 (*C_{11}*), 73.7 (*C_3*), 70.9 (*C_1*), 42.4 (*C_4*), 42.1 (*C_2*), 39.0 (*C_{12}*), 37.9 (*C_6*), 37.2 (*C_7*), 33.0 (*C_5*), 29.7 (*C_9*), 27.5 (*C_{13}*), 11.7 (*C_8*), 7.8 (*C_9*). HRMS (ESI+) *m/z* calculated for $C_{15}H_{28}O_3$ $[M+Na]^+$ 279.1931, found 279.1931. Chiral GC analysis were done using J&W CYCLOSIL-B.

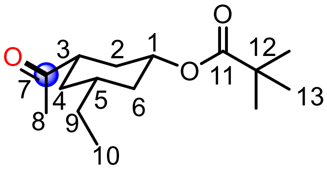

8c

Purified by flash chromatography over silica using pentane: diethyl ether (5:1). The product was isolated as a colorless oil (31% yield, 4 % ee). ^{1}H NMR (400 MHz, $CDCl_3$) δ 4.72 (tt, *J* = 11.4, 4.4 Hz, 1H, *C_1[1H]*), 2.49 (tt, *J* = 12.5, 3.4 Hz, 1H, *C_3[1H]*), 2.16 (s, 3H, *C_8[3H]*), 2.15 – 2.09 (m, 1H, *C_2[1H]*),

2.03 – 1.96 (m, 1H, *C_6[1H]*), 1.91 (ddt, *J* = 13.2, 3.6, 1.8 Hz, 1H, *C_4[1H]*), 1.36 – 1.27 (m, 3H, *C_2[1H] C_5[1H] C_9[1H]*), 1.18 (d, *J* = 1.1 Hz, 9H, *C_{13}[9H]*), 1.02 – 0.85 (m, 6H, *C_4[1H] C_6[1H] C_9[1H] C_{10}[3H]*). ^{13}C NMR (101 MHz, $CDCl_3$) δ 210.5 (*C_7*), 178.4 (*C_{11}*), 72.2 (*C_1*), 49.4 (*C_3*), 39.0 (*C_{12}*), 37.6 (*C_6*), 37.4 (*C_5*), 34.0 (*C_4*), 33.5 (*C_2*), 29.7 (*C_9*), 28.6 (*C_8*), 27.5 (*C_{13}*), 11.6 (*C_{10}*). HRMS (ESI+) *m/z* calculated for $C_{15}H_{26}O_3$ $[M+Na]^+$ 277.1774, found 277.1774. Chiral GC analysis were done using J&W CYCLOSIL-B.

Oxidation of 9a

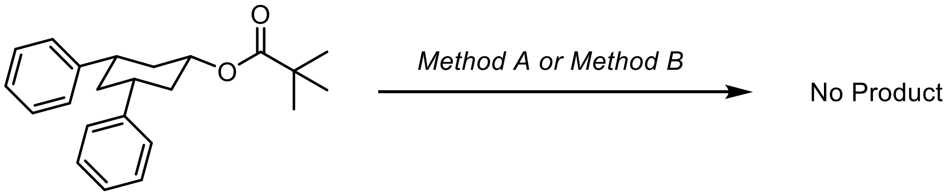

9a
rsm >95%

Oxidation of **9a** using method A or B were unsuccessful with no formation of the desired product.

Oxidation of 10a

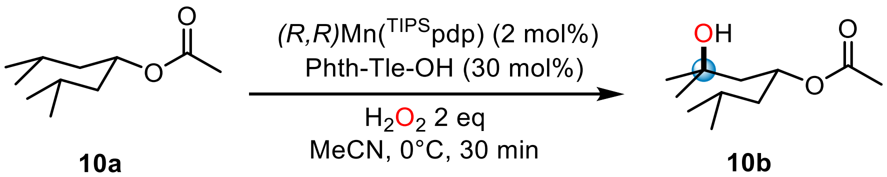

10a conv. 35% → **10b** yield 28% *(rac ee)*

The yields of **10b** was determined by GC analysis, using biphenyl as internal standard. **10a** was oxidized following oxidation protocol B with 15 mol% of the aminoacid. (Phth-Tle-OH), 1 mol% of *(R,R)*-Mn(^{TIPS}pdp) as catalyst 2 eq of H_2O_2. **10b** was obtained following oxidation protocol A with 17 eq. of acetic acid (AcOH), 1 mol% of *(R,R)*-Mn(^{TIPS}pdp) and 2 eq. of H_2O_2. Purified by flash chromatography over silica using hexane: ethyl acetate 5:1 and the product was concentrated to dryness. The product was isolated as a yellow oil (43% yield, 7% ee). ^{1}H NMR (400 MHz, $CDCl_3$) δ 5.25 – 5.15 (m, 1H), 2.07 (s, 3H), 1.83 (dd, *J* = 15.0, 8.0 Hz, 1H), 1.69 (dd, *J* = 14.9, 3.4 Hz, 1H), 1.42 – 1.29 (m, 3H), 1.26 (s, 3H), 1.24 (s, 3H), 0.94 (dd, *J* = 6.5, 2.1 Hz, 6H). ^{13}C NMR (101 MHz, $CDCl_3$) δ 171.5, 70.5, 70.1, 48.1, 45.3, 30.2, 29.8, 23.1, 22.4, 21.7. HRMS (ESI+) *m/z* calculated for $C_{11}H_{22}O_3$ $[M+Na]^+$ 225.1461, found 225.1464. Chiral GC analysis were done using J&W CYCLOSIL-B.

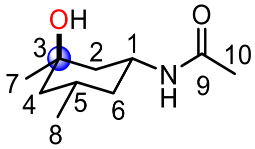

(1R,3R,5S)-11b: Purified by flash chromatography over silica using hexane:ethyl acetate 1:10 and the product was concentrated to dryness. The product was isolated as a white solid (53% yield, 97% ee). ^{1}H NMR (400 MHz, $CDCl_3$) δ 5.22 (s, 1H, *N[1H]*), 4.13 (dtt, *J* = 12.1, 8.0, 4.0 Hz, 1H, *C_1[1H]*), 2.09 – 1.82 (m, 6H, *C_2[1H] C_5[1H] C_6[1H] C_{10}[3H]*), 1.65 (ddt, *J* = 13.6, 4.4, 2.3 Hz, 1H, *C_4[1H]*), 1.50 (s, 1H, *O[H]*), 1.24 (s, 3H, *C_7[3H]*), 1.09 (t, *J* = 12.6 Hz, 1H, *C_2[1H]*), 0.96 – 0.86 (m, 4H, *C_4[1H] C_8[3H]*), 0.68 (q, *J* = 12.3 Hz, 1H, *C_6[1H]*). ^{13}C NMR (101 MHz, $CDCl_3$) δ 169.2 (*C_9*), 70.6 (*C_3*), 46.5 (*C_4*), 45.3 (*C_1*), 45.1 (*C_2*), 41.1 (*C_6*), 31.6 (*C_7*), 26.9 (*C_{10}*), 23.6 (*C_5*), 21.9 (*C_8*). HRMS (ESI+) *m/z* calculated for $C_{10}H_{19}NO_2$ $[M+Na]^+$ 208.1308, found 208.1311. Chiral GC analysis were done using J&W CYCLOSIL-B. $[\alpha]_D^{24}$ +23.093 ($CHCl_3$, c 0.050). X-ray quality crystals were obtained by slow evaporation of a solution of **11b** in CH_3Cl.

Solid state structure of ***(1R,3R,5S)-11b***

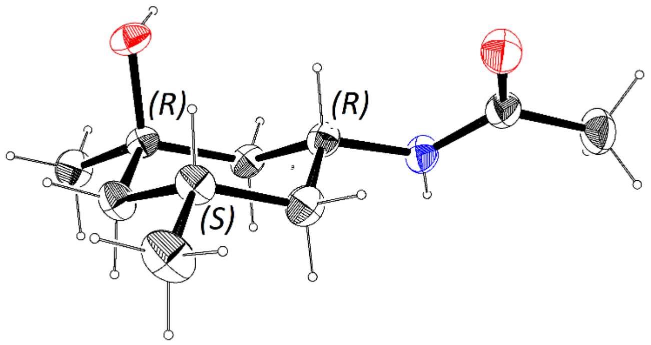

The determination of the absolute configuration was done having in consideration the methodology proposed by Escuderoet al.[19] Thus, a high quallity, high resolution, Full-sphere with phi and omega scans data set was acquired, with a high number of independent reflections, and a high coverage of the Bijvoet pairs.

Values of P2(right), P3, Hooft parameter y,[20] and Flack parameter using the Parsons method z,[21] were calculated with PLATON.[22]

P2(true)	1.000
P3(true)	1.000
P3(rac-twin)	0.4E-03
P3(false)	0.2E-10
Parsons z	-0.19(17)
Hooft y	-0.17(16)

Crystal data of *(+)*-***(1R,3R,5S)-11b*****.**

Chemical formula	$C_{10}H_{19}NO_2$	
Formula weight	185.26 g/mol	
Temperature	100(2) K	
Wavelength	0.71076 Å	
Crystal size	0.030 x 0.040 x 0.500 mm	
Crystal habit	colorless needle	
Crystal system	orthorhombic	
Space group	P 21 21 21	
Unit cell dimensions	a = 6.081(4) Å	α = 90°
	b = 8.771(6) Å	β = 90°
	c = 21.095(12) Å	γ = 90°
Volume	1125.1(13) $Å^3$	
Z	4	
Density (calculated)	1.094 g/cm^3	
Absorption coefficient	0.075 mm^{-1}	
F(000)	408	
Diffractometer	D8 QUEST ECO three-circle diffractometer	
Radiation source	Ceramic x-ray tube (Mo Kα, λ = 0.71076 Å)	
Theta range for data collection	3.49 to 34.44°	
Index ranges	-9<=h<=9, -13<=k<=13, -33<=l<=33	
Reflections collected	74348	
Independent reflections	4675 [R(int) = 0.0434]	
Coverage of independent reflections	98.5%	
Absorption correction	Multi-Scan	
Max. and min. transmission	0.9980 and 0.9630	
Structure solution technique	direct methods	
Structure solution program	SHELXT 2014/5 (Sheldrick, 2014)	
Refinement method	Full-matrix least-squares on F2	
Refinement program	SHELXL-2017/1 (Sheldrick, 2017)	
Function minimized	Σ w(Fo2 - Fc2)2	
Data / restraints / parameters	4675 / 0 / 194	
Goodness-of-fit on F2	1.054	
Δ/σmax	0.001	

Final R indices	4230 data; I>2σ(I)	R1 = 0.0323, wR2 = 0.0796
	all data	R1 = 0.0392, wR2 = 0.0824
Weighting scheme	$w=1/[\sigma^2(F_o^2)+(0.0465P)^2+0.0906P]$ where $P=(F_o^2+2F_c^2)/3$	
Flack x	-0.15(19)	
Parsons z	-0.19(17)	
Hooft y	-0.17(16)	
P2(true)	1.000	
P3(true)	1.000	
P3(rac-twin)	0.4E-03	
P3(false)	0.2E-10	
G	1.3358	
G (su)	0.3290	
Largest diff. peak and hole	0.304 and -0.168 eÅ^{-3}	
R.M.S. deviation from mean	0.035 eÅ^{-3}	

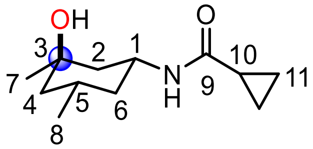

12b: Purified by flash chromatography over silica using hexane:ethyl acetate 1:1 and the product was concentrated to dryness. The product was isolated as a white solid (60 % yield, 83% ee). ^{1}H NMR (400 MHz, $CDCl_3$) δ 5.37 (s, 1H, *N[1H]*), 4.14 (tdt, *J* = 11.9, 7.8, 3.9 Hz, 1H, *C_1[1H]*), 1.97 (m, 2H, *C_2[1H] C_6[1H]*), 1.92 (dd, *J* = 8.7, 3.4 Hz, 1H, *C_5[1H]*), 1.67 (m, 1H, *C_4[1H]*), 1.47 (s, 1H, *O[H] C_6[1H]*), 1.30 – 1.25 (m, 1H, *C_{10}[1H]*), 1.23 (s, 3H, *C_7[3H]*), 1.13 (t, *J* = 12.6 Hz, 1H, *C_2[1H]*), 0.98 – 0.85 (m, 6H, *C_4[1H] C_8[3H] C_{11}[2H]*), 0.78 – 0.66 (m, 3H, *C_6[1H] C_{11}[2H]*) ^{13}C NMR (101 MHz, $CDCl_3$) δ 172.7 (*C_9*), 70.6 (*C_3*), 46.5 (*C_4*), 45.4 (*C_1*), 45.3 (*C_2*), 41.2 (*C_6*), 31.6 (*C_7*), 26.9 (*C_1*), 21.9 (*C_8*), 14.9 (*C_{10}*), 7.1 (*C_{11}*). HRMS (ESI+) *m/z* calculated for $C_{12}H_{21}NO_2$ $[M+Na]^+$ 234,1465, found 234,1473. Chiral GC analysis were done using HP-CHIRAL-20B. $[\alpha]_D^{24}$ +15.200 ($CHCl_3$, c 0.080).

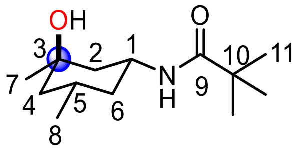

13b: Purified by flash chromatography over silica using hexane:ethyl acetate 1:1 and the product was concentrated to dryness. The product was isolated as a white solid (91% yield, 90% ee). ^{1}H NMR (400 MHz, $CDCl_3$) δ 5.37 (d, *J* = 7.9 Hz, 1H, *N[1H]*), 4.10 (tdt, *J* = 11.9, 7.8, 3.9 Hz, 1H, *C_1[1H]*), 2.02 (s, 1H, *O[H]*), 1.95 (ddq, *J* = 12.1, 5.1, 2.9 Hz, 3H, *C_2[1H] C_5[1H] C_6[1H]*), 1.66

(ddt, *J* = 13.6, 3.7, 2.2 Hz, 1H, $C_4[1H]$), 1.24 (s, 3H, $C_7[3H]$), 1.17 (s, 9H, $C_{11}[9H]$), 1.10 (dd, *J* = 13.1, 12.0 Hz, 1H, $C_2[1H]$), 0.95 – 0.86 (m, 4H, $C_4[1H]$ $C_8[3H]$), 0.70 (q, *J* = 12.5 Hz, 1H, $C_6[1H]$). ^{13}C NMR (101 MHz, $CDCl_3$) δ 177.8 (C_9), 70.6 (C_3) , 46.6 (C_4), 45.2 (C_1), 45.2 (C_6), 41.2 (C_2), 38.6 (C_{10}), 31.7 (C_7), 27.7 (C_{10}), 27.1 (C_5), 22.0 (C_8). HRMS (ESI+) *m/z* calculated for $C_{13}H_{25}NO_2$ $[M+Na]^+$ 250.1778, found 250.1783. Chiral GC analysis were done using J&W CYCLOSIL-B. $[\alpha]_D^{24}$ +11.267 ($CHCl_3$, c 0.060).

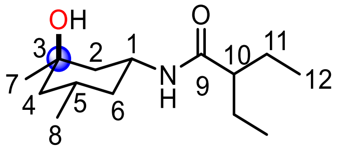

14b: Purified by flash chromatography over silica using hexane:ethyl acetate 1:1 and the product was concentrated to dryness. The product was isolated as a white solid (72% yield, 86% ee). ^{1}H NMR (400 MHz, $CDCl_3$) δ 5.22 (d, *J* = 8.2 Hz, 1H, *N[1H]*), 4.19 (tdt, *J* = 12.1, 8.1, 3.9 Hz, 1H, $C_1[1H]$), 2.04 – 1.88 (m, 3H, $C_5[1H]$, $C_2[1H]$, $C_6[1H]$), 1.76 (tt, *J* = 9.5, 5.0 Hz, 1H, $C_{10}[1H]$), 1.71 – 1.63 (m, 2H, $C_4[1H]$ *O[H]*), 1.59 (ddt, *J* = 13.5, 7.3, 1.5 Hz, 2H, $C_{11}[2H]$), 1.44 (dqd, *J* = 15.3, 7.4, 3.6 Hz, 2H, $C_{11}[2H]$), 1.24 (s, 3H, $C_7[3H]$), 1.12 (dd, *J* = 13.1, 12.0 Hz, 1H, $C_2[1H]$), 0.95 – 0.88 (m, 6H, $C_{12}[3H]$ $C_{12}[3H]$), 0.88 (d, *J* = 2.4 Hz, 3H, $C_8[3H]$), 0.86 (d, *J* = 2.4 Hz, 1H, $C_4[1H]$), 0.71 (q, *J* = 12.6 Hz, 1H, $C_6[1H]$). ^{13}C NMR (101 MHz, $CDCl_3$) δ 174.8 (C_9), 70.6 (C_3), 51.7 (C_{10}), 46.5 (C_4), 45.3 (C_2), 45.0 (C_1), 41.3 (C_6), 31.6 (C_7), 26.9 (C_5), 25.8 (C_{11}), 21.9 (C_{12}) , 12.1 (C_8). HRMS (ESI+) *m/z* calculated for $C_{14}H_{27}NO_2$ $[M+Na]^+$ 264.1934, found 264.1933. Chiral GC analysis were done using J&W CYCLOSIL-B. $[\alpha]_D^{24}$ +8.572 ($CHCl_3$, c 0.060).

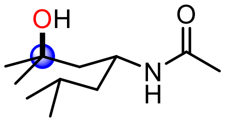

Oxidation of **15a** using method B was unsuccessful with the formation of traces amounts of the desired product. **15b** was obtained following oxidation protocol A with 17 eq. of acetic acid (AcOH), 1 mol% of *(R,R)*-Mn(^{TIPS}pdp) and 3 eq. of H_2O_2. Purified by flash chromatography over silica using hexane:ethyl acetate 1:1 and the product was concentrated to dryness. The product was isolated as a white solid (41% yield, 23% ee). ^{1}H NMR (400 MHz, $CDCl_3$) δ 5.68 (d, *J* = 8.5 Hz, 1H), 4.13 (qdd, *J* = 8.7, 5.4, 3.6 Hz, 1H), 3.33 (s, 1H), 1.99 (s, 3H), 1.65 – 1.61 (m, 1H), 1.55 (d, *J* = 14.6 Hz, 1H), 1.43 (ddd, *J* = 14.5, 9.1, 5.6 Hz, 1H), 1.30 – 1.25 (m, 5H), 1.22 (s, 3H), 0.93 (dd, *J* = 6.6, 2.9 Hz, 6H). ^{13}C NMR (101 MHz, $CDCl_3$) δ 170.7, 69.8, 49.5, 46.4, 44.9, 30.8, 29.5, 25.1, 23.6, 23.1, 22.2.HRMS (ESI+) *m/z* calculated for $C_{11}H_{23}NO_2$ $[M+Na]^+$ 208.1672, found 208.1671. **15b** was functionalized for Chiral SFC analysis (Section 1.8). Chiral SFC analysis were done using Chiralpack IC-3.

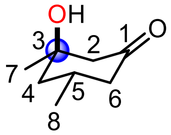

16b was obtained following oxidation protocol B using 5 mol% of the catalyst. Purified by flash chromatography over silica using hexane:ethyl acetate 2:1 and the product was concentrated to dryness. The product was isolated as a yellow oil (49% yield, 83% ee). ^{1}H NMR (400 MHz, $CDCl_3$) δ 2.44 – 2.35 (m, 3H, $C_2[2H]$ $C_6[1H]$), 2.35 – 2.23 (m, 1H, $C_5[1H]$), 1.99 – 1.82 (m, 2H, $C_4[1H]$ $C_6[1H]$), 1.45 (t, *J* = 13.8, 12.2 Hz, 1H, $C_4[1H]$), 1.37 (s, 3H, $C_7[3H]$), 1.33 (s, 1H, *O[H]*), 1.05 (d, *J* = 6.6 Hz, 3H, $C_8[3H]$). ^{13}C NMR (101 MHz, $CDCl_3$) δ 210.0 (C_1), 73.7 (C_3), 54.0 (C_2), 49.0 (C_6), 46.2 (C_4), 31.2 (C_7), 28.7 (C_5), 21.9 (C_8). HRMS (ESI+) *m/z* calculated for $C_8H_{14}O_2$ $[M+Na]^+$ 165.0891, found 165.0897. Chiral GC analysis were done using J&W CYCLOSIL-B. $[\alpha]_D^{24}$ +3.282 ($CHCl_3$, c 0.146).

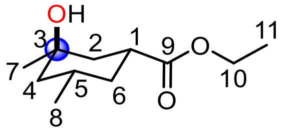

17b was obtained following oxidation protocol B using 2 mol% of the catalyst. Purified by flash chromatography over silica using hexane: ethyl acetate 5:1 and the product was concentrated to dryness. The product was isolated as a yellow oil (55% yield, 18% ee). ^{1}H NMR (400 MHz, $CDCl_3$) δ 4.12 (q, *J* = 7.1 Hz, 2H, $C_{10}[2H]$), 2.74 (tt, *J* = 12.6, 3.6 Hz, 1H, $C_1[1H]$), 1.95 (dtt, *J* = 12.8, 3.6, 1.9 Hz, 1H, $C_6[1H]$), 1.87 (m, 2H, $C_2[1H]$ $C_5[1H]$), 1.63 (ddt, *J* = 13.6, 4.0, 2.2 Hz, 1H, $C_4[1H]$), 1.59 (bs, 1H, *O[H]*), 1.46 – 1.38 (m, 1H, $C_2[1H]$), 1.28 – 1.22 (m, 6H, $C_7[3H]$ $C_{11}[3H]$), 1.04 – 0.95 (m, 2H, $C_4[1H]$ $C_6[1H]$), 0.92 (d, *J* = 6.6 Hz, 3H, $C_8[3H]$). ^{13}C NMR (101 MHz, $CDCl_3$) δ 176.1 (C_9), 70.0 (C_3), 60.2 (C_{10}), 46.8 (C_4), 40.6 (C_2), 39.2 (C_1), 36.7 (C_6), 31.7 (C_7), 27.3 (C_5), 22.0 (C_8), 14.2 (C_{11}). HRMS (ESI+) *m/z* calculated for $C_{11}H_{20}O_3$ $[M+Na]^+$ 223.1305, found 223.1311. Chiral GC analysis were done using J&W CYCLOSIL-B. $[\alpha]_D^{24}$ +0.979 ($CHCl_3$, c 0.205).

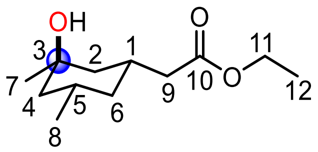

18b: Purified by flash chromatography over silica using hexane:ethyl acetate 2:1 and the product was concentrated to dryness. The product was isolated as a yellow oil (80% yield, 96% ee). ^{1}H NMR (400 MHz, $CDCl_3$) δ 4.13 (q, *J* = 7.1 Hz, 2H, $C_{11}[2H]$), 2.24 – 2.15 (m, 3H, $C_1[1H]$ $C_9[2H]$), 1.81 (m, 1H, $C_5[1H]$), 1.73 (ddq, *J* = 12.7, 3.4, 1.9 Hz, 1H, $C_6[1H]$), 1.70 – 1.60 (m, 2H, $C_2[1H]$ $C_4[1H]$), 1.26 (t, *J* = 7.1 Hz, 4H, *O[H]* $C_{12}[3H]$), 1.22 (s, 3H, $C_7[3H]$), 1.02 (ddt, *J* = 12.1, 8.7, 6.2

Hz, 1H, *C2[1H]*), 0.97 – 0.92 (m, 1H, *C4[1H]*), 0.89 (d, *J* = 6.6 Hz, 3H, *C8[3H]*), 0.56 (q, *J* = 12.1 Hz, 1H, *C6[1H]*). ^{13}C NMR (101 MHz, $CDCl_3$) δ 172.8 (*C10*), 70.3 (*C3*), 60.2 (*C11*), 47.1 (*C4*), 44.9 (*C2*), 41.7 (*C9*), 40.8 (*C6*), 31.6 (*C7*), 30.4 (*C1*), 27.6 (*C5*), 22.2 (*C8*), 14.3 (*C12*). HRMS (ESI+) *m/z* calculated for $C_{12}H_{22}O_3$ $[M+Na]^+$ 237.1461, found 237.1462. Chiral GC analysis were done using HP-CHIRAL-20B. $[\alpha]_D^{24}$ +3.102 ($CHCl_3$, c 0.160).

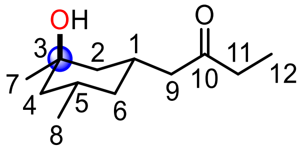

19b: Purified by flash chromatography over silica using hexane:ethyl acetate 5:1 and the product was concentrated to dryness. The product was isolated as a yellow oil (71% yield, 95% ee). ^{1}H NMR (400 MHz, $CDCl_3$) δ 2.41 (q, *J* = 7.3 Hz, 2H, *C11[2H]*), 2.33 – 2.17 (m, 3H, *C1[1H] C9[2H]*), 1.80 (tdq, *J* = 13.0, 6.6, 3.3 Hz, 1H, *C5[1H]*), 1.70 – 1.57 (m, 3H, *C2[1H] C4[1H] C6[1H]*), 1.27 – 1.24 (bs, 1H, *O[H]*), 1.21 (s, 3H, *C7[3H]*), 1.04 (t, *J* = 7.3 Hz, 3H, *C12[3H]*), 1.01 – 0.94 (m, 1H, *C4[1H]*), 0.94 – 0.90 (m, 1H, *C2[1H]*), 0.87 (d, *J* = 6.6 Hz, 3H, *C8[3H]*), 0.51 (q, *J* = 11.8 Hz, 1H, *C6[1H]*). ^{13}C NMR (101 MHz, $CDCl_3$) δ 211.2 (*C10*), 70.3 (*C3*), 49.7 (*C9*), 47.1 (*C2*), 45.0 (*C4*), 41.0 (*C6*), 36.4 (*C11*), 31.6 (*C8*), 29.5 (*C1*), 27.7 (*C5*), 22.2 (*C8*), 7.8 (*C12*). HRMS (ESI+) *m/z* calculated for $C_{12}H_{22}O_2$ $[M+Na]^+$ 221.1516, found 221.1519. **19b** was functionalized for Chiral SFC analysis (Section 1.8). Chiral SFC analysis were done using Chiralpack IB-3. $[\alpha]_D^{24}$ +3.708 ($CHCl_3$, c 0.087).

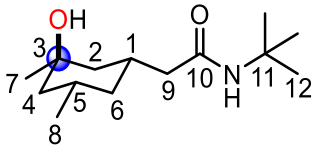

20b was obtained following oxidation protocol B using 2 mol% of the catalyst. Purified by flash chromatography over silica using hexane:ethyl acetate 1:1 and the product was concentrated to dryness. The product was isolated as a white solid (51% yield, >99% ee). ^{1}H NMR (400 MHz, $CDCl_3$) δ 5.21 (s, 1H, *N[1H]*), 2.25 – 2.069 (m, 1H, *C1[1H]*), 2.01 – 1.90 (m, 2H, *C9[2H]*), 1.80 (dd, *J* = 6.5, 3.4 Hz, 1H, *C5[1H]*), 1.73 (d, *J* = 13.5 Hz, 1H, *C6[1H]*), 1.70 – 1.62 (m, 2H, *C2[1H] C4[1H]*), 1.35 (s, 9H, *C12[3H] C12[3H] C12[3H]*), 1.25 (bs, 1H, *O[H]*), 1.21 (s, 3H, *C7[3H]*), 1.04 – 0.95 (m, 1H, *C2[1H]*), 0.91 – 0.86 (m, 4H, *C4[1H] C8[3H]*), 0.53 (q, *J* = 12.1 Hz, 1H, *C6[1H]*).^{13}C NMR (101 MHz, $CDCl_3$) δ 171.4 (*C10*), 70.3 (*C3*), 51.2 (*C11*), 47.1 (*C4*), 45.2 (*C9*), 45.0 (*C2*), 40.8 (*C6*), 31.6 (*C7*), 31.0 (*C1*), 28.9 (*C12*), 27.7 (*C5*), 22.2 (*C8*). HRMS (ESI+) *m/z* calculated for $C_{14}H_{27}NO_2$ $[M+Na]^+$ 264.1934, found 264.1932. Chiral SFC analysis were done using Chiralpack IG-3. $[\alpha]_D^{24}$ +5.890 ($CHCl_3$, c 0.127).

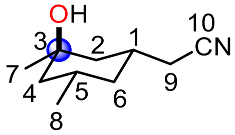

21b was obtained following oxidation protocol B using 2 mol% of the catalyst. Purified by flash chromatography over silica using hexane:ethyl acetate 5:1 and the product was concentrated to dryness. The product was isolated as a yellow oil (52% yield, 43% ee). ^{1}H NMR (400 MHz, $CDCl_3$) δ 2.27 (dd, *J* = 6.3, 1.3 Hz, 2H, *C_9[2H]*), 2.15 (m, 1H, *C_1[1H]*), 1.87 (ddt, *J* = 15.4, 8.6, 2.7 Hz, 1H, *C_5[1H]*), 1.82 – 1.78 (m, 1H, *C_6[1H]*), 1.74 (ddd, *J* = 11.8, 3.0, 1.6 Hz, 1H, *C_2[1H]*), 1.67 – 1.62 (m, 1H, *C_4[1H]*), 1.26 (m, 4H, *C_7[3H] O[H]*), 1.12 (dd, *J* = 13.2, 12.4 Hz, 1H, *C_2[1H]*), 1.03 – 0.94 (m, 1H, *C_4[1H]*), 0.92 (d, *J* = 6.5 Hz, 3H, *C_8[3H]*), 0.71 (q, *J* = 12.1 Hz, 1H, *C_6[1H]*). ^{13}C NMR (101 MHz, $CDCl_3$) δ 118.8 (*C_{10}*), 70.4 (*C_3*), 47.0 (*C_4*), 44.1 (*C_2*), 40.1 (*C_6*), 31.8 (*C_7*), 30.3 (*C_1*), 27.5 (*C_5*), 24.5 (*C_9*), 22.1 (*C_8*). HRMS (ESI+) *m/z* calculated for $C_{10}H_{17}NO$ $[M+Na]^+$ 190.1202, found 190.1209. **21b** was functionalized for Chiral SFC analysis (Section 1.8). Chiral SFC analysis were done using Chiralpack IA-3. $[\alpha]_D^{24}$ -1.705 ($CHCl_3$, c 0.220).

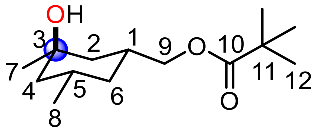

22b: was obtained following oxidation protocol B using 2 mol% of the catalyst. Purified by flash chromatography over silica using hexane: ethyl acetate 5:1 and the product was concentrated to dryness. The product was isolated as a yellow oil (62% yield, 39% ee). ^{1}H NMR (400 MHz, $CDCl_3$) δ 3.94 – 3.84 (m, 2H, *C_9[2H]*), 2.09 (ttt, *J* = 12.7, 6.2, 3.4 Hz, 1H, *C_1[1H]*), 1.81 (m, 1H, *C_5[1H]*), 1.72 (ddt, *J* = 12.5, 3.5, 1.7 Hz, 1H, *C_6[1H]*), 1.65 (m, 2H, *C_2[1H] C_4[1H]*), 1.25 (s, 3H, *C_7[3H]*), 1.21 (s, 9H, *C_{12}[3H] C_{12}[3H] C_{12}[3H]*), 1.11 (bs, 1H, *O[H]*), 1.08 – 0.93 (m, 2H, *C_2[1H] C_4[1H]*), 0.91 (d, *J* = 6.6 Hz, 3H, *C_8[3H]*), 0.59 (q, *J* = 12.2 Hz, 1H, *C_6[1H]*). ^{13}C NMR (101 MHz, $CDCl_3$) δ 178.6 (*C_{10}*), 70.2 (*C_3*), 69.1 (*C_9*), 47.4 (*C_4*), 41.5 (*C_2*), 38.9 (*C_{11}*), 37.5 (*C_6*), 33.0 (*C_1*), 31.8 (*C_7*), 27.4 (*C_5*), 27.3 (*C_{12}*), 22.3 (*C_8*). HRMS (ESI+) *m/z* calculated for $C_{14}H_{26}O_3$ $[M+Na]^+$ 265.1774, found 265.1777. Chiral GC analysis were done using J&W CYCLOSIL-B. $[\alpha]_D^{24}$ -0.725 ($CHCl_3$, c 0.173).

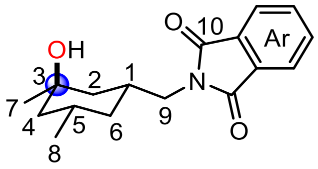

23b was obtained following oxidation protocol B using 2 mol% of the catalyst. Purified by flash chromatography over silica using hexane:ethyl acetate 1:1 and the product was concentrated to dryness. The product was isolated as a white solid (51% yield, 78% ee). ^{1}H NMR

(400 MHz, $CDCl_3$) δ 7.87 – 7.80 (m, 2H, *Ar*), 7.78 – 7.68 (m, 2H, *Ar*), 3.61 – 3.48 (m, 2H, *C_9[2H]*), 2.21 (ddq, *J* = 16.0, 12.3, 3.8 Hz, 1H, *C_1[1H]*), 1.85 – 1.73 (m, 1H, *C_5[1H]*), 1.69 (d, *J* = 13.0 Hz, 1H, *C_6[1H]*), 1.67 – 1.58 (m, 2H, *C_2[1H] C_4[1H]*), 1.21 (s, 3H, *C_7[3H]*), 1.13 – 1.02 (m, 2H, *C_2[1H] O[H]*), 0.96 (dd, *J* = 13.7, 12.2 Hz, 1H, *C_4[1H]*), 0.89 (d, *J* = 6.5 Hz, 3H, *C_8[3H]*), 0.62 (q, *J* = 12.2 Hz, 1H, *C_6[1H]*). ^{13}C NMR (101 MHz, $CDCl_3$) δ 168.6 (*C_{10}*), 133.9 (*Ar*), 132.1 (*Ar*), 123.3 (*Ar*), 70.2 (*C_3*), 47.2 (*C_4*), 43.7 (*C_9*), 42.7 (*C_2*), 38.6 (*C_6*), 33.1 (*C_1*), 31.7 (*C_7*), 27.4 (*C_5*), 22.2 (*C_8*).HRMS (ESI+) *m/z* calculated for $C_{17}H_{21}NO_3$ $[M+Na]^+$ 310.1414, found 310.1414. Chiral SFC analysis were done using Chiralpack IG-3. $[\alpha]_D^{24}$ +5.299 ($CHCl_3$, c 0.245).

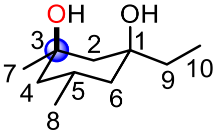

24b was obtained following oxidation protocol B using 2 mol% of the catalyst. Purified by flash chromatography over silica using hexane:ethyl acetate 2:1 and the product was concentrated to dryness. The product was isolated as a yellow oil (61% yield, 45% ee). ^1H NMR (400 MHz, $CDCl_3$) δ 3.32 (bs, 1H, *O[H]*), 2.93 (bs, 1H, *O[H]*), 2.20 – 2.05 (m, 1H, *C_5[1H]*), 1.71 (m, 3H, *C_2[1H] C_4[1H] C_6[1H]*), 1.46 (q, *J* = 7.7 Hz, 2H, *C_9[2H]*), 1.27 (d, *J* = 14.1 Hz, 1H, *C_2[1H]*), 1.21 (s, 3H, *C_7[3H]*), 1.00 – 0.88 (m, 8H, *C_4[1H] C_6[1H] C_8[3H] C_{10}[3H]*).^{13}C NMR (101 MHz, $CDCl_3$) δ 74.1 (*C_3*), 71.8 (*C_1*), 47.8 (*C_4*), 45.7 (*C_2*), 45.1 (*C_6*), 36.5 (*C_9*), 31.2 (*C_7*), 23.6 (*C_5*), 22.1 (*C_8*), 7.4 (*C_{10}*). HRMS (ESI+) *m/z* calculated for $C_{10}H_{20}O_2$ $[M+Na]^+$ 195.1356, found 195.1361. Chiral GC analysis were done using J&W CYCLOSIL-B. $[\alpha]_D^{24}$ +1.757 ($CHCl_3$, c 0.046).

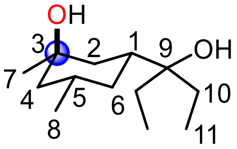

25b was obtained following oxidation protocol B using 2 mol% of the catalyst. Purified by flash chromatography over silica using hexane: ethyl acetate 2:1 and the product was concentrated to dryness. The product was isolated as a white solid (76% yield, 92% ee). ^1H NMR (400 MHz, $CDCl_3$) δ 1.85 (tt, *J* = 12.5, 3.1 Hz, 1H, *C_1[1H]*), 1.77 (dtd, *J* = 12.0, 6.2, 2.8 Hz, 1H, *C_5[1H]*), 1.73 – 1.67 (m, 1H, *C_6[1H]*), 1.67 – 1.59 (m, 2H, *C_2[1H] C_4[1H]*), 1.57 – 1.44 (m, 4H, *C_{10}[2H] C_{10}[2H]*), 1.25 (m, 4H, *C_7[3H] O[H]*), 1.15 (t, *J* = 13.0 Hz, 2H, *C_2[1H] O[H]*), 0.99 – 0.90 (m, 4H, *C_4[1H] C_8[3H]*), 0.86 (td, *J* = 7.5, 2.1 Hz, 6H, *C_{11}[3H] C_{11}[3H]*), 0.68 (q, *J* = 12.1 Hz, 1H, *C_6[1H]*).

^{13}C NMR (101 MHz, $CDCl_3$) δ 75.5 (*C_9*), 70.7 (*C_3*), 47.3 (*C_4*), 38.9 (*C_1*), 38.6 (*C_2*), 34.5 (*C_6*), 32.0 (*C_7*), 28.2 (*C_5*), 28.1 (*C_{10}*), 28.0 (*C_{10}*), 22.6 (*C_8*), 7.6 (*C_{11}*), 7.6 (*C_{11}*).HRMS (ESI+) *m/z* calculated for $C_{13}H_{26}O_2$

[M+Na]$^+$ 237.1825, found 237.1832. Chiral GC analysis were done using J&W CYCLOSIL-B. $[\alpha]_D^{24}$ +9.227 ($CHCl_3$, c 0.207).

Oxidation of 26a

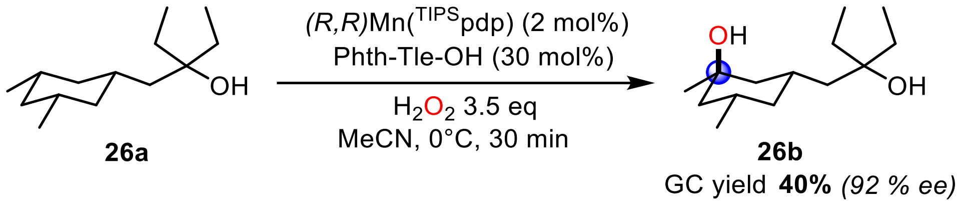

The yield of **26b** was determined by GC analysis, using biphenyl as internal standard. **26a** was oxidized following oxidation protocol B with 30 mol% of the aminoacid. (Phth-Tle-OH) using 2 mol% of *(R,R)*-Mn(^{TIPS}pdp) as catalyst at 0ºC.

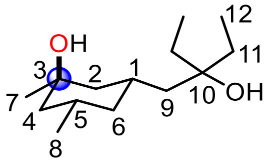

26b was obtained following oxidation protocol A with 17 eq. of cyclopropane carboxylic acid (cpcOH), 1 mol% of *(R,R)*-Mn(^{TIPS}pdp) and 3.5 eq of H_2O_2,. Purified by flash chromatography over silica using hexane: ethyl acetate 2:1 and the product was concentrated to dryness. The product was isolated as a white solid (70% yield, 74% ee). ^{1}H NMR (400 MHz, $CDCl_3$) δ 1.87 (ddt, *J* = 12.2, 10.1, 6.2 Hz, 1H, *C_1[1H]*), 1.78 (ddt, *J* = 14.9, 5.3, 2.5 Hz, 3H, *C_4[1H] C_5[1H] C_6[1H]*), 1.61 (ddd, *J* = 9.6, 4.2, 1.9 Hz, 1H, *C_2[1H]*), 1.50 (tt, *J* = 6.8, 4.0 Hz, 4H, *C_{11}[2H] C_{11}[2H]*), 1.31 (dd, *J* = 6.8, 5.5 Hz, 2H, *C_9[2H]*, 1.29 – 1.24 (bs, 1H, *O[H]*), 1.20 (s, 3H, *C_7[3H]*), 0.98 (s, *O[H]*), 0.95 – 0.89 (m, 2H, *C_2[1H] C_4[1H]*), 0.89 – 0.84 (m, 9H, *C_8[1H] C_{12}[3H] C_{12}[13H]*), 0.63 – 0.50 (m, 1H, *C_6[1H]*). ^{13}C NMR (101 MHz, $CDCl_3$) δ 75.5 (*C_{10}*), 70.7 (*C_3*), 47.2 (*C_2*), 47. 1 (*C_4*) 45.4 (*C_9*), 43.3 (*C_6*), 31.7 (*C_7*), 31.5 (*C_{11}*), 31.3 (*C_{11}*), 28.4 (*C_1*), 28.0 (*C_5*), 22.3 (*C_8*), 8.0 (*C_{12}*), 8.0 (*C_{12}*). HRMS (ESI+) *m/z* calculated for $C_{14}H_{28}O_2$ [M+Na]$^+$ 251.1987, found 251.1992. Chiral GC analysis were done using J&W CYCLOSIL-B. $[\alpha]_D^{24}$ +5.460 ($CHCl_3$, c 0.067).

Oxidation of 27a

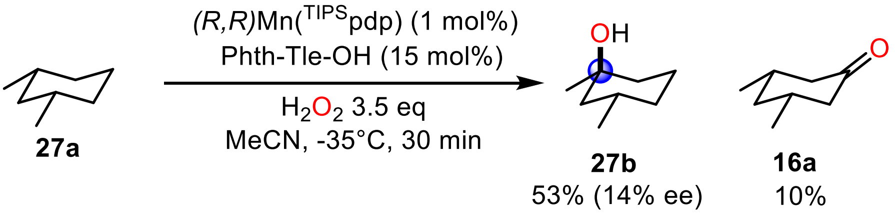

27a **27b** 53% (14% ee) **16a** 10%

The yields of **27b** and **16a** were determined by GC analysis, using biphenyl as internal standard.

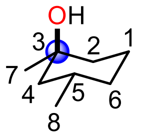

27b: Purified by flash chromatography over silica using hexane:ethyl acetate 5:1 and the product was concentrated to dryness. The product was isolated as a yellow oil (8% yield, 14% ee). ^{1}H NMR (400 MHz, $CDCl_3$) δ 1.80 – 1.63 (m, 2H, $C_5[1H]$ $C_6[1H]$), 1.63 – 1.50 (m, 4H $C_1[2H]$ $C_2[1H]$ $C_4[1H]$), 1.34 – 1.28 (bs, 1H, *O[H]*), 1.24 (m, 1H, $C_2[1H]$), 1.20 (s, 3H, $C_7[1H]$), 0.97 (dd, *J* = 13.5, 12.0 Hz, 1H, $C_4[1H]$), 0.88 (d, *J* = 6.2 Hz, 3H, $C_8[3H]$), 0.85 – 0.74 (m, 1H, $C_6[1H]$). ^{13}C NMR (101 MHz, $CDCl_3$) δ 70.1 (C_3), 47.6 (C_4), 38.3 (C_2), 34.4 (C_6), 31.7 (C_7), 28.0 (C_5), 22.5 (C_8), 21.8 (C_1). ^{13}C NMR Spectral data match those previously reported.[23] Chiral GC analysis were done using J&W CYCLOSIL-B.

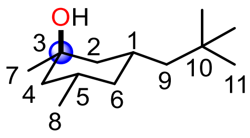

28b was obtained following oxidation protocol B using 2 mol% of the catalyst. Purified by flash chromatography over silica using pentane: diethyl ether 5:1 and the product was concentrated to dryness. The product was isolated as a yellow oil (48% yield, 80% ee). ^{1}H NMR (400 MHz, $CDCl_3$) δ 1.81 – 1.66 (m, 4H, $C_1[1H]$ $C_2[1H]$ $C_5[1H]$ $C_6[1H]$), 1.62 (dt, *J* = 8.7, 2.7 Hz, 1H, $C_4[1H]$), 1.58 (s, 1H, *O[H]*), 1.19 (s, 3H, $C_3[3H]$), 1.08 (dd, *J* = 5.8, 4.6 Hz, 2H, $C_9[2H]$), 0.98 – 0.94 (m, 1H, $C_2[1H]$), 0.90 (m, 10H, $C_4[1H]C_{11}[9H]$), 0.87 (d, *J* = 6.5 Hz, 3H, $C_8[3H]$), 0.57 – 0.46 (m, 1H, $C_6[1H]$). ^{13}C NMR (101 MHz, $CDCl_3$) δ 70.8 (C_3), 51.2 (C_9), 47.9 (C_2), 47.1 (C_4), 43.9 (C_6), 31.7 (C_7), 31.1 (C_{10}), 30.1 (C_9), 29.4 (C_5), 28.1 (C_1), 22.4 (C_8). HRMS (ESI+) *m/z* calculated for $C_{13}H_{26}O$ $[M+Na]^+$ 221.1876, found 221.1520. Chiral GC analysis were done using J&W CYCLOSIL-B. $[\alpha]_D^{24}$ +3.032 ($CHCl_3$, c 0.187).

Oxidation of 29a

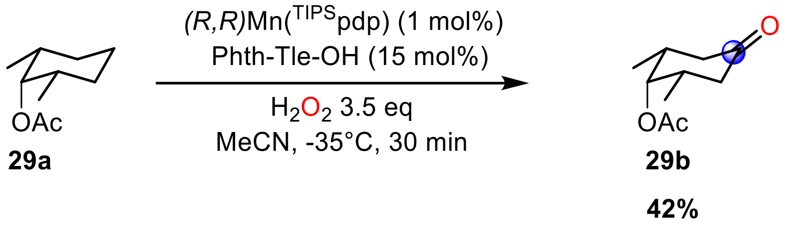

The yields of **29b** was determined by GC analysis, using biphenyl as internal standard.

29b was obtained following oxidation protocol A with 17 eq. of Acetic acid (AcOH) using (*S,S*)-Mn(pdp) as catalyst and purified by flash chromatography over silica using hexane: ethyl acetate 5:1 and the products were concentrated to dryness.

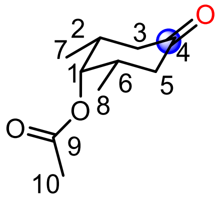

29b

^{1}H NMR (400 MHz, $CDCl_3$) δ 5.19 (d, *J* = 2.5 Hz, 1H, *C₁[1H]*), 2.33 (t, *J* = 13.9 Hz, 2H, *C₃[1H] C₅[1H]*), 2.25 – 2.14 (m, 5H, *C₃[1H] C₅[1H] C₁₀[3H]*), 2.10 (ddt, *J* = 11.0, 9.0, 4.6 Hz, 2H, *C₂[1H] C₆[1H]*), 0.97 (d, *J* = 6.7 Hz, 6H, *C₇[1H] C₈[1H]*). ^{13}C NMR (101 MHz, $CDCl_3$) δ 210.2 (*C₄*), 170.8 (*C₉*), 74.3 (*C₁*), 43.9 (*C₃*) (*C₅*), 36.4 (*C₂*) (*C₆*), 20.8 (*C₁₀*), 18.0 (*C₇*) (*C₈*). HRMS (ESI+) *m/z* calculated for $C_{10}H_{16}O_3$ $[M+Na]^+$ 207.0997, found 207.1001.

Oxidation of 30a

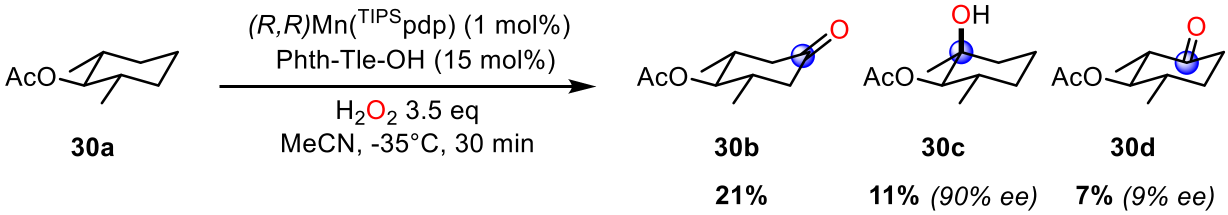

The yields of **30b**, **30c** and **30d** were determined by GC analysis, using biphenyl as internal standard. Chiral GC analysis were done using HP-CHIRAL-20B. **30b**, **30c** and **30d** were obtained following oxidation protocol A with 17 eq. of Acetic acid (AcOH) using (*S,S*)-Mn(pdp) as catalyst and purified by flash chromatography over silica using hexane: ethyl acetate 5:1 and the products were concentrated to dryness.

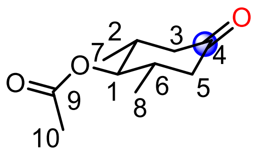

30b

^{1}H NMR (400 MHz, $CDCl_3$) δ 4.76 (t, *J* = 10.3 Hz, 1H, *C_1[1H]*), 2.48 – 2.37 (m, 2H, *C_3[1H] C_5[1H]*), 2.22 (t, *J* = 14.0 Hz, 2H, *C_3[1H] C_5[1H]*), 2.13 (s, 3H, *C_{10}[3H]*), 2.00 (m, 2H, *C_2[1H] C_6[1H]*), 0.98 (d, *J* = 6.4 Hz, 6H, *C_7[3H] C_8[3H]*). ^{13}C NMR (101 MHz, $CDCl_3$) δ 208.2 (*C_4*), 170.9 (*C_9*), 79.7 (*C_1*), 47.1 (*C_3*) (*C_5*), 37.0 (*C_2*) (*C_6*), 20.9 (*C_{10}*), 18.4 (*C_7*) (*C_8*). HRMS (ESI+) *m/z* calculated for $C_{10}H_{16}O_3$ $[M+Na]^+$ 207.0997, found 207.0994.

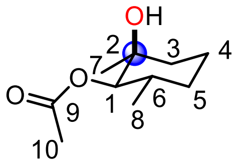

30c

^{1}H NMR (400 MHz, $CDCl_3$) δ 4.52 (d, *J* = 10.8 Hz, 1H, *C_1[1H]*), 2.14 (s, 3H, *C_{10}[3H]*), 2.01 – 1.86 (m, 1H, *C_6[1H]*), 1.83 – 1.78 (m, 1H, *C_3[1H]*), 1.73 (ddt, *J* = 13.1, 7.2, 3.6 Hz, 1H, *C_5[1H]*), 1.65 (dt, *J* = 13.3, 3.7 Hz, 1H, *C_4[1H]*), 1.46 (ddd, *J* = 10.0, 6.6, 3.1 Hz, 1H, *C_4[1H]*), 1.39 (td, *J* = 13.4, 4.3 Hz, 1H, *C_3[1H]*), 1.26 (s, 1H, *O[H]*), 1.12 (s, 3H, *C_7[1H]*), 1.09 – 1.01 (m, 1H, *C_5[1H]*), 0.85 (d, *J* = 6.6 Hz, 3H, *C_8[3H]*). ^{13}C NMR (101 MHz, $CDCl_3$) δ 170.7 (*C_9*), 82.6 (*C_1*), 71.3 (*C_2*), 37.9 (*C_3*), 33.4 (*C_5*), 32.5 (*C_6*), 27.7 (*C_7*), 20.9 (*C_{10}*), 20.5 (*C_4*), 18.2 (*C_8*). HRMS (ESI+) *m/z* calculated for $C_{10}H_{18}O_3$ $[M+Na]^+$ 209.1148, found 209.1151.

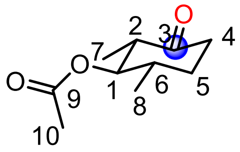

30d

^{1}H NMR (400 MHz, $CDCl_3$) δ 4.55 (dd, *J* = 11.0, 10.0 Hz, 1H, *C_1[1H]*), 2.56 – 2.47 (m, 1H, *C_2[1H]*), 2.47 – 2.34 (m, 2H, *C_4[2H]*), 2.12 (s, 3H, *C_{10}[3H]*), 2.03 – 1.94 (m, 2H, *C_5[1H] C_6[1H]*), 1.39 – 1.32 (m, 1H, *C_5[1H]*), 1.01 (d, *J* = 6.5 Hz, 3H, *C_7[3H]*), 0.98 (d, *J* = 6.3 Hz, 3H, *C_8[3H]*). ^{13}C NMR (101 MHz, $CDCl_3$) δ 208.7 (*C_3*), 170.4 (*C_9*), 80.3 (*C_1*), 50.5 (*C_2*), 40.6 (*C_4*), 37.2 (*C_6*), 29.3 (*C_5*), 20.9 (*C_{10}*), 17.7 (*C_8*), 10.6 (*C_7*). HRMS (ESI+) *m/z* calculated for $C_{10}H_{16}O_3$ $[M+Na]^+$ 207.0997, found 207.0999.

Oxidation of 31a

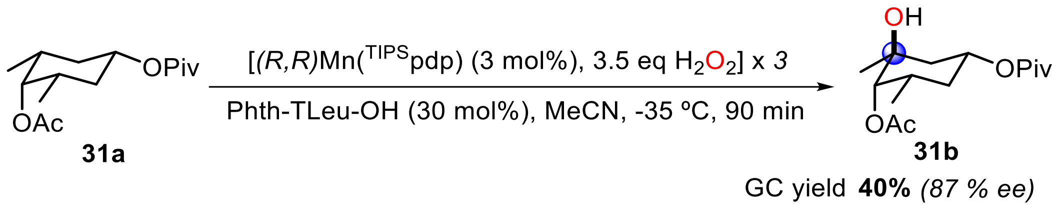

The yield of **31b** was determined by GC analysis, using biphenyl as internal standard. **27a** was oxidized following oxidation protocol B with 30 mol% of the aminoacid. (Phth-Tle-OH) using 3 mol% of *(R,R)*-Mn(^{TIPS}pdp) as catalyst. The catalyst were added twice to the reaction mixture and re-exposed two times to 3.5 equivalent of H_2O_2.

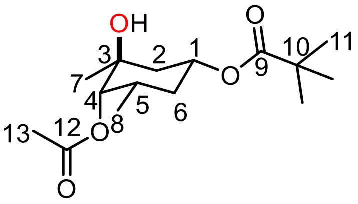

31b was obtained following oxidation protocol A with 17 eq. of acetic acid (AcOH), 3 mol% of *(R,R)*-Mn(^{TIPS}pdp) and 3.5 eq of H_2O_2, after 30 min to the reaction mixture were added other 3 mol% of the catalyst and re-exposed to 3.5 equivalent of H_2O_2. Purified by flash chromatography over silica using hexane: ethyl acetate 2:1 and the product was concentrated to dryness. The product was isolated as a white solid (54% yield, 73% ee). ^{1}H NMR (400 MHz, $CDCl_3$) δ 5.03 (tt, *J* = 11.6, 4.6 Hz, 1H, *C_1[1H]*), 4.77 (d, *J* = 2.5 Hz, 1H, *C_4[1H]*), 2.36 – 2.24 (m, 1H, *C_5[1H]*), 2.12 (s, 3H, *C_{13}[3H]*), 1.90 – 1.82 (m, 1H, *C_2[1H]*), 1.74 (d, *J* = 12.5 Hz, 1H, *C_6[1H]*), 1.60 (d, *J* = 11.6 Hz, 1H, *C_2[1H]*), 1.41 (bs, 1H, *O[H]*), 1.35 – 1.27 (m, 1H, *C_6[1H]*), 1.18 (s, 12H, *C_7[3H] C_{11}[9H]*), 0.88 (d, *J* = 6.9 Hz, 3H, *C_8[3H]*). ^{13}C NMR (101 MHz, $CDCl_3$) δ 178.0 (*C_9*), 170.4 (*C_{12}*), 76.5 (*C_4*), 72.7 (*C_3*), 69.3 (*C_1*), 39.1 (*C_2*), 38.6 (*C_{10}*), 33.2 (*C_3*), 29.3 (*C_5*), 27.7 (*C_7*), 27.1 (*C_{11}*), 20.9 (*C_{13}*), 17.2 (*C_8*). HRMS (ESI+) *m/z* calculated for $C_{15}H_{26}O_5$ $[M+Na]^+$ 309.1672, found 309.1674. Chiral GC analysis were done using HP-CHIRAL-20B. $[\alpha]_D^{24}$ +10.730 ($CHCl_3$, c 0.210).

1.6 Elaboration of tertiary alcohol

Synthesis of 6c

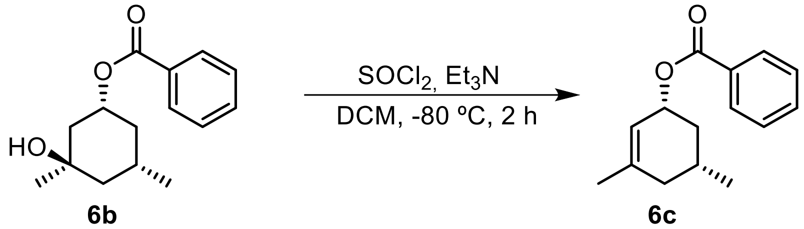

A round-bottom flask equipped with a septum and kept under nitrogen was charged with a 0.10 M solution of the alcohol **6b** (106 mg, 0.4 mmol, 1 equiv) in dry dichloromethane and triethylamine (550 µl, 4 mmol, 10 equiv), then the reaction mixture was cooled to -80 °C. The thionyl chloride (100 µl, 1.2 mmol, 3 equiv) was added dropwise over 10 minutes and the reaction was left stirring 10 min. At this point, HCl 1M solution was added until pH~3 and then diluted with dichloromethane. The aqueous layer was extracted with dichloromethane (3x) and the organic layers were combined. The organic layer was washed with saturated $NaHCO_3$ and dried over anhydrous sodium sulfate (Na_2SO_4), filtered and evaporated to dryness, then the crude was purified by flash chromatography over silica using hexane:ethyl acetate 5:1 to obtain **6c** (71 mg, 77% yield, 90% ee) as a yellow oil. ^{1}H NMR (400 MHz, $CDCl_3$) δ 8.09 – 8.01 (m, 2H), 7.60 – 7.52 (m, 1H), 7.48 – 7.38 (m, 2H), 5.28 – 5.16 (m, 2H), 2.49 – 2.34 (m, 2H), 2.23 – 2.08 (m, 2H), 1.70 (s, 3H), 1.33 (q, *J* = 11.6 Hz, 1H), 1.03 (d, *J* = 7.1 Hz, 3H). ^{13}C NMR (101 MHz, $CDCl_3$) δ 166.2, 132.8, 130.8, 130.5, 129.6, 128.3, 127.5, 71.6, 36.9, 35.8, 30.4, 23.2, 21.8. HRMS (ESI+) *m/z* calculated for $C_{15}H_{18}O_2$ $[M+Na]^+$ 253.1199, found 253.1196. Chiral GC analysis were done using J&W CYCLOSIL-B. $[\alpha]_D^{24}$ +2.044 ($CHCl_3$, c 0.120).

Synthesis of 6d

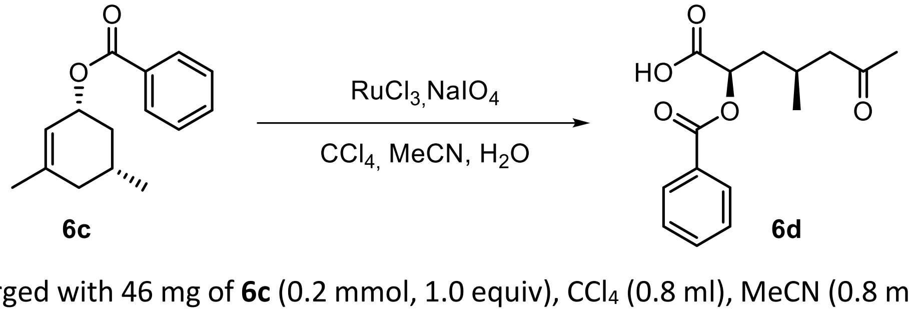

A vial was charged with 46 mg of **6c** (0.2 mmol, 1.0 equiv), CCl_4 (0.8 ml), MeCN (0.8 ml) and H_2O (1.2 ml) followed by the addition of $RuCl_3$ (2 mg, 0.01 mmol, 0.05 equiv). The sodium periodate (170 mg, 0.8 mmol, 4 equiv) was added portion wise over 5 minutes and the reaction was left stirring 4 h at

room temperature. At this point, HCl 1M solution was added until pH~1 and then diluted with dichloromethane. The aqueous layer was extracted with dichloromethane (3x), the organic layers were combined and dried over anhydrous sodium sulfate (Na_2SO_4), filtered and evaporated to dryness. Then the crude was purified by flash chromatography over silica using hexane:ethyl acetate 1:1 to obtain **6d** (48 mg, 82% yield) as a brown oil. ^1H NMR (400 MHz, $CDCl_3$) δ 8.04 – 7.91 (m, 2H), 7.59 – 7.50 (m, 1H), 7.46 – 7.39 (m, 2H), 5.56 (dtd, *J* = 8.5, 6.3, 3.7 Hz, 1H), 2.94 (dd, *J* = 16.2, 6.5 Hz, 1H), 2.75 (dd, *J* = 16.2, 6.1 Hz, 1H), 2.63 (dqd, *J* = 9.2, 7.1, 4.4 Hz, 1H), 2.19 (s, 3H), 2.18 – 2.09 (m, 1H), 1.88 (ddd, *J* = 14.5, 8.6, 4.4 Hz, 1H), 1.23 (d, *J* = 7.1 Hz, 3H). ^{13}C NMR (101 MHz, $CDCl_3$) δ 205.4, 181.0, 166.1, 133.3, 129.8, 128.5, 69.4, 48.2, 37.8, 36.0, 30.6, 17.8. HRMS (ESI+) *m/z* calculated for $C_{15}H_{18}O_5$ $[M+Na]^+$ 301.1046, found 301.1042. $[\alpha]_D^{24}$ +24.760 ($CHCl_3$, c 0.100).

Synthesis of 6e

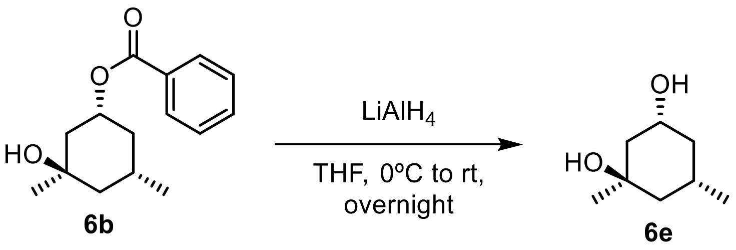

A round-bottom flask equipped with a septum and kept under nitrogen, was cooled to 0º C, then was charged with lithium aluminium hydride (17 mg, 0.45 mmol, 1.1 equiv) and 5 ml of dry diethyl ether. At this point, **6b** (50 mg, 0.2 mmol, 1 equiv) was added slowly as a solution in 1 ml of dry diethyl ether. The reaction was left stirring overnight, letting to warm up to room temperature. After this, the reaction mixture was quenched with the minimum quantity of 2M NaOH solution at 5°C. After filtration through Celite©, the organic fractions were dried over anhydrous Na_2CO_3, filtered and evaporated to dryness to obtain **6e** (19 mg, 65 % yield) as a white solid without further purification. ^1H NMR (400 MHz, $CDCl_3$) δ 3.97 (tt, *J* = 11.2, 4.4 Hz, 1H), 1.97 (dtd, *J* = 15.1, 4.7, 2.6 Hz, 2H), 1.85 (dddd, *J* = 15.4, 13.0, 6.7, 3.4 Hz, 1H), 1.58 (ddt, *J* = 13.7, 4.0, 2.1 Hz, 2H), 1.29 – 1.17 (m, 5H), 1.05 – 0.91 (m, 4H), 0.84 (q, *J* = 11.8 Hz, 1H). ^{13}C NMR (101 MHz, $CDCl_3$) δ 71.7, 67.5, 47.4, 46.8, 43.9, 31.7, 26.5, 21.9. HRMS (ESI+) *m/z* calculated for $C_8H_{16}O_2$, $[M+Na]^+$ 167.1043, found 167.1039. Chiral GC analysis were done using J&W CYCLOSIL-B. $[\alpha]_D^{24}$ +3.810 ($CHCl_3$, c 0.035).

Synthesis of 16c

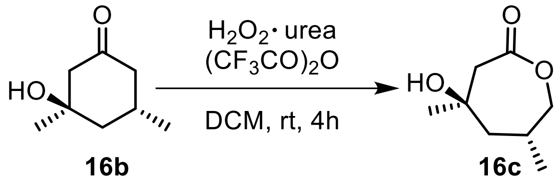

A round-bottom flask equipped with a septum and kept under nitrogen was charged with a 0.10 M solution of the alcohol **16b** (80 mg, 0.6 mmol, 1 equiv) in dry dichloromethane and hydrogen peroxide • urea (560 mg, 6 mmol, 10 equiv). Trifluoroacetic anhydride (350 μl, 2.5 mmol, 4.2 equiv) was added slowly and the reaction was left stirring 1 h at room temperature. At this point, the reaction was quenched with saturated $NaHCO_3$ and then the aqueous layer was extracted with dichloromethane (3x). The organic layers were combined and dried over anhydrous sodium sulfate (Na_2SO_4), filtered and evaporated to dryness. Then the crude was purified by flash chromatography over silica using hexane: ethyl acetate 1:1 to obtain **16c** (38 mg, 40% yield) as a white solid.

^1H NMR (400 MHz, $CDCl_3$) δ 4.08 (dt, *J* = 12.7, 2.0 Hz, 1H), 3.99 (dd, *J* = 12.6, 9.3 Hz, 1H), 3.00 (d, *J* = 13.8 Hz, 1H), 2.76 (dd, *J* = 13.8, 2.3 Hz, 1H), 2.42 – 2.27 (m, 1H), 2.17 (s, 1H), 2.00 – 1.93 (m, 1H), 1.46 – 1.32 (m, 4H), 0.95 (d, *J* = 7.1 Hz, 3H). ^{13}C NMR (101 MHz, $CDCl_3$) δ 206.9, 172.1, 74.2, 68.9, 50.5, 47.2, 32.9, 30.9, 29.6, 18.5. HRMS (ESI+) *m/z* calculated for $C_8H_{14}O_3$, $[M+Na]^+$ 159.1016, found 159.1019. Chiral GC analysis were done using J&W CYCLOSIL-B. $[\alpha]_D^{24}$ +5.2622 ($CHCl_3$, c 0.107). X-ray quality crystals were obtained by slow evaporation of a solution of **16c** in MeCN.

*Solid state structure of **16c***

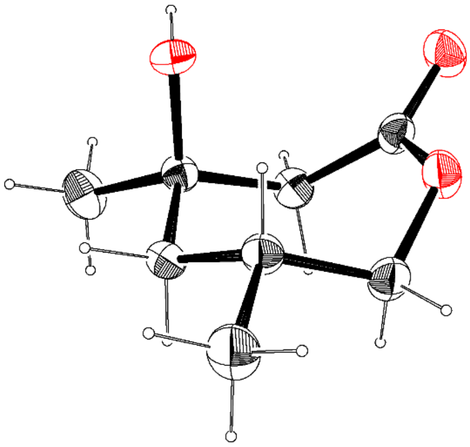

*Crystal data of **16c.***

Chemical formula	$C_8H_{14}O_3$	
Formula weight	158.19 g/mol	
Temperature	100(2) K	
Wavelength	0.71076 Å	
Crystal size	0.080 x 0.370 x 0.380 mm	
Crystal habit	colorless plate	
Crystal system	orthorhombic	
Space group	P b c a	
Unit cell dimensions	a = 10.205(3) Å	α = 90°
	b = 9.516(3) Å	β = 90°
	c = 17.035(4) Å	γ = 90°
Volume	1654.3(8) $Å^3$	
Z	8	
Density (calculated)	1.270 g/cm^3	
Absorption coefficient	0.096 mm^{-1}	
F(000)	688	
Diffractometer	D8 QUEST ECO three-circle diffractometer	
Radiation source	Ceramic x-ray tube (Mo Kα, λ = 0.71076 Å)	
Theta range for data collection	4.53 to 33.16°	
Index ranges	-15<=h<=15, -14<=k<=14, -26<=l<=26	
Reflections collected	98671	
Independent reflections	3142 [R(int) = 0.0202]	
Coverage of independent reflections	99.4%	
Absorption correction	Multi-Scan	
Max. and min. transmission	0.9920 and 0.9640	
Structure solution technique	direct methods	
Structure solution program	SHELXT 2014/5 (Sheldrick, 2014)	

Refinement method	Full-matrix least-squares on F2
Refinement program	SHELXL-2017/1 (Sheldrick, 2017)
Function minimized	Σ w(Fo2 - Fc2)2
Data / restraints / parameters	3142 / 0 / 104
Goodness-of-fit on F2	1.086
Δ/σmax	0.001
Final R indices	2983 data; I>2σ(I) R1 = 0.0326, wR2 = 0.0892
	all data R1 = 0.0342, wR2 = 0.0908
Weighting scheme	$w=1/[\sigma^2(F_o^2)+(0.0469P)^2+0.3730P]$ where $P=(F_o^2+2F_c^2)/3$
Largest diff. peak and hole	0.482 and -0.219 eÅ^{-3}
R.M.S. deviation from mean	0.043 eÅ^{-3}

Synthesis of 16d

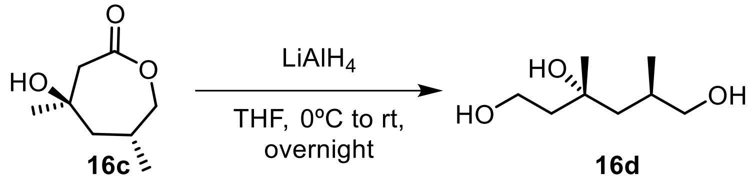

A round-bottom flask equipped with a septum and kept under nitrogen, was cooled to 0º C, then was charged with lithium aluminium hydride (20 mg, 0.5 mmol, 2.1 equiv) and 5 ml of dry THF. At this point, **16c** (40 mg, 0.25 mmol, 1 equiv) was added slowly as a solid under nitrogen flow. The reaction was left stirring overnight, letting to warm up to room temperature. After this, the reaction mixture was quenched with the minimum quantity of 2M NaOH solution at 5°C. After filtration through Celite©, the organic fractions were dried over anhydrous Na_2CO_3, filtered and evaporated to dryness to obtain **16d** (32 mg, 78 % yield) as a yellow oil without further purification.

^{1}H NMR (400 MHz, $CDCl_3$) δ 4.50 (bs, 1H), 3.99 – 3.88 (m, 2H), 3.65 (dd, *J* = 10.5, 3.5 Hz, 1H), 3.34 (dd, *J* = 10.5, 9.0 Hz, 1H), 2.97 (s, 1H), 2.15 – 2.01 (m, 1H), 1.82 – 1.67 (m, 4H), 1.45 (dd, *J* = 14.5, 2.8 Hz, 1H), 1.30 (s, 3H), 0.90 (d, *J* = 7.0 Hz, 3H). ^{13}C NMR (101 MHz, $CDCl_3$) δ 73.2, 69.3, 59.9, 49.1, 43.8, 31.5, 25.9, 19.6. HRMS (ESI+) *m/z* calculated for $C_8H_{18}O_3$, $[M+Na]^+$ 185.1148, found 185.1153.

$[\alpha]_D^{24}$ +27.900 ($CHCl_3$, c 0.207).

Synthesis of 16e

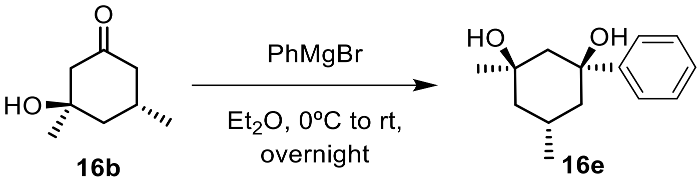

To a round-bottom flask equipped with a septum and kept under nitrogen charged with **16b** (75 mg, 0.5 mmol, 1 equiv) in diethyl ether (0.75 M) was added dropwisely 0.6 ml of phenylmagnesium bromide solution 1.0 M in THF (0.6 mmol, 1.2 equiv) at 0 ºC. The reaction was left stirring overnight, letting to warm up to room temperature. At this point, the reaction mixture was cooled down to 0 ºC, quenched with $NH_4Cl_{(aq,\ sat)}$ and washed with Et_2O (3x). The combined organic layers was washed with H_2O and $NaCl_{(aq,\ sat)}$ and dried over anhydrous sodium sulfate (Na_2SO_4), filtered and evaporated to dryness, then the crude alcohol was purified by flash chromatography over silica using hexane:ethyl acetate 1 :1 to obtain **16e** (52 mg, 47% yield) as a white solid ^{1}H NMR (400 MHz, CD_2Cl_2) δ 7.51 – 7.44 (m, 2H), 7.33 (dd, *J* = 8.5, 6.9 Hz, 2H), 7.26 – 7.19 (m, 1H), 2.65 (bs, 2H, water), 2.26 (tdp, *J* = 13.0, 6.6, 3.2 Hz, 1H), 1.88 – 1.67 (m, 4H), 1.45 (dd, *J* = 13.5, 12.2 Hz, 1H), 1.24 (s, 3H), 1.14 (dd, *J* = 13.5, 12.4 Hz, 1H), 1.00 (d, *J* = 6.6 Hz, 3H). ^{13}C NMR (101 MHz, CD_2Cl_2) δ 149.1, 128.5, 127.0, 124.8, 75.5, 72.4, 48.6, 47.7, 47.6, 31.6, 24.3, 22.1. HRMS (ESI+) *m/z* calculated for $C_{14}H_{20}O_2$ $[M+Na]^+$ 243.1361, found 243.1364. $[\alpha]_D^{24}$ +7.400 ($CHCl_3$, c 0.186).

1.7 NMR Spectra

^{1}H-NMR of **2a** in $CDCl_3$

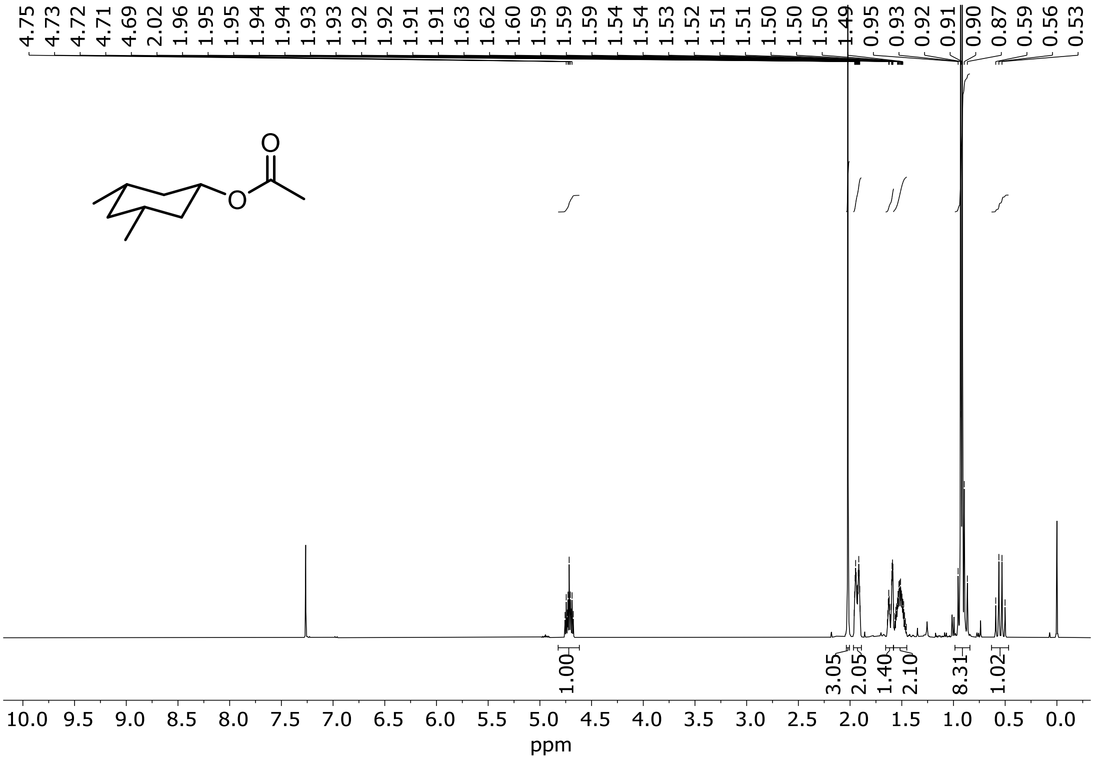

^{13}C-NMR of **2a** in $CDCl_3$

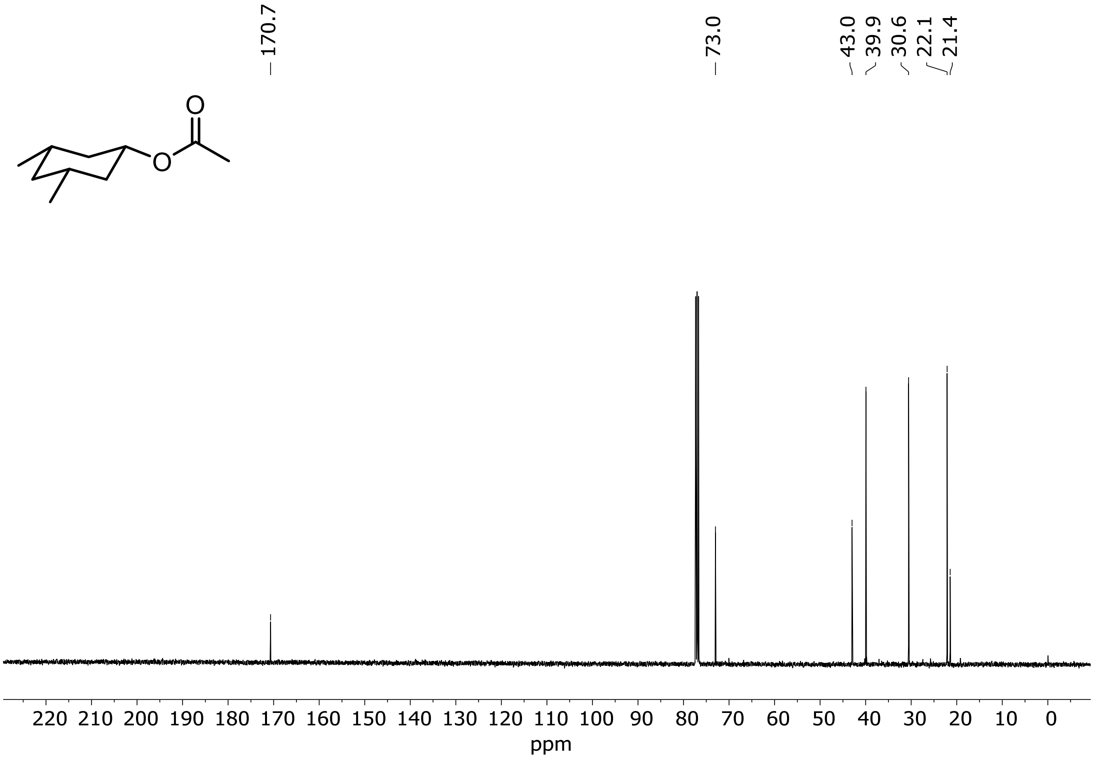

^{1}H-NMR of **3a** in $CDCl_3$

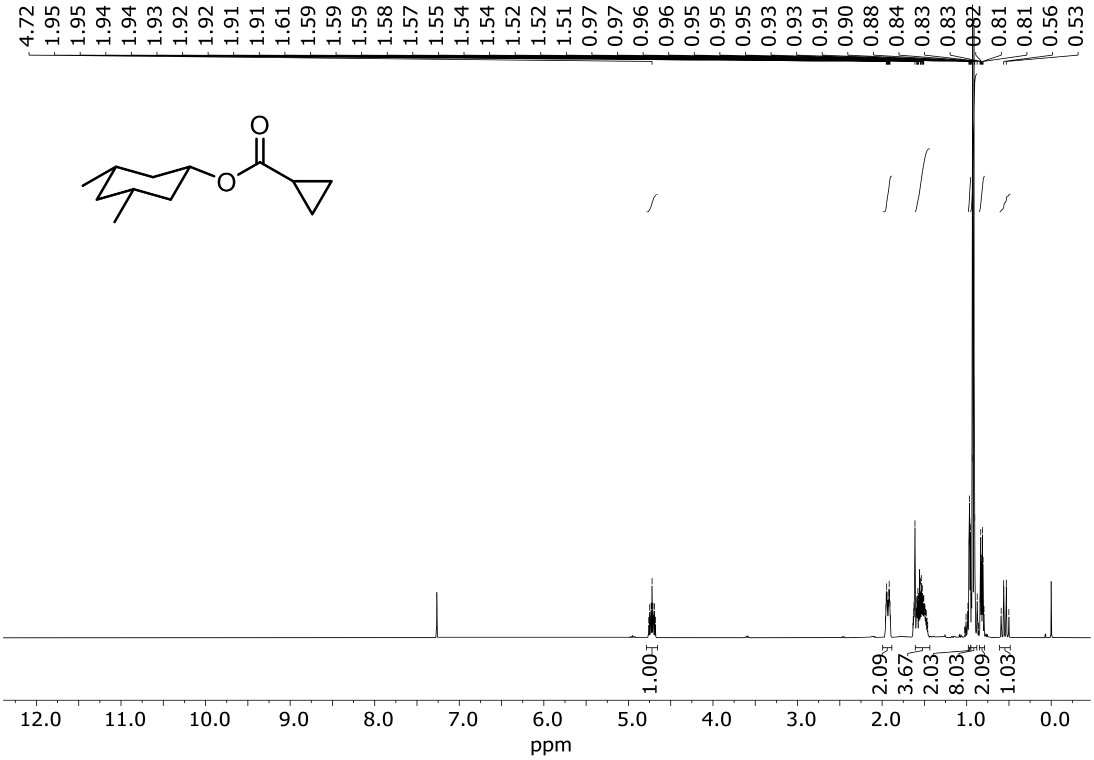

^{13}C-NMR of **3a** in $CDCl_3$

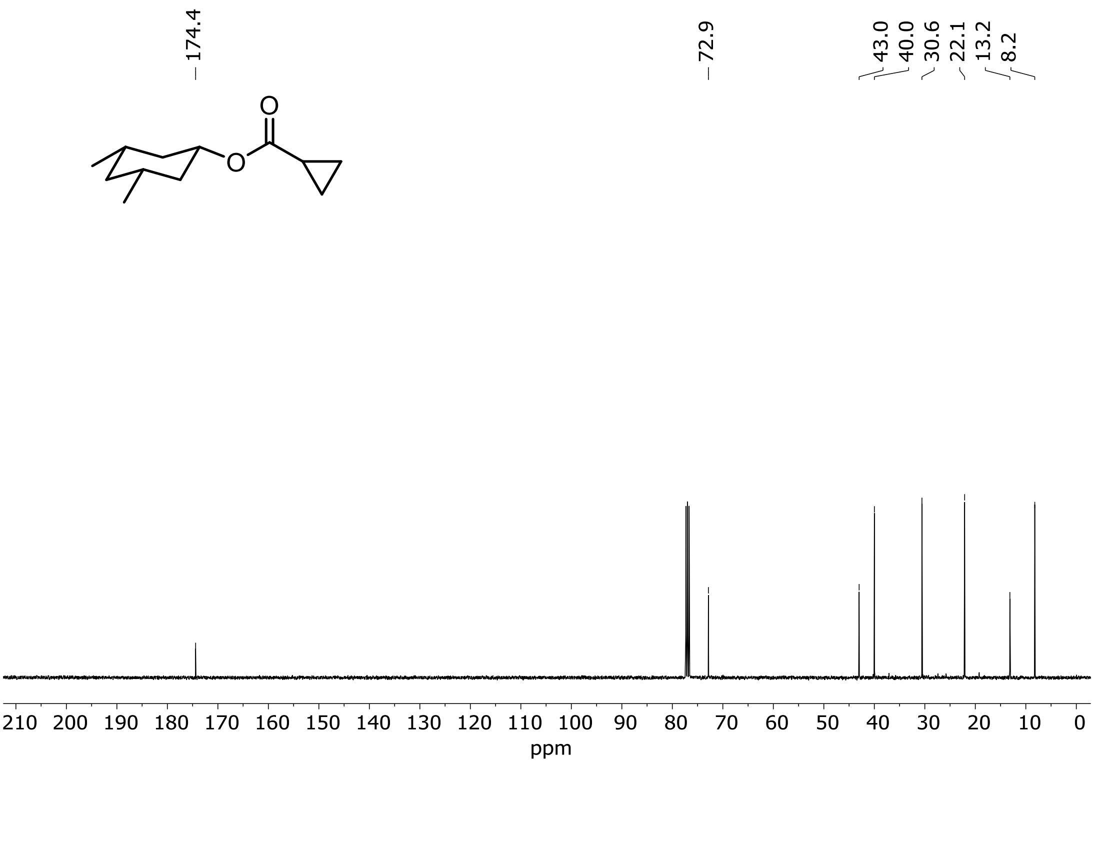

^{1}H-NMR of **3ax** in $CDCl_3$

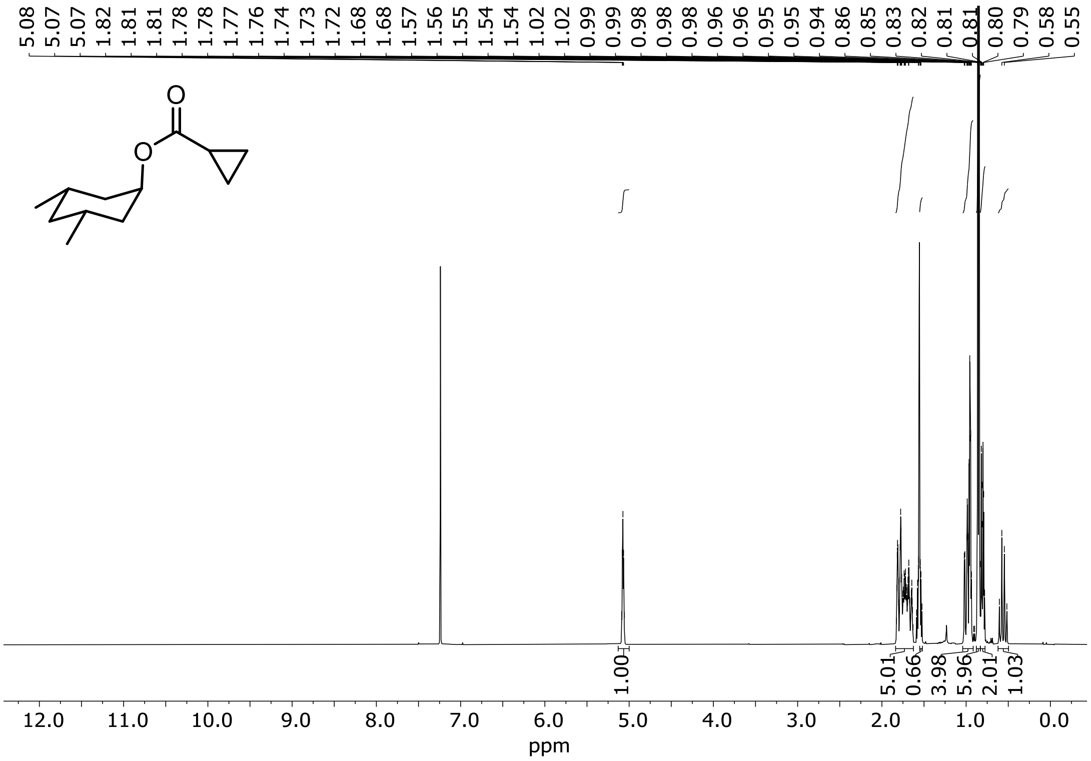

^{1}H-NMR of **3ax** in $CDCl_3$

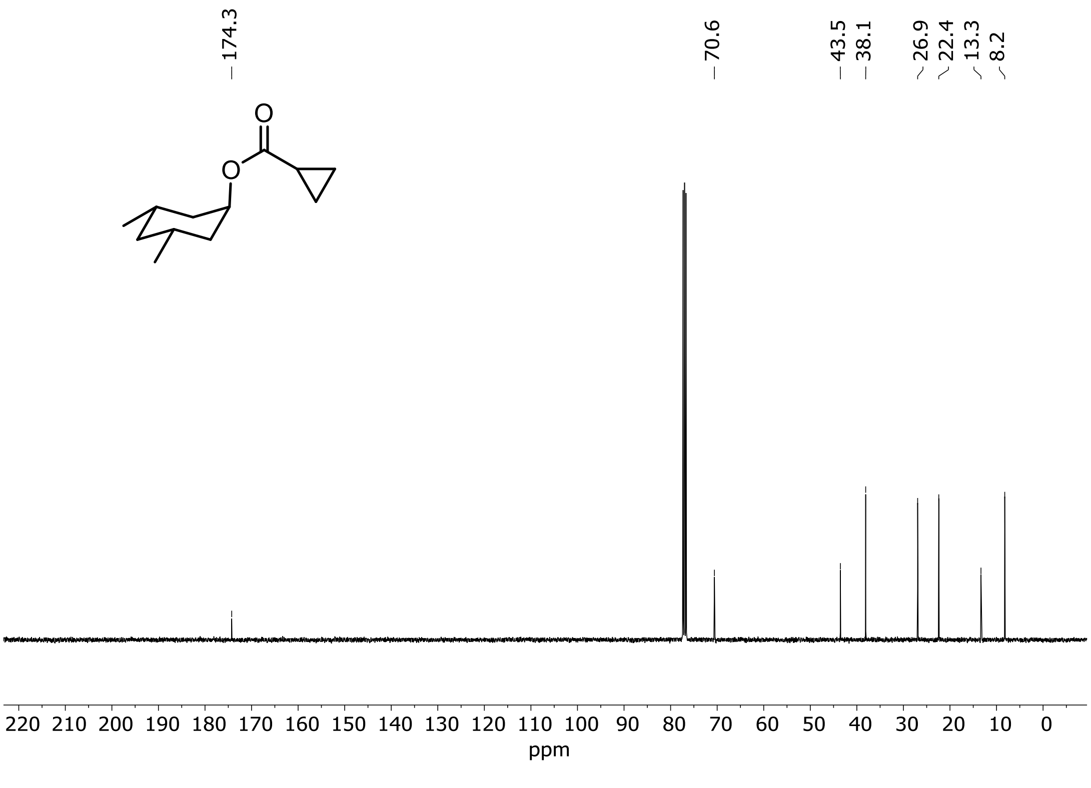

^{1}H-NMR of **4a** in $CDCl_3$

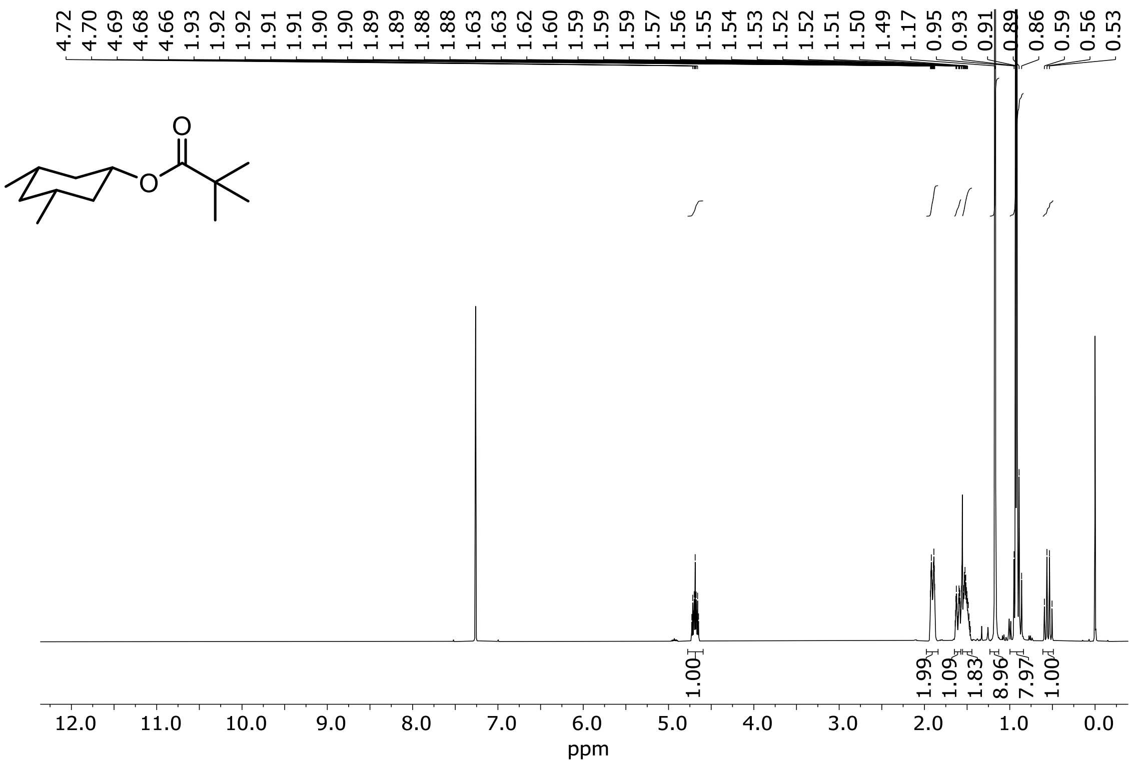

^{13}C-NMR of **4a** in $CDCl_3$

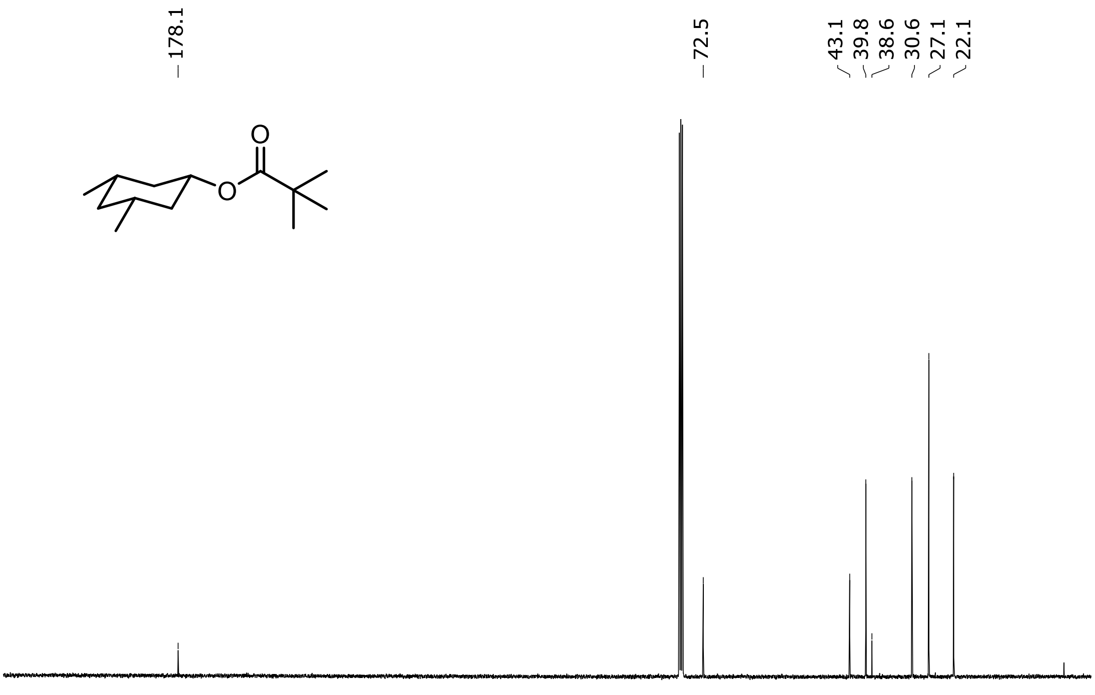

^{1}H-NMR of **5a** in $CDCl_3$

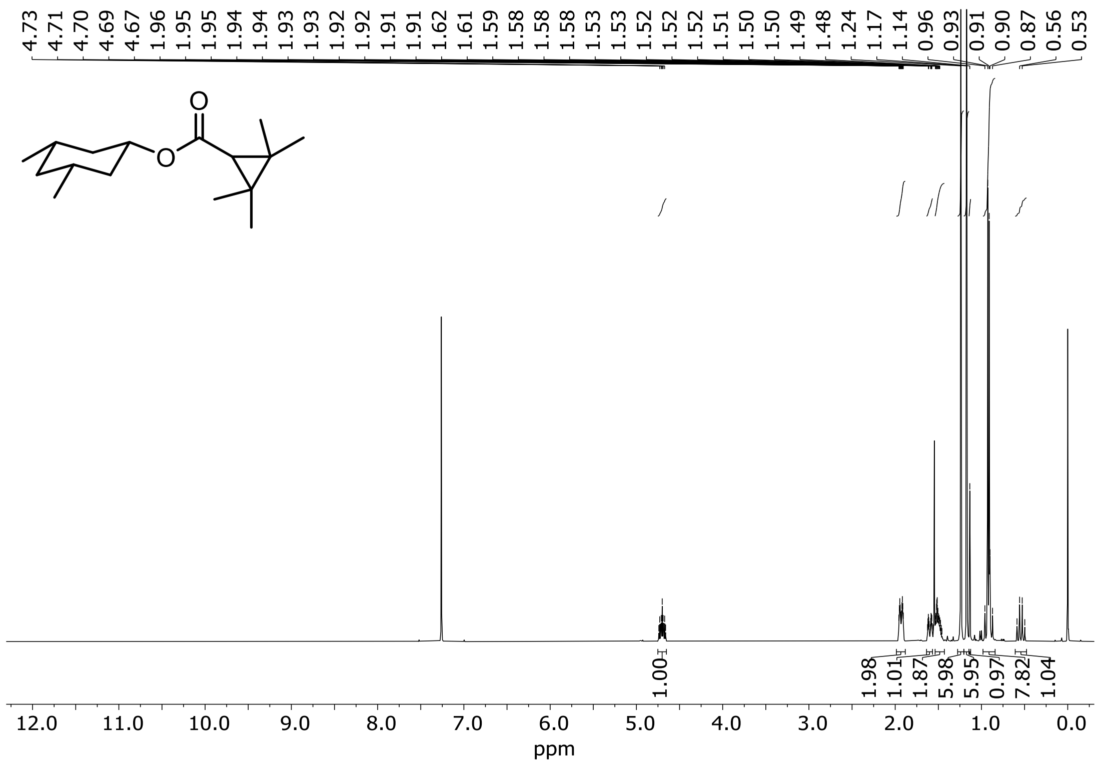

^{13}C-NMR of **5a** in $CDCl_3$

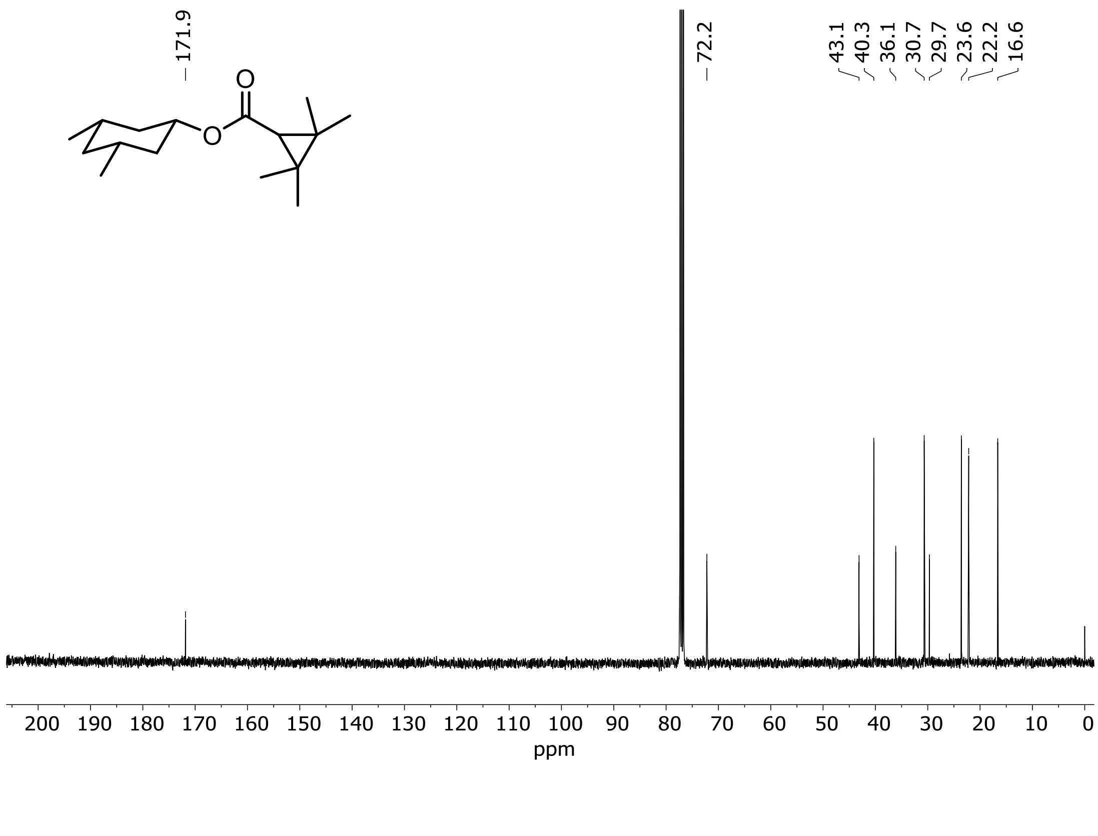

^{1}H-NMR of **6a** in $CDCl_3$

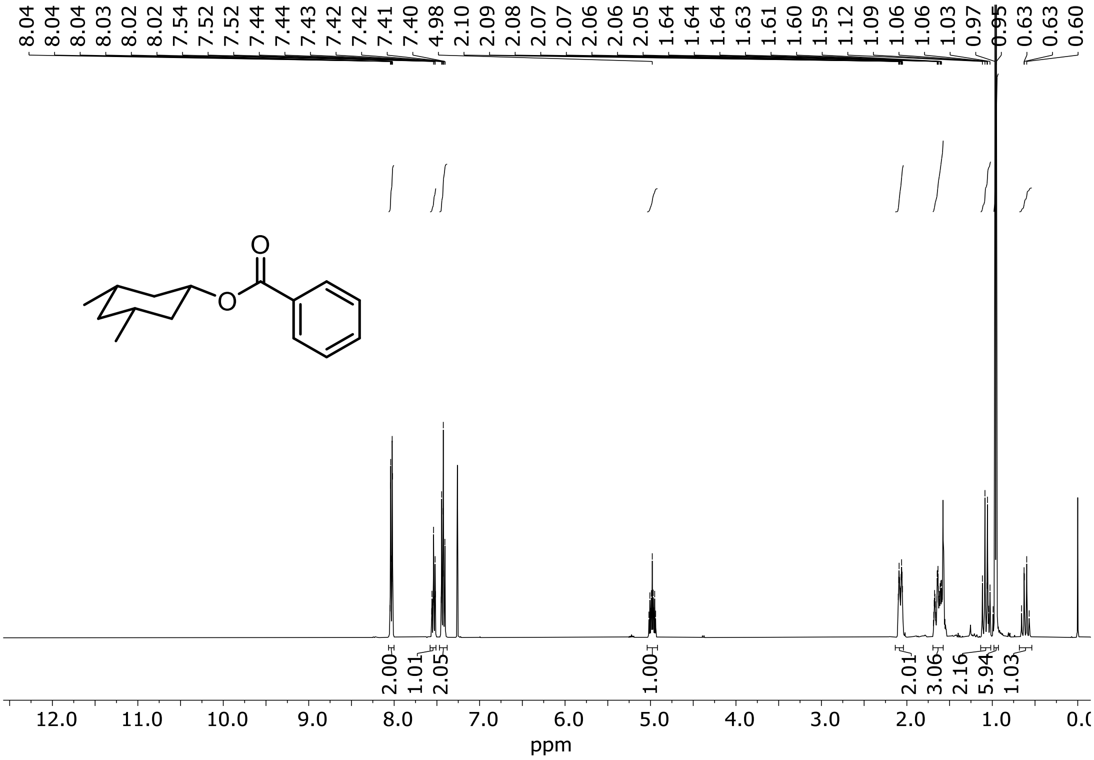

^{13}C-NMR of **6a** in $CDCl_3$

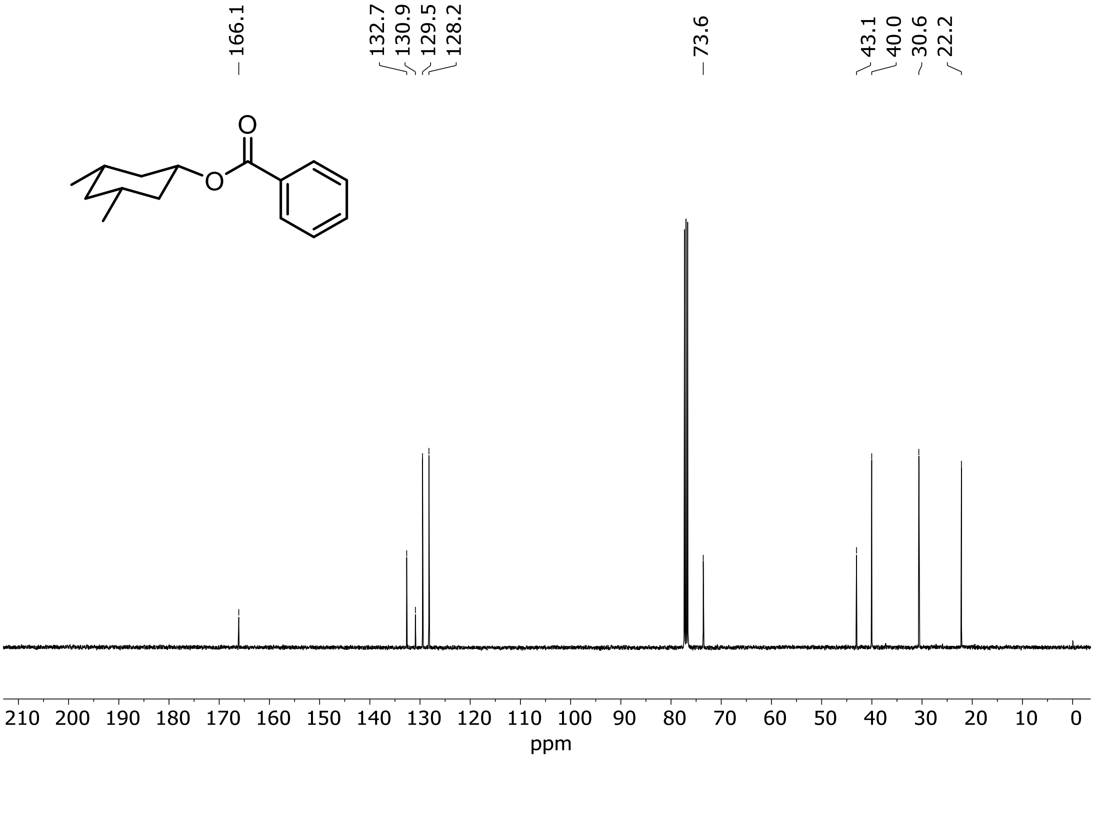

^{1}H-NMR of **7a** in $CDCl_3$

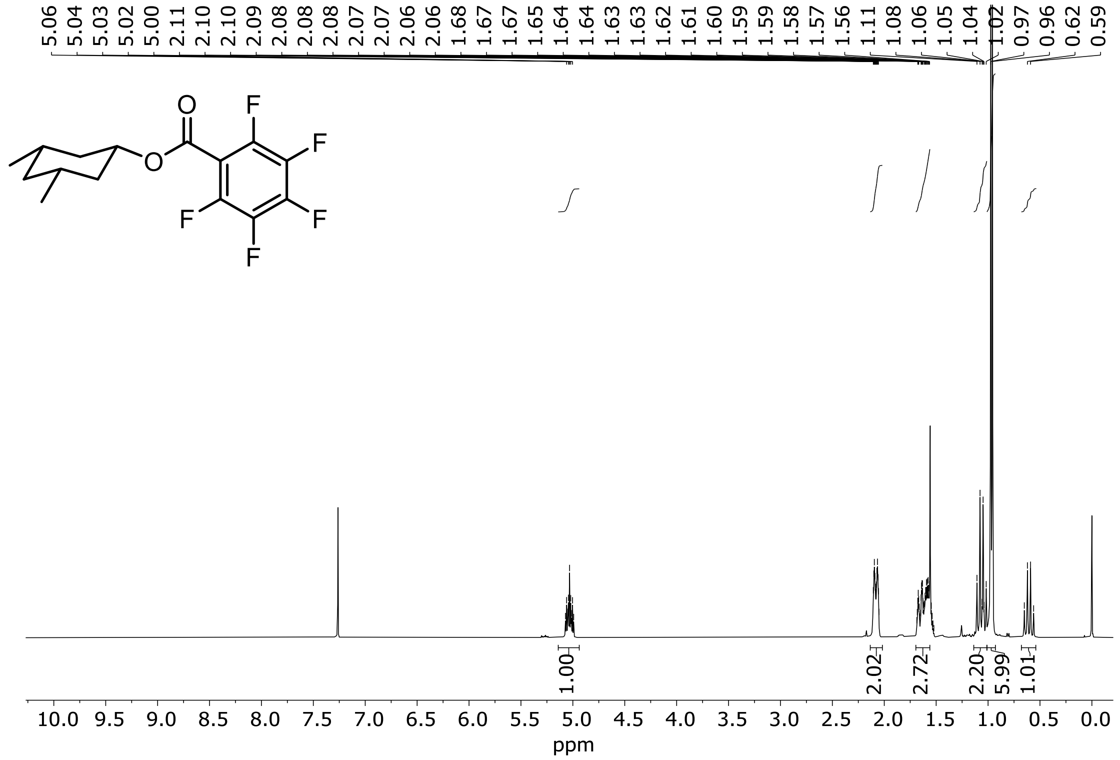

^{13}C-NMR of **7a** in $CDCl_3$

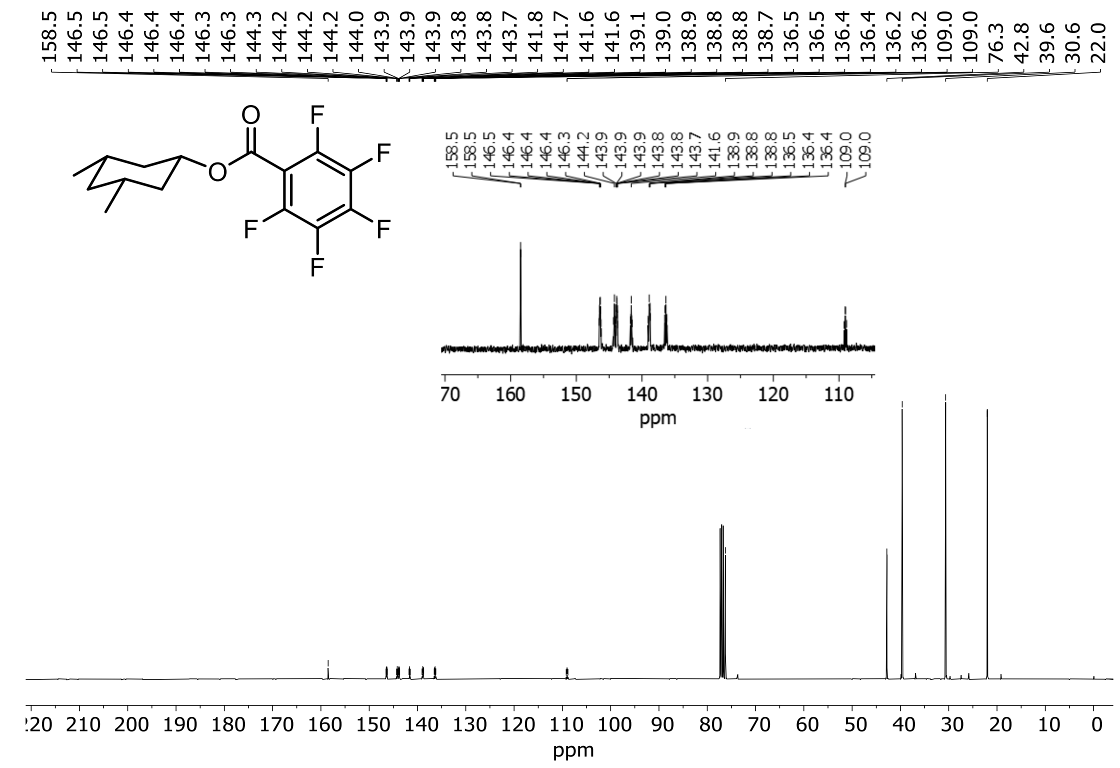

^{19}F-NMR of **7a** in $CDCl_3$

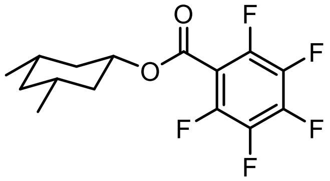

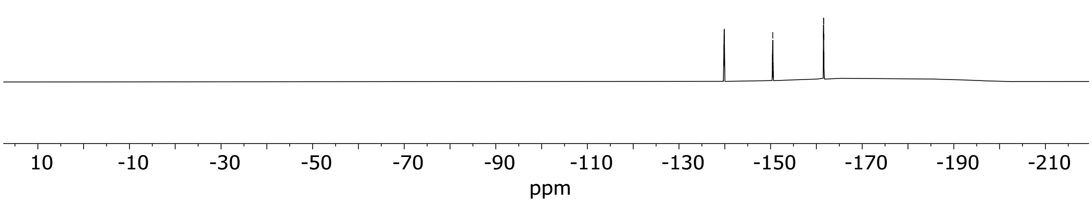

^{1}H-NMR of **Et2** in $CDCl_3$

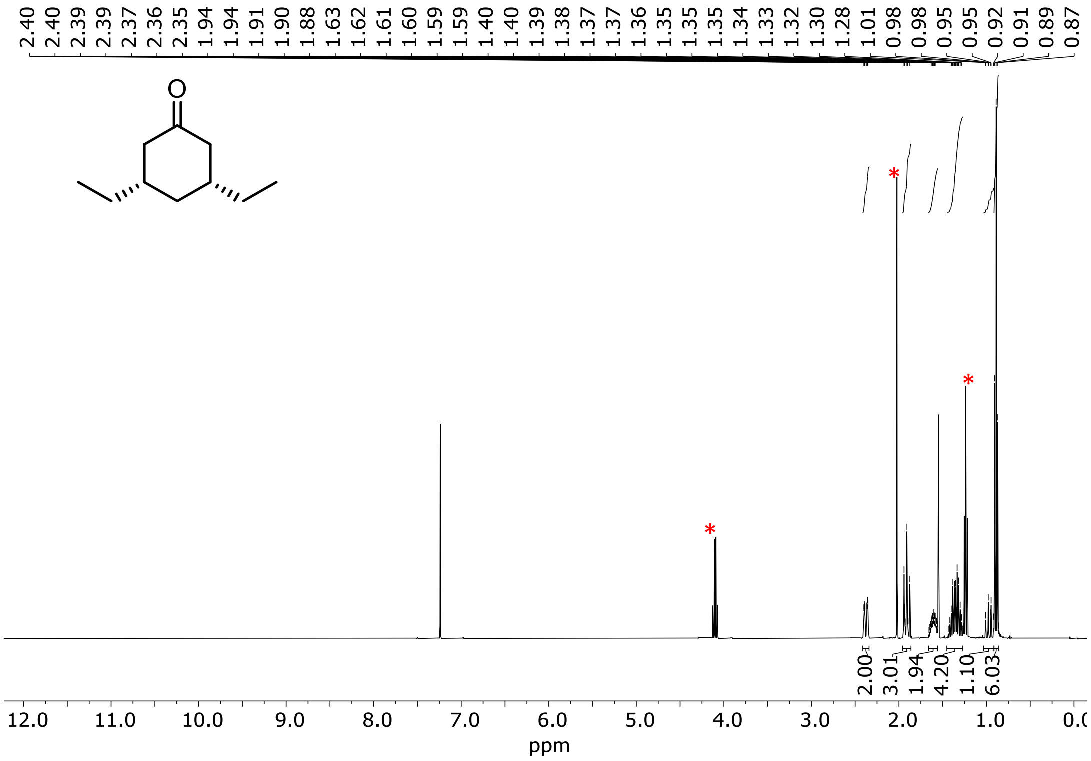

*Ethyl acetate

^{13}C-NMR of **Et2** in $CDCl_3$

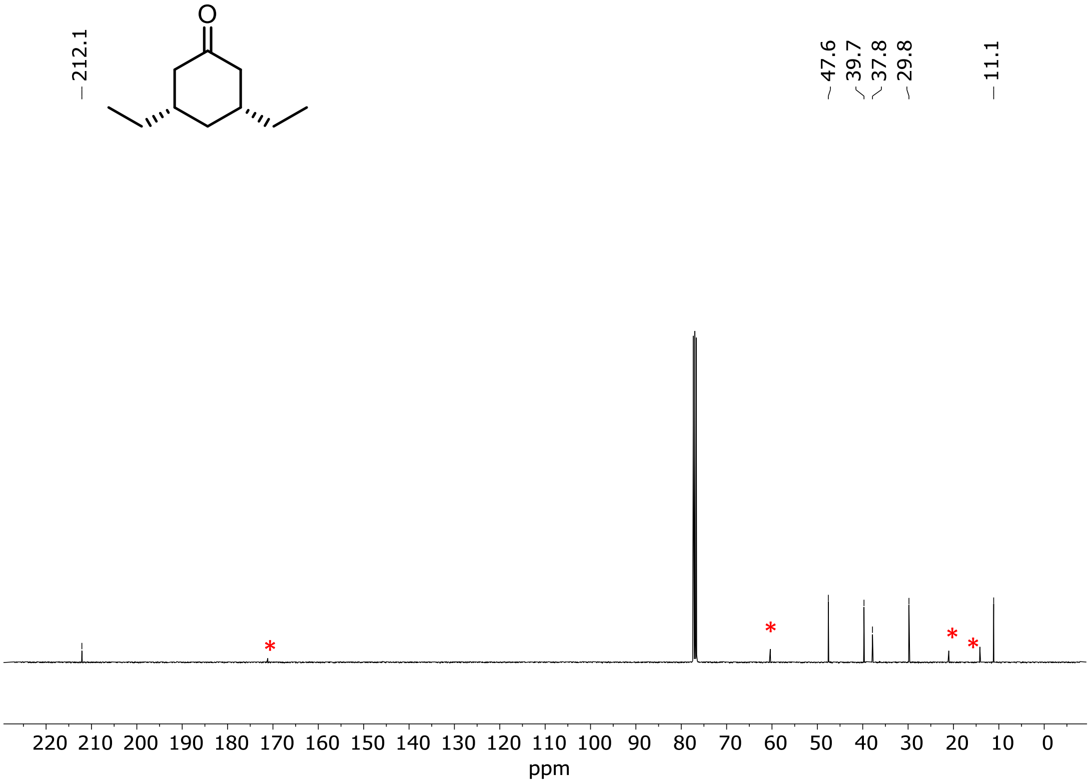

*Ethyl acetate

^{1}H-NMR of **Et3** in $CDCl_3$

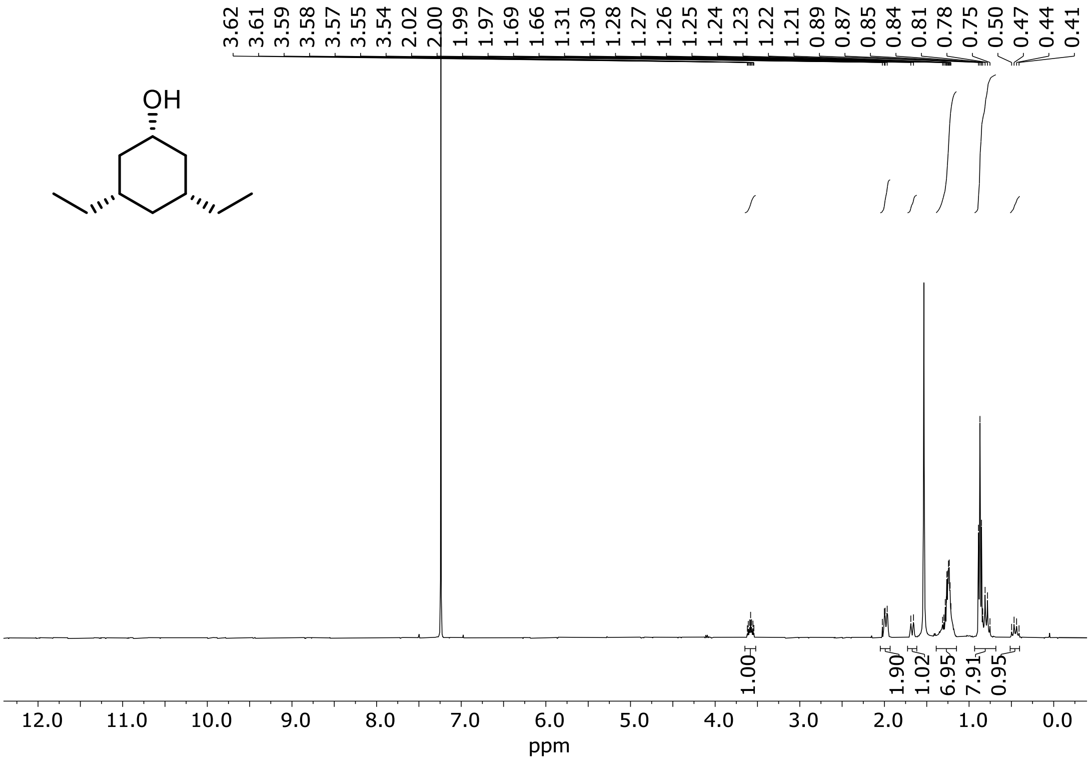

^{13}C-NMR of **Et3** in $CDCl_3$

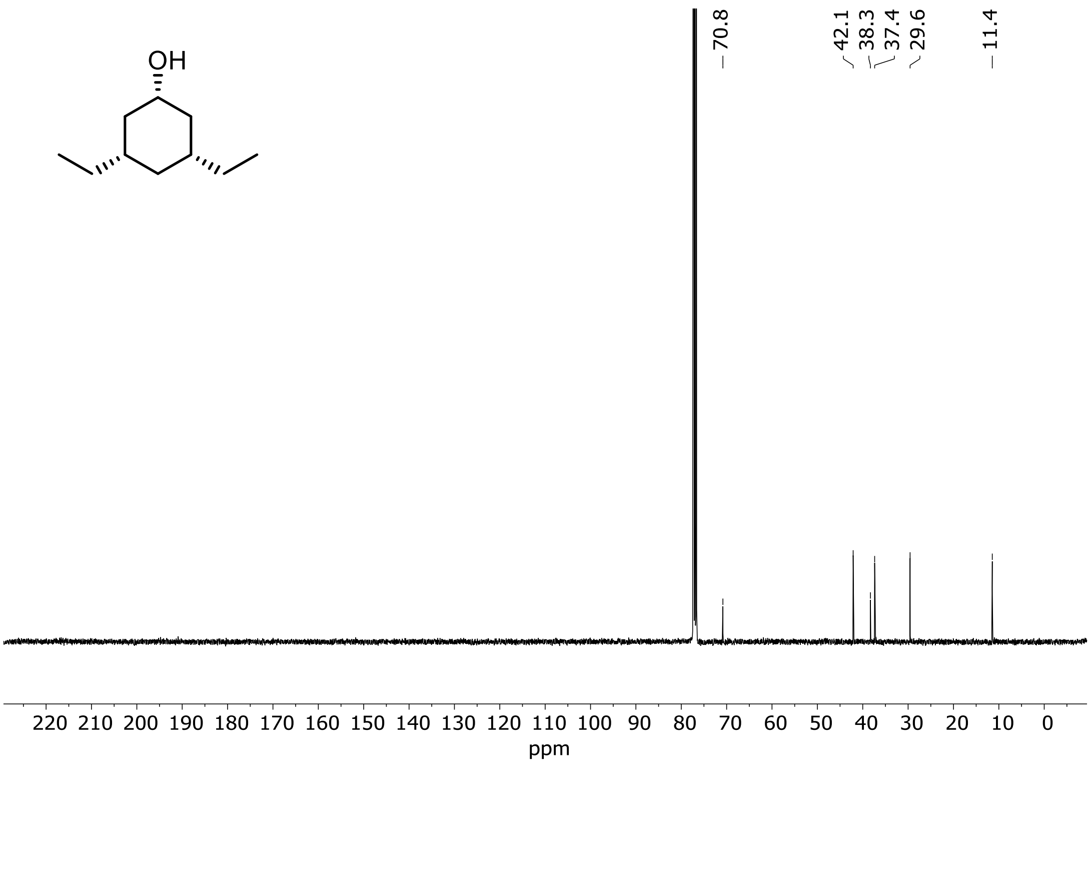

^{1}H-NMR of **8a** in $CDCl_3$

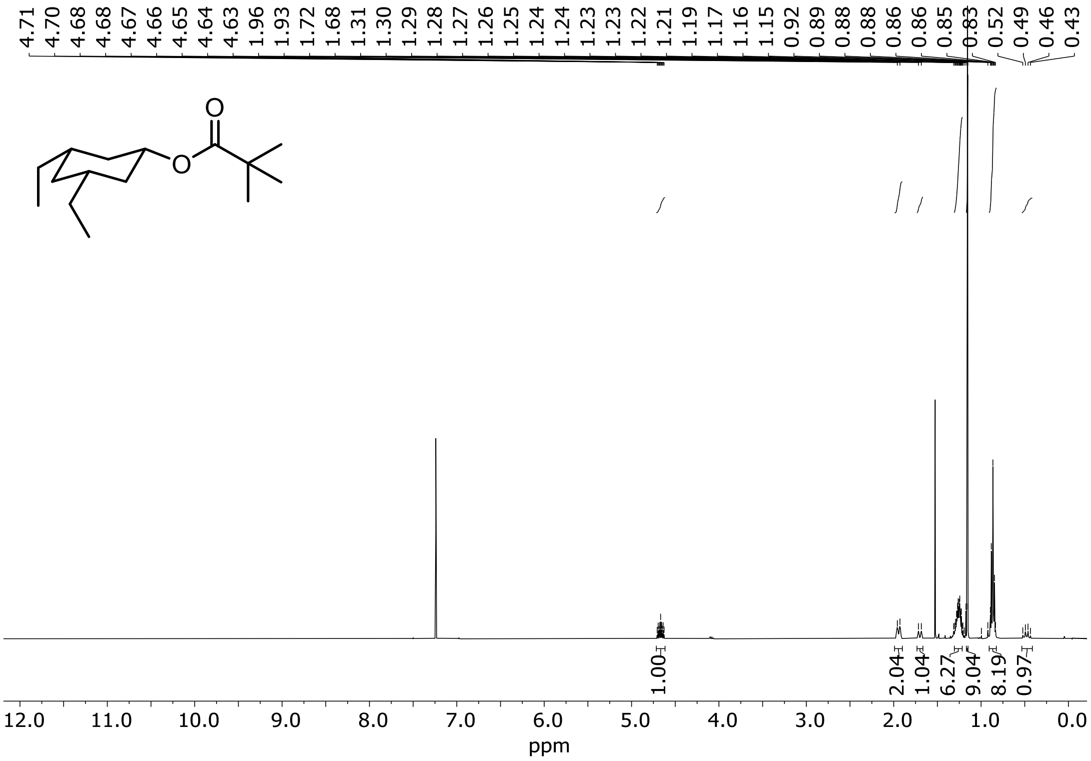

^{13}C-NMR of **8a** in $CDCl_3$

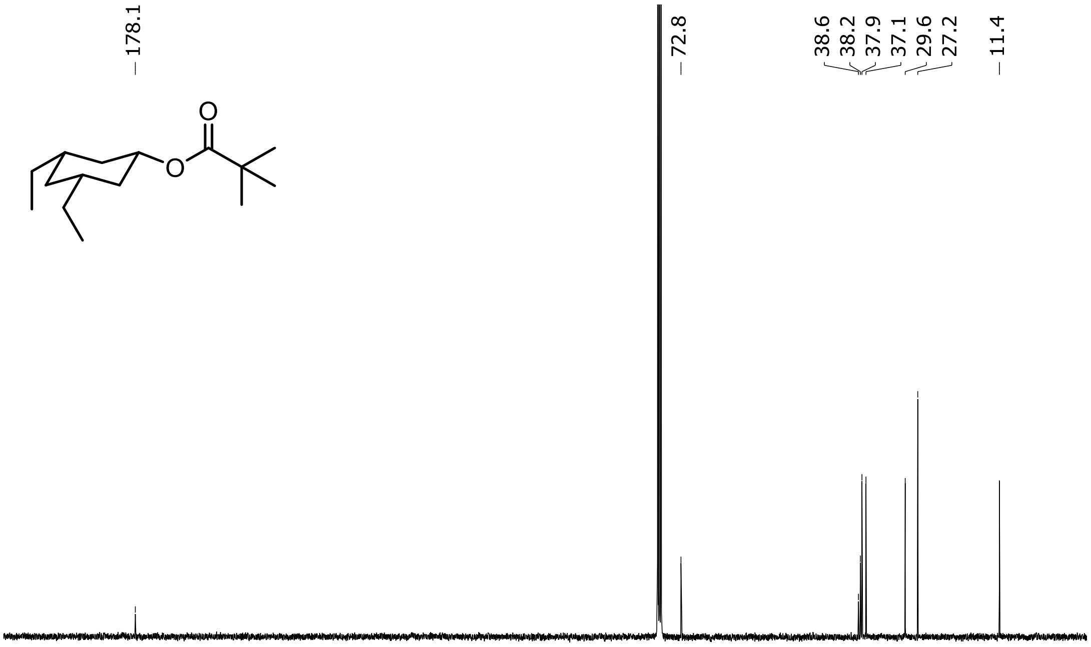

^{1}H-NMR of **9a** in CD_2Cl_2

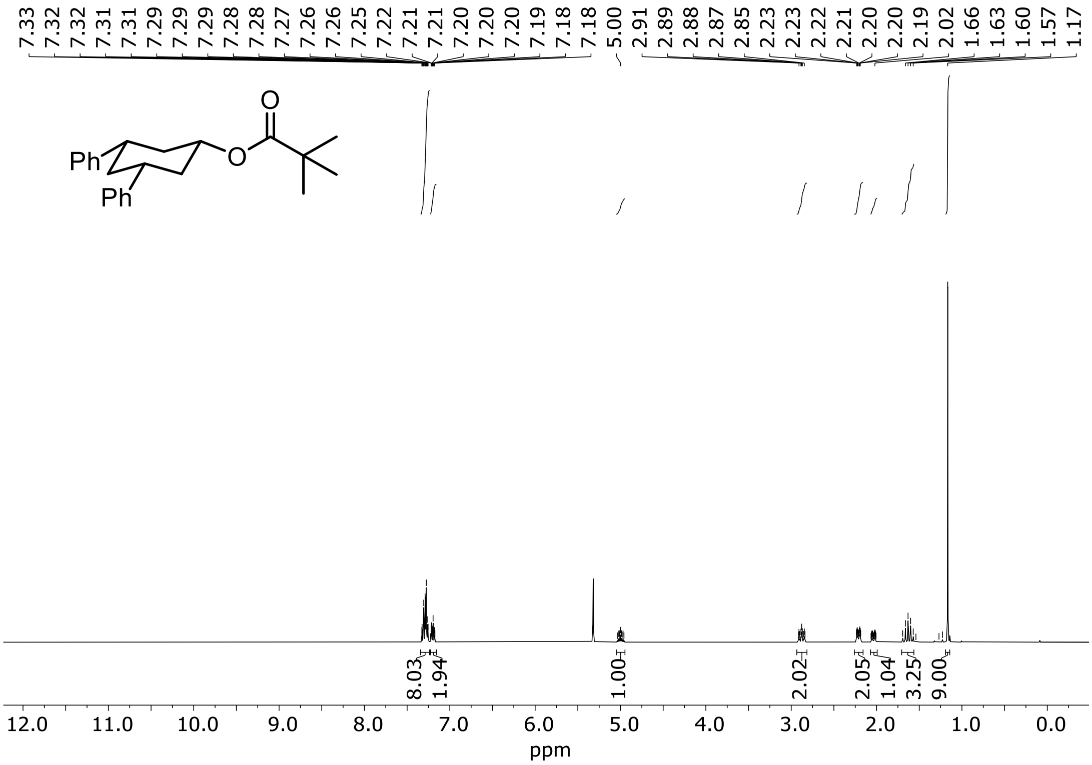

^{1}H-NMR of **9a** in CD_2Cl_2

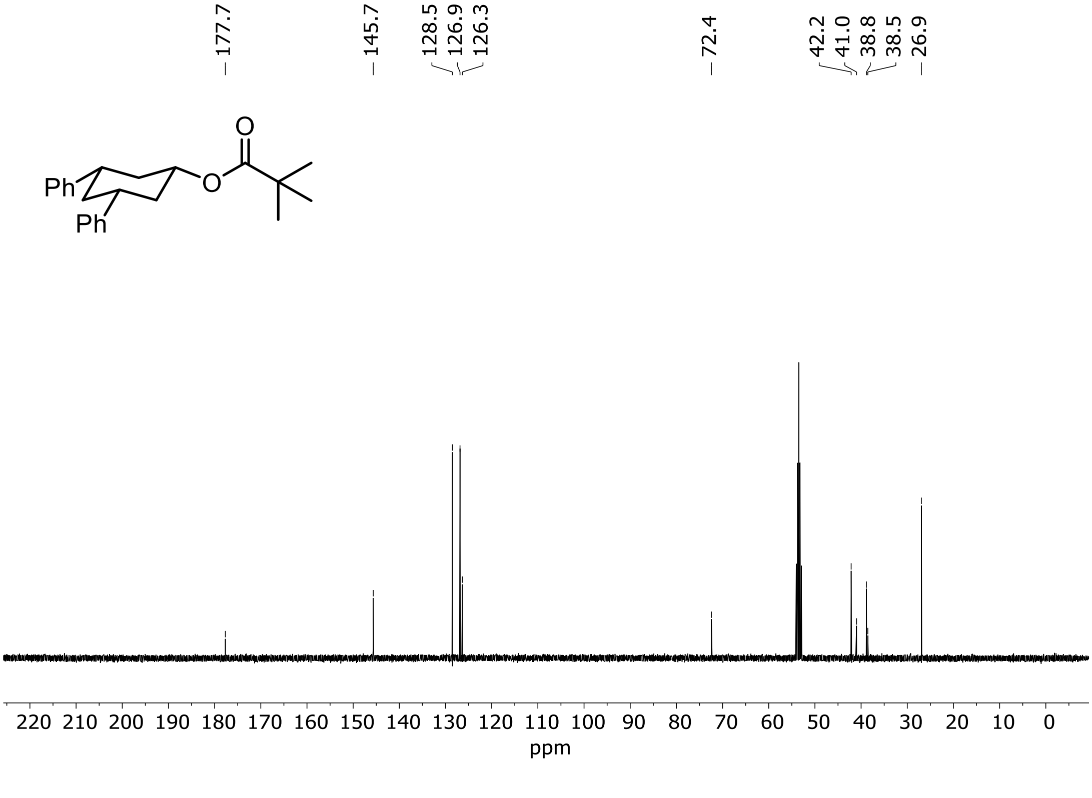

^{1}H-NMR of **10a** in $CDCl_3$

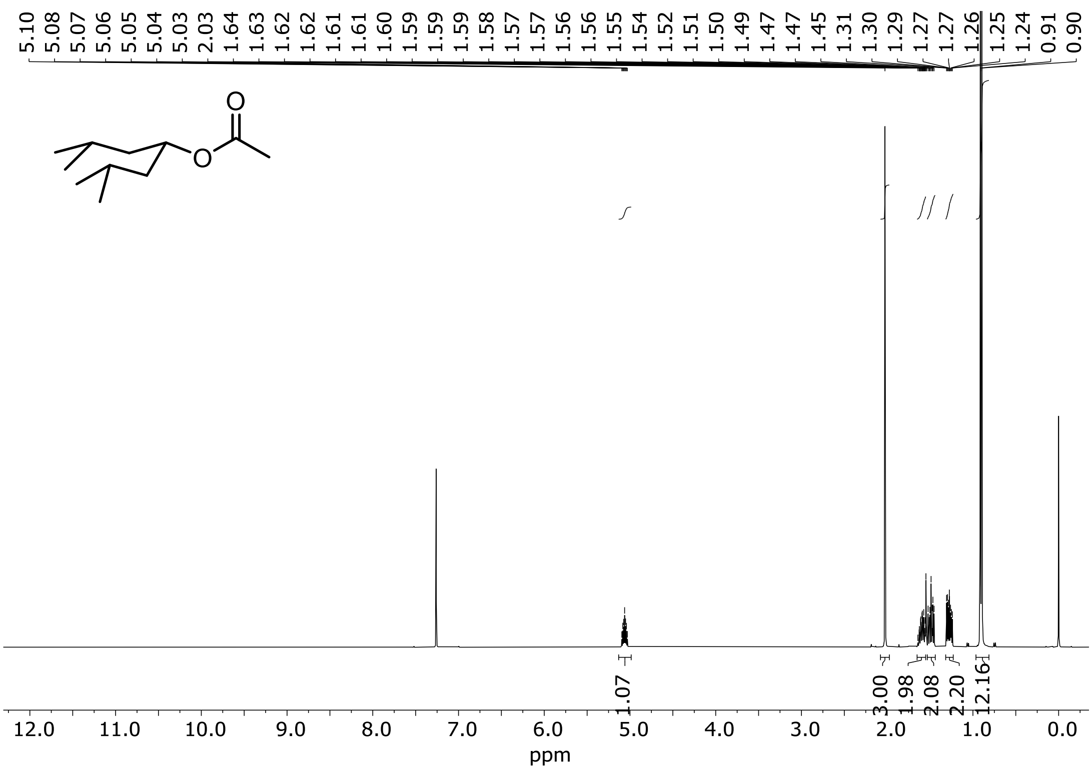

^{13}C-NMR of **10a** in $CDCl_3$

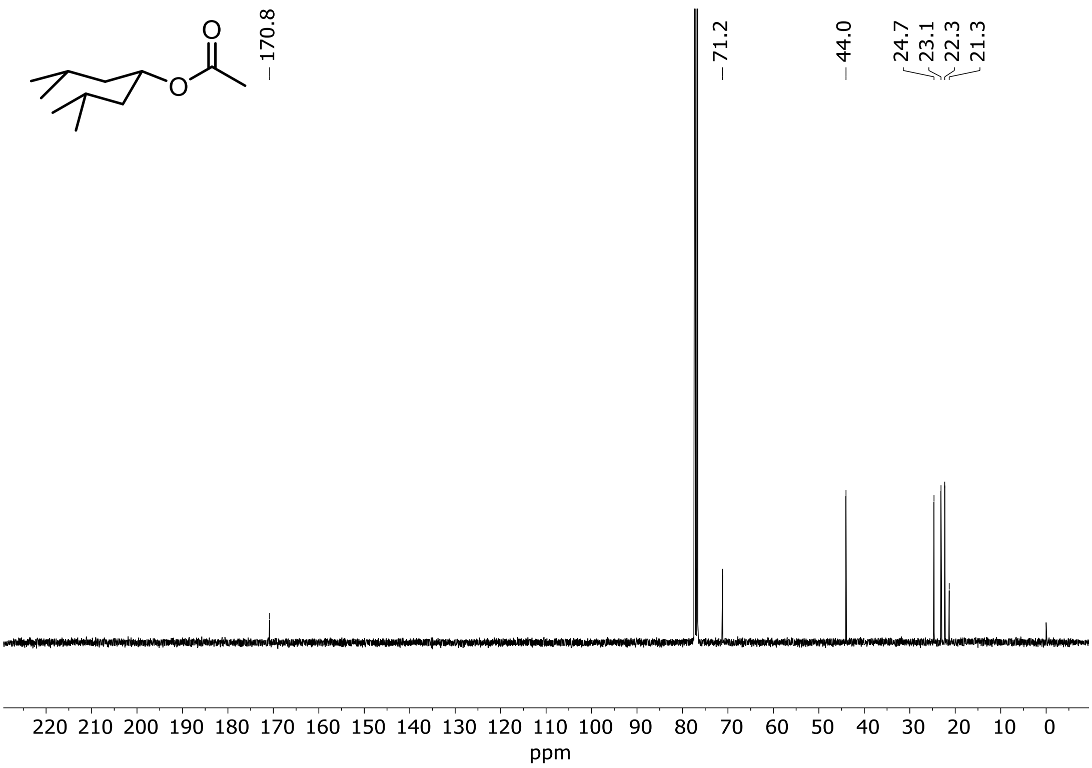

^{1}H-NMR of **B1** in $CDCl_3$

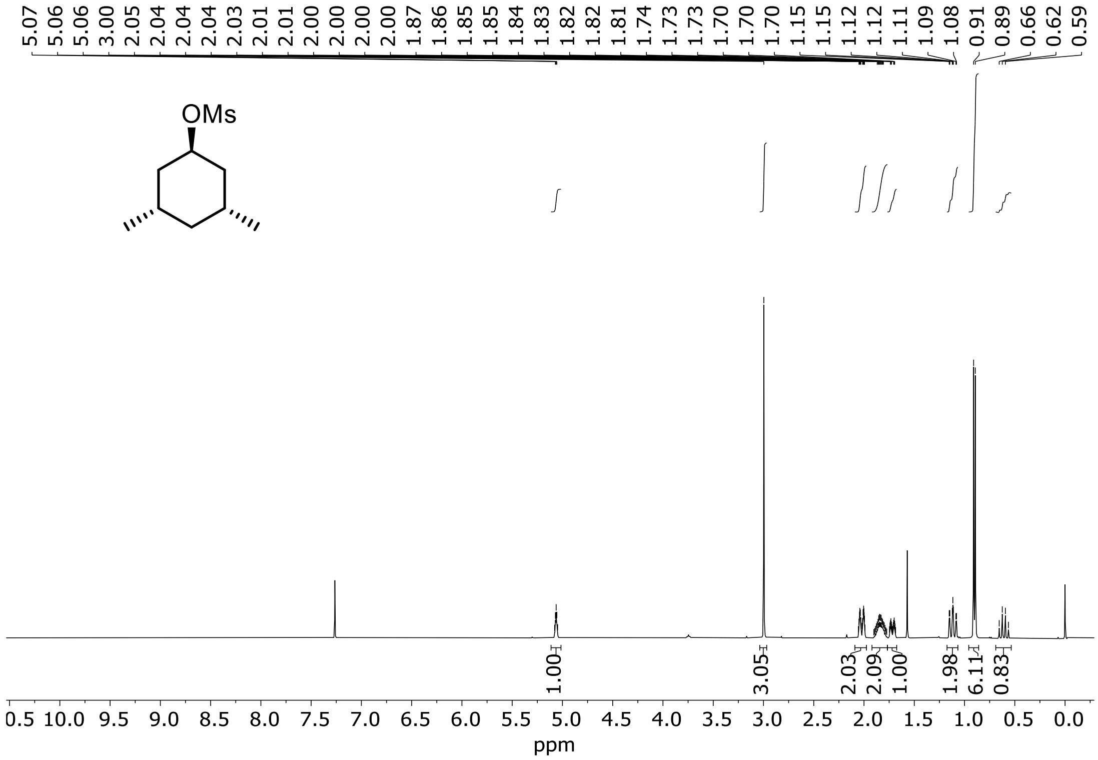

^{13}C-NMR of **B1** in $CDCl_3$

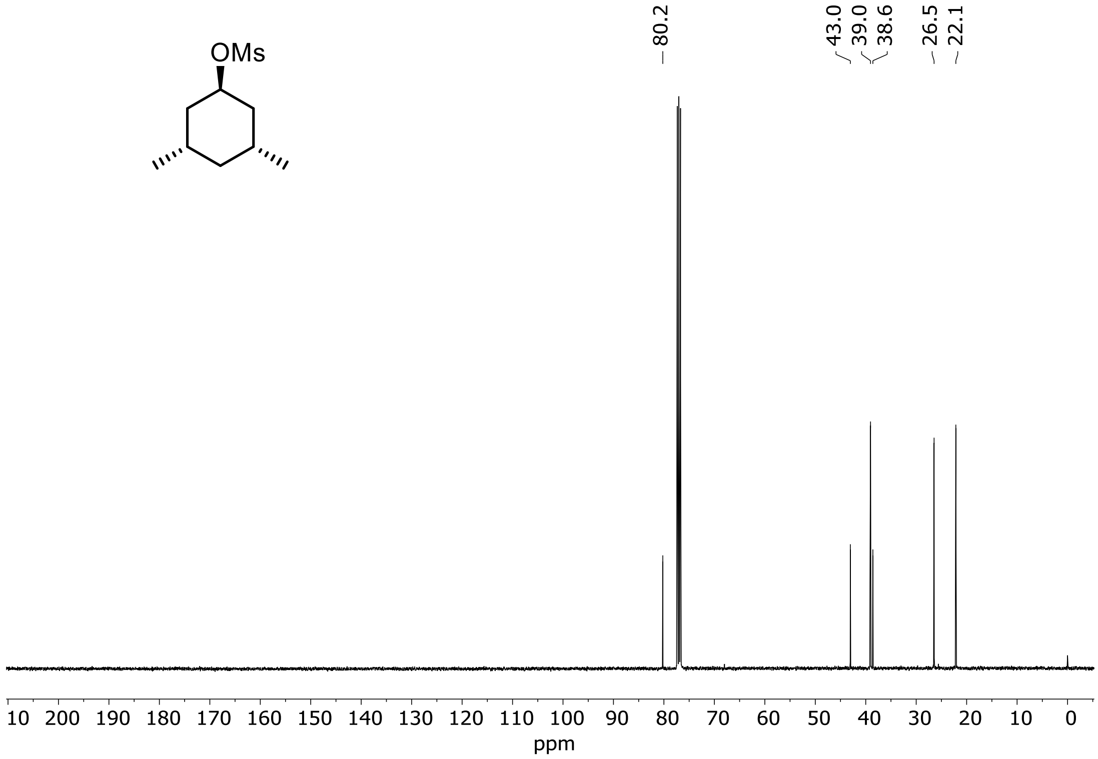

^{1}H-NMR of **B2** in $CDCl_3$

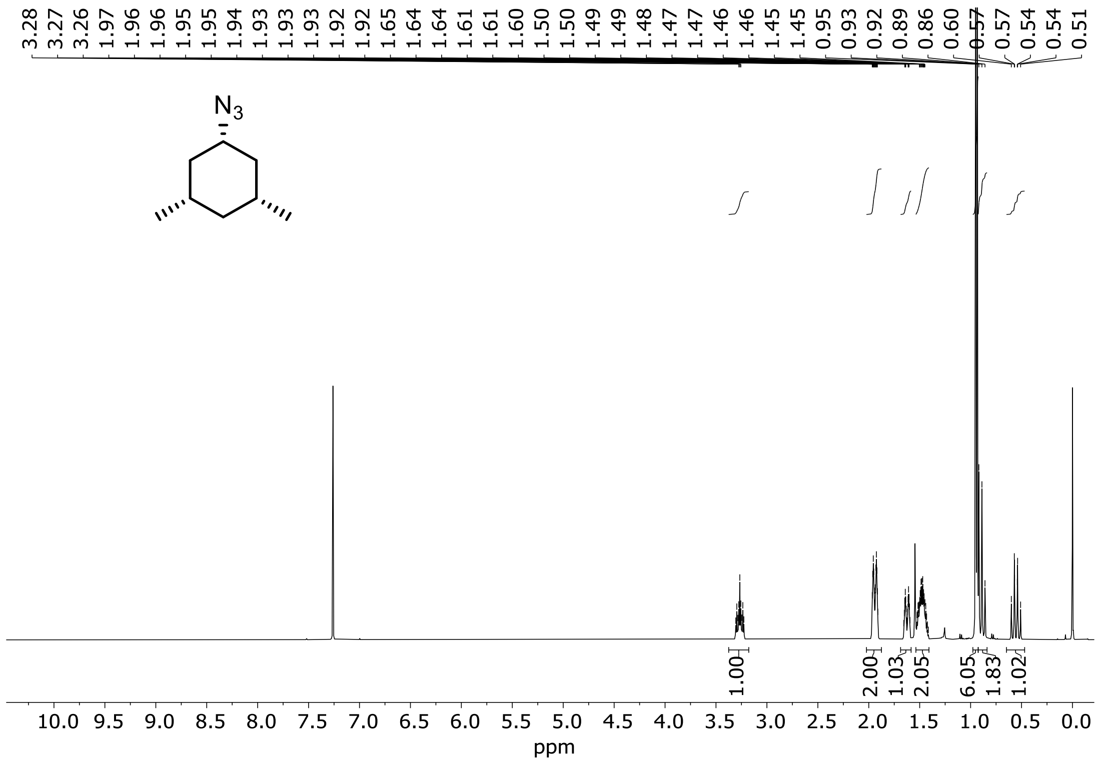

^{13}C-NMR of **B2** in $CDCl_3$

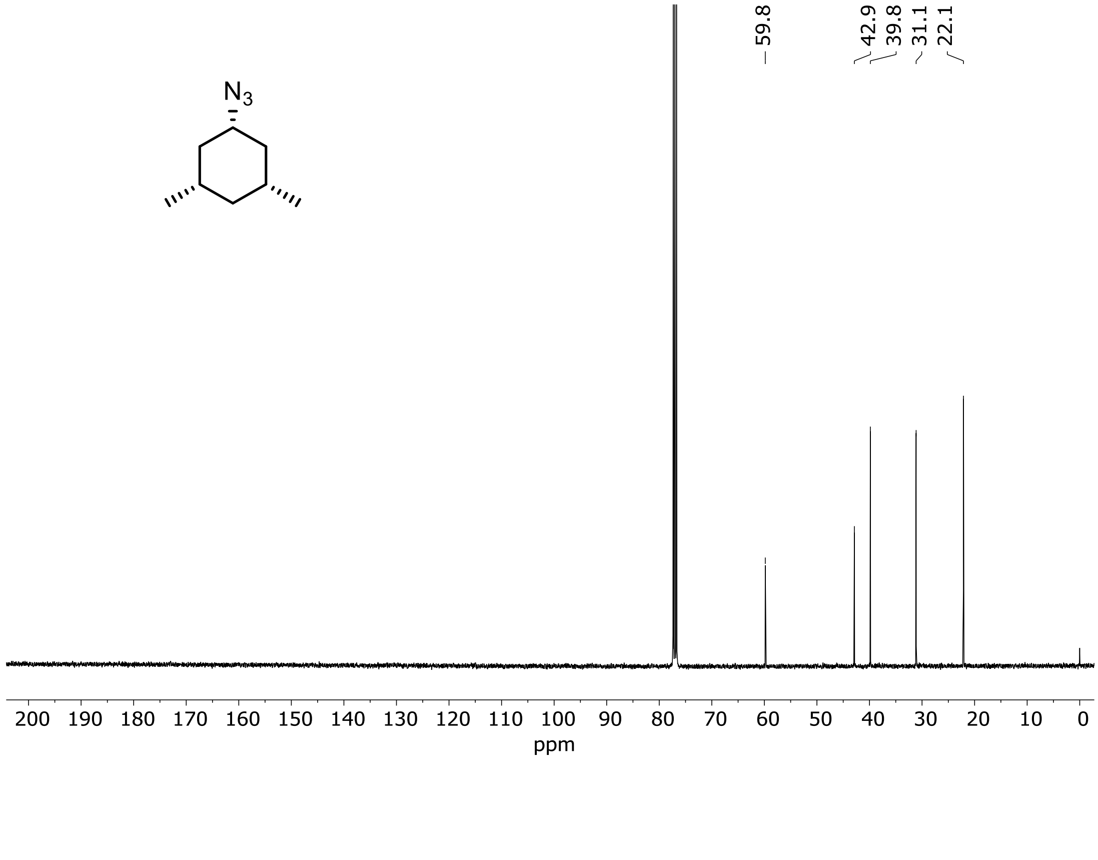

^{1}H-NMR of **11a** in $CDCl_3$

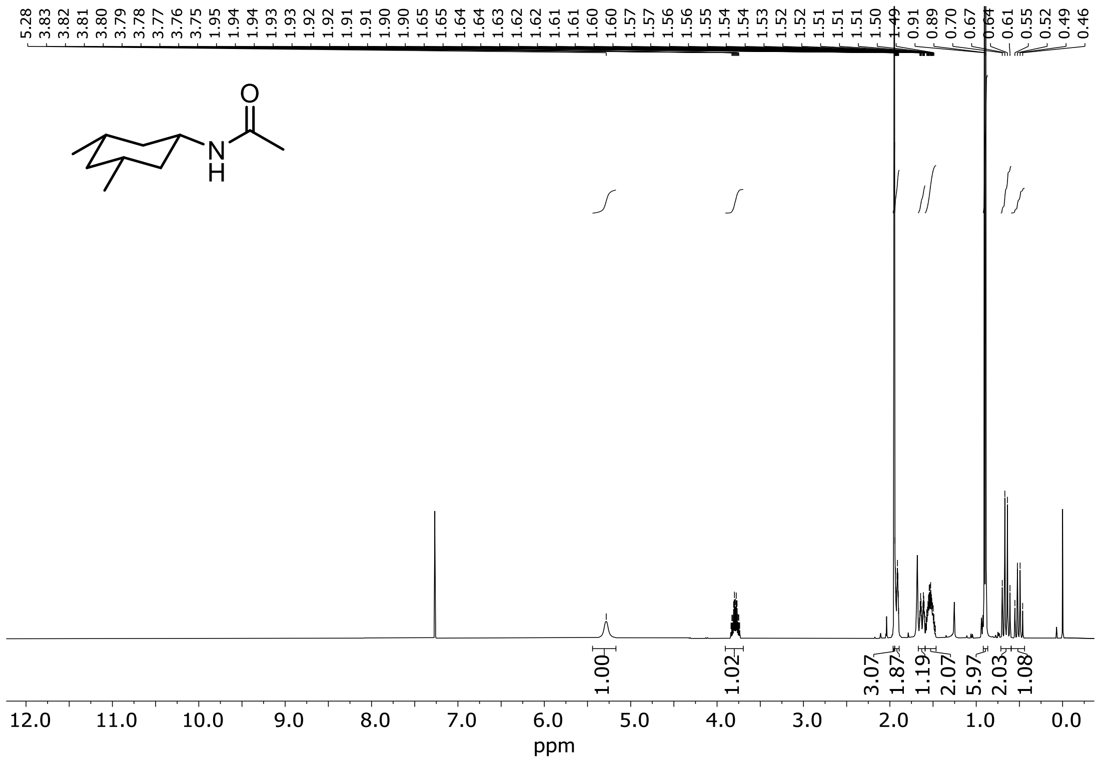

^{13}C-NMR of **11a** in $CDCl_3$

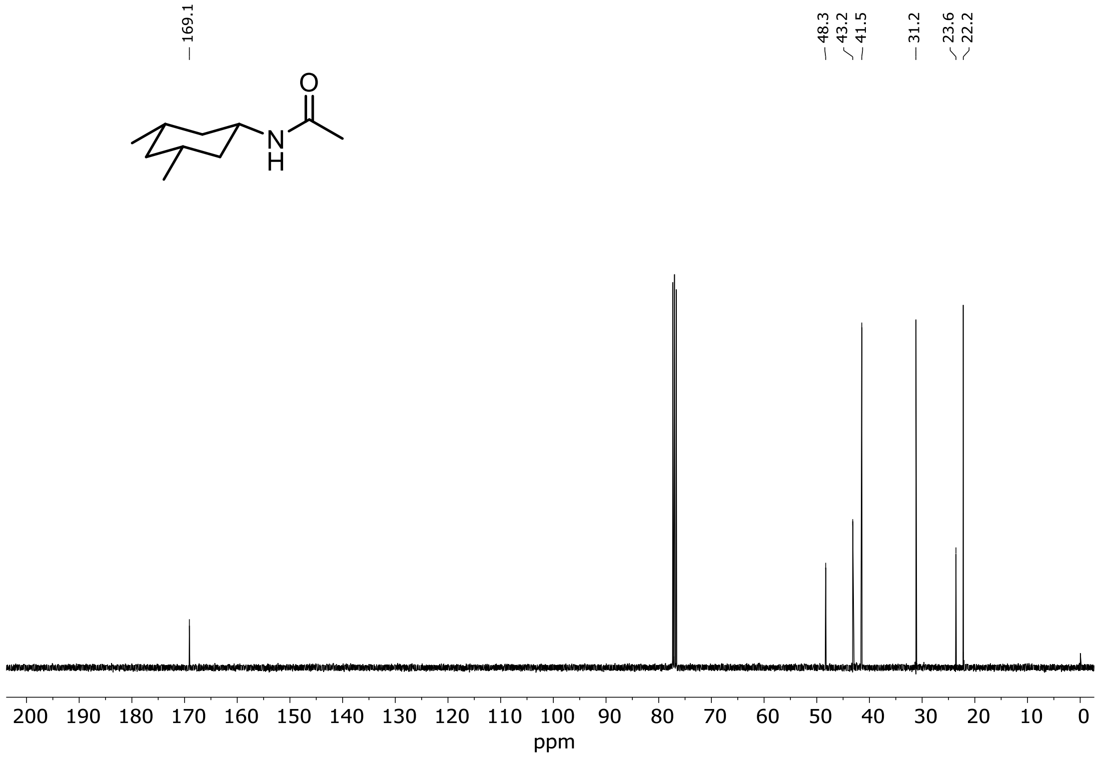

^{1}H-NMR of **12a** in CDCl$_3$

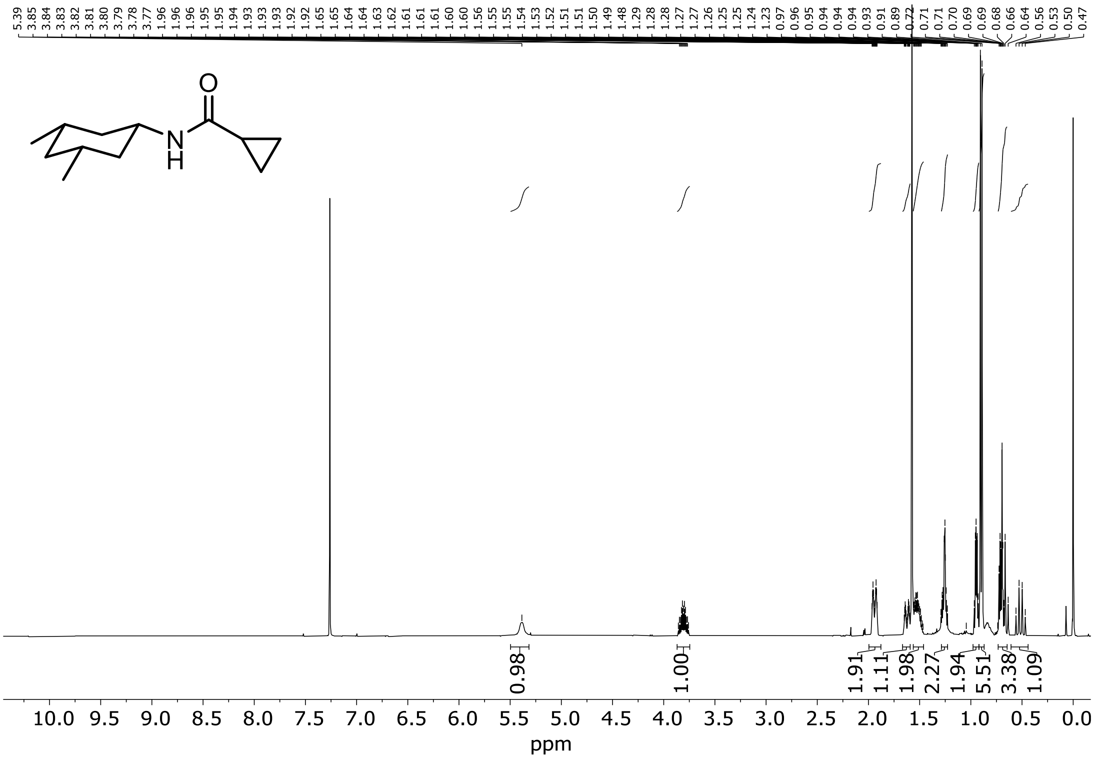

^{13}C-NMR of **12a** in CDCl$_3$

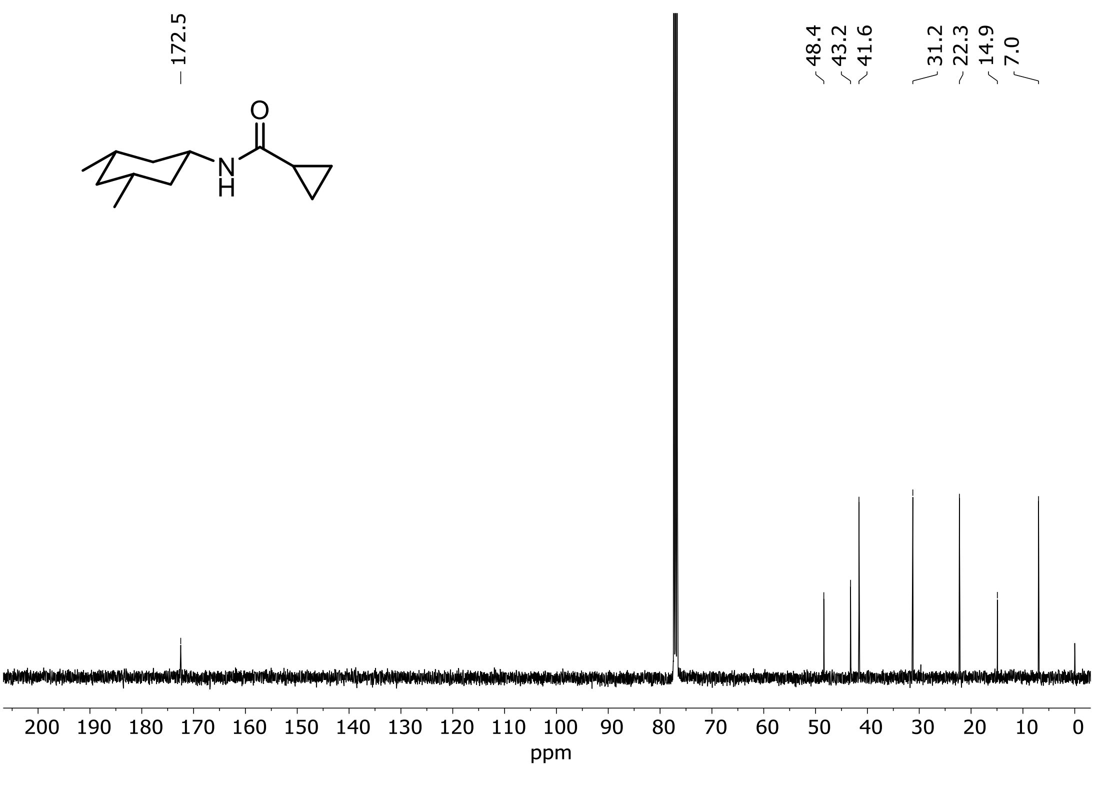

^{1}H-NMR of **13a** in CDCl$_3$

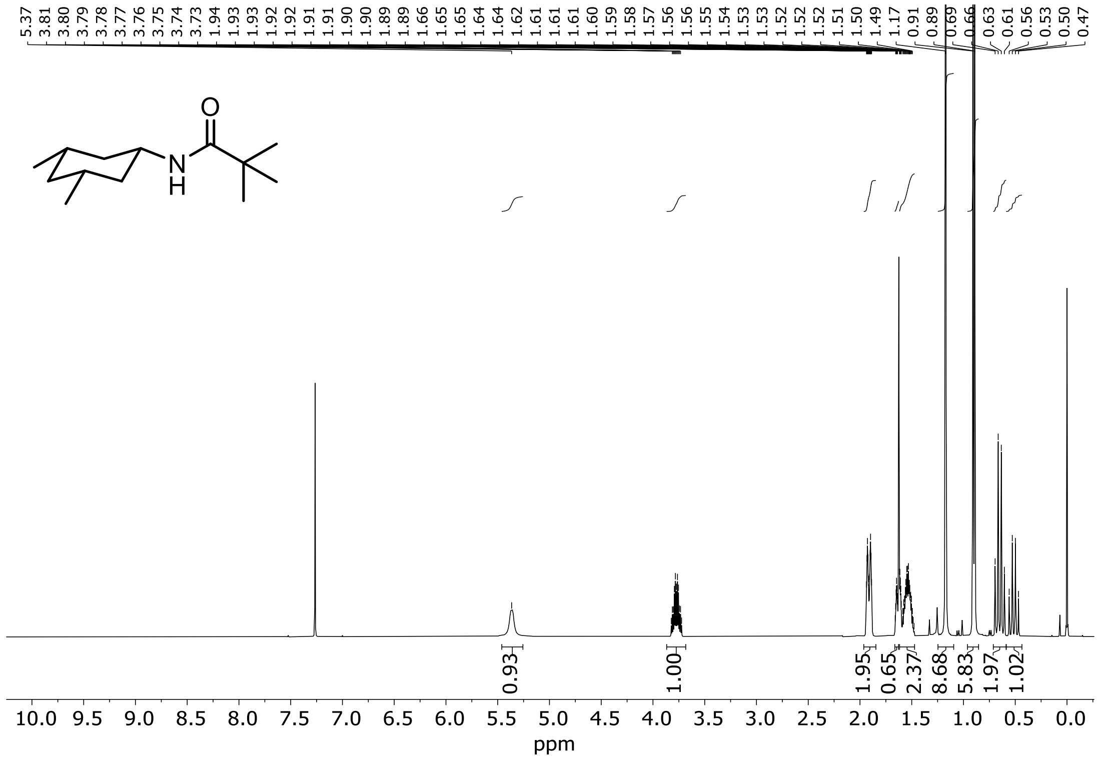

^{13}C-NMR of **13a** in CDCl$_3$

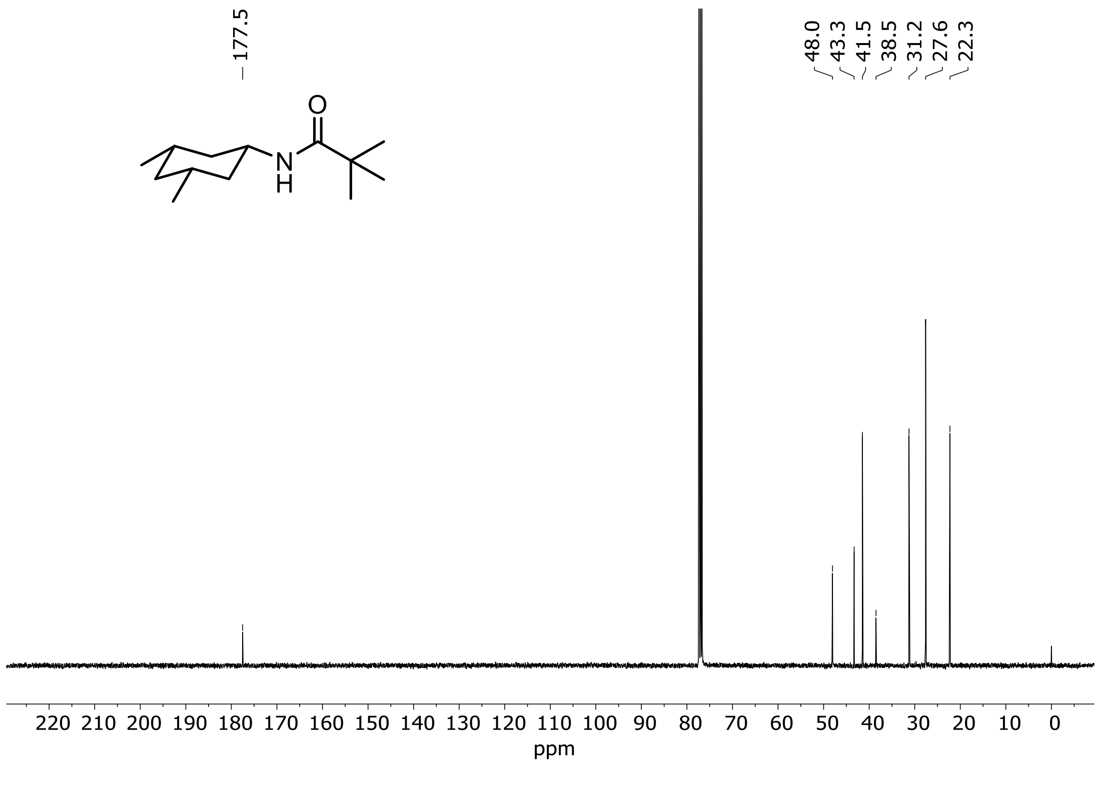

^{1}H-NMR of **14a** in $CDCl_3$

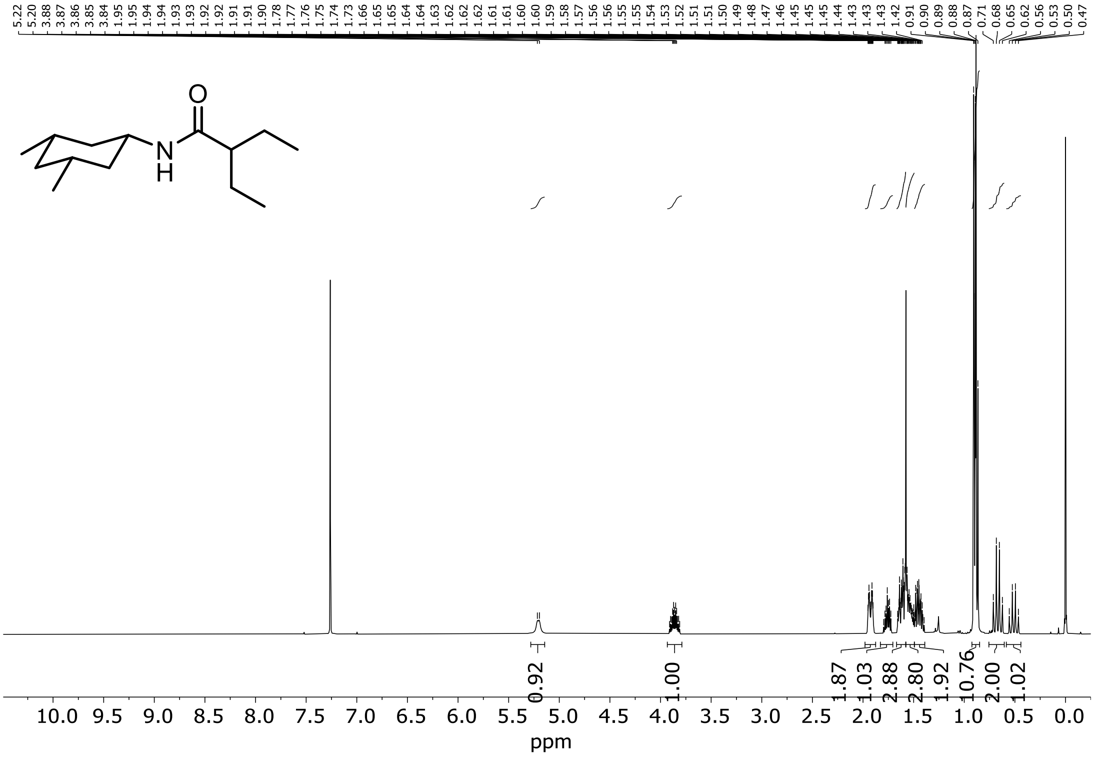

^{13}C-NMR of **14a** in $CDCl_3$

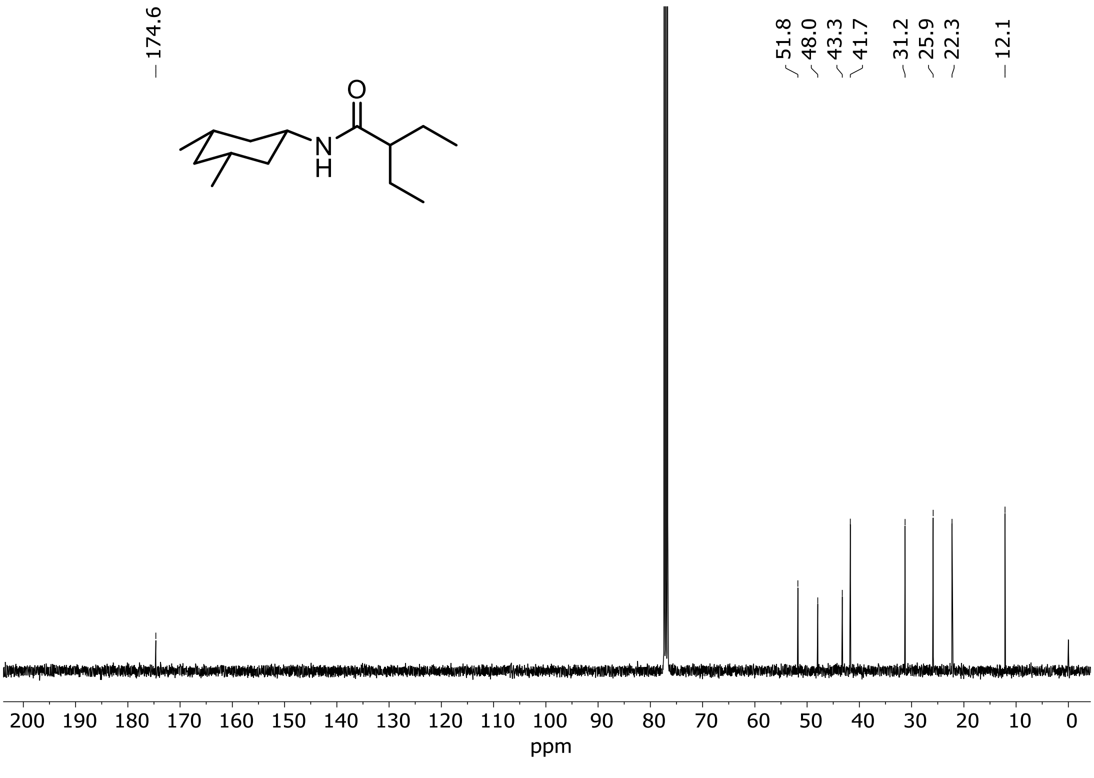

^{1}H-NMR of **10Ms** in $CDCl_3$

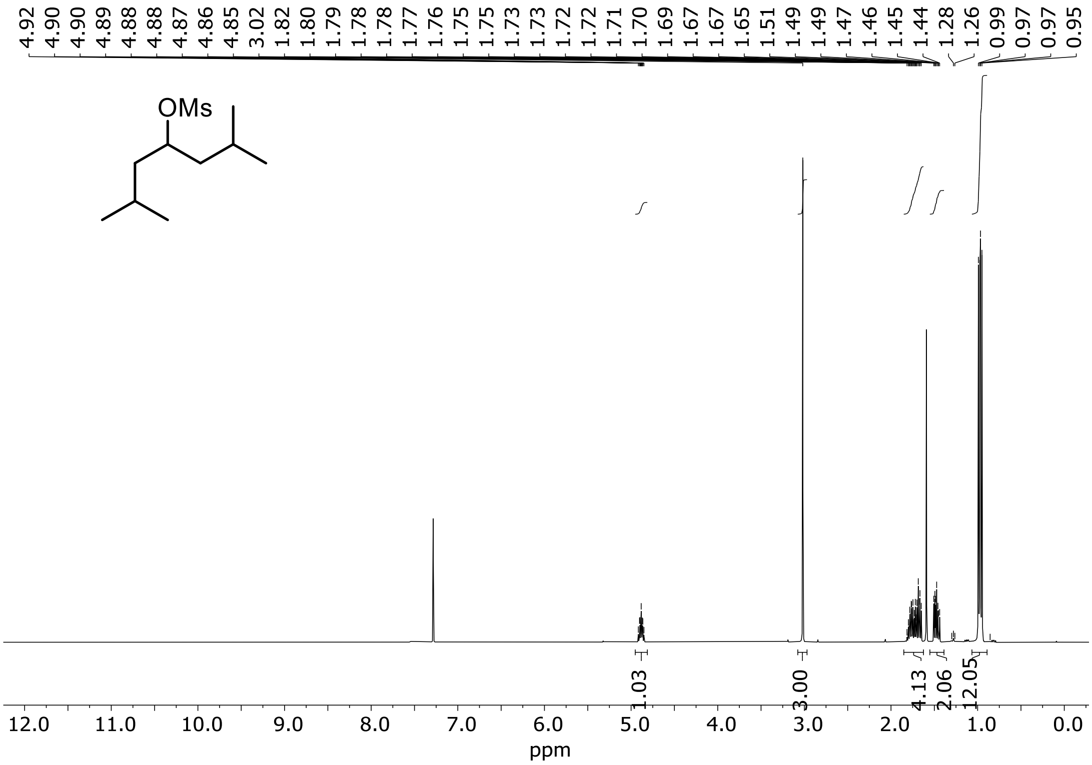

^{13}C-NMR of **10Ms** in $CDCl_3$

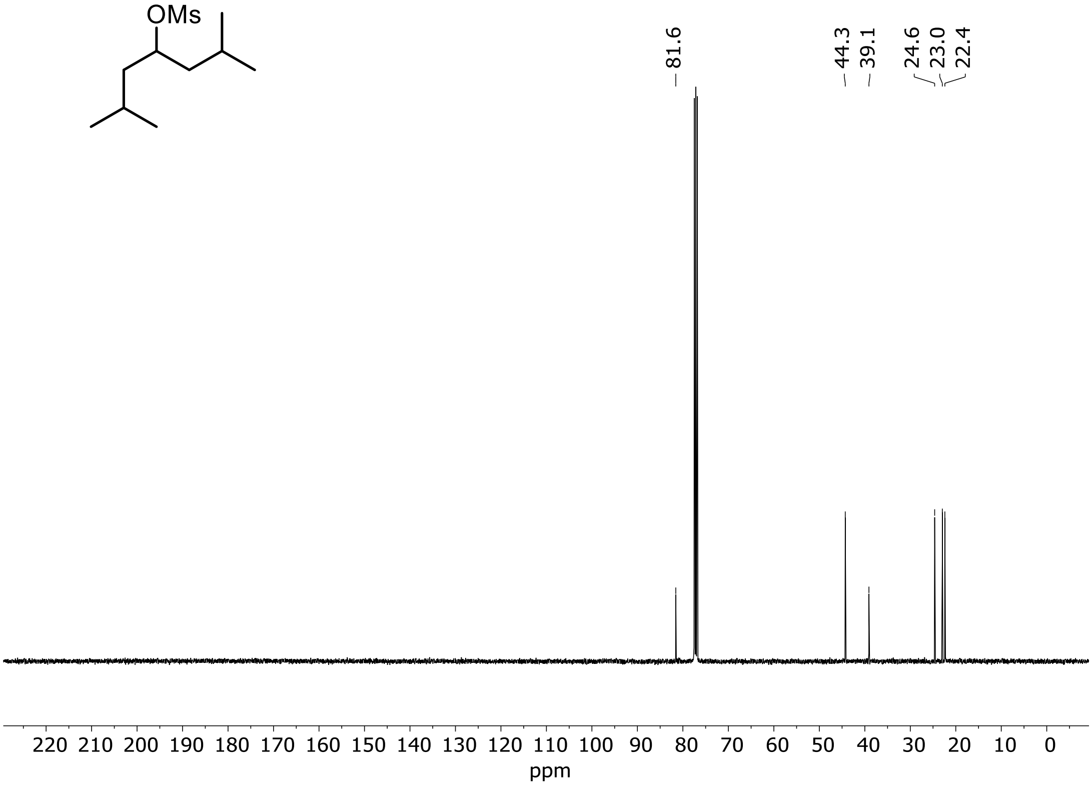

^{1}H-NMR of **10N$_3$** in $CDCl_3$

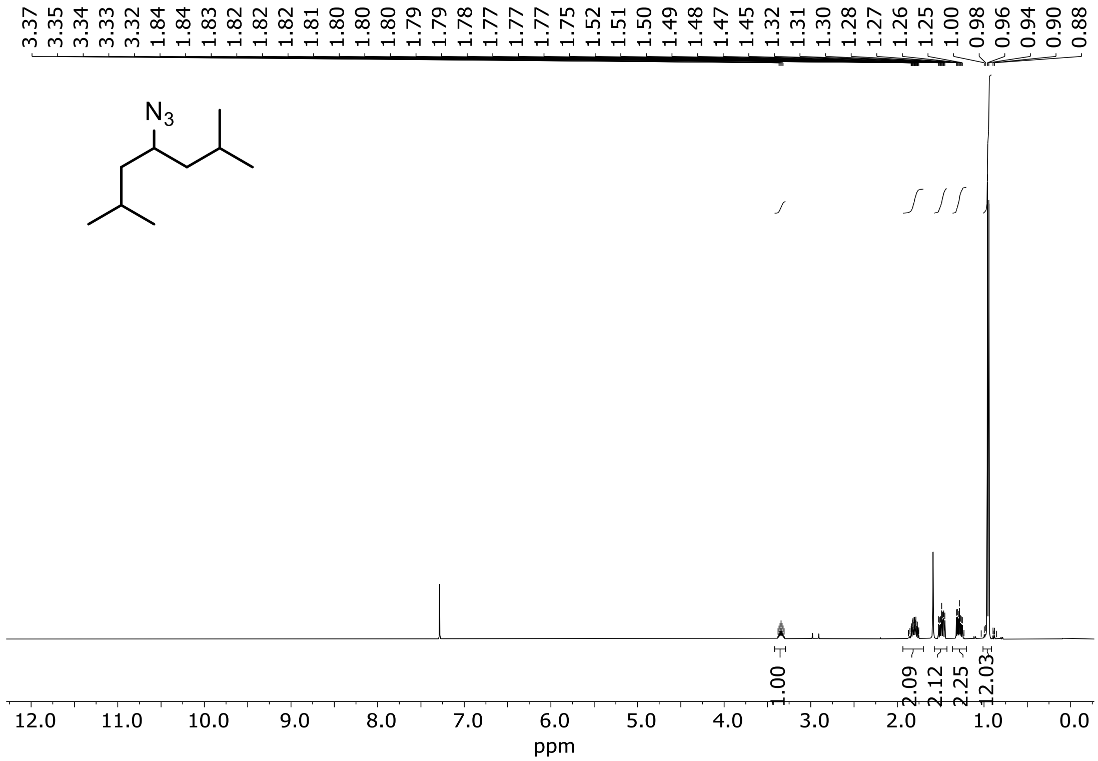

^{13}C-NMR of **10N$_3$** in $CDCl_3$

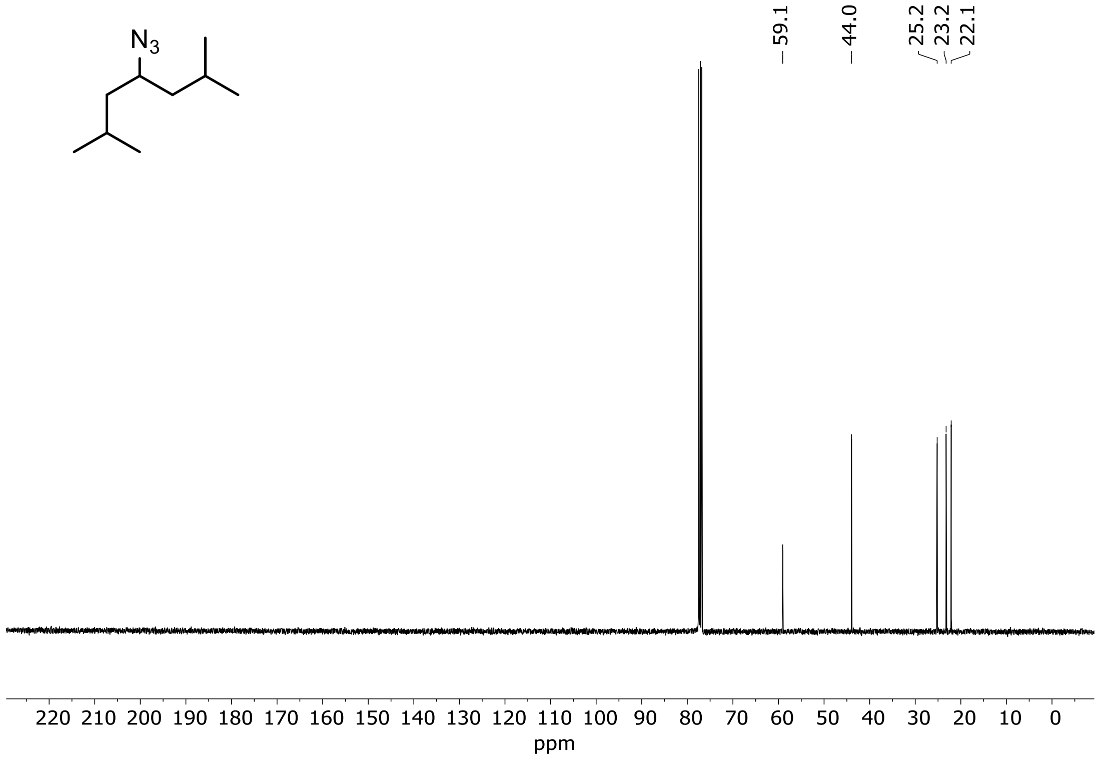

^{1}H-NMR of **15a** in $CDCl_3$

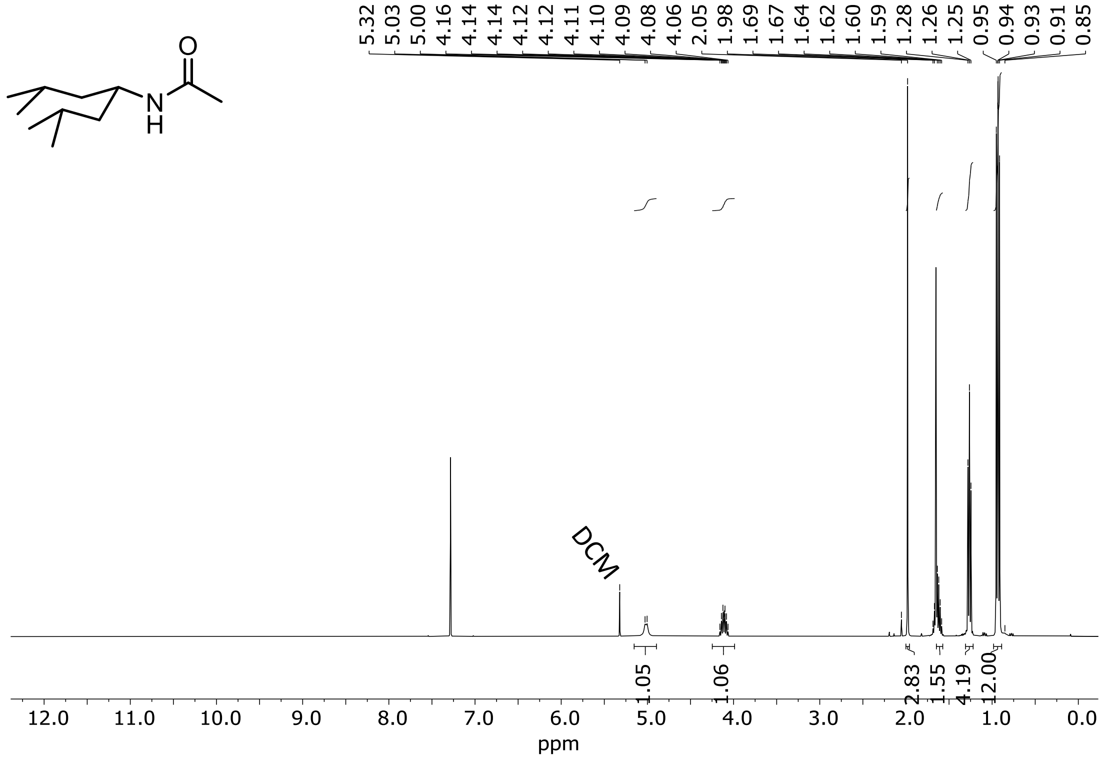

^{13}C-NMR of **15a** in $CDCl_3$

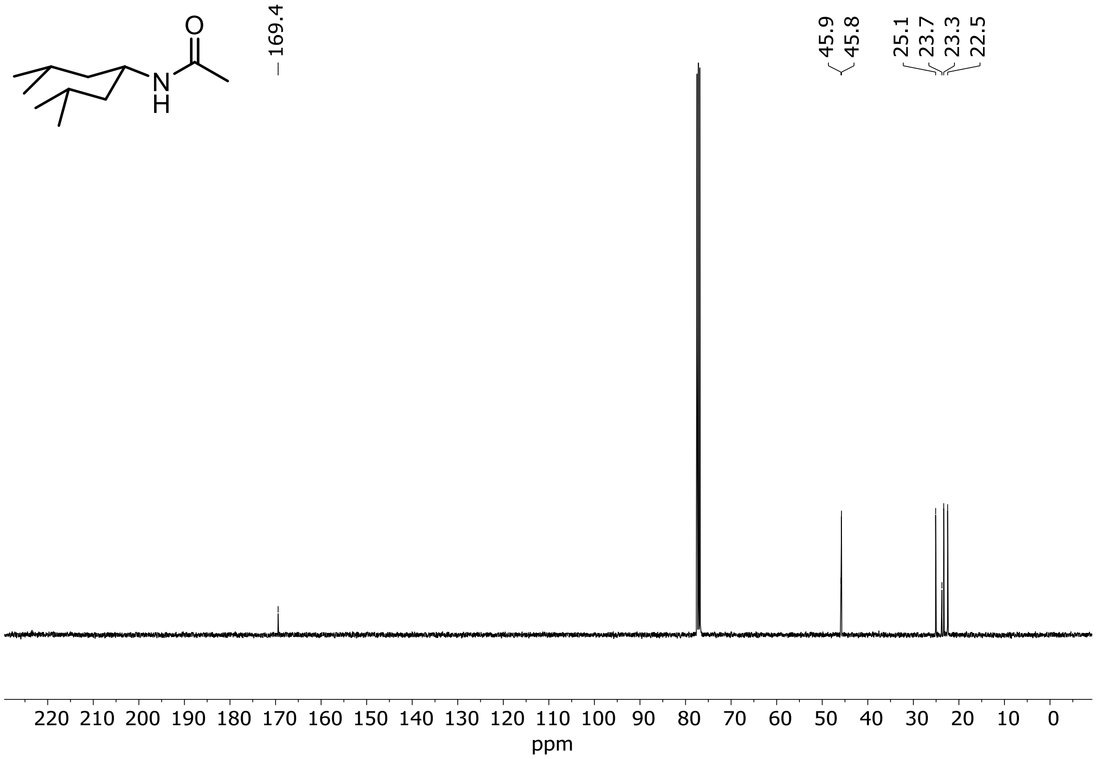

^{1}H-NMR of **17a** in $CDCl_3$

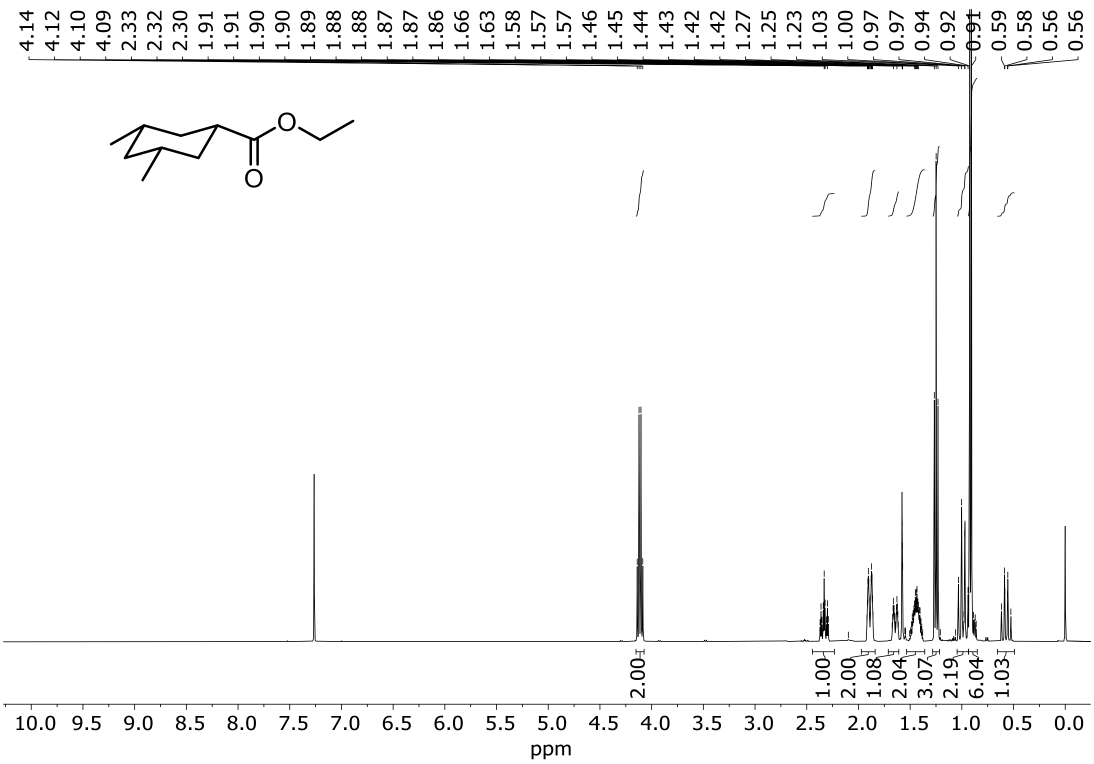

^{13}C-NMR of **17a** in $CDCl_3$

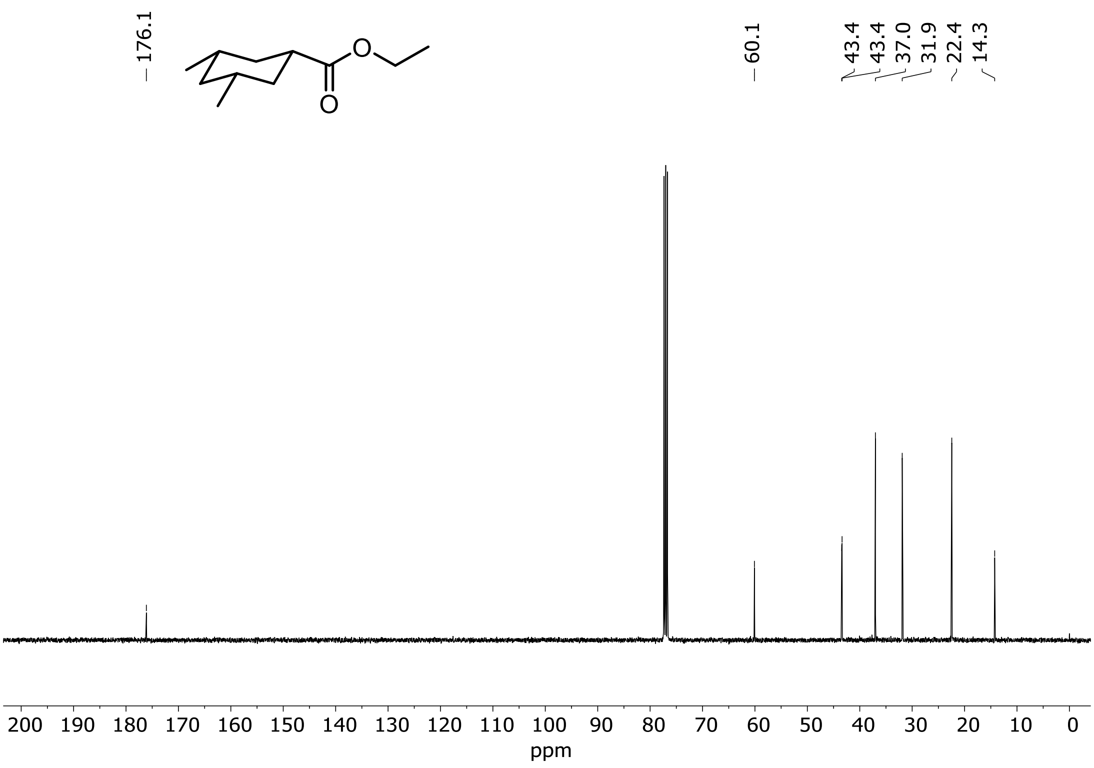

^{1}H-NMR of **C1** in $CDCl_3$

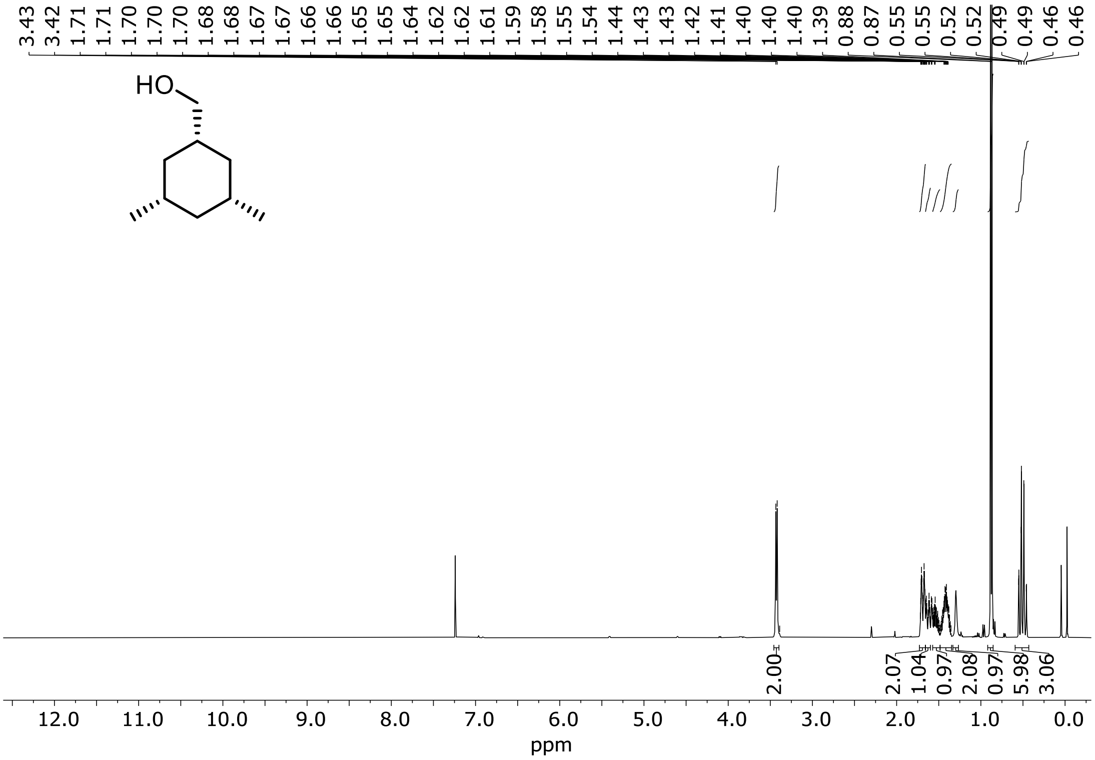

^{13}C-NMR of **C1** in $CDCl_3$

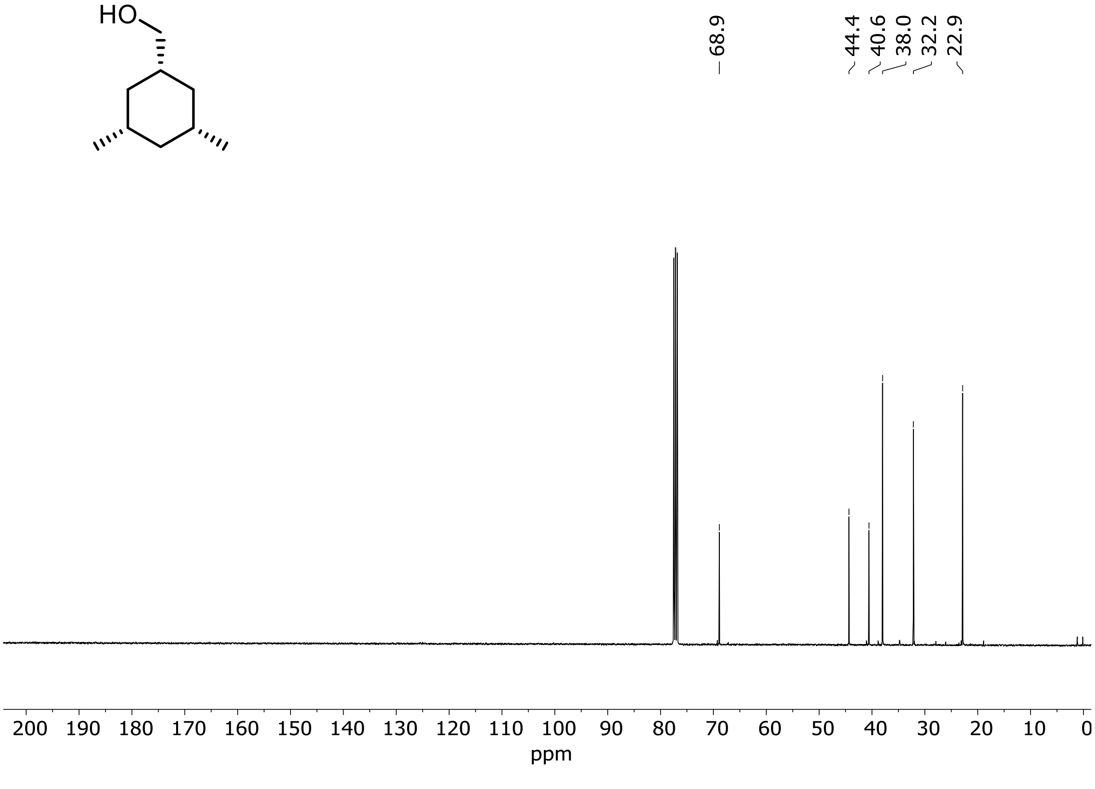

^{1}H-NMR of **C2** in $CDCl_3$

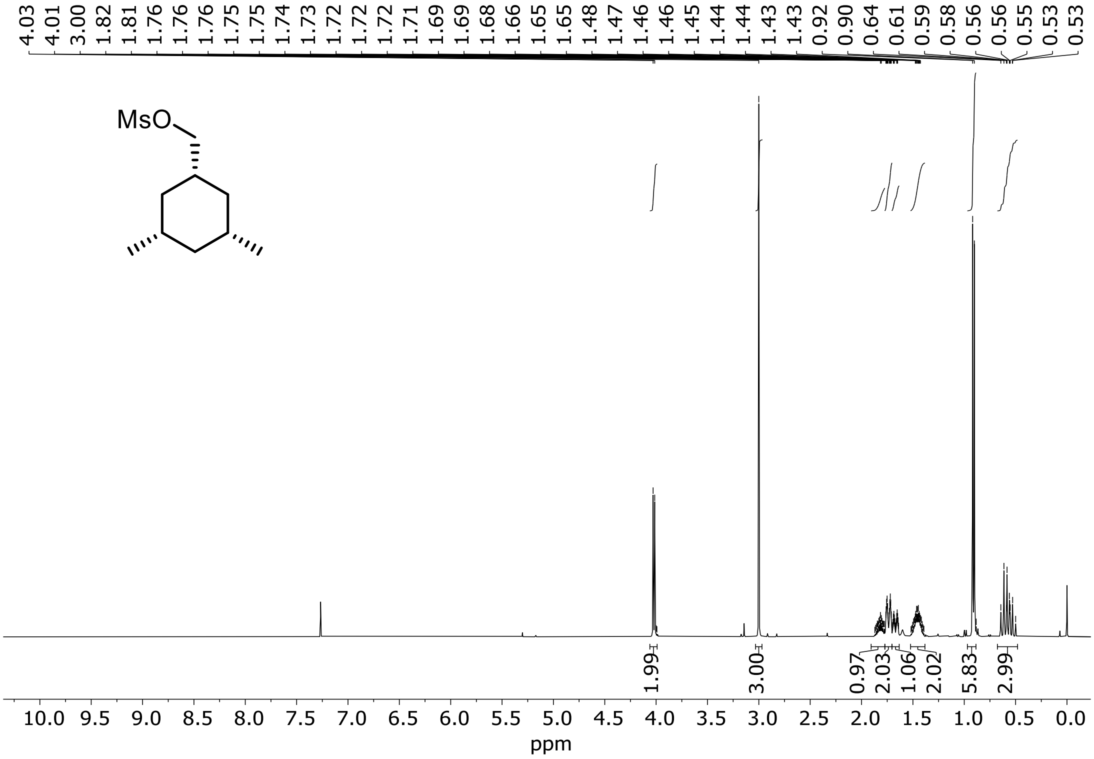

^{13}C-NMR of **C2** in $CDCl_3$

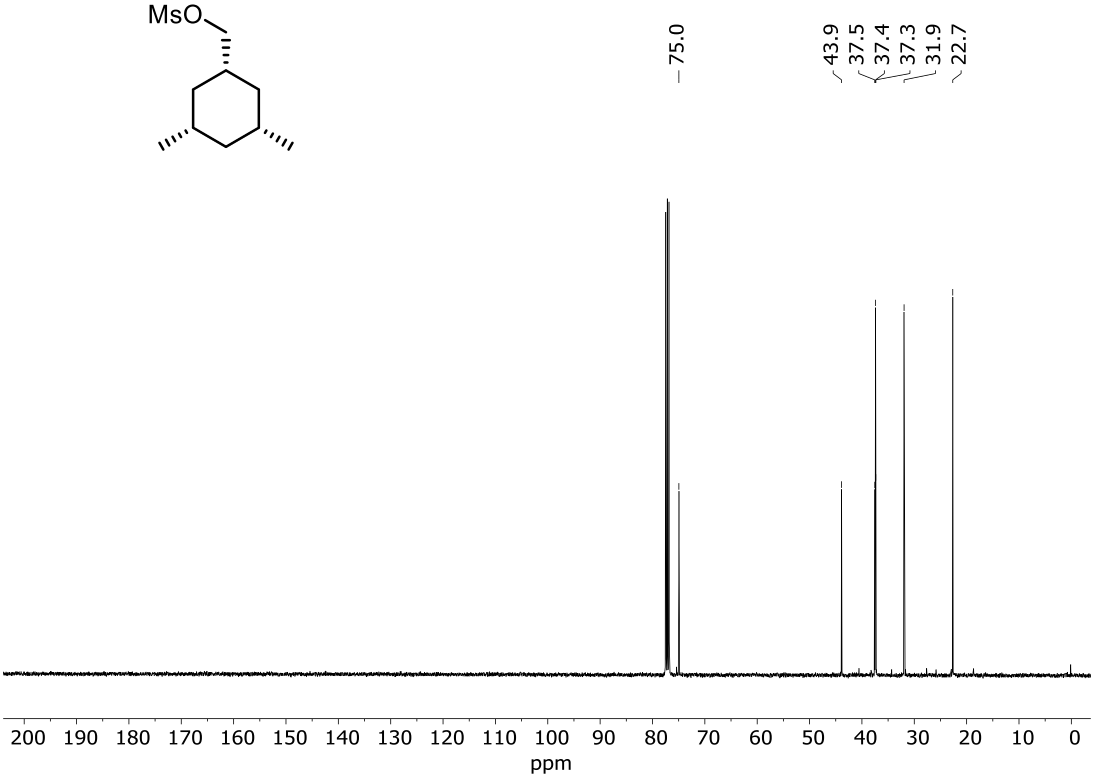

^{1}H-NMR of **21a** in $CDCl_3$

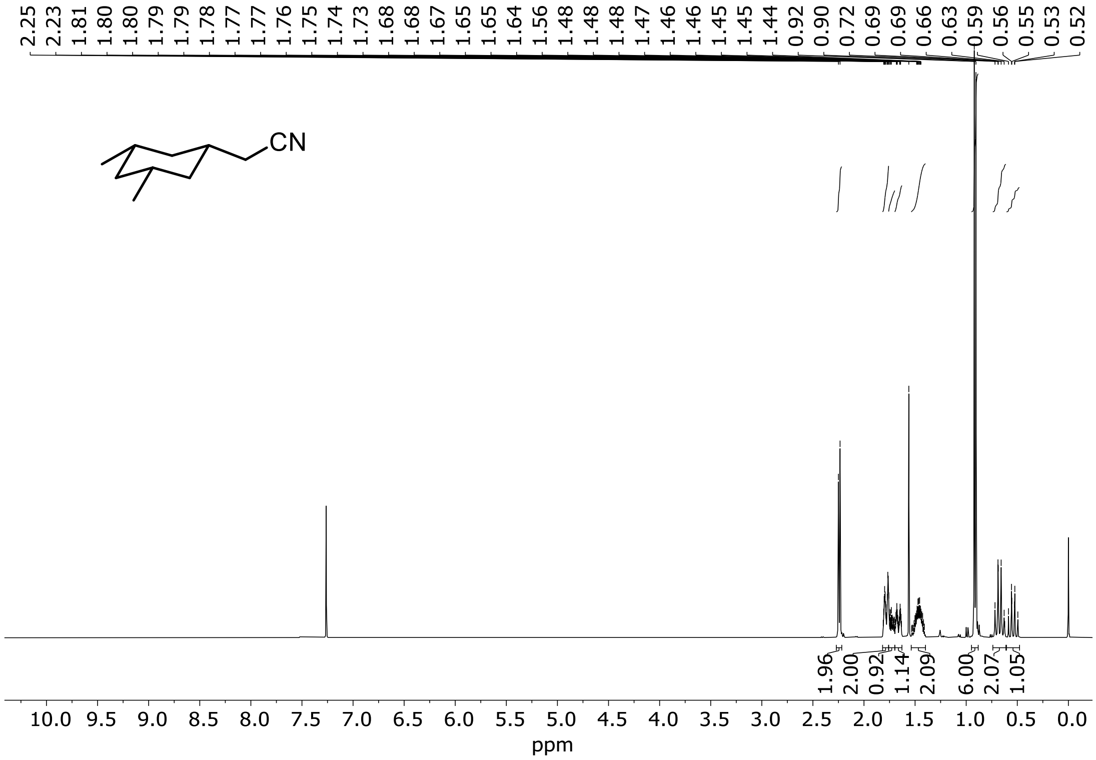

^{13}C-NMR of **21a** in $CDCl_3$

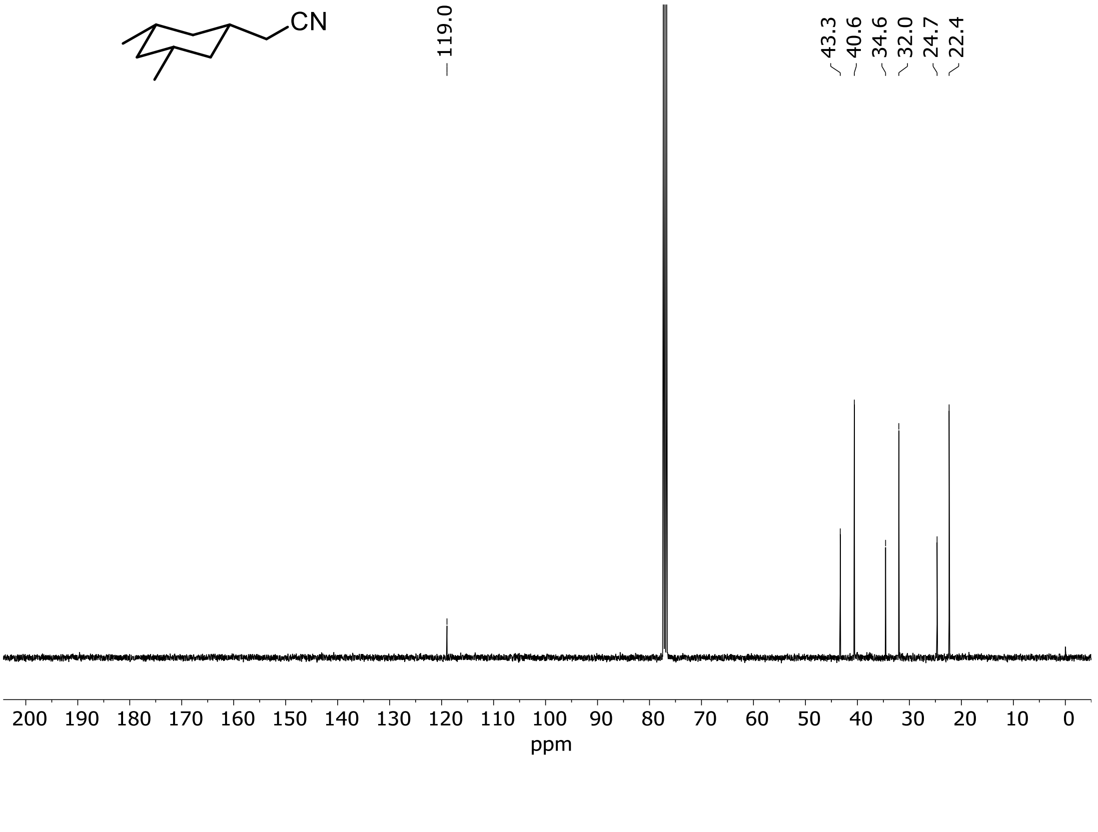

^{1}H-NMR of **22a** in $CDCl_3$

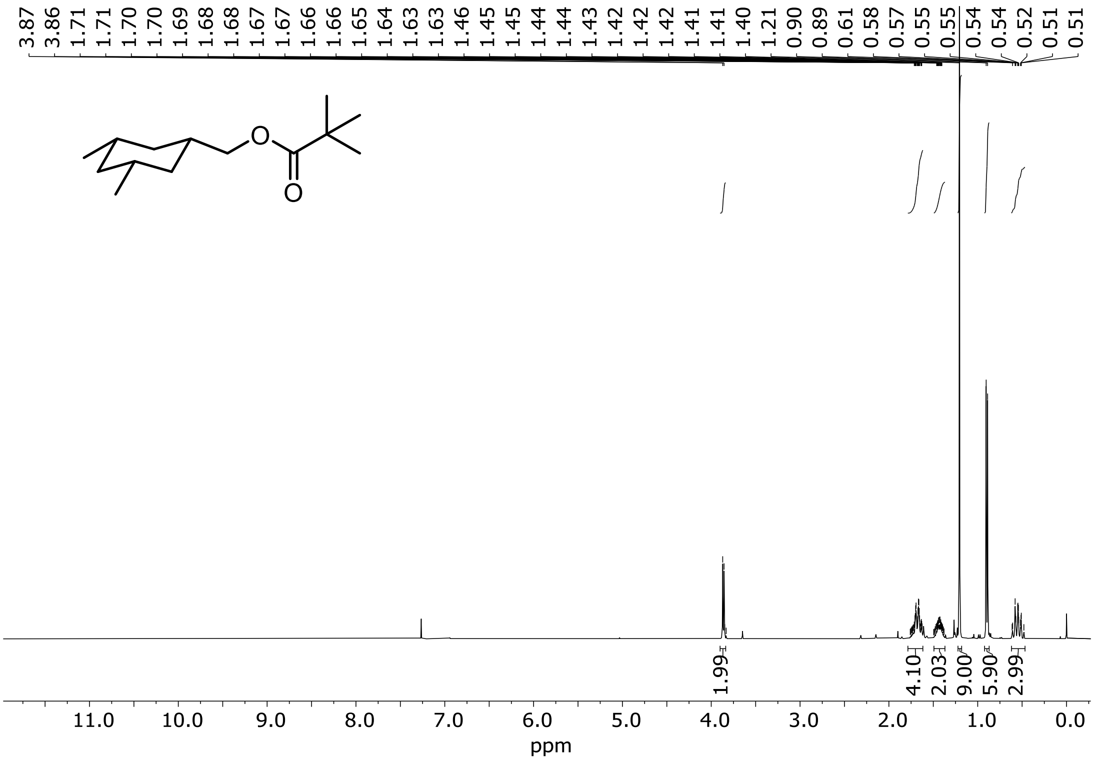

^{13}C-NMR of **22a** in $CDCl_3$

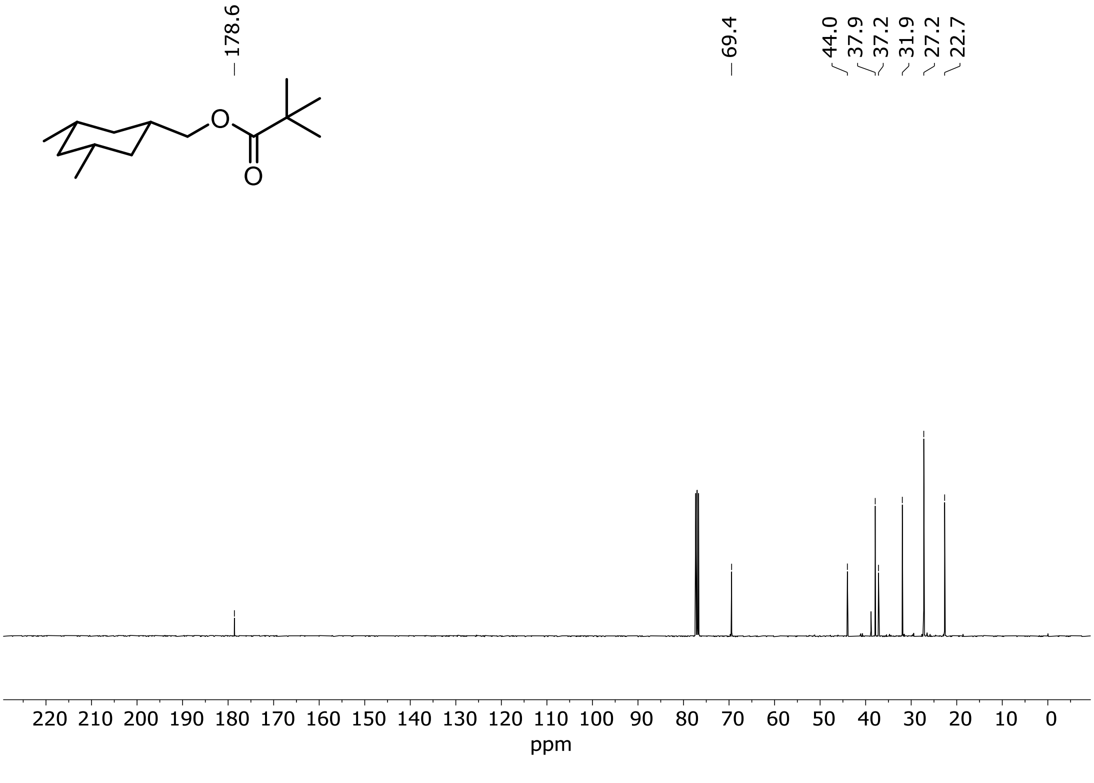

^{1}H-NMR of **23a** in $CDCl_3$

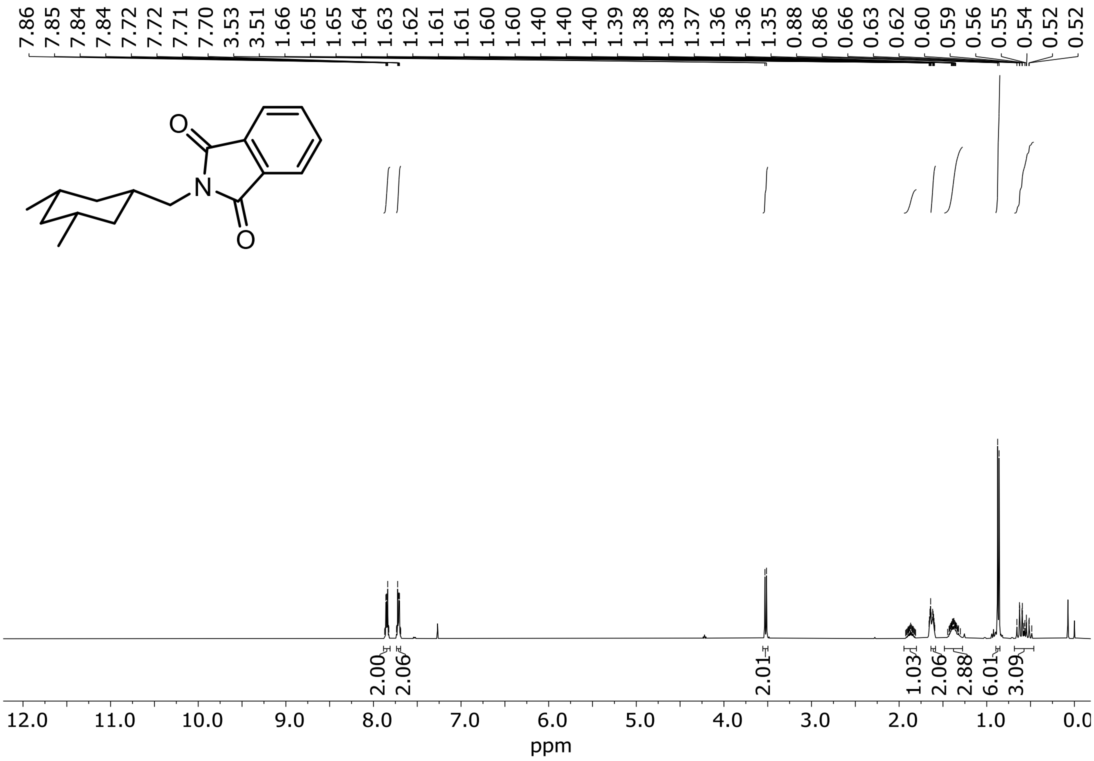

^{13}C-NMR of **23a** in $CDCl_3$

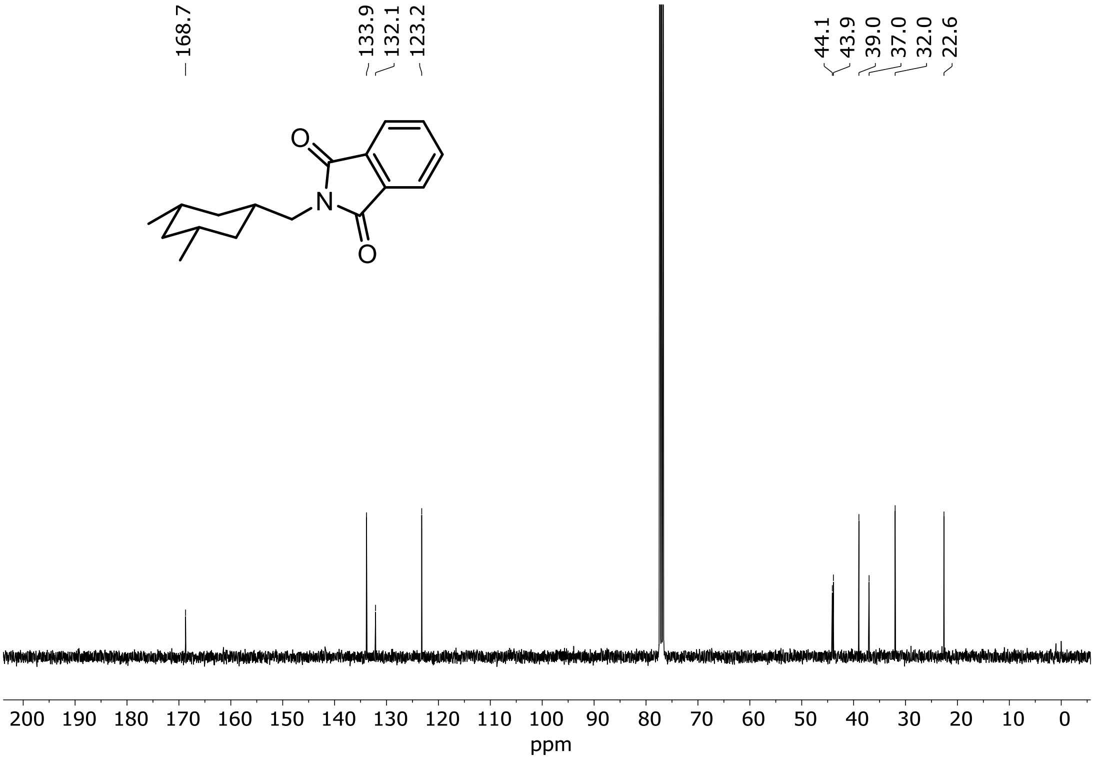

^{1}H-NMR of **19a** in $CDCl_3$

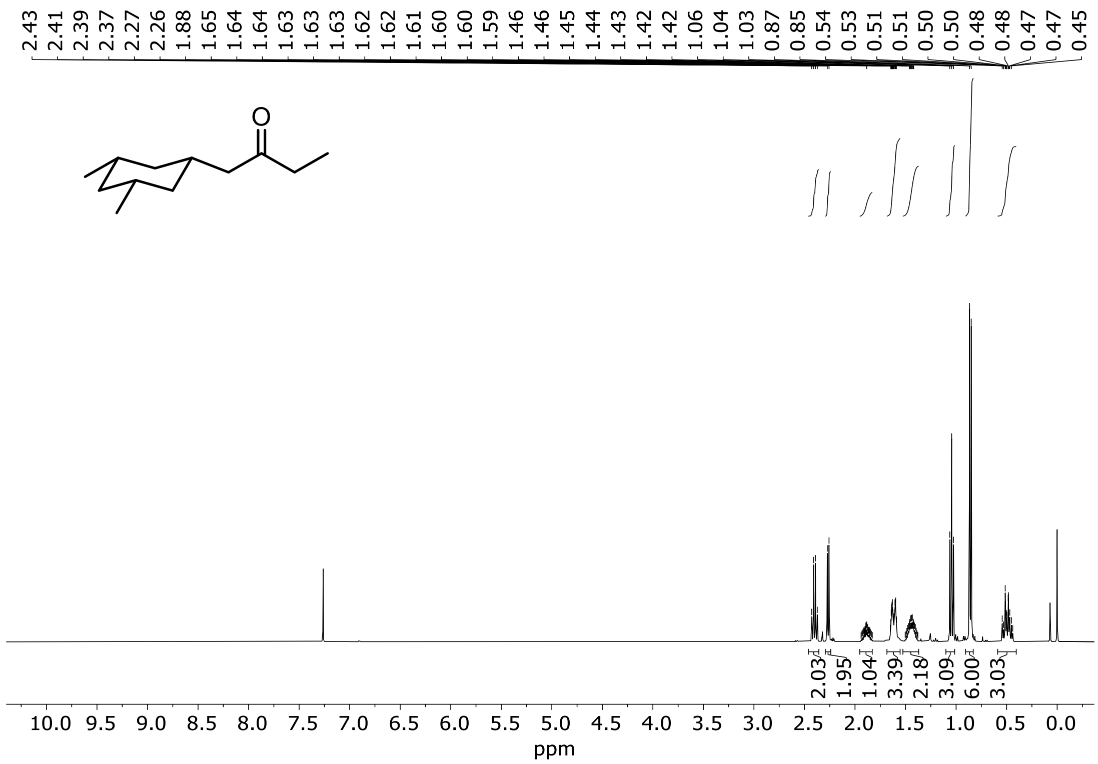

^{13}C-NMR of **19a** in $CDCl_3$

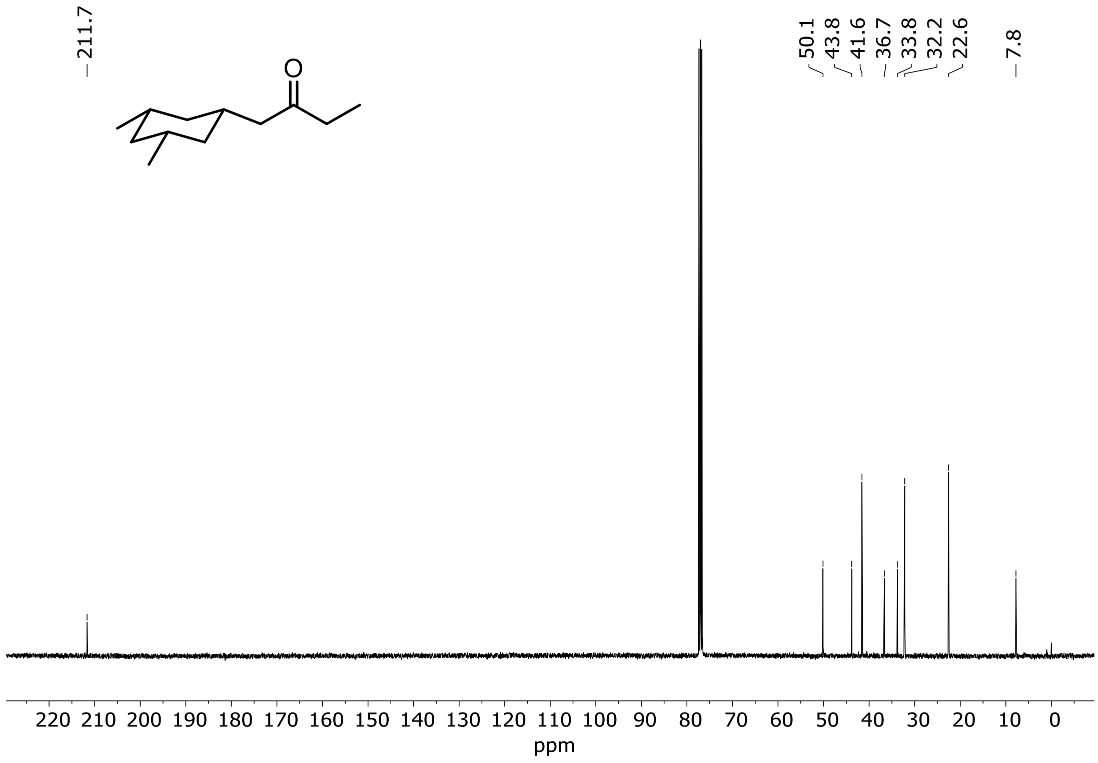

^{1}H-NMR of **C3** in $CDCl_3$

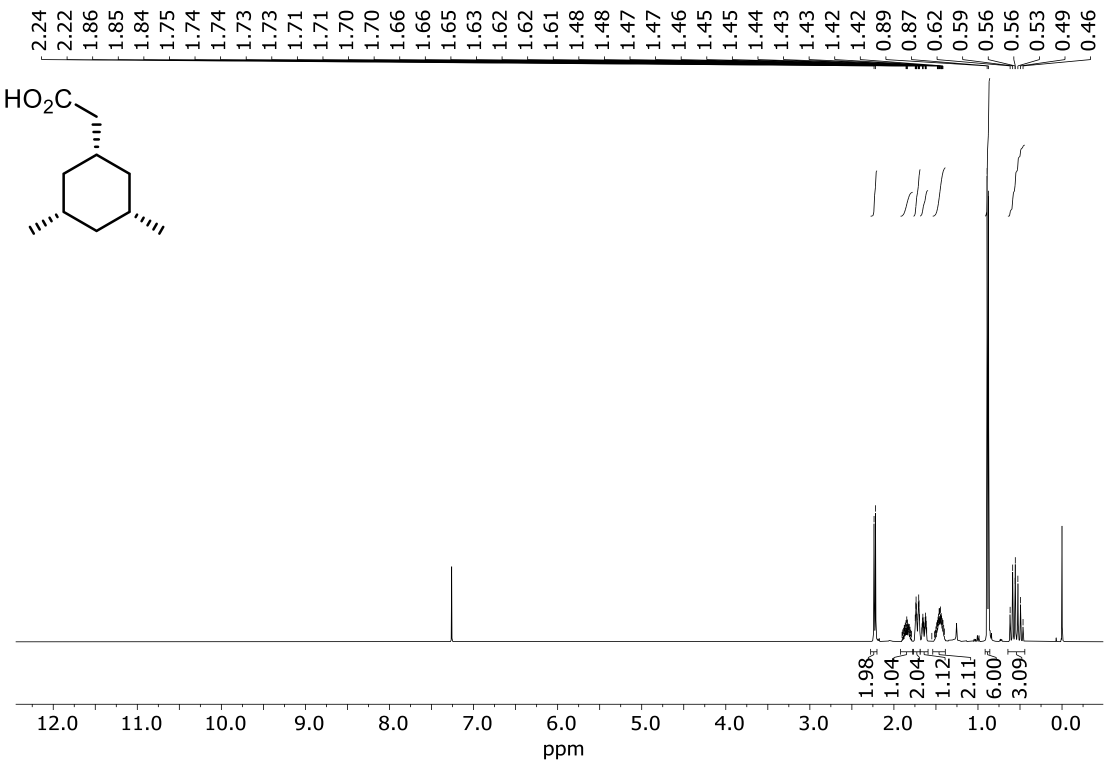

^{13}C-NMR of **C3** in $CDCl_3$

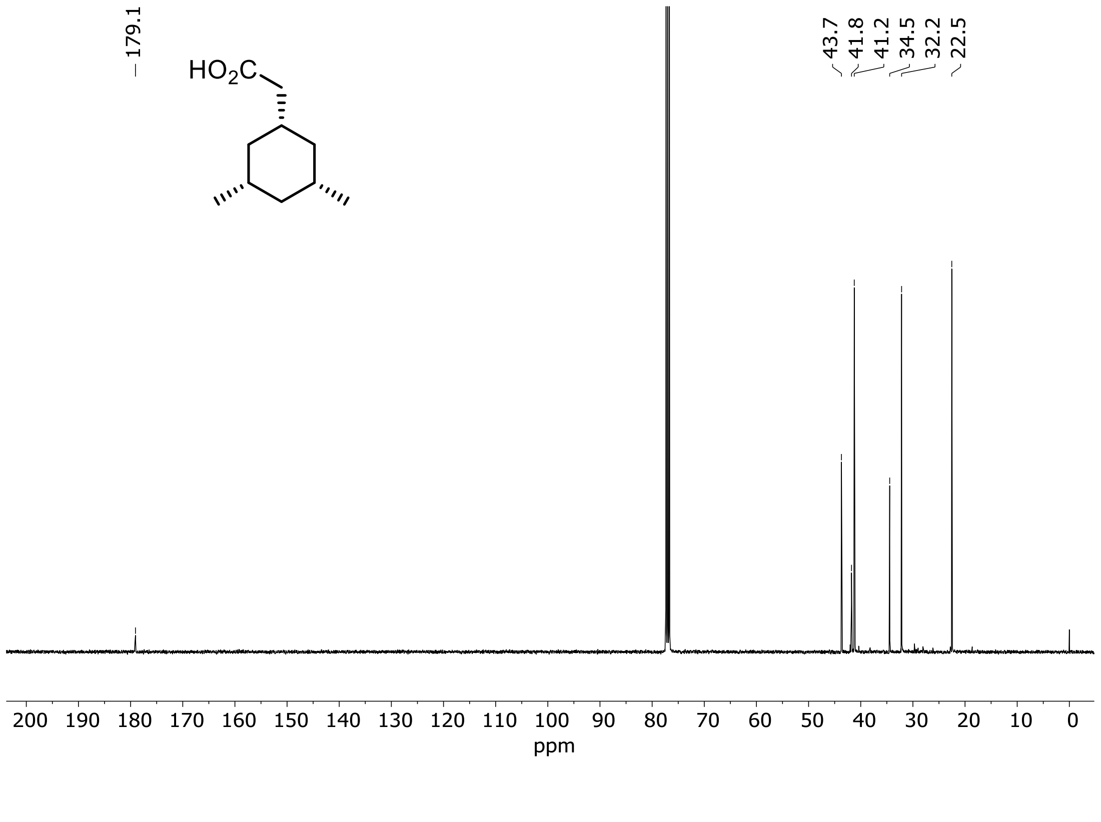

^{1}H-NMR of **18a** in $CDCl_3$

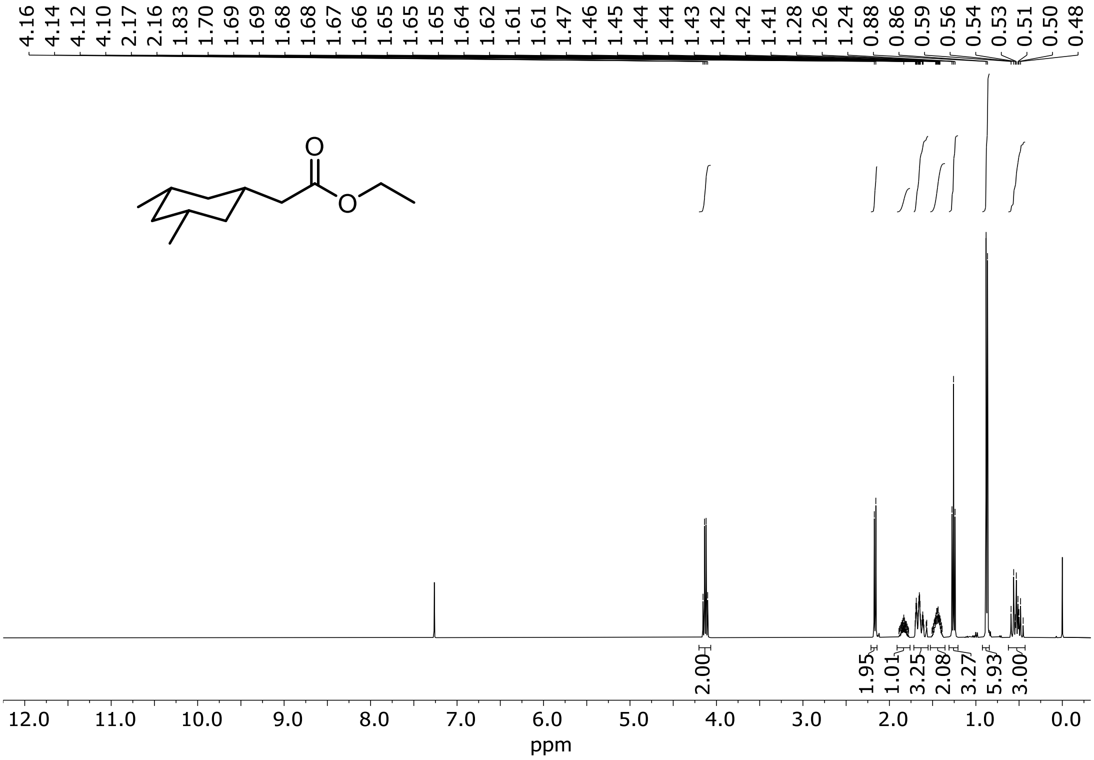

^{13}C-NMR of **18a** in $CDCl_3$

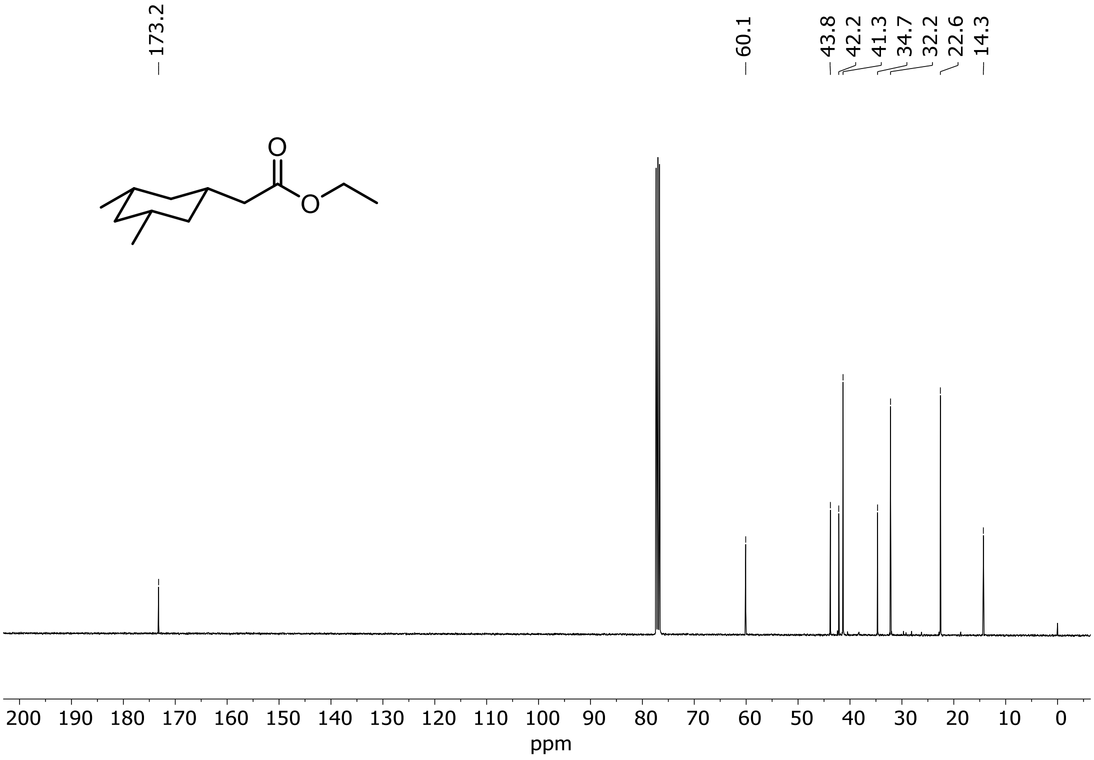

^{1}H-NMR of **20a** in $CDCl_3$

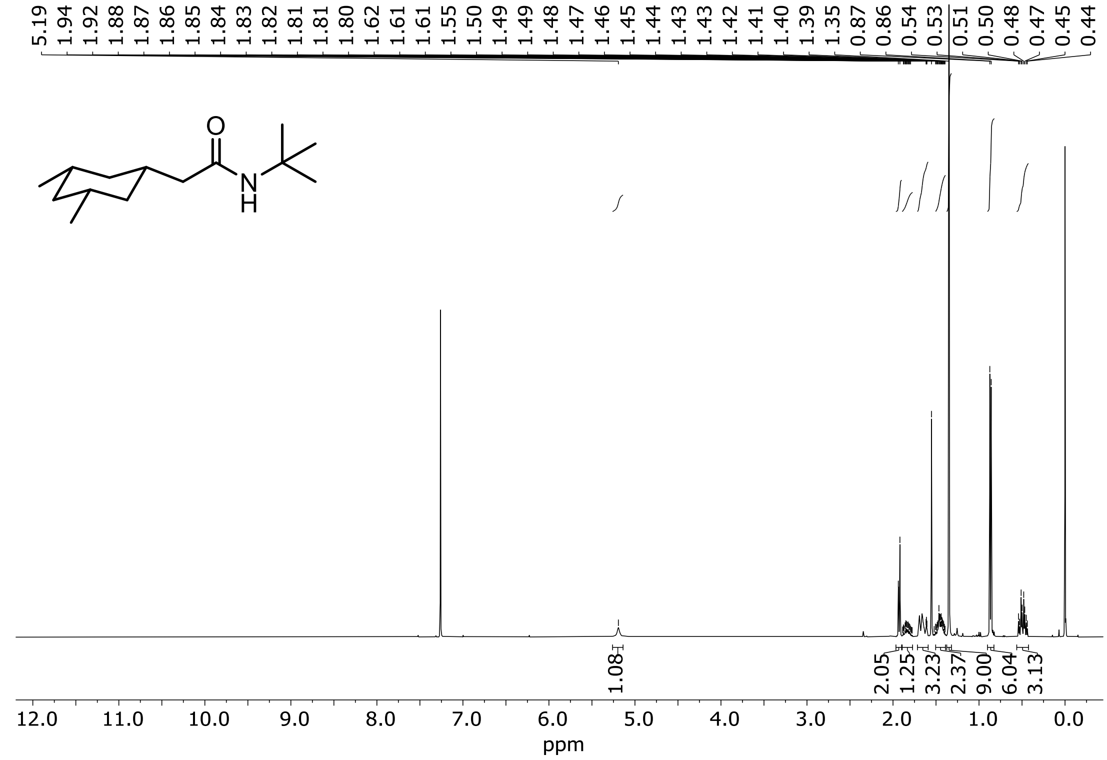

^{13}C-NMR of **20a** in $CDCl_3$

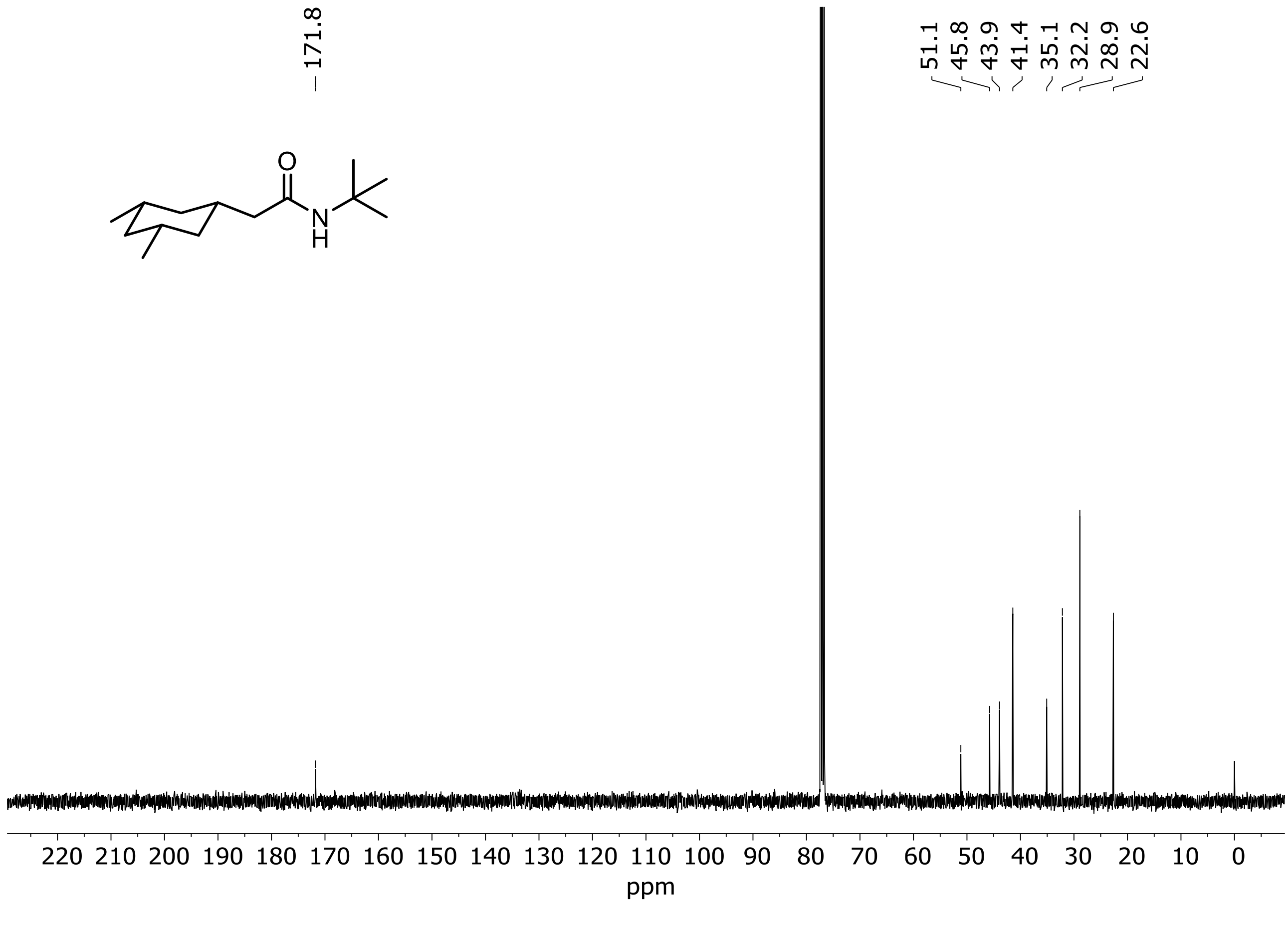

^{1}H-NMR of **24a** in $CDCl_3$

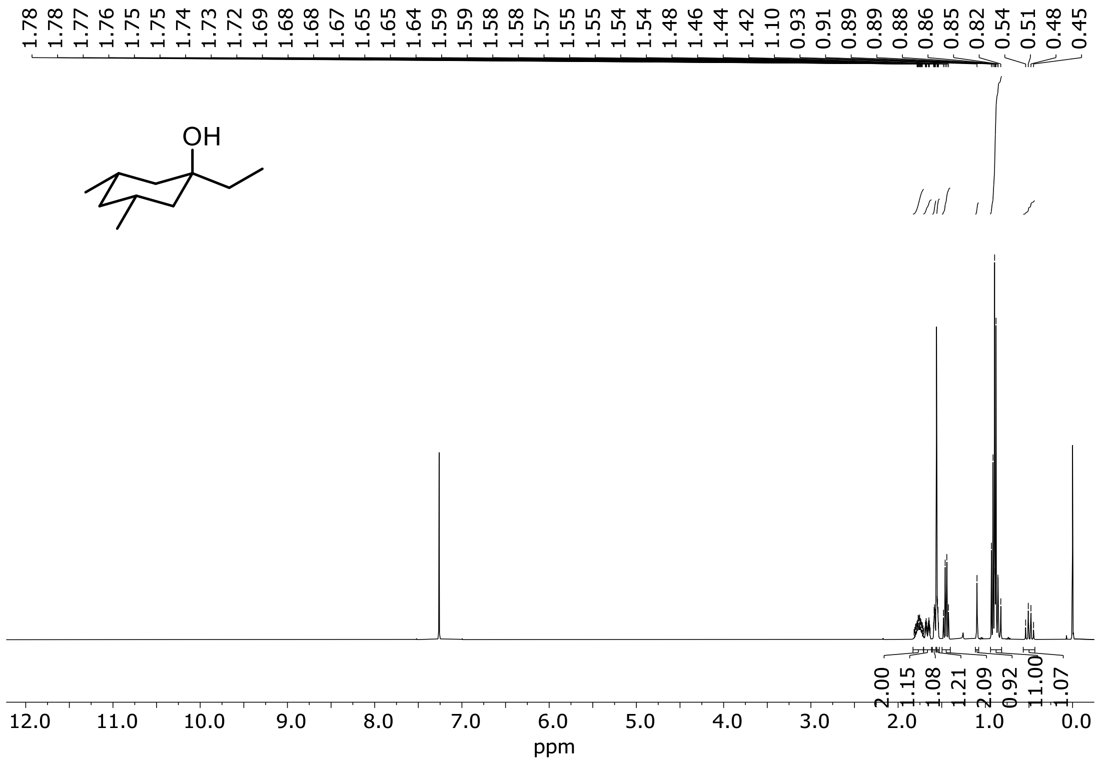

^{13}C-NMR of **24a** in $CDCl_3$

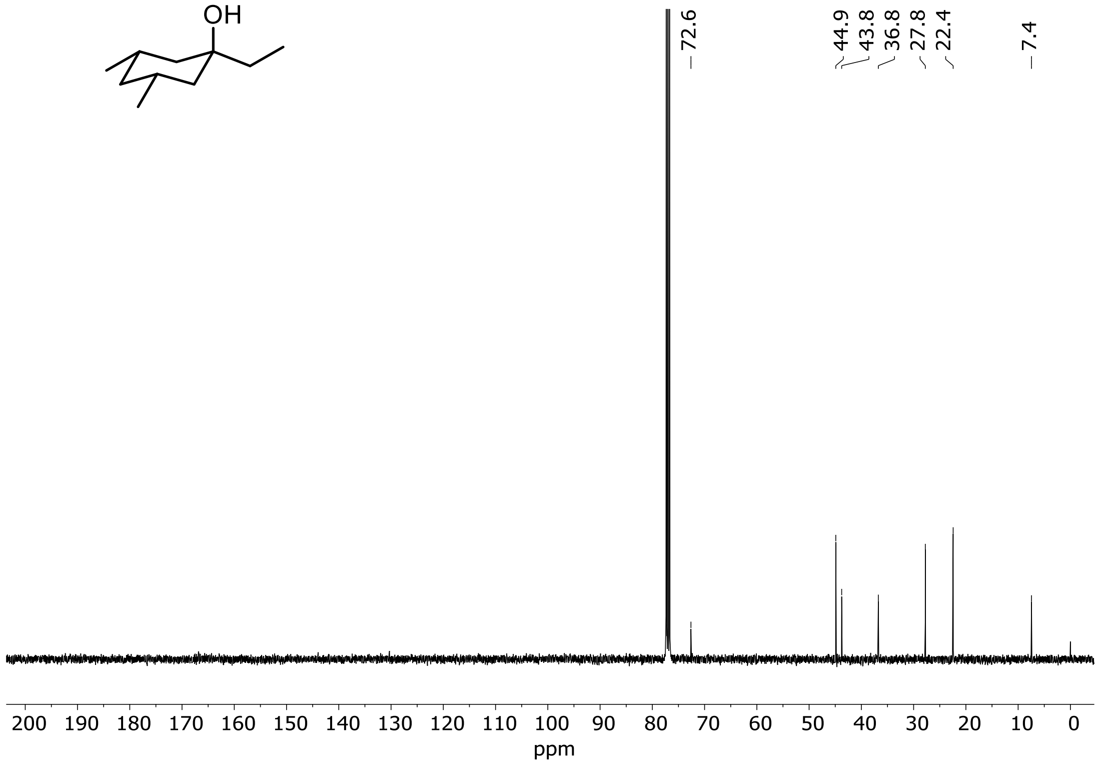

^{1}H-NMR of **25a** in $CDCl_3$

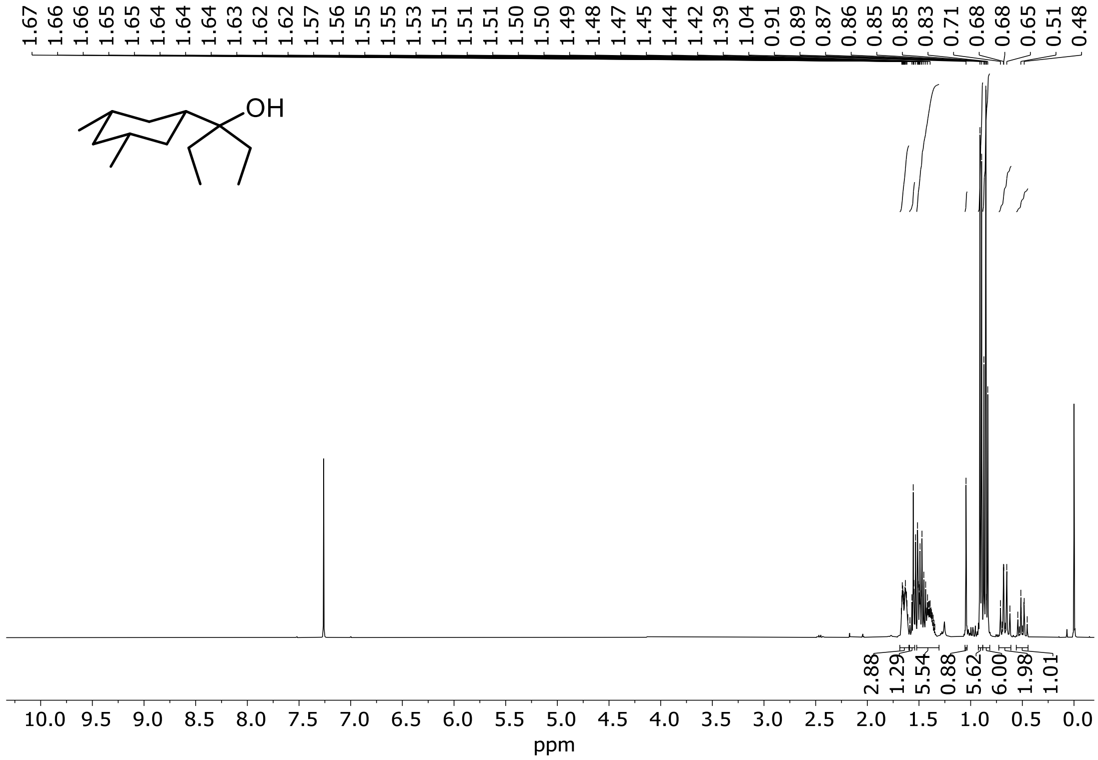

^{13}C-NMR of **25a** in $CDCl_3$

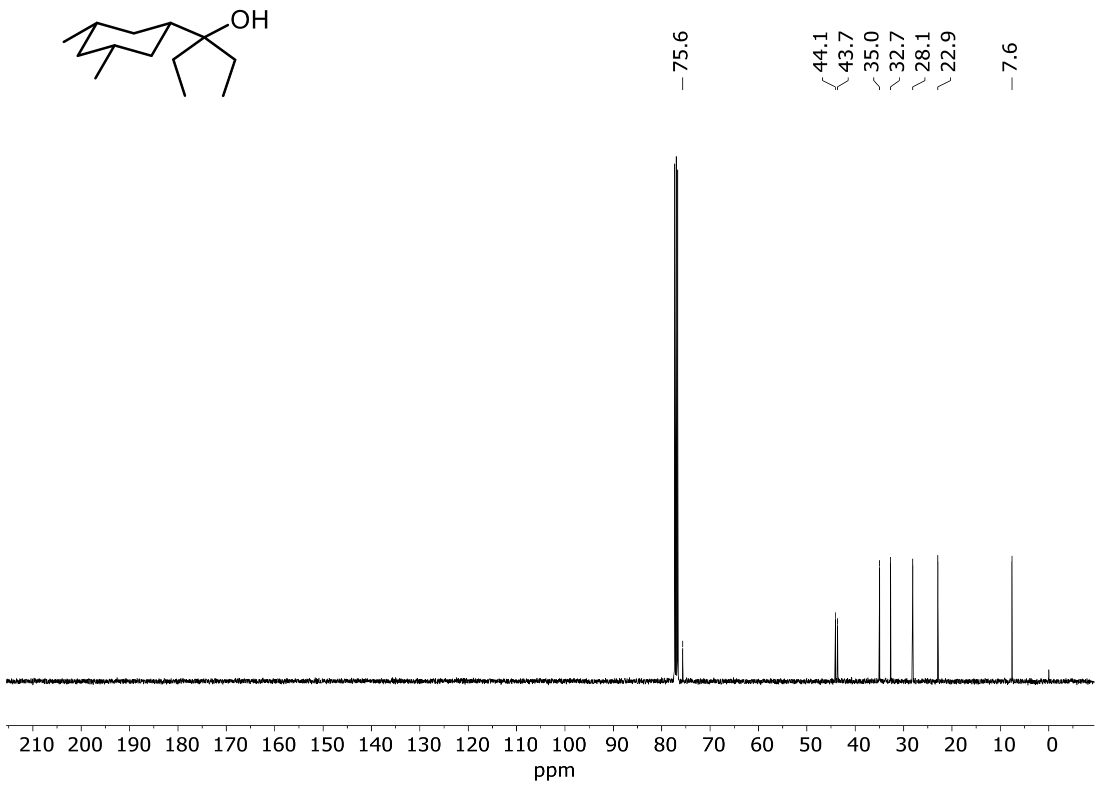

^{1}H-NMR of **26a** in $CDCl_3$

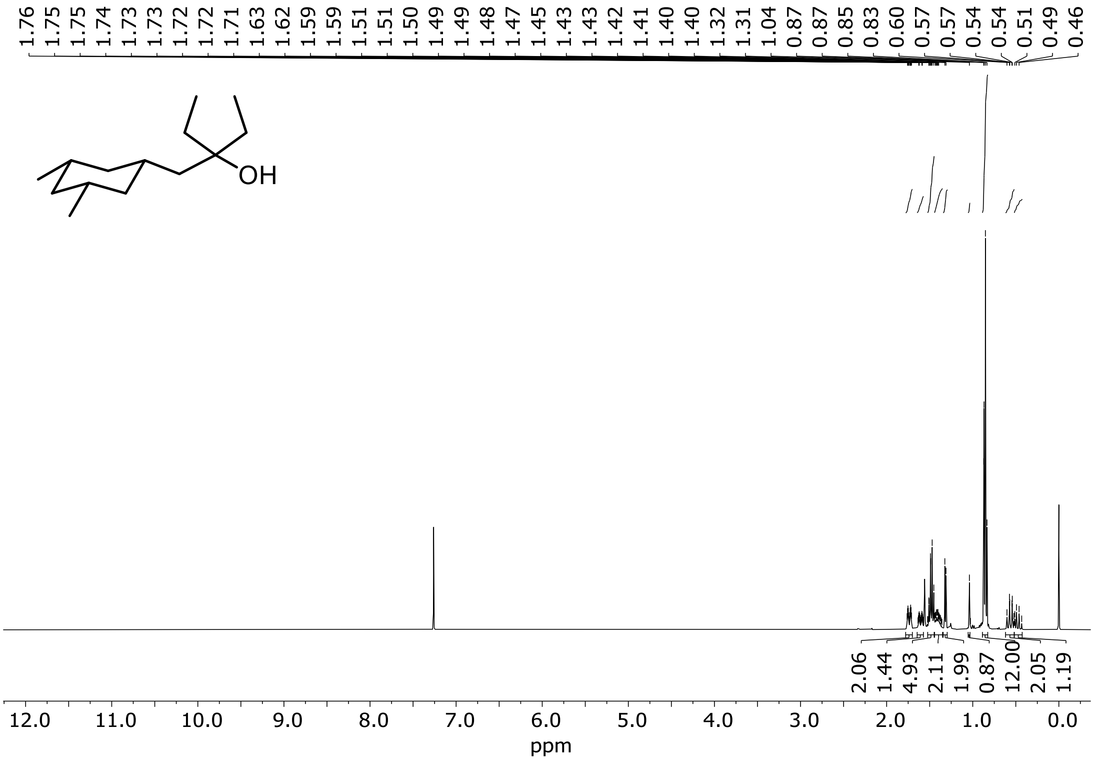

^{13}C-NMR of **26a** in $CDCl_3$

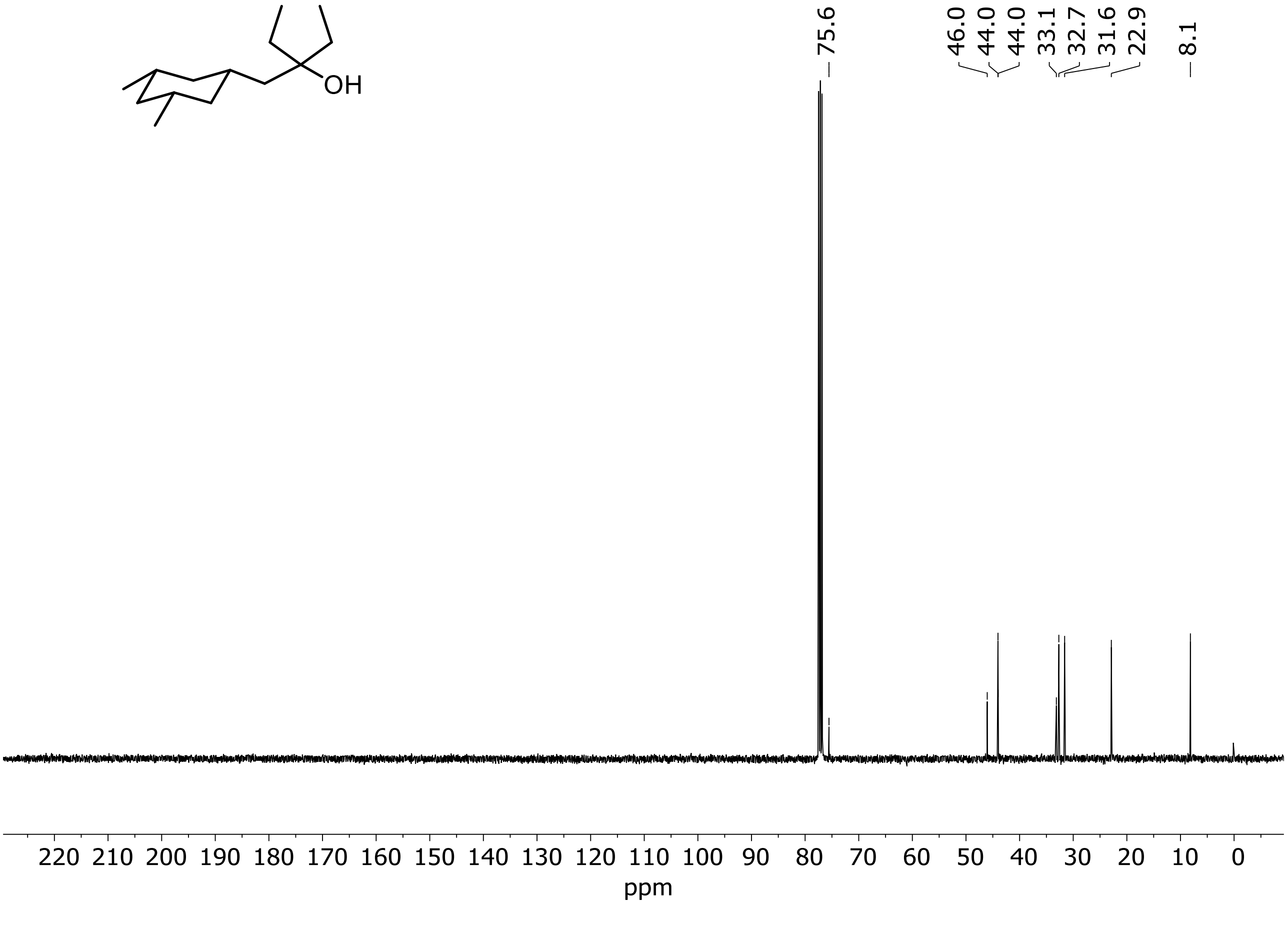

^{1}H-NMR of **28a** in $CDCl_3$

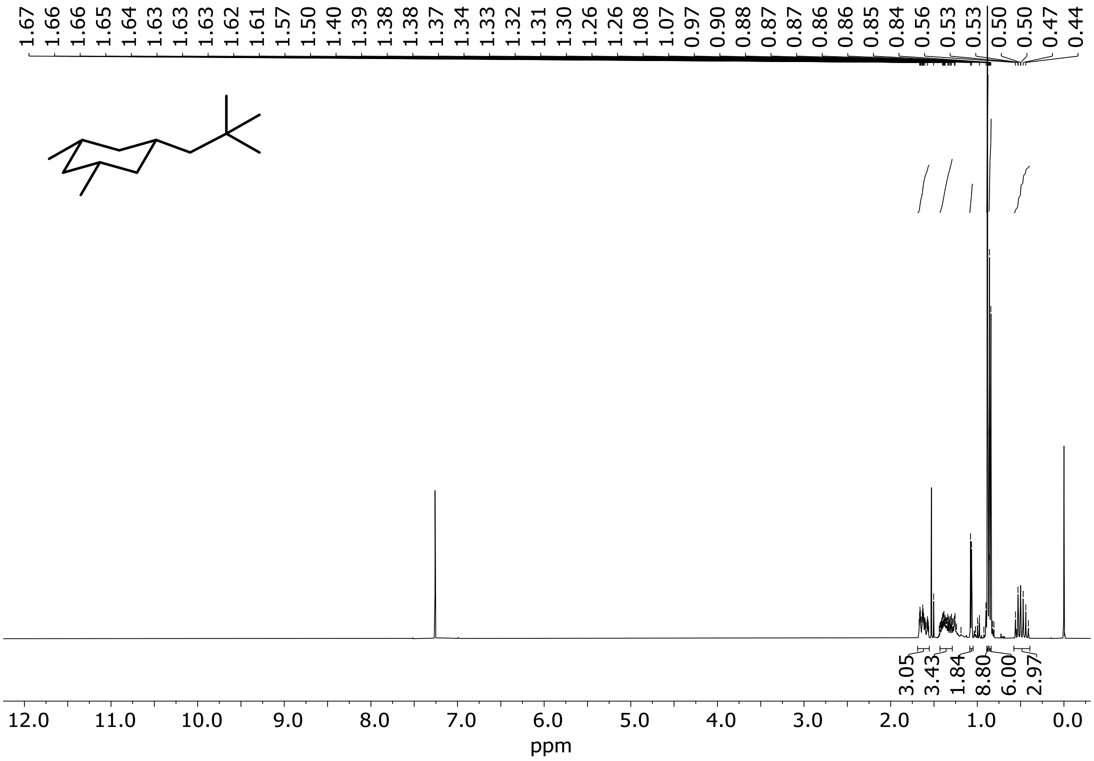

^{13}C-NMR of **28a** in $CDCl_3$

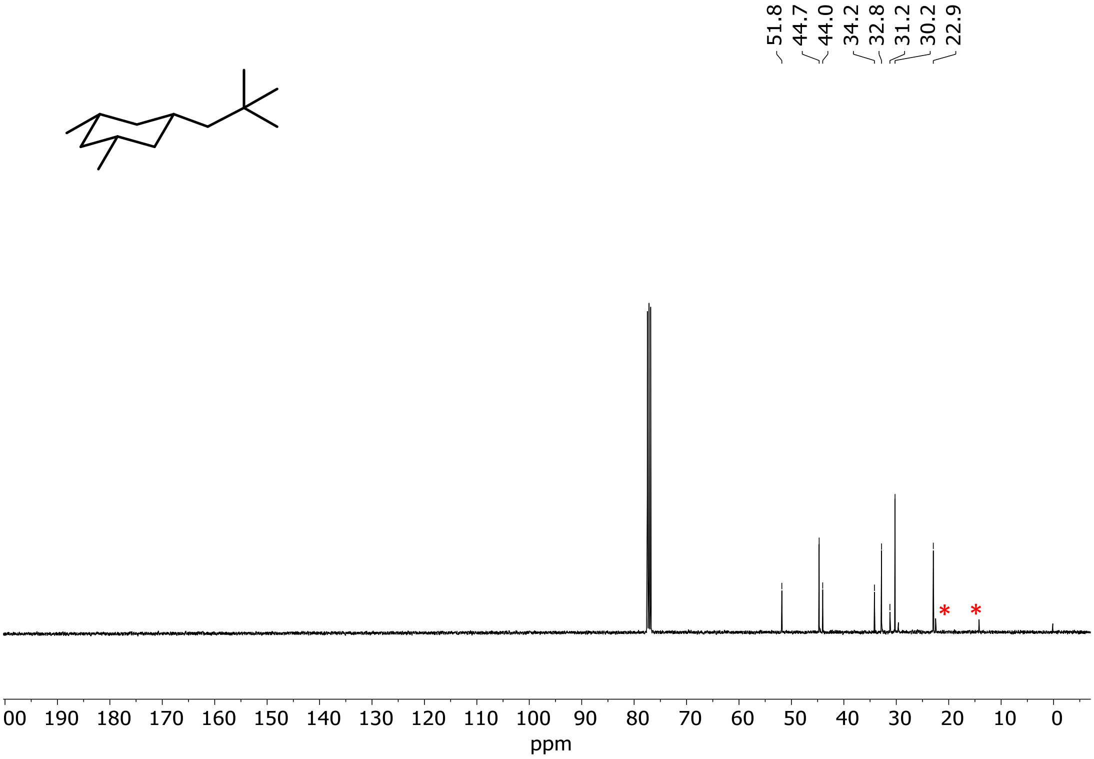

* Pentane

^{1}H-NMR of **29a** in $CDCl_3$

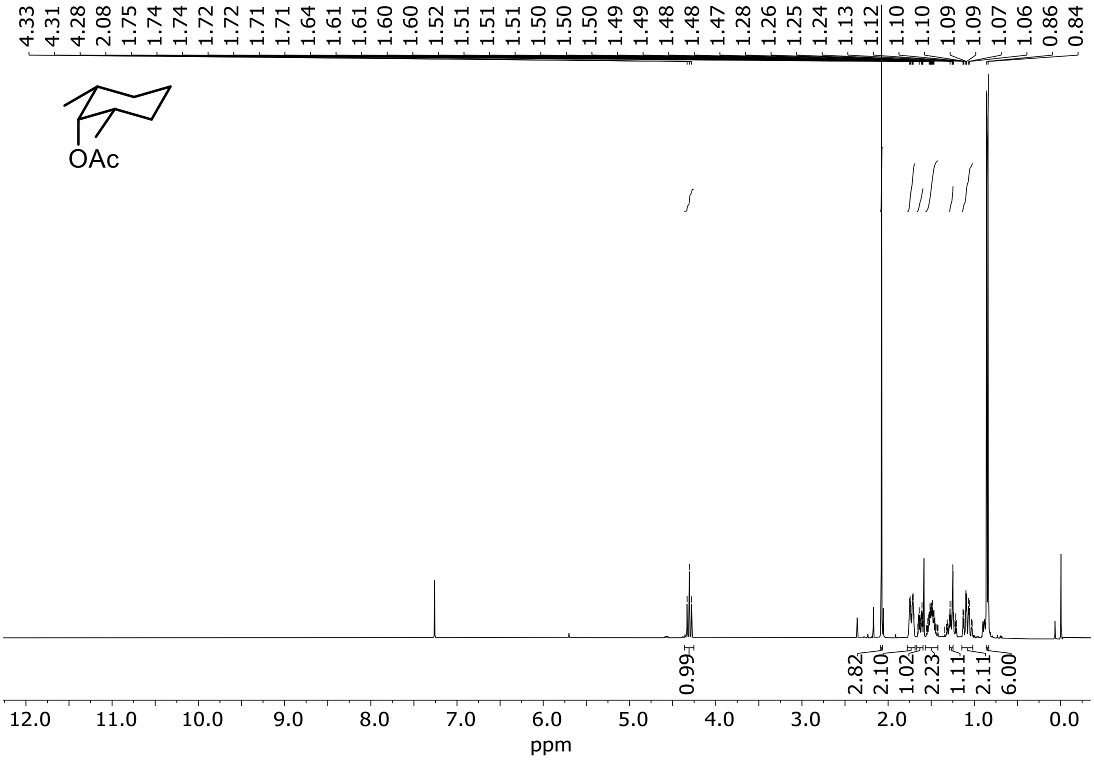

^{13}C-NMR of **29a** in $CDCl_3$

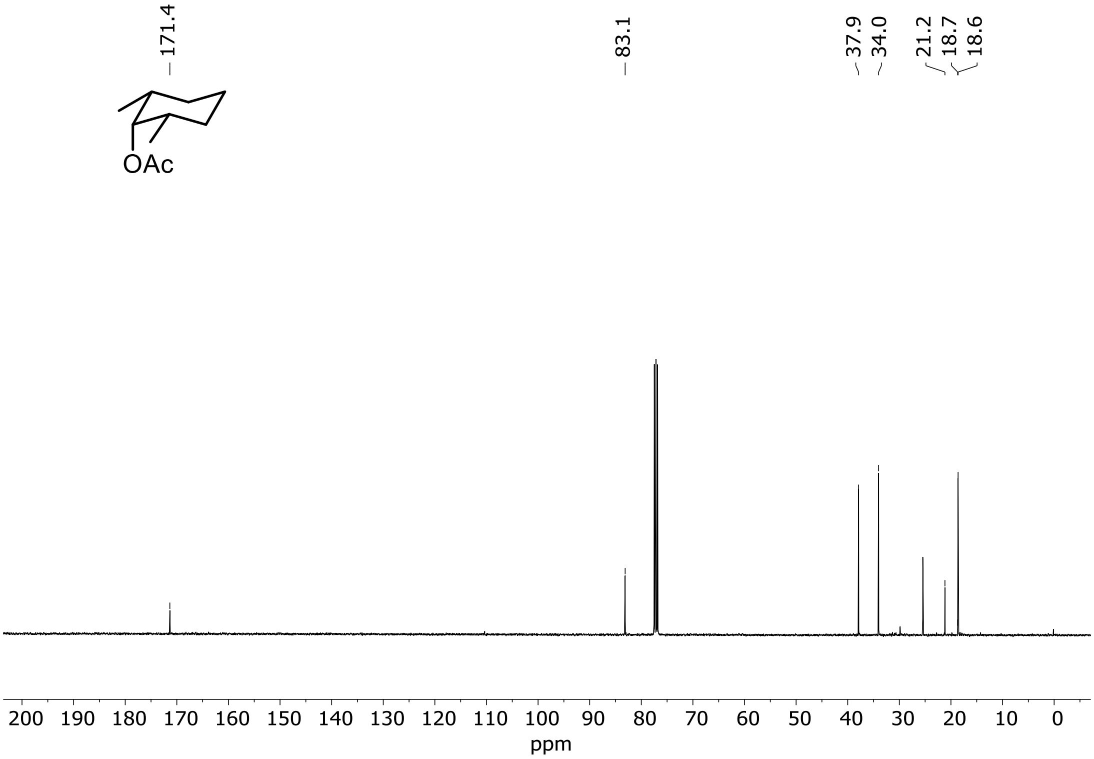

^{1}H-NMR of **30a** in $CDCl_3$

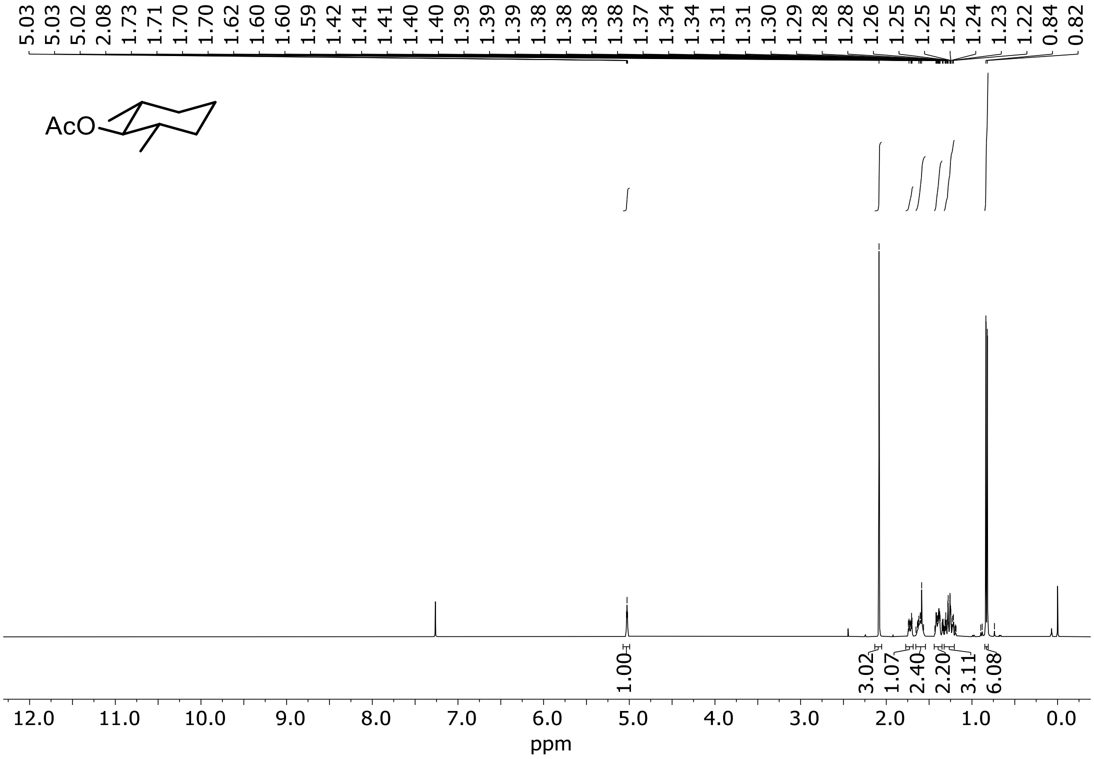

^{13}C-NMR of **30a** in $CDCl_3$

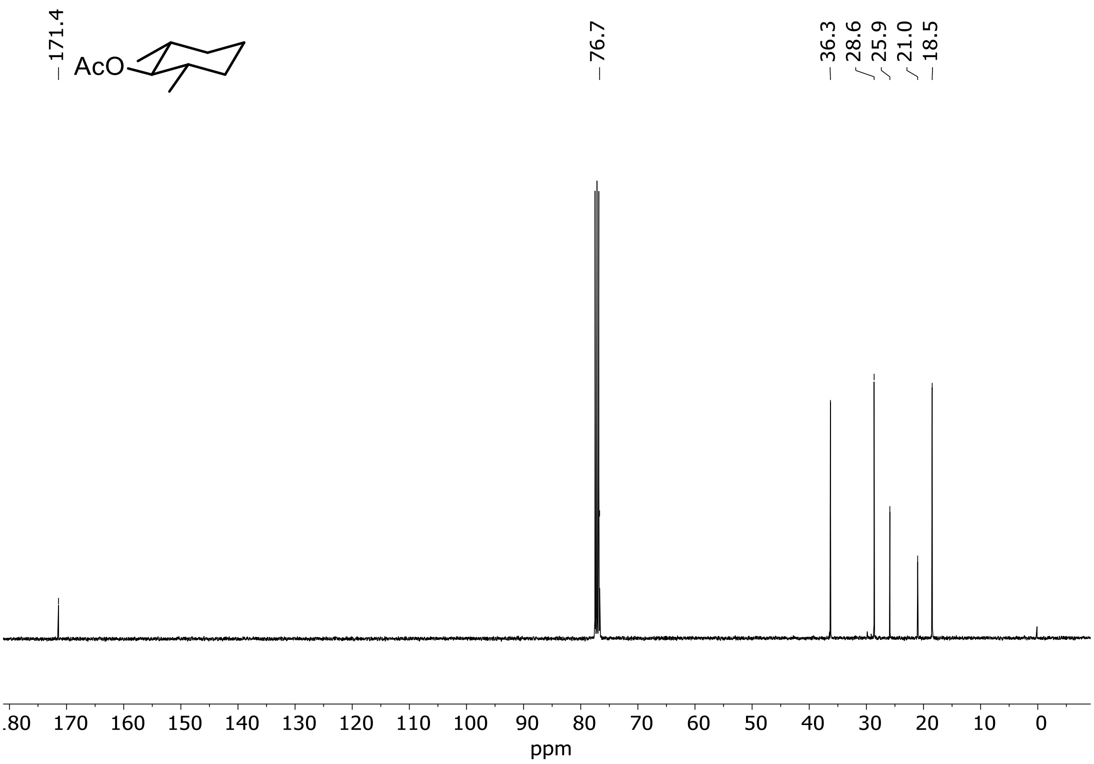

^{1}H-NMR of **D1** in $CDCl_3$

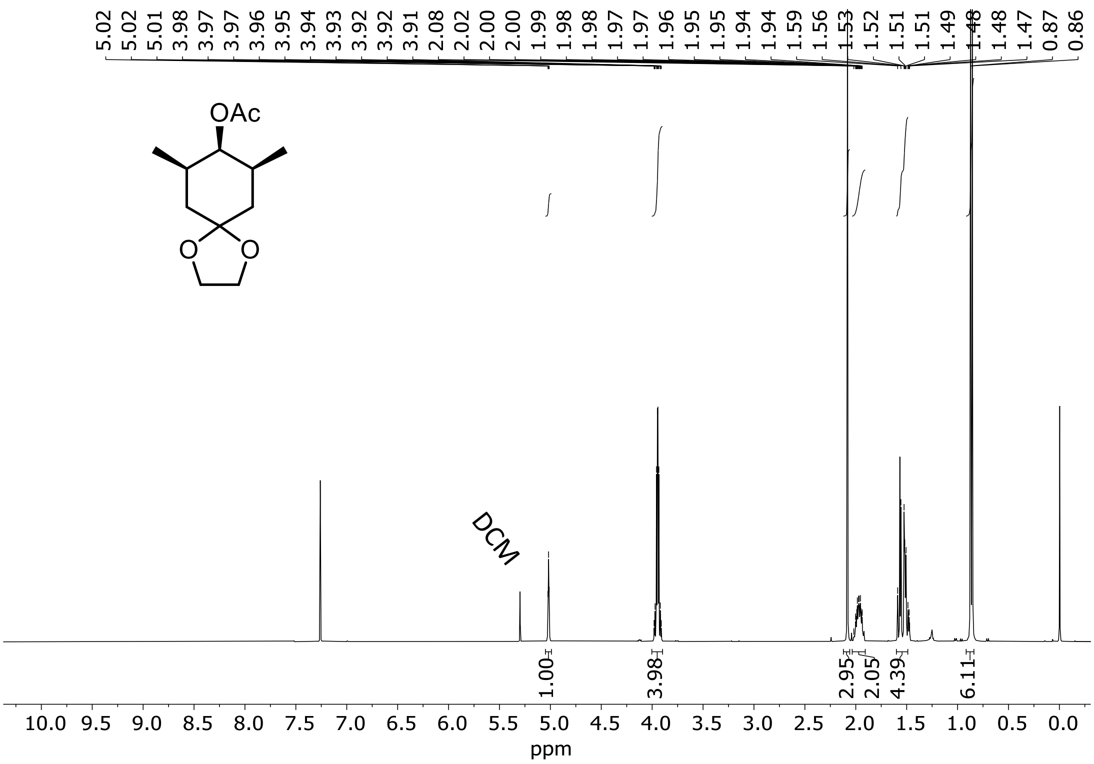

^{13}C-NMR of **D1** in $CDCl_3$

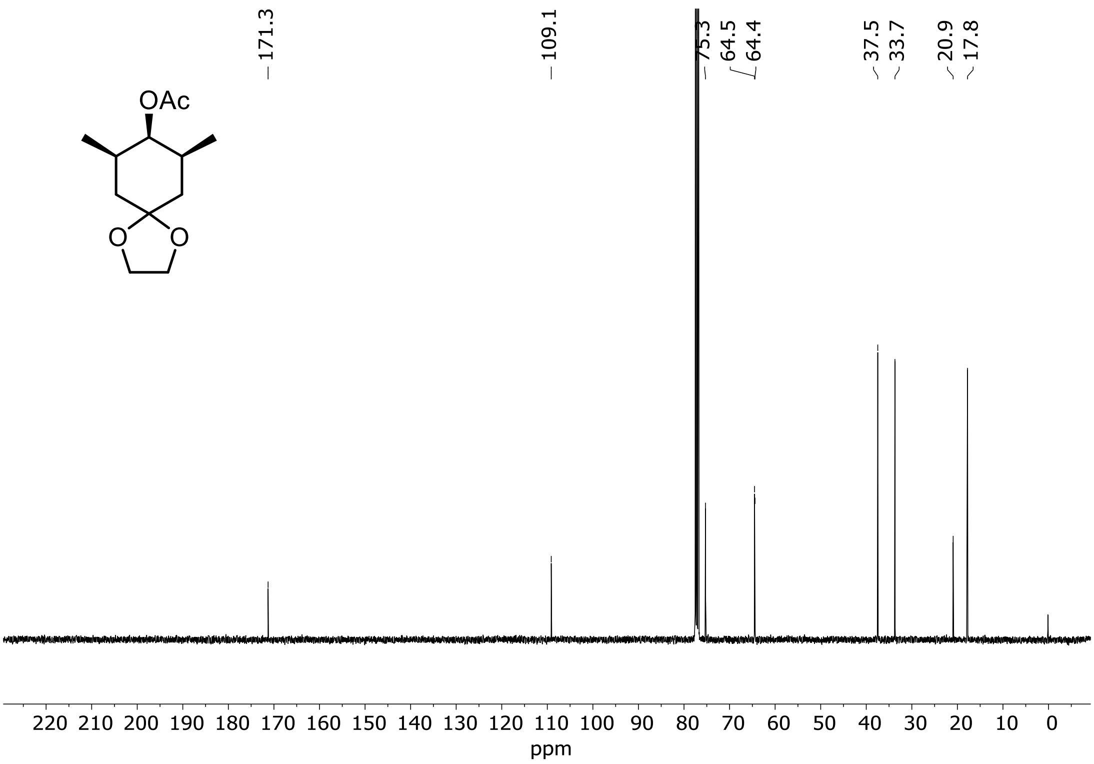

^{1}H-NMR of **D2** in $CDCl_3$

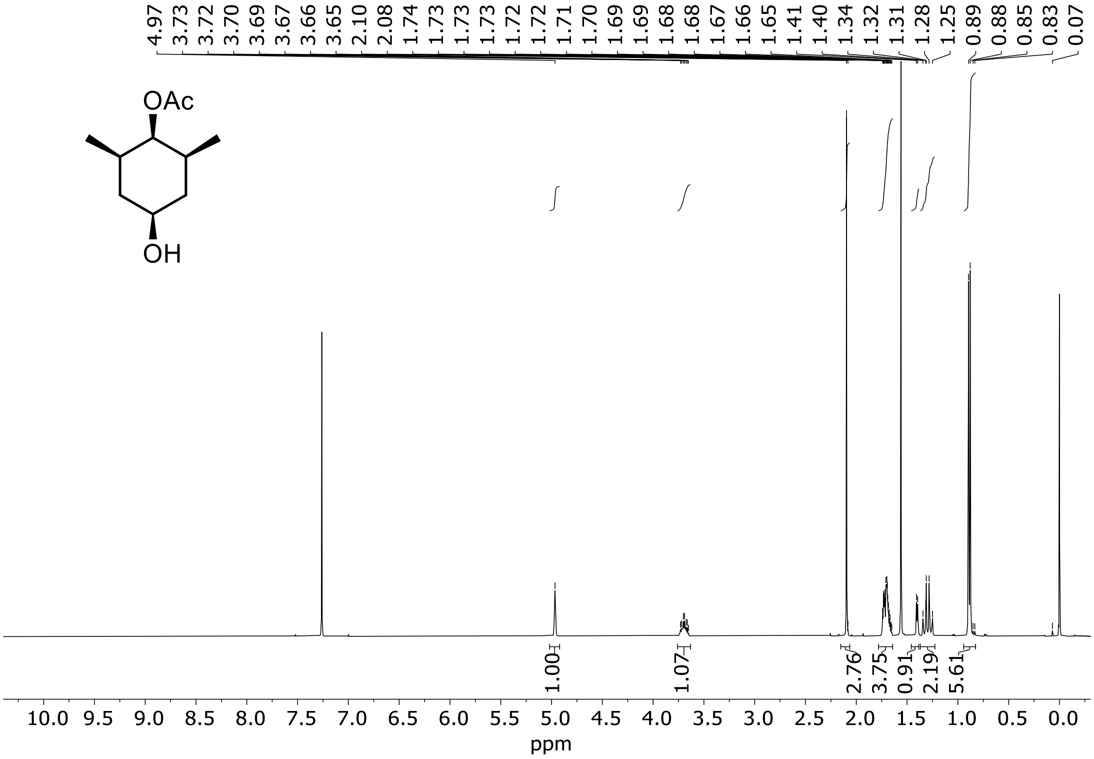

^{13}C-NMR of **D2** in $CDCl_3$

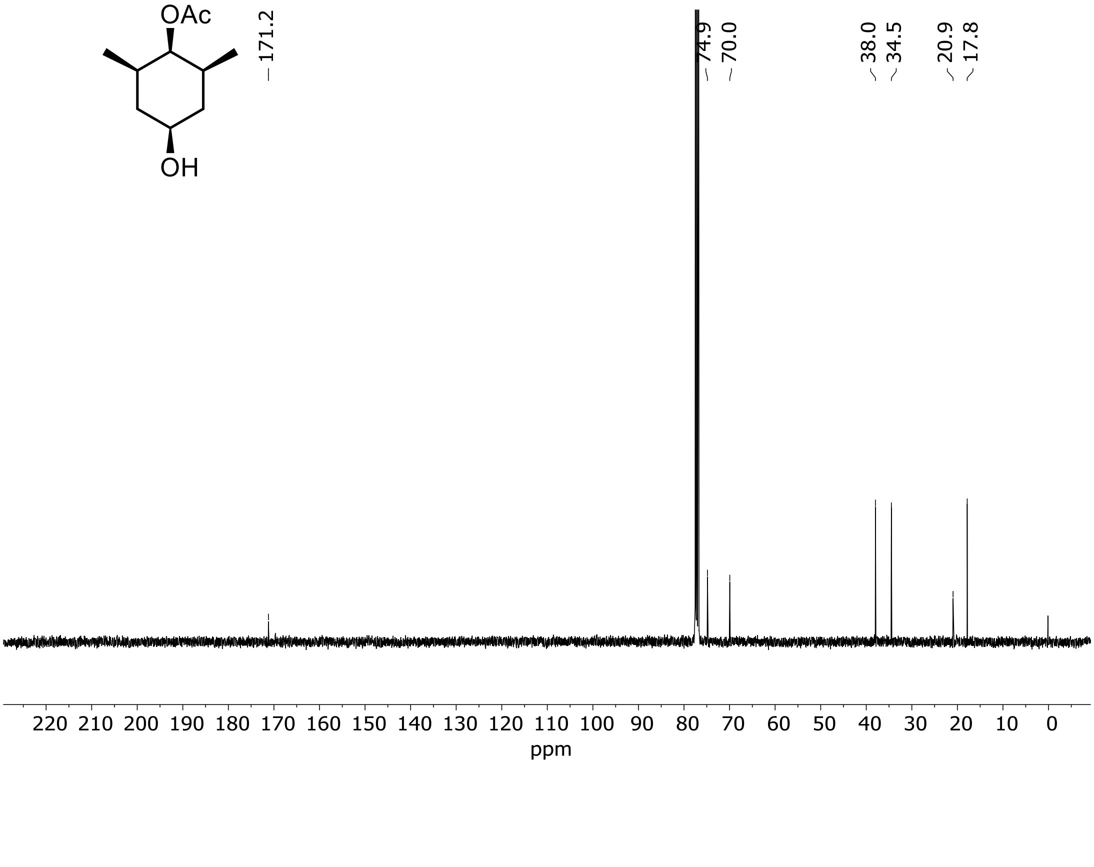

^{1}H-NMR of **31a** in $CDCl_3$

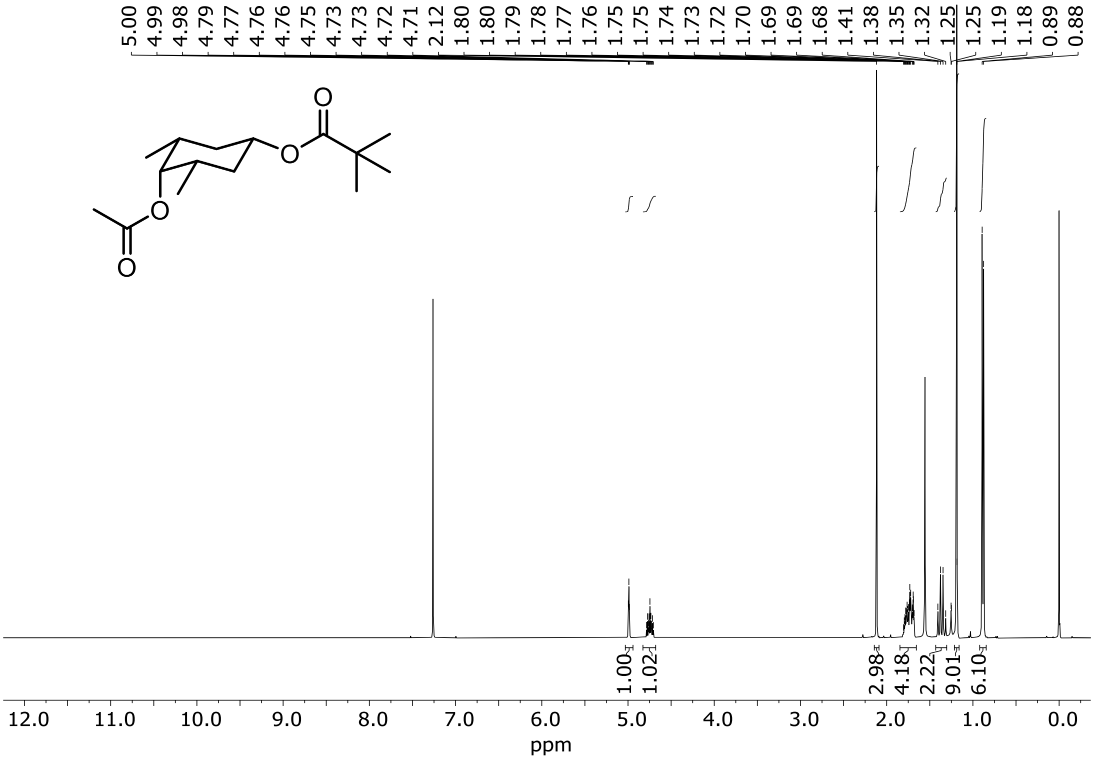

^{1}H-NMR of **31a** in $CDCl_3$

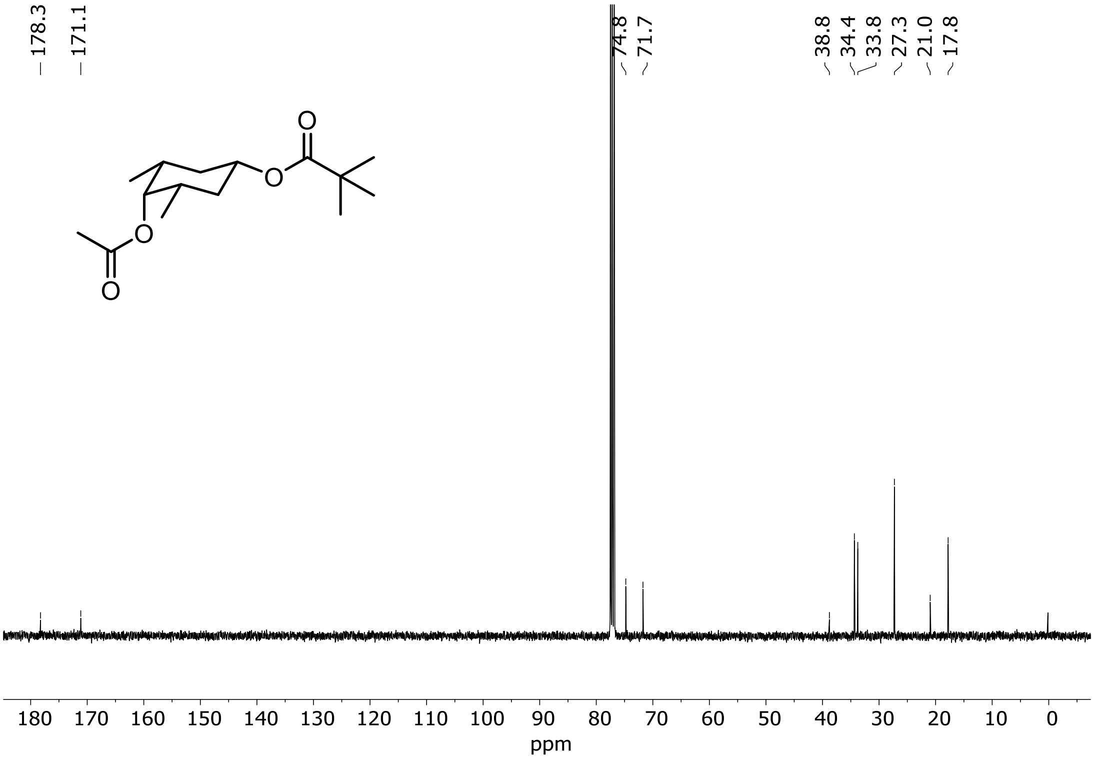

^{1}H-NMR of **^{TIPS}mpea** in $CDCl_3$

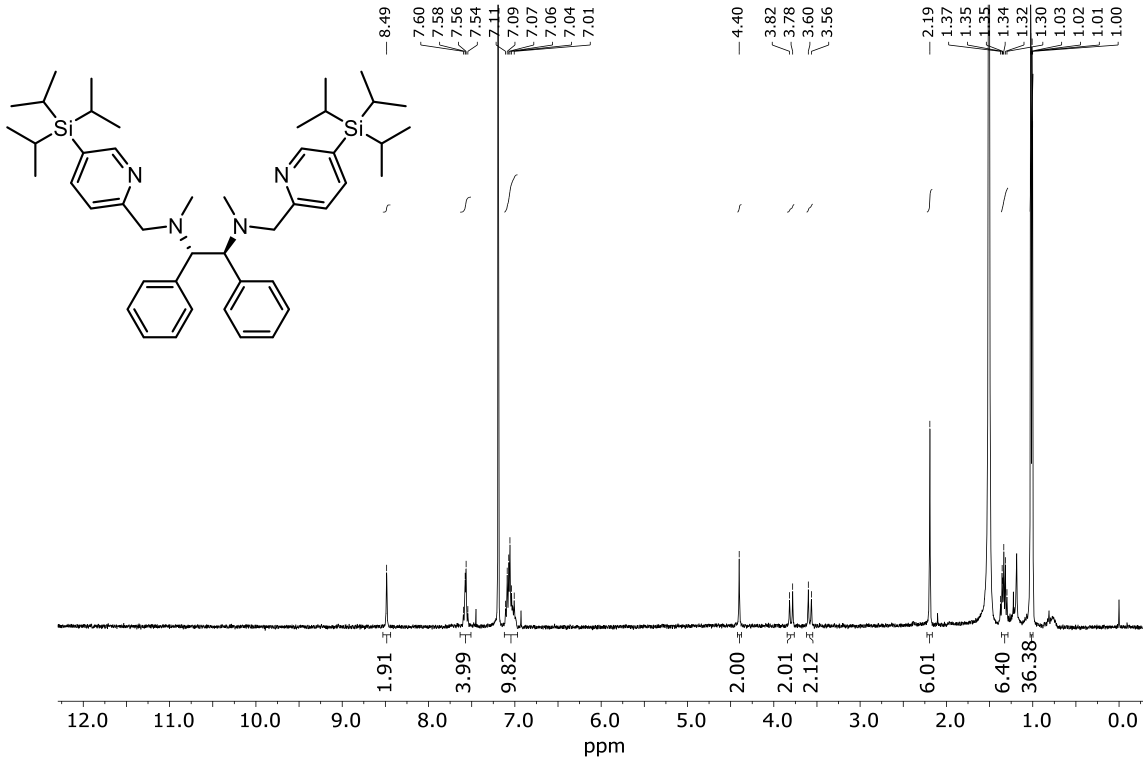

^{13}C-NMR of **^{TIPS}mpea** in $CDCl_3$

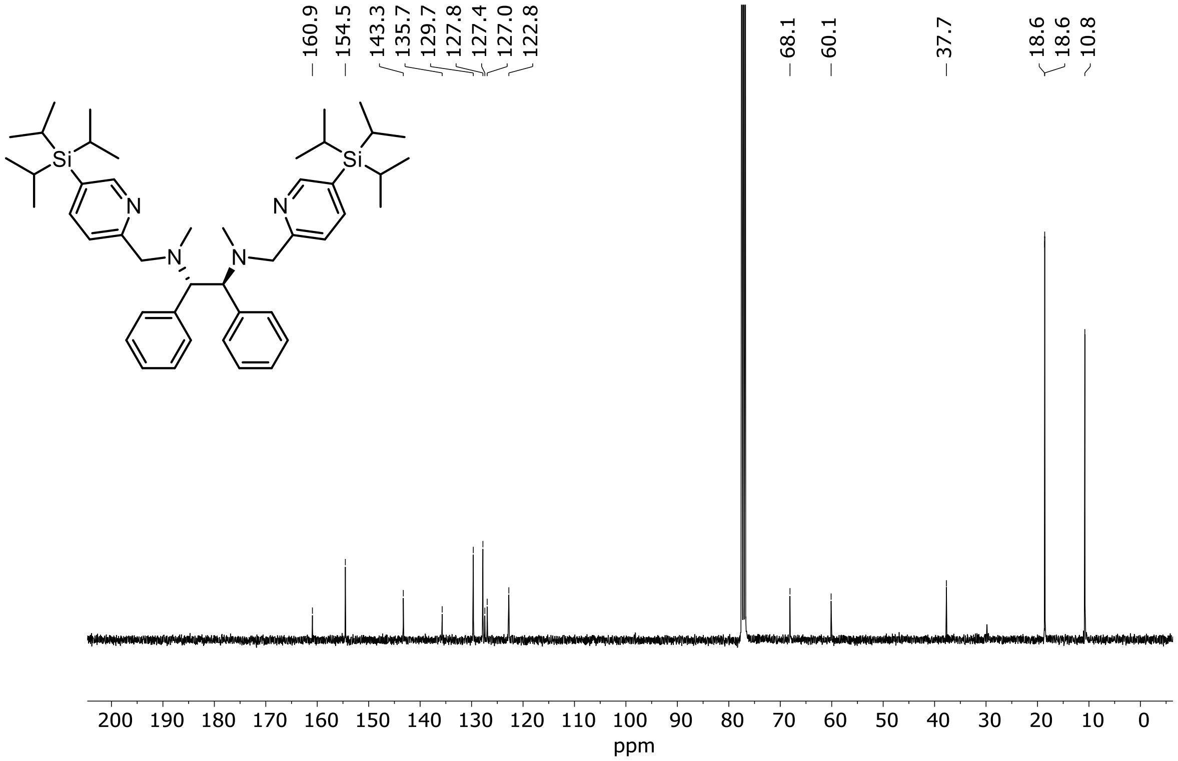

^{1}H-NMR of **1b**(OH-3 ax) in $CDCl_3$

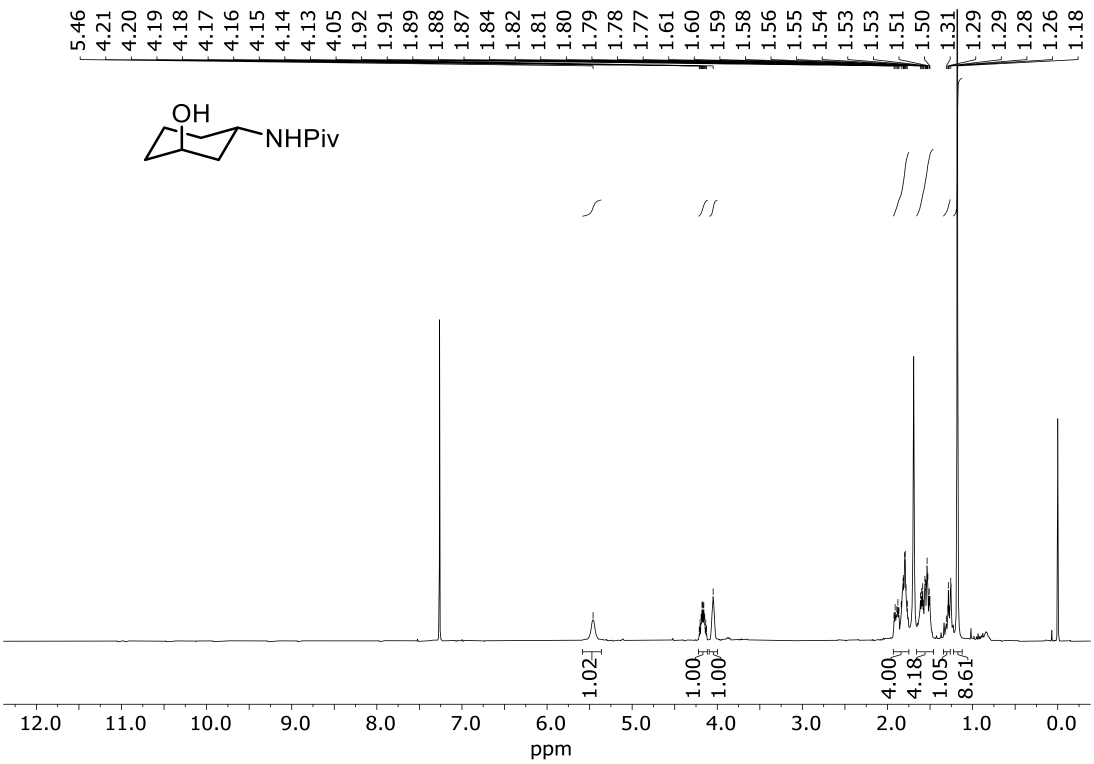

^{13}C-NMR of **1b**(OH-3 ax) in $CDCl_3$

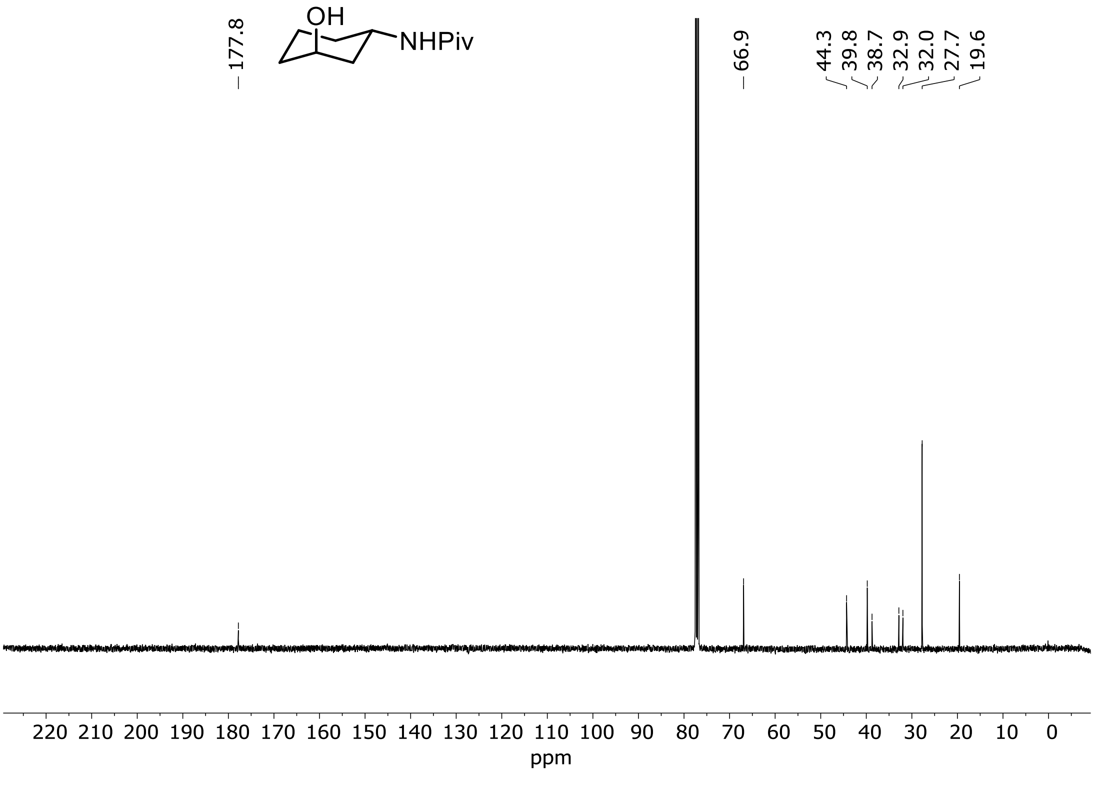

^{1}H-NMR of **1b**(OAc-3 ax) in $CDCl_3$

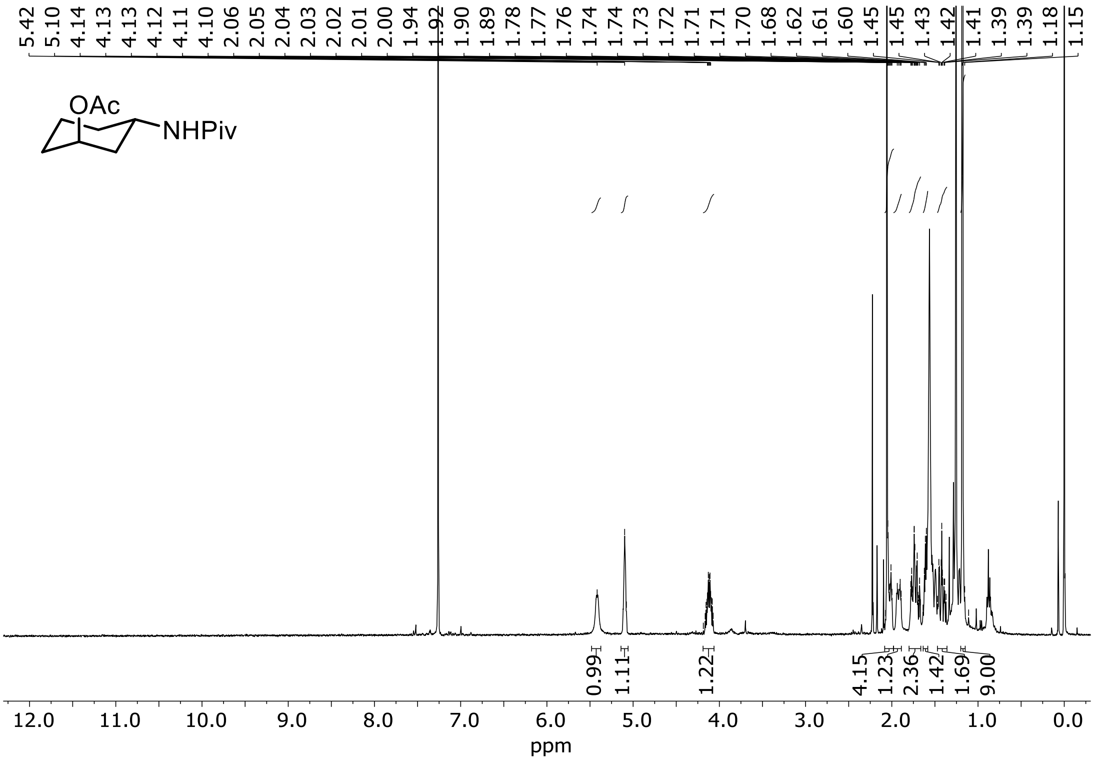

^{13}C-NMR of **1b**(OAc-3 ax) in $CDCl_3$

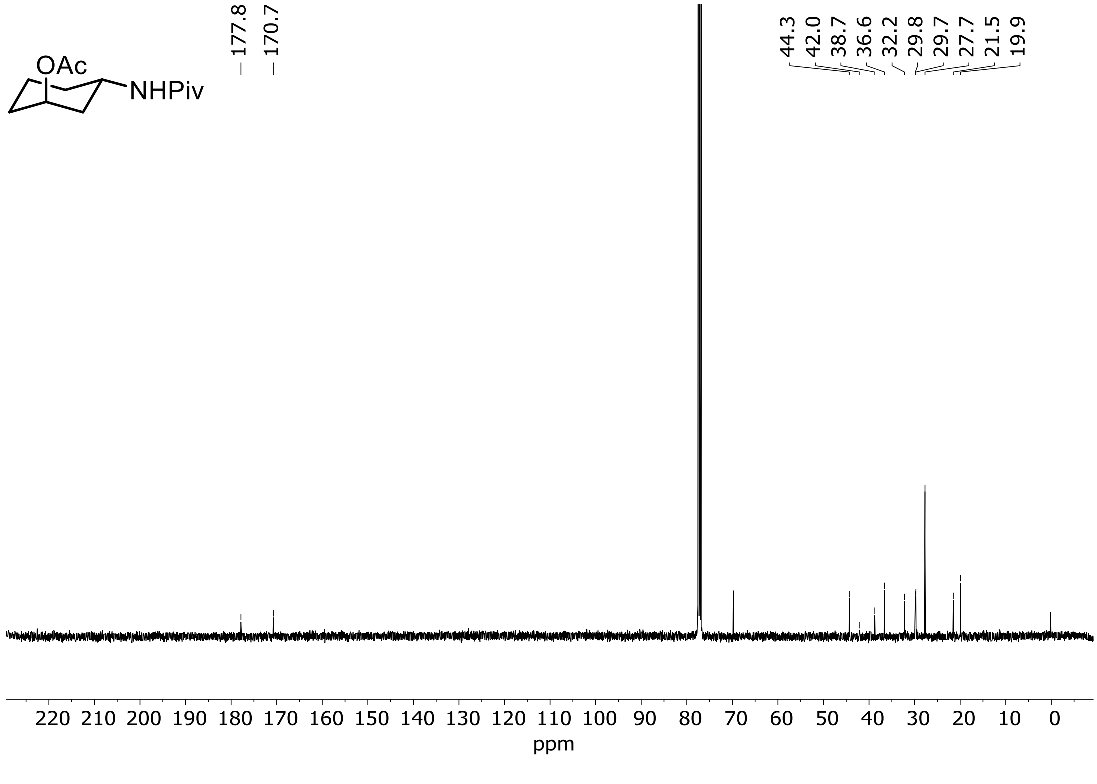

^{1}H-NMR of **1c**(OH-3 eq) in $CDCl_3$

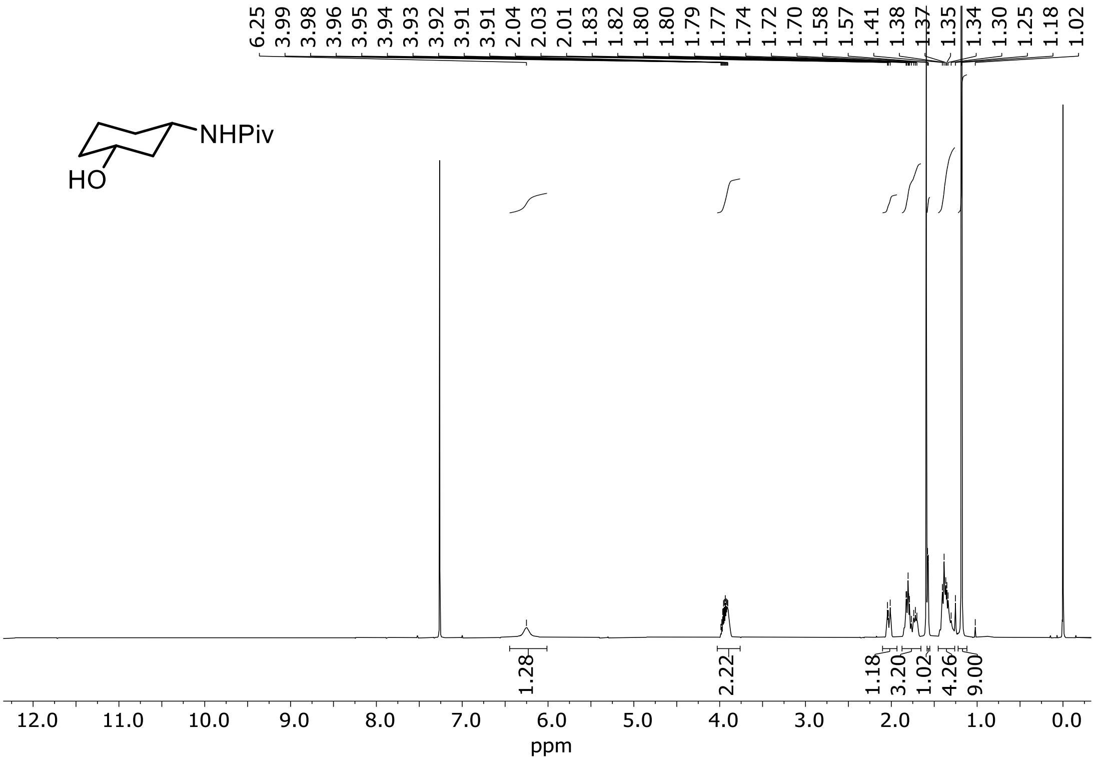

^{13}C-NMR of **1c**(OH-3 eq) in $CDCl_3$

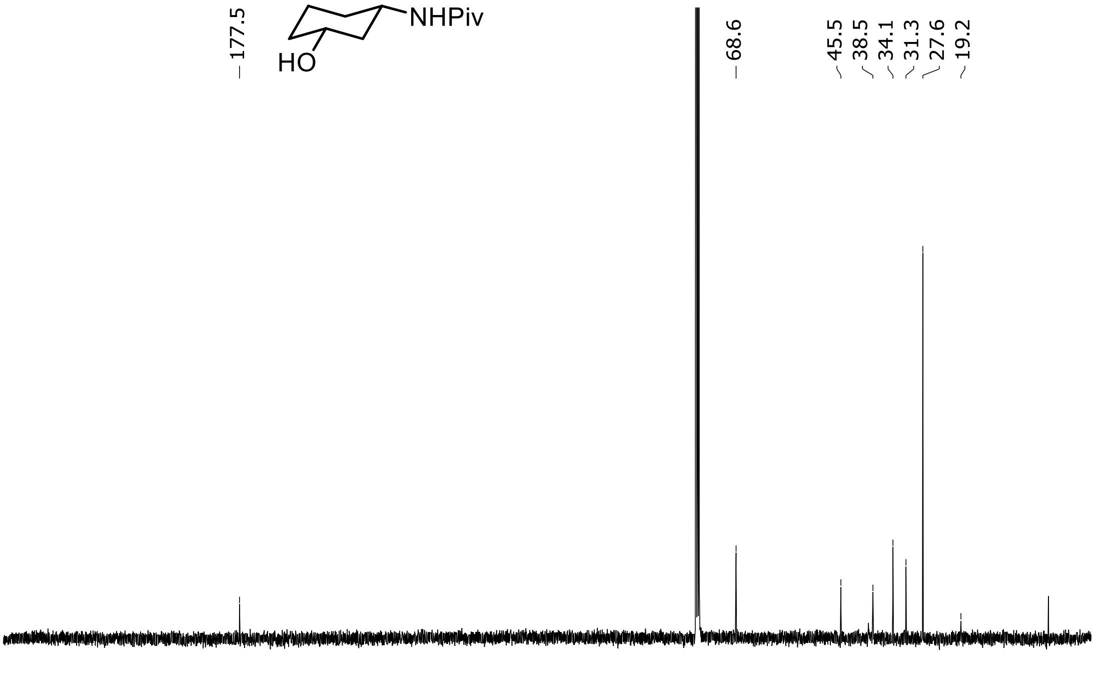

^{1}H-NMR of **1c**(OAc-3 eq) in $CDCl_3$

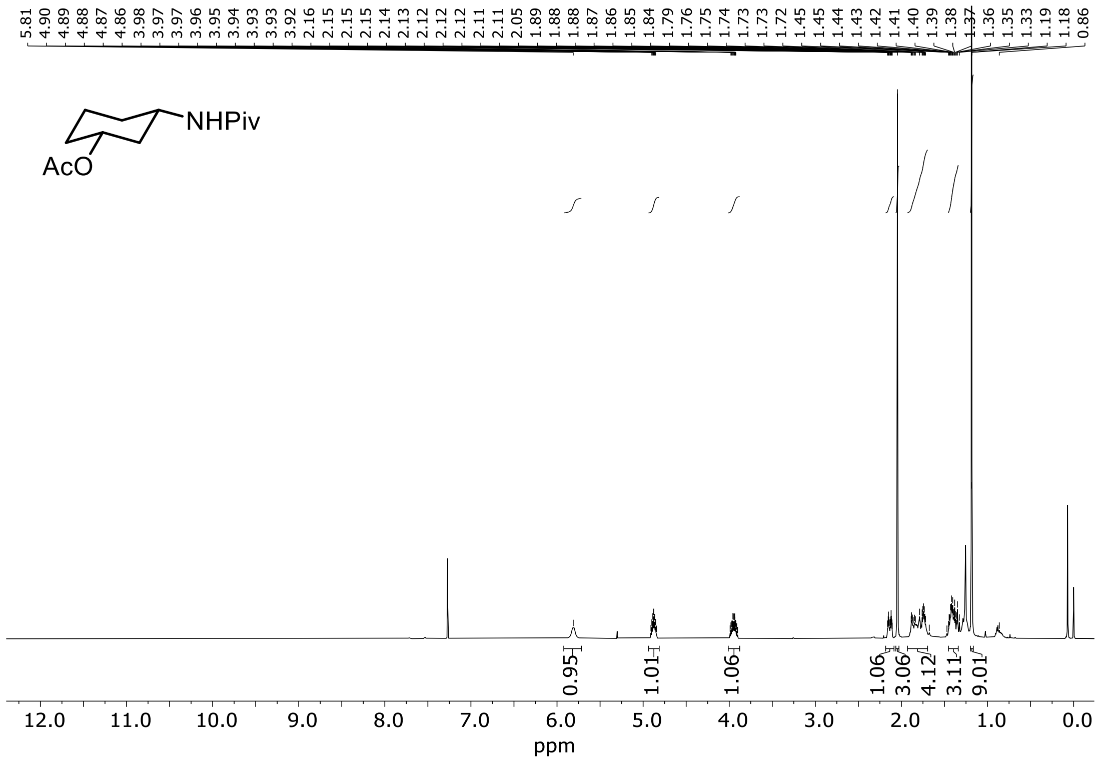

^{13}C-NMR of **1c**(OAc-3 eq) in $CDCl_3$

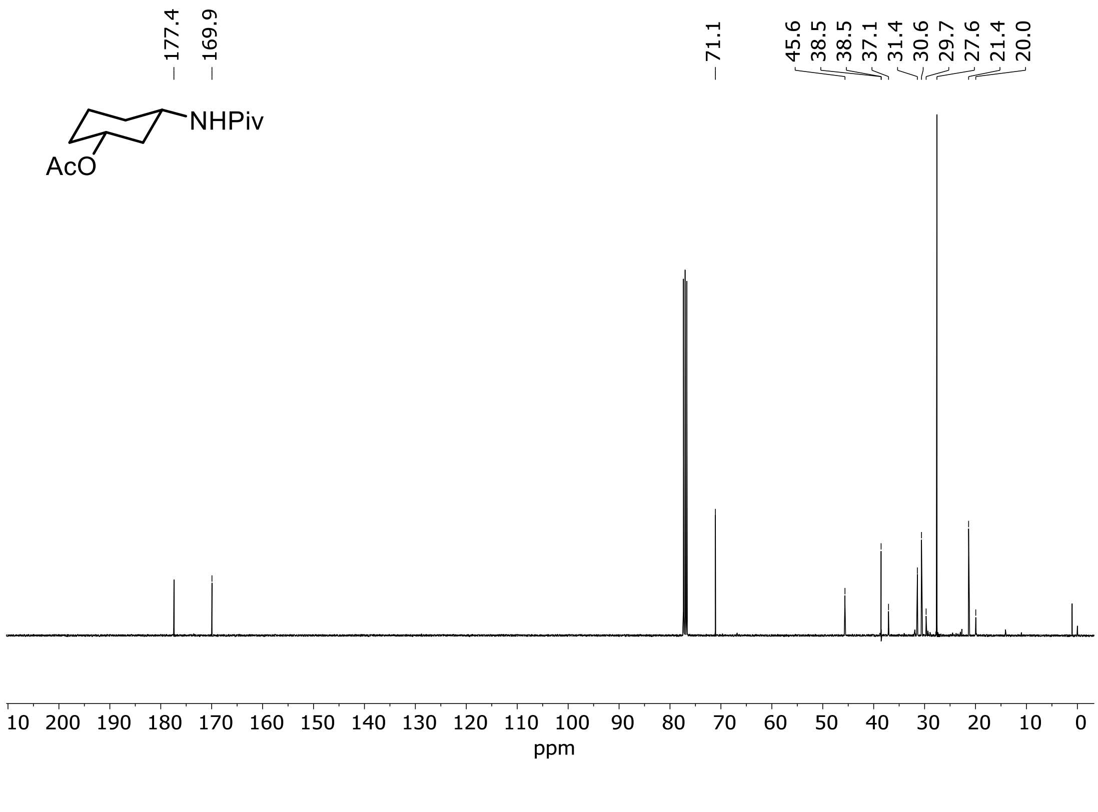

^{1}H-NMR of **1d**(OH-4 ax) in $CDCl_3$

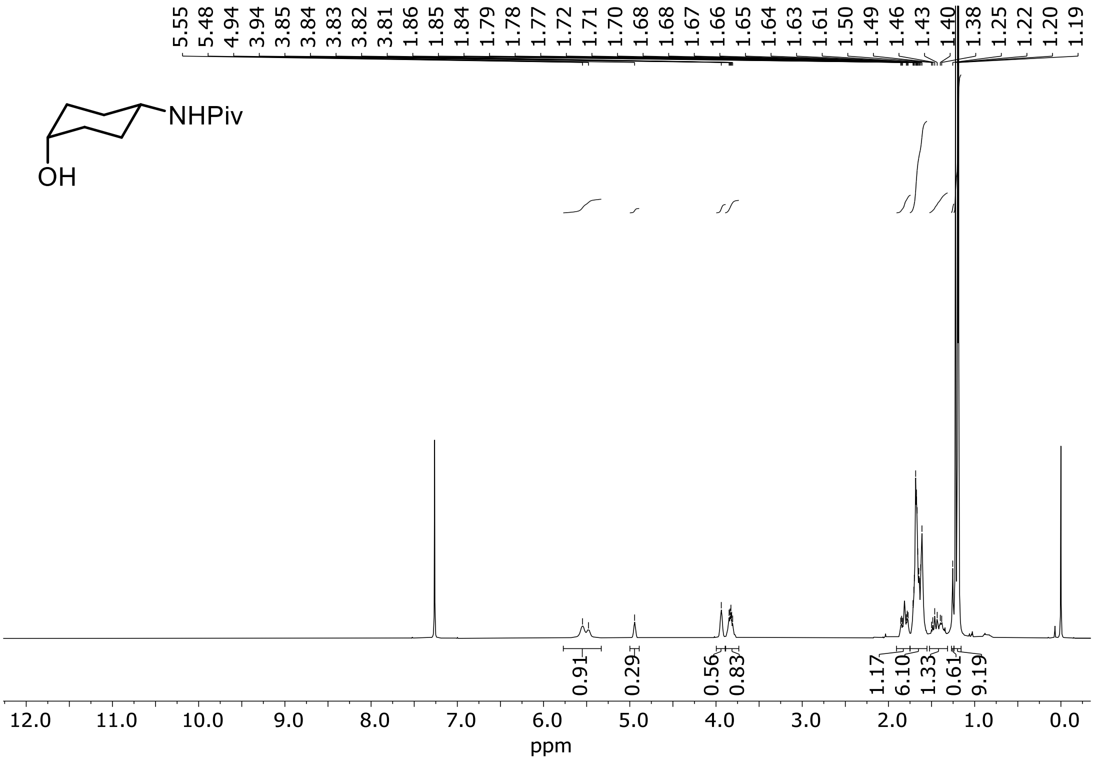

^{13}C-NMR of **1d**(OH-4 ax) in $CDCl_3$

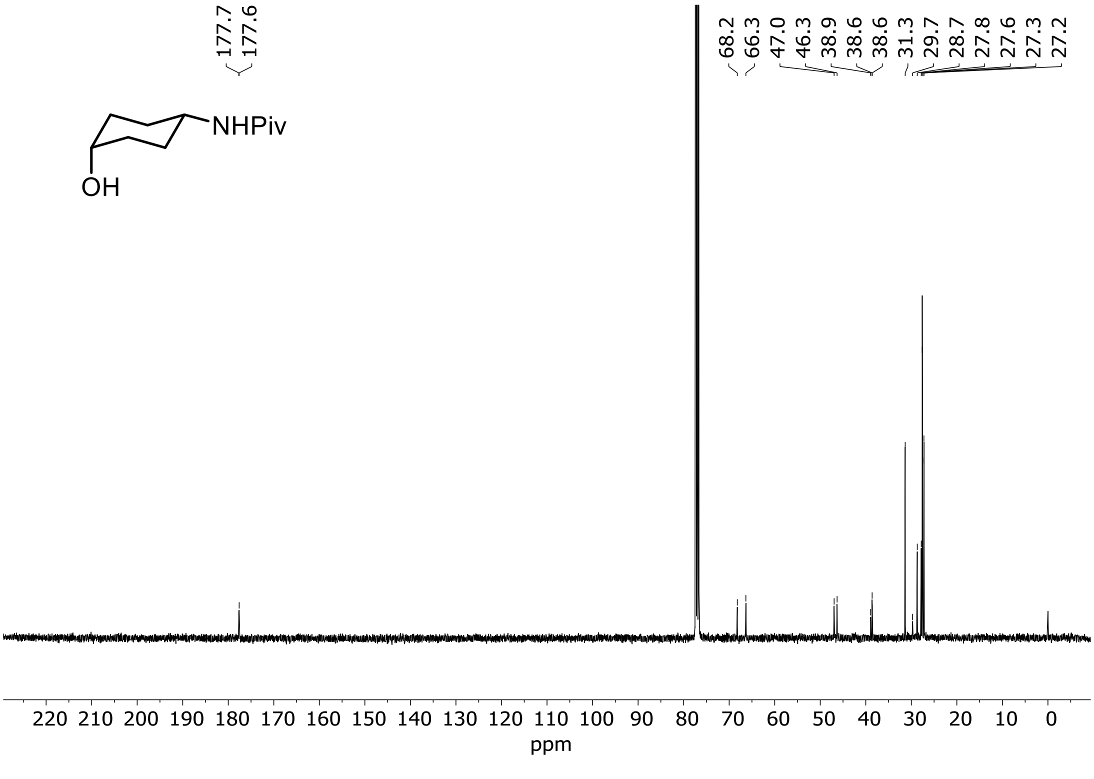

^{1}H-NMR of **1d**(OAc-4 ax) in $CDCl_3$

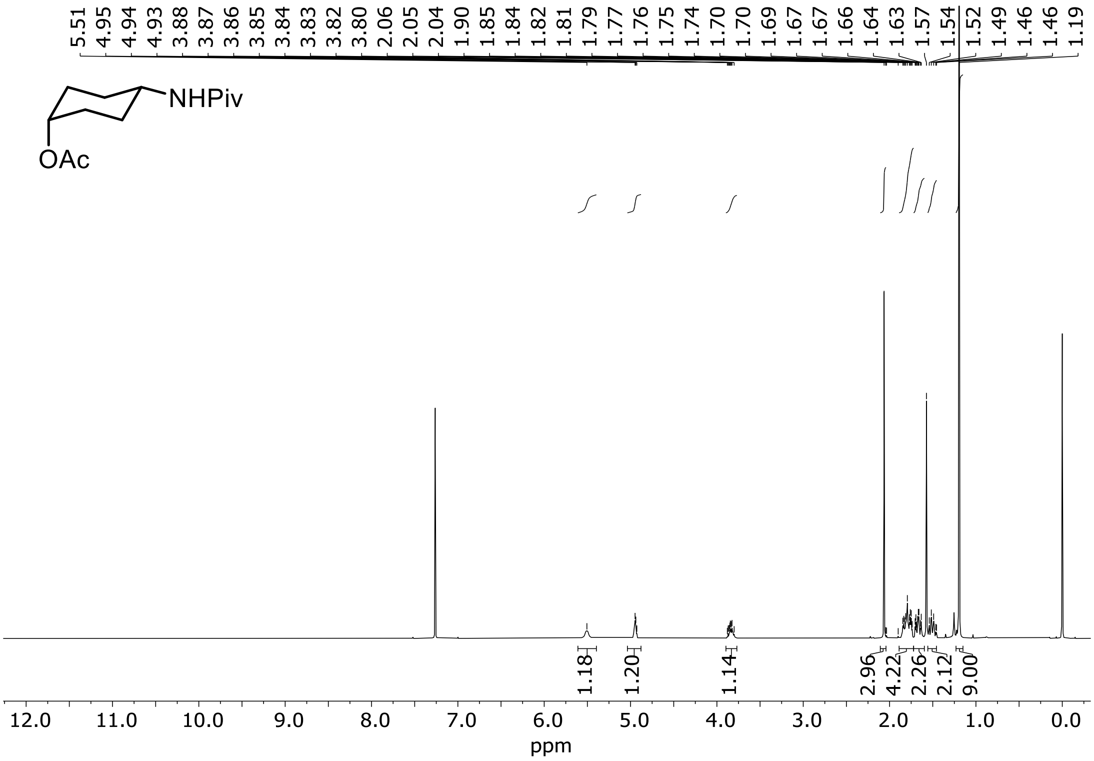

^{13}C-NMR of **1d**(OAc-4 ax) in $CDCl_3$

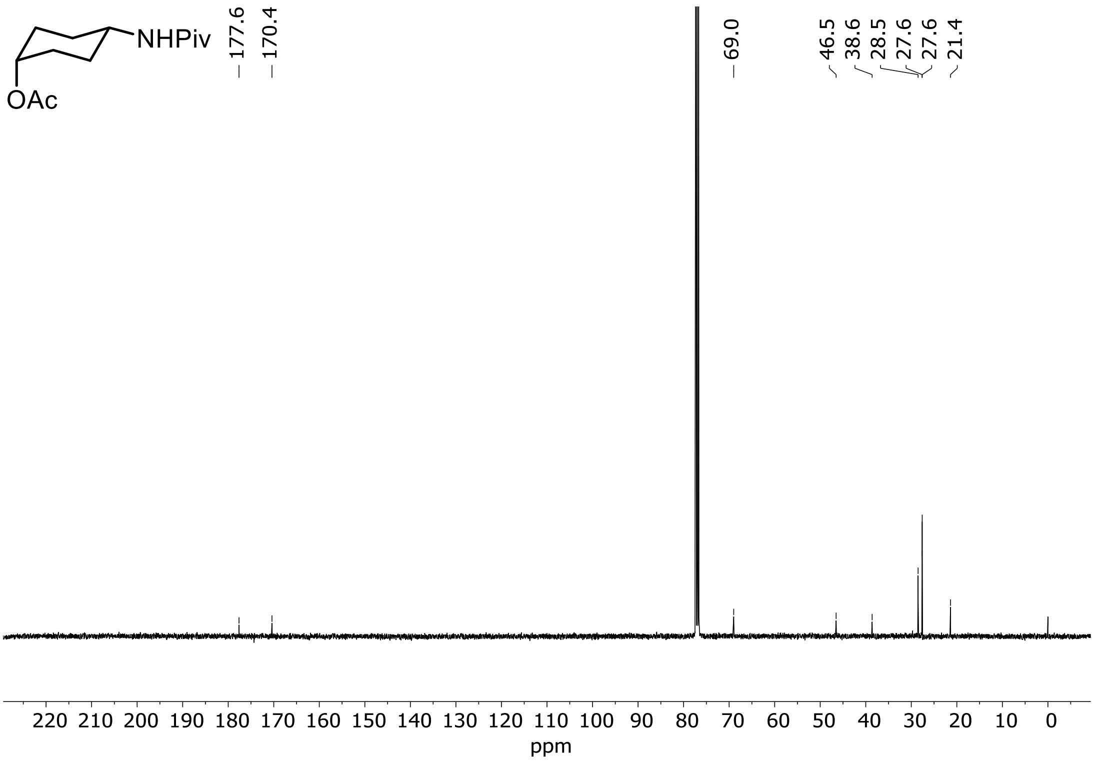

^{1}H-NMR of **1e**(OH-4 eq) in $CDCl_3$

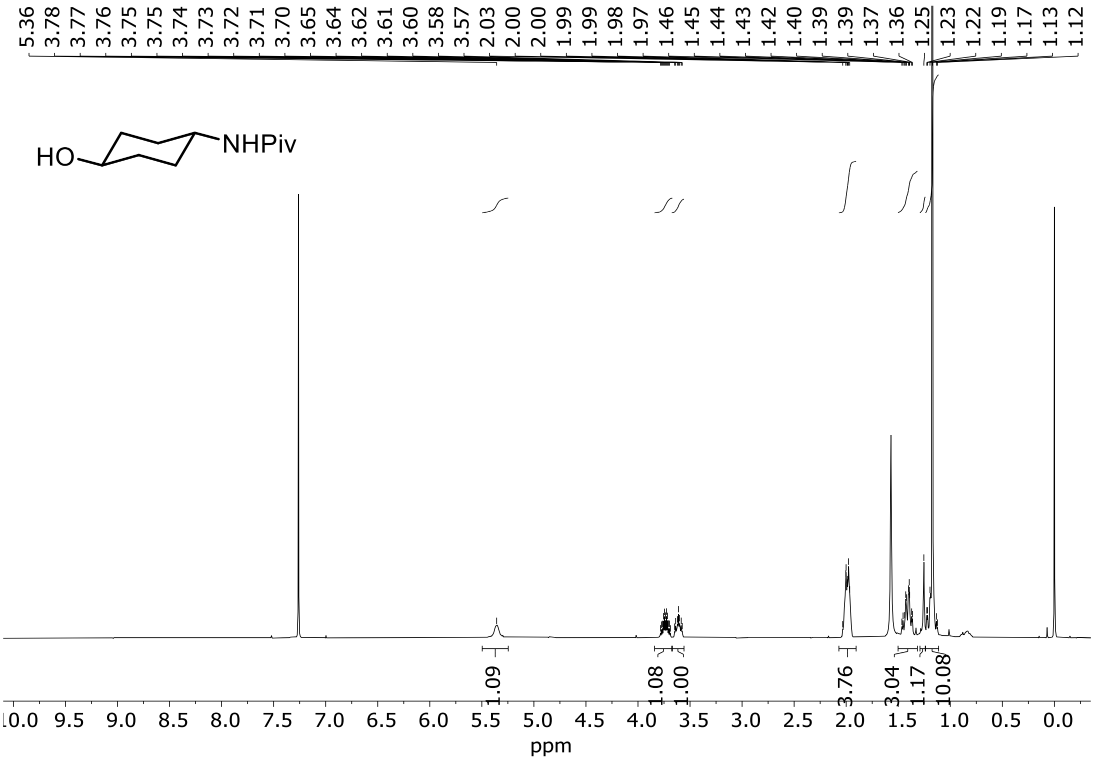

^{13}C-NMR of **1e**(OH-4 eq)in $CDCl_3$

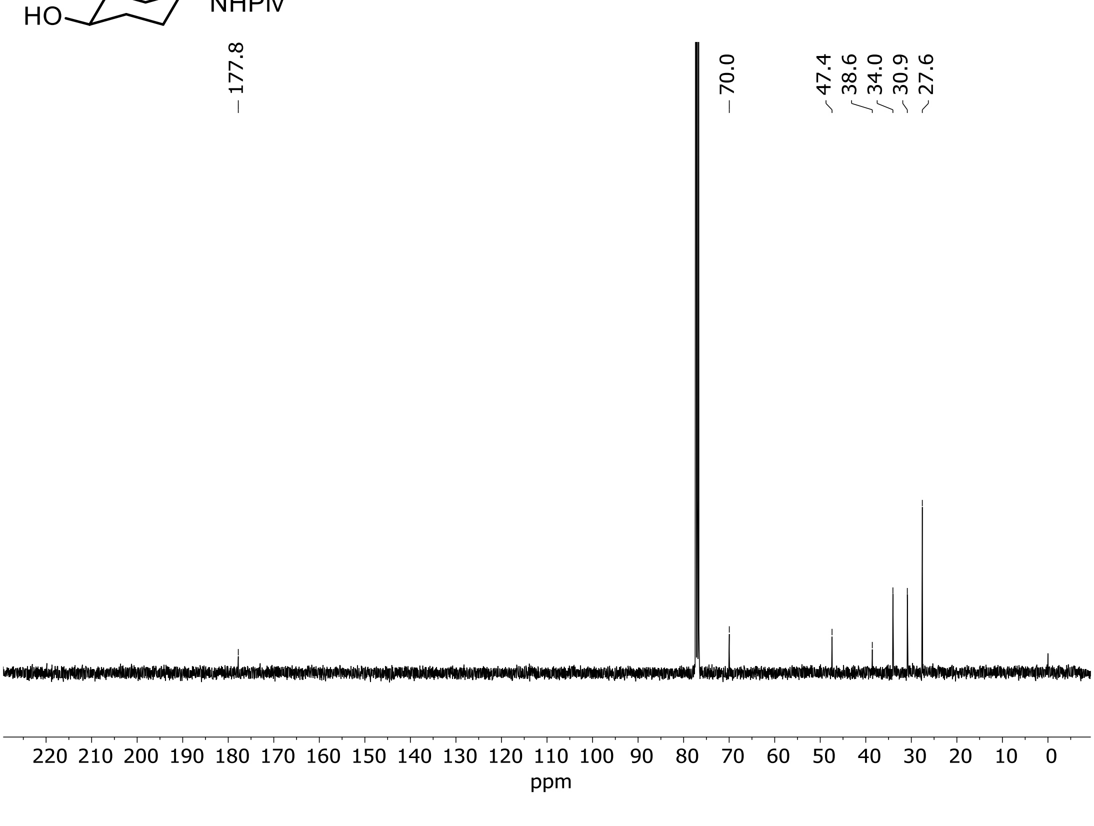

^{1}H-NMR of **1e**(OAc-4 eq) in $CDCl_3$

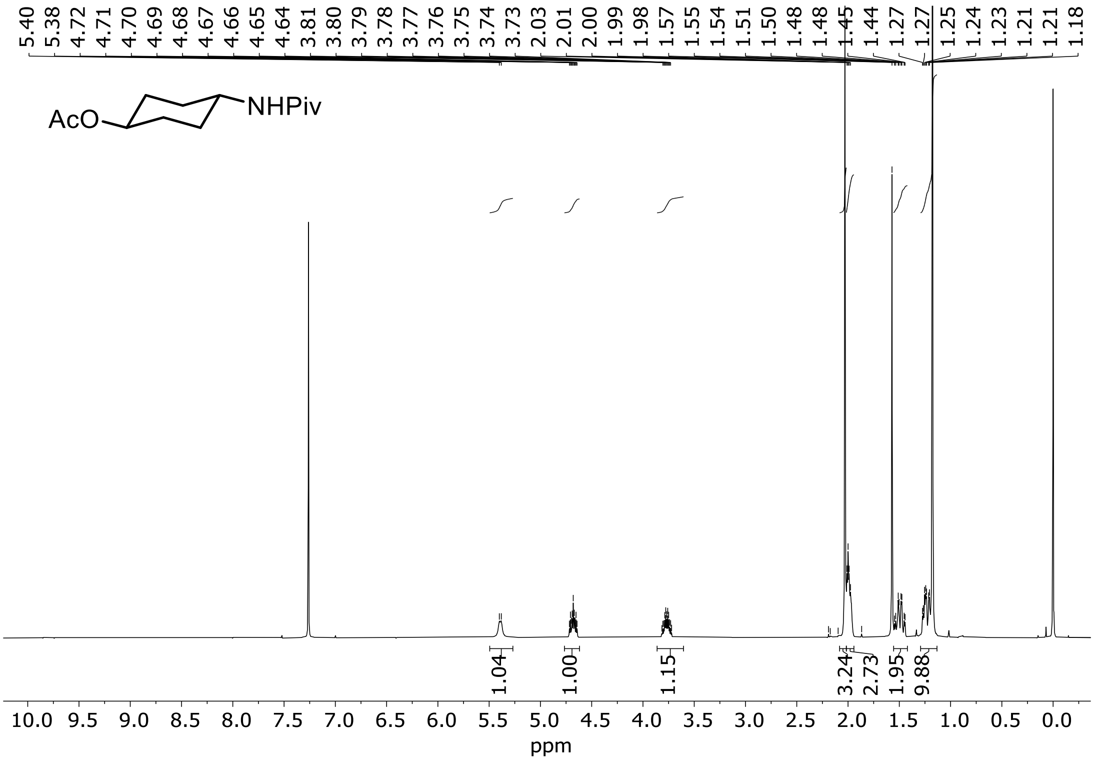

^{1}H-NMR of **1e**(OAc-4 eq) in $CDCl_3$

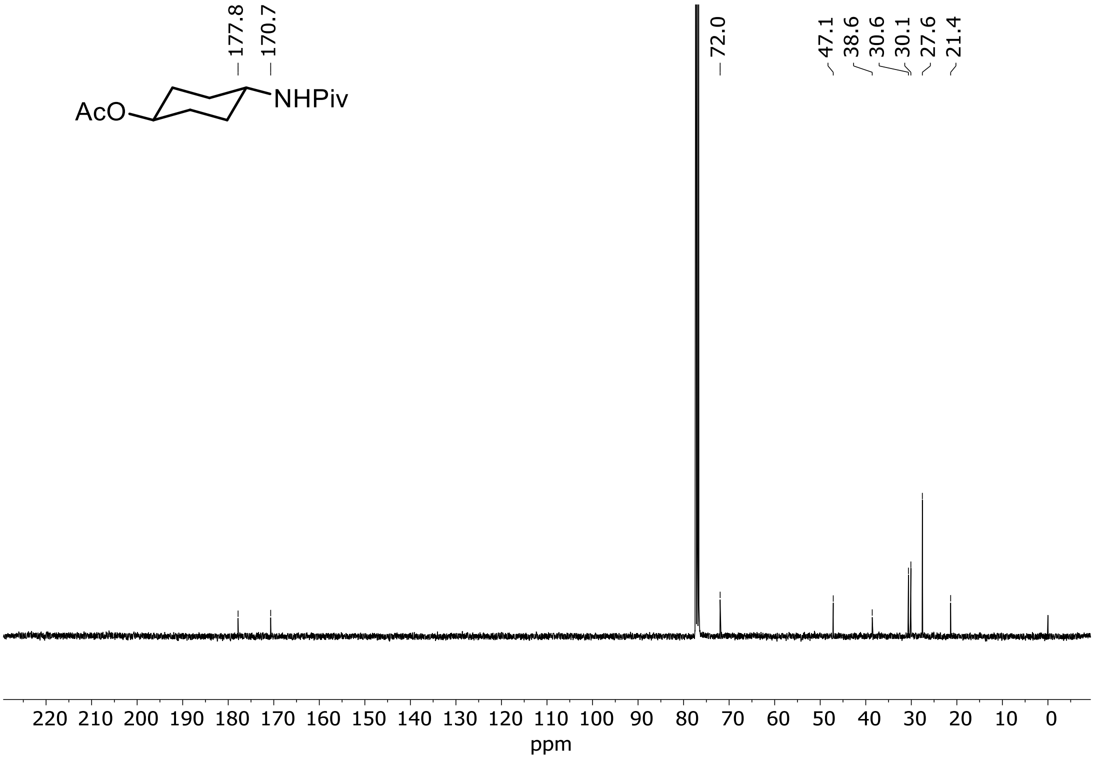

^{1}H-NMR of **2b** in $CDCl_3$

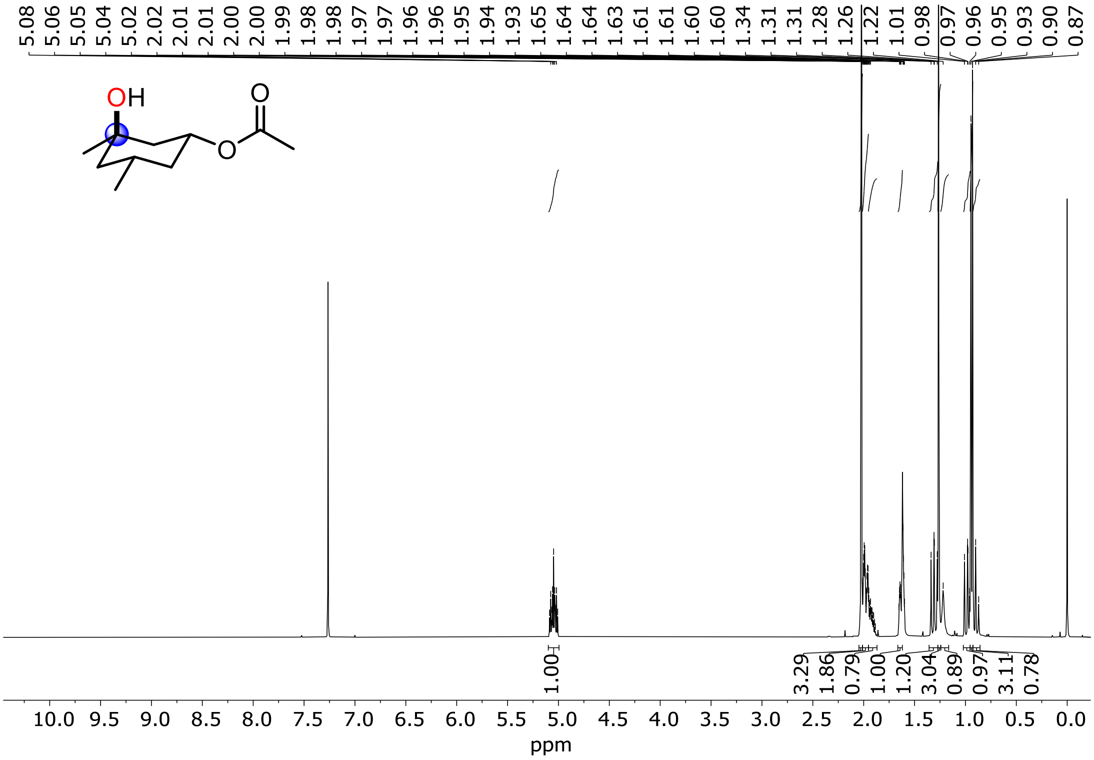

^{13}C-NMR of **2b** in $CDCl_3$

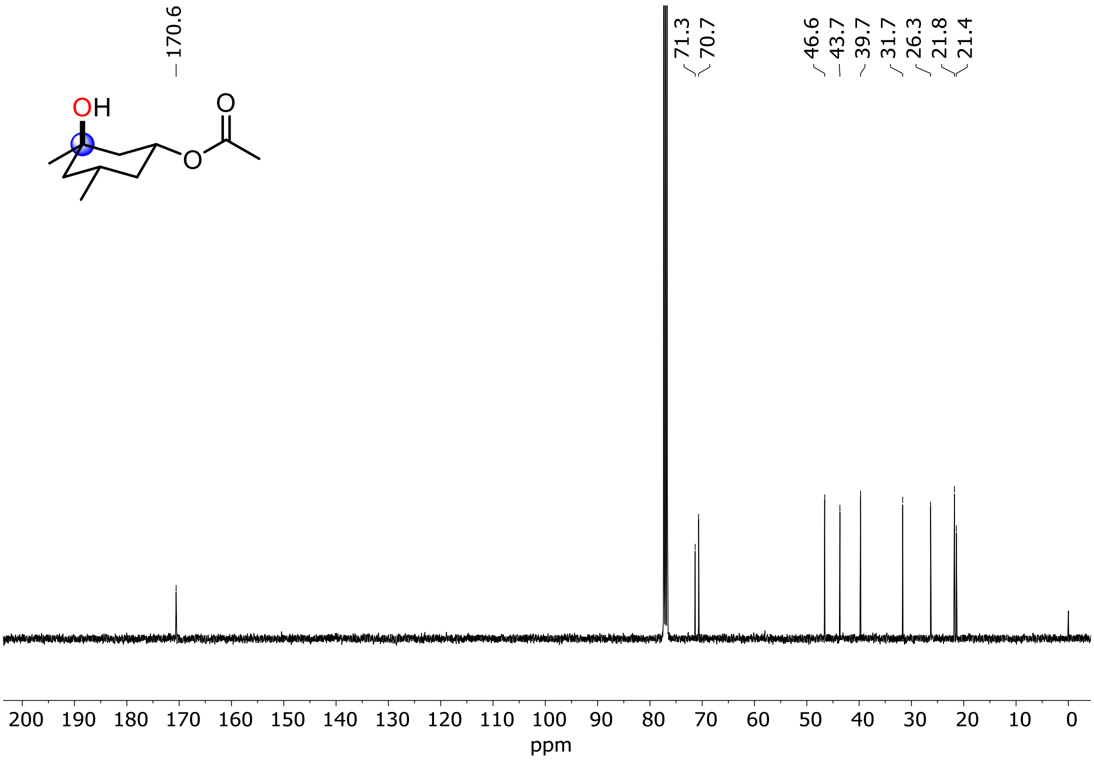

COSY-NMR of **2b** in $CDCl_3$

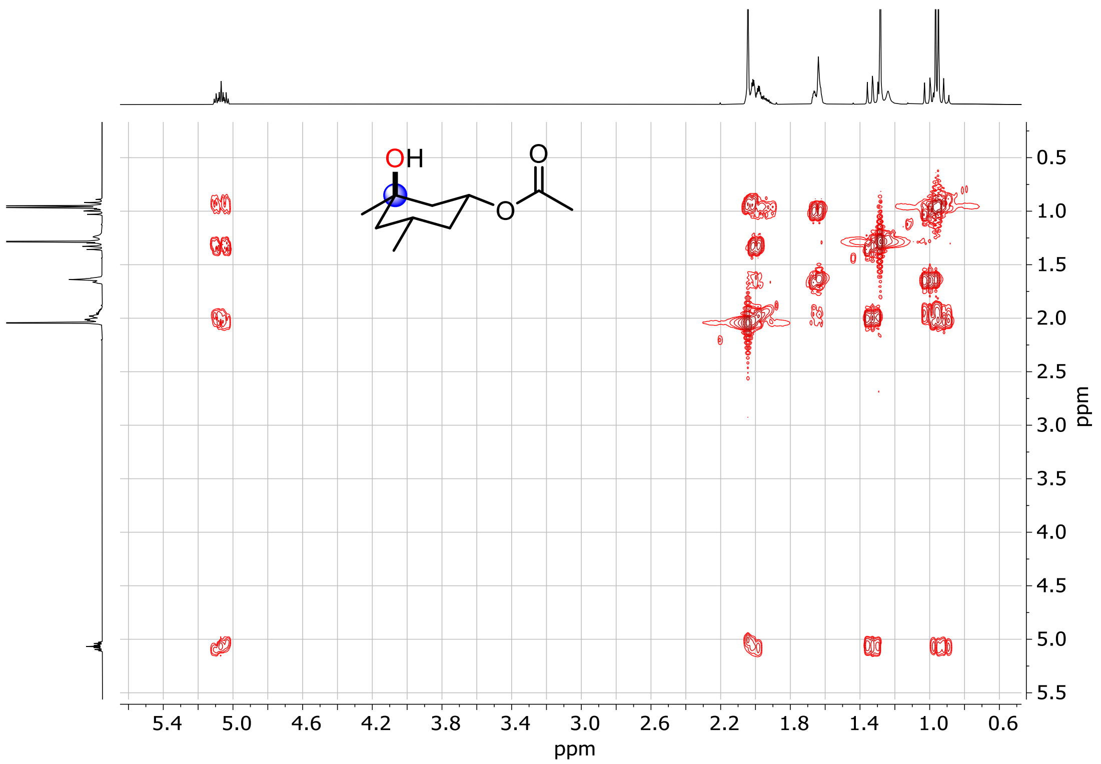

HSQC-NMR of **2b** in $CDCl_3$

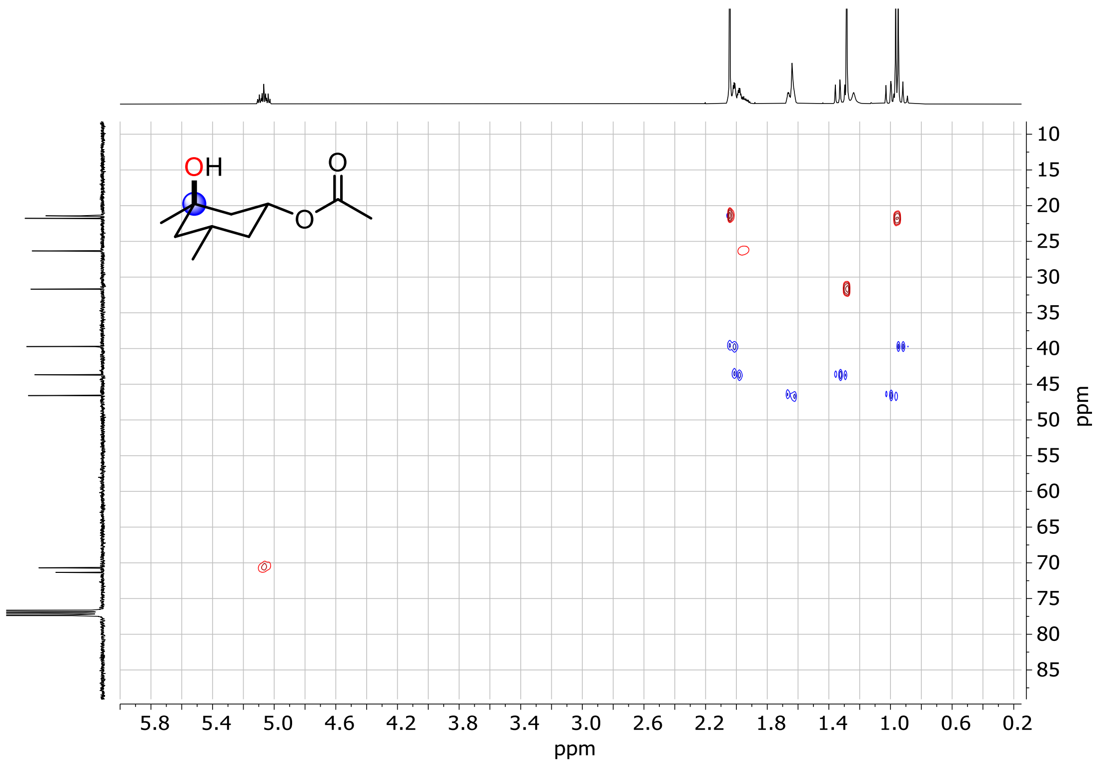

^{1}H-NMR of **3b** in $CDCl_3$

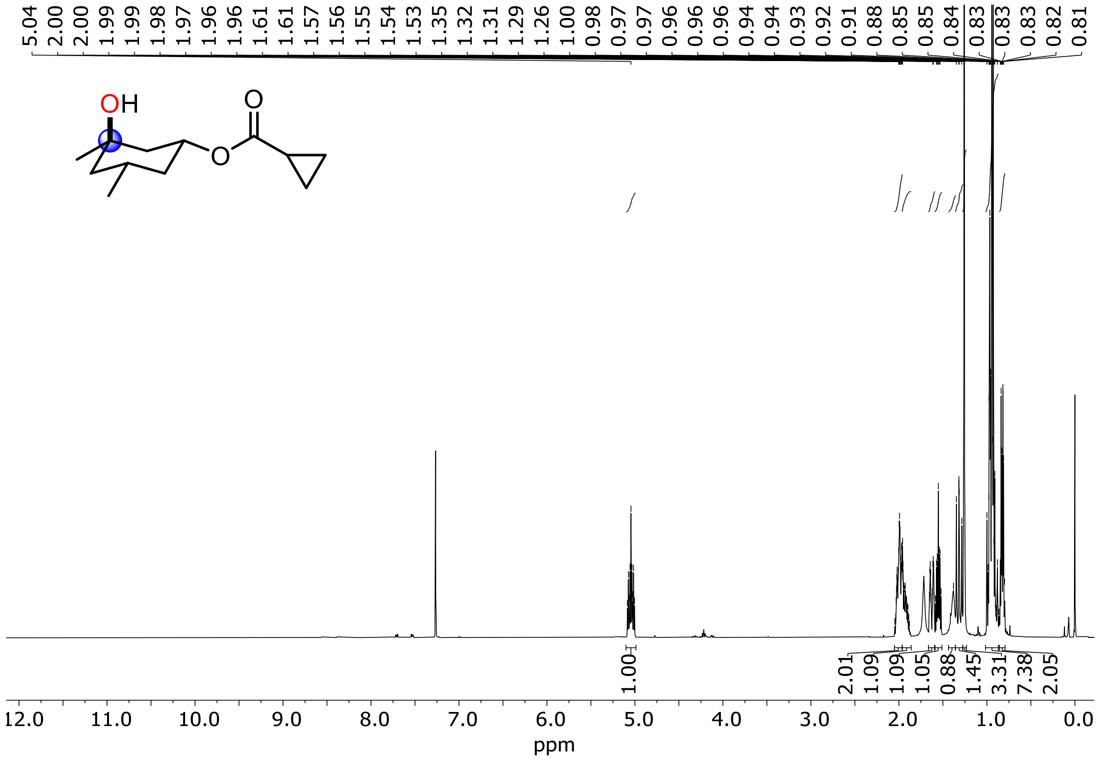

^{13}C-NMR of **3b** in $CDCl_3$

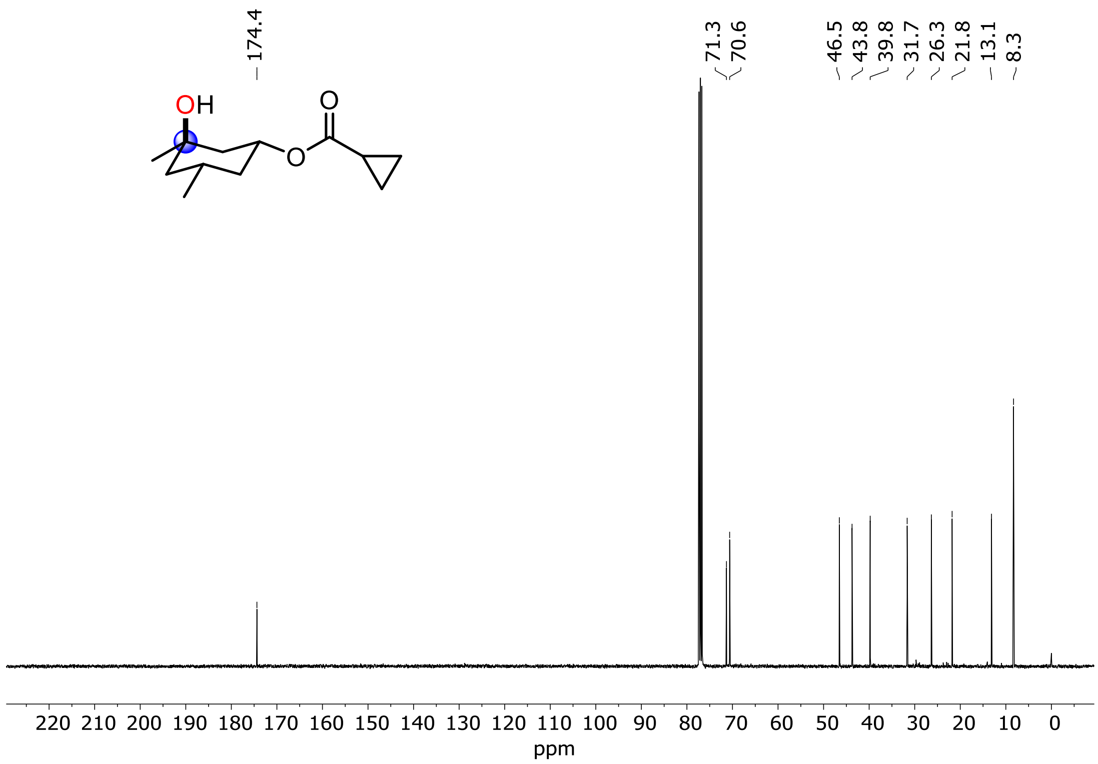

COSY-NMR of **3b** in $CDCl_3$

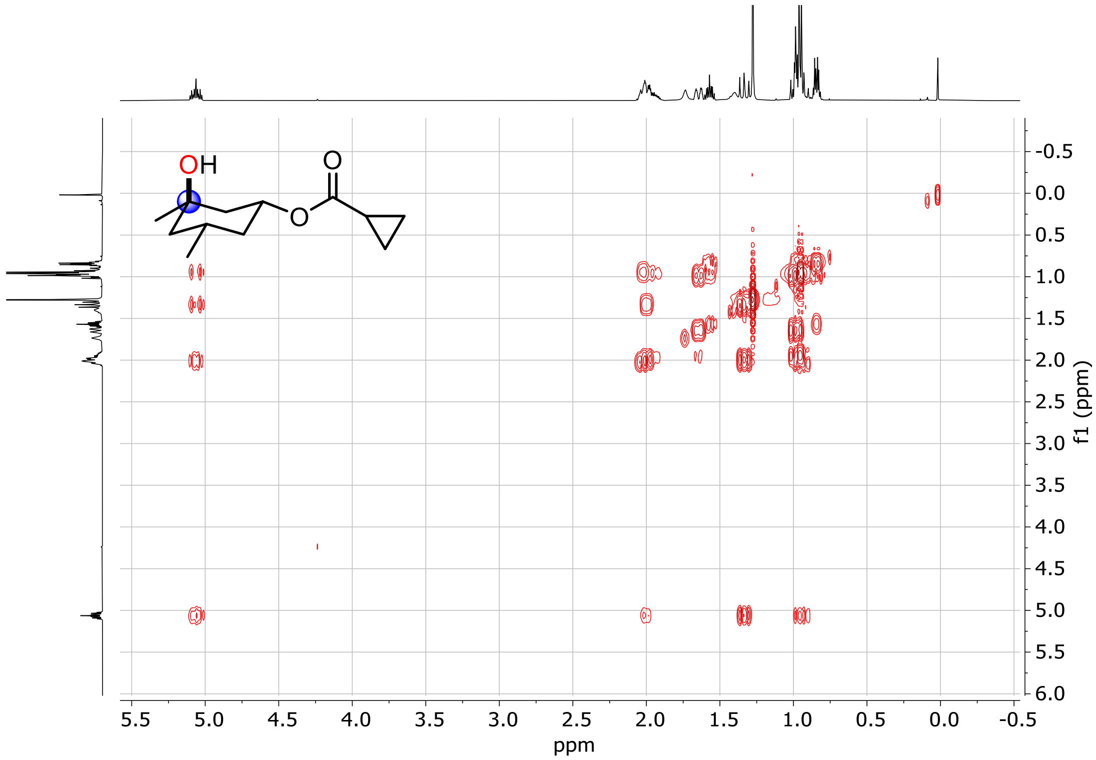

HSQC-NMR of **3b** in $CDCl_3$

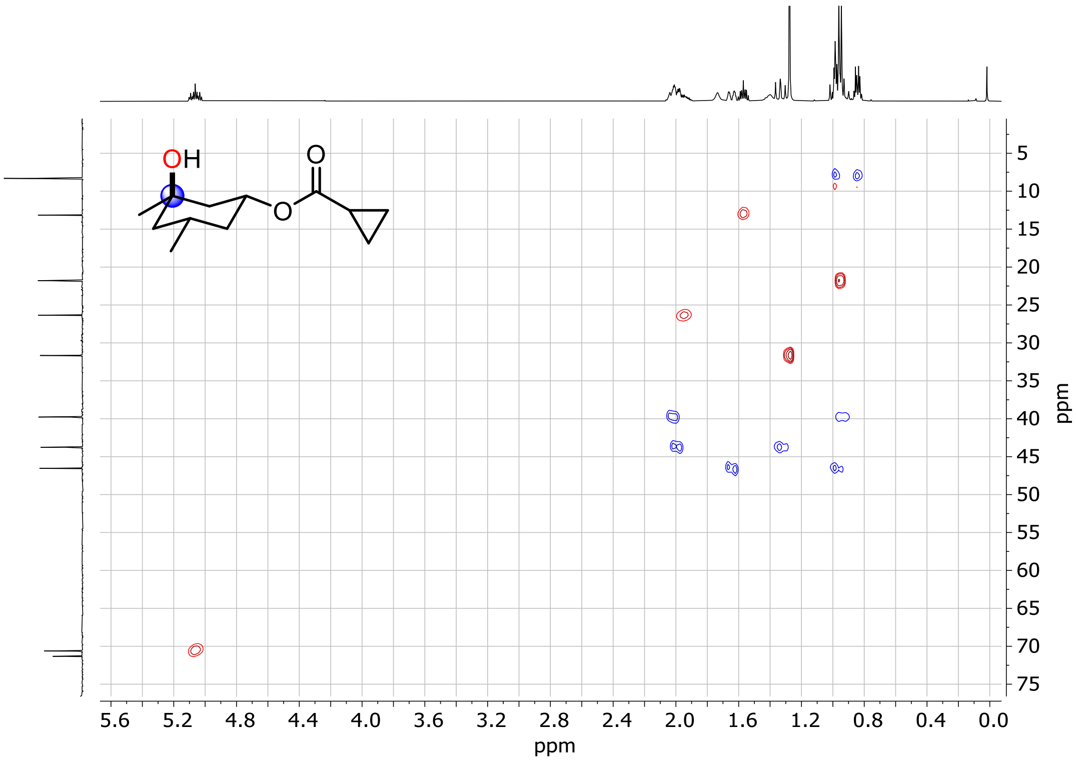

^{1}H-NMR of **4b** in $CDCl_3$

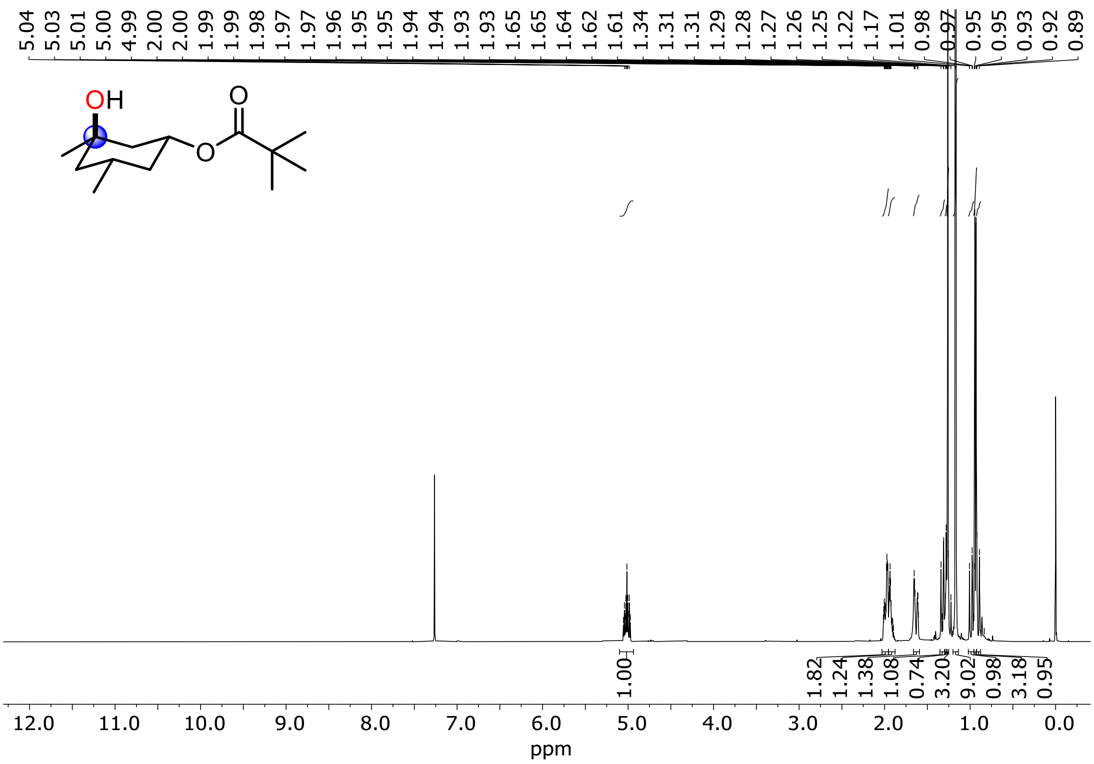

^{13}C-NMR of **4b** in $CDCl_3$

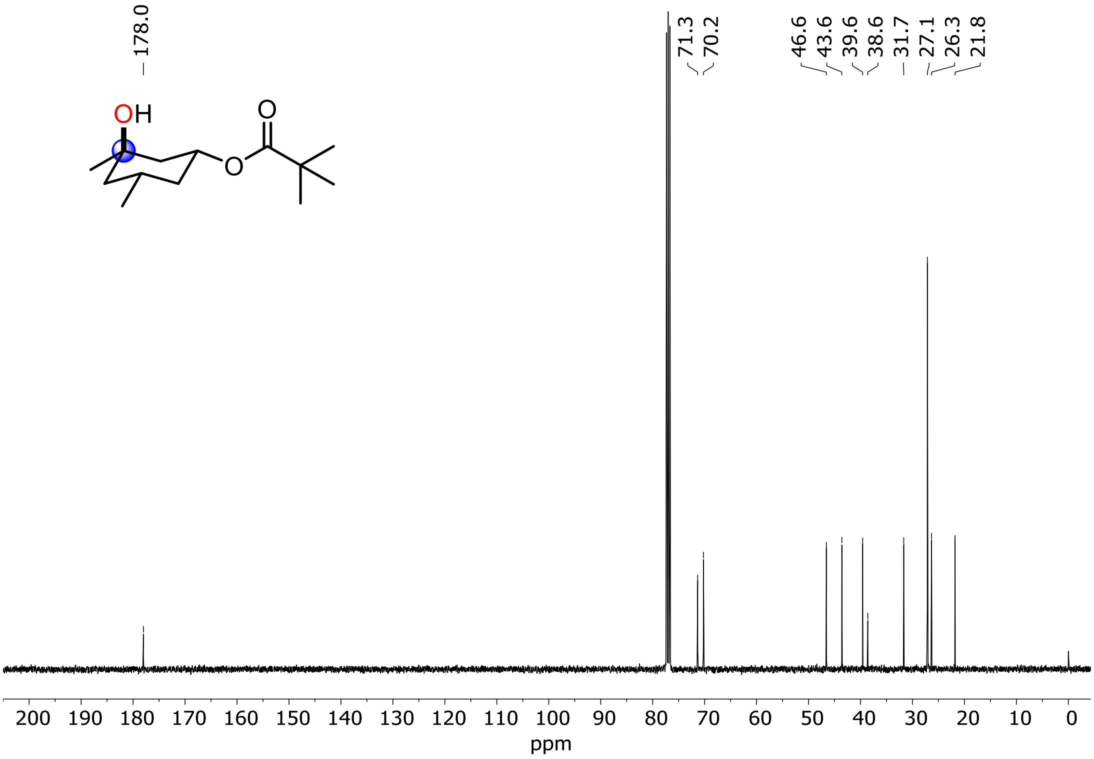

COSY-NMR of **4b** in $CDCl_3$

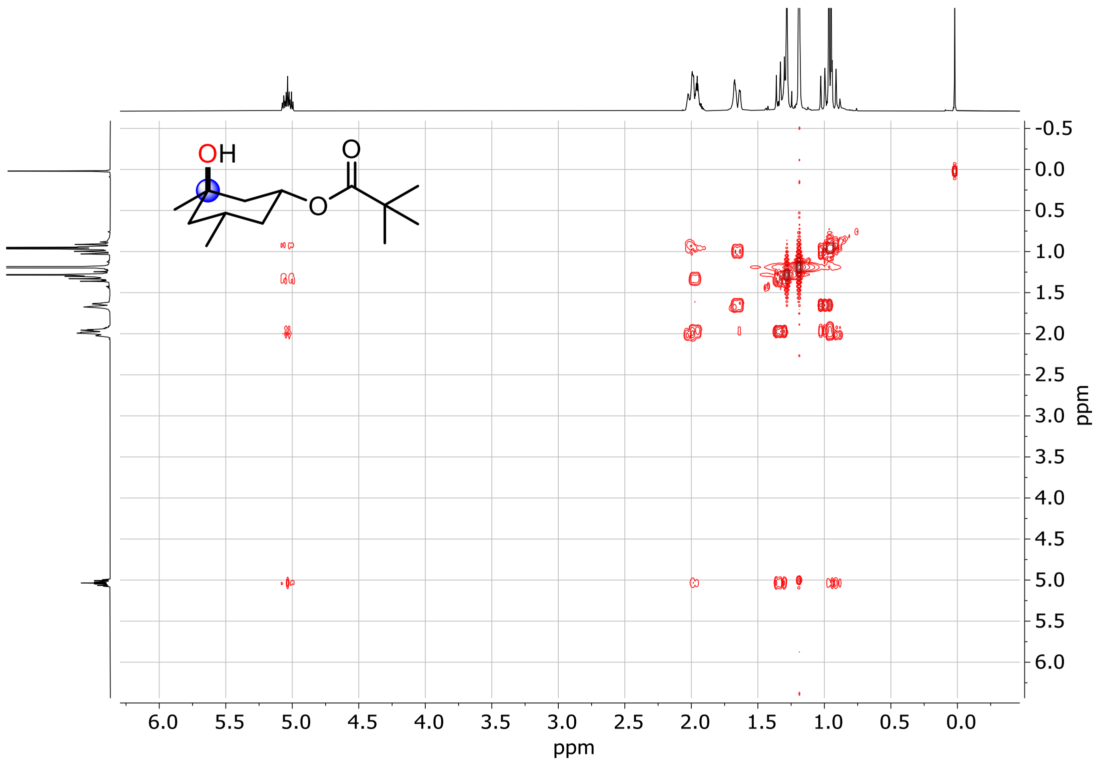

HSQC-NMR of **4b** in $CDCl_3$

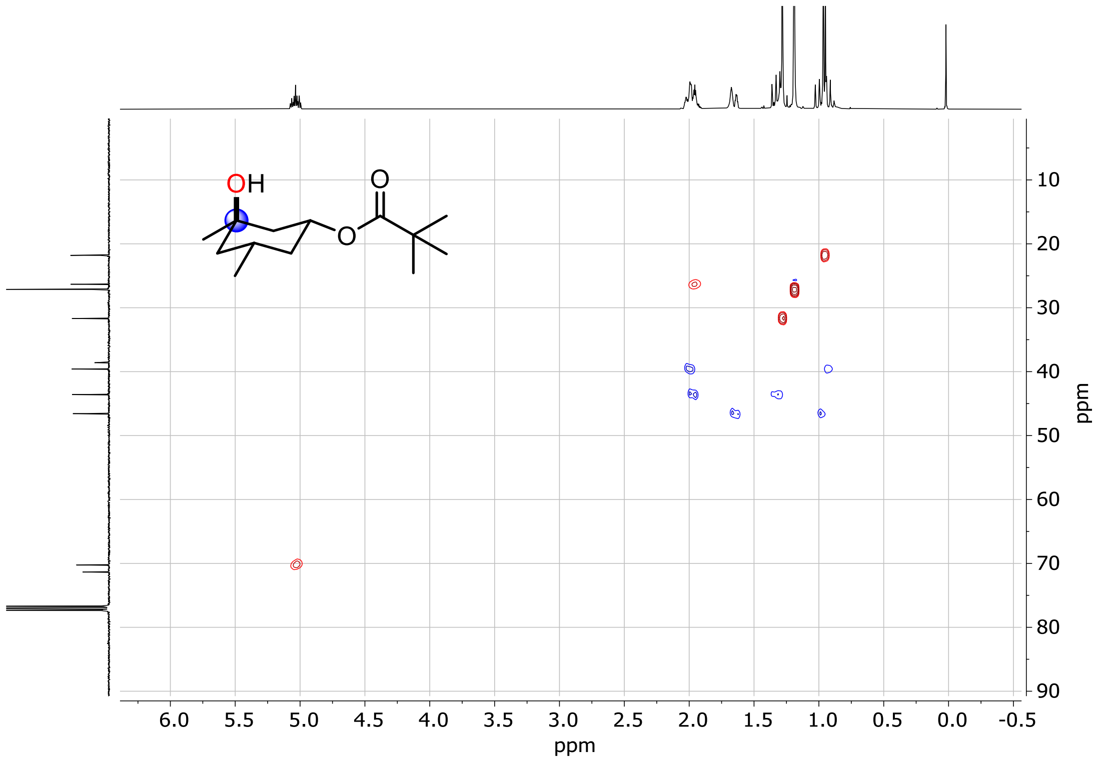

^{1}H-NMR of **5b** in $CDCl_3$

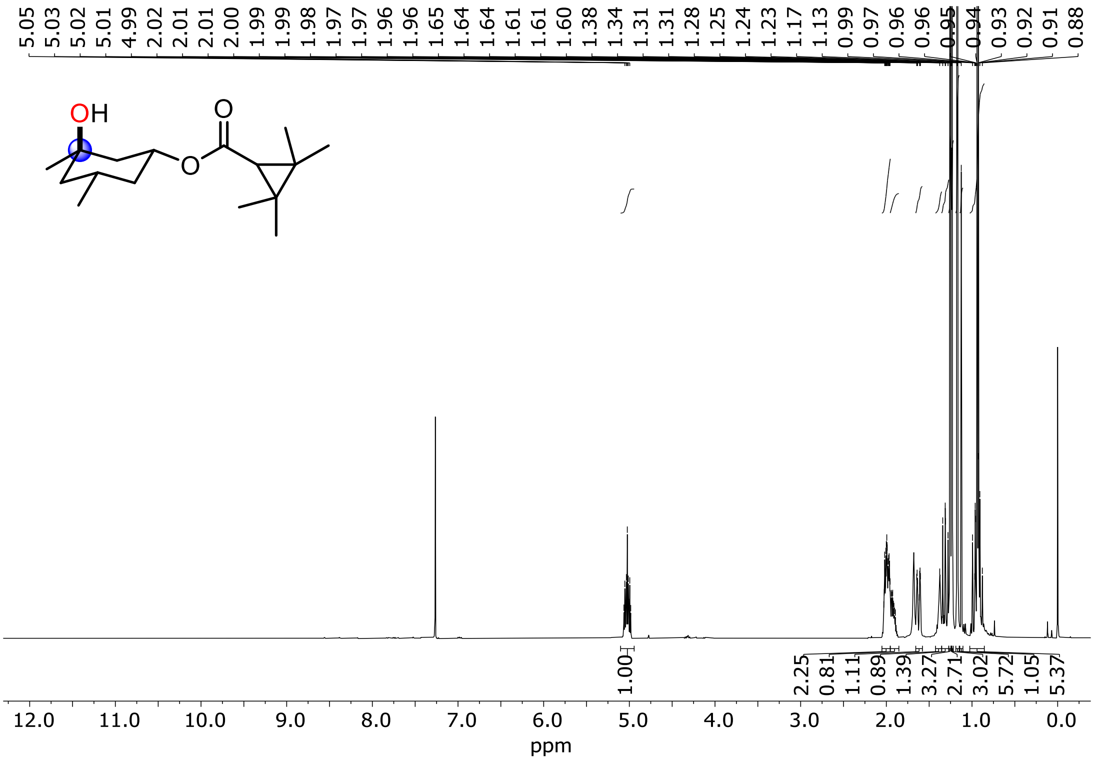

^{13}C-NMR of **5b** in $CDCl_3$

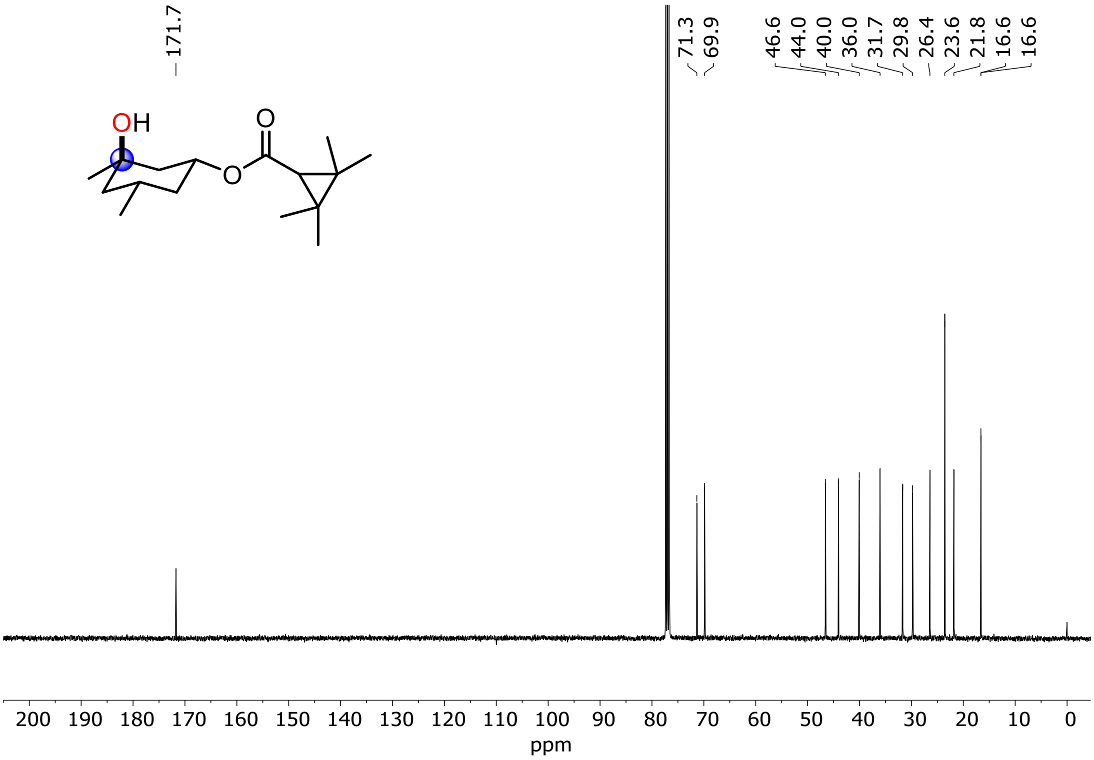

COSY-NMR of **5b** in $CDCl_3$

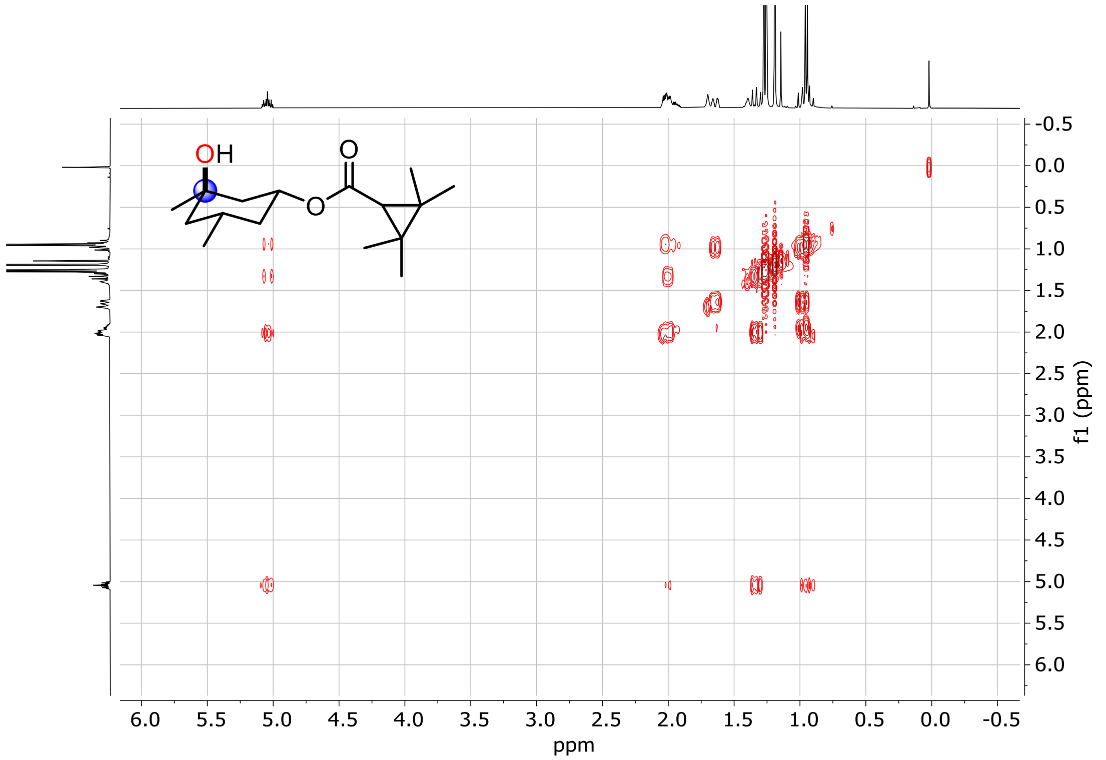

HSQC-NMR of **5b** in $CDCl_3$

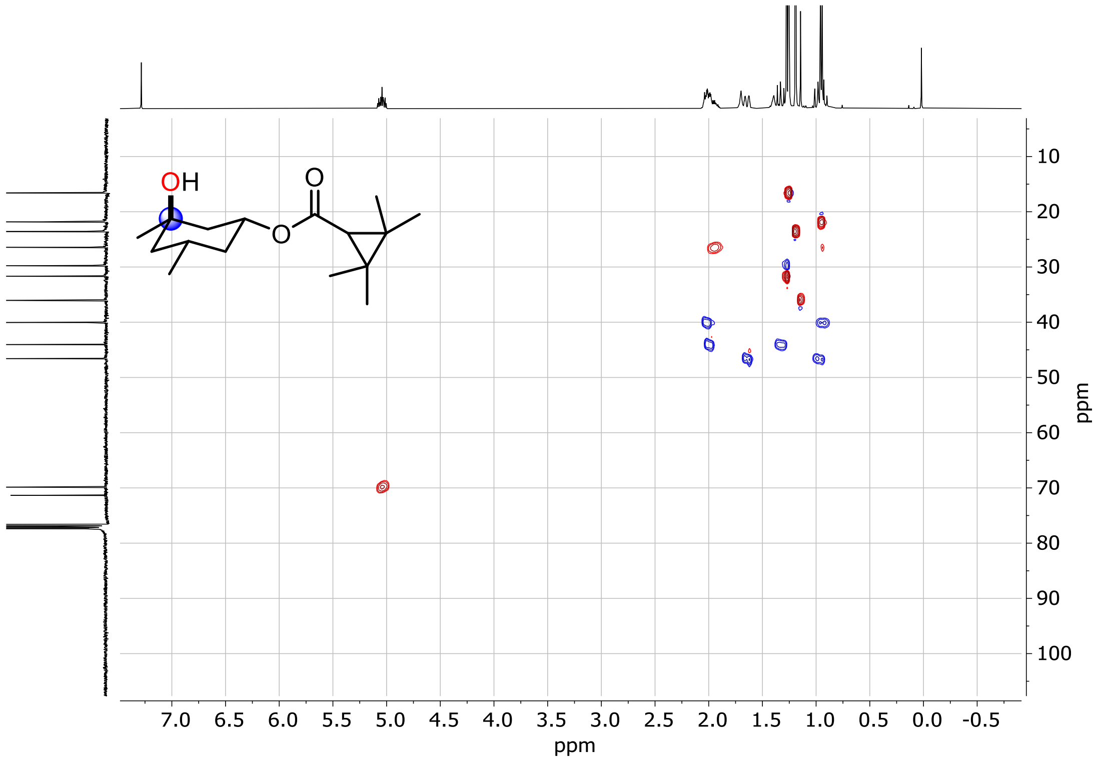

^{1}H-NMR of **6b** in $CDCl_3$

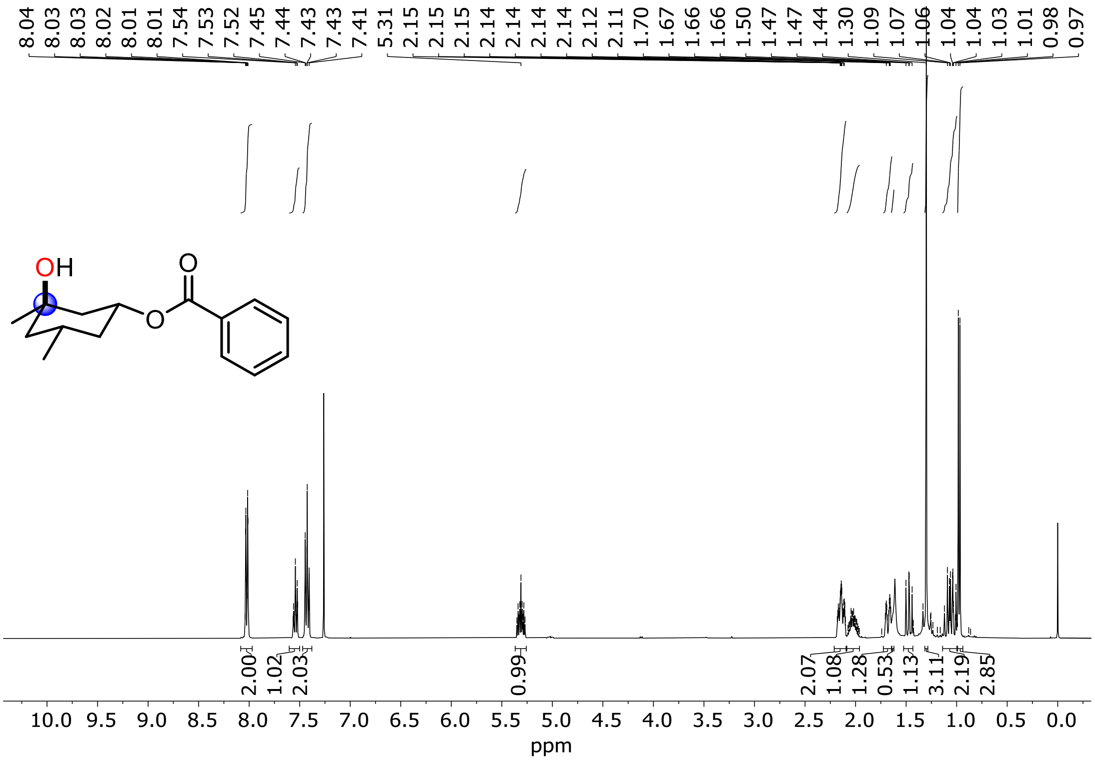

^{13}C-NMR of **6b** in $CDCl_3$

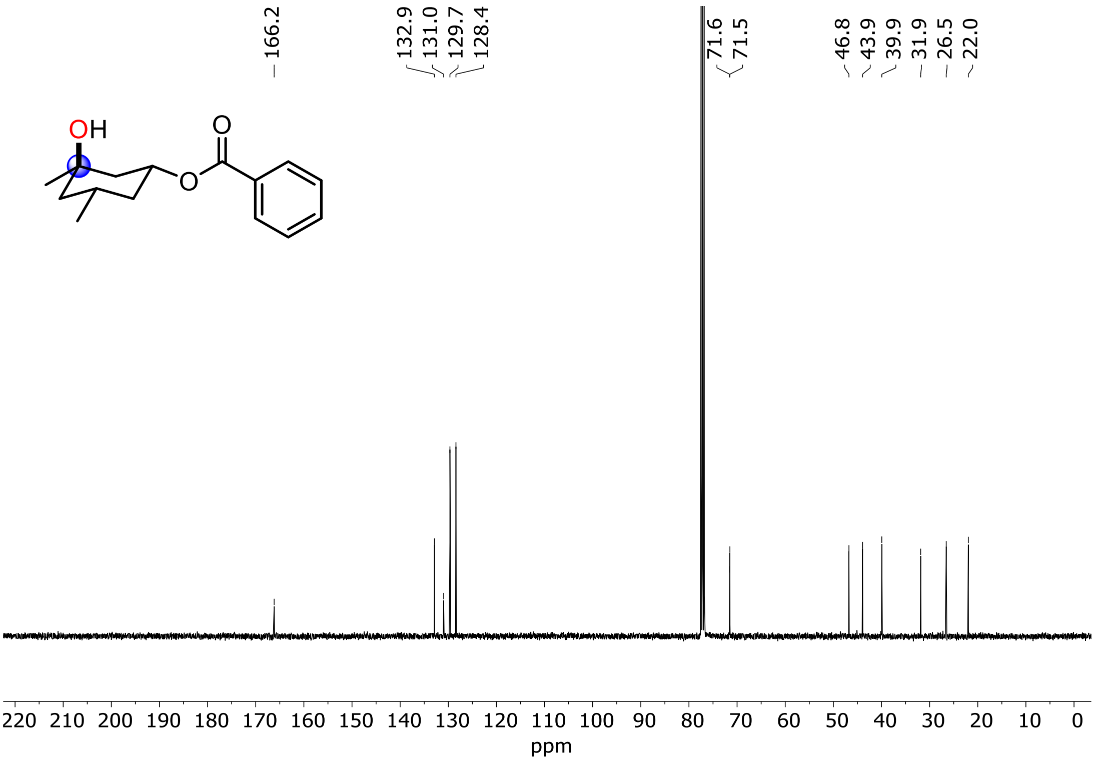

COSY-NMR of **6b** in $CDCl_3$

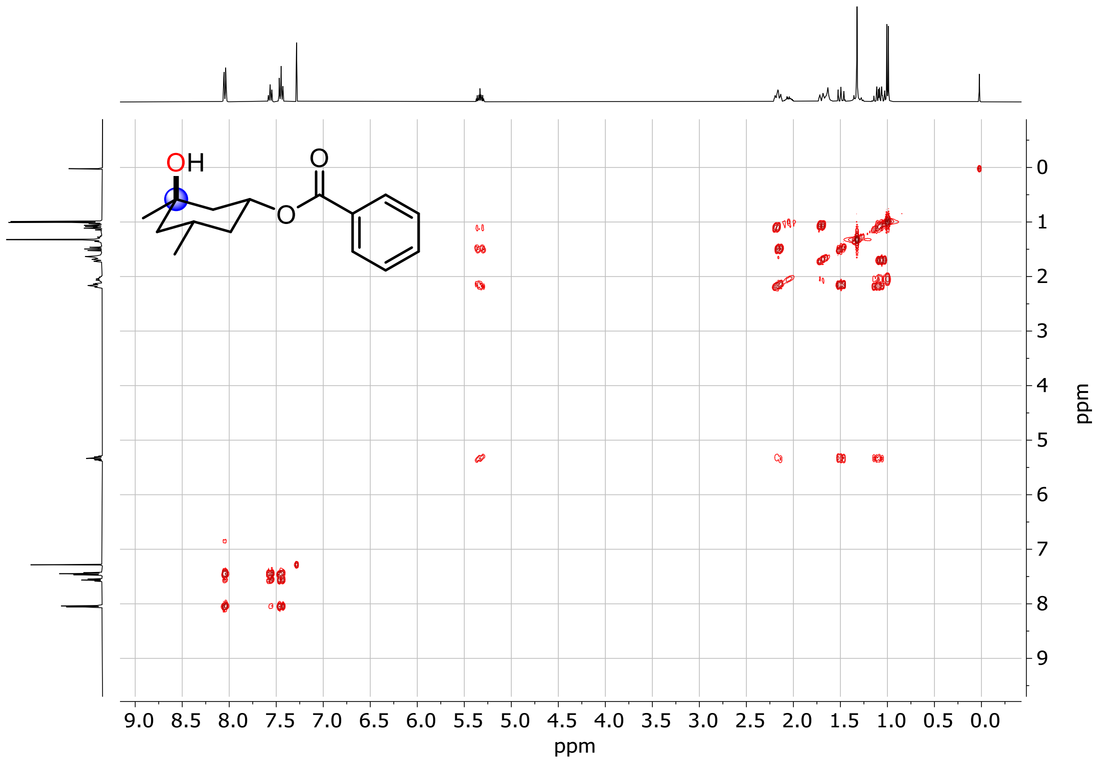

HSQC-NMR of **6b** in $CDCl_3$

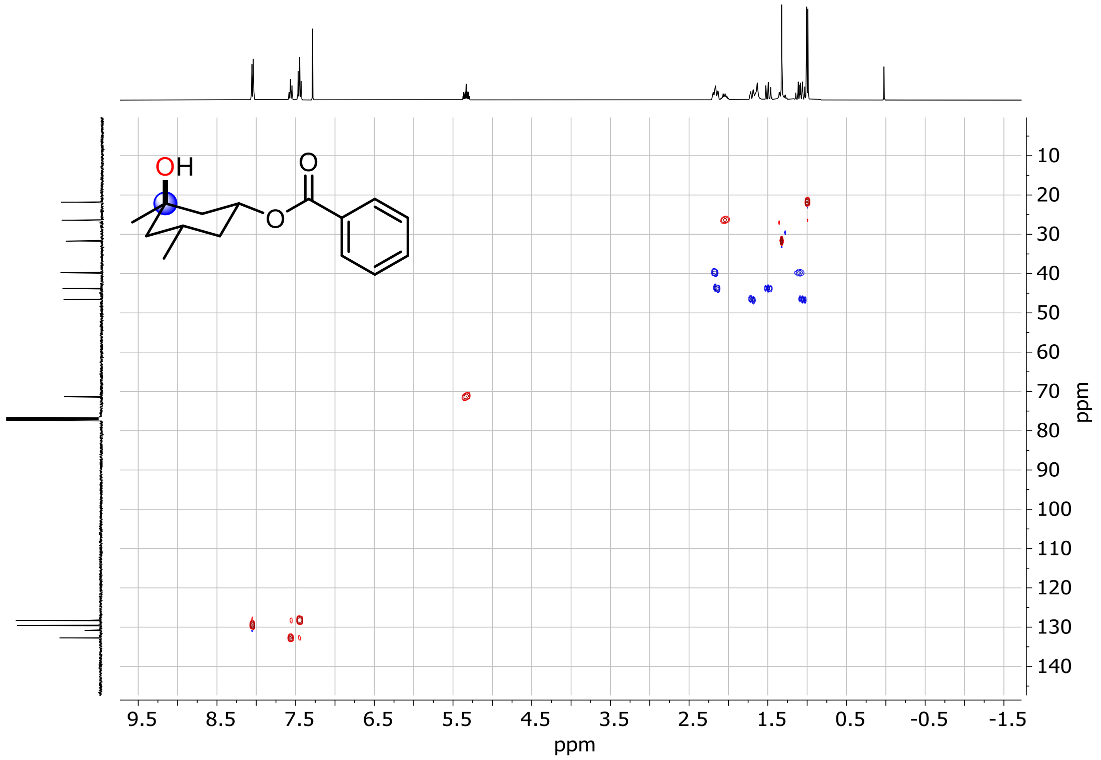

^{1}H-NMR of **7b** in $CDCl_3$

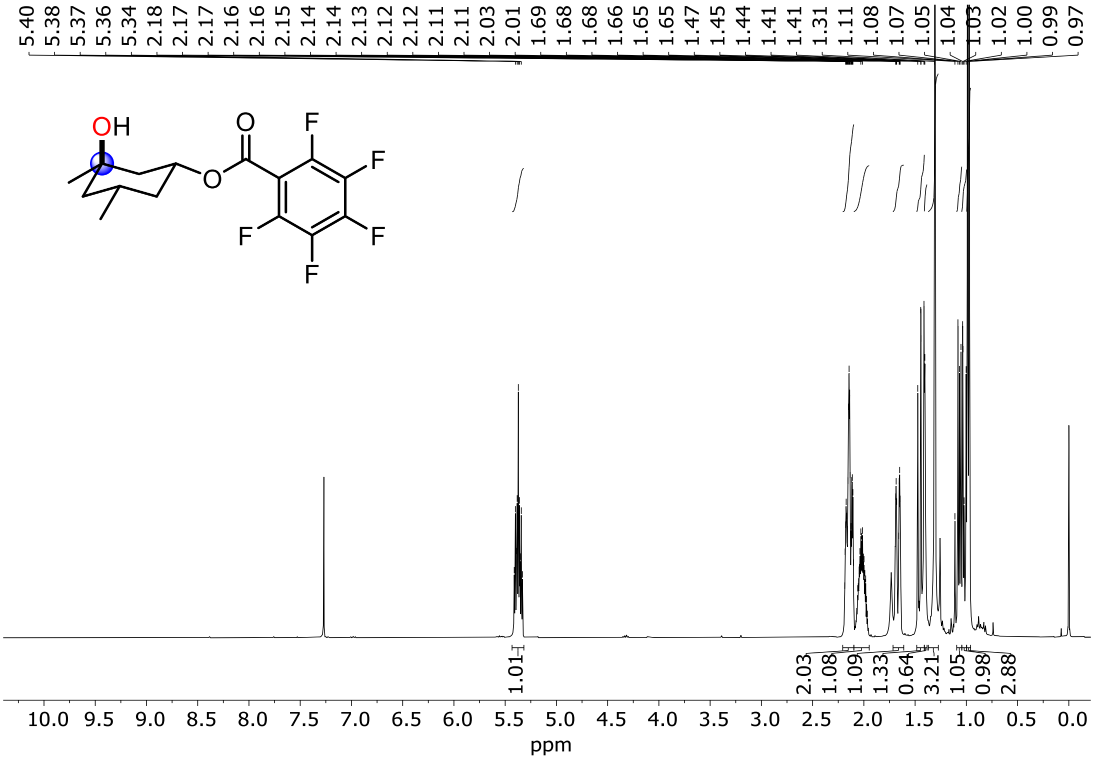

^{13}C-NMR of **7b** in $CDCl_3$

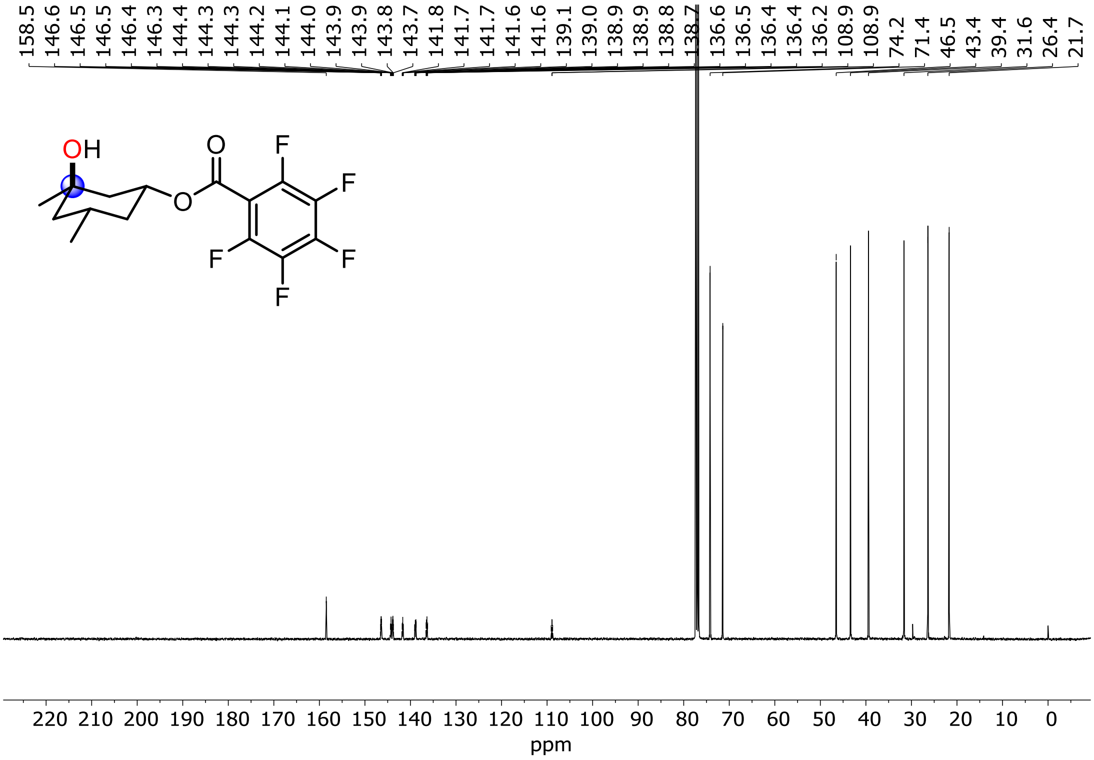

^{19}F-NMR of **7b** in $CDCl_3$

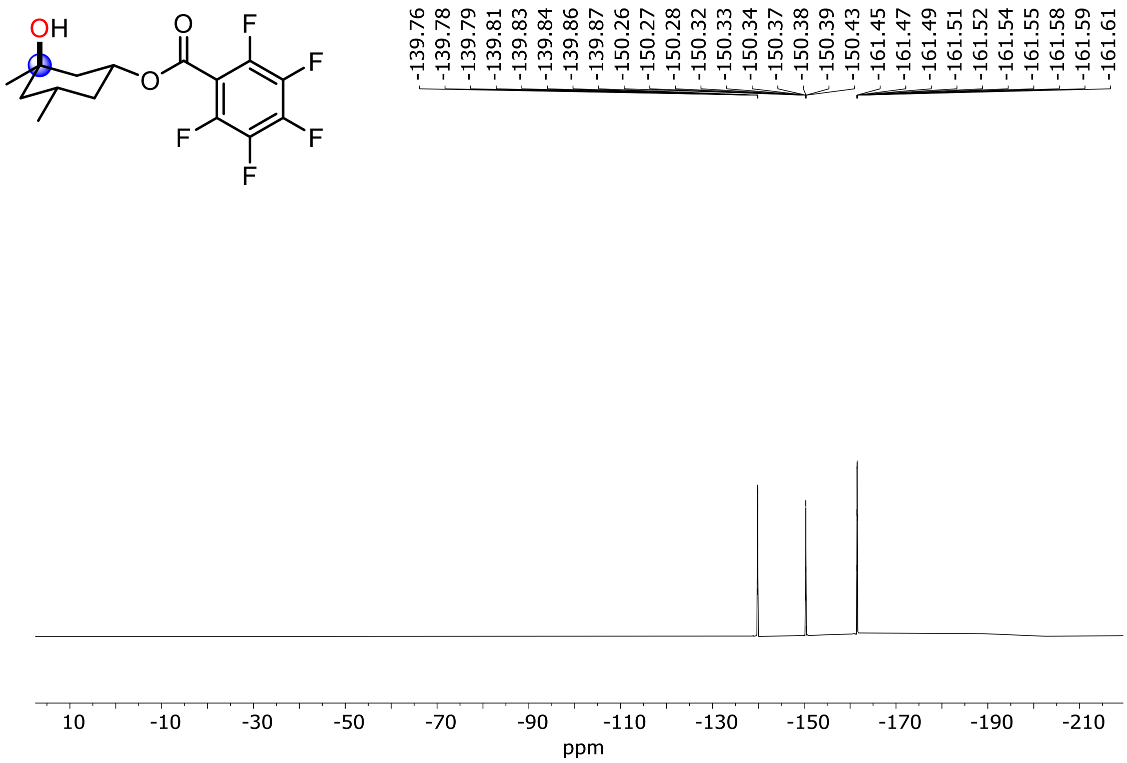

COSY-NMR of **7b** in $CDCl_3$

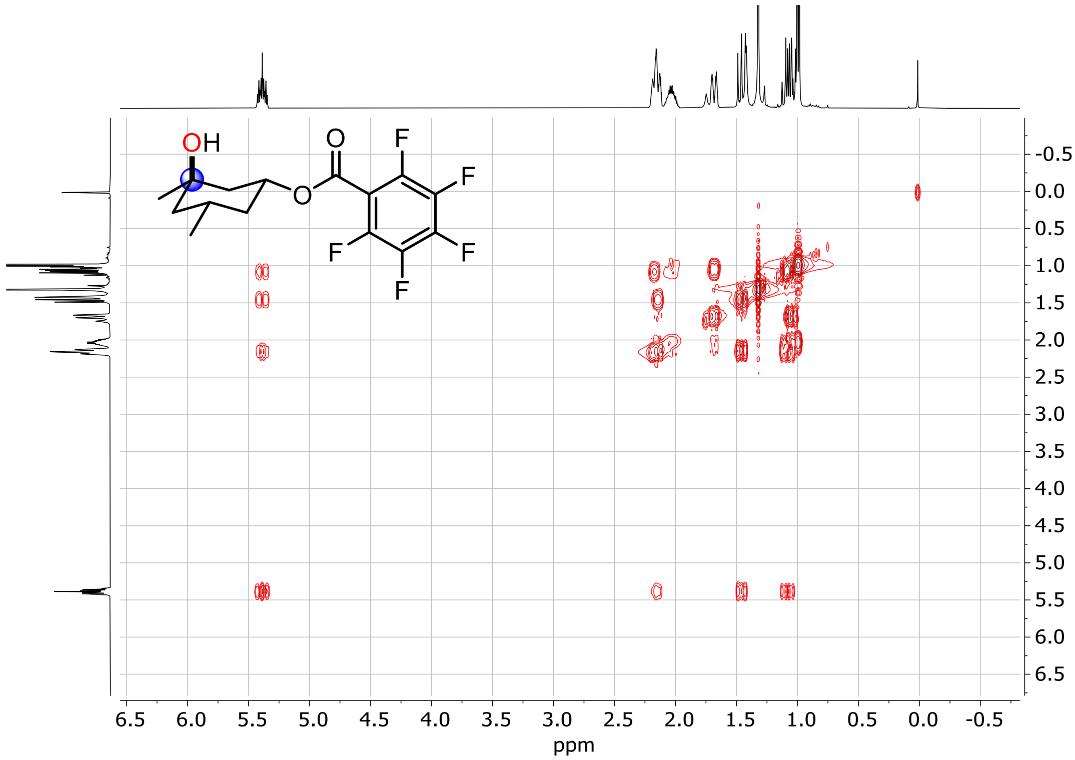

HSQC-NMR of **7b** in $CDCl_3$

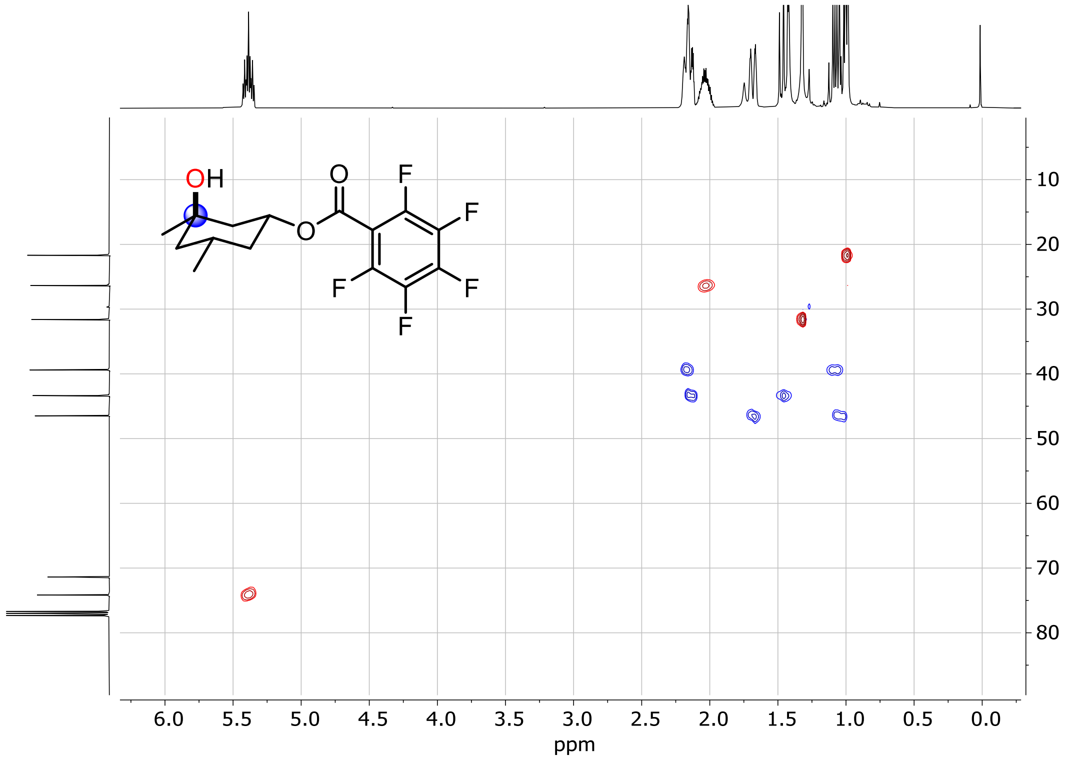

^{1}H-NMR of **8b** in $CDCl_3$

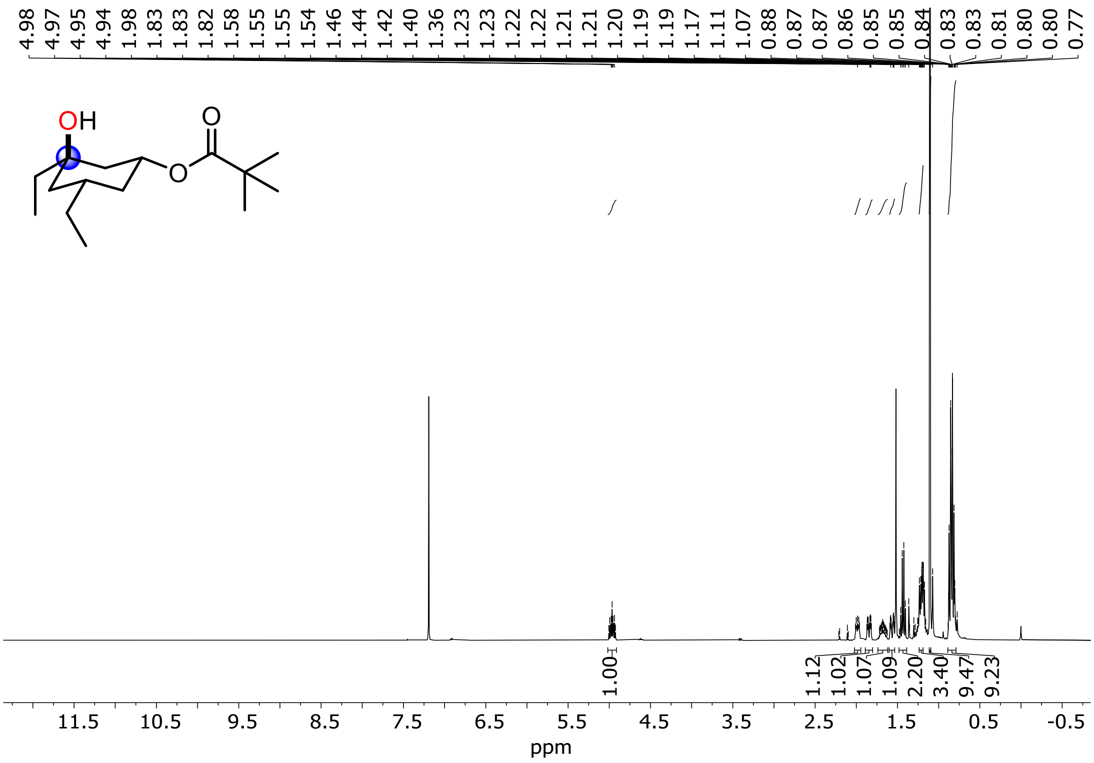

^{13}C-NMR of **8b** in $CDCl_3$

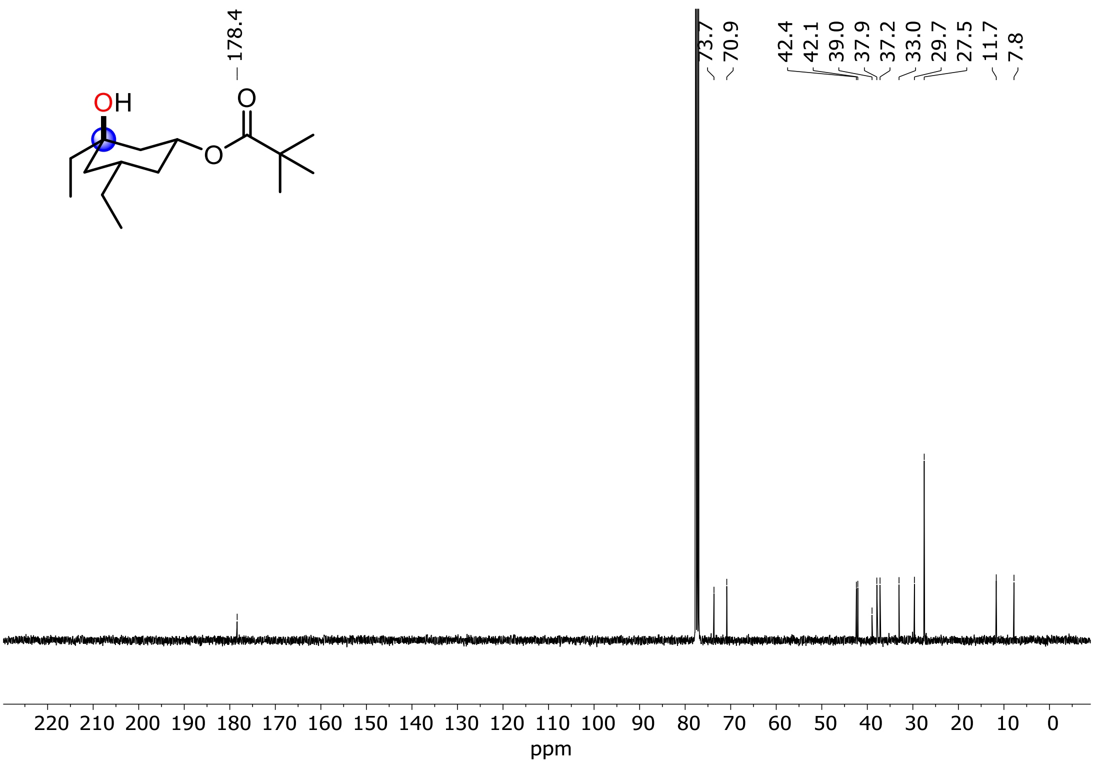

COSY-NMR of **8b** in $CDCl_3$

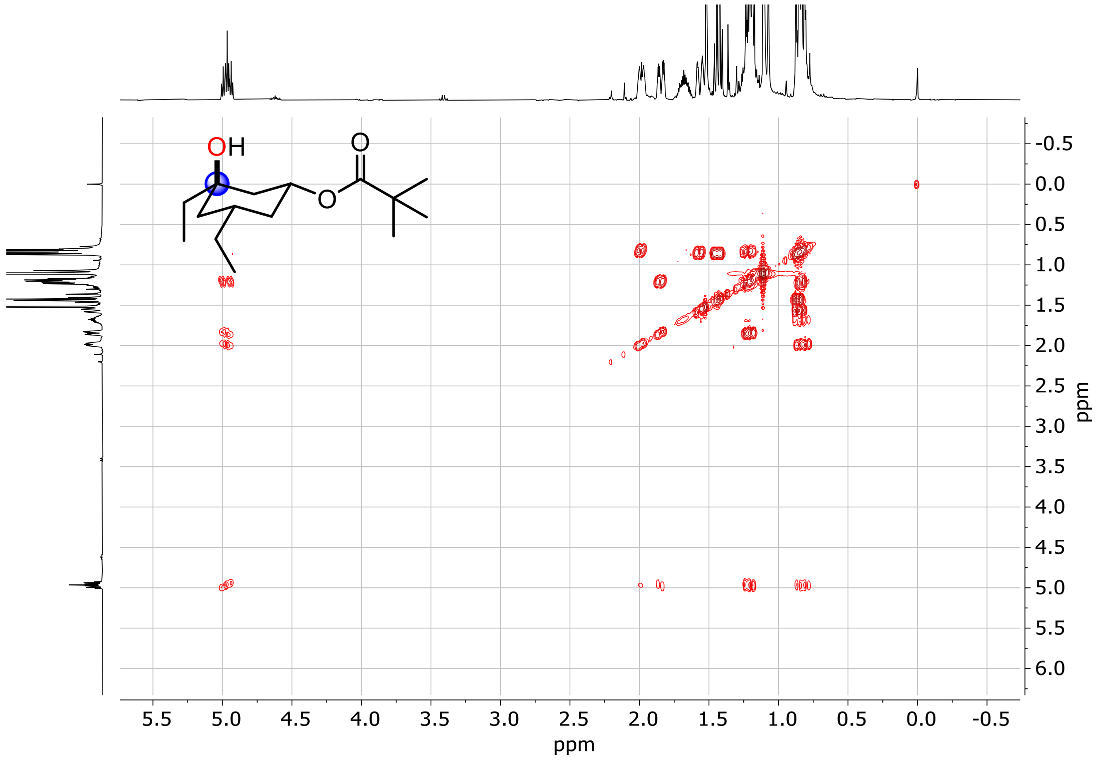

HSQC-NMR of **8b** in $CDCl_3$

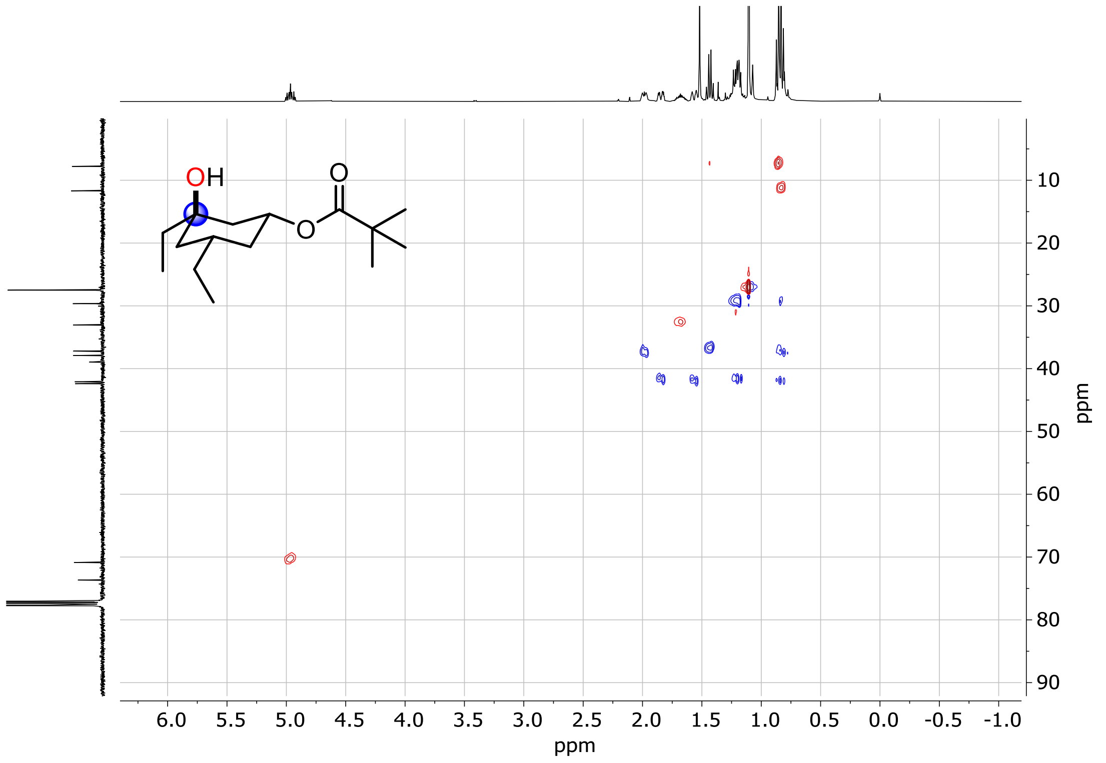

^{1}H-NMR of **8c** in $CDCl_3$

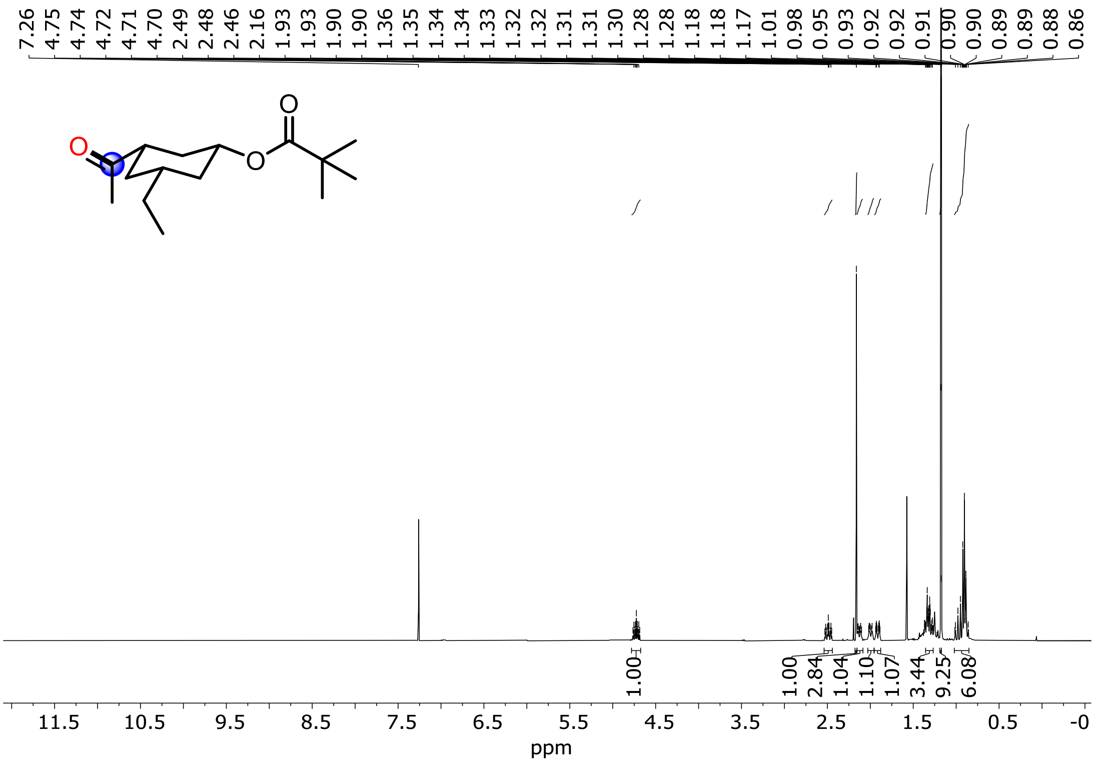

^{13}C-NMR of **8c** in $CDCl_3$

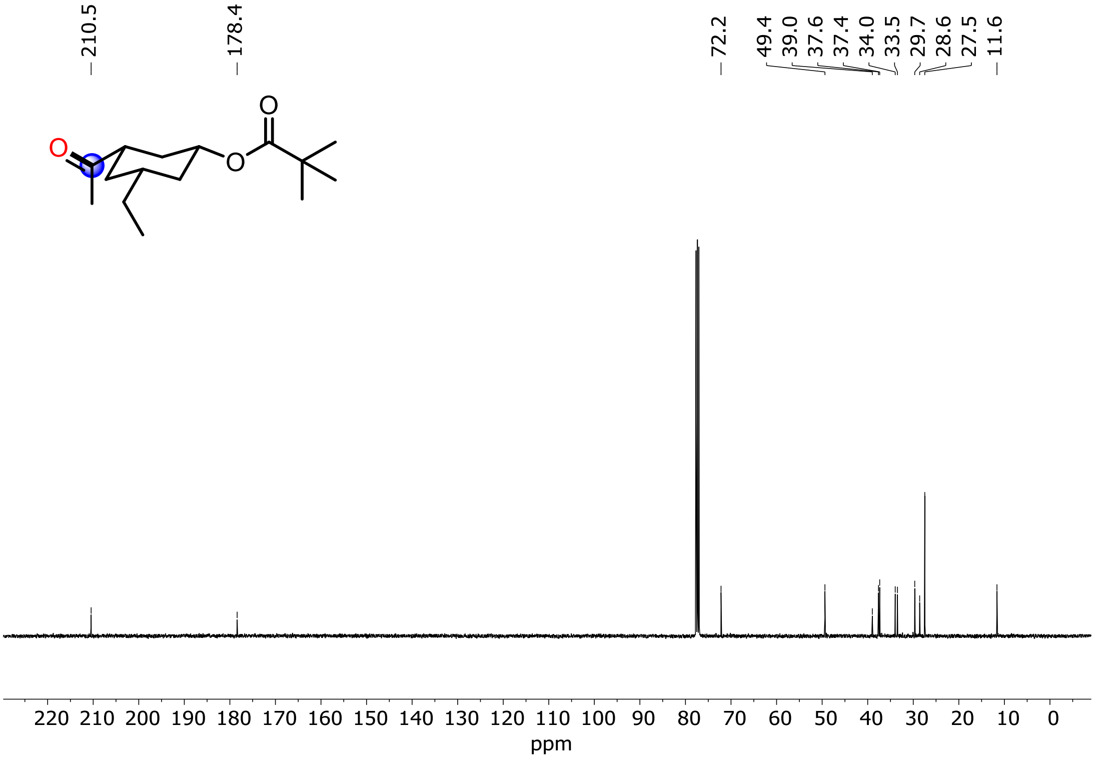

COSY-NMR of **8c** in $CDCl_3$

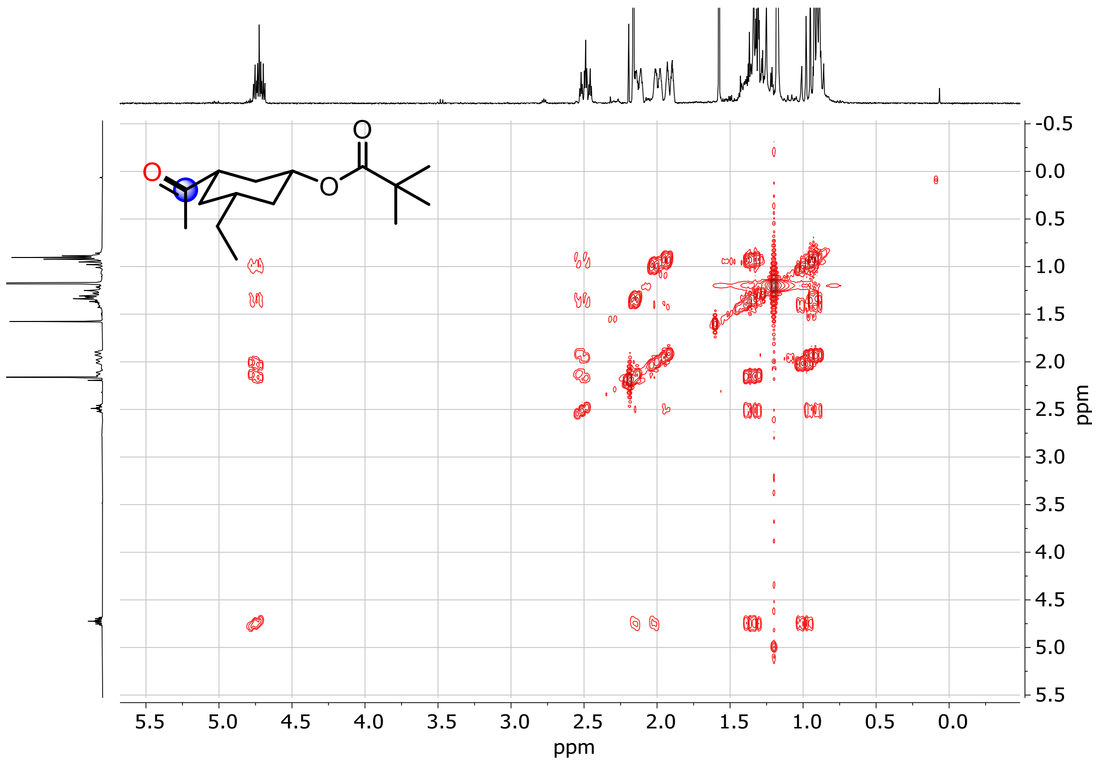

HSQC-NMR of **8c** in $CDCl_3$

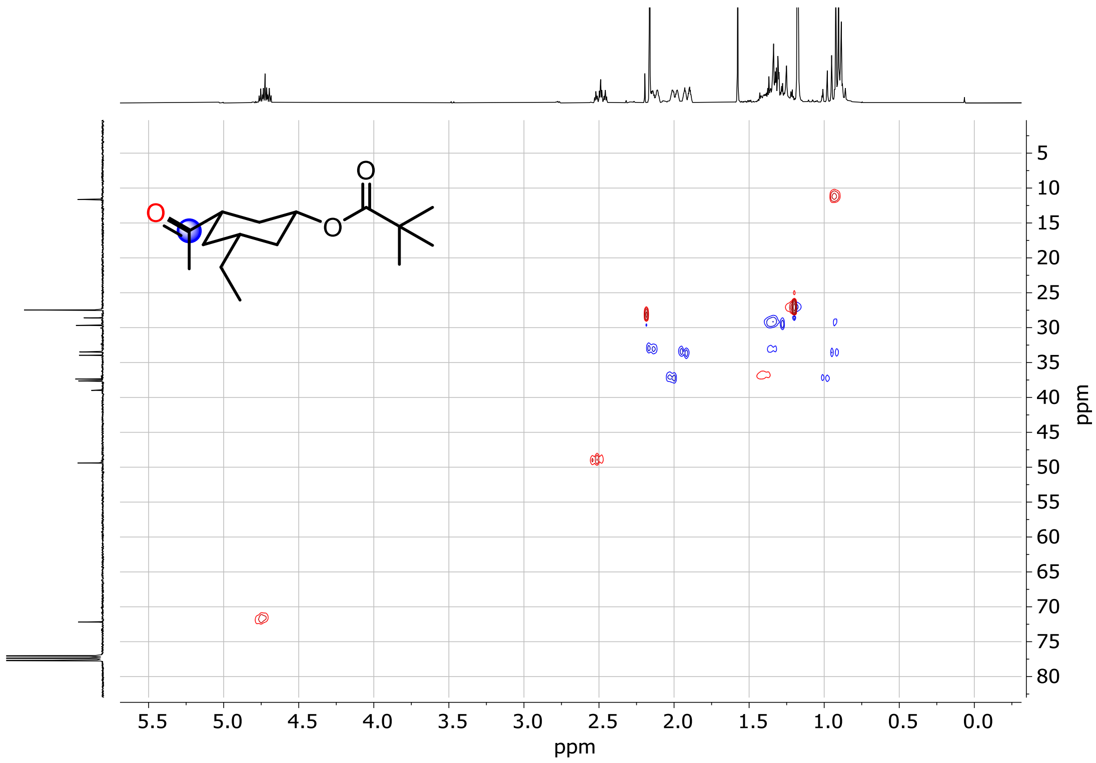

^{1}H-NMR of **10b** in $CDCl_3$

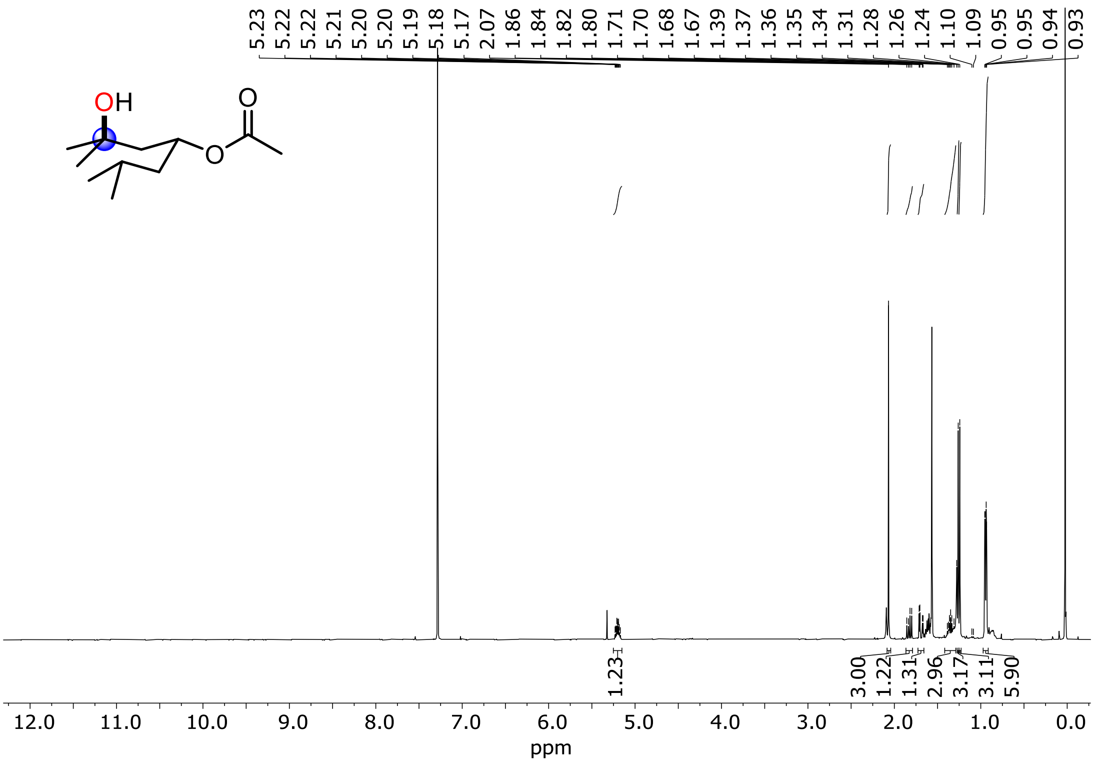

^{13}C-NMR of **10b** in $CDCl_3$

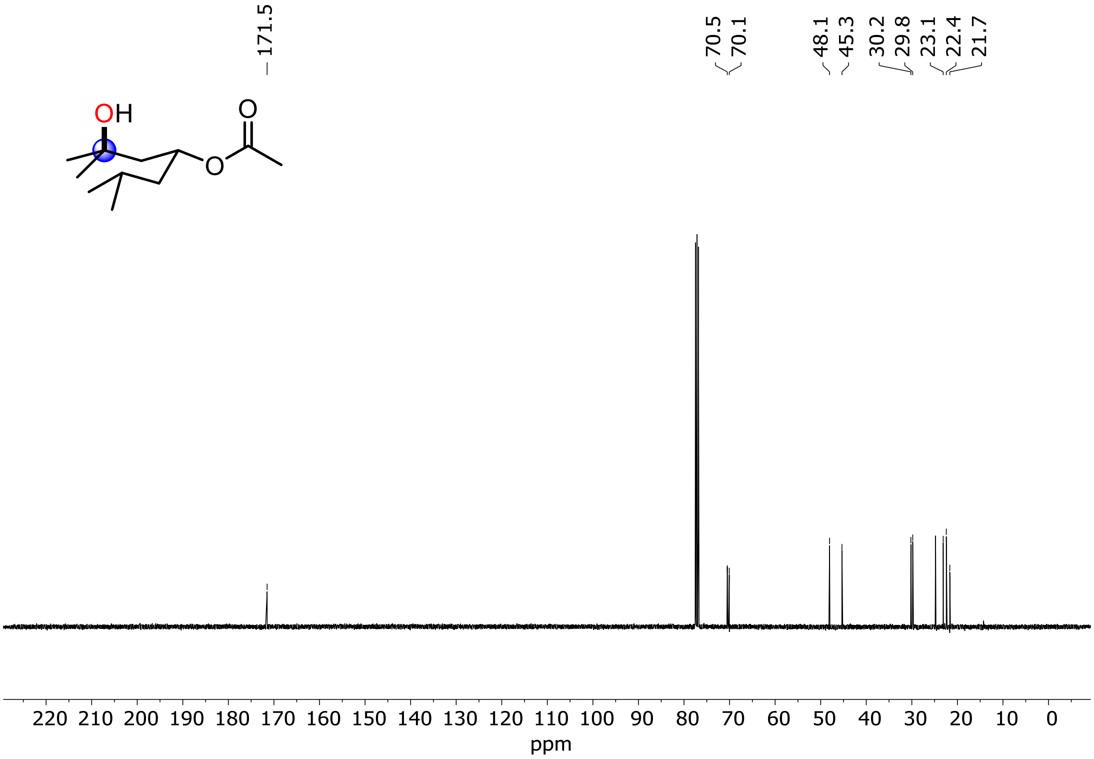

^{1}H-NMR of **11b** in $CDCl_3$

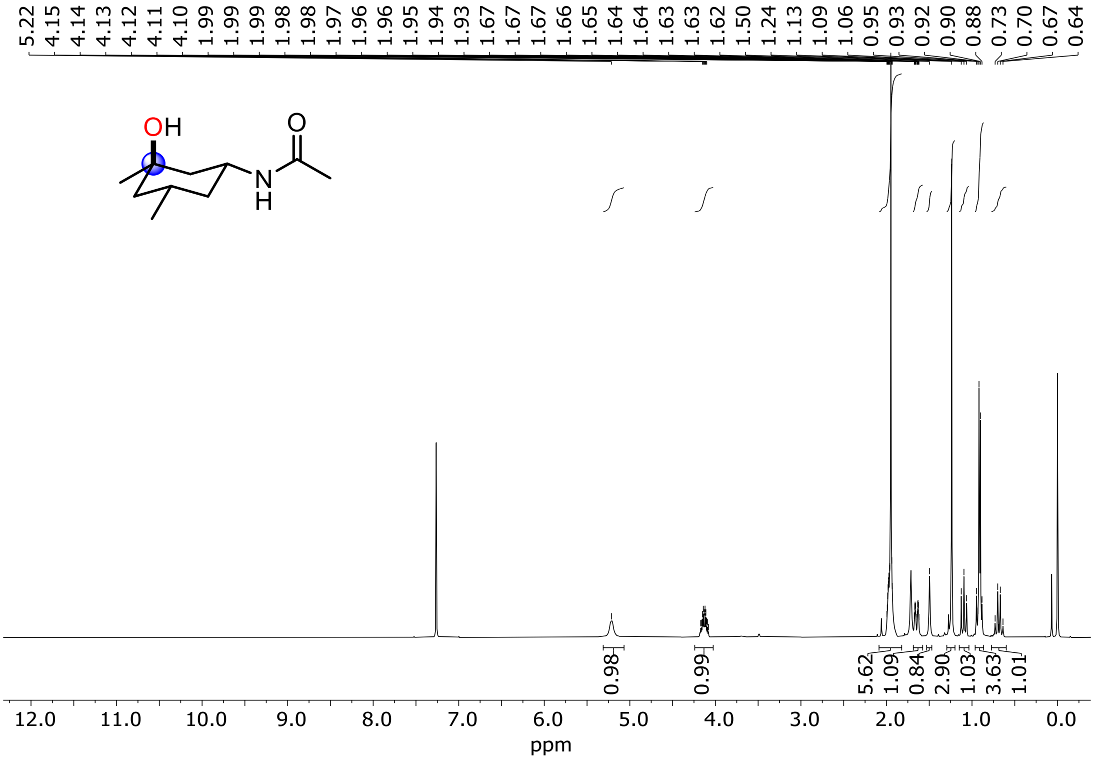

^{13}C-NMR of **11b** in $CDCl_3$

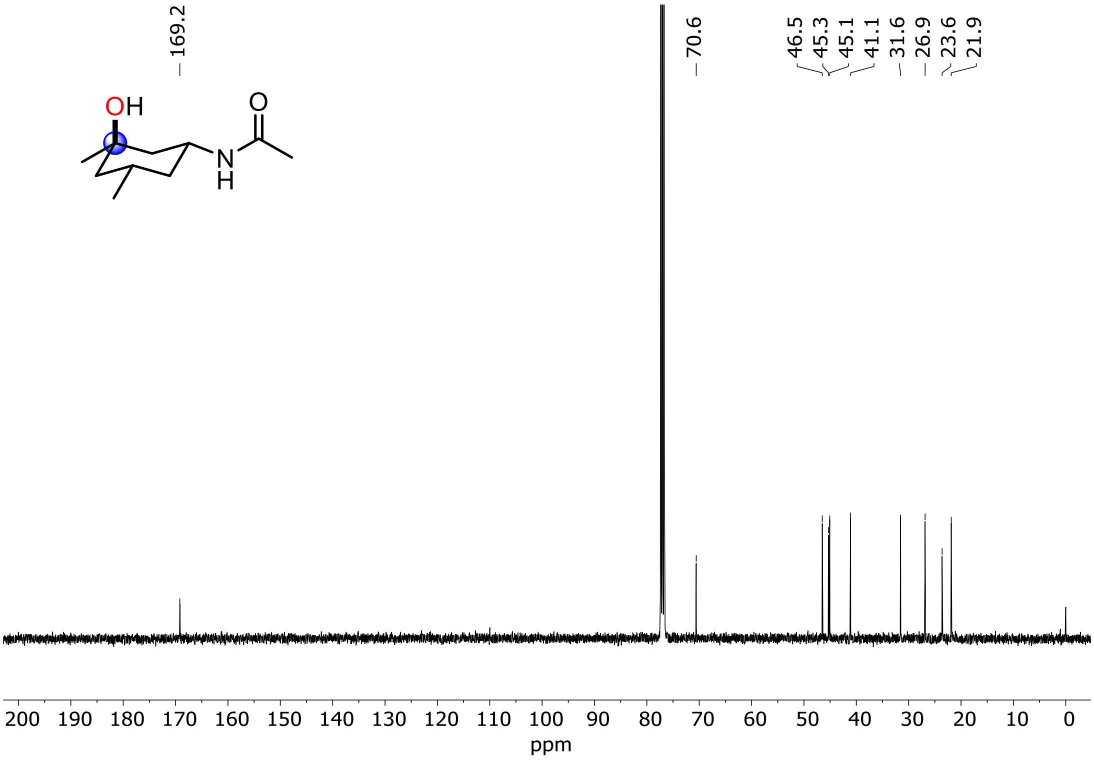

COSY-NMR of **11b** in $CDCl_3$

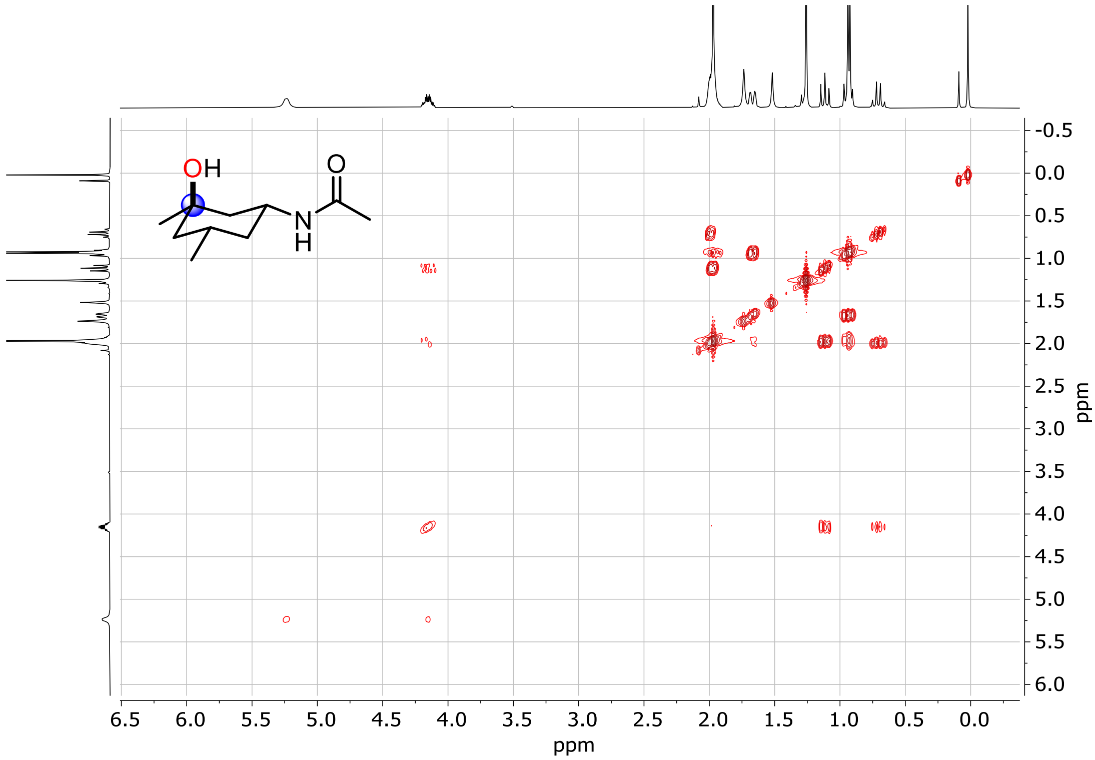

HSQC-NMR of **11b** in $CDCl_3$

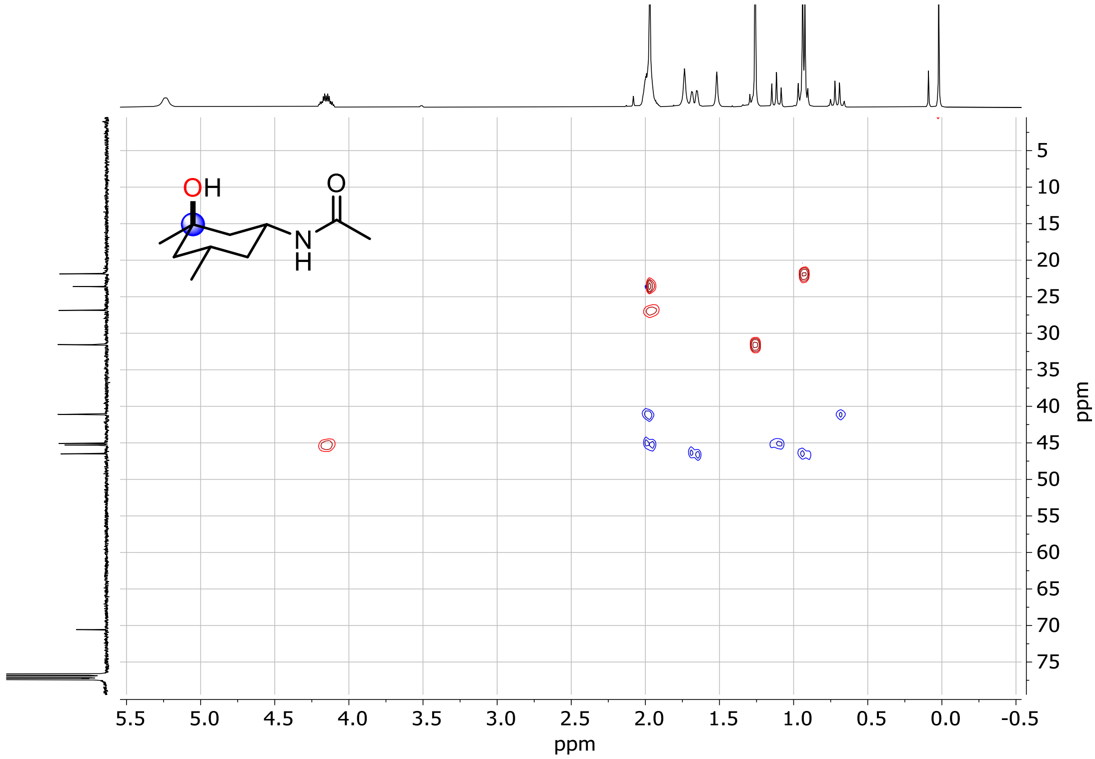

^{1}H-NMR of **12b** in $CDCl_3$

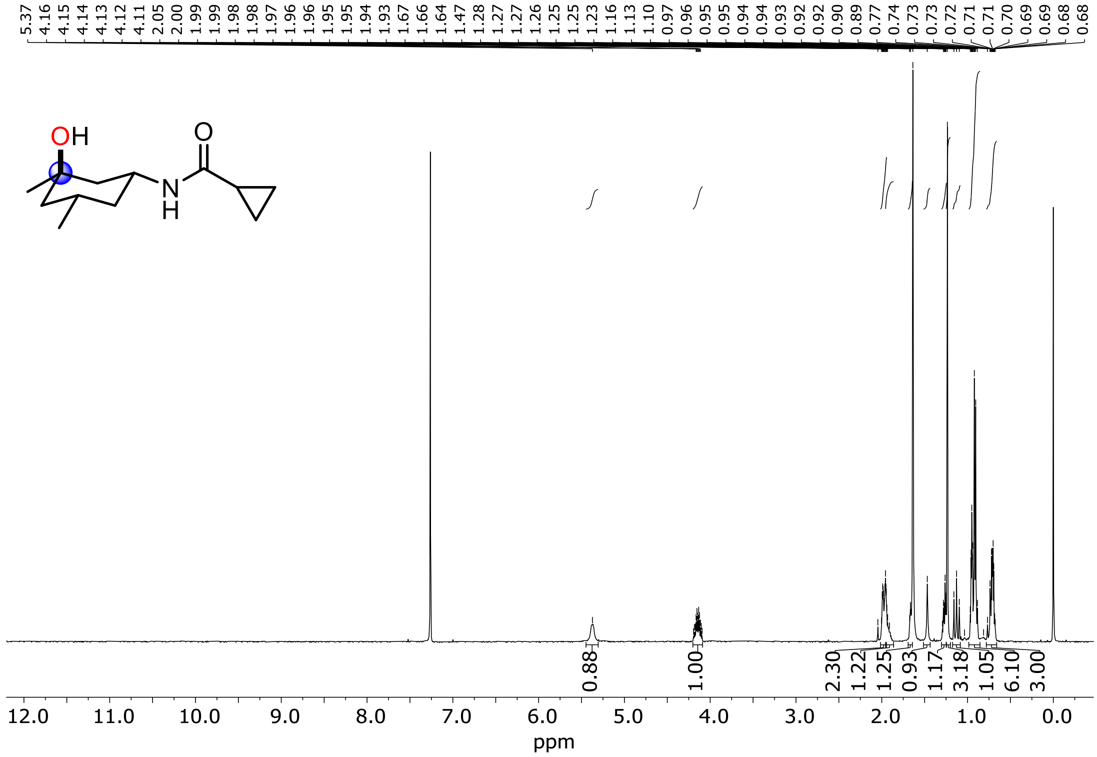

^{13}C-NMR of **12b** in $CDCl_3$

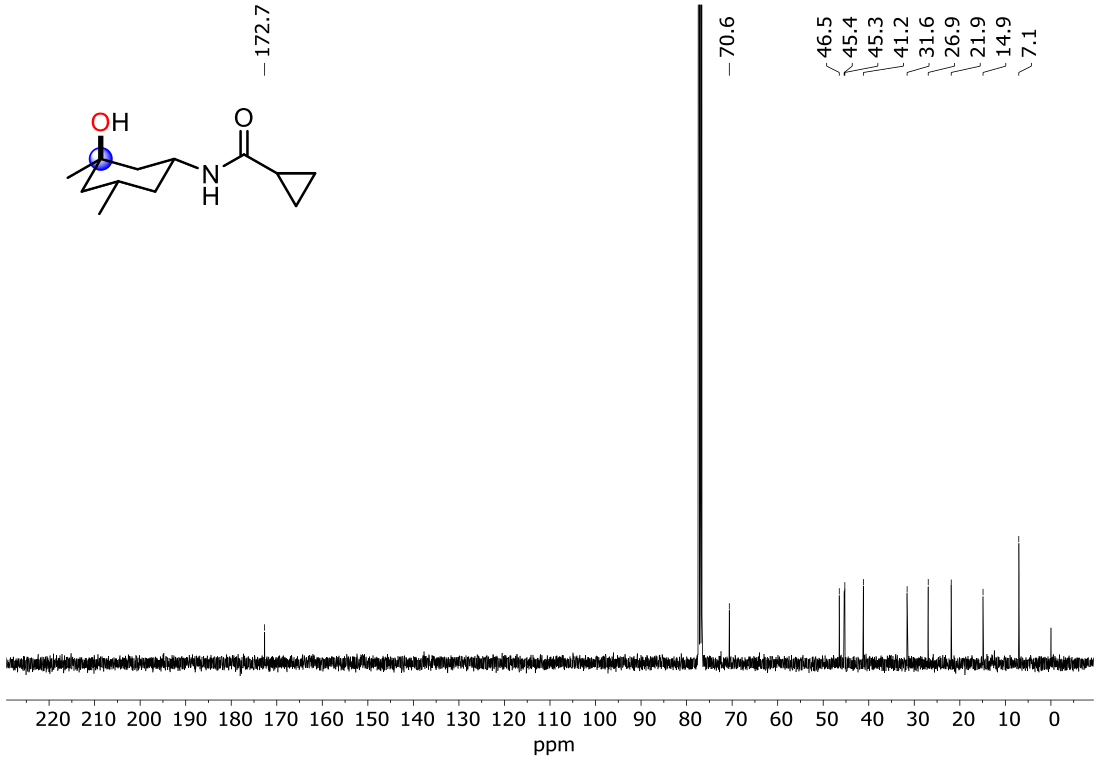

COSY-NMR of **12b** in $CDCl_3$

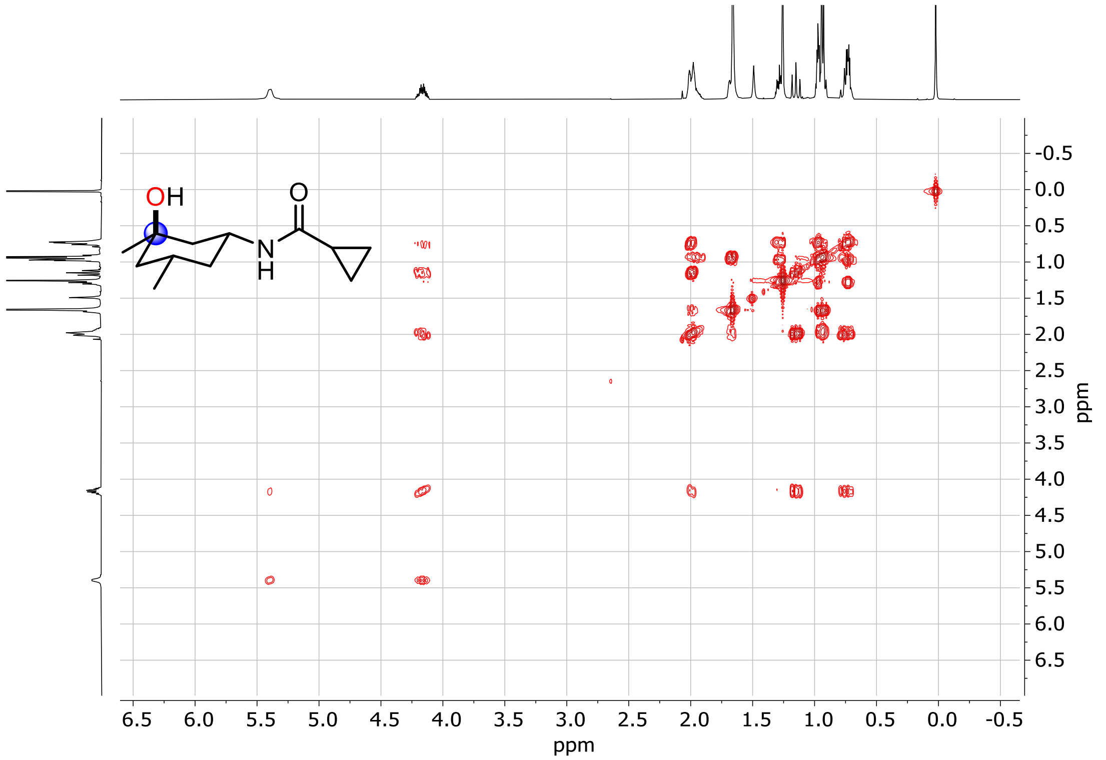

HSQC-NMR of **12b** in $CDCl_3$

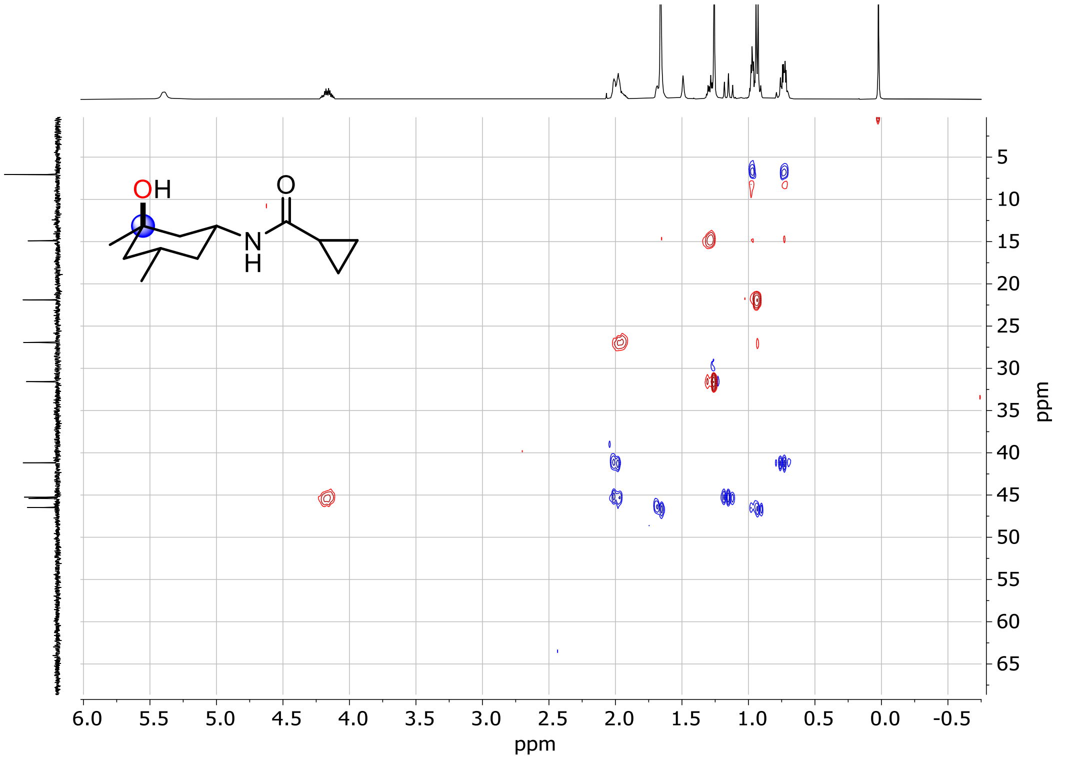

^{1}H-NMR of **13b** in $CDCl_3$

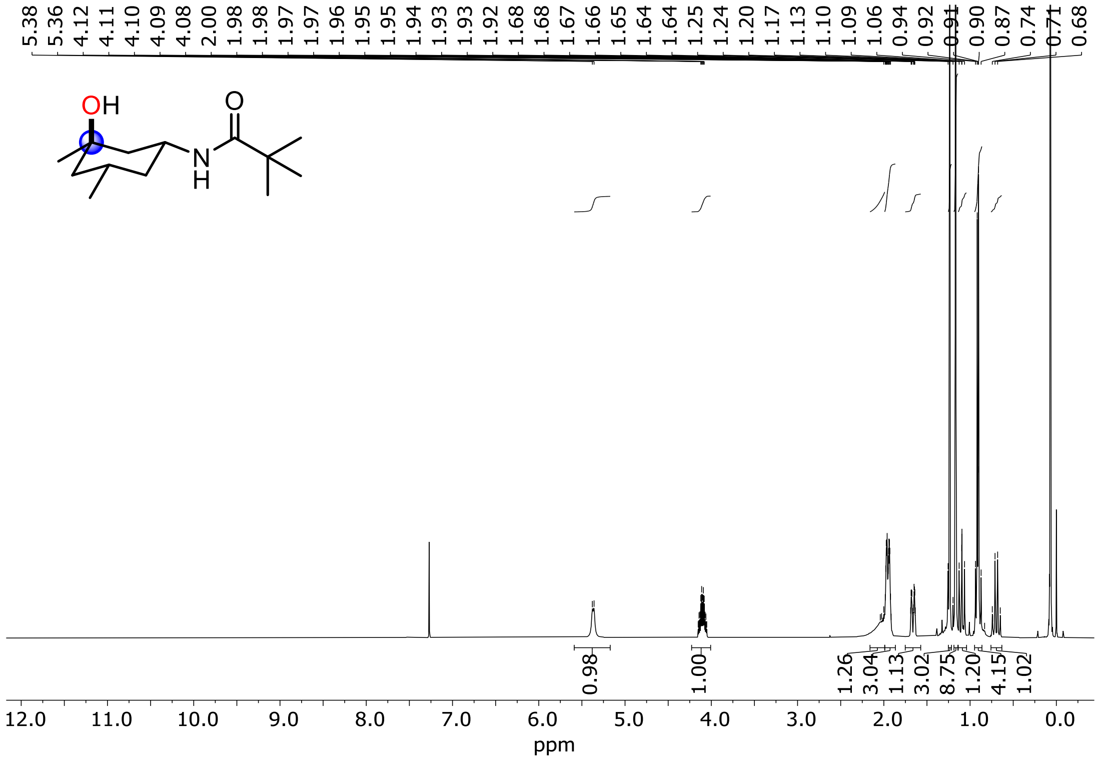

^{13}C-NMR of **13b** in $CDCl_3$

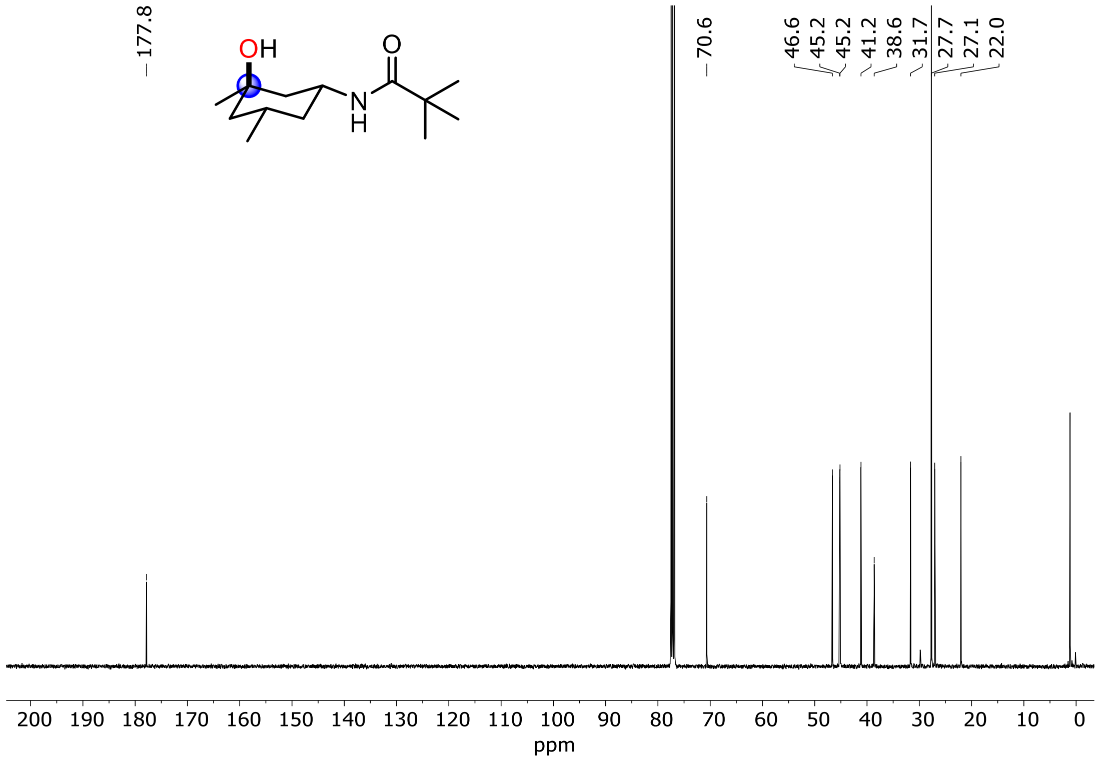

COSY-NMR of **13b** in $CDCl_3$

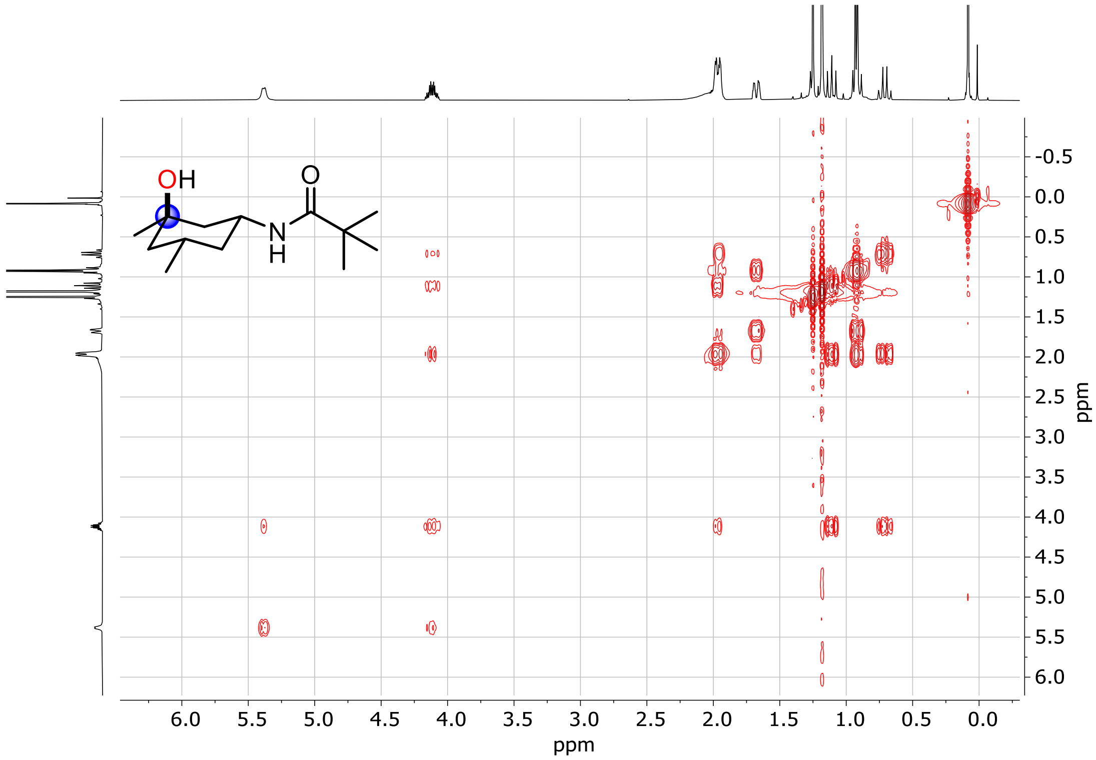

HSQC-NMR of **13b** in $CDCl_3$

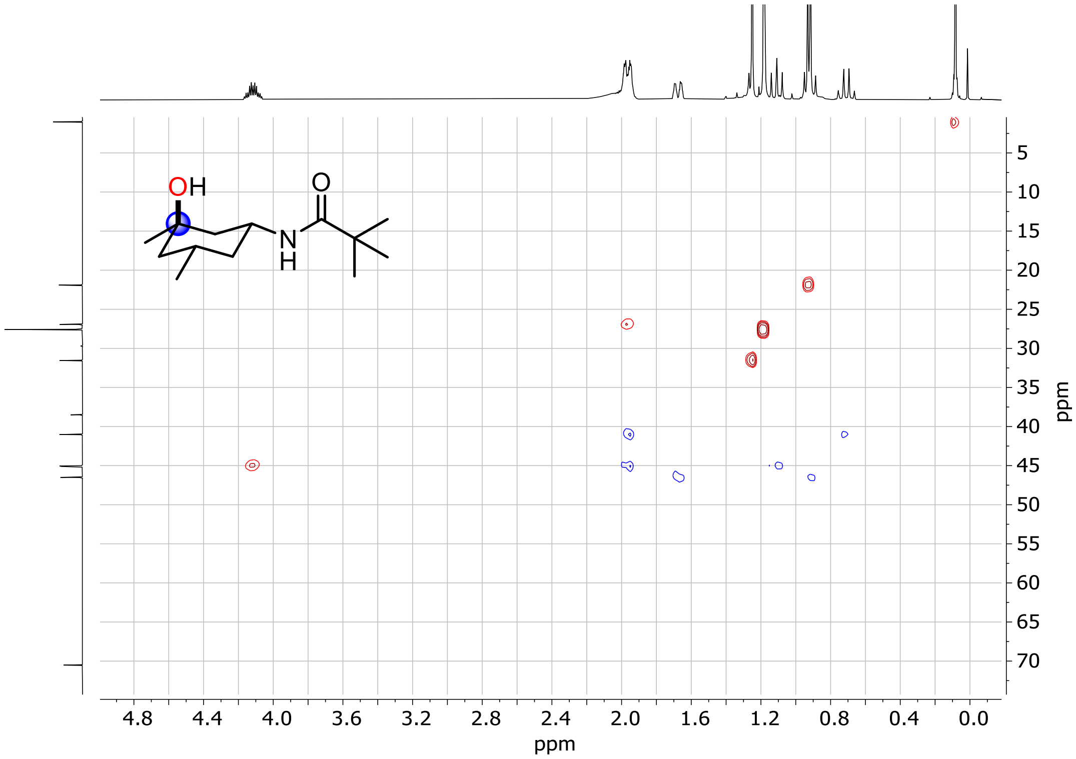

^{1}H-NMR of **14b** in $CDCl_3$

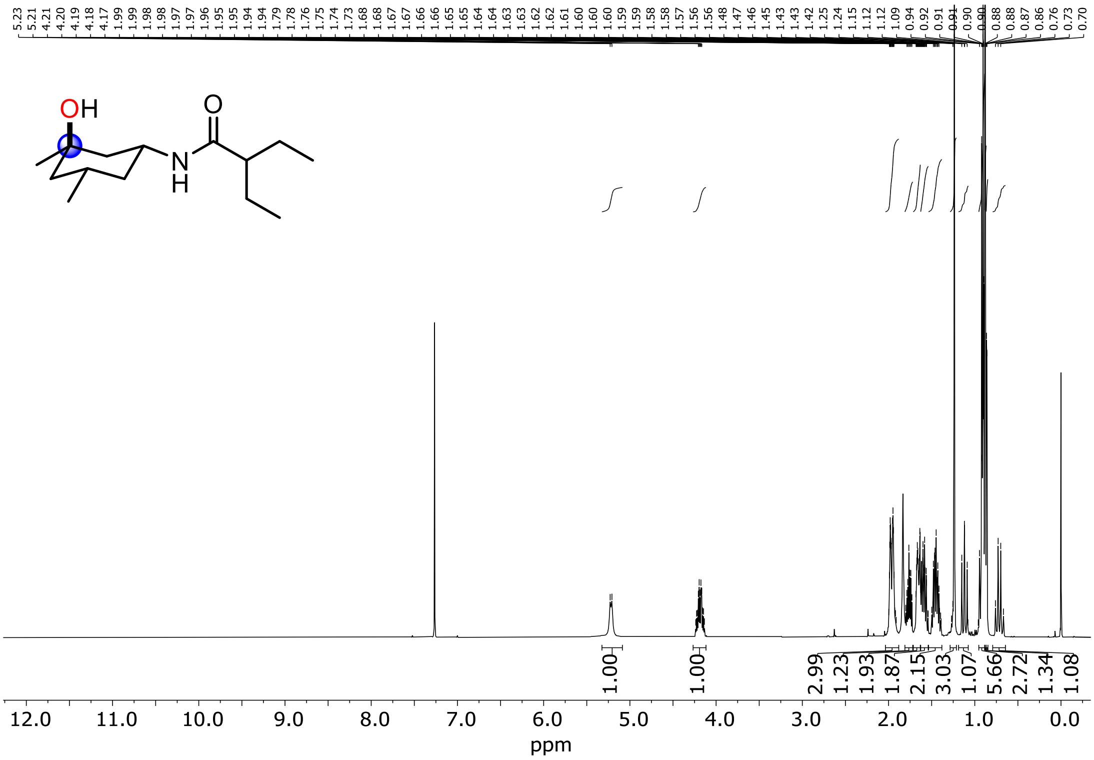

^{13}C-NMR of **14b** in $CDCl_3$

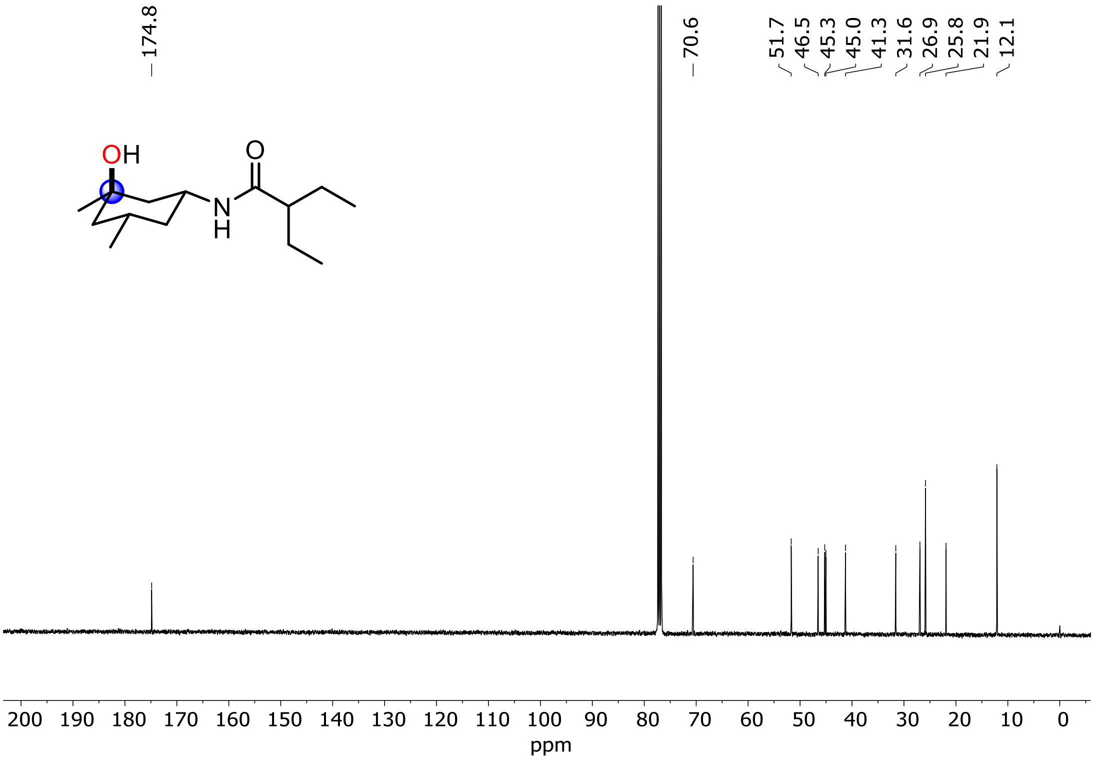

COSY-NMR of **14b** in $CDCl_3$

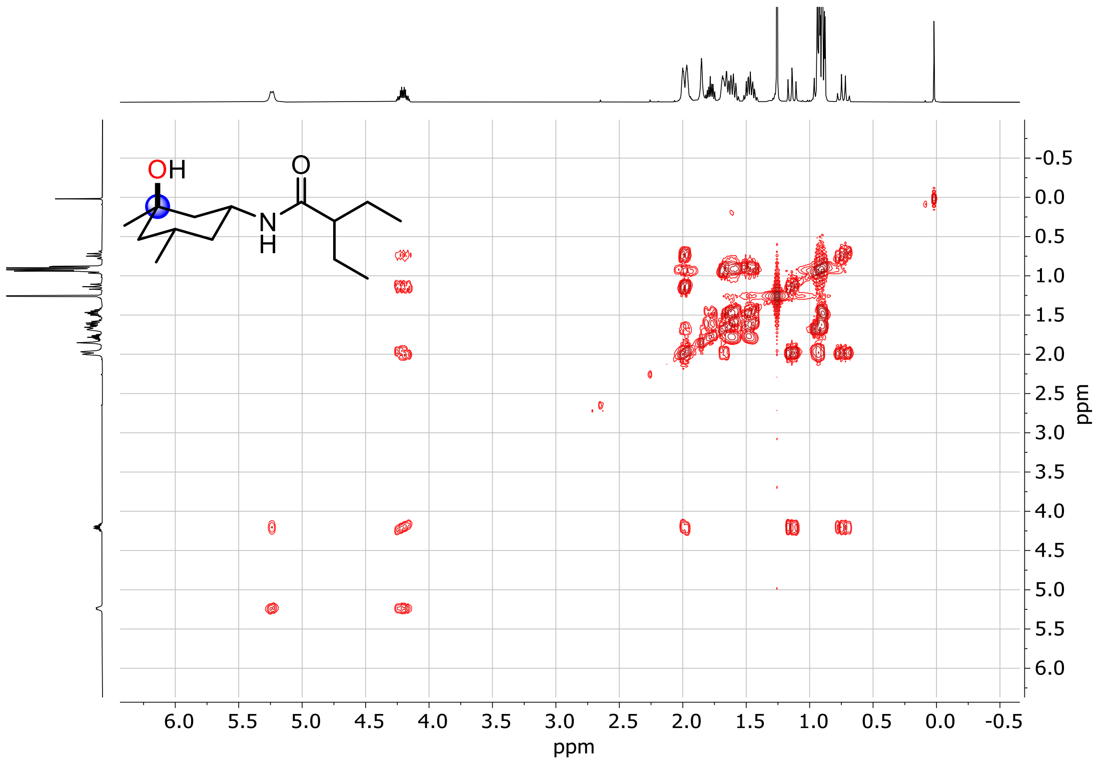

HSQC-NMR of **14b** in $CDCl_3$

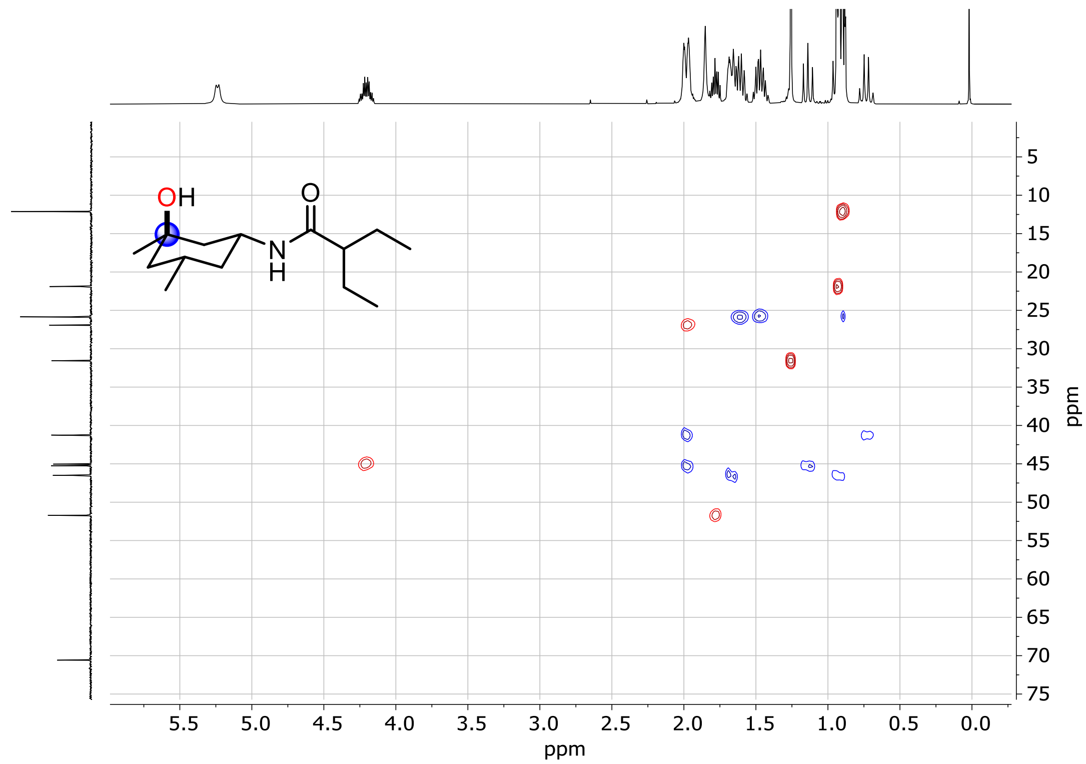

^{1}H-NMR of **15b** in $CDCl_3$

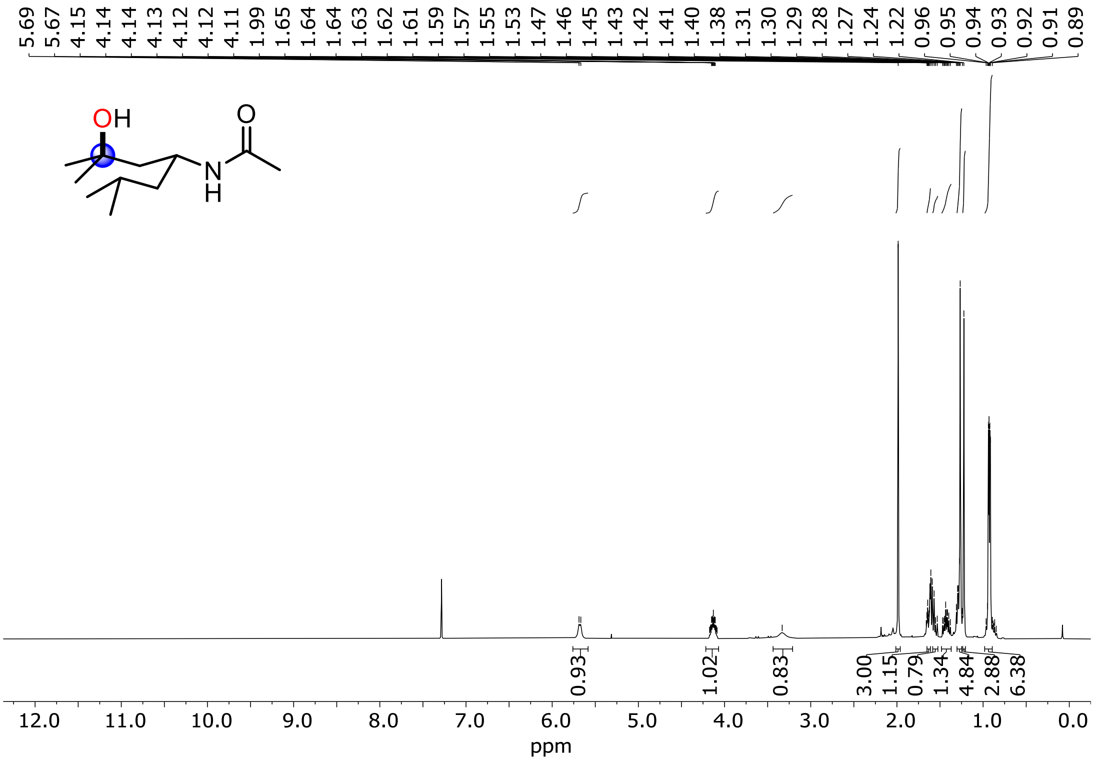

^{13}C-NMR of **15b** in $CDCl_3$

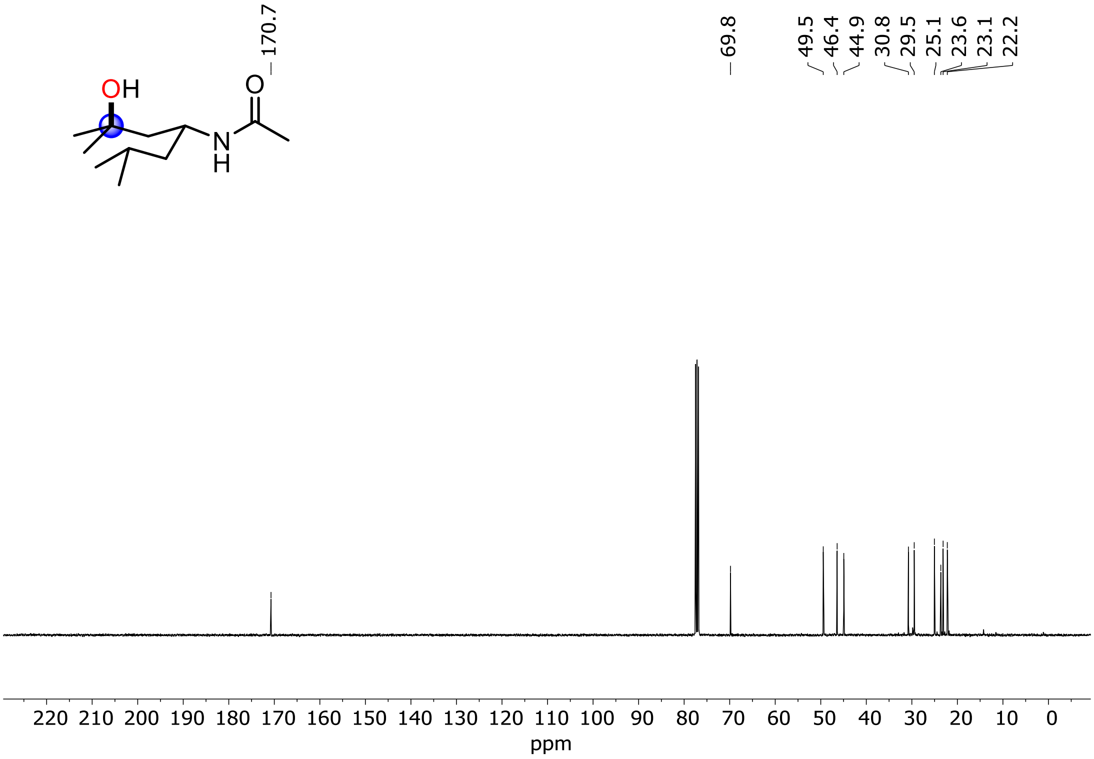

^{1}H-NMR of **16b** in $CDCl_3$

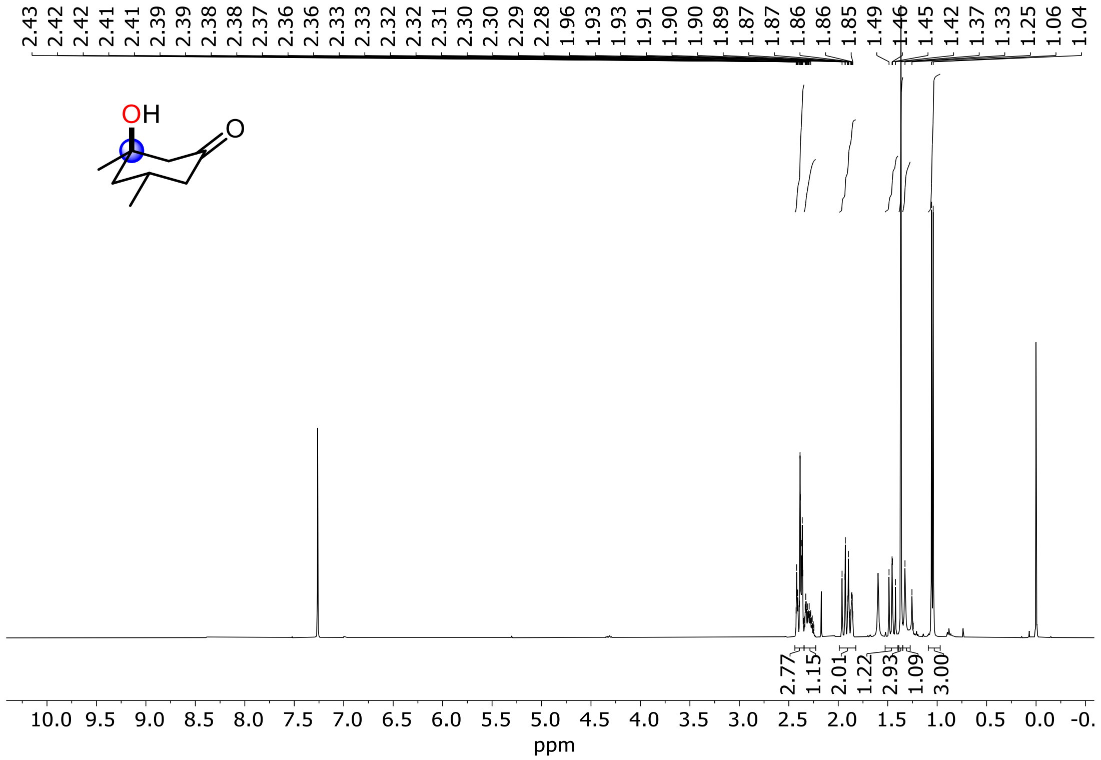

^{13}C-NMR of **16b** in $CDCl_3$

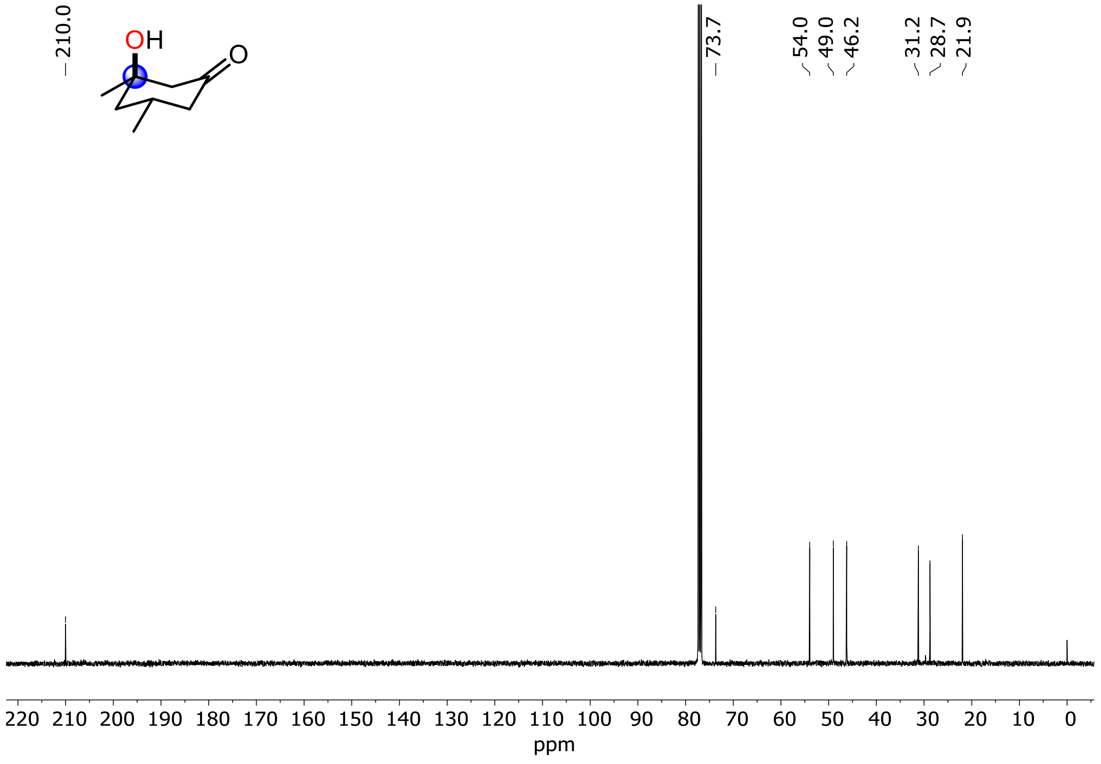

COSY-NMR of **16b** in $CDCl_3$

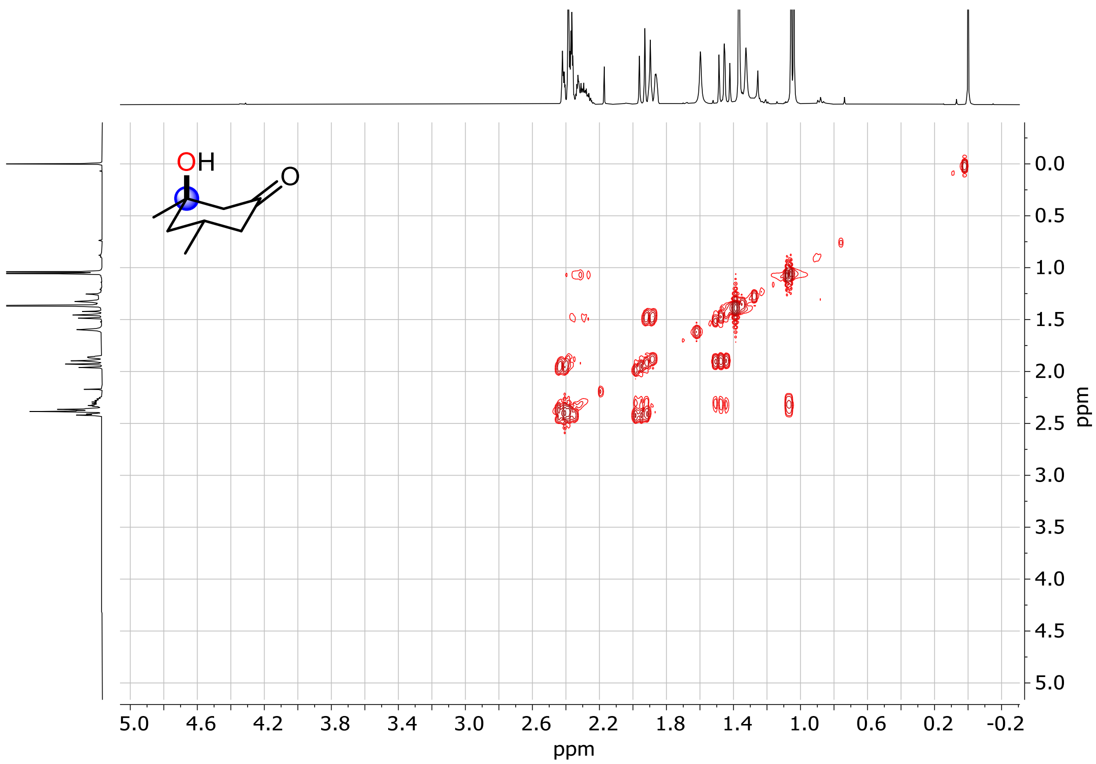

HSQC-NMR of **16b** in $CDCl_3$

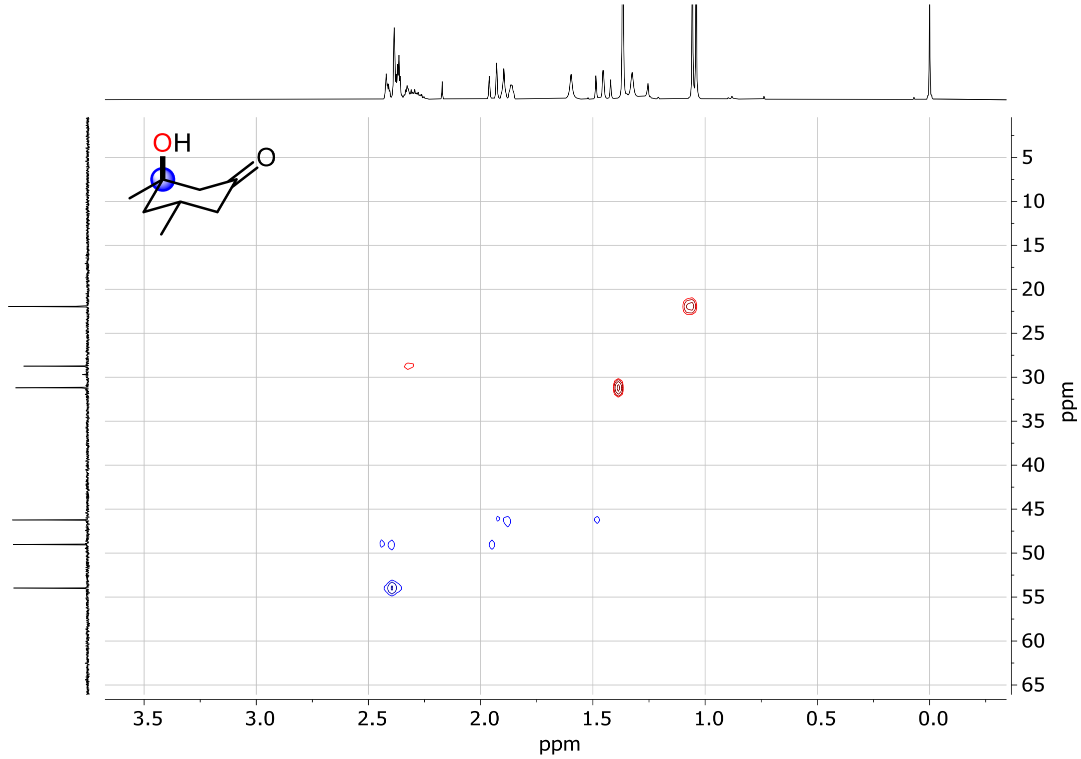

^{1}H-NMR of **17b** in $CDCl_3$

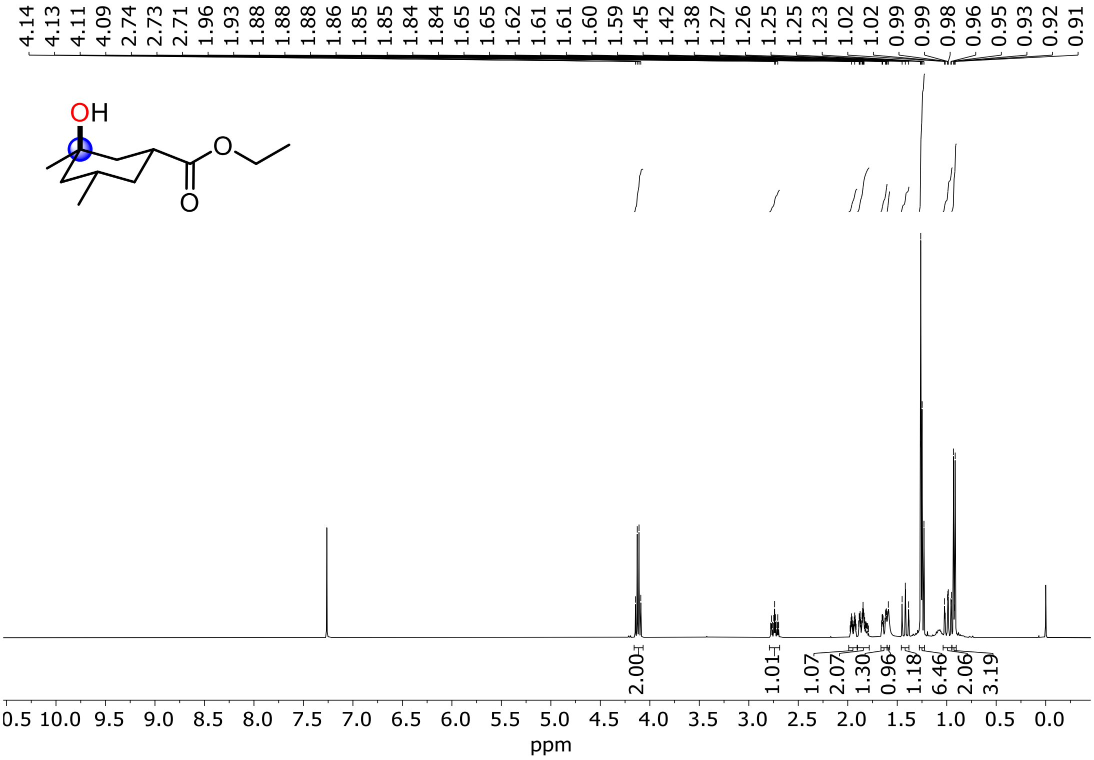

^{13}C-NMR of **17b** in $CDCl_3$

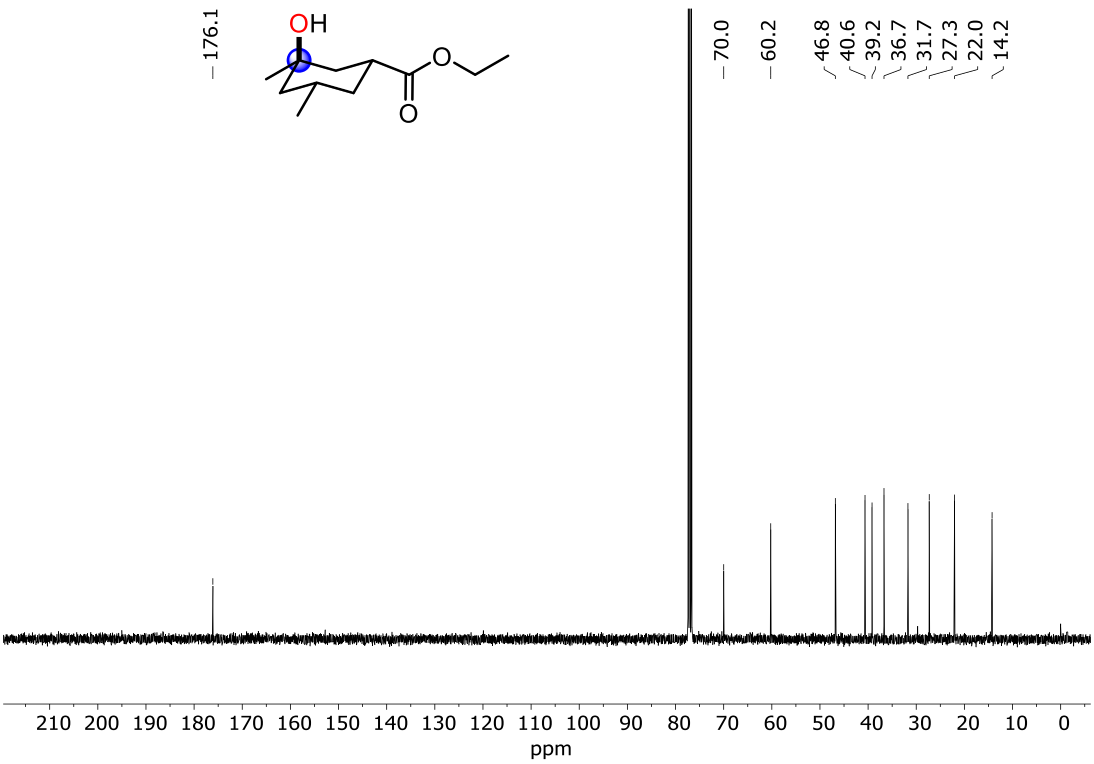

COSY-NMR of **17b** in $CDCl_3$

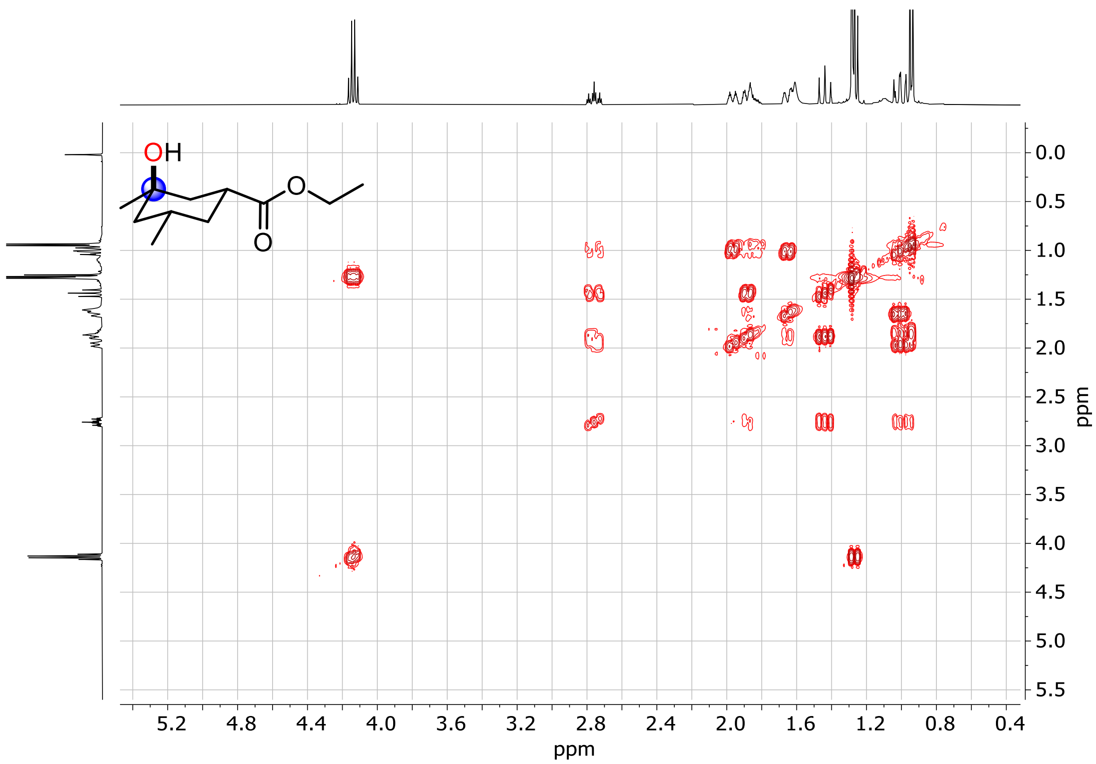

HSQC-NMR of **17b** in $CDCl_3$

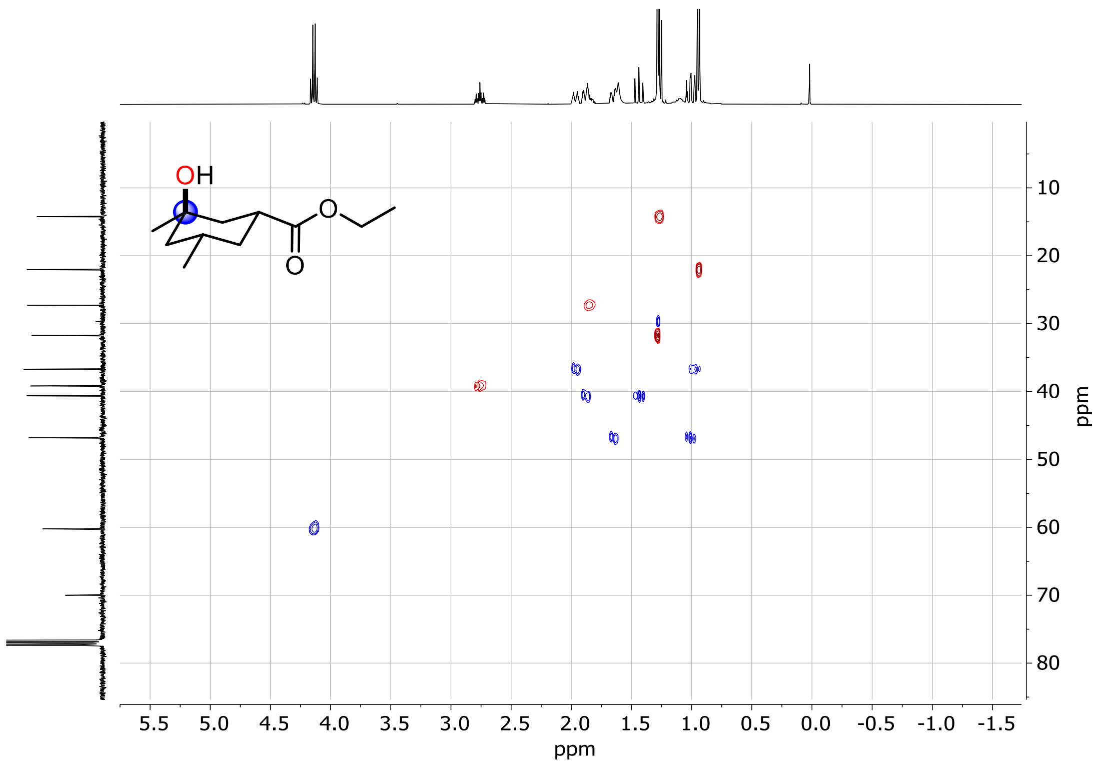

^{1}H-NMR of **18b** in $CDCl_3$

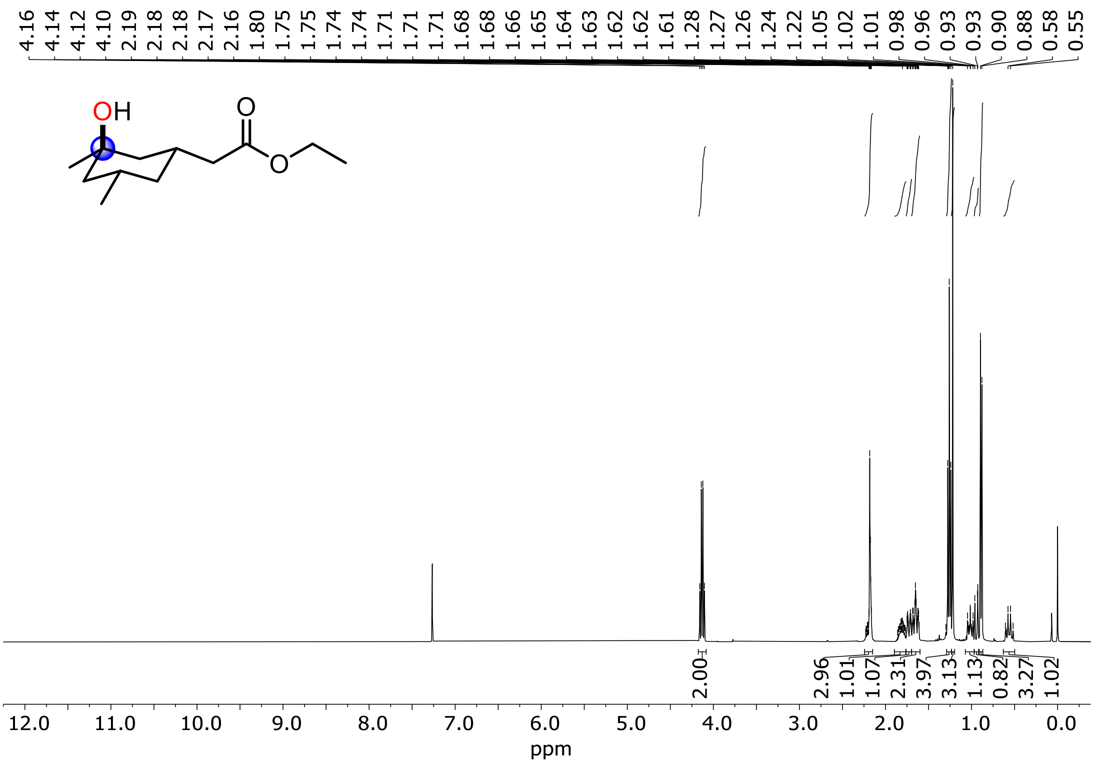

^{13}C-NMR of **18b** in $CDCl_3$

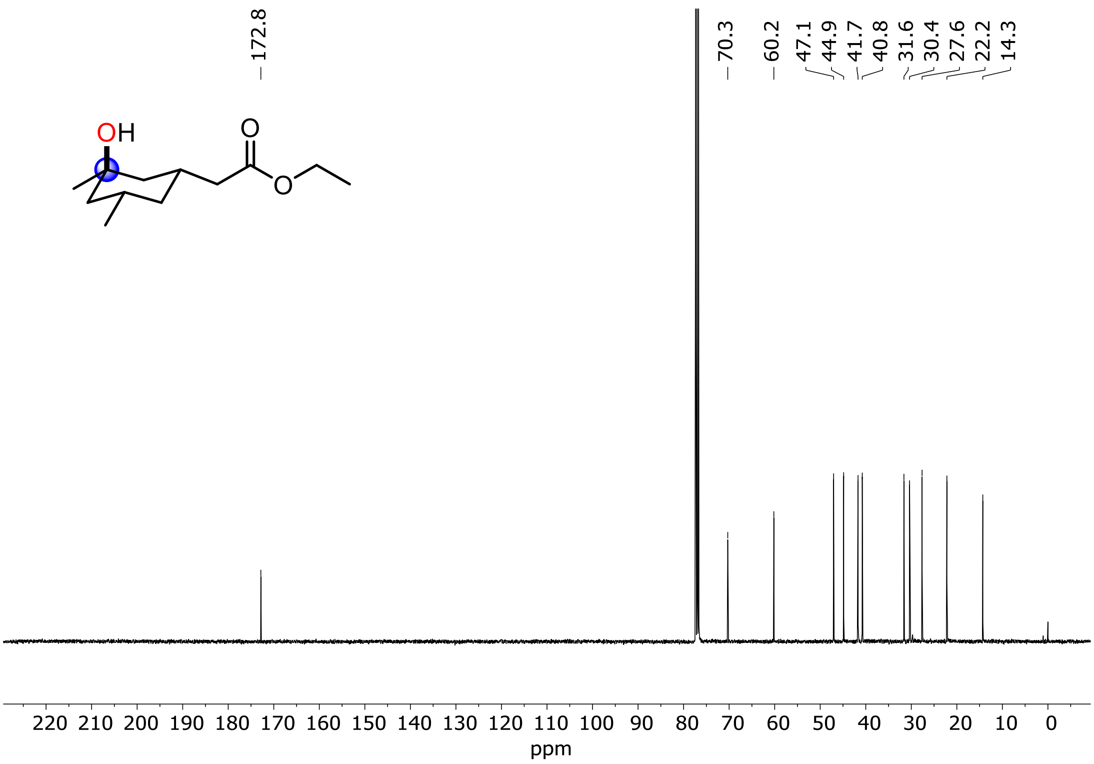

COSY-NMR of **18b** in $CDCl_3$

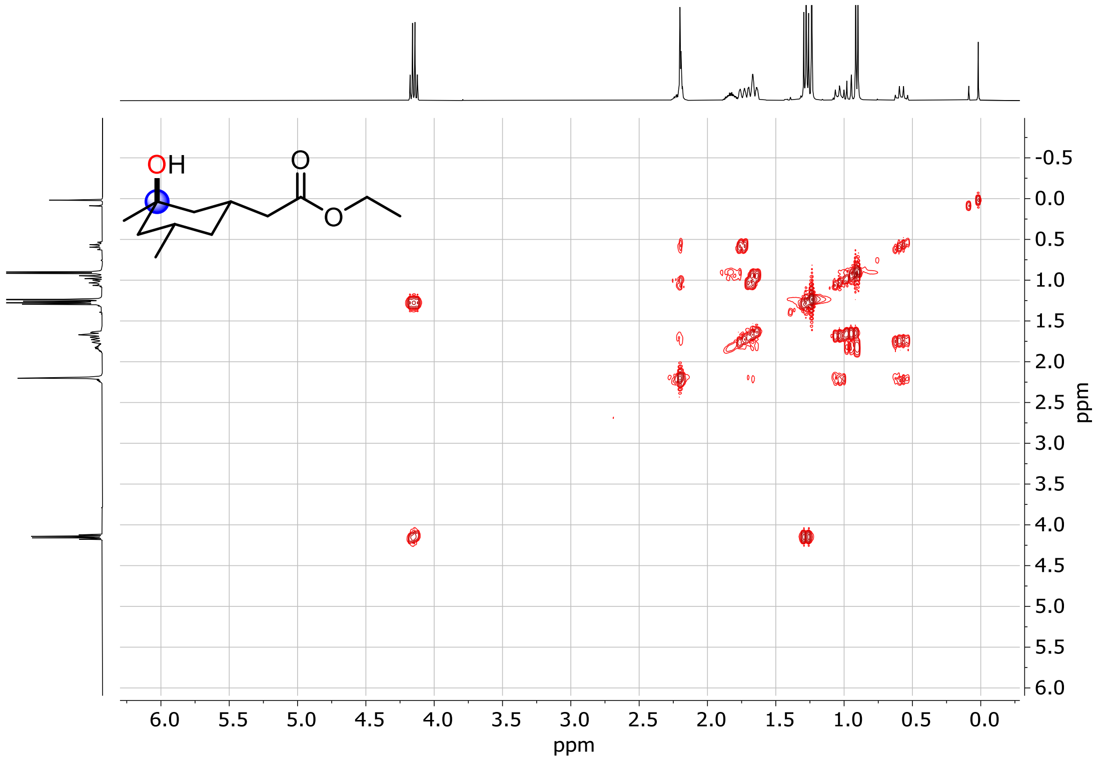

HSQC-NMR of **18b** in $CDCl_3$

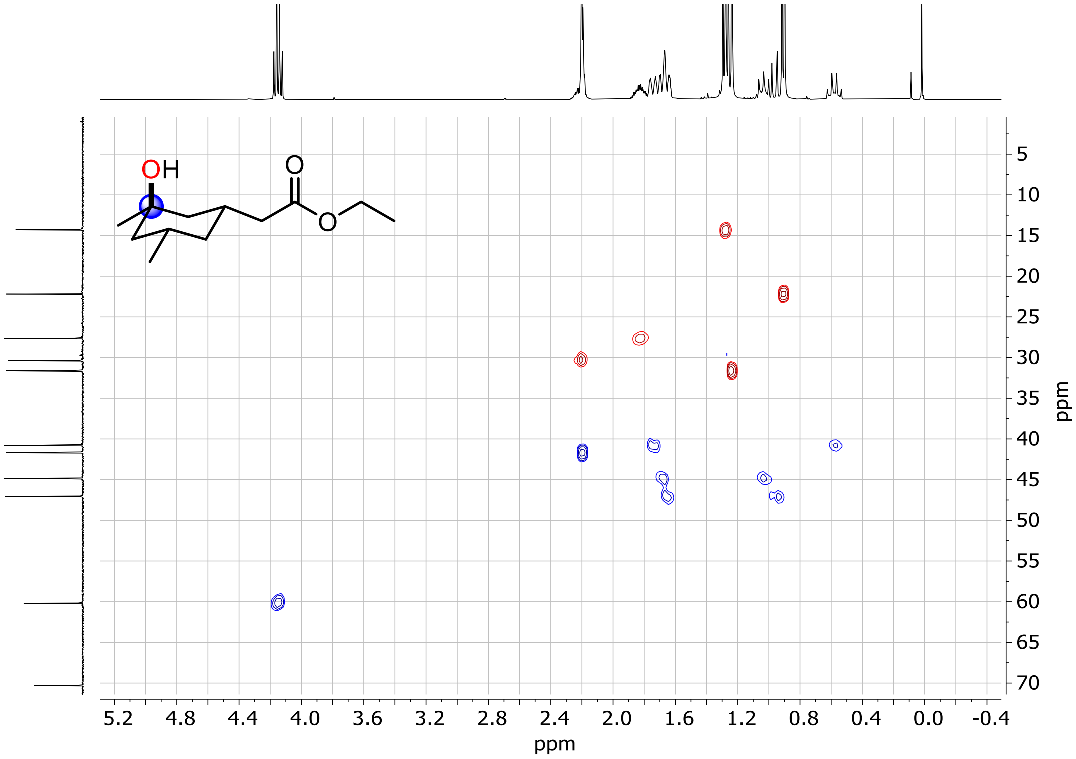

^{1}H-NMR of **19b** in $CDCl_3$

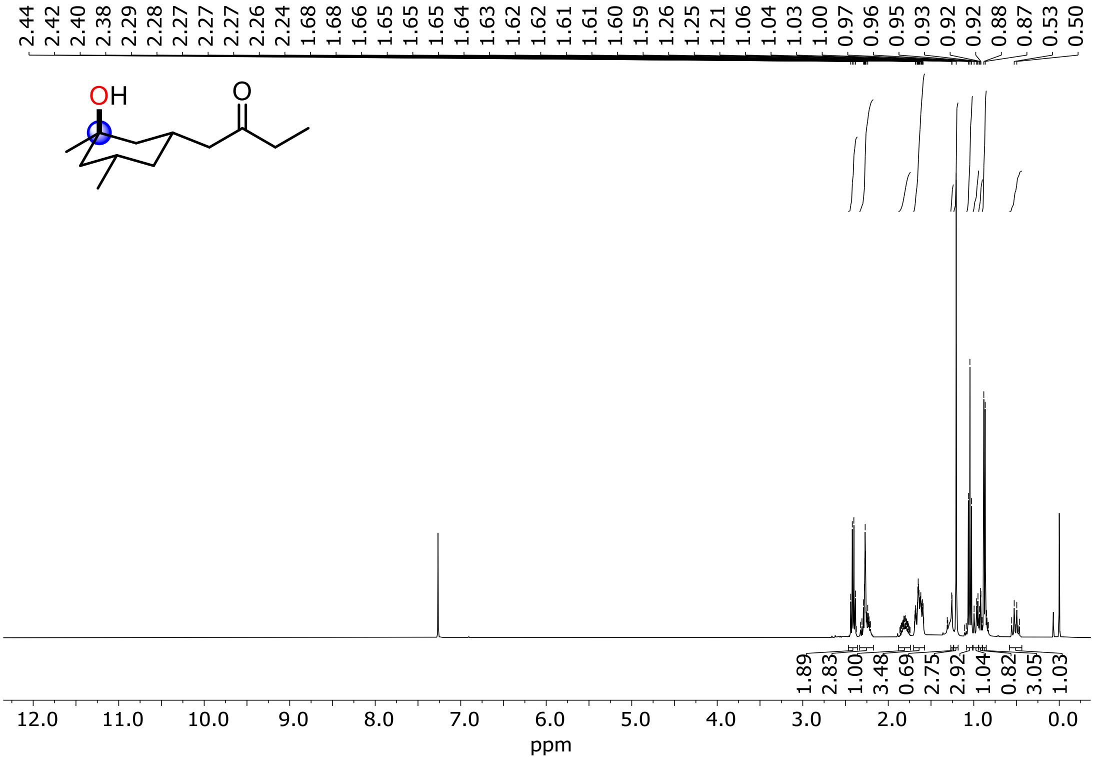

^{13}C-NMR of **19b** in $CDCl_3$

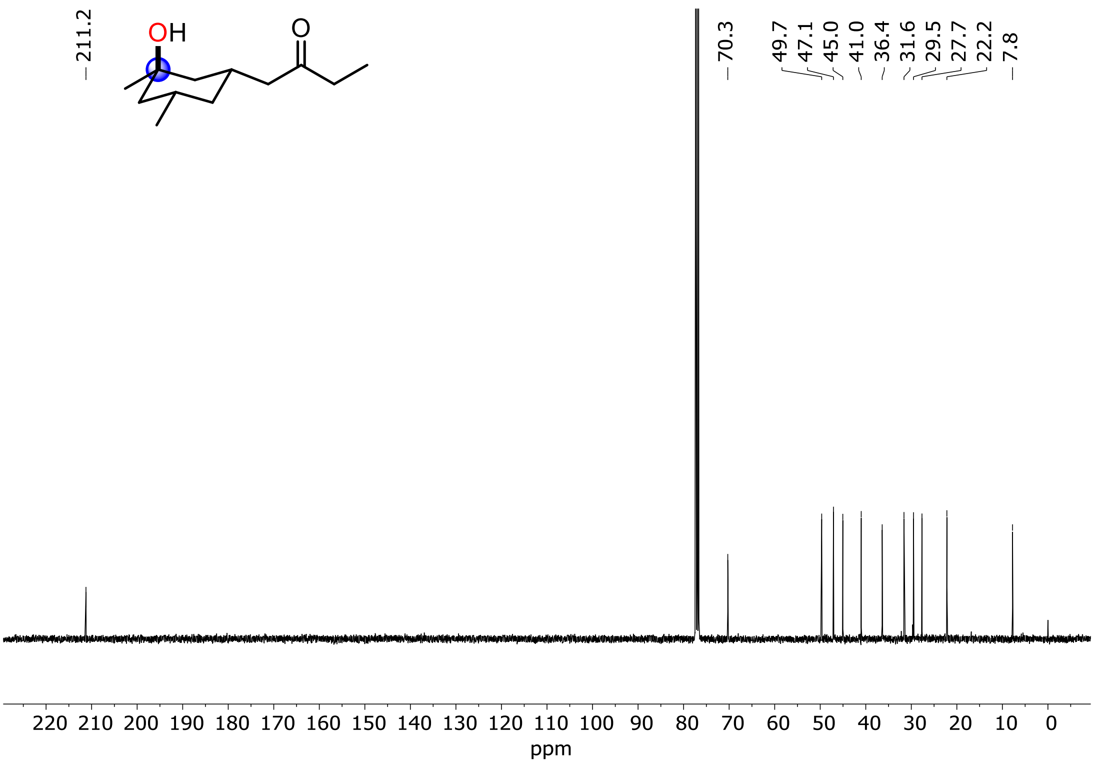

COSY-NMR of **19b** in $CDCl_3$

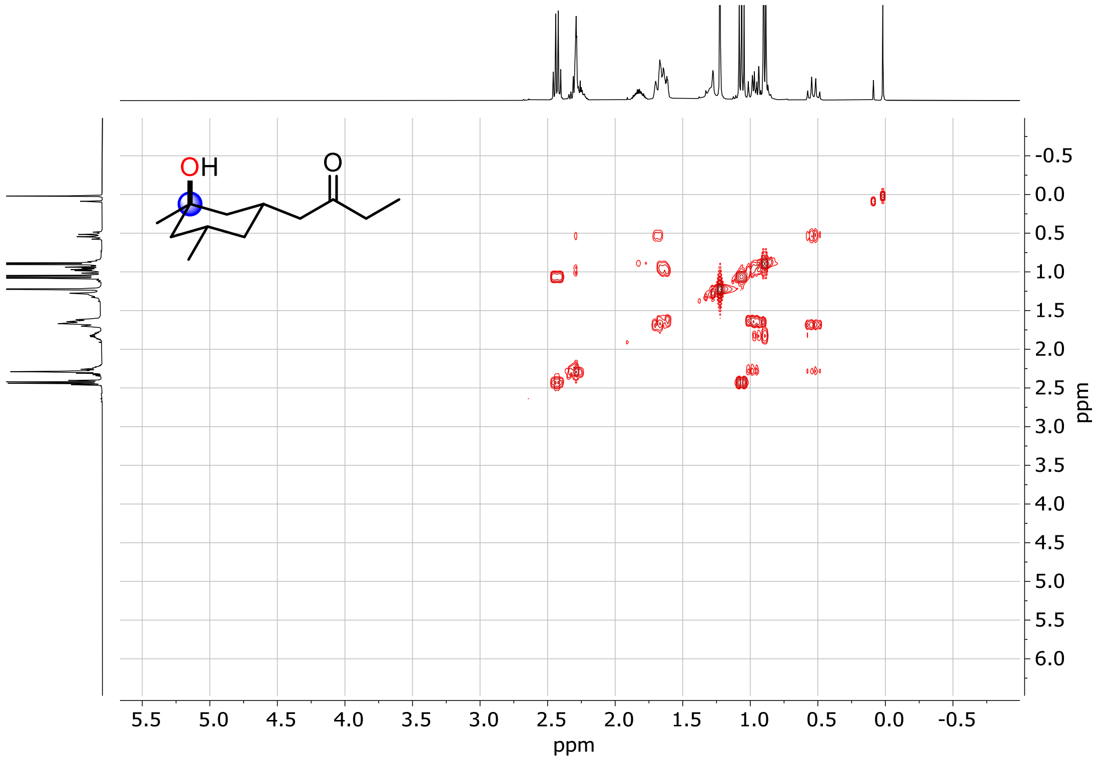

HSQC-NMR of **19b** in $CDCl_3$

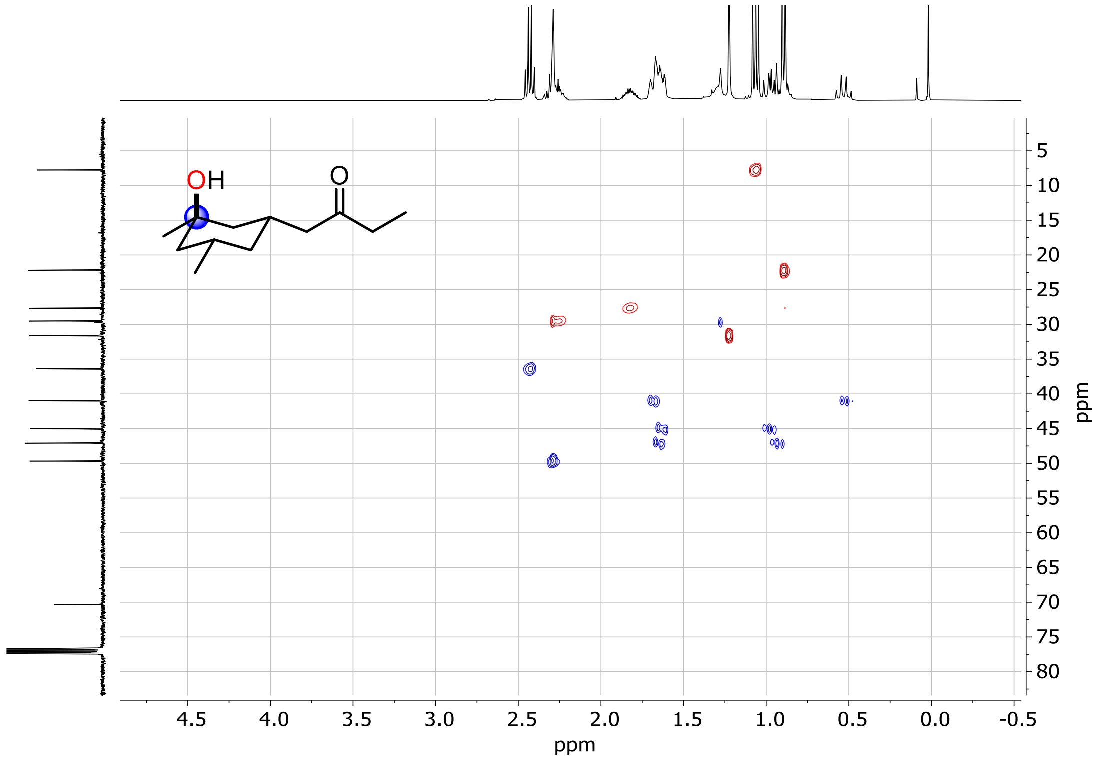

^{1}H-NMR of **20b** in $CDCl_3$

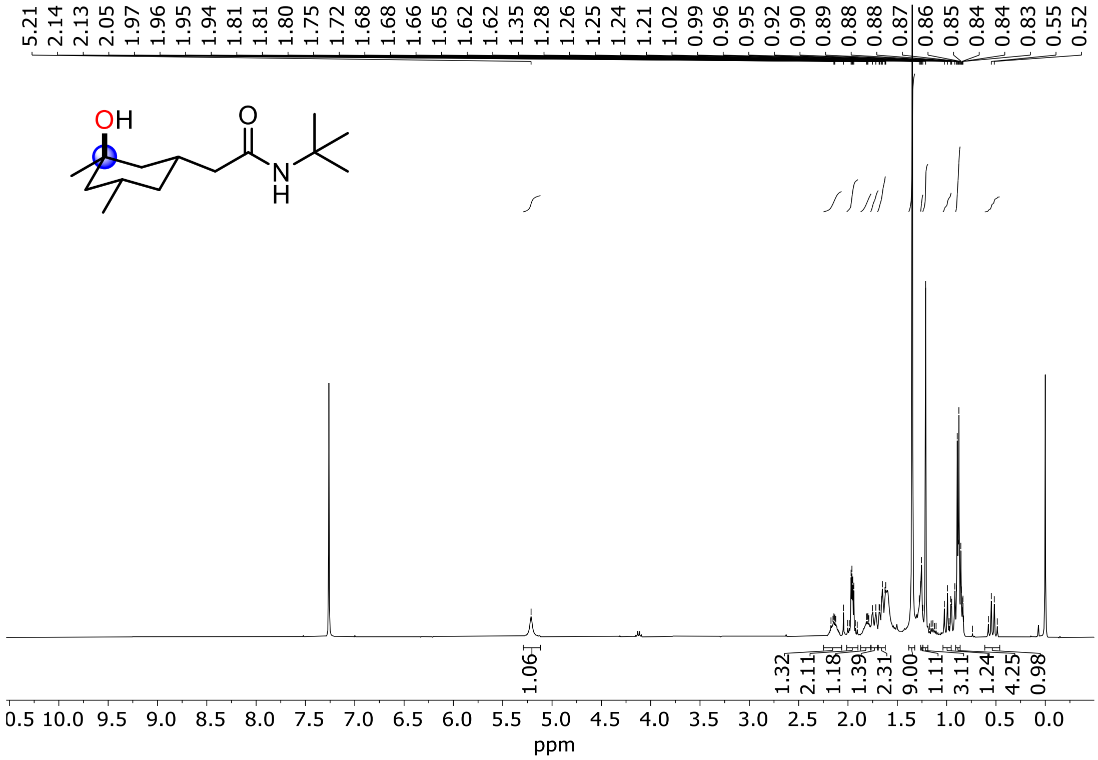

^{13}C-NMR of **20b** in $CDCl_3$

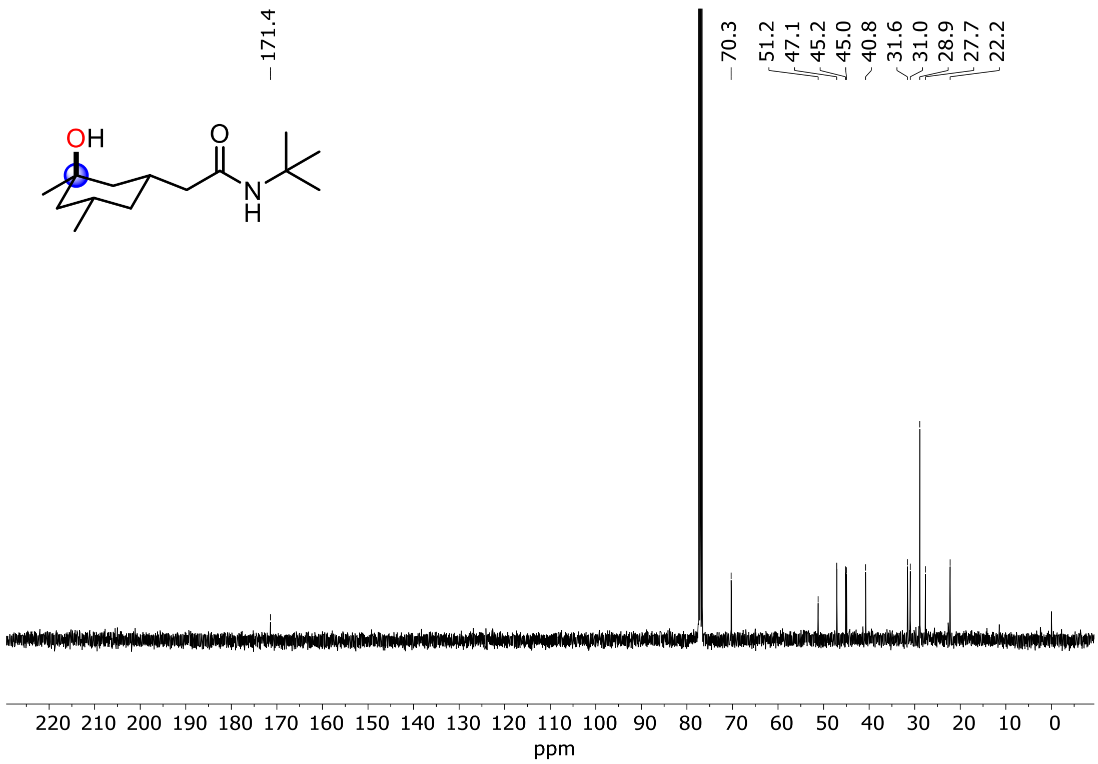

COSY-NMR of **20b** in $CDCl_3$

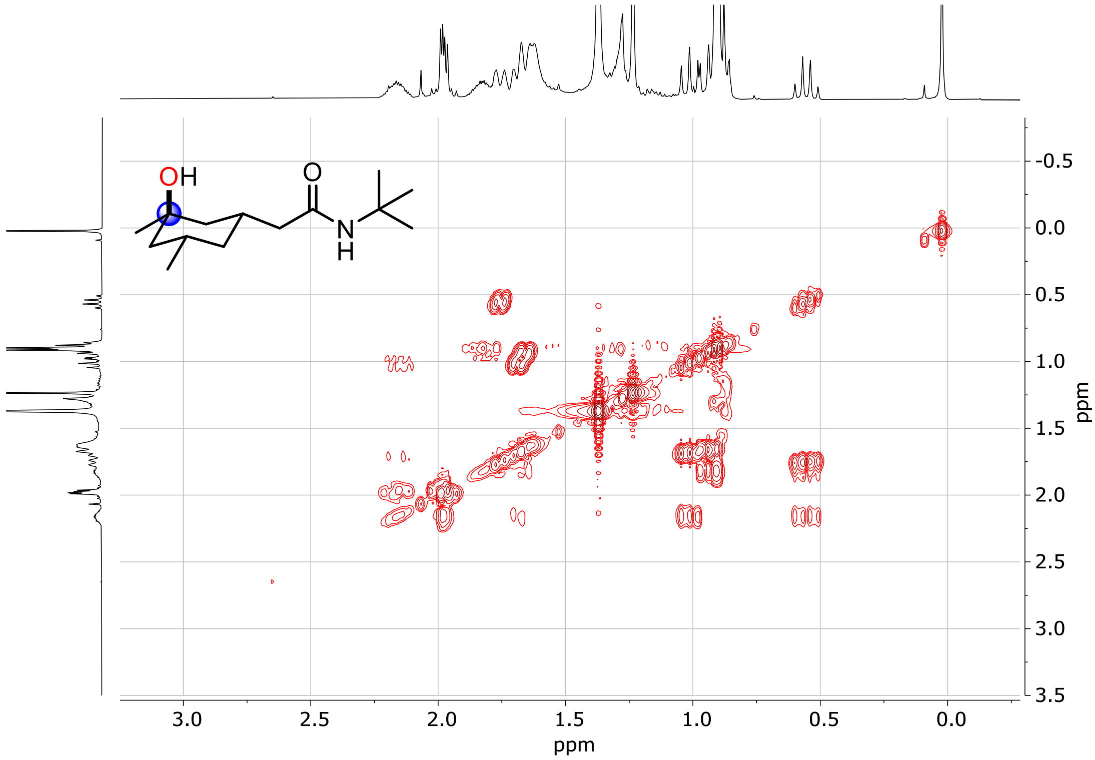

HSQC-NMR of **20b** in $CDCl_3$

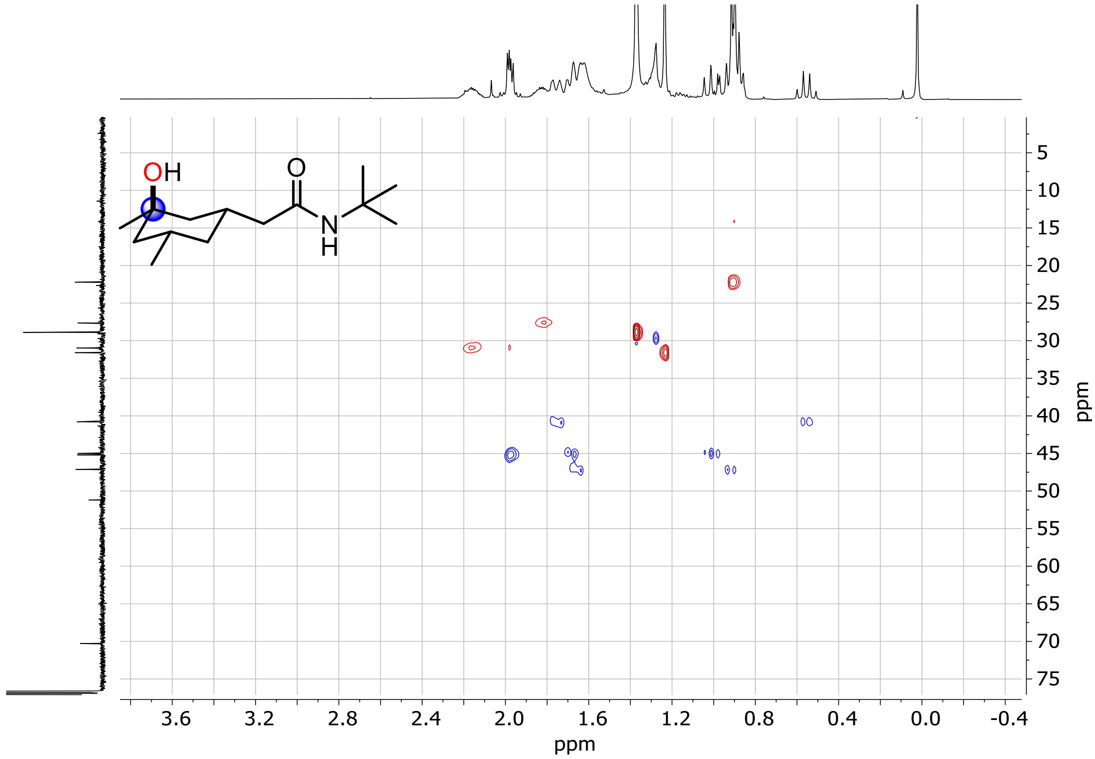

^{1}H-NMR of **21b** in $CDCl_3$

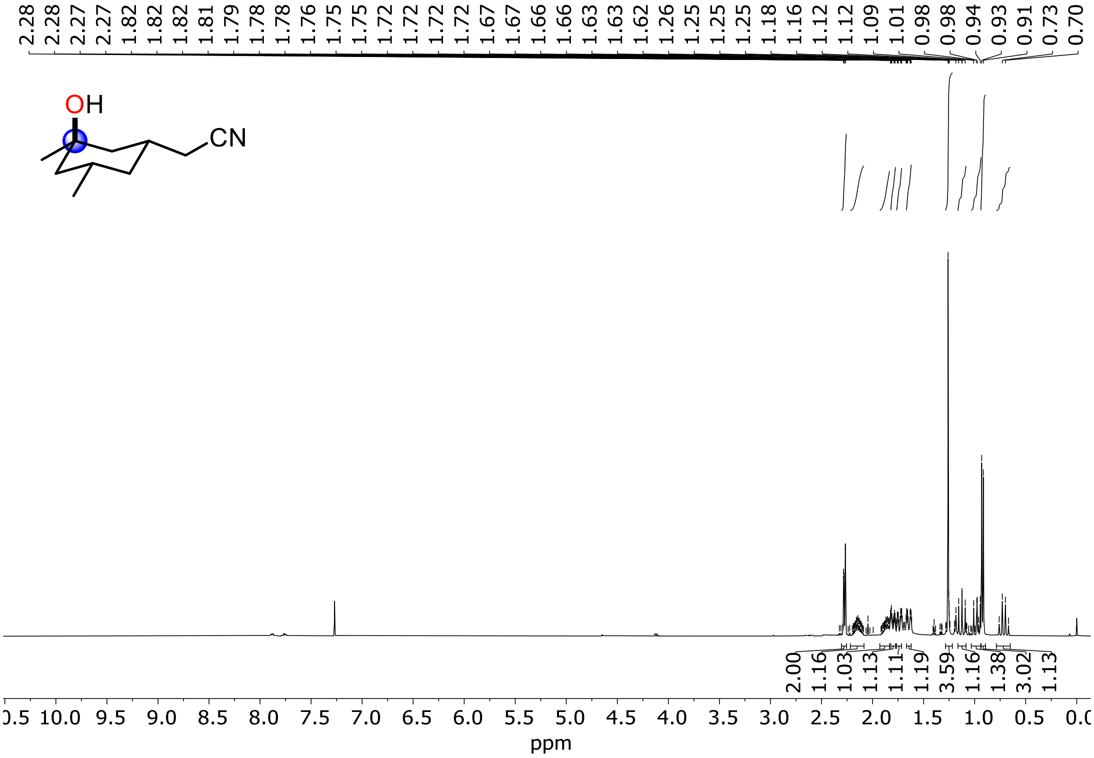

^{13}C-NMR of **21b** in $CDCl_3$

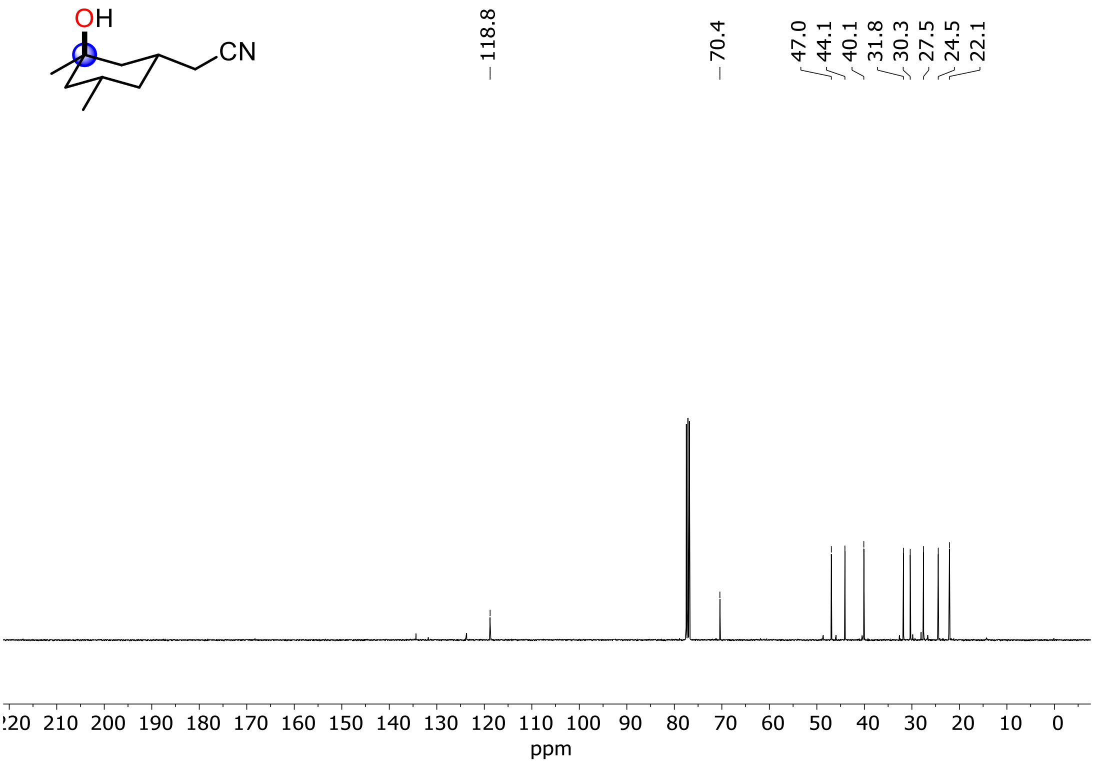

COSY-NMR of **21b** in $CDCl_3$

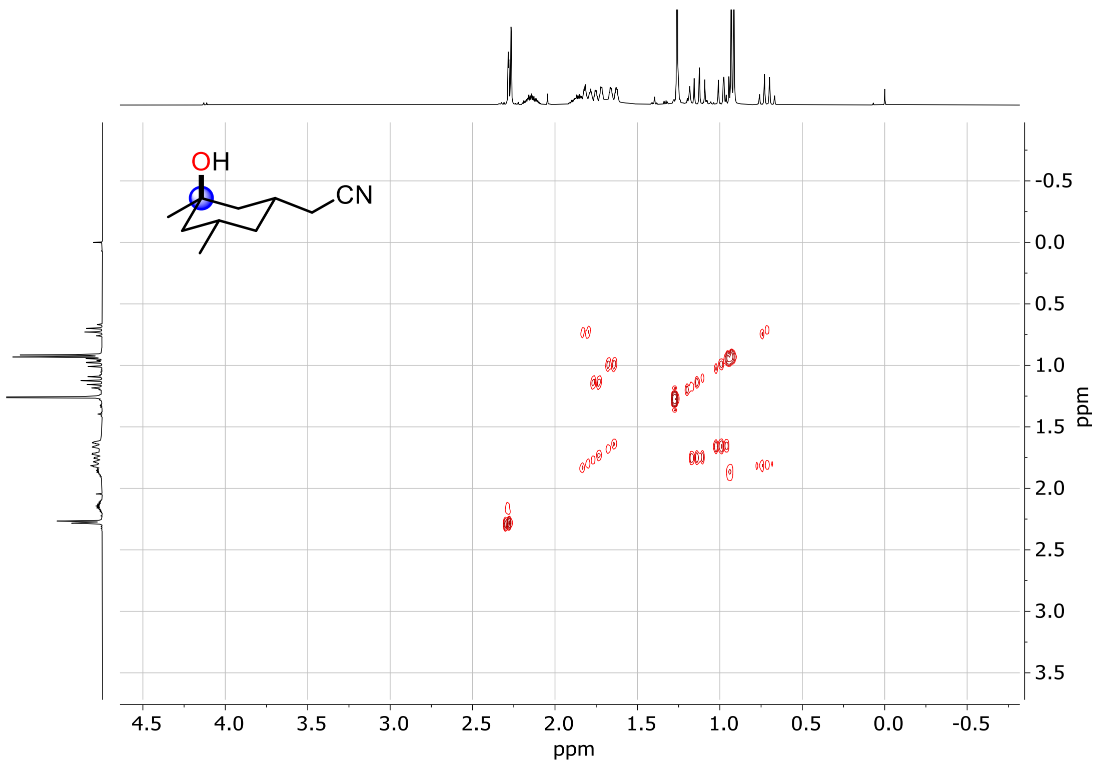

HSQC-NMR of **21b** in $CDCl_3$

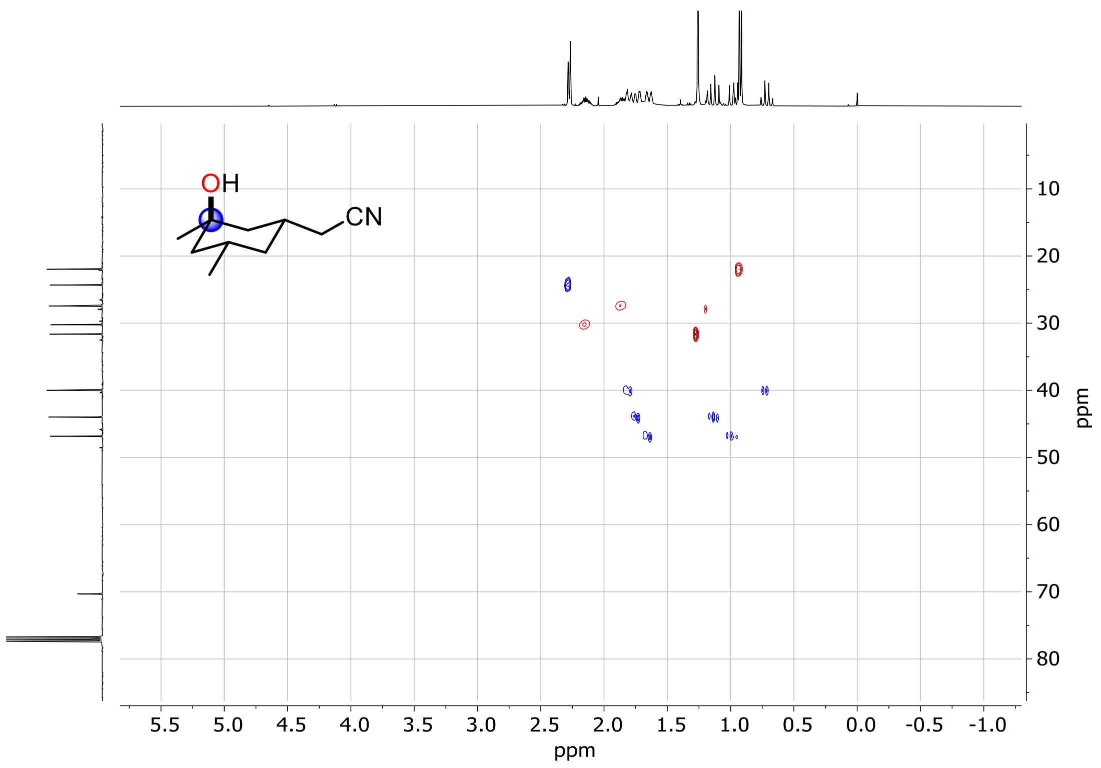

^{1}H-NMR of **22b** in $CDCl_3$

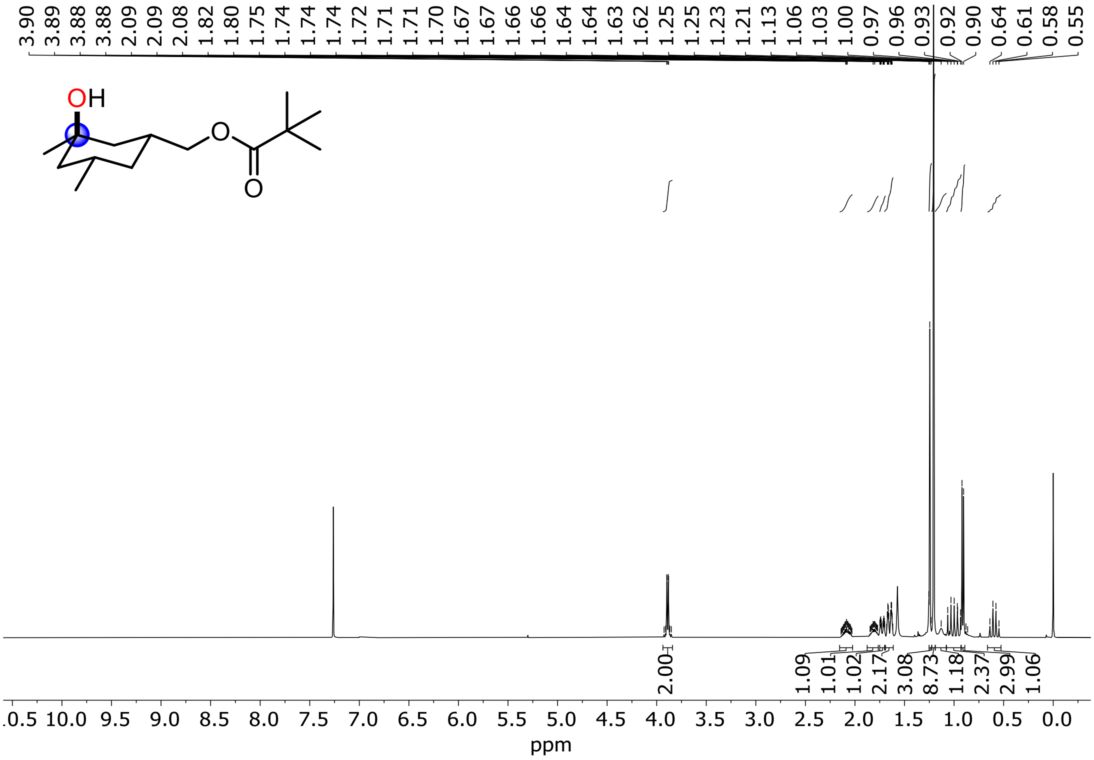

^{13}C-NMR of **22b** in $CDCl_3$

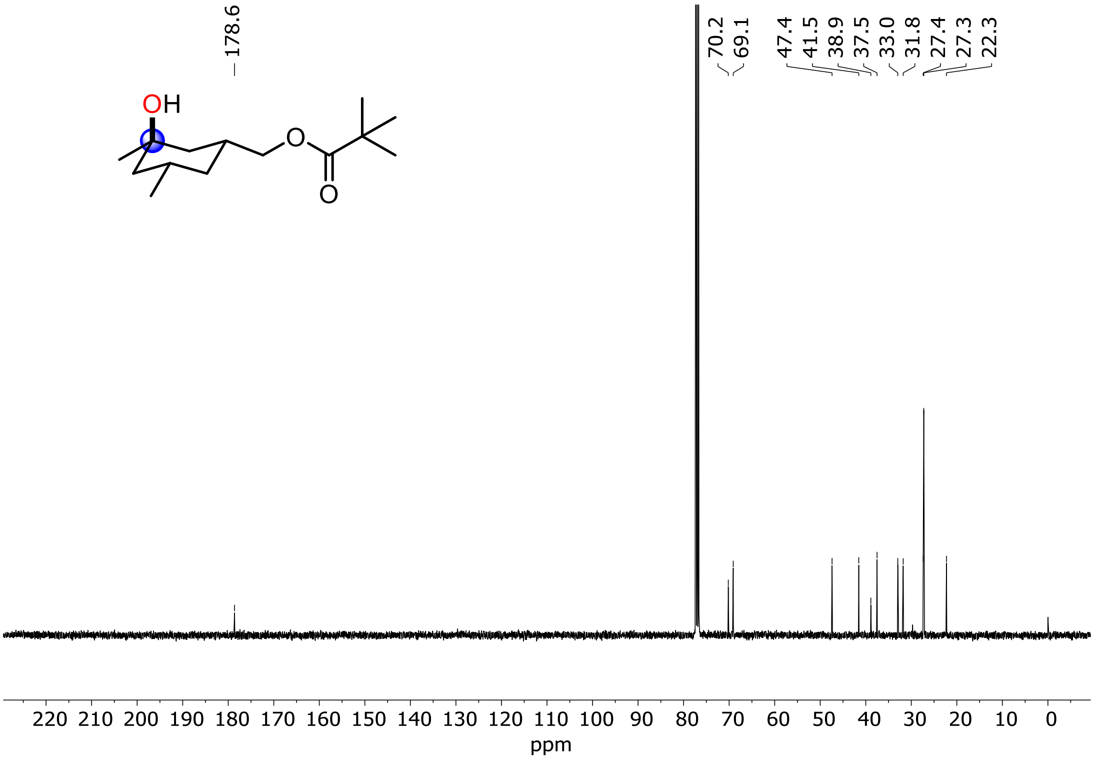

COSY-NMR of **22b** in $CDCl_3$

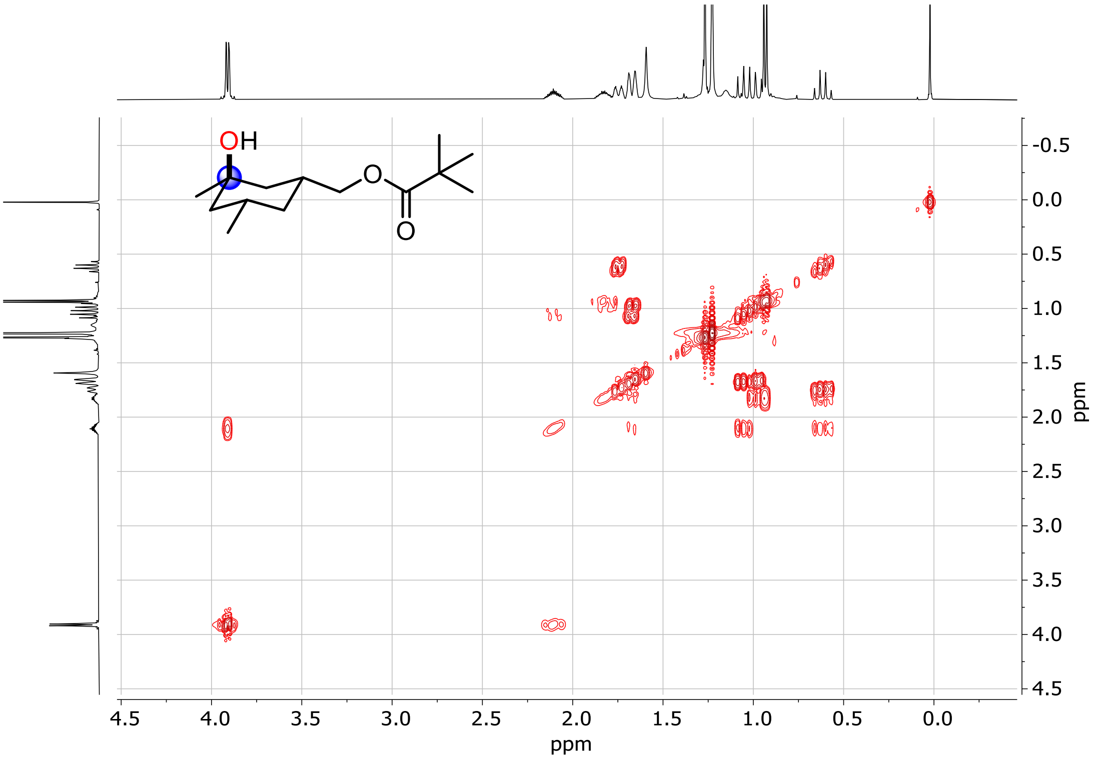

HSQC-NMR of **22b** in $CDCl_3$

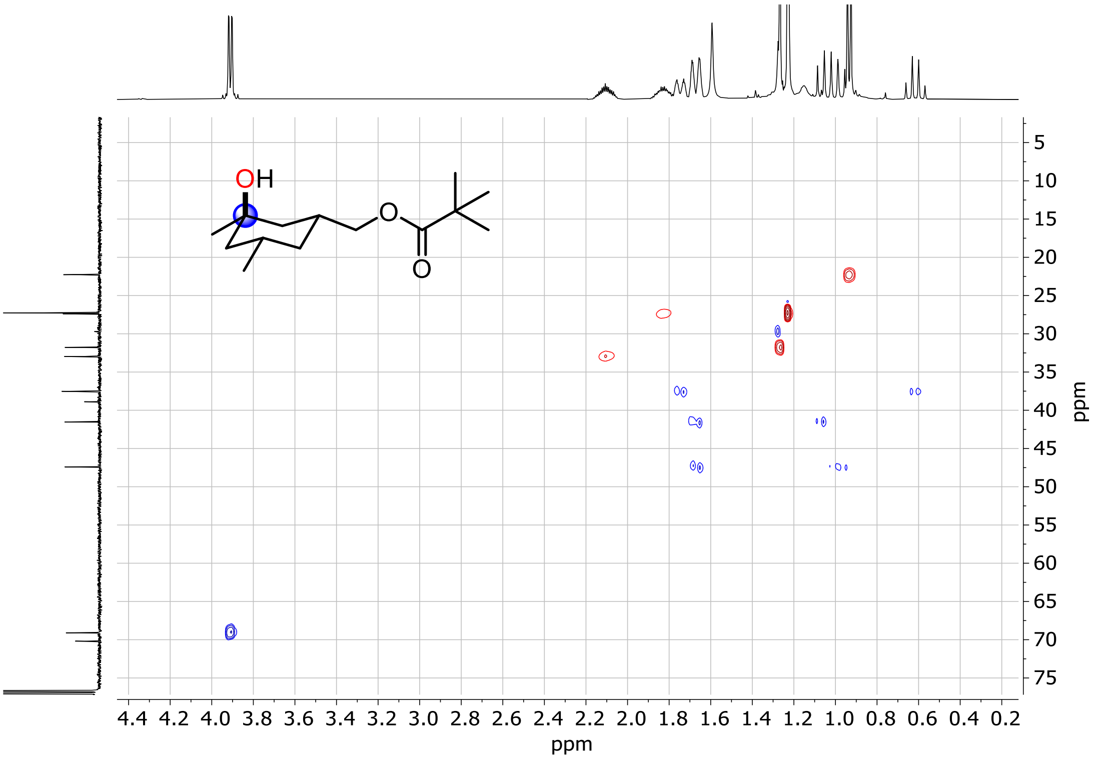

^{1}H-NMR of **23b** in $CDCl_3$

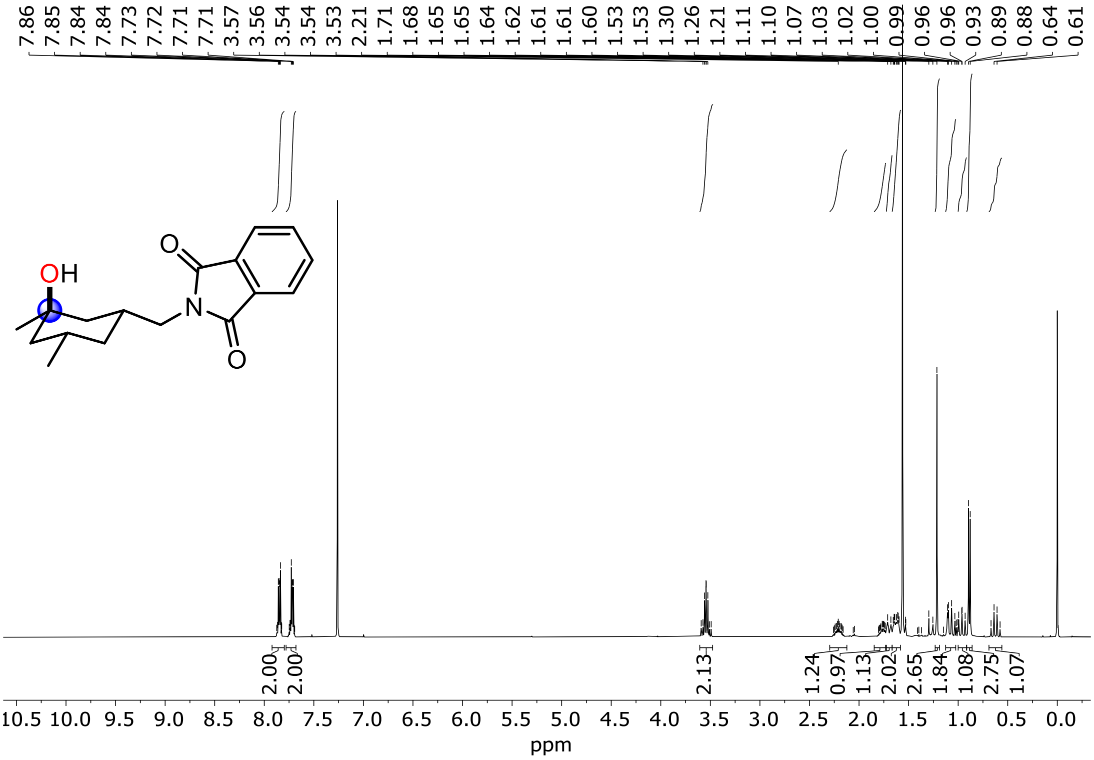

^{13}C-NMR of **23b** in $CDCl_3$

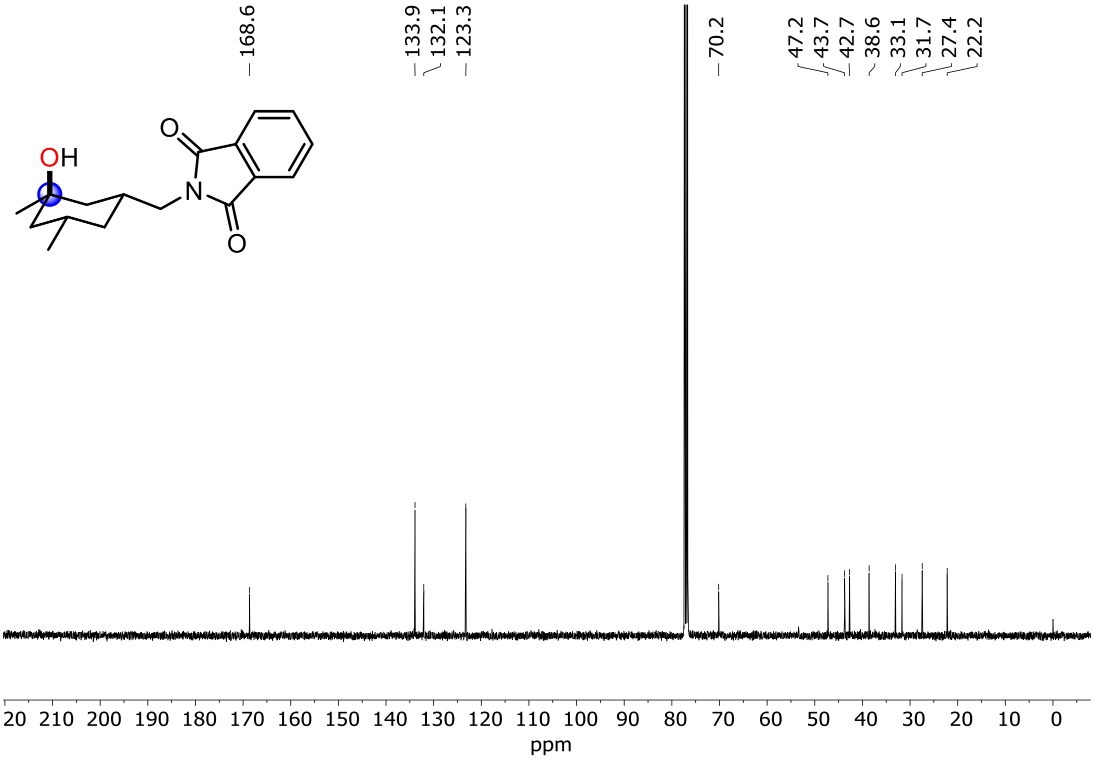

COSY-NMR of **23b** in $CDCl_3$

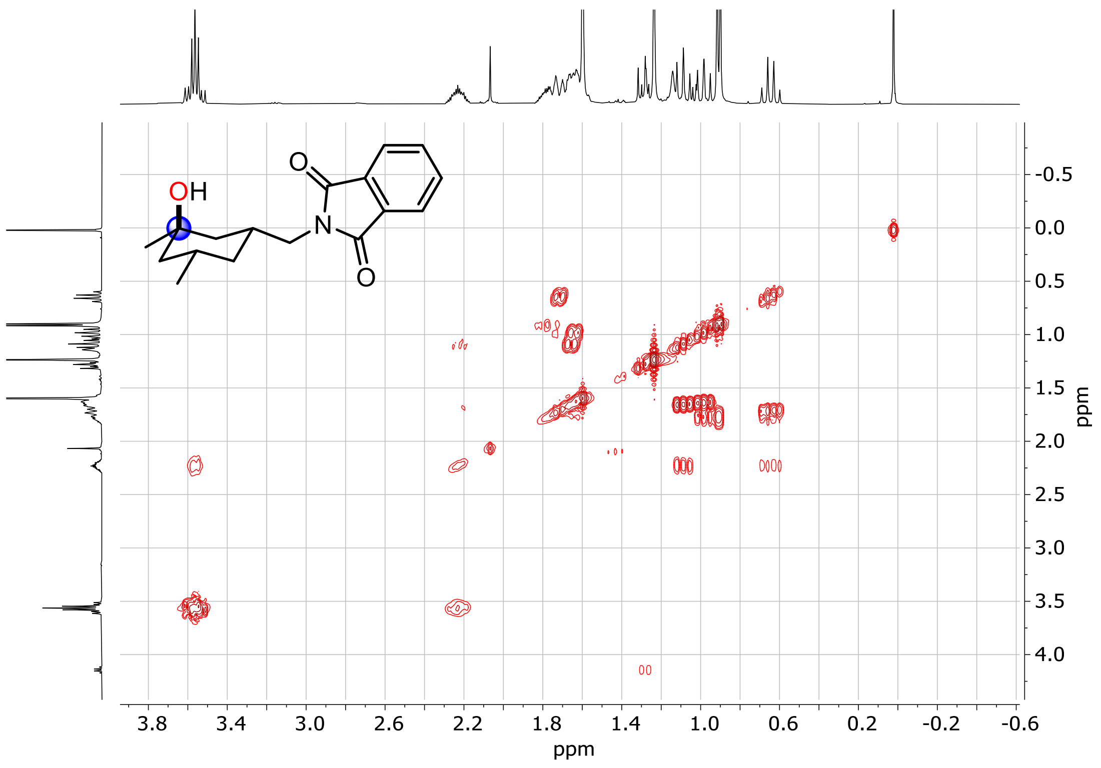

HSQC-NMR of **23b** in $CDCl_3$

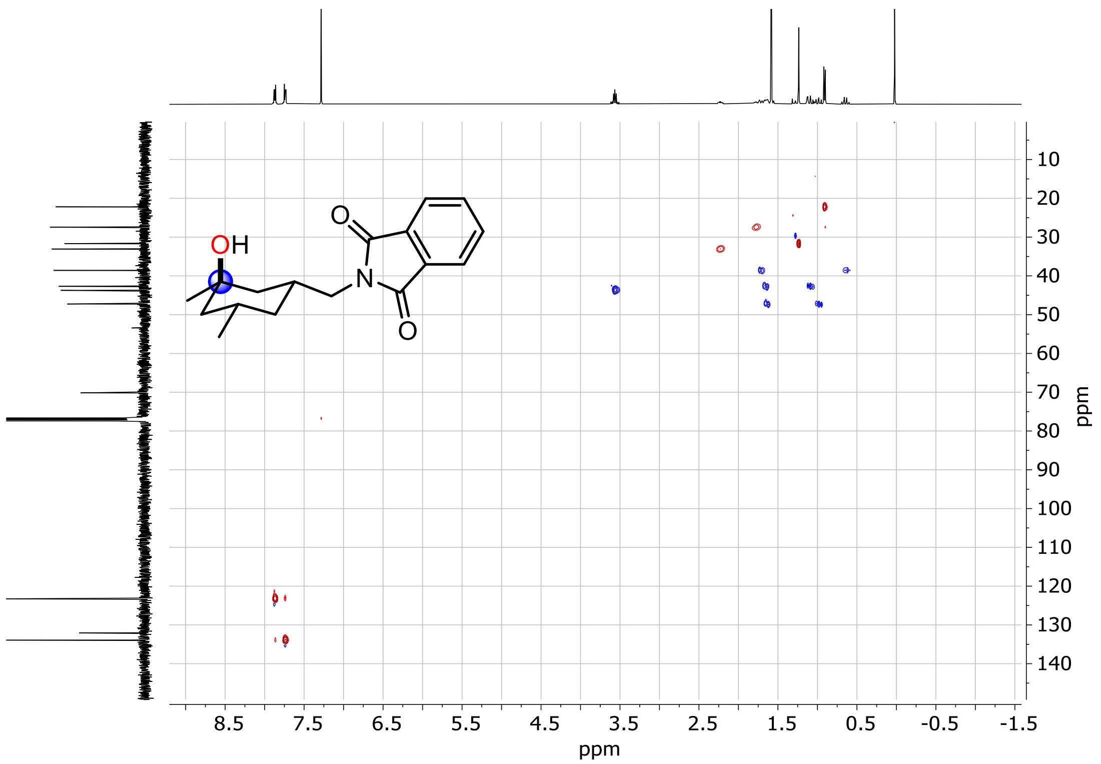

^{1}H-NMR of **24b** in $CDCl_3$

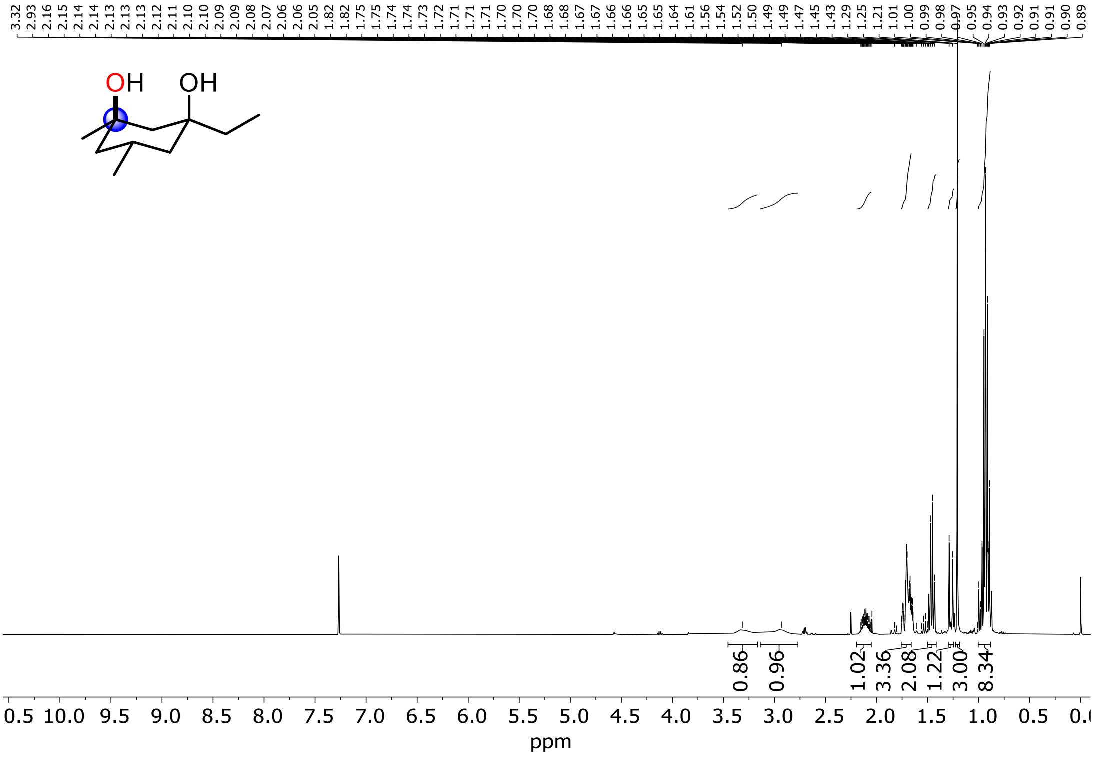

^{13}C-NMR of **24b** in $CDCl_3$

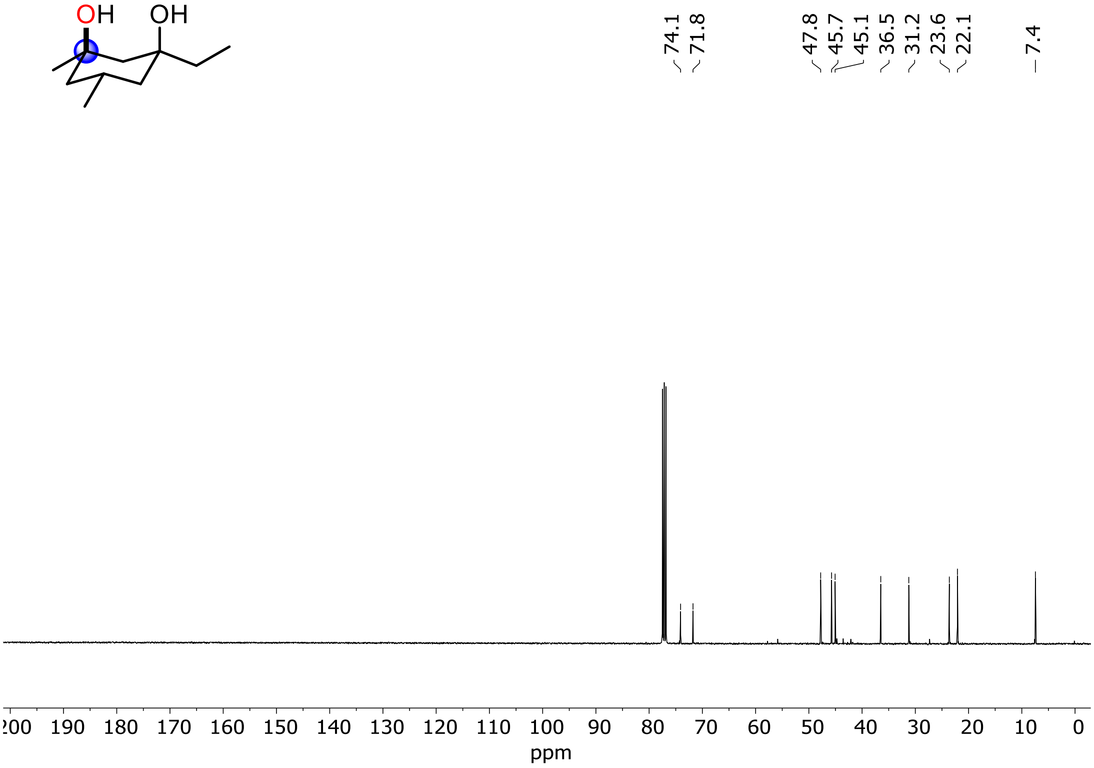

COSY-NMR of **24b** in $CDCl_3$

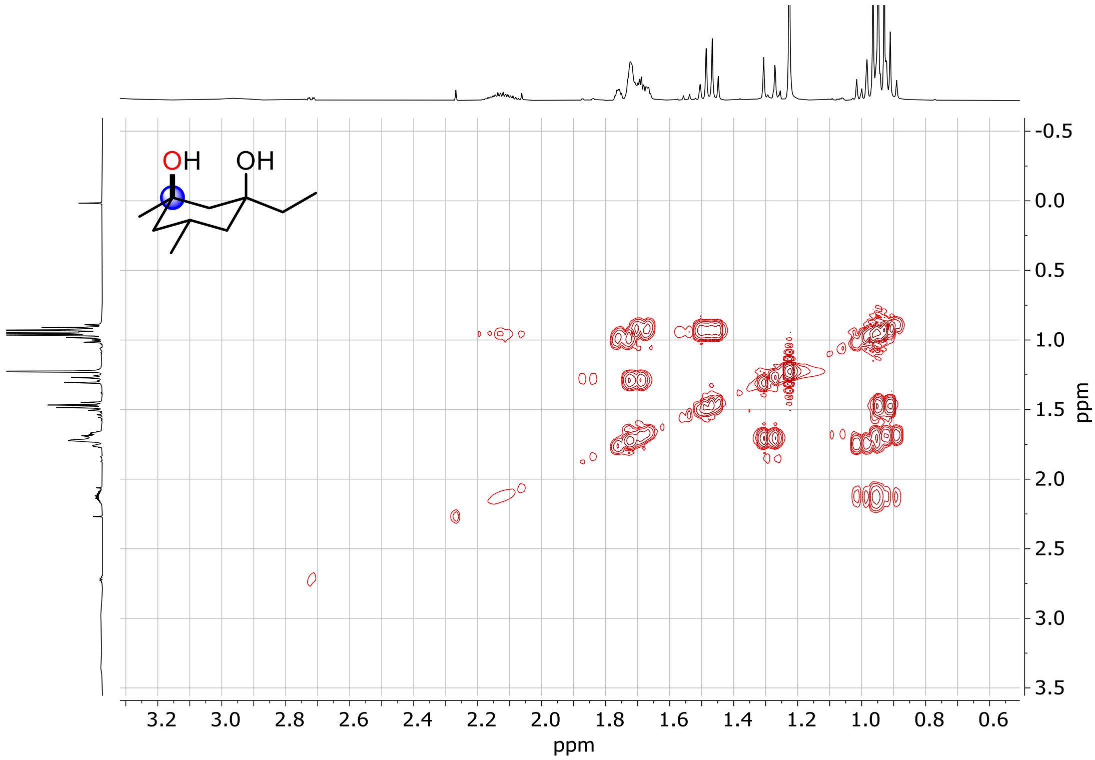

HSQC-NMR of **24b** in $CDCl_3$

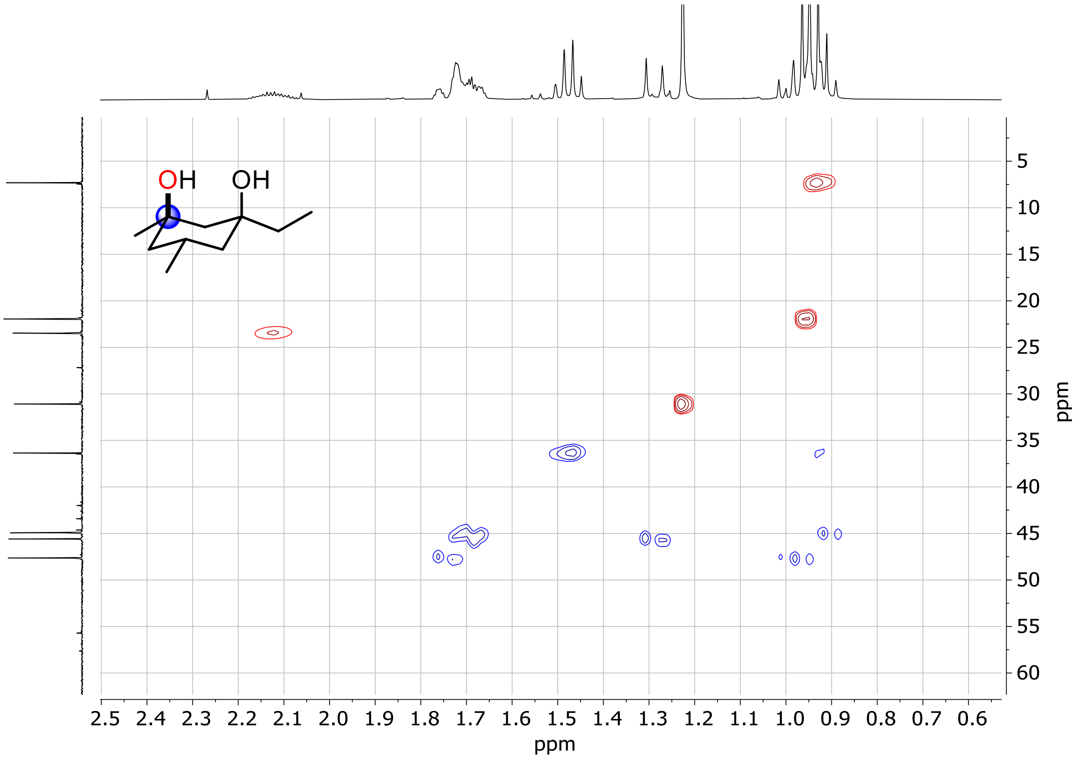

^{1}H-NMR of **25b** in $CDCl_3$

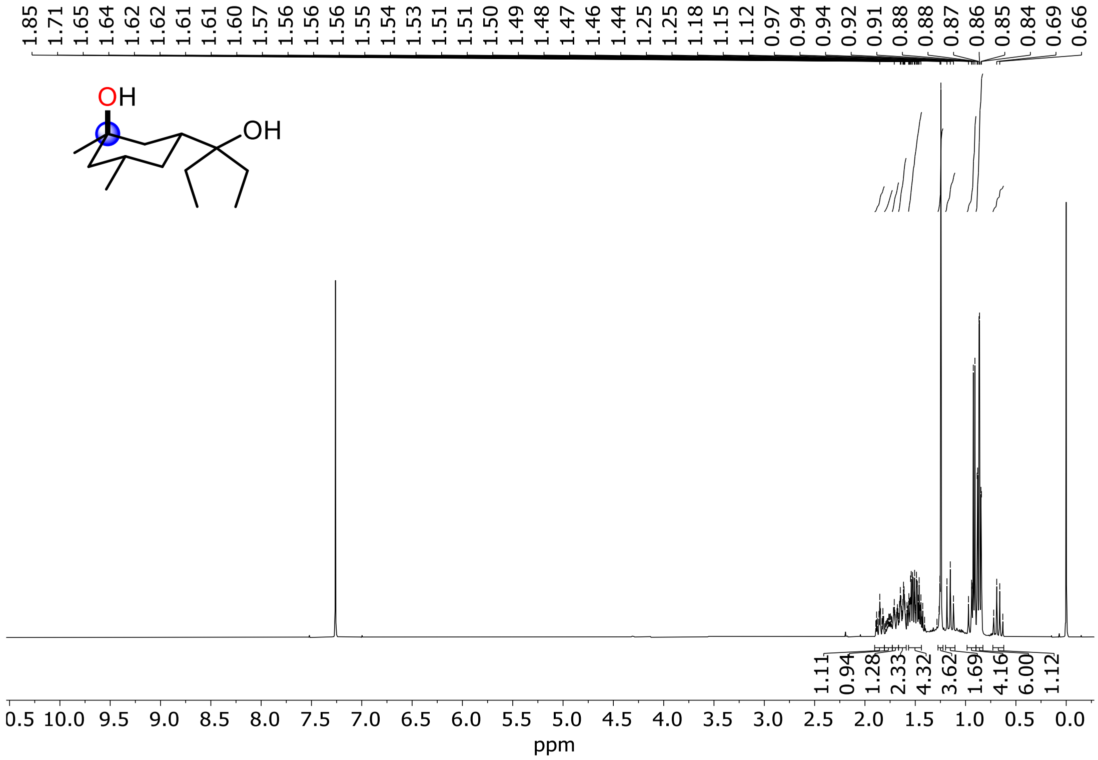

^{13}C-NMR of **25b** in $CDCl_3$

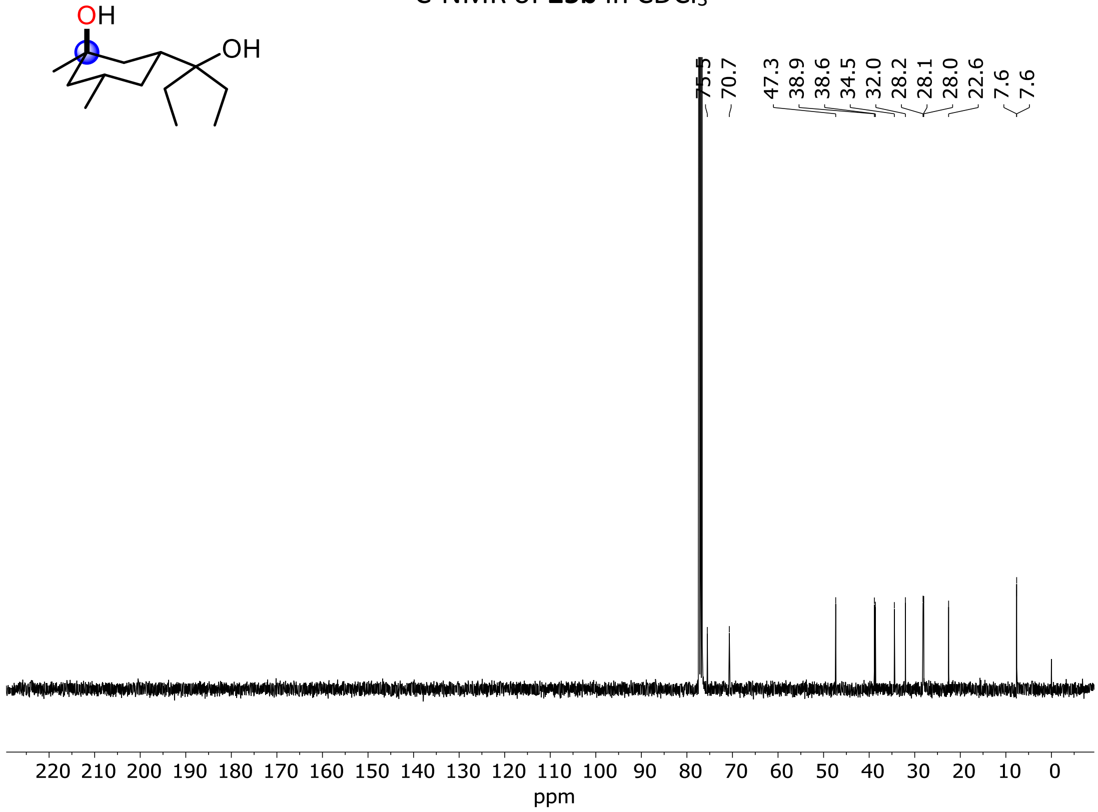

COSY-NMR of **25b** in $CDCl_3$

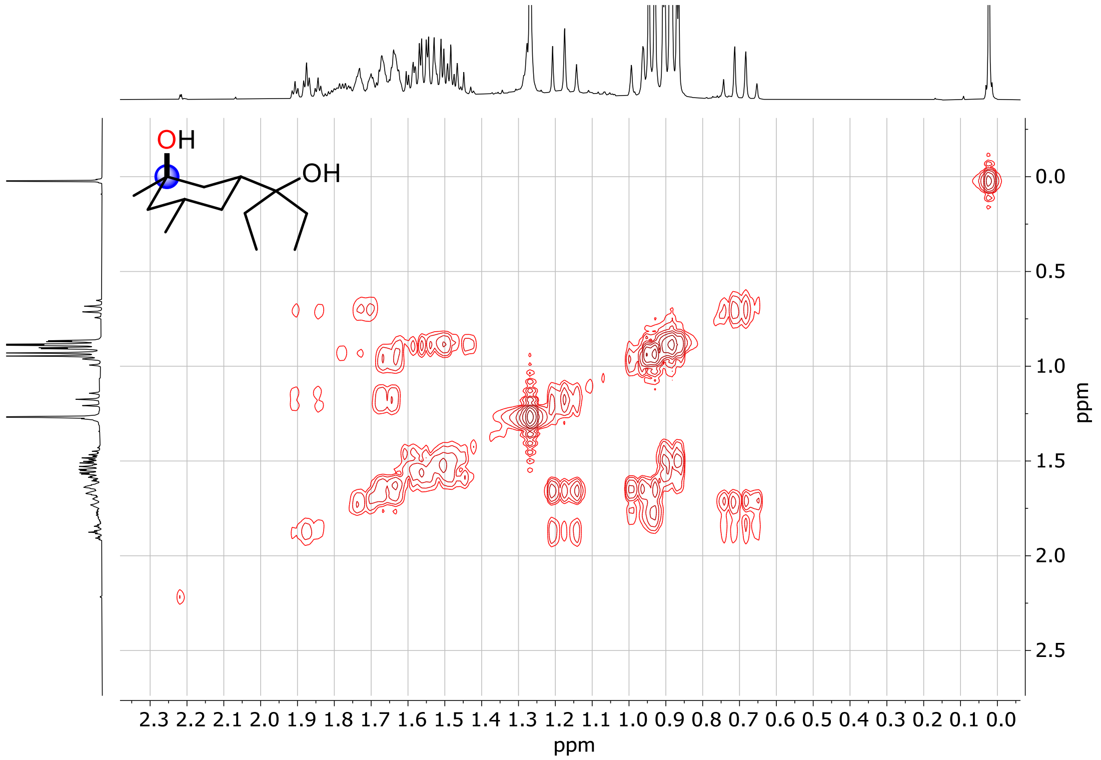

HSQC-NMR of **25b** in $CDCl_3$

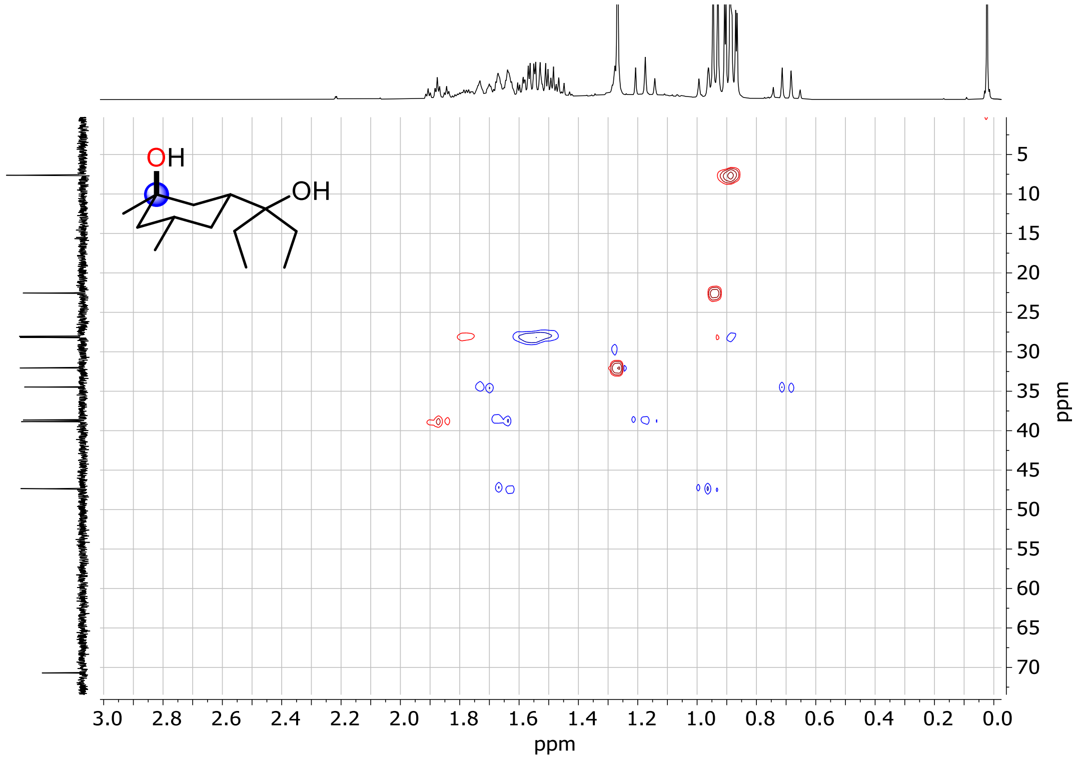

^{1}H-NMR of **26b** in $CDCl_3$

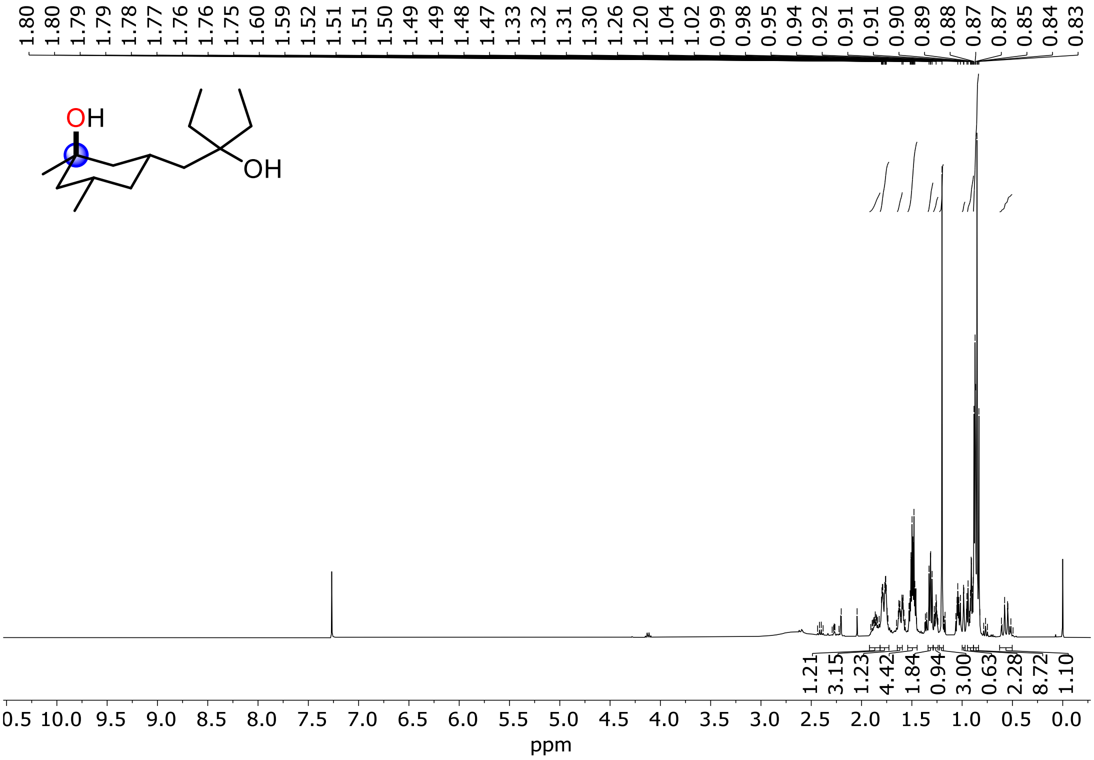

^{13}C-NMR of **26b** in $CDCl_3$

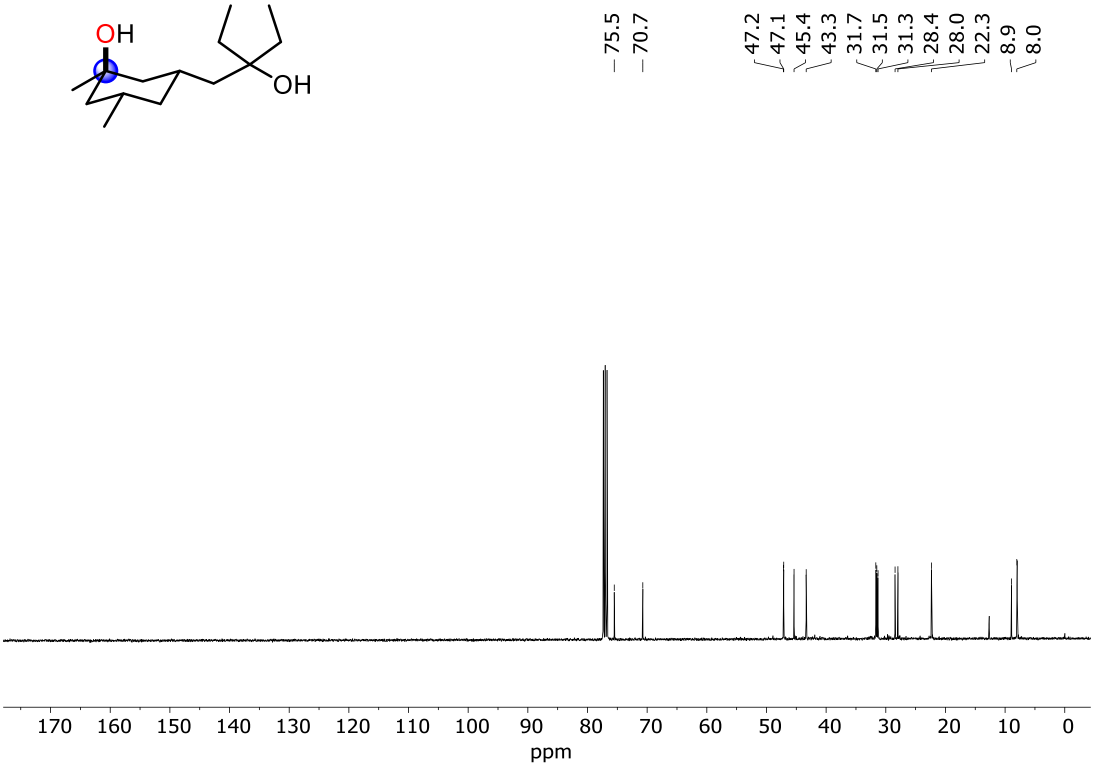

COSY-NMR of **26b** in $CDCl_3$

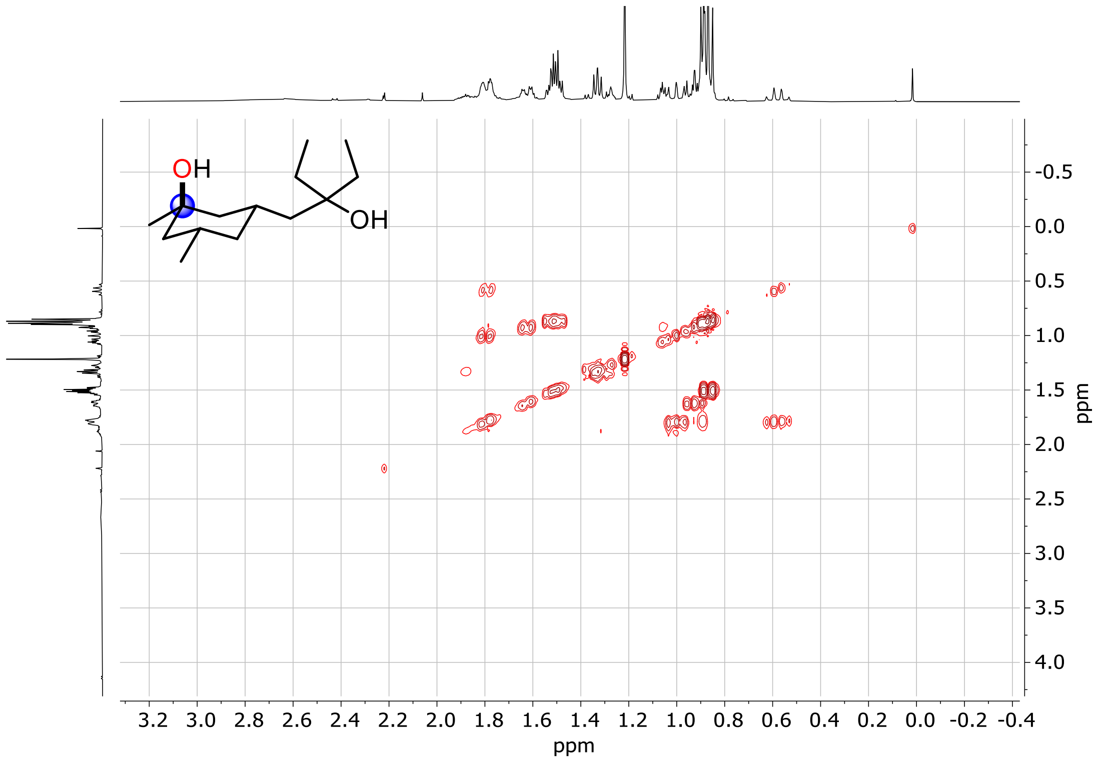

HSQC-NMR of **26b** in $CDCl_3$

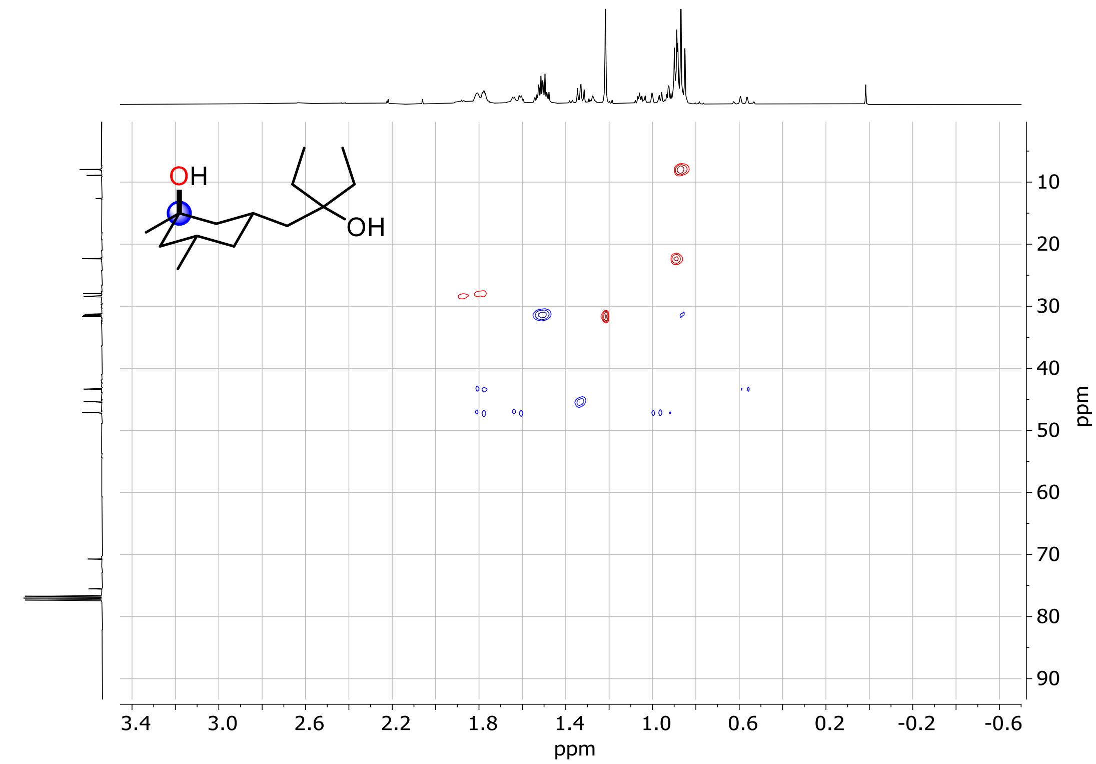

^{1}H-NMR of **27b** in $CDCl_3$

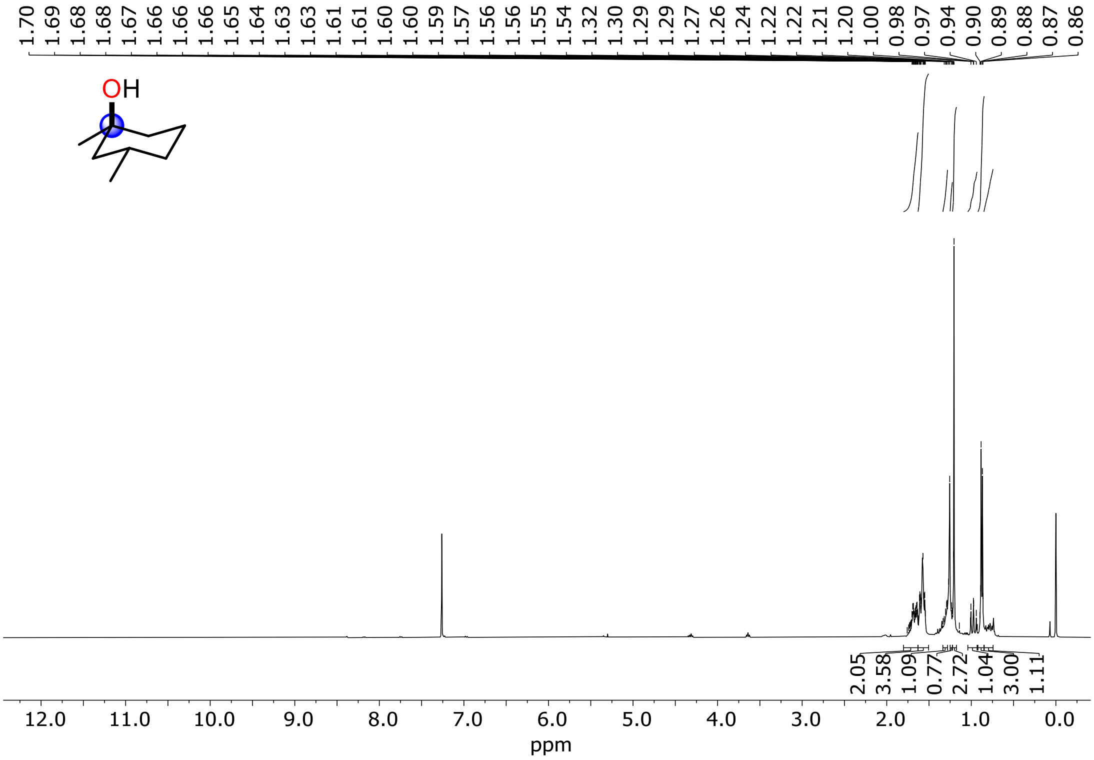

^{13}C-NMR of **27b** in $CDCl_3$

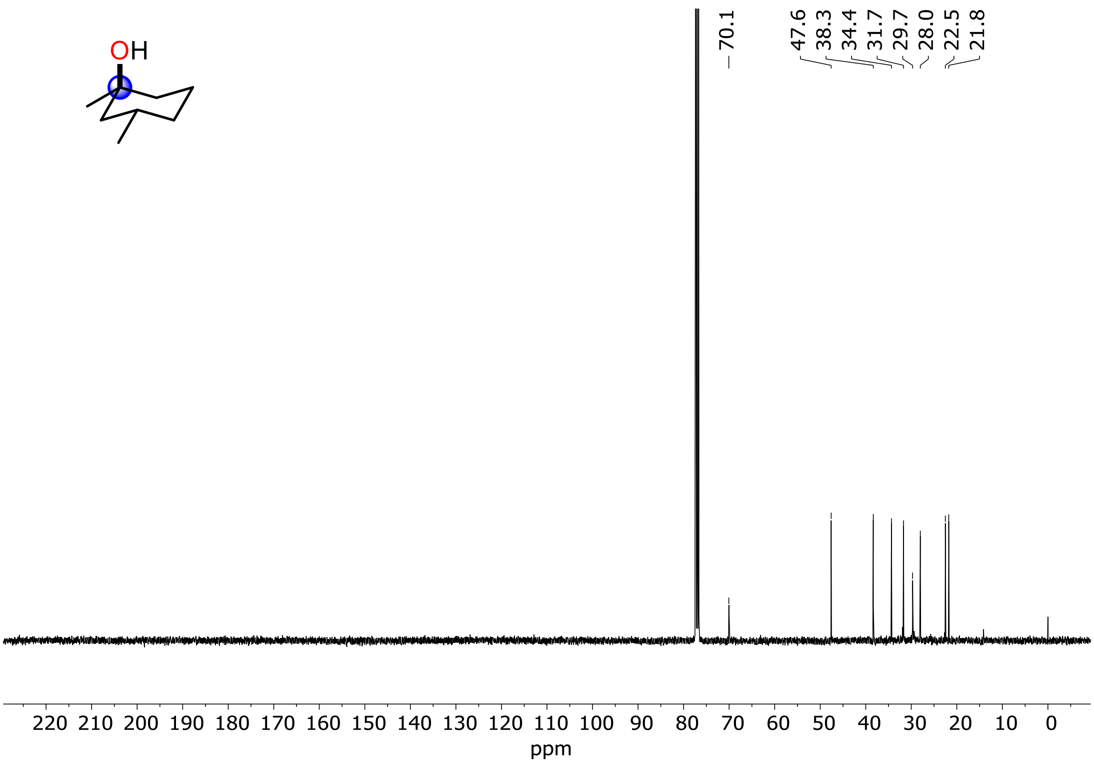

COSY-NMR of **27b** in $CDCl_3$

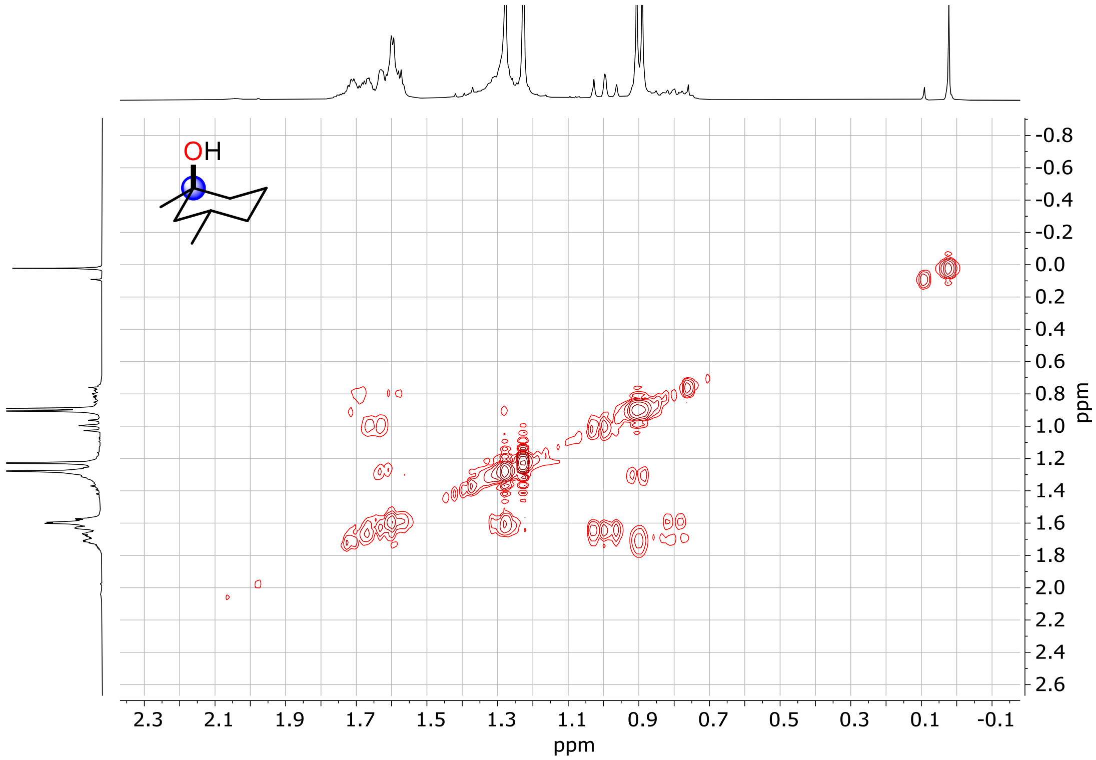

HSQC-NMR of **27b** in $CDCl_3$

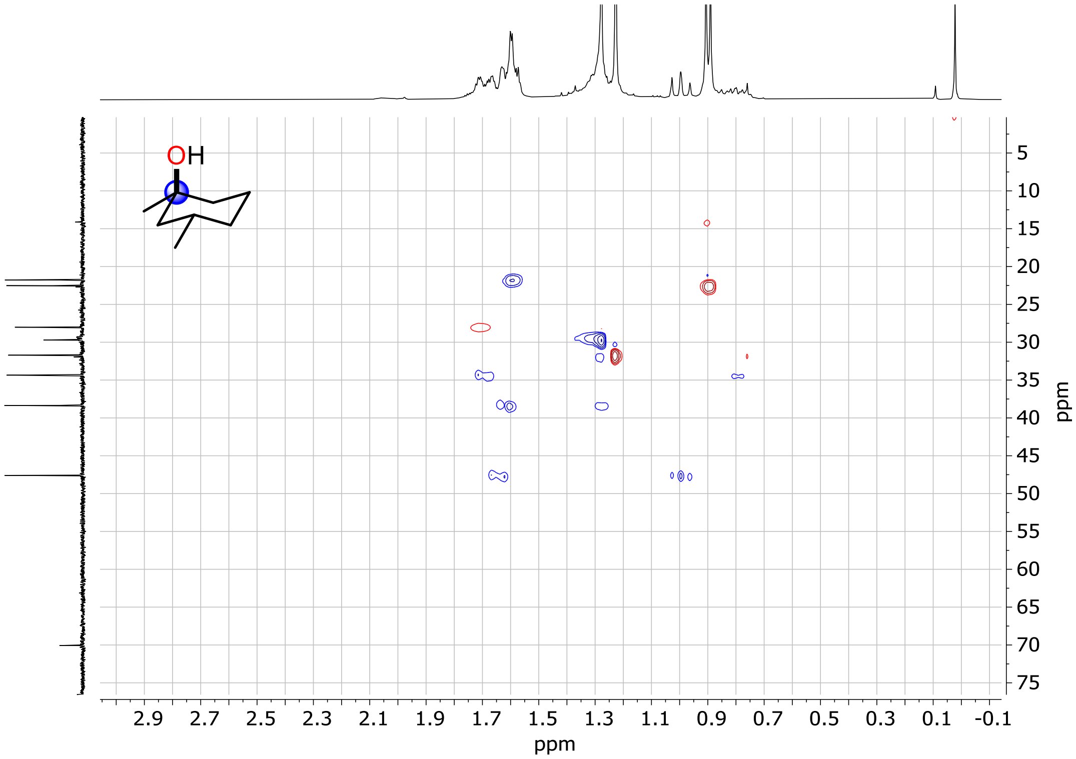

^{1}H-NMR of **28b** in CDCl$_3$

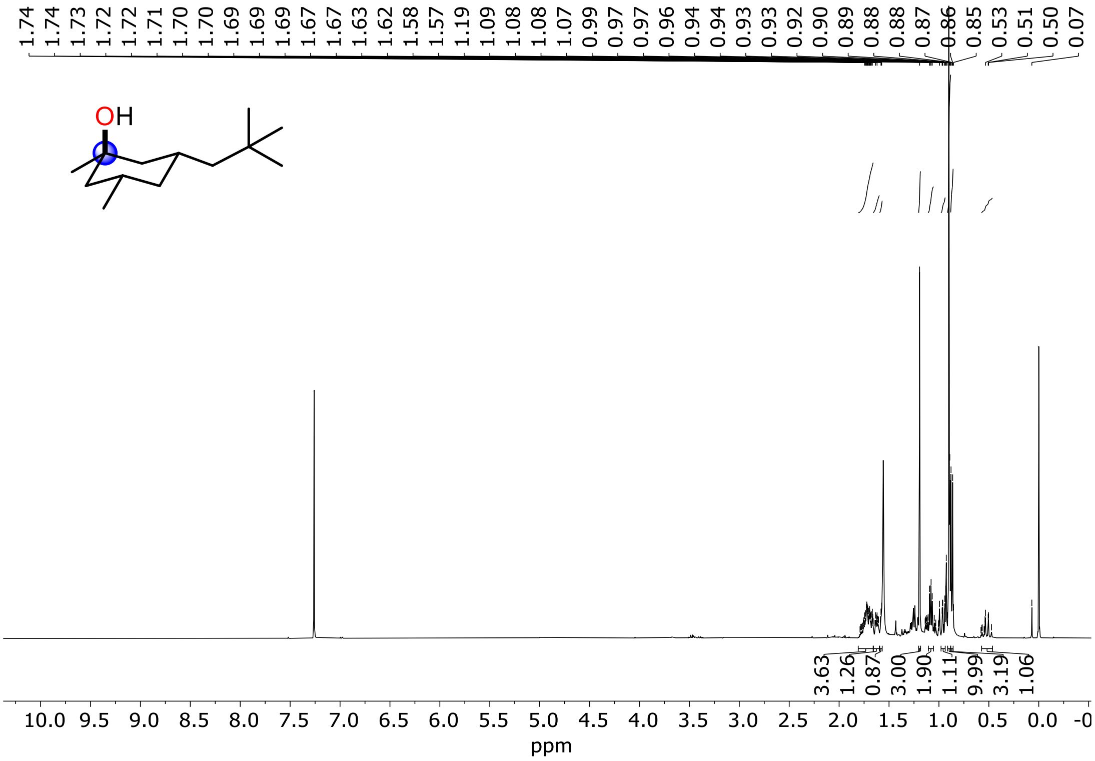

^{13}C-NMR of **28b** in CDCl$_3$

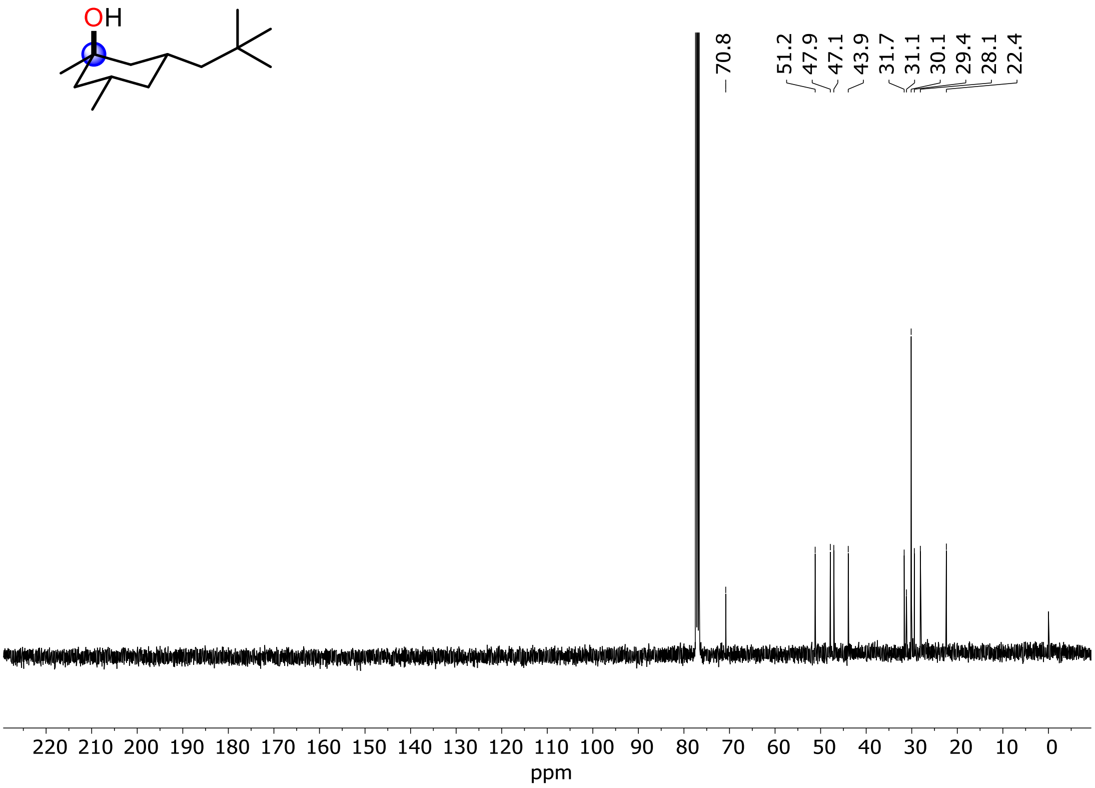

COSY-NMR of **28b** in $CDCl_3$

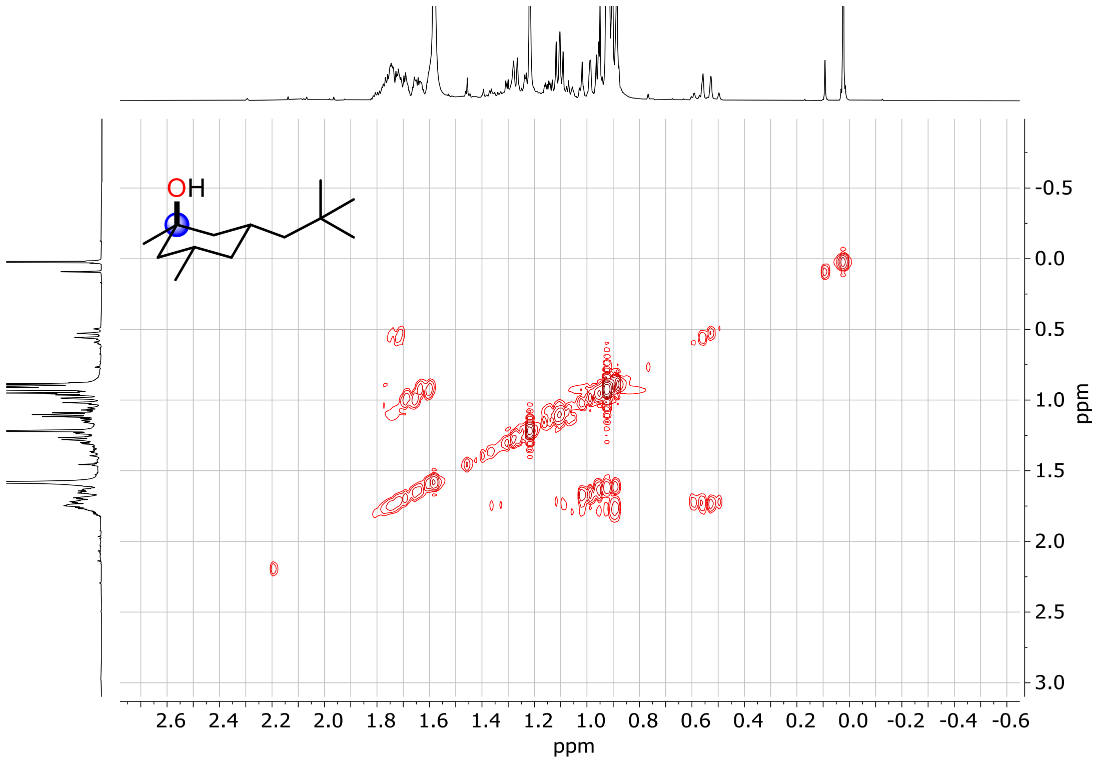

HSQC-NMR of **28b** in $CDCl_3$

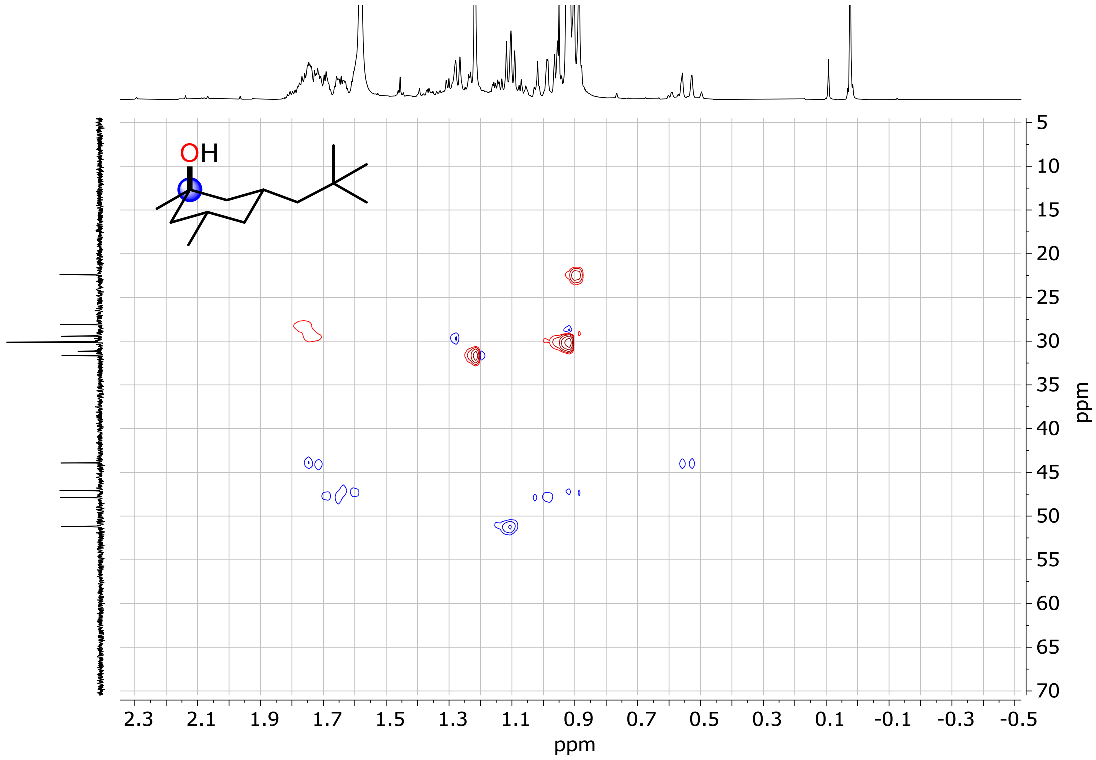

^{1}H-NMR of **29b** in $CDCl_3$

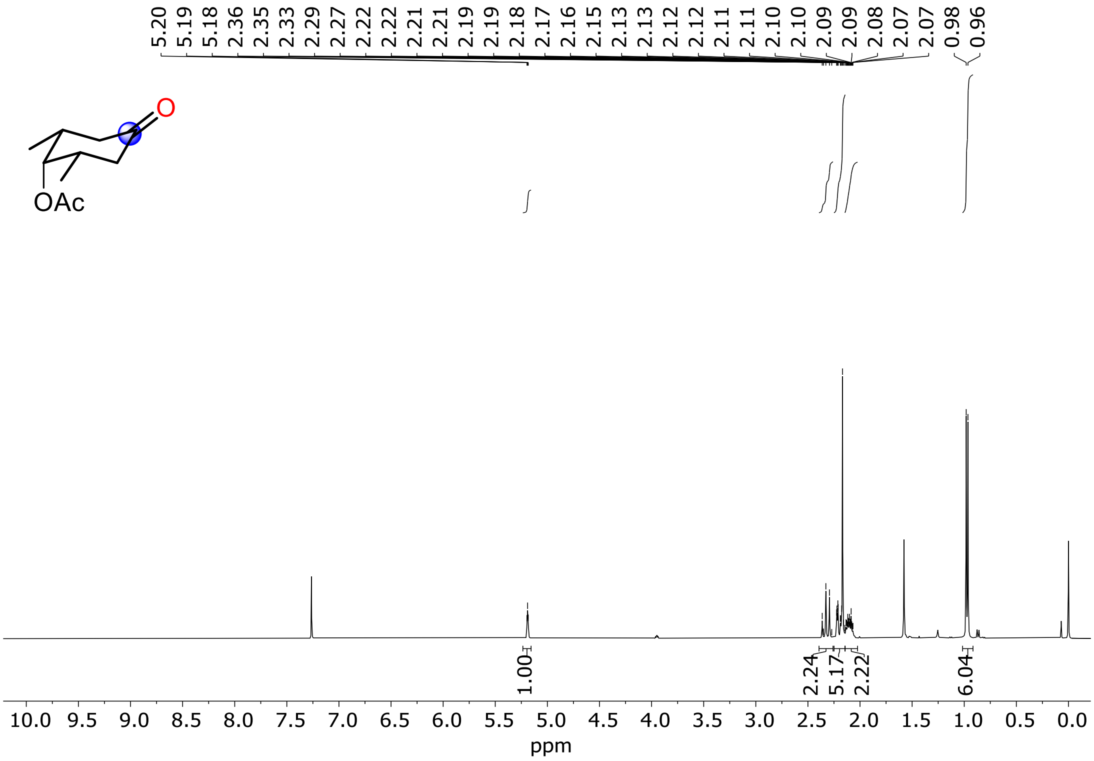

^{13}C-NMR of **29b** in $CDCl_3$

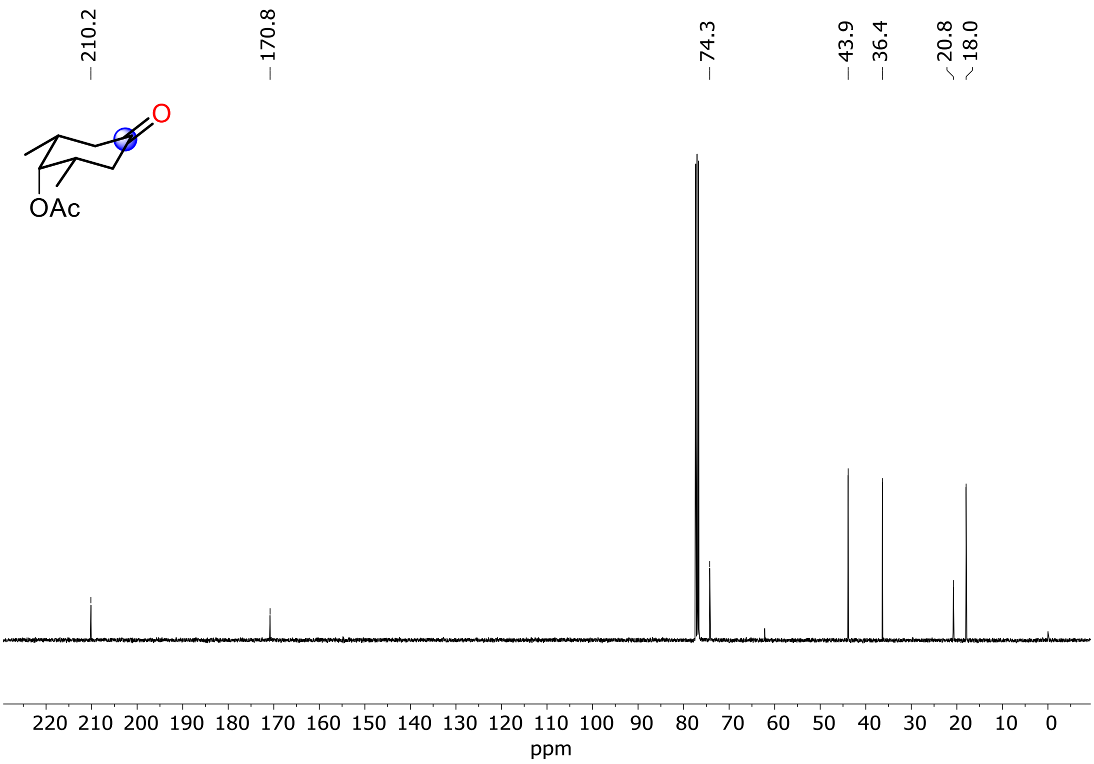

^{1}H-NMR of **30b** in $CDCl_3$

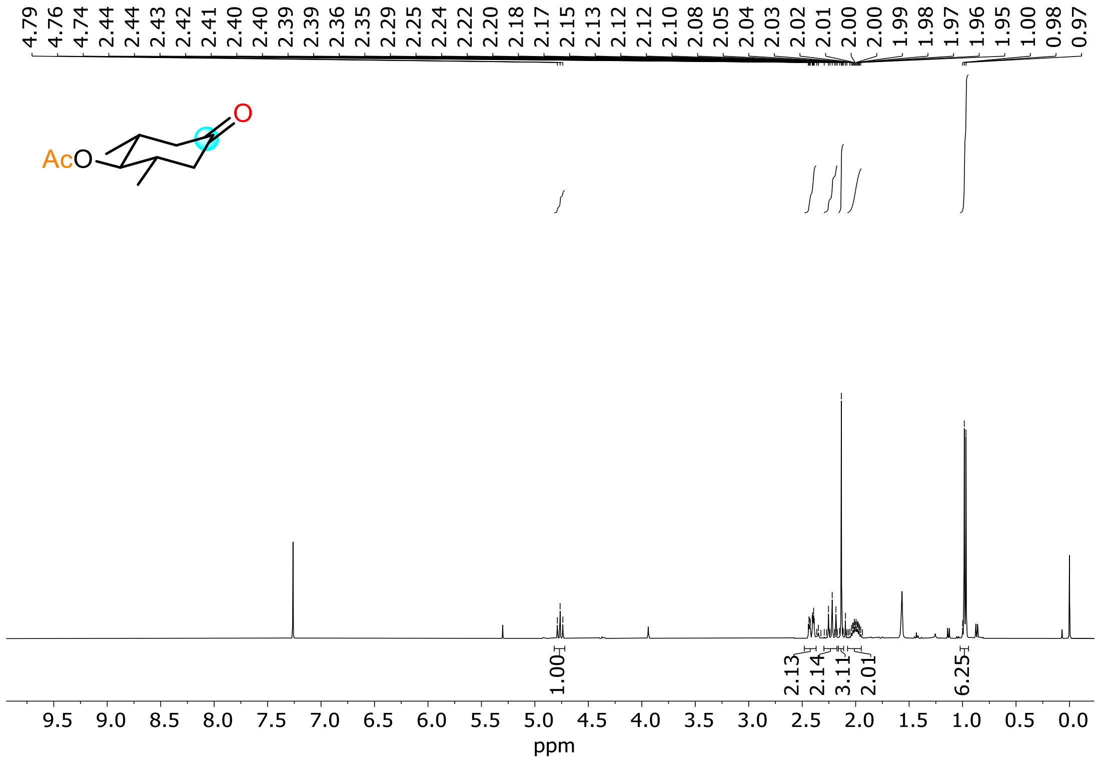

^{13}C-NMR of **30b** in $CDCl_3$

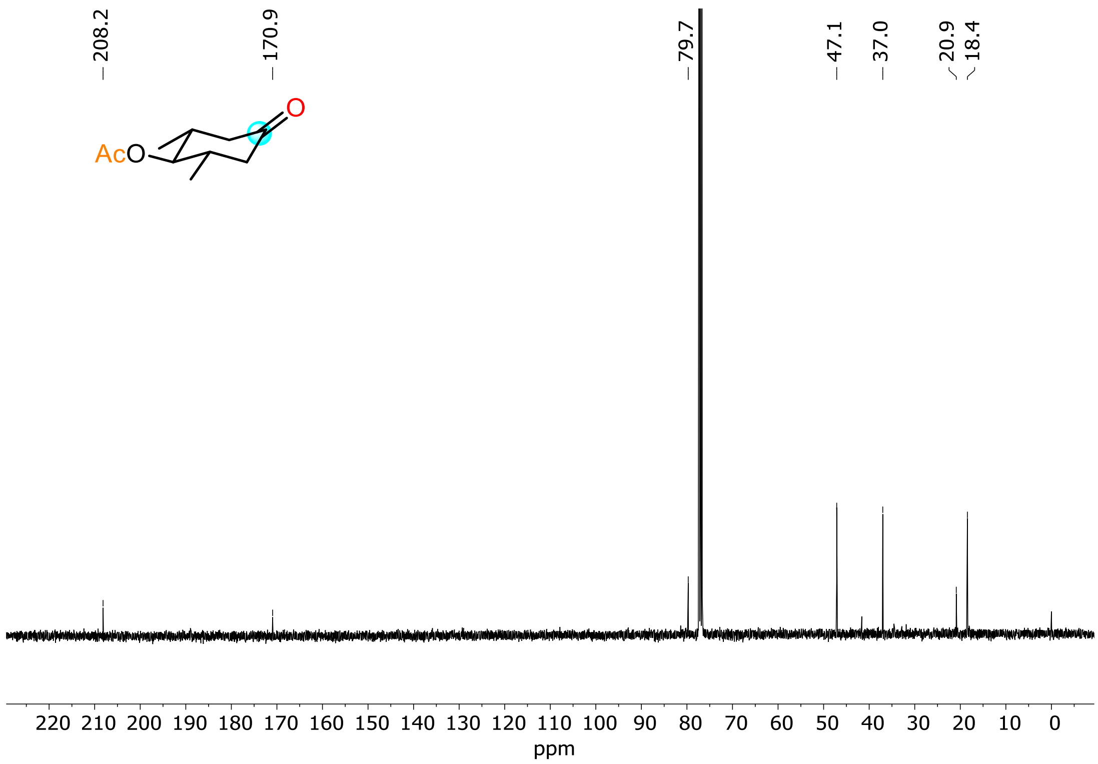

^{1}H-NMR of **30c** in $CDCl_3$

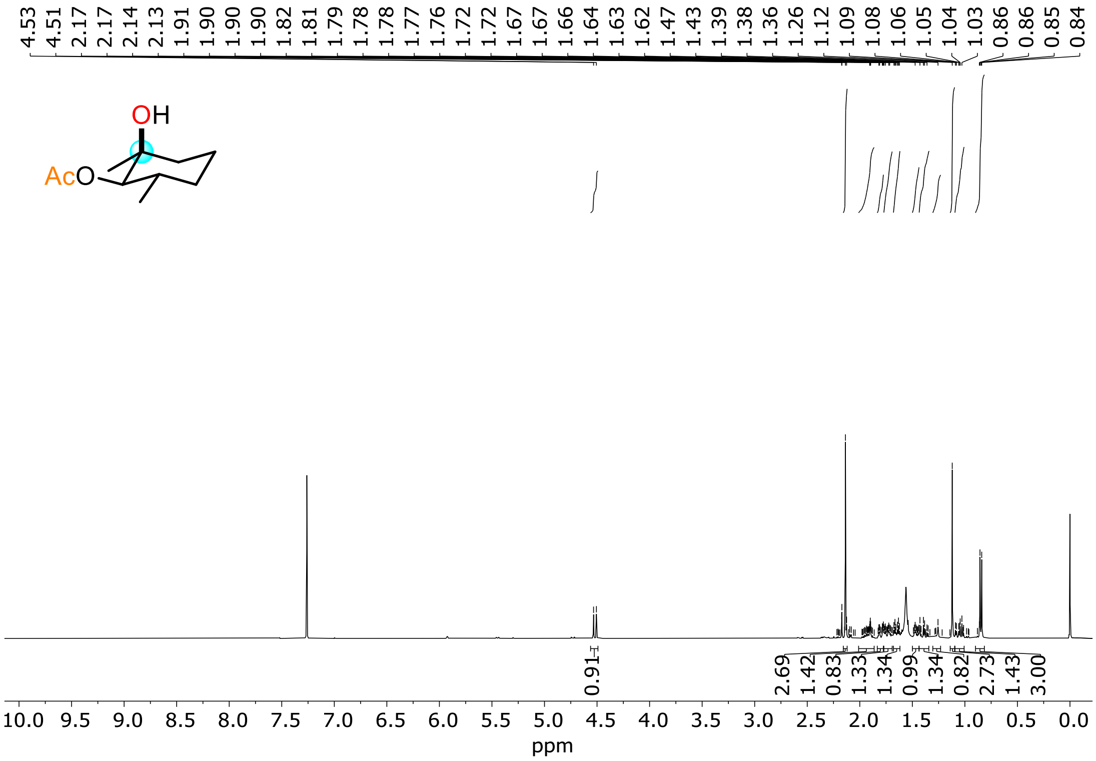

^{13}C-NMR of **30c** in $CDCl_3$

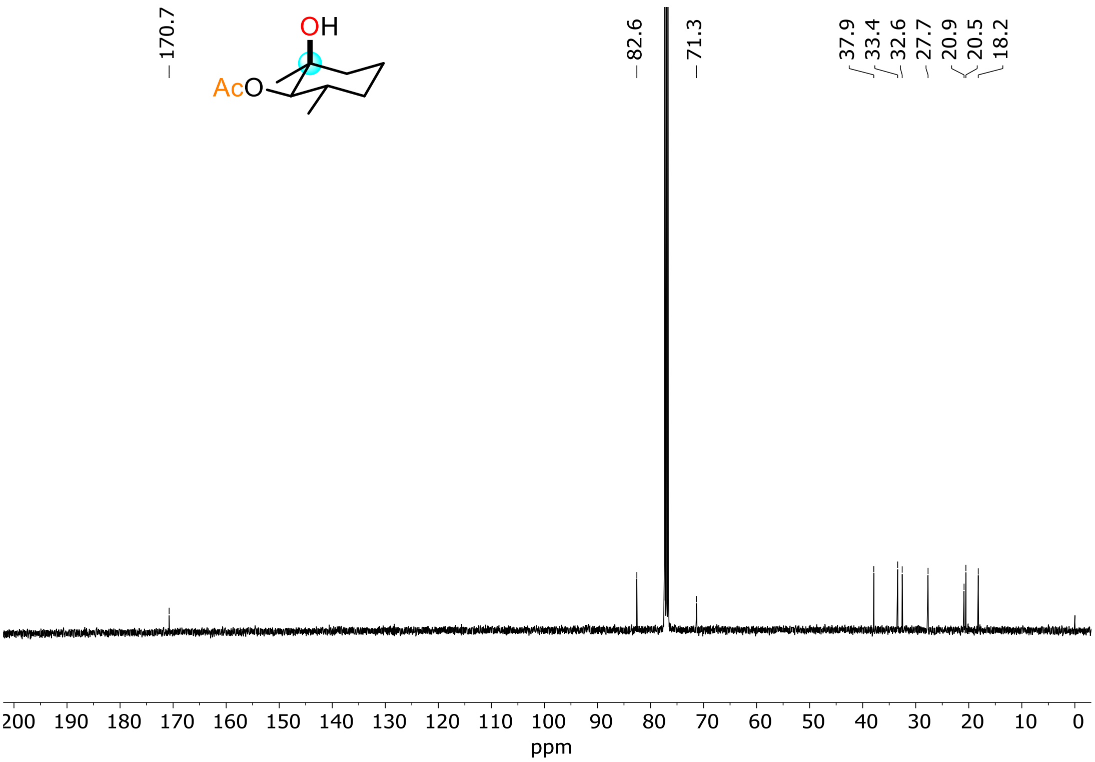

COSY-NMR of **30c** in $CDCl_3$

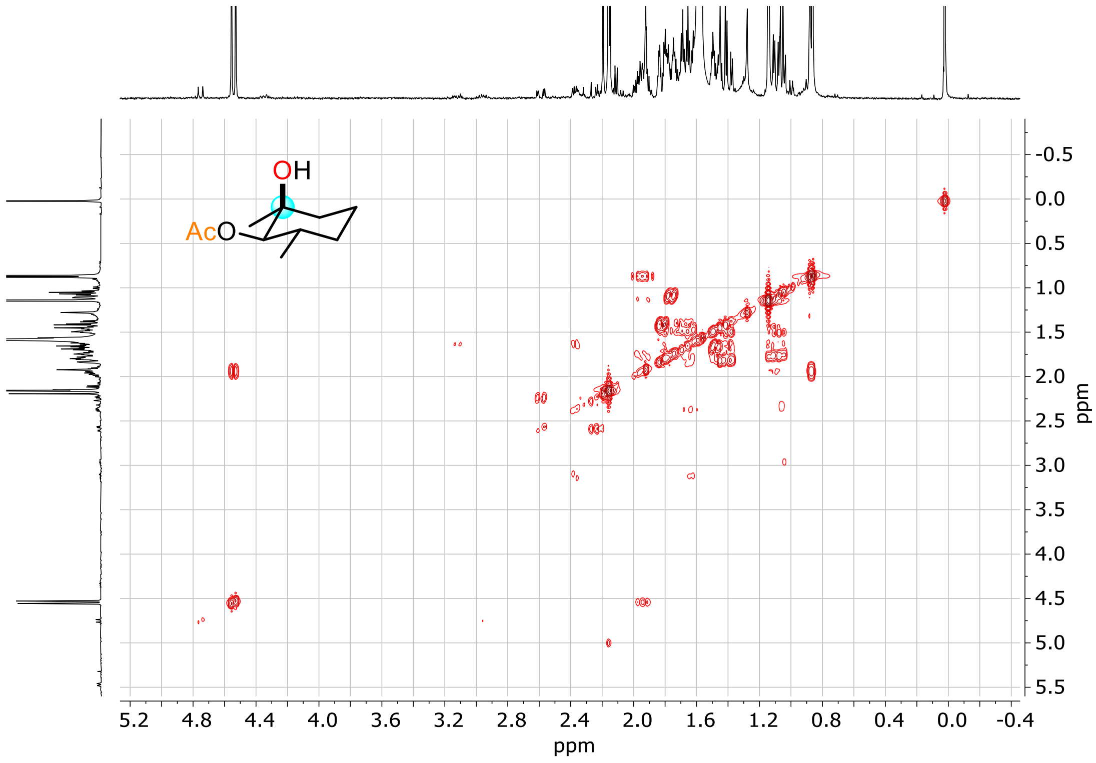

HSQC-NMR of **30c** in $CDCl_3$

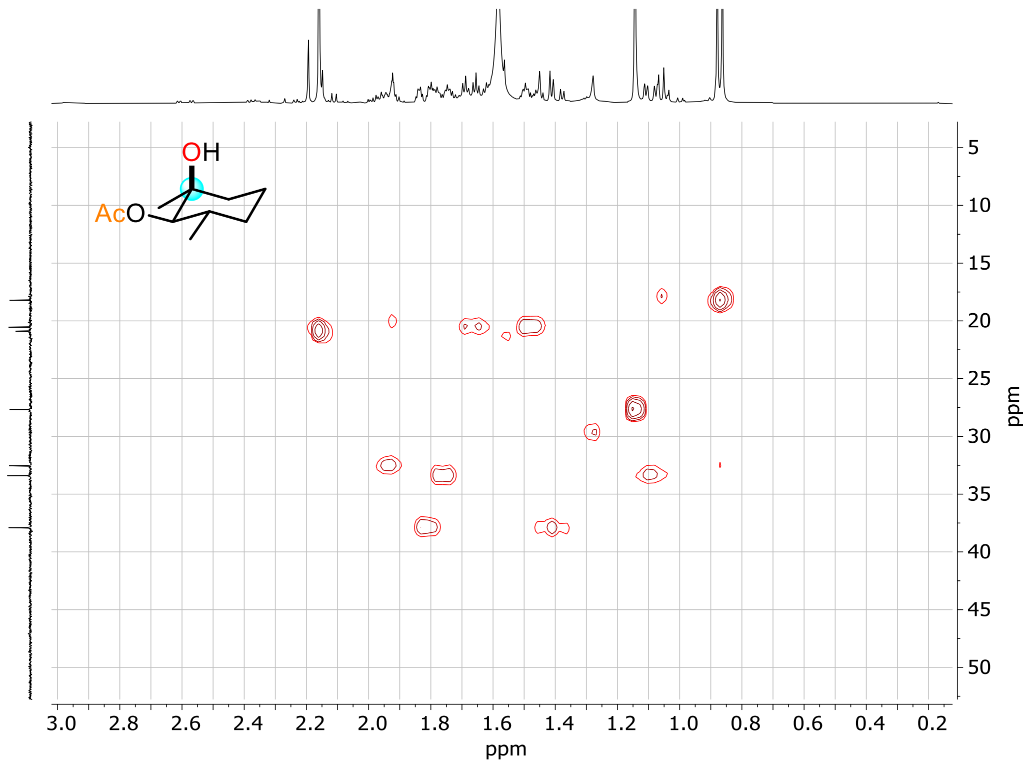

^{1}H-NMR of **30d** in $CDCl_3$

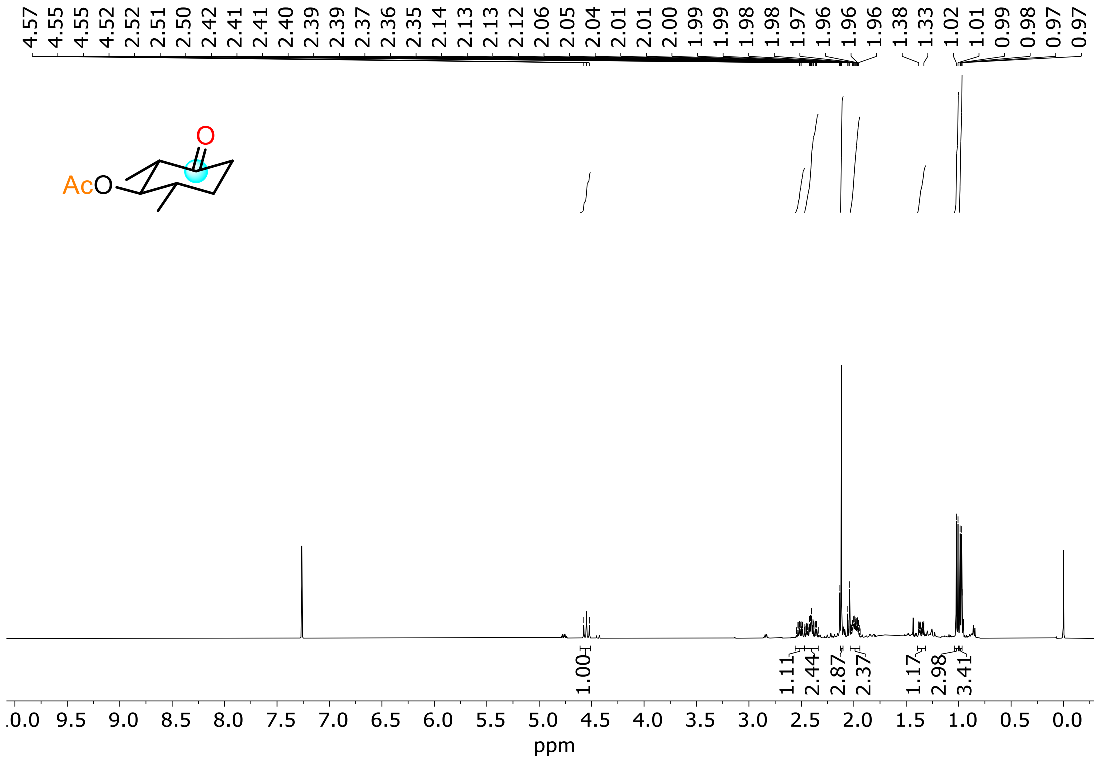

^{13}C-NMR of **30d** in $CDCl_3$

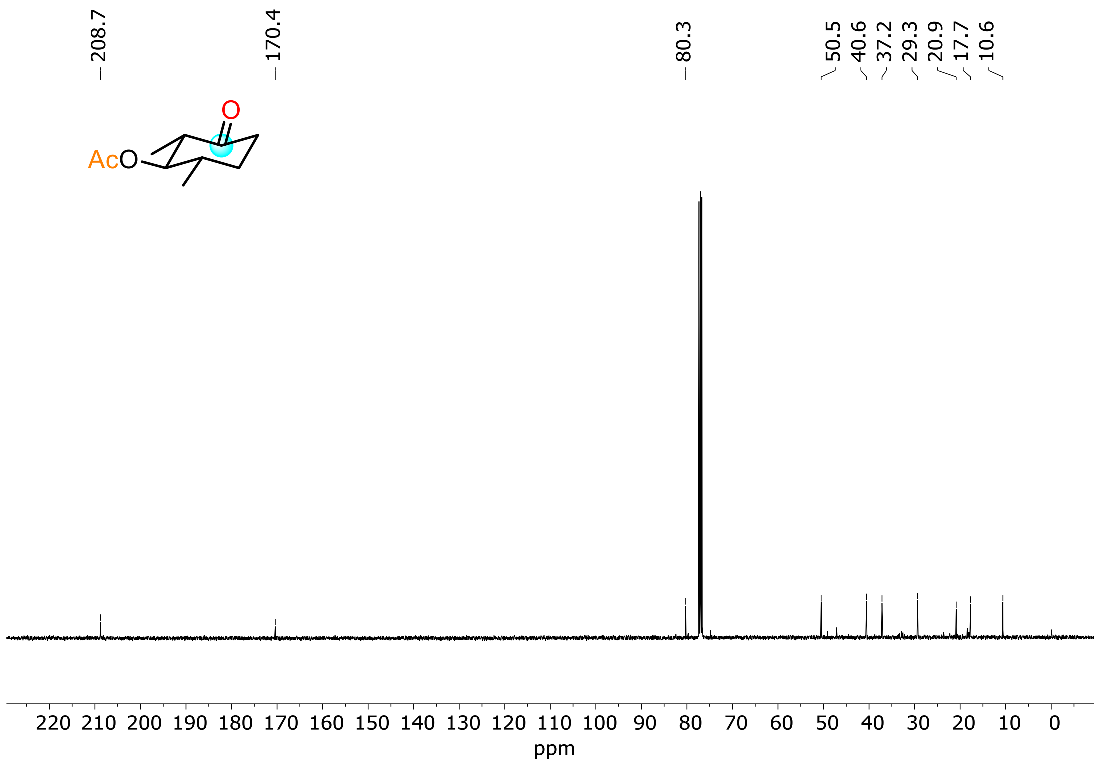

COSY-NMR of **30d** in $CDCl_3$

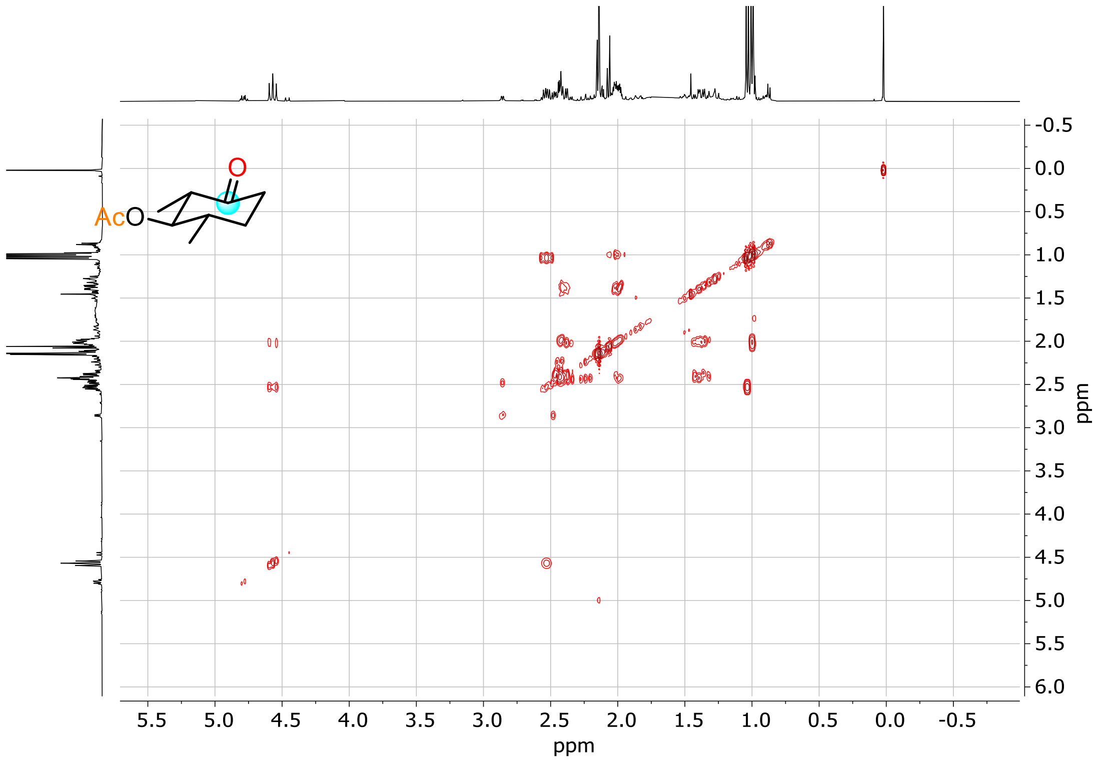

HSQC-NMR of **30d** in $CDCl_3$

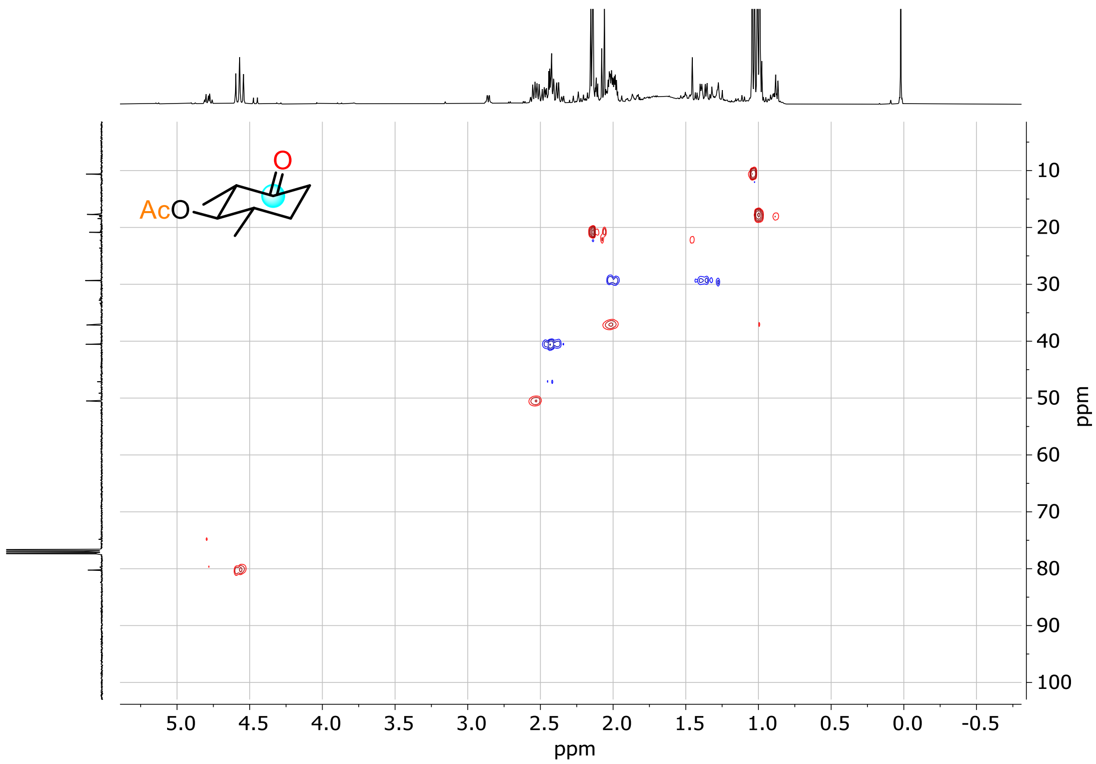

^{1}H-NMR of **31b** in $CDCl_3$

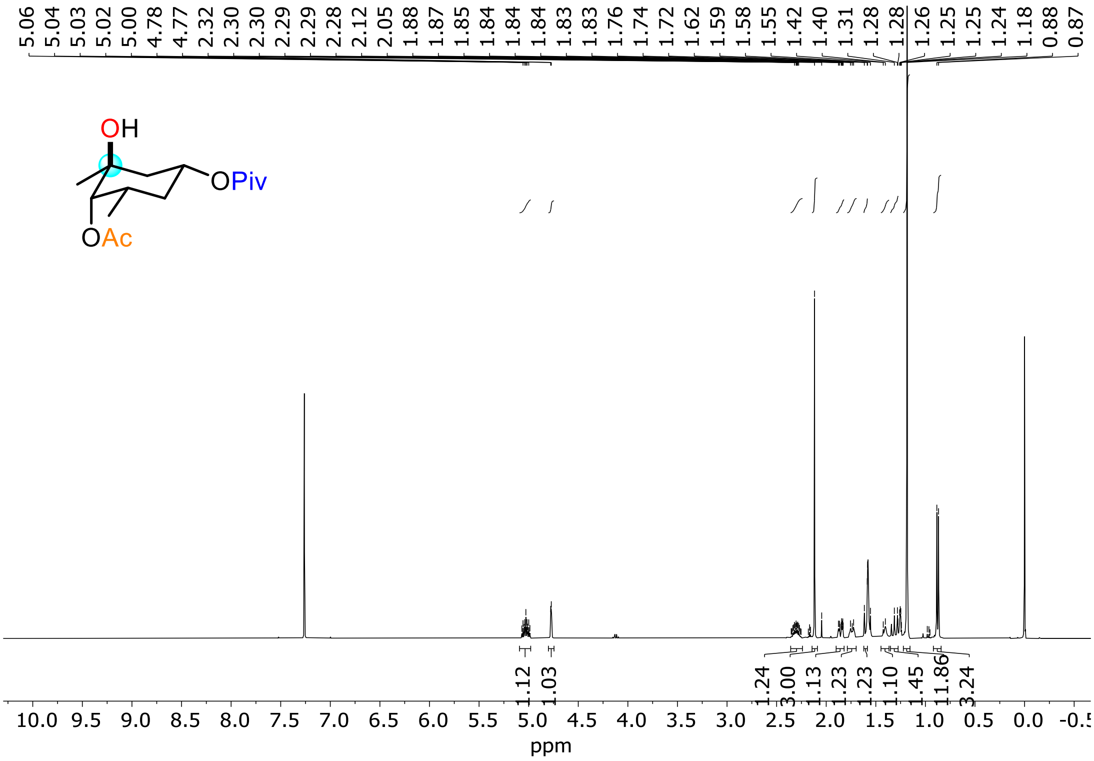

^{13}C-NMR of **31b** in $CDCl_3$

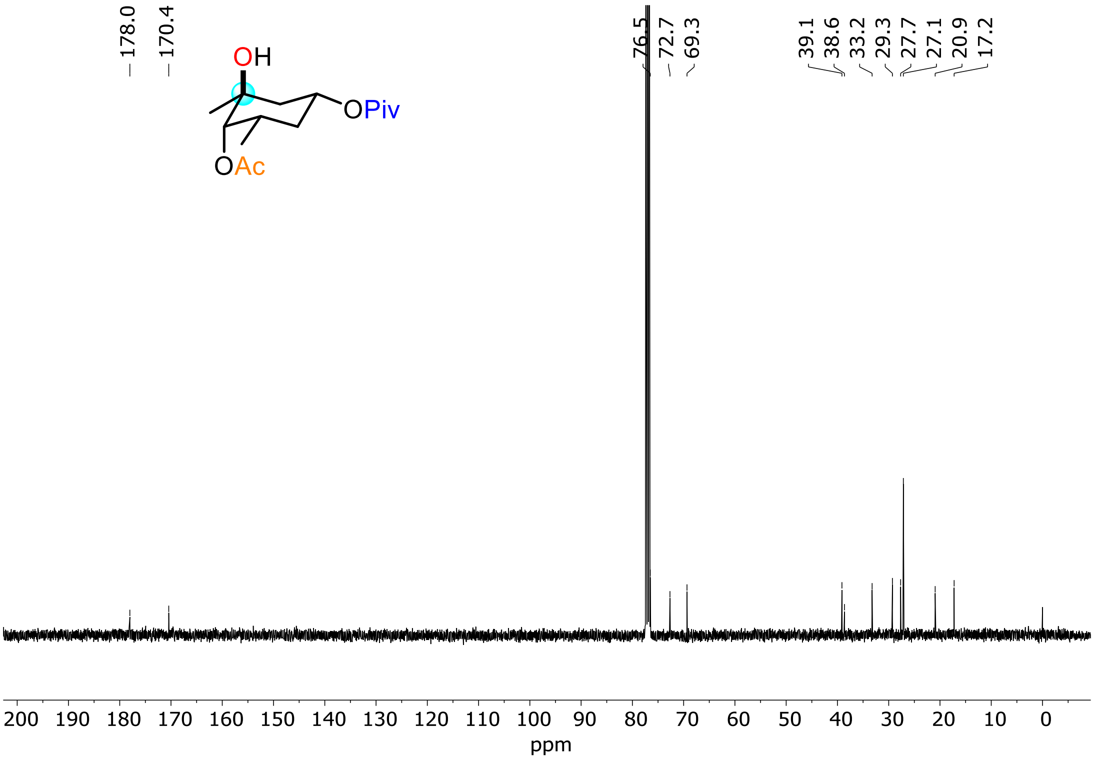

COSY-NMR of **31b** in $CDCl_3$

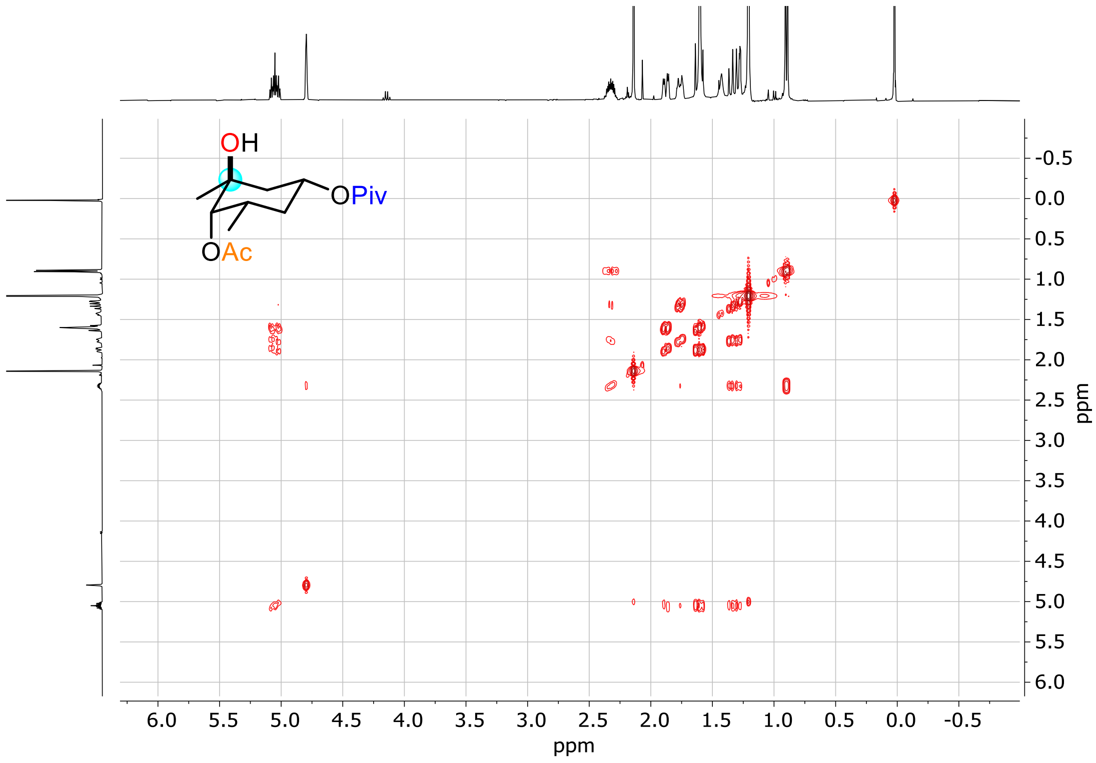

COSY-NMR of **31b** in $CDCl_3$

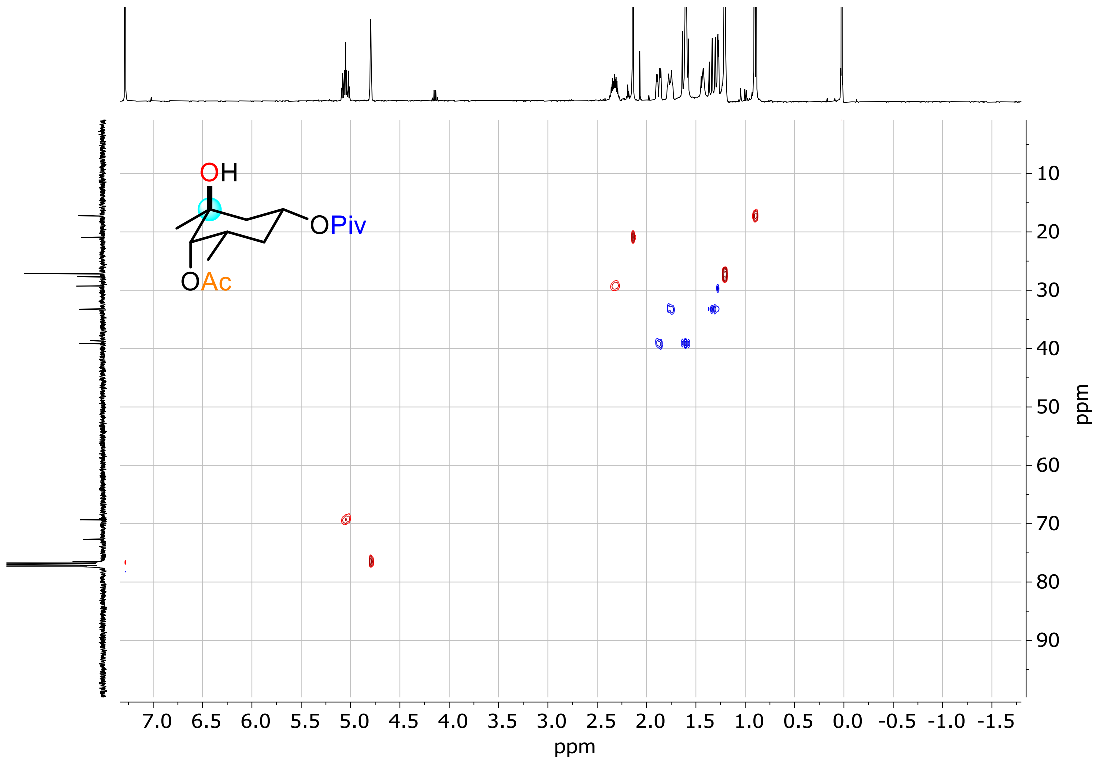

^{1}H-NMR of **6c** in $CDCl_3$

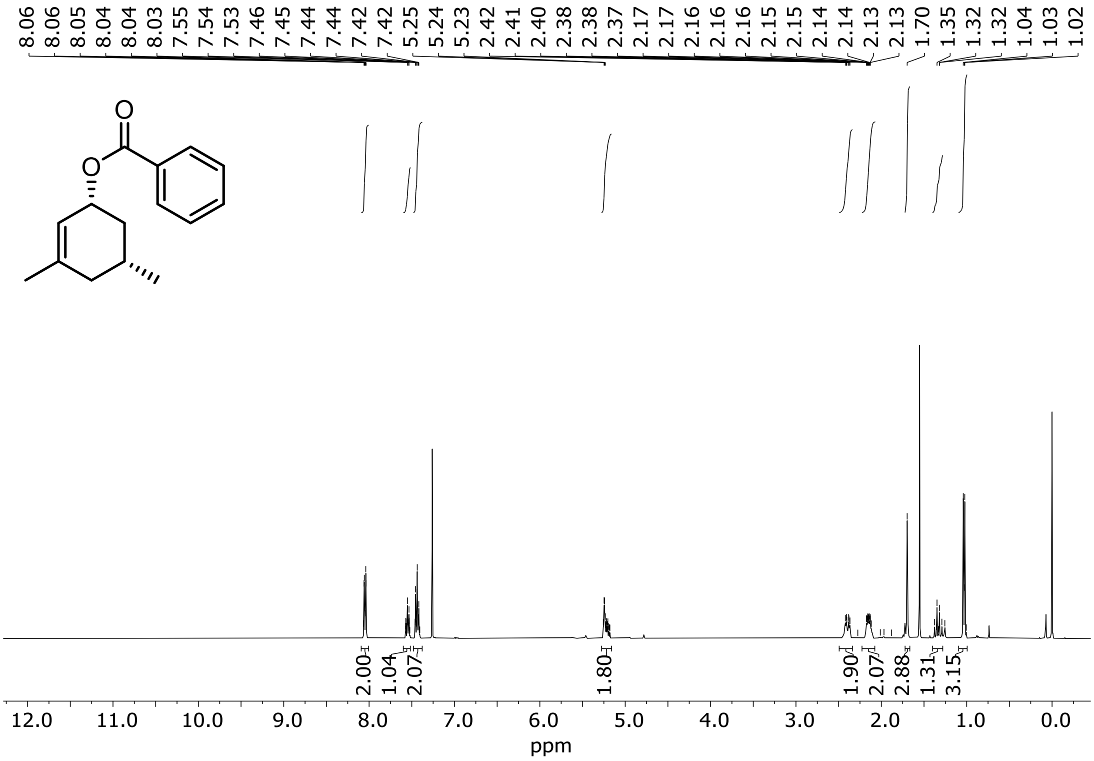

^{13}C-NMR of **6c** in $CDCl_3$

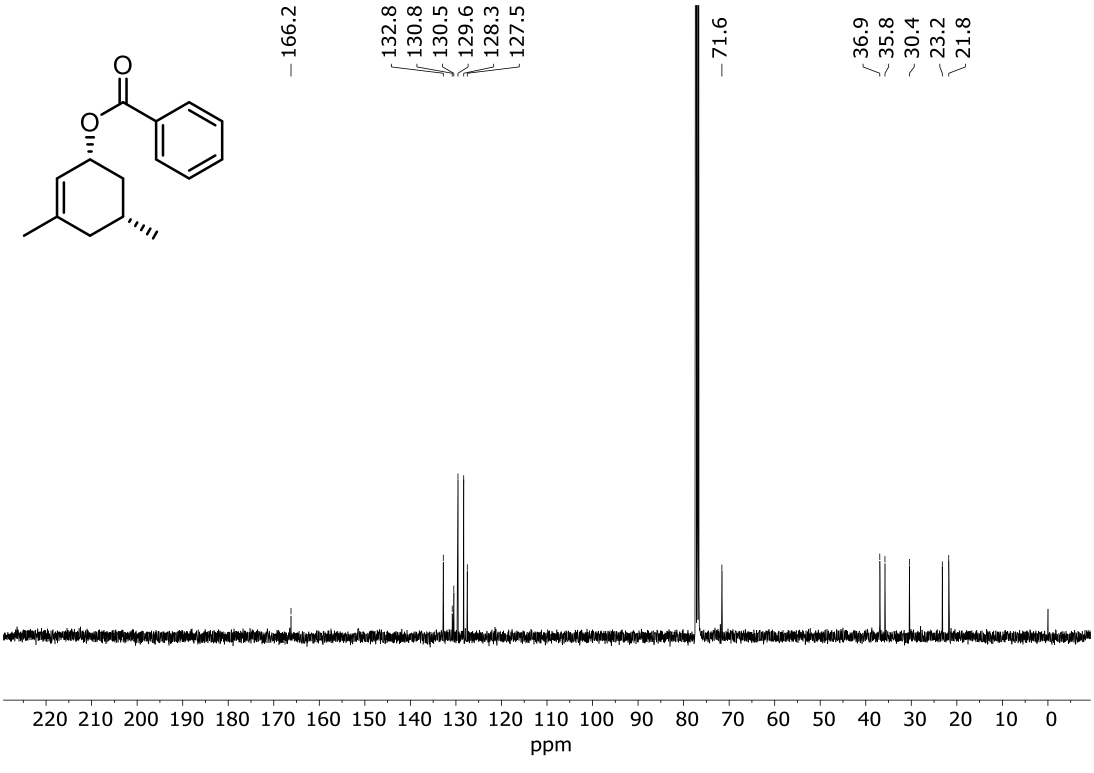

^{1}H-NMR of **6d** in $CDCl_3$

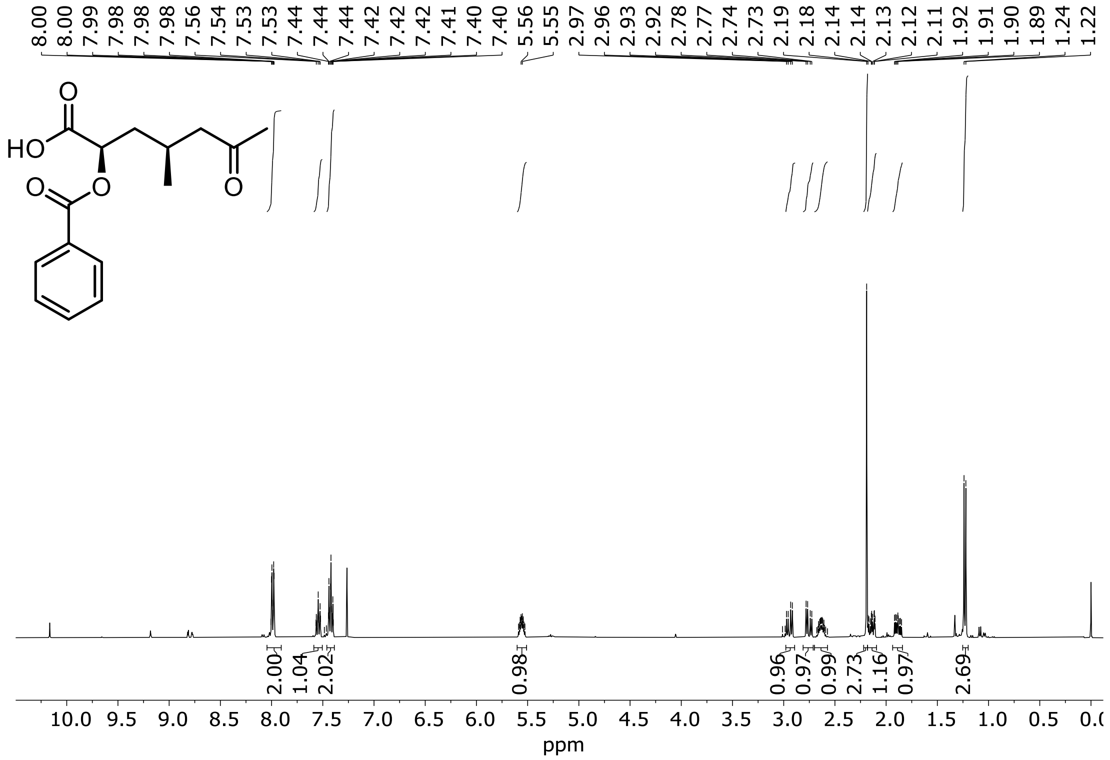

* Aldehyde by-product less than 8 %

^{13}C-NMR of **6d** in $CDCl_3$

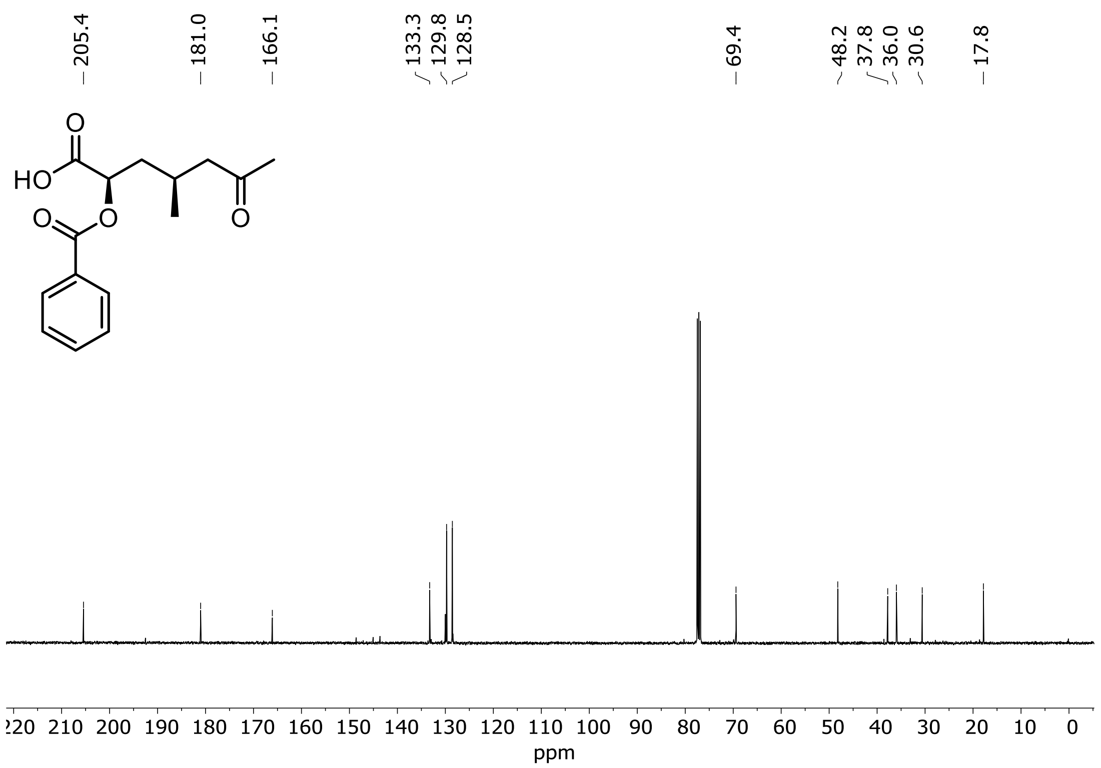

^{1}H-NMR of **6e** in $CDCl_3$

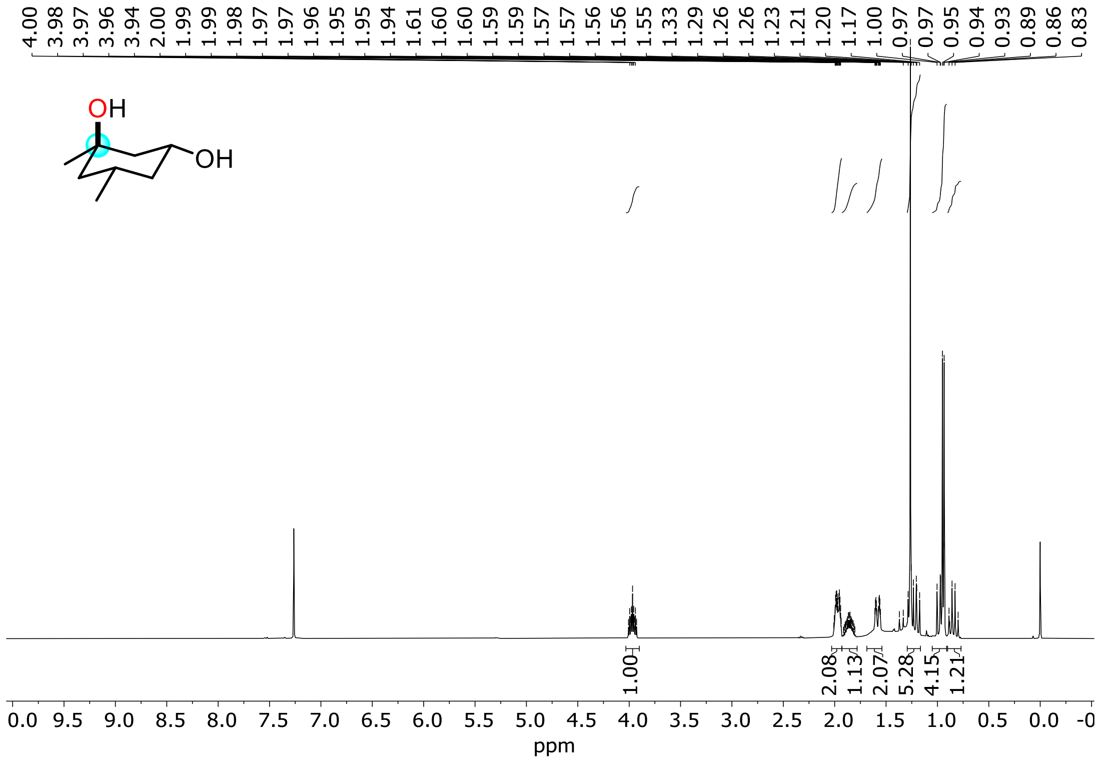

^{13}C-NMR of **6e** in $CDCl_3$

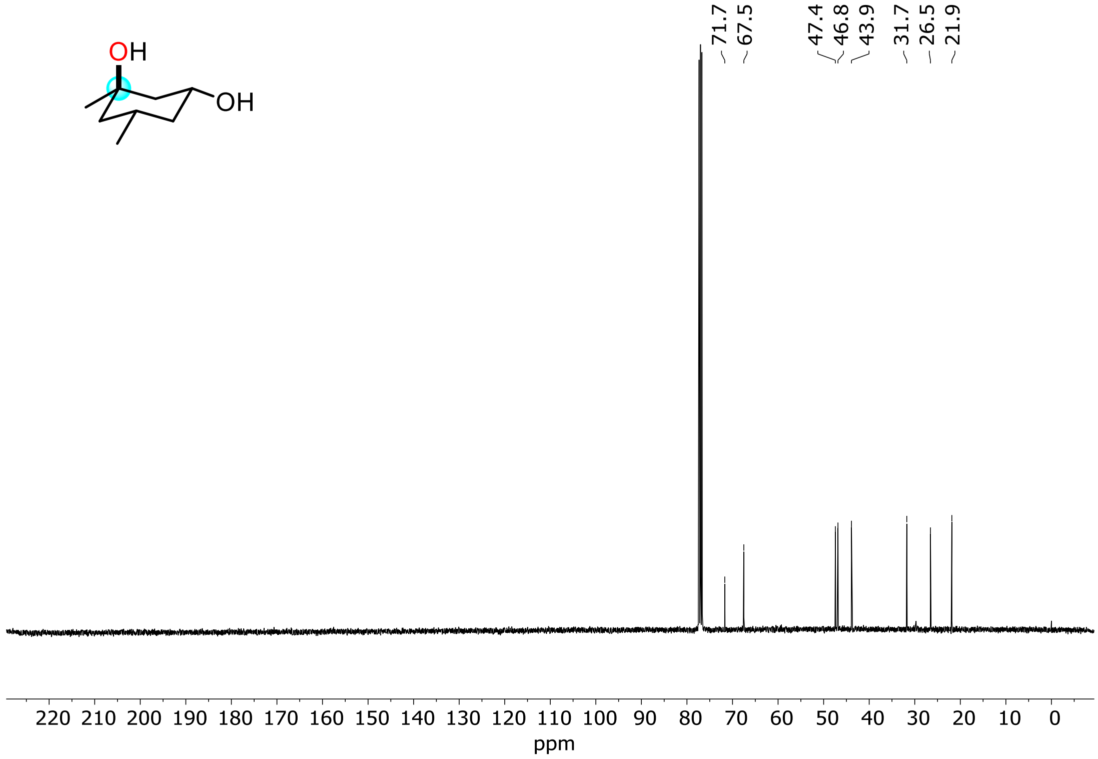

^{1}H-NMR of **16c** in $CDCl_3$

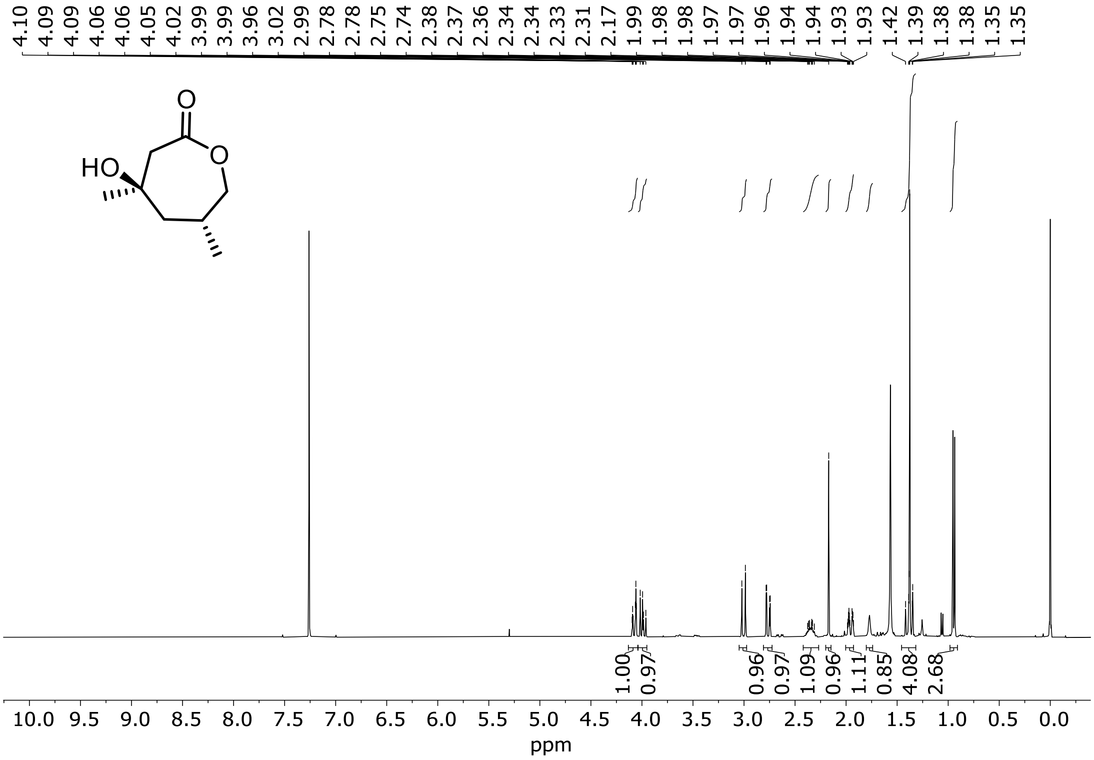

^{13}C-NMR of **16c** in $CDCl_3$

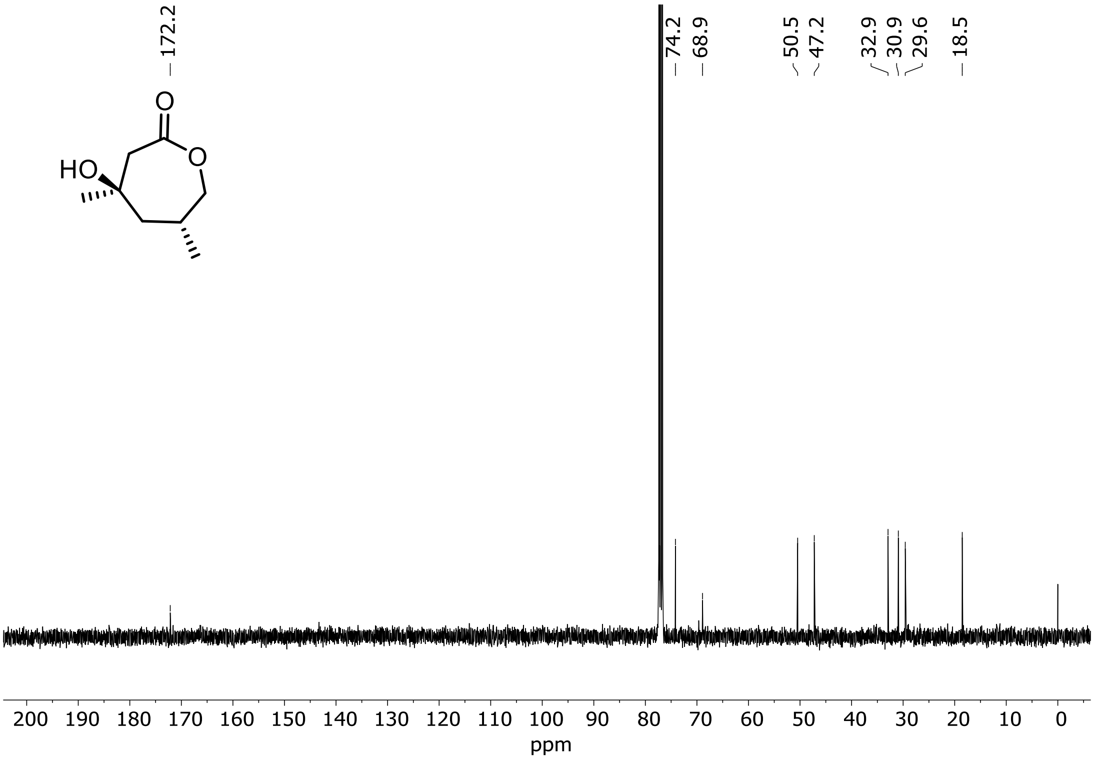

^{1}H-NMR of **16d** in $CDCl_3$

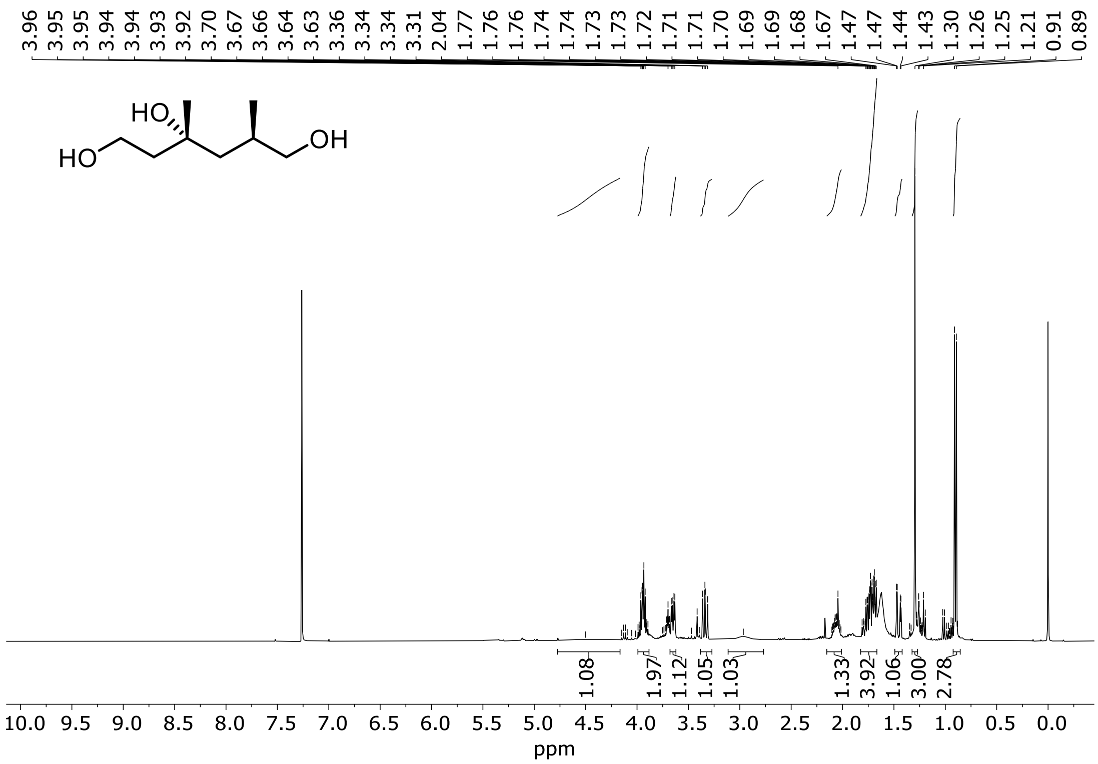

^{13}C-NMR of **16d** in $CDCl_3$

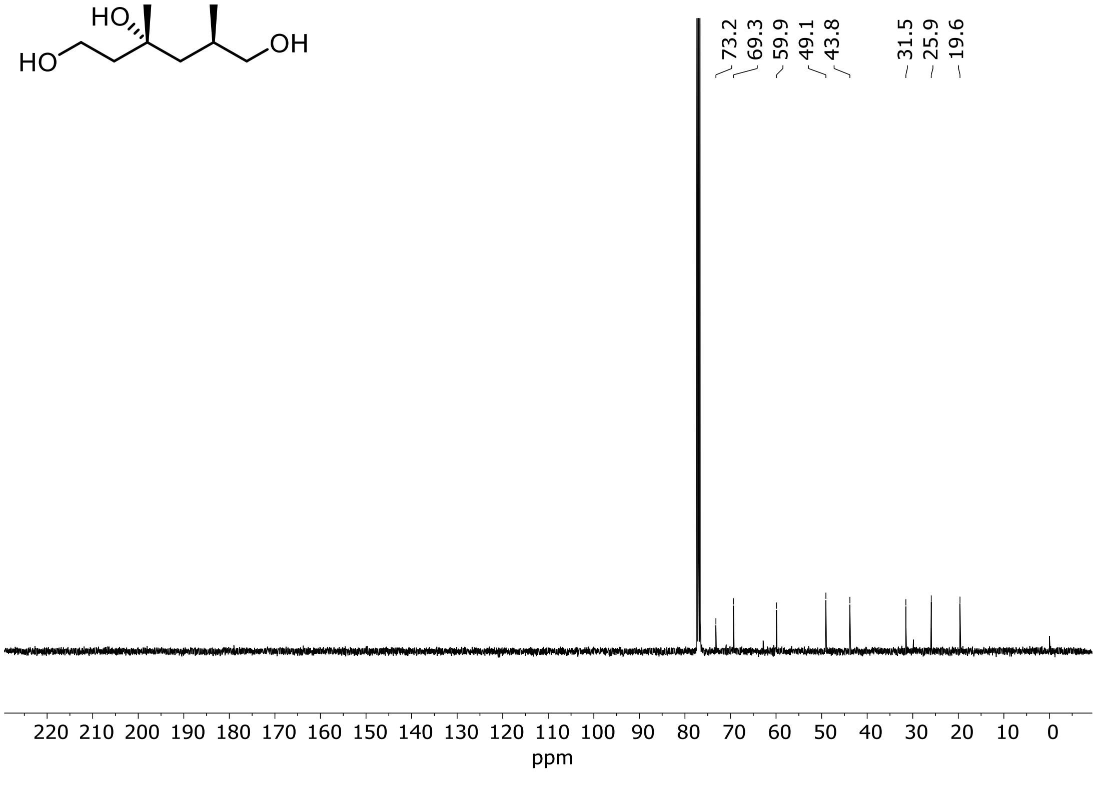

^{1}H-NMR of **16e** in CD_2Cl_2

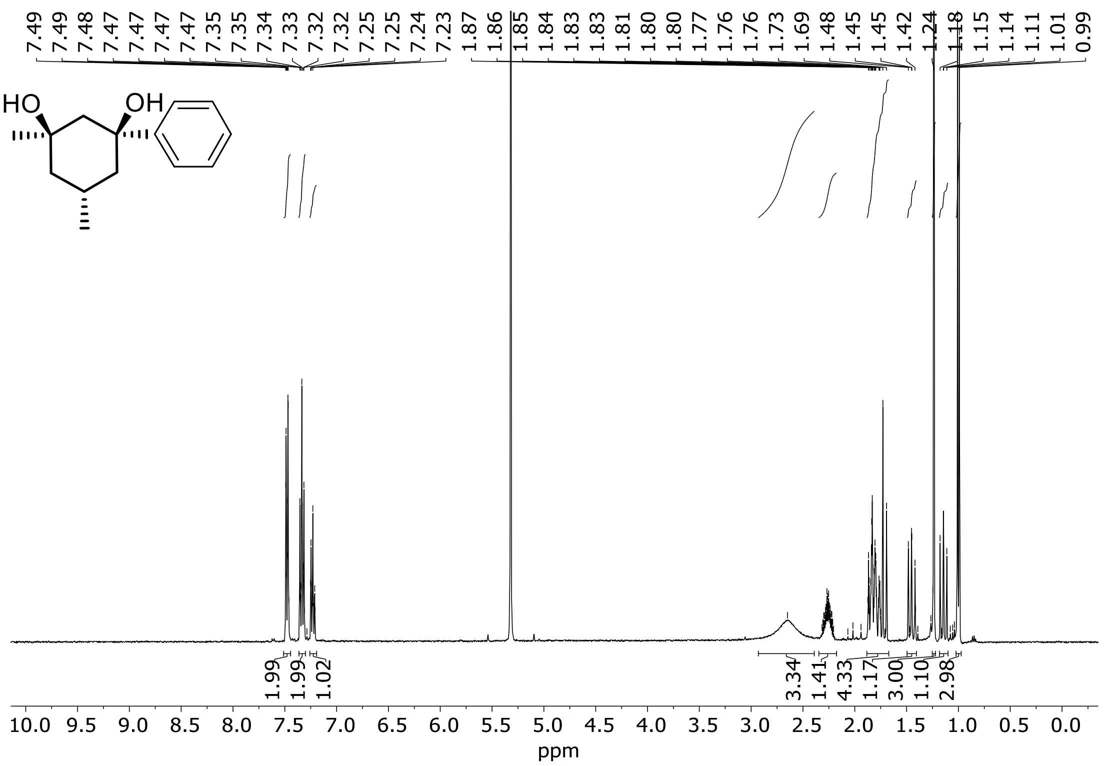

^{13}C-NMR of **16e** in CD_2Cl_2

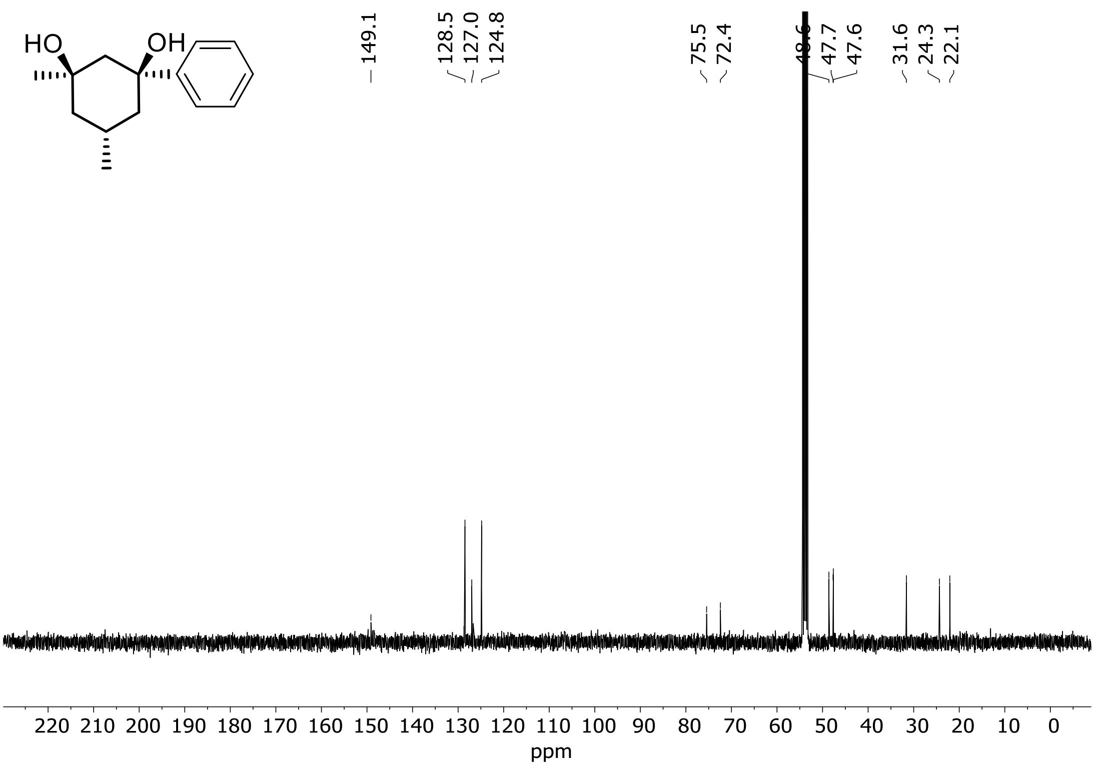

^{1}H-NMR of **15b^D** in $CDCl_3$

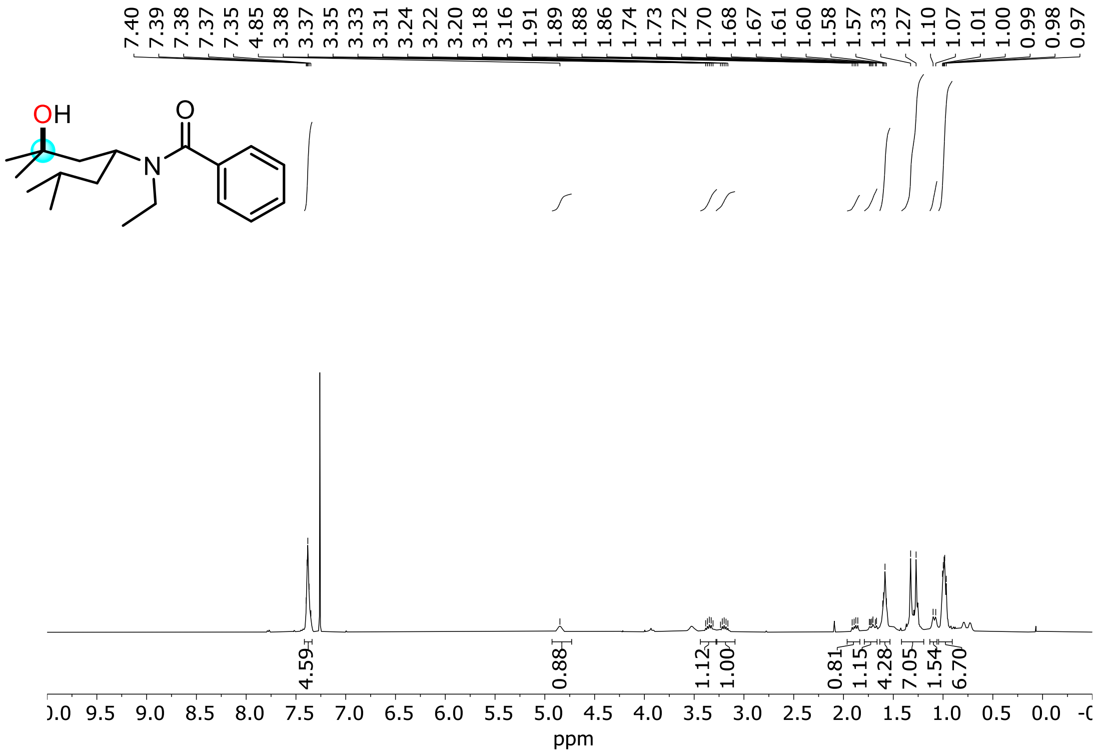

^{13}C-NMR of **15b^D** in $CDCl_3$

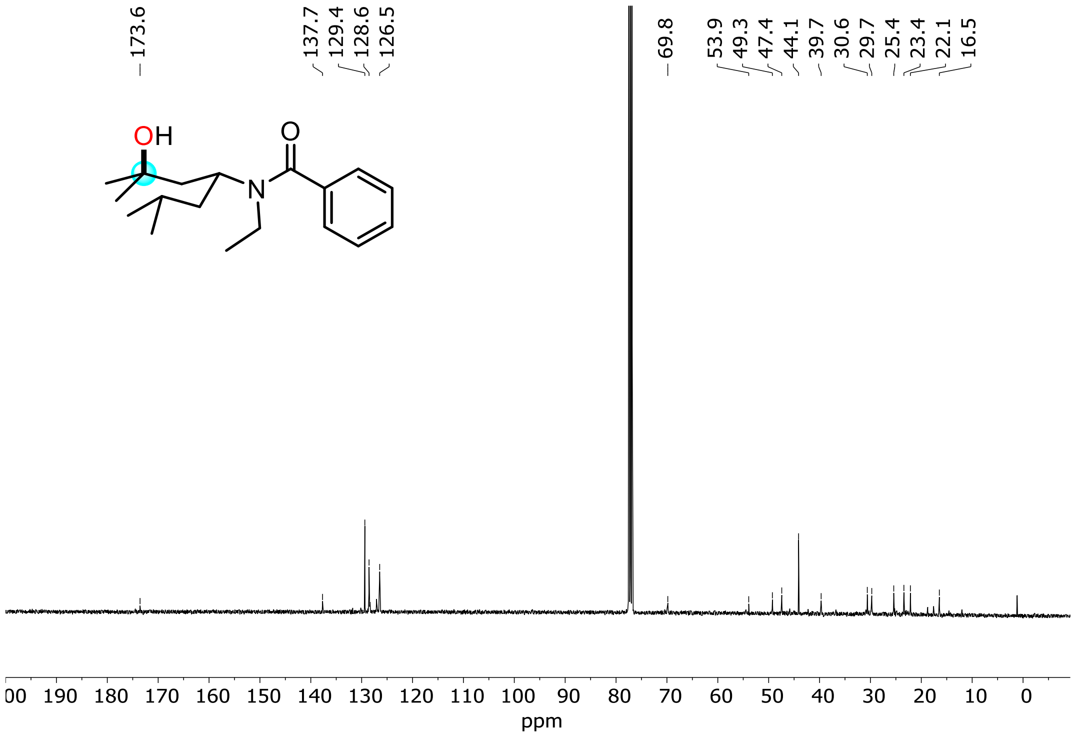

^{1}H-NMR of **19b^{D}** in CDCl3 (*mixture of two rotamers*)

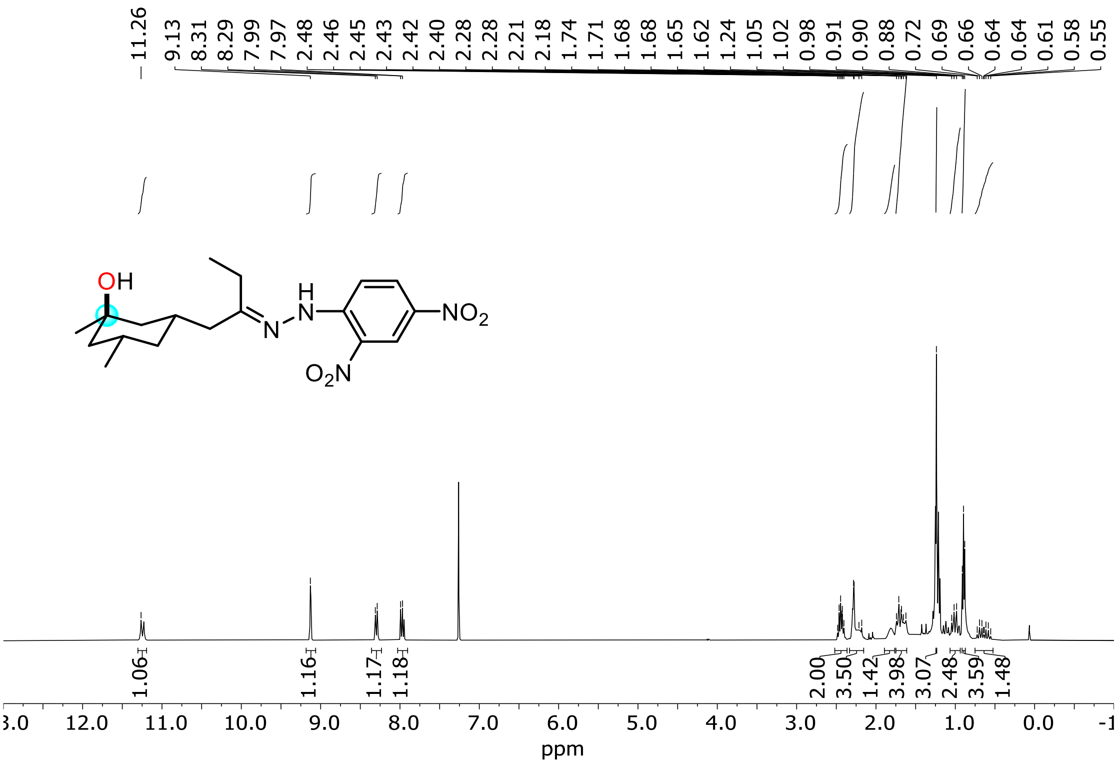

^{13}C-NMR of **19b^{D}** in CDCl3 (*mixture of two rotamers*)

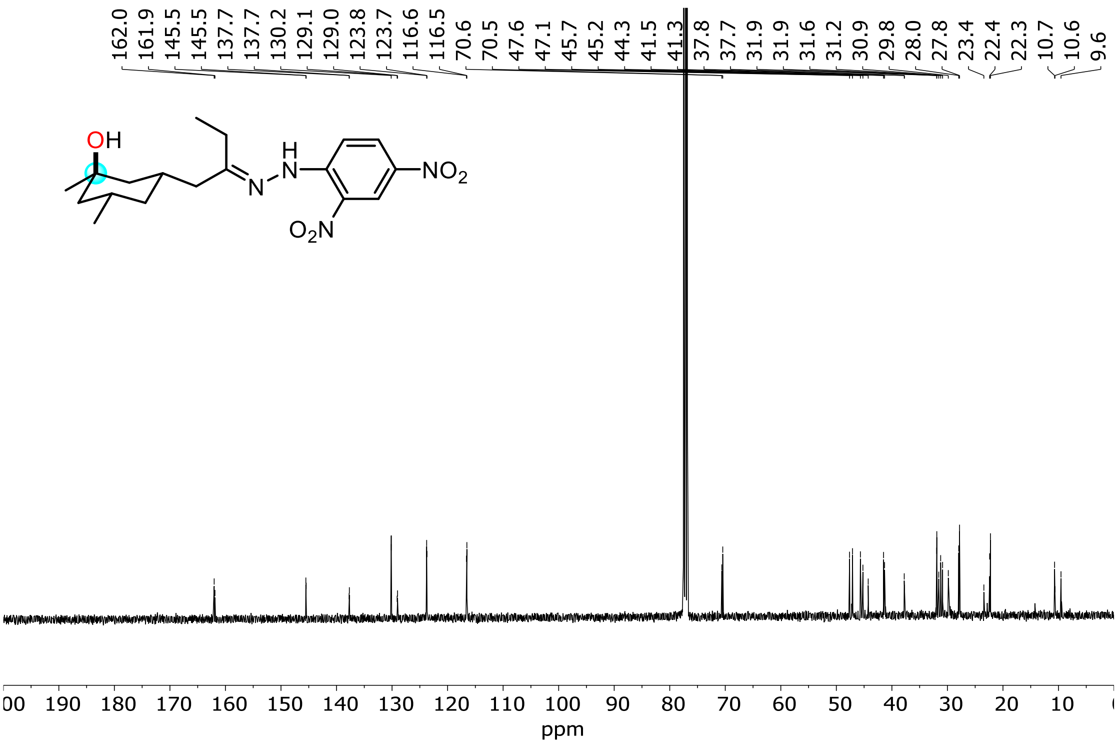

^{1}H-NMR of **21b[D]** in $CDCl_3$

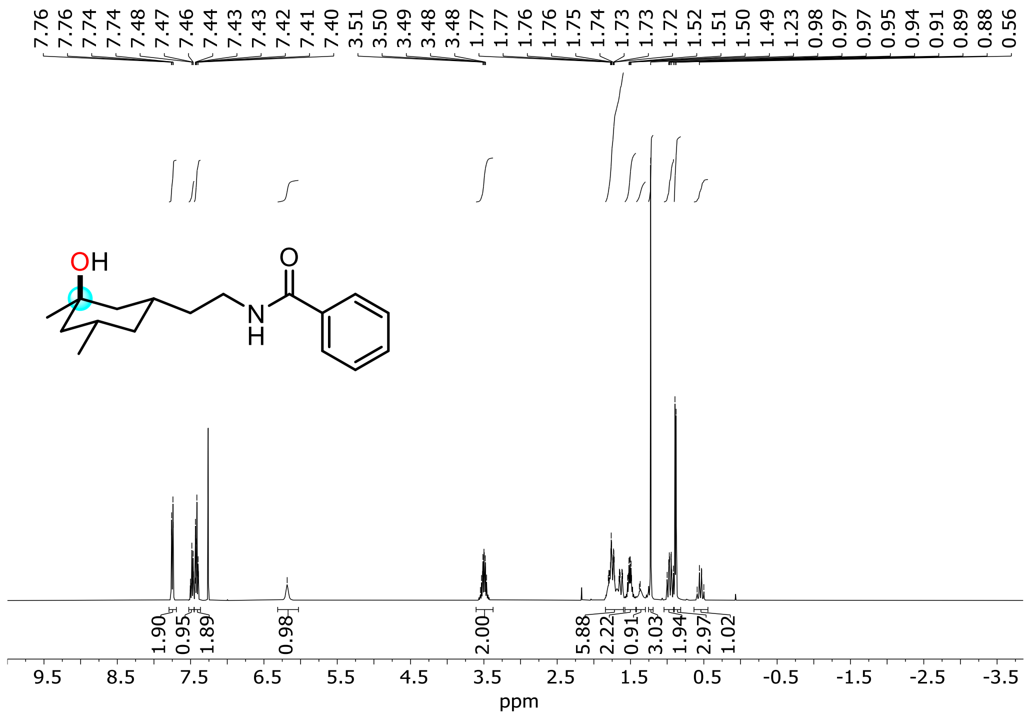

^{13}C-NMR of **21b[D]** in $CDCl_3$

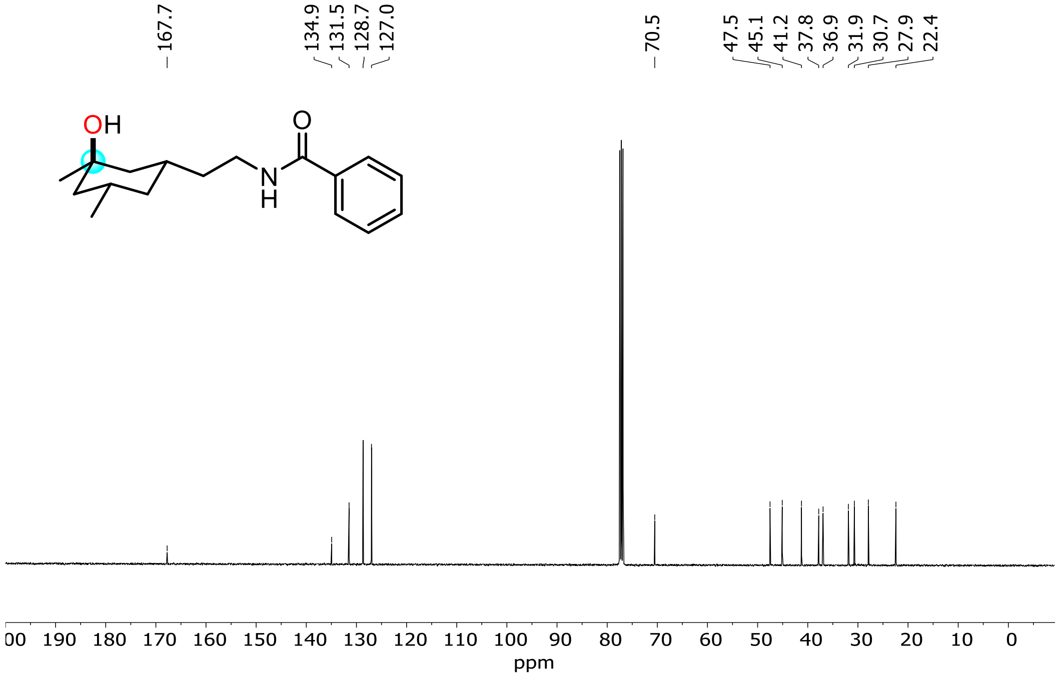

1.8 SFC and GC traces

The racemic products were obtained following oxidation protocol A, with 17 eq. of acetic acid (AcOH), *rac*-Mn complex and 1 eq of H_2O_2. Alcohol products **15b**, **19b** and **21b** were functionalized for chiral SFC analysis.

Derivatization of product 15b

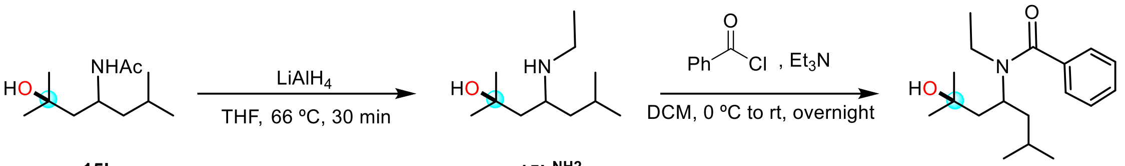

A round-bottom flask equipped with a septum and kept under nitrogen was charged with **15b** (20 mg, 0.1 mmol, 1 equiv.) and 5 mL of dry THF. After that, lithium aluminium hydride (11 mg, 0.3 mmol, 3 equiv) was slowly added at 0 ºC. The resulting mixture was stirred at the same temperature for 10 min, before heating it at reflux temperature for 30 min. After that, the solution was quenched with the minimum quantity of 2 M NaOH solution at 5°C. After filtration through Celite©, the organic fractions were dried over anhydrous Na_2CO_3, filtered and evaporated to dryness to obtain **15b^{NH2}**. The amine **15b^{NH2}** was identified by ESI-MS (188.1 $[M+H]^+$) and immediately used without further purification.

To a freshly prepared solution of **15b^{NH2}** in 2 mL of CH_2Cl_2 was added triethylamine (1.0 equiv.) and then the reaction mixture was cooled to 0 °C. Then, benzoyl chloride (1.0 equiv) was added dropwise over 10 minutes and the reaction was left stirring overnight, letting to warm up to room temperature. After that, saturated aqueous Na_2CO_3 solution was added until pH~10-11 and the aqueous layer was extracted with dichloromethane (2x). The combined organic layers were then washed with 1N HCl and dried over anhydrous sodium sulfate (Na_2SO_4). The organic solvent was evaporated to dryness and the crude amide was purified by flash chromatography over silica gel using hexane : ethyl acetate (7:3) to obtain **15b^D** (13 mg, 69% yield, over 2 steps) as a colorless oil. ^1H NMR (400 MHz, $CDCl_3$) δ 7.40 – 7.35 (m, 5H), 4.85 (br, 1H), 3.38 – 3.31 (m, 1H), 3.24 – 3.16 (m, 1H), 1.91 – 1.86 (m, 1H), 1.74 – 1.67 (m, 1H), 1.61 – 1.57 (m, 4H), 1.37 – 1.25 (m, 7H), 1.10 – 1.07 (m, 1H), 1.01 – 0.97 (m, 7H). ^{13}C NMR (101 MHz, $CDCl_3$) δ 173.6, 137.7, 129.4, 128.6, 126.5, 69.8, 53.9, 49.3, 47.4, 44.1, 39.7, 30.6, 29.7, 25.4, 23.4, 22.1, 16.5. ESI-MS *m/z* found for $C_{18}H_{29}NO_2$ $[M+H]^+$ 292.2, $[M+Na]^+$ 314.2.

Derivatization of product 19b

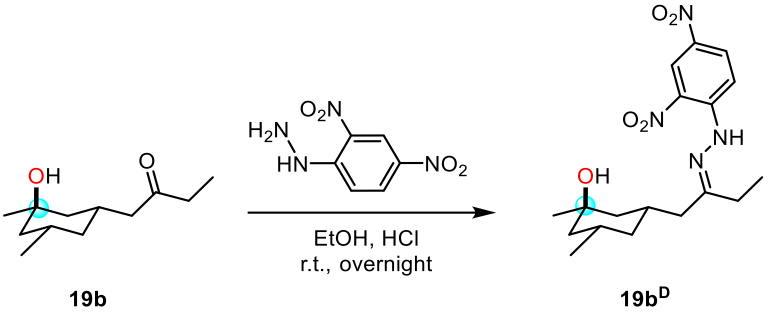

To a solution of alcohol **19b** (9 mg, 0.045 mmol) in absolute ethanol (2 mL) and HCl (37%, 5 uL), was added 2,4-dinitrophenylhydrazine (8.9 mg, 0.045 mmol). The mixture was stirred overnight at room temperature. After that, the organic solvent was evaporated to dryness and the crude solid was purified by flash chromatography over silica gel using hexane : ethyl acetate (7:3) to obtain **19b^D^** (12.5 mg, 73% yield) as a yellow solid. The reaction gave the product **19b^D^** as a mixture of two rotamers in a 1.2:1 ratio. *^{13}H NMR of the major rotamer:* ^{1}H NMR (400 MHz, $CDCl_3$) δ 11.26 (s, 1H), 9.13 (s, 1H), 8.30 (d, *J* = 9.6 Hz, 1H), 7.98 (d, *J* = 9.6 Hz, 1H), 2.44 (q, *J* = 7.3 Hz, 2H), 2.33 – 2.17 (m, 3H), 1.87-1.76 (m, 1H), 1.74 – 1.62 (m, 3H), 1.21 (s, 3H), 1.04-0.98 (m, 3H), 1.01 – 0.94 (m, 1H), 0.91 – 0.88 (m, 3H), 0.72-0.55 (m, 1H). *^{13}C NMR of two rotamers*: ^{13}C NMR (101 MHz, $CDCl_3$) δ 162.0, 161.9, 145.5, 145.5, 137.7, 137.7, 130.2, 129.1, 129.0, 123.8, 123.7, 116.6, 116.5, 70.6, 70.5, 47.6, 47.1, 45.7, 45.2, 44.3, 41.5, 41.3, 37.8, 37.7, 31.9, 31.9, 31.6, 31.2, 30.9, 29.8, 28.0, 27.8, 23.4, 22.4, 22.3. ESI-MS *m/z* found for $C_{18}H_{26}N_4O_5$ $[M+H]^+$ 292.2, $[M+Na]^+$ 314.2.

Derivatization of product 21b

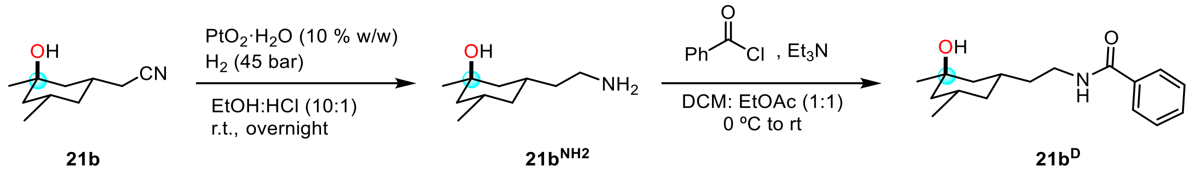

A vial was charged with the alcohol **21b** (20 mg, 0.12 mmol) and $PtO_2 \cdot H_2O$ catalyst (10% w/w), and it was sealed with a septum and kept under nitrogen. Then, absolute ethanol (1.7 mL) and HCl (37%, 0.3 mL) were added under nitrogen flow. At this point, the vial was placed in a Parr reactor and the system was purged with hydrogen flow under stirring. After 5 minutes, the hydrogen pressure was set at 50 psi and left stirring overnight at 25 ºC. After carefully depressurization, the reaction mixture was filtered over acrodisc®, and the resulting solution was evaporated under reduced pressure. The resulting oil was dissolved in CH_2Cl_2 and the organic phase was washed with $NaHCO_3$ solution (x3). After that, the organic solvent was dried over $MgSO_4$ anhydrous and removed under reduced pressure. The amine **21b^{NH2}** was identified by ESI-MS (154.1 $[M+H]^+$) and immediately used without further purification.

To a freshly prepared solution of **21b^{NH2}** in 2 mL of CH_2Cl_2:EtOAc (1:1) was added triethylamine (1.0 equiv.) and then the reaction mixture was cooled to 0 °C. Then, benzoyl chloride (1.0 equiv) was added dropwise over 10 minutes and the reaction was left stirring overnight, letting to warm up to room temperature. After that, saturated aqueous Na_2CO_3 solution was added until pH~10-11 and the aqueous layer was extracted with dichloromethane (2x). The combined organic layers were then washed with 1N HCl and dried over anhydrous sodium sulfate (Na_2SO_4). The organic solvent was evaporated to dryness and the crude amide was purified by flash chromatography over silica gel using hexane : ethyl acetate (8:2) to obtain **21b^D** (13 mg, 39% yield, over 2 steps) as a colorless oil. ^1H NMR (400 MHz, $CDCl_3$) δ 7.76 – 7.74 (m, 2H), 7.50 – 7.46 (m, 1H), 7.44 – 7.39 (m, 2H), 6.18 (br, 1H), 3.55 – 3.46 (m, 2H), 1.84 – 1.61 (m, 5H), 1.55 – 1.45 (m, 2H), 1.37 (br, 1H), 1.23 (s, 3H), 1.00 – 0.91 (m, 2H), 0.88 (d, J = 6.4 Hz, 3H), 0.59 – 7.74 (m, 1H). ^{13}C NMR (101 MHz, $CDCl_3$) δ 167.7, 134.9, 131.5, 128.7, 127.0, 70.5, 47.5, 45.1, 41.2, 37.8, 36.9, 31.9, 30.7, 27.9, 22.4. ESI-MS *m/z* found for $C_{17}H_{25}NO_2$ $[M+Na]^+$ 298.1.

***Rac*-1f**

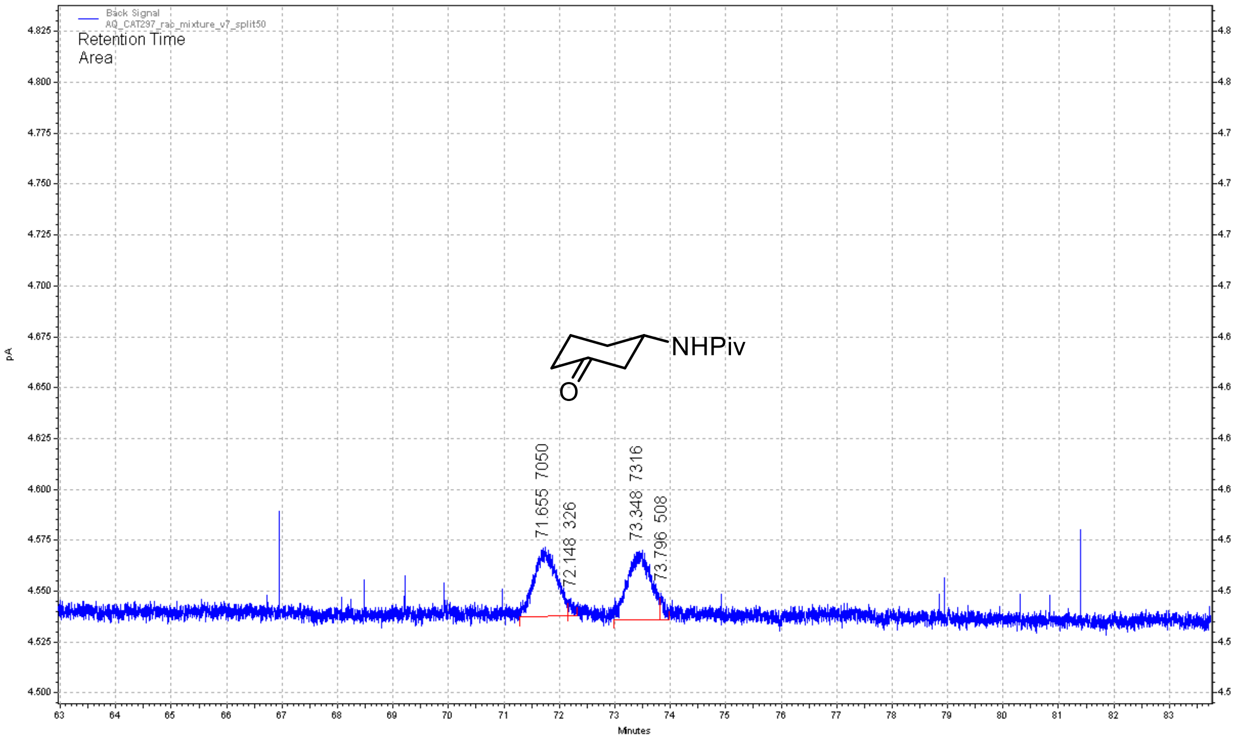

***(S,S)-Mn(^{TIPS}pdp)*-1f**

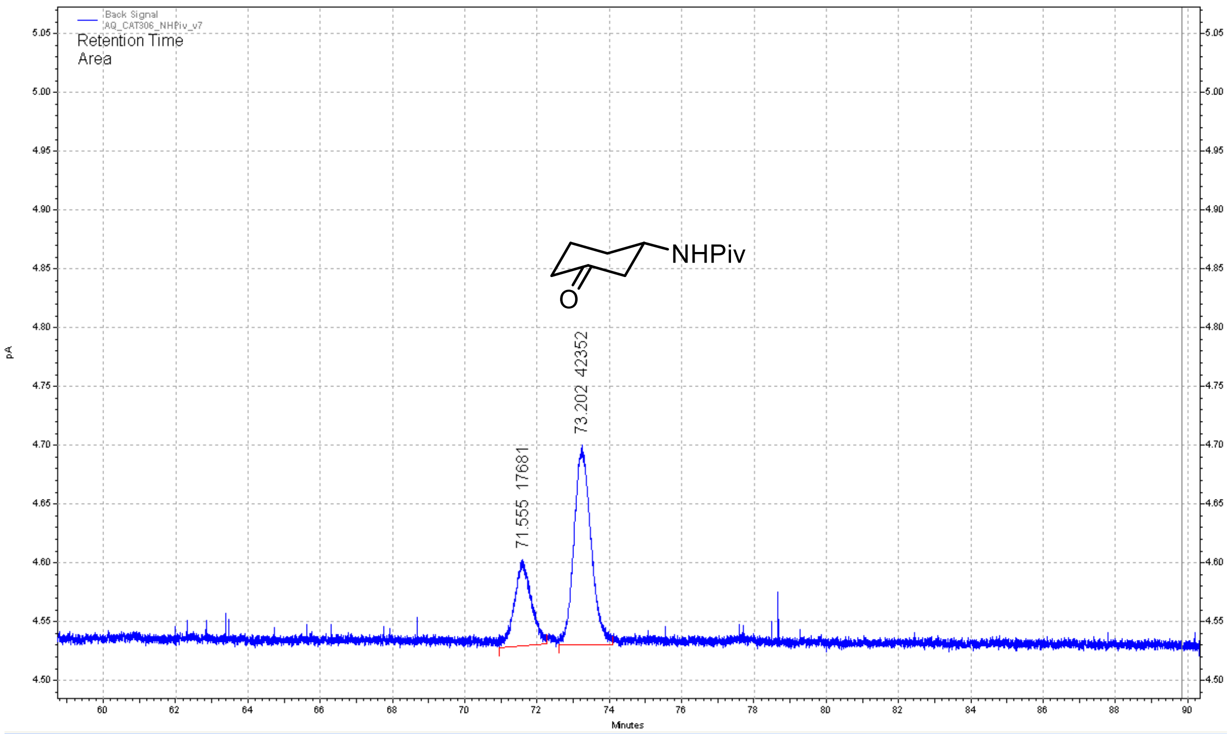

***Rac*-1b**

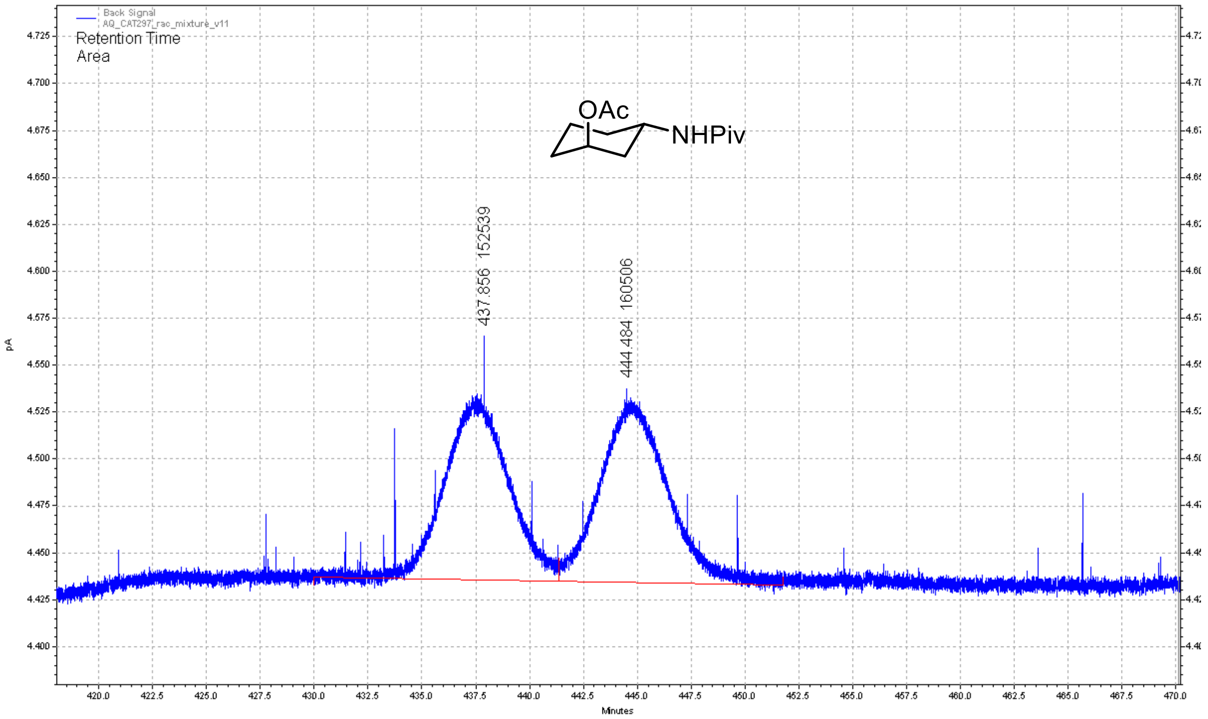

***(S,S)*-*Mn(*^{TIPS}*pdp)*-1b**

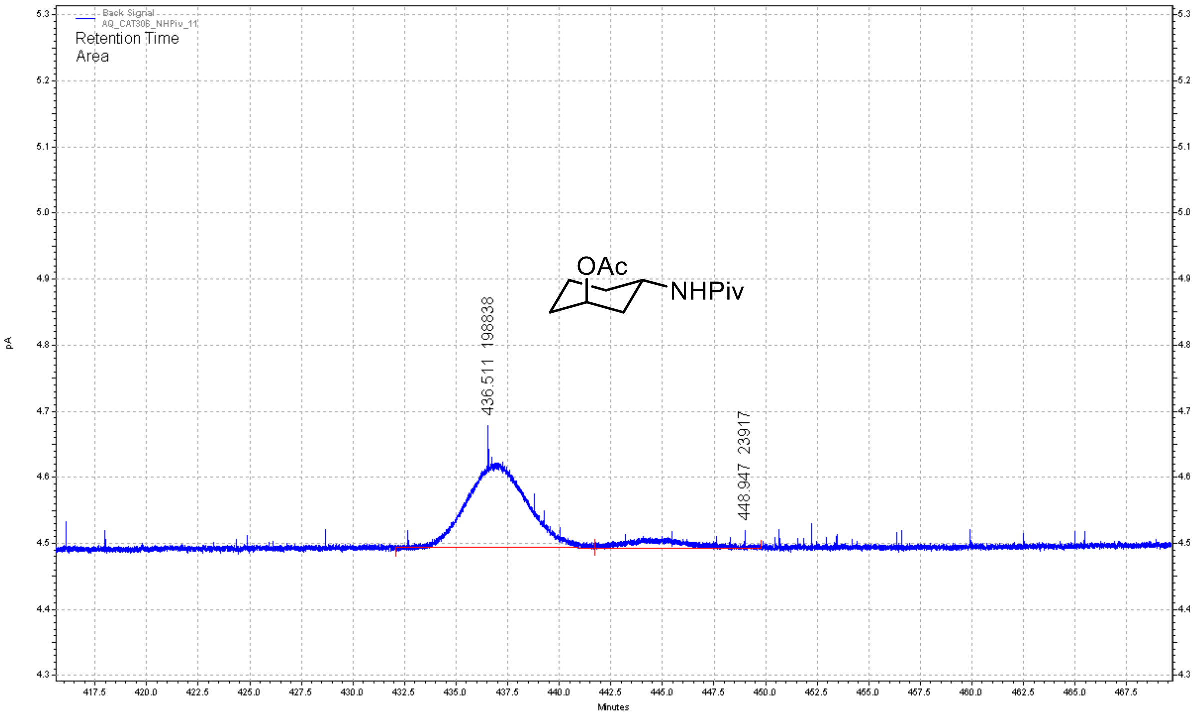

***Rac*-1c**

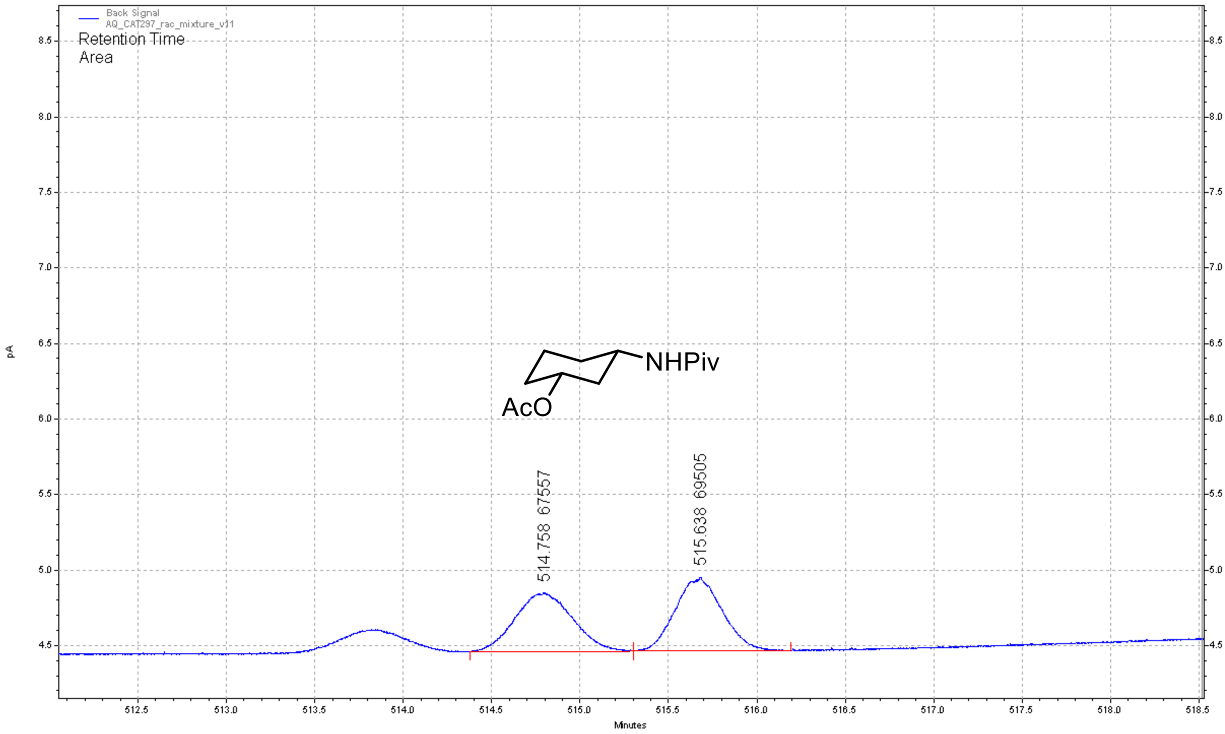

***(S,S)-Mn(^{TIPS}pdp)*-1c**

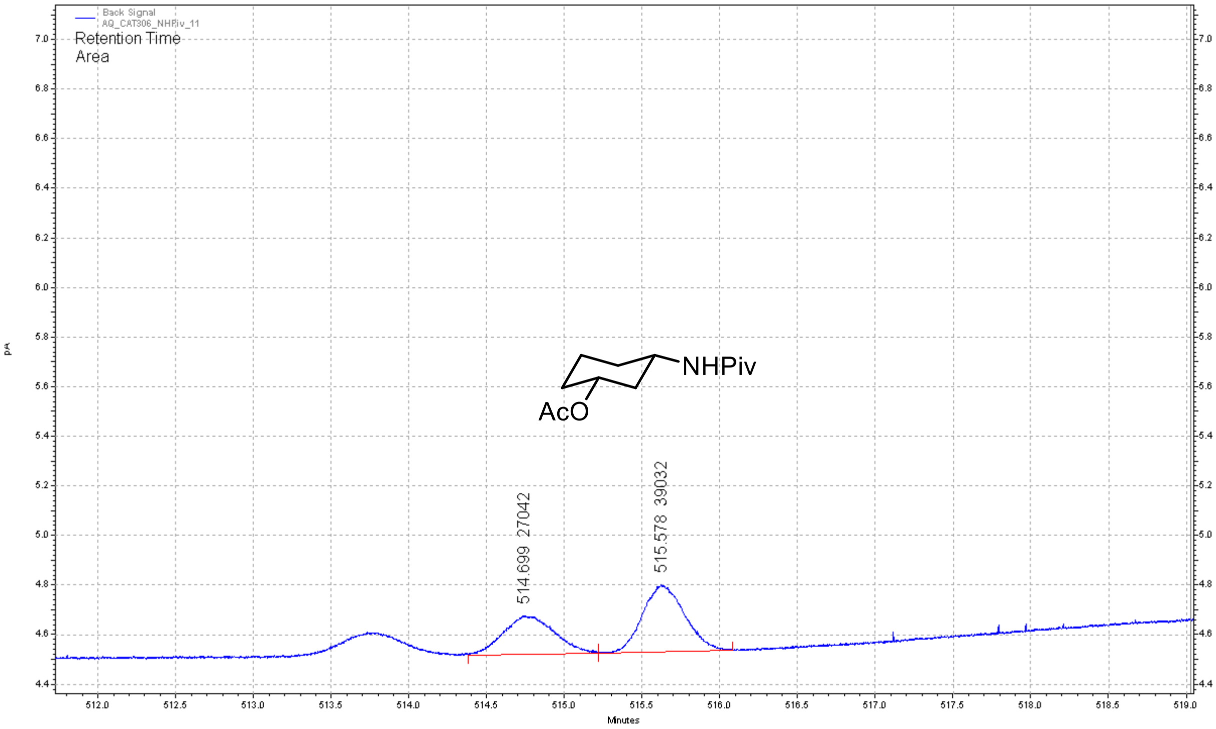

Rac-2b

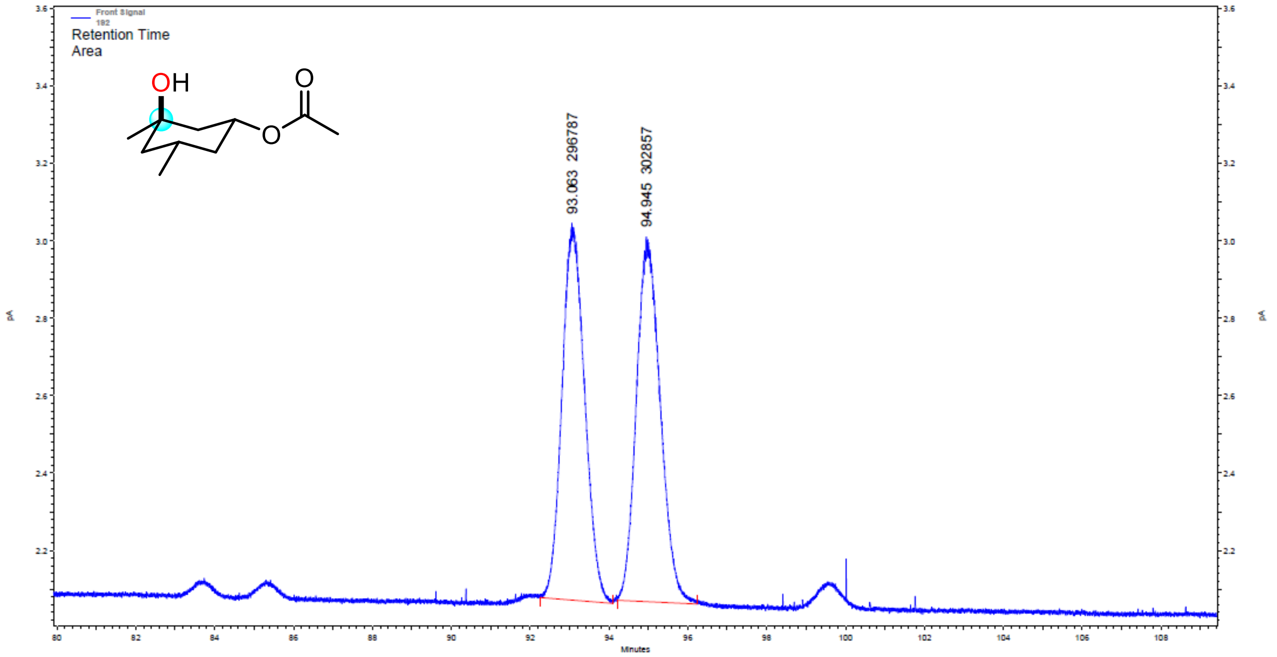

(R,R)-Mn(TIPSpdp)-2b

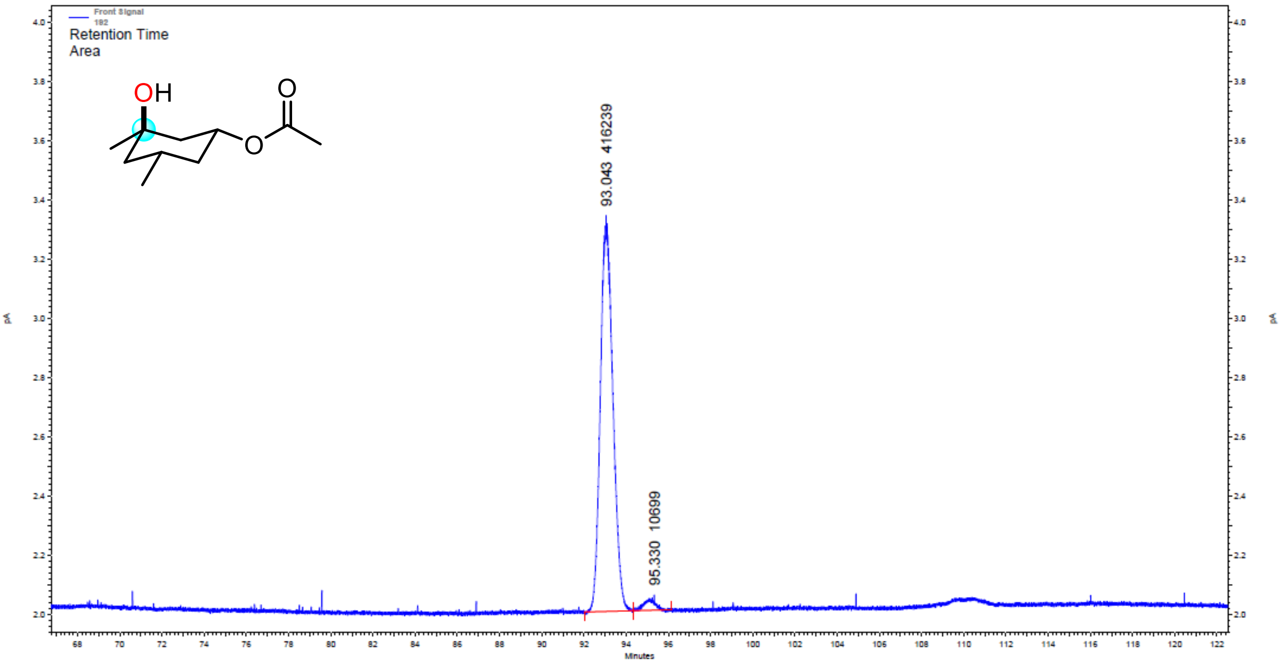

***Rac*-3b**

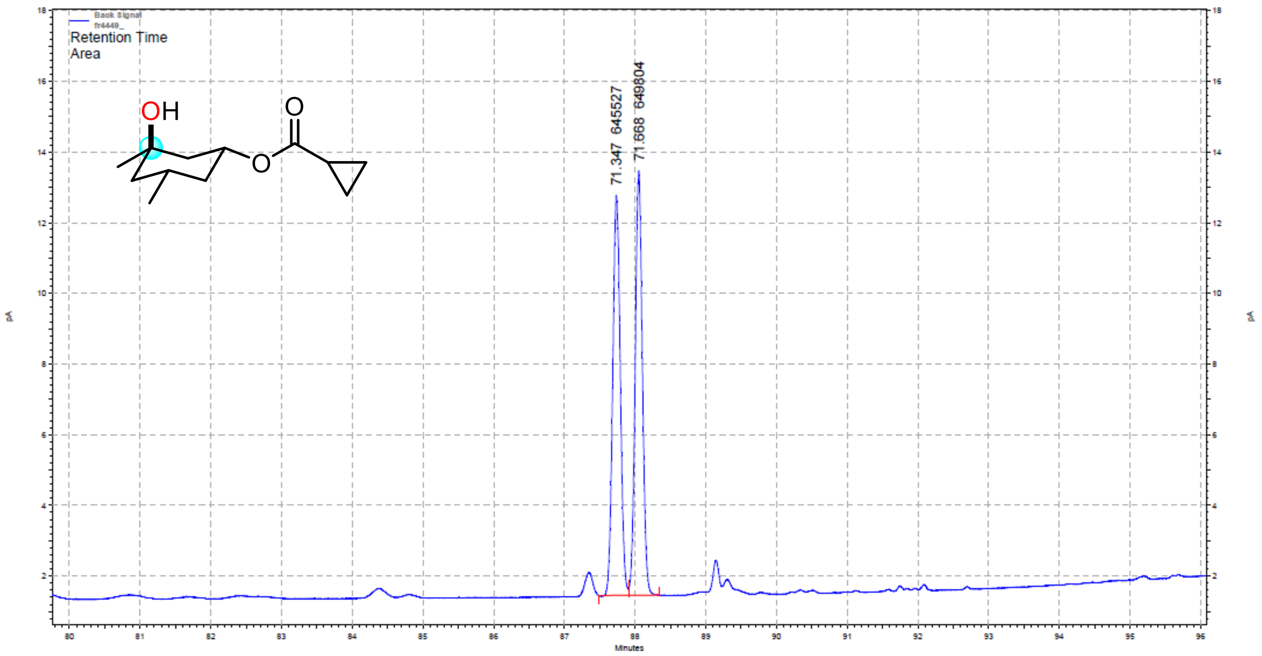

***(R,R)-Mn(TIPSpdp)*-3b**

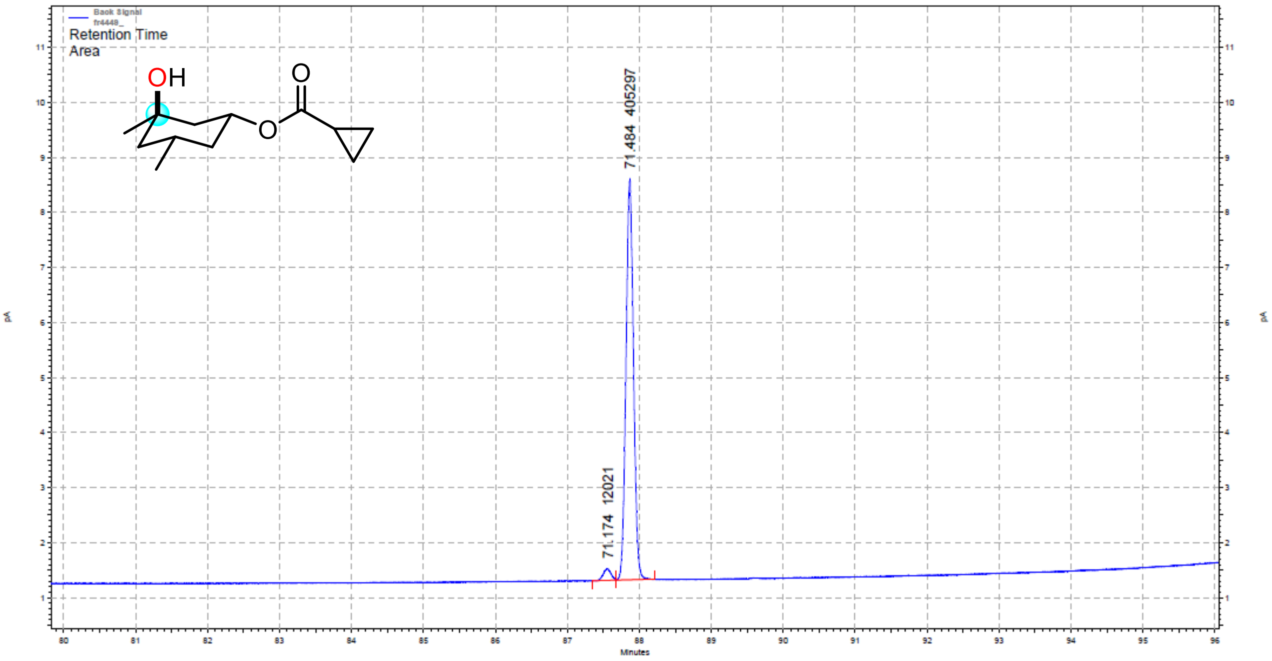

***Rac*-4b**

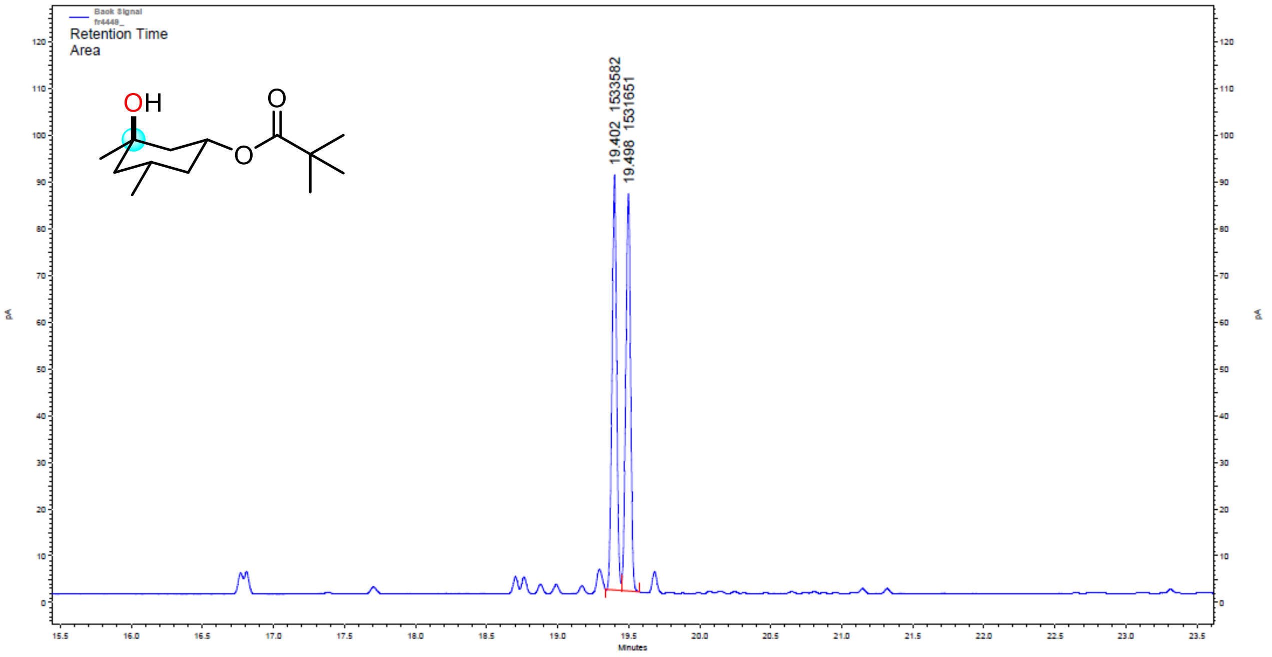

***(R,R)-Mn(TIPSpdp)*-4b**

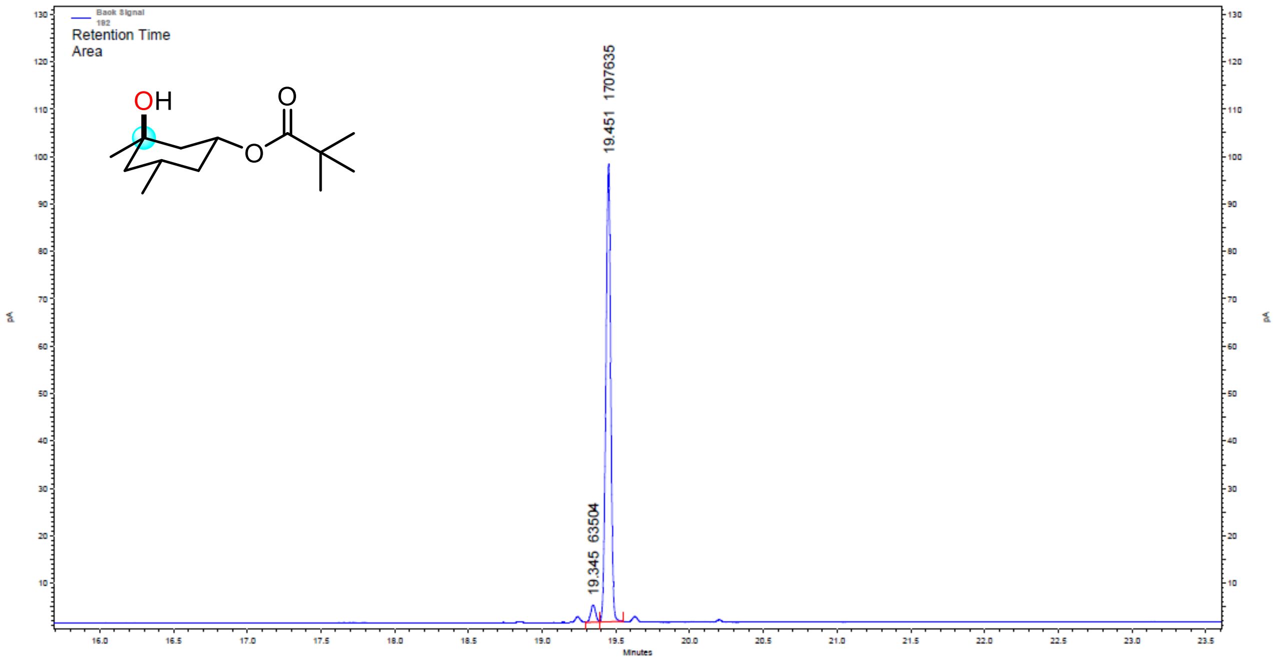

***Rac*-5b**

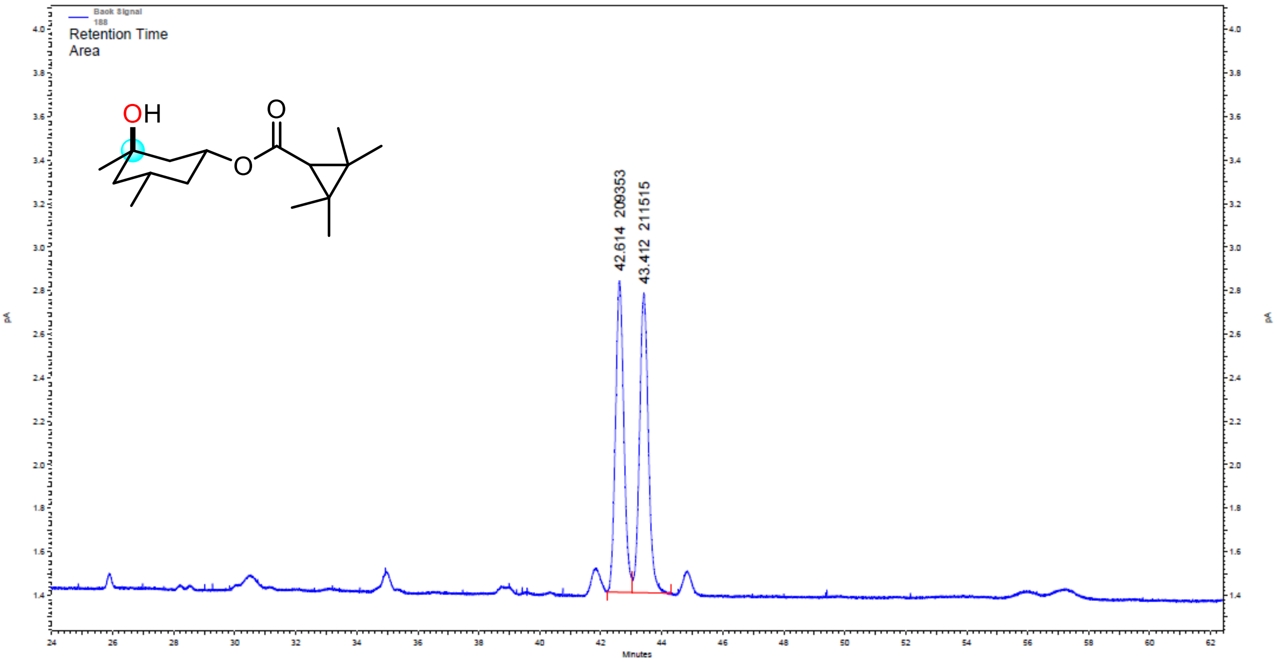

***(R,R)-Mn(TIPSpdp)*-5b**

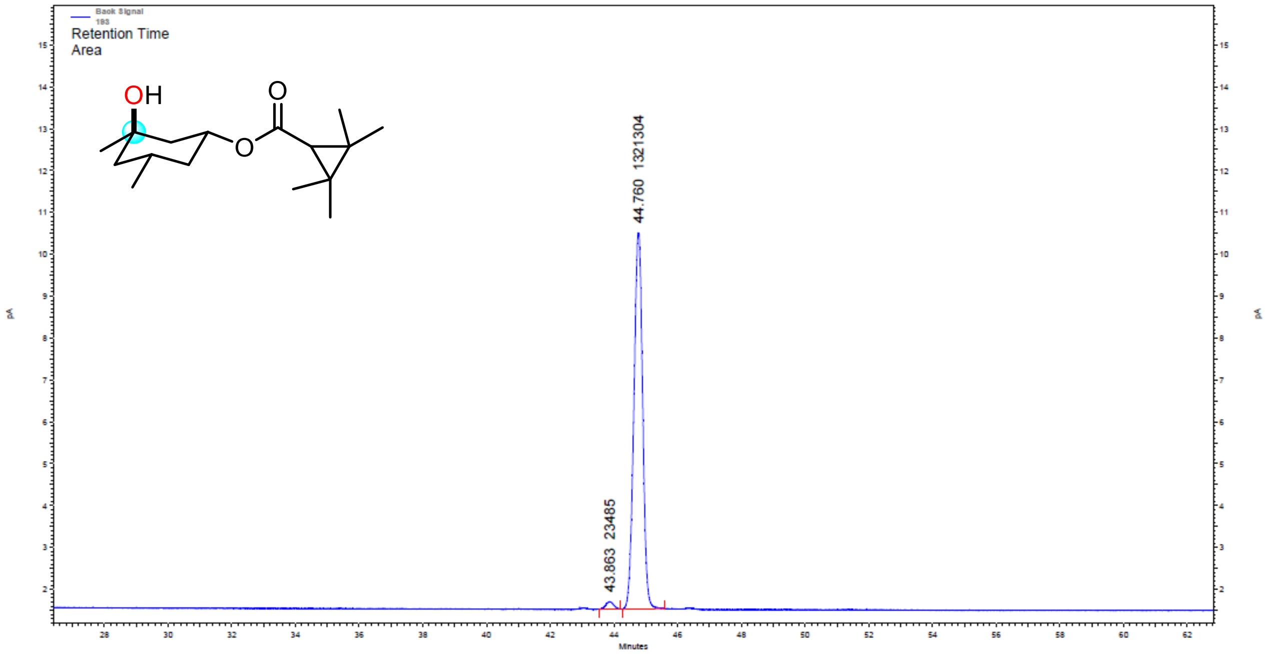

SFC separation conditions: Chiralpack IC-3 (100 × 4.6mm, 3µm), 220.4 nm, CO_2/ IPA= 90:10, 1.5 mL/min; r.t.(minor) = 1.59 min, r.t.(major) = 1.88 min.

***Rac*-6b**

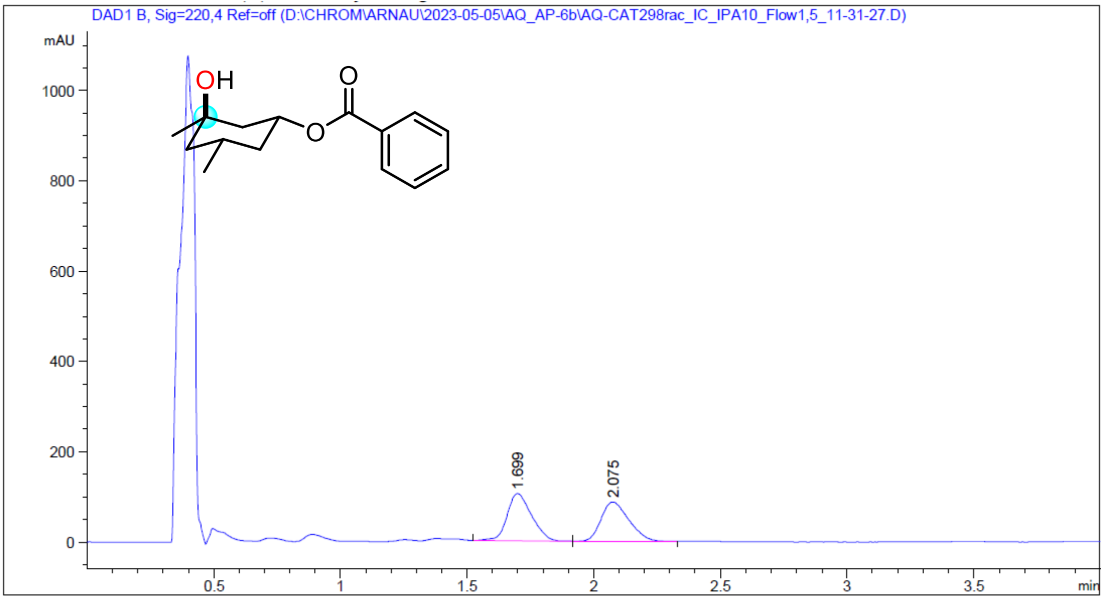

Peak #	RetTime [min]	Type	Width [min]	Area [mAU*s]	Height [mAU]	Area %
1	1.699	BB	0.0992	701.90204	104.77829	51.3338
2	2.075	BV R	0.1167	665.42749	87.07394	48.6662

***(R,R)-Mn(TIPSpdp)*-6b**

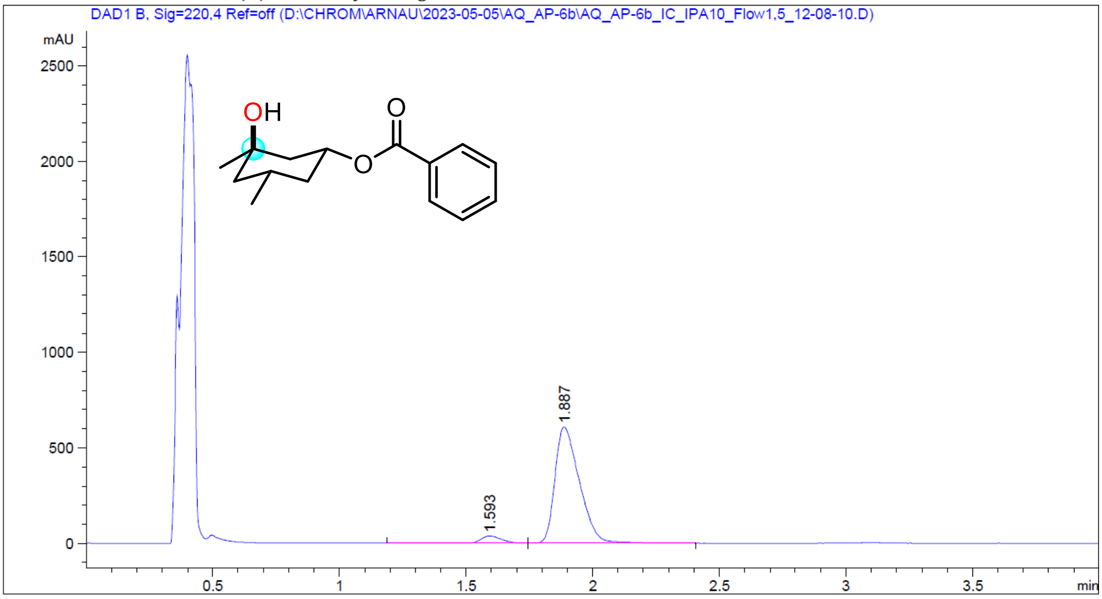

Peak #	RetTime [min]	Type	Width [min]	Area [mAU*s]	Height [mAU]	Area %
1	1.593	VV R	0.0850	222.71700	37.89082	5.1754
2	1.887	VV R	0.1012	4080.62817	609.00482	94.8246

***Rac*-7b**

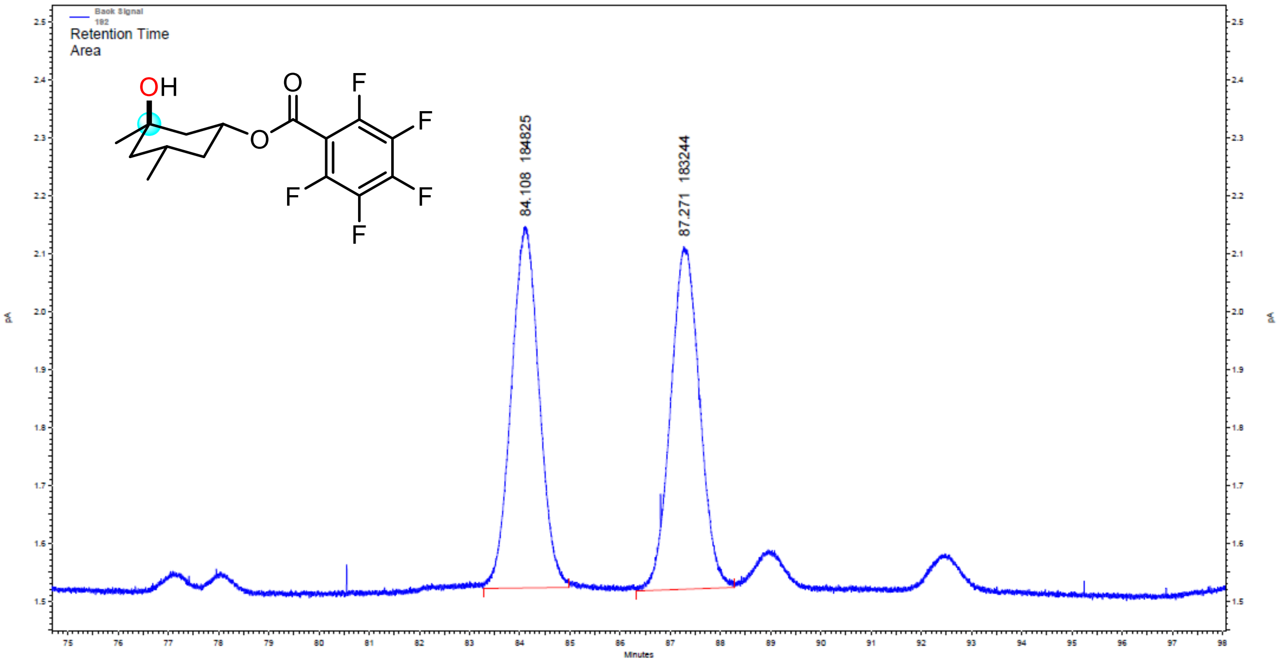

***(R,R)*-*Mn(*^{TIPS}*pdp)*-7b**

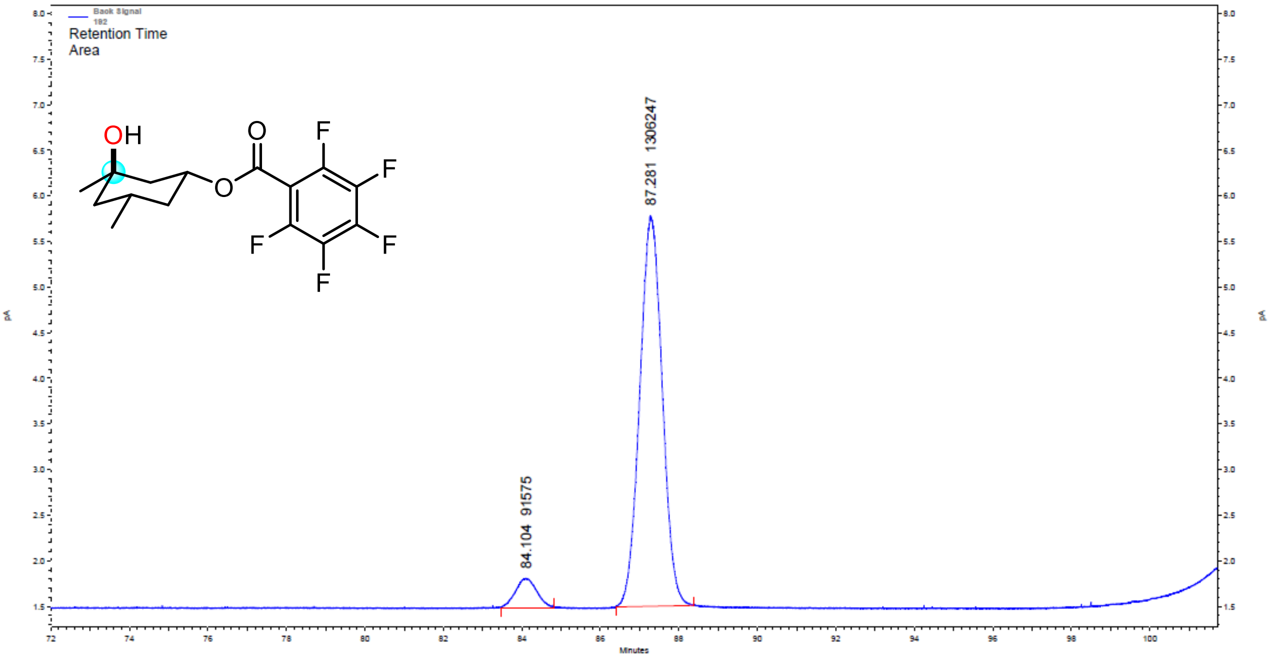

Rac-8b and 8c

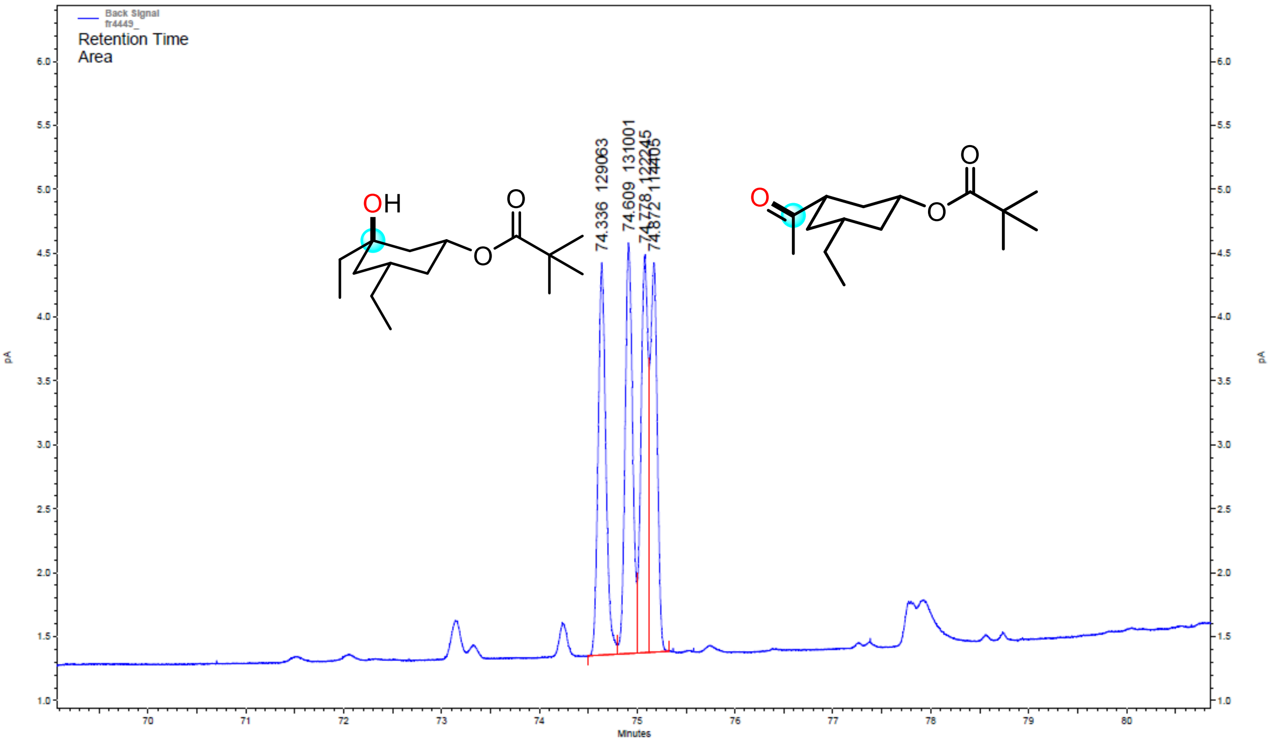

***(R,R)-Mn(TIPSpdp)*-8b and 8c** Method B

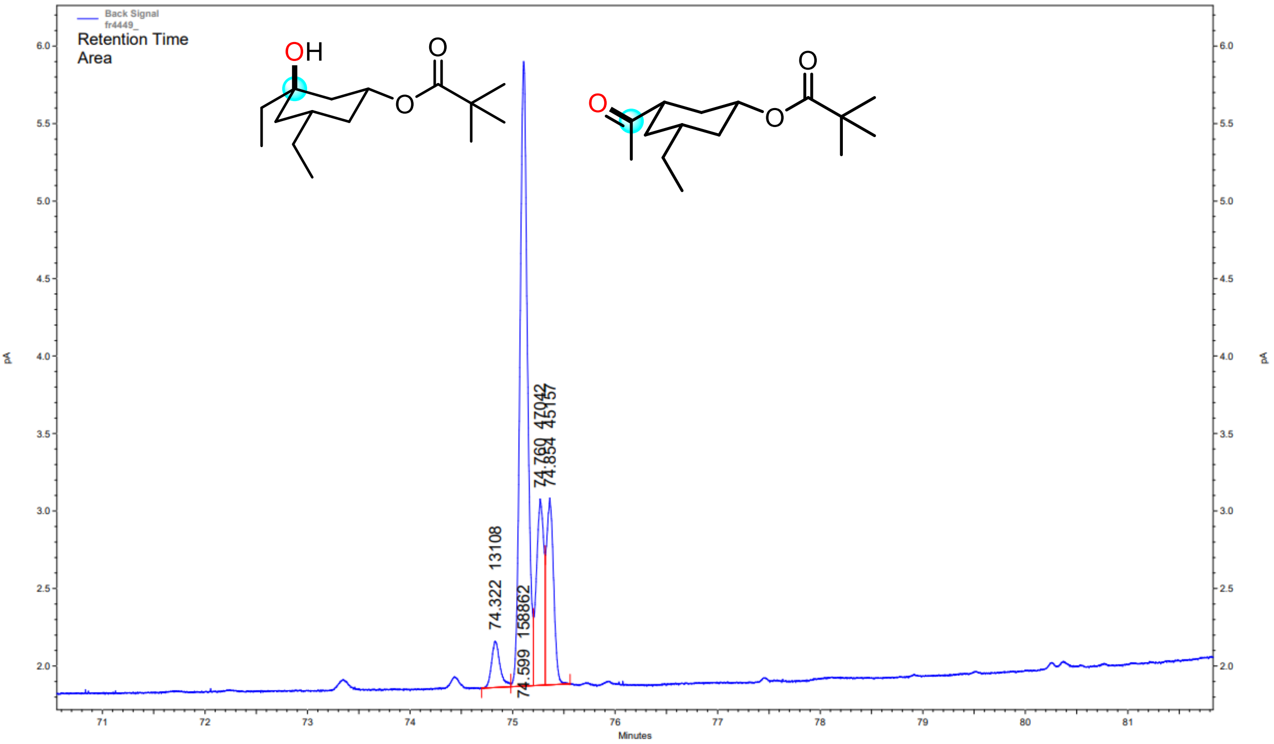

(R,R)-Mn(^{TIPS}***pdp)*-8b** Method A

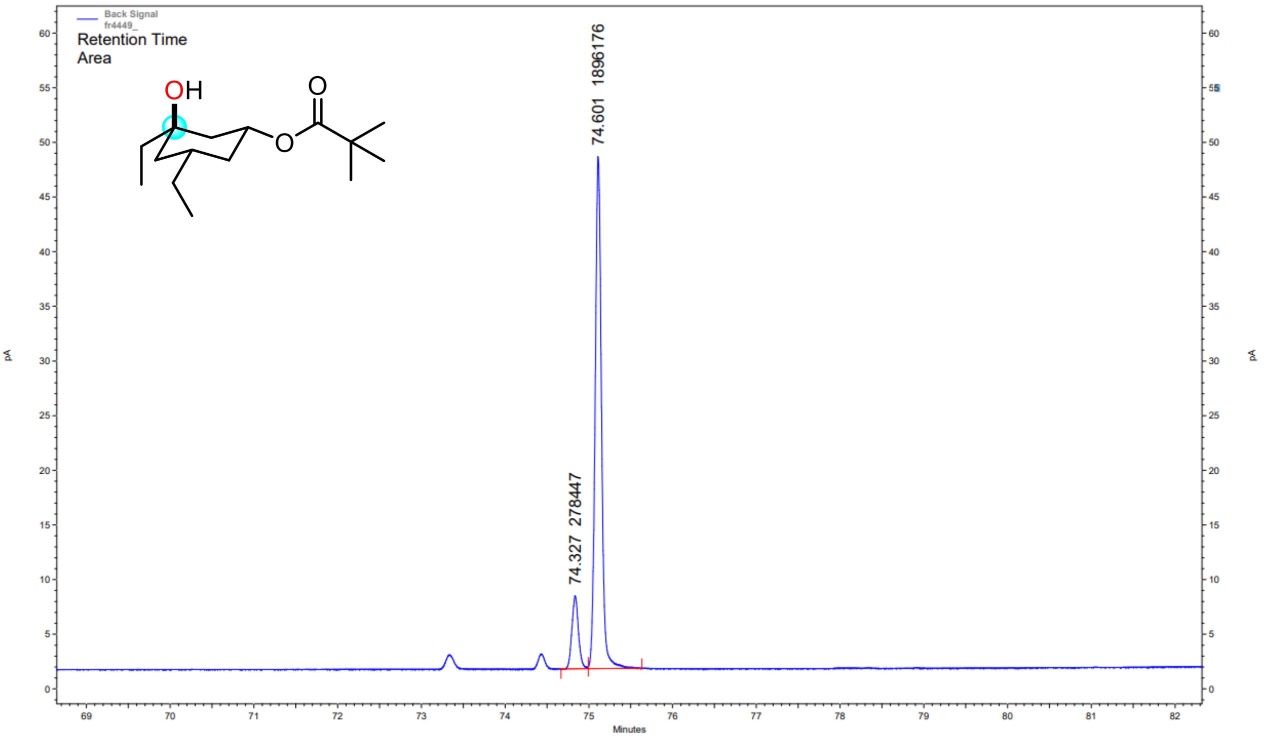

(R,R)-Mn(^{TIPS}***pdp)*-8c** Method A

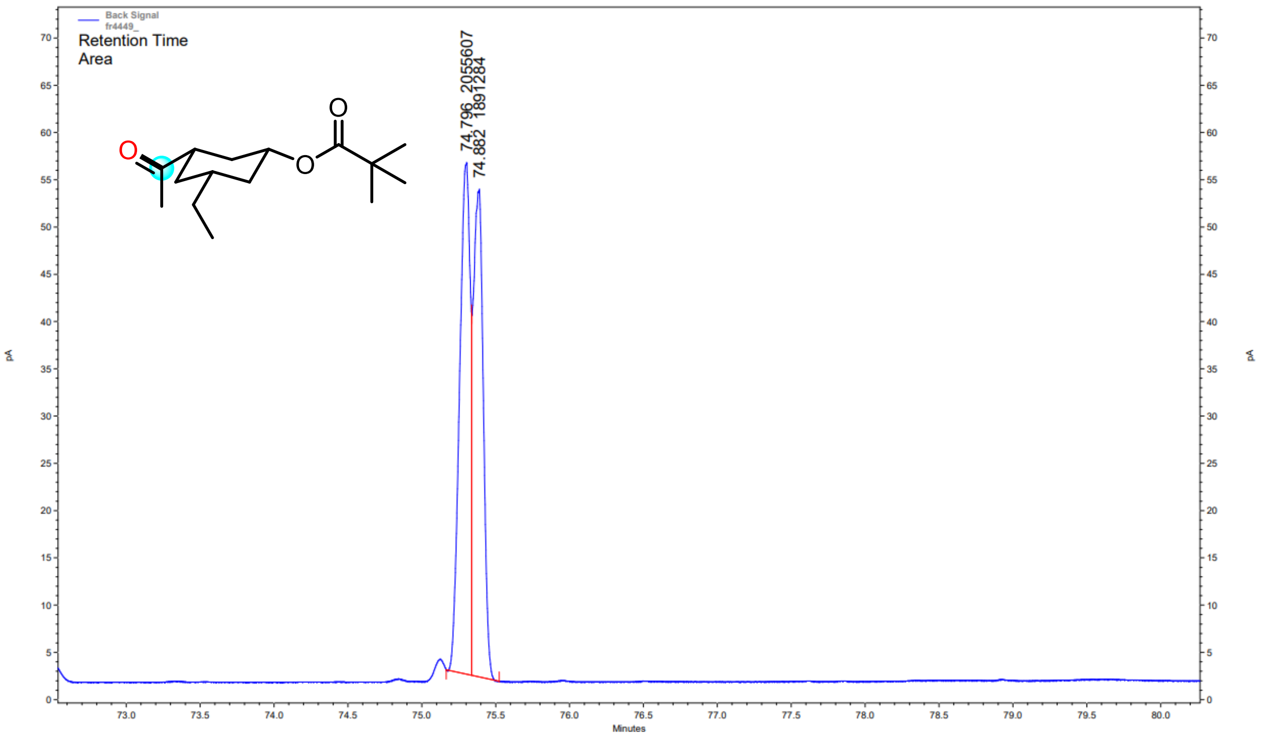

***Rac*-10b**

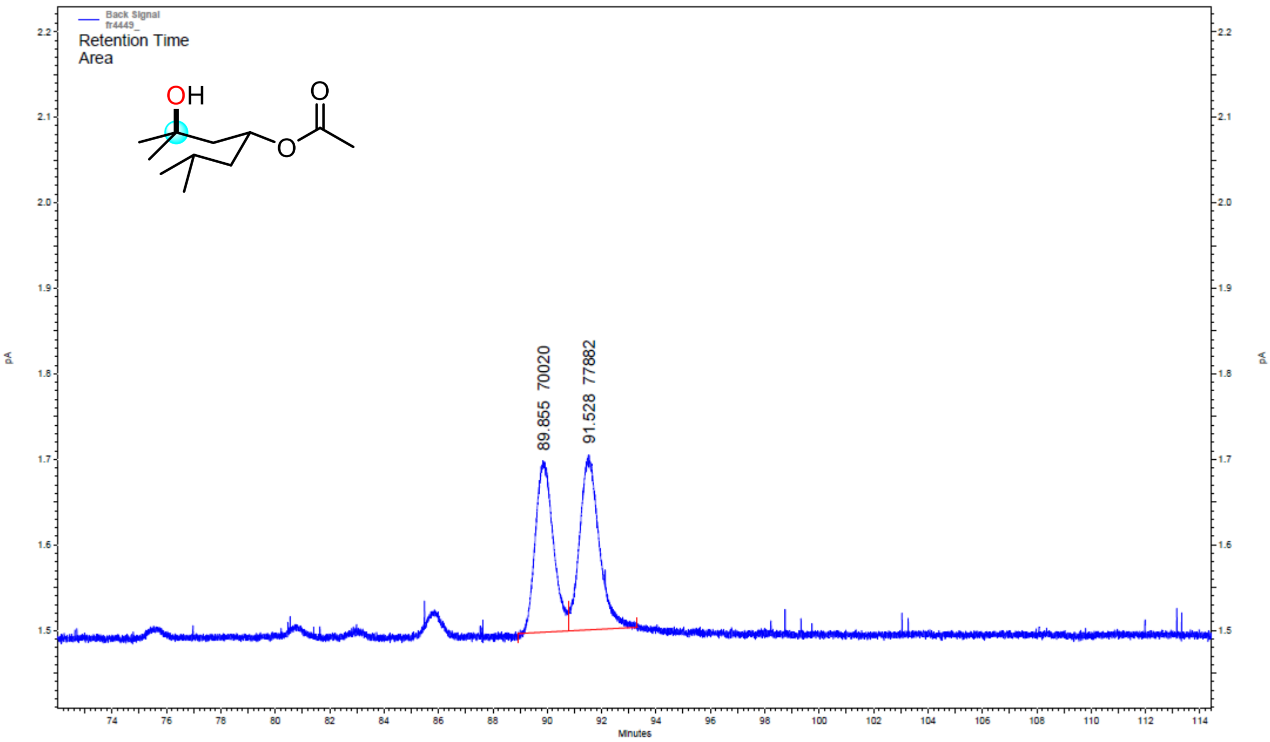

***(R,R)-Mn(*TIPS*pdp)*-10b**

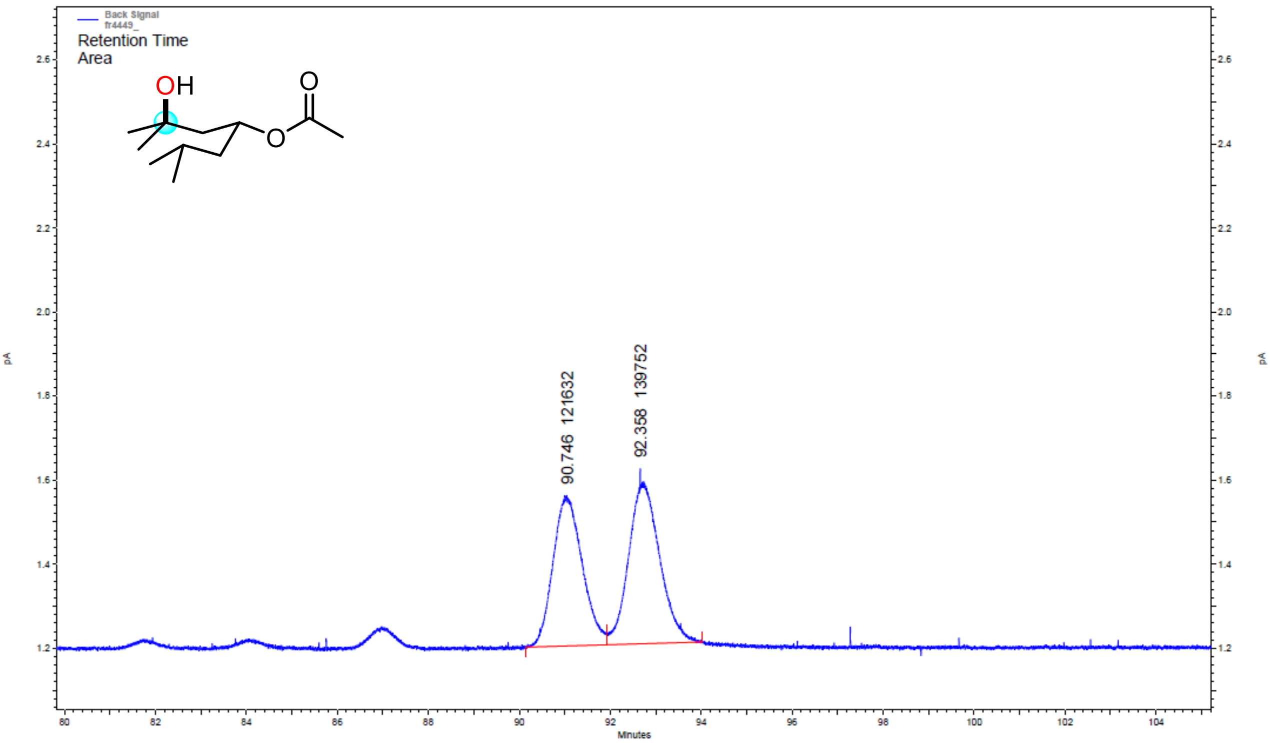

***Rac*-11b**

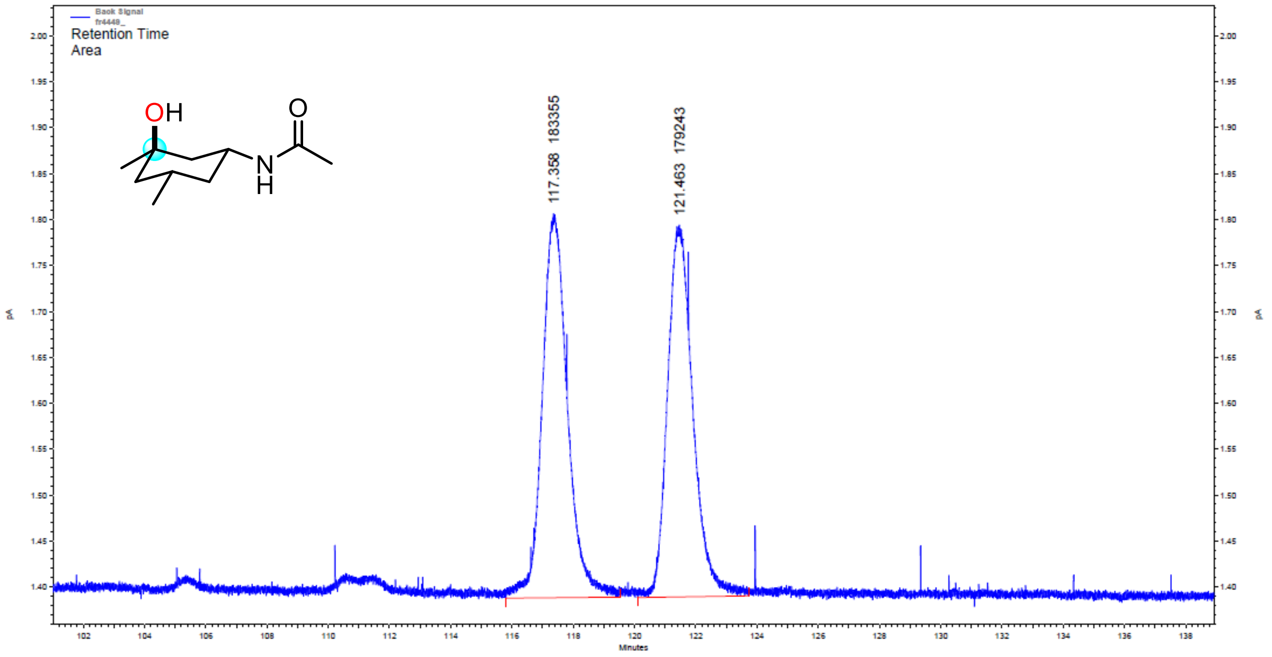

***(R,R)-Mn(TIPSpdp)*-11b**

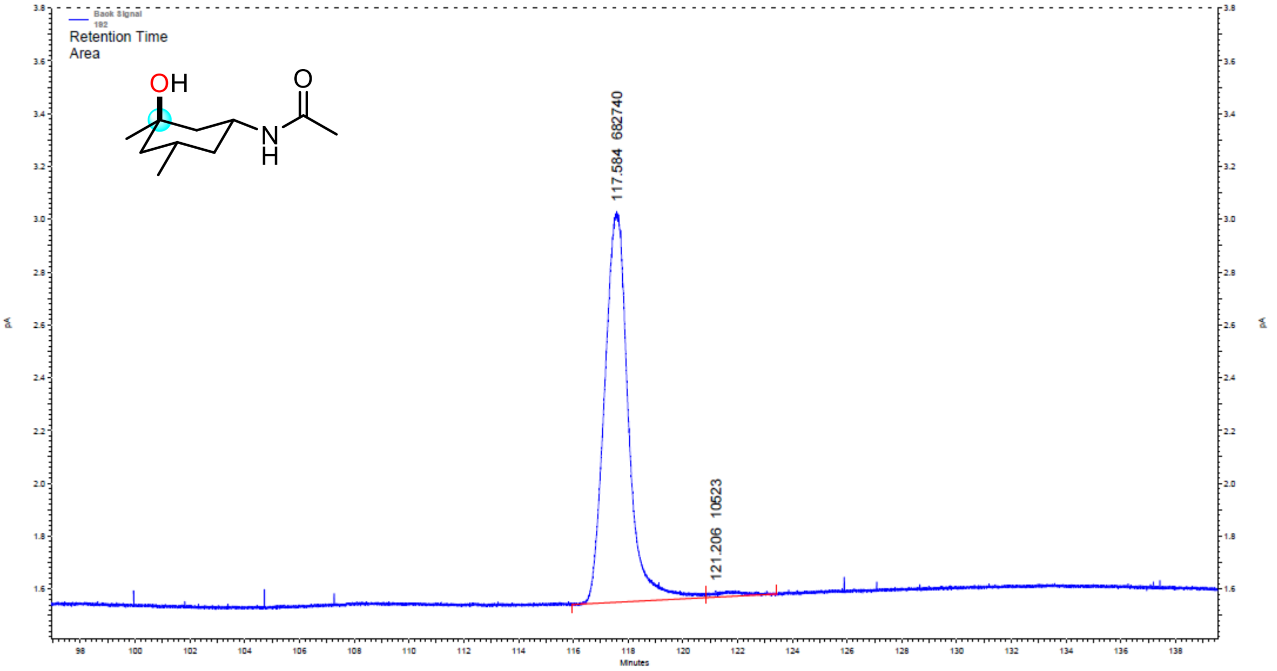

***Rac*-12b**

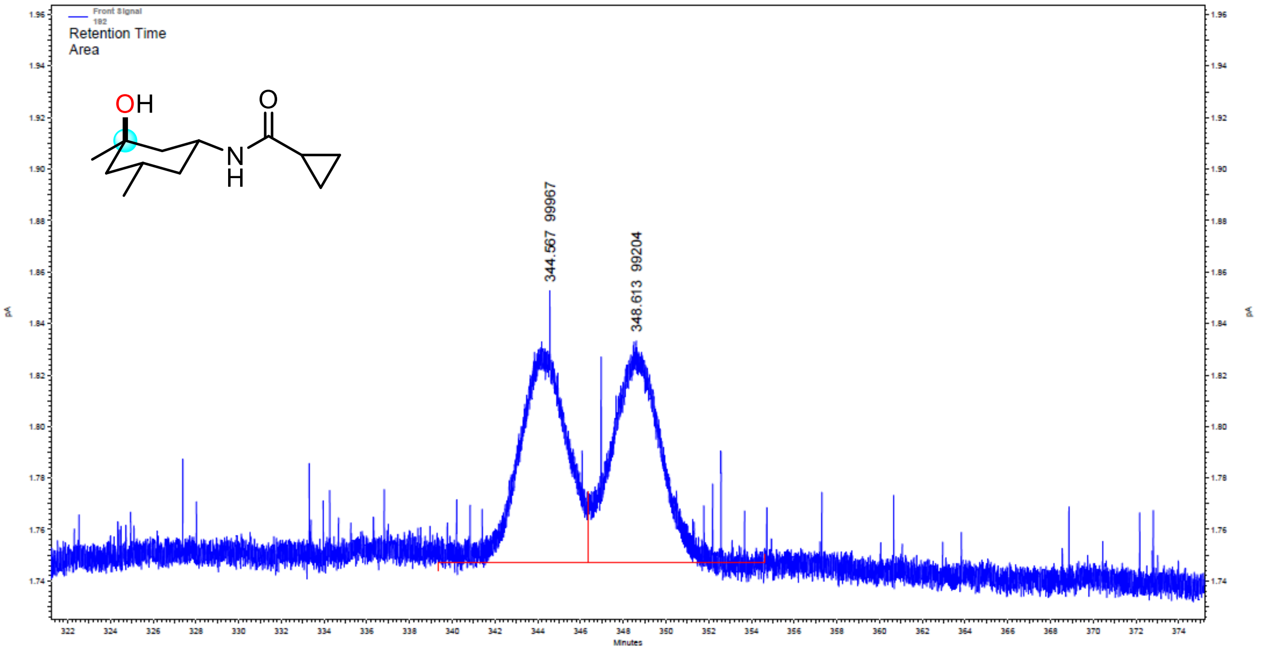

***(R,R)-Mn(^{TIPS}pdp)*-12b**

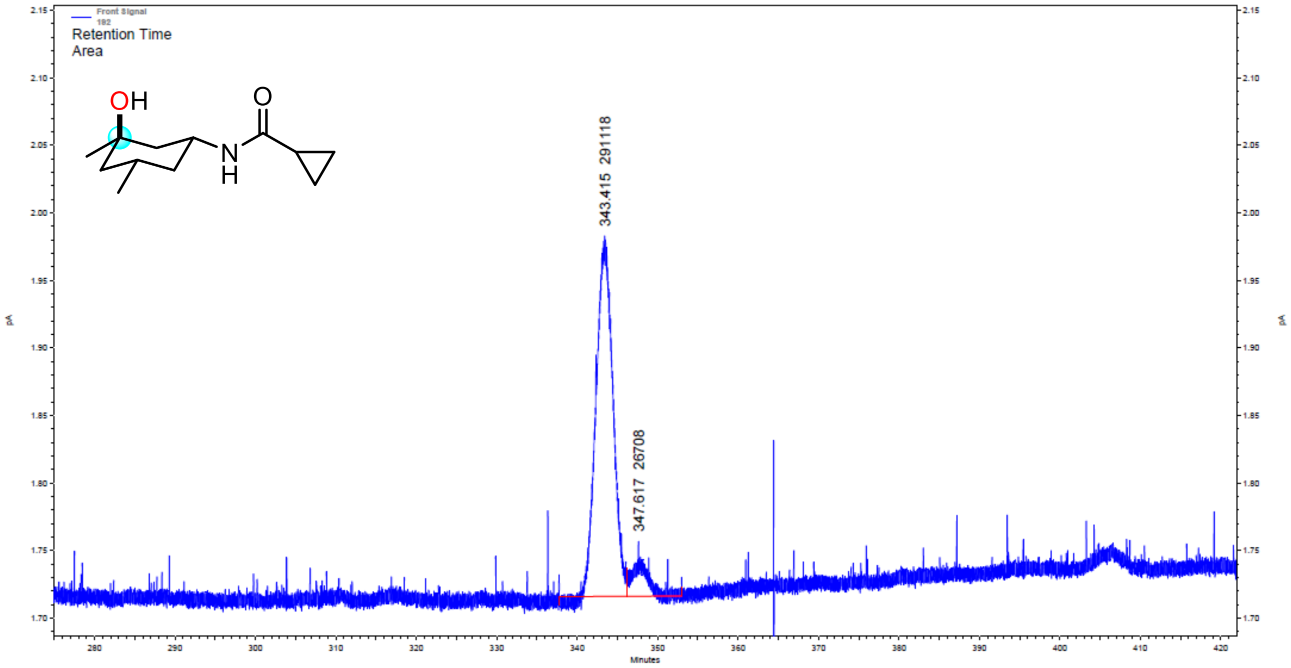

Rac-13b

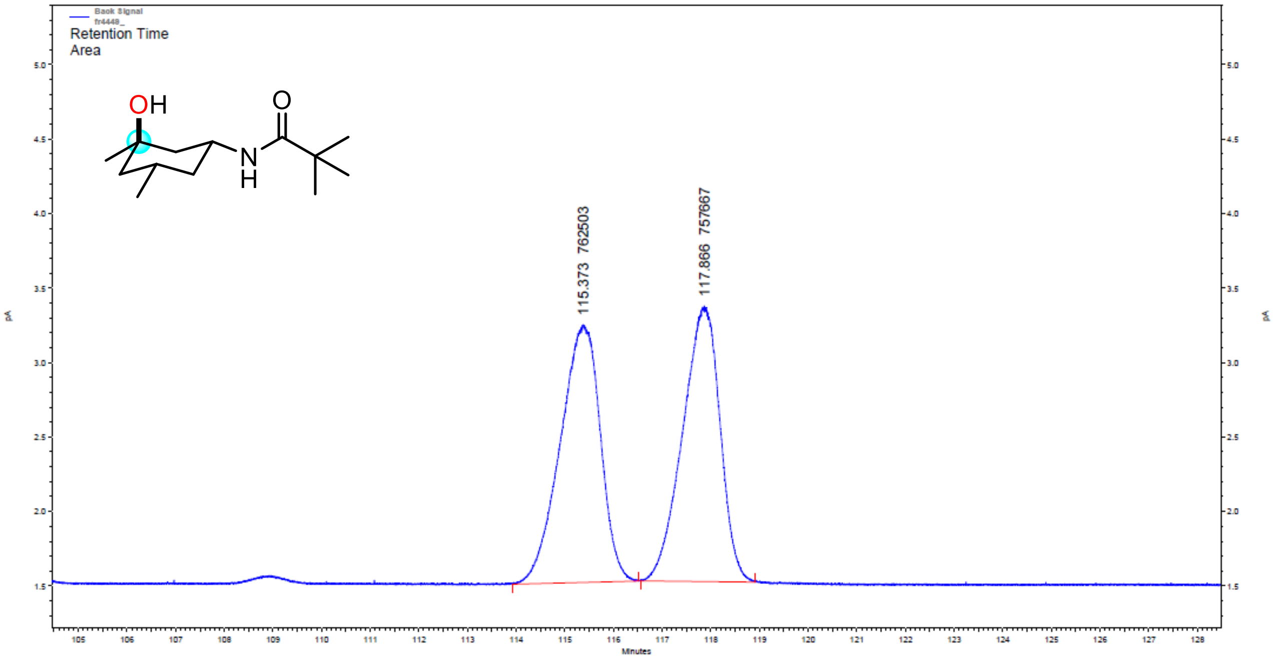

***(R,R)*-Mn(TIPSpdp)-13b**

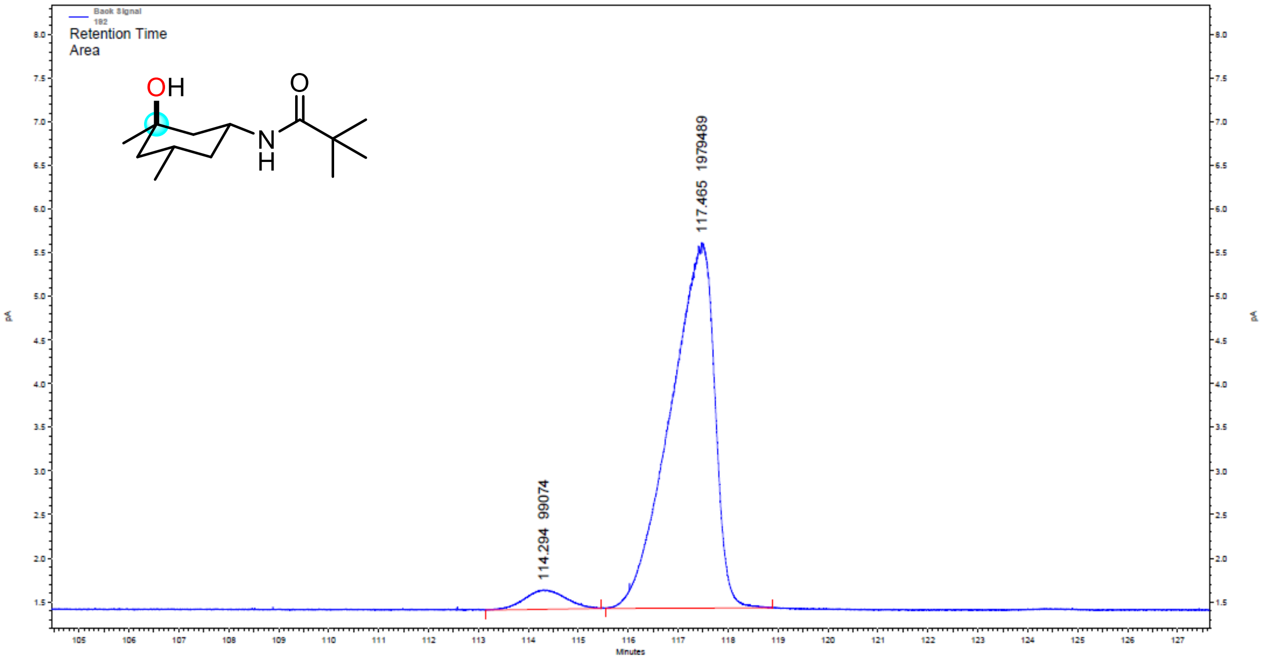

***Rac*-14b**

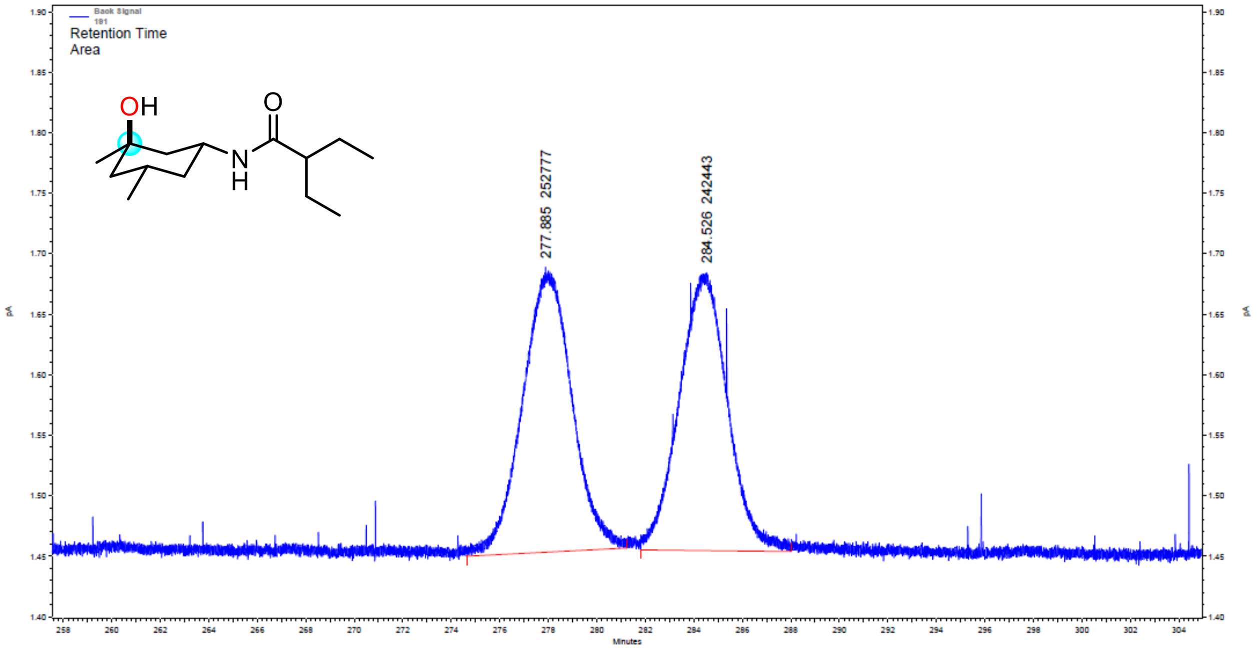

***(R,R)-Mn(TIPSpdp)*-14b**

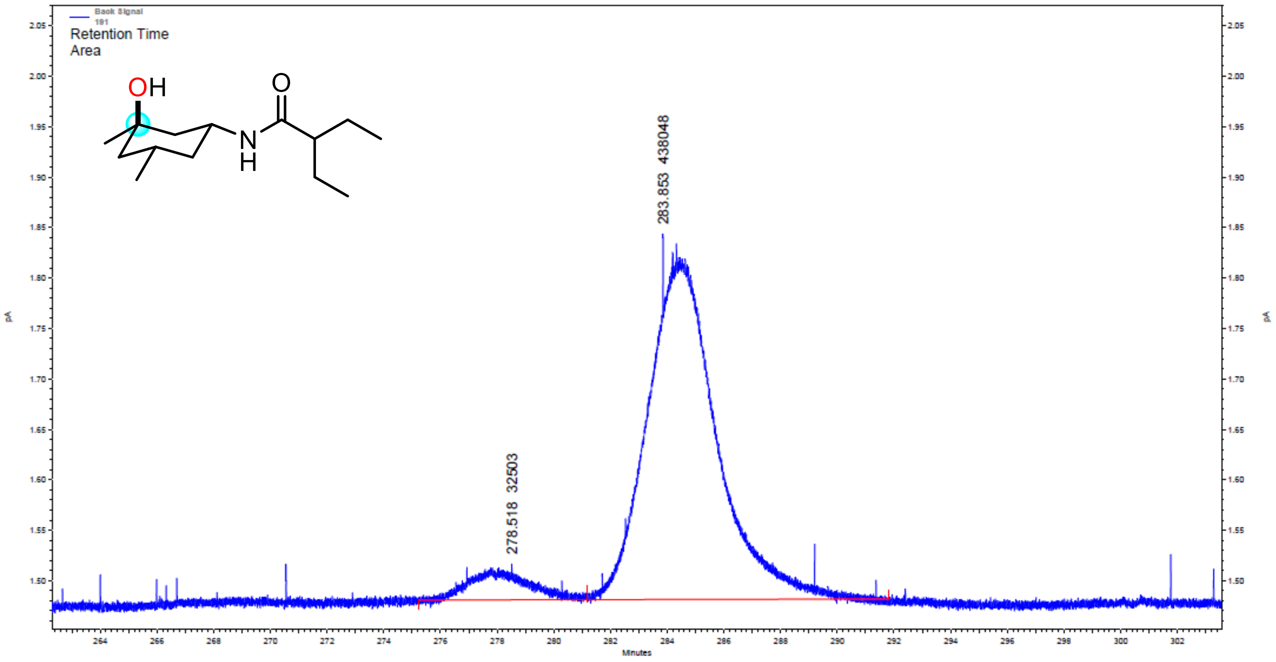

SFC separation conditions: Chiralpack IC-3 (100 × 4.6mm, 3µm), 210 nm, CO_2/ IPA= 75:25, 1.5 mL/min; r.t.(minor) = 1.45 min, r.t.(major) = 1.90 min.

***Rac*-15b**[D]

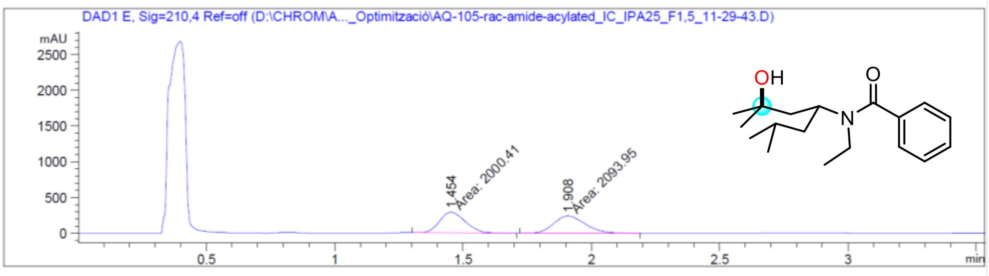

cv

Peak #	RetTime [min]	Type	Width [min]	Area [mAU*s]	Height [mAU]	Area %
1	1.454	MM	0.1159	2000.40930	287.64645	48.8577
2	1.908	MM	0.1449	2093.94653	240.81343	51.1423

***(R,R)-Mn(TIPSpdp)*-15b**[D]

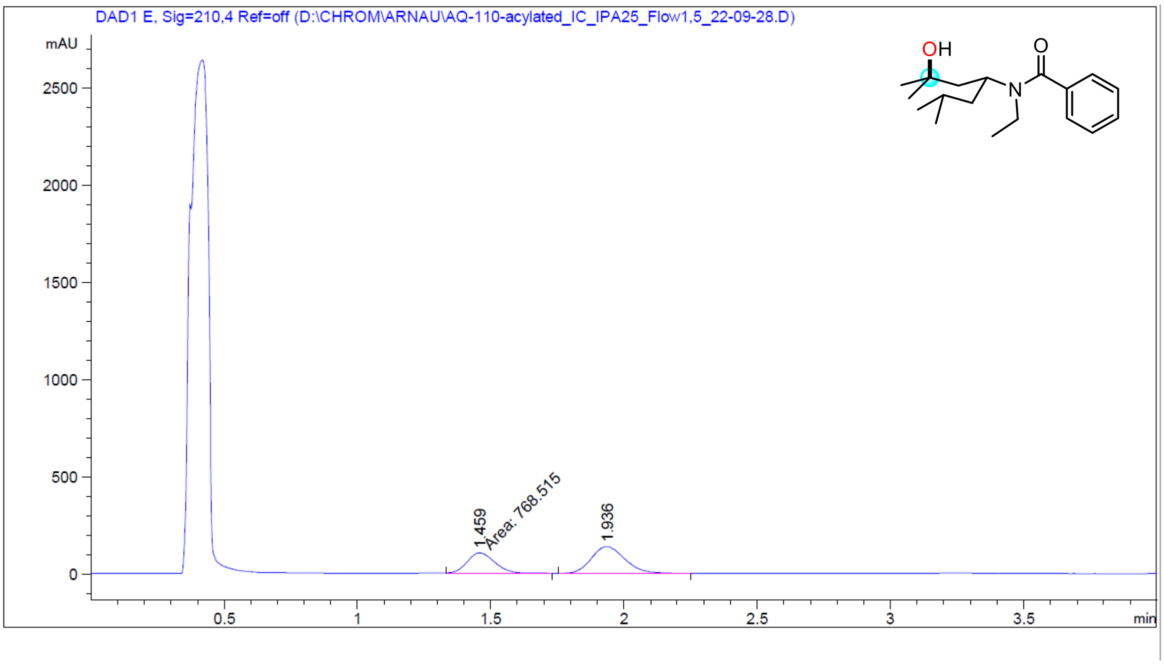

Peak #	RetTime [min]	Type	Width [min]	Area [mAU*s]	Height [mAU]	Area %
1	1.459	FM	0.1208	768.51471	106.01713	38.7189
2	1.936	BV R	0.1375	1216.34302	138.44902	61.2811

***Rac*-16b**

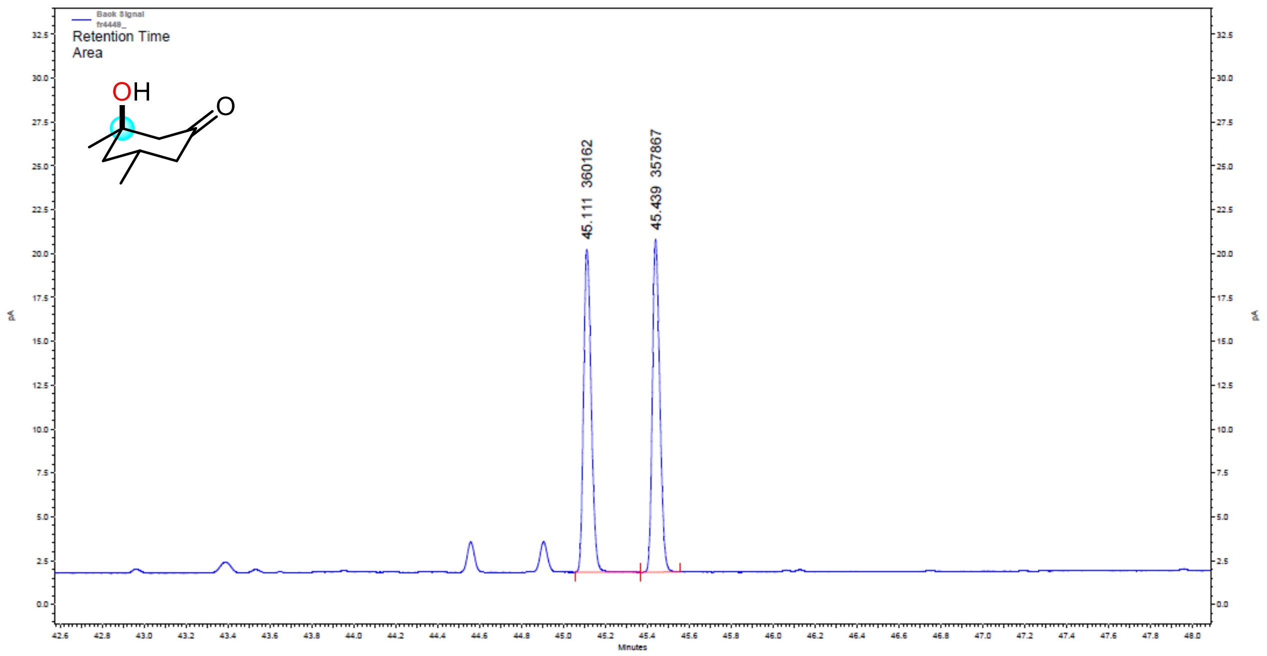

***(R,R)-Mn(TIPSpdp)*-P16b**

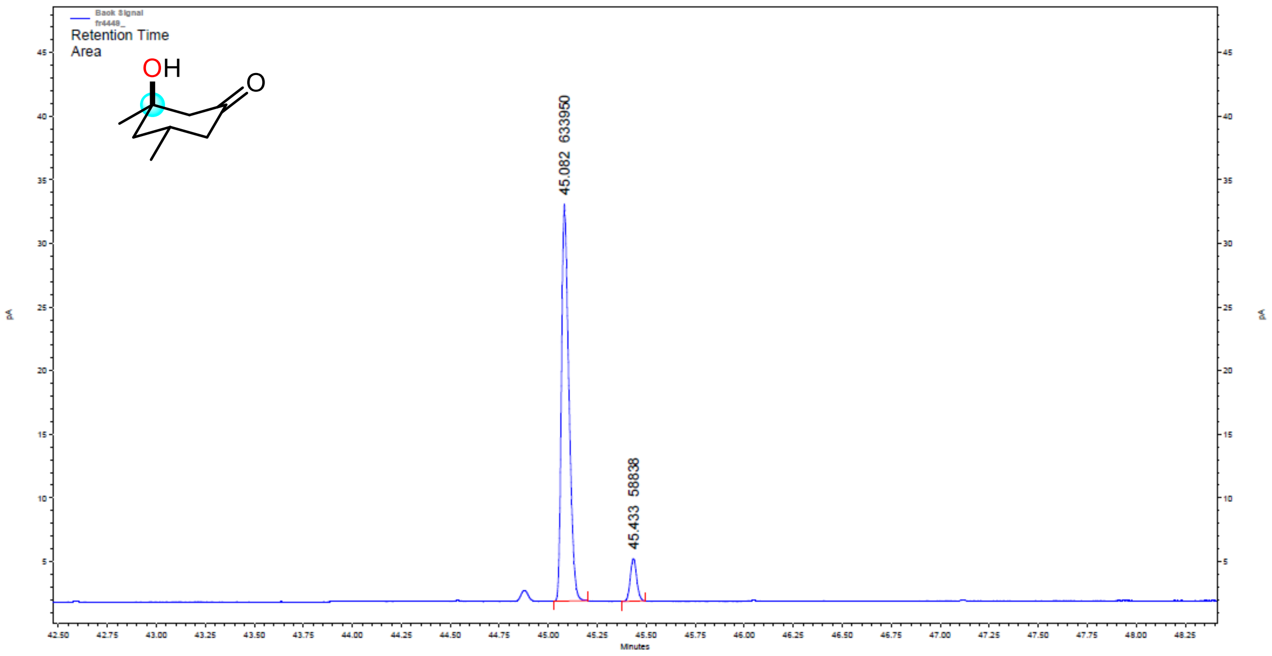

Rac-17b

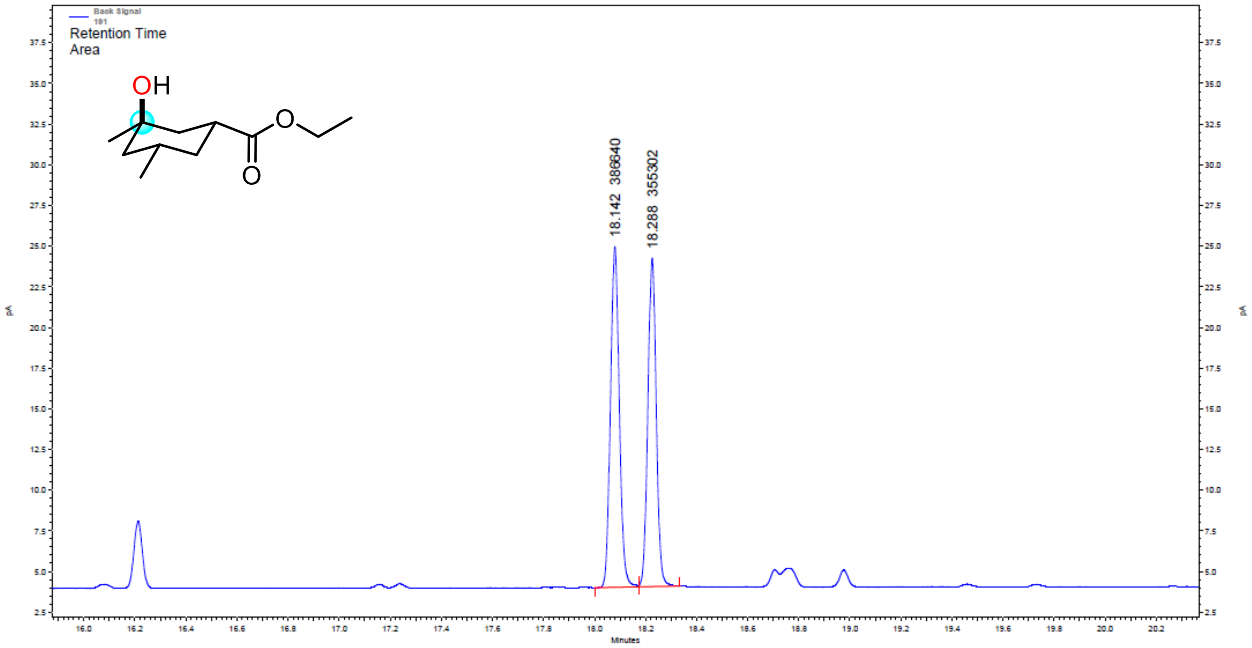

(R,R)-Mn(^{TIPS} ***pdp)*-17b**

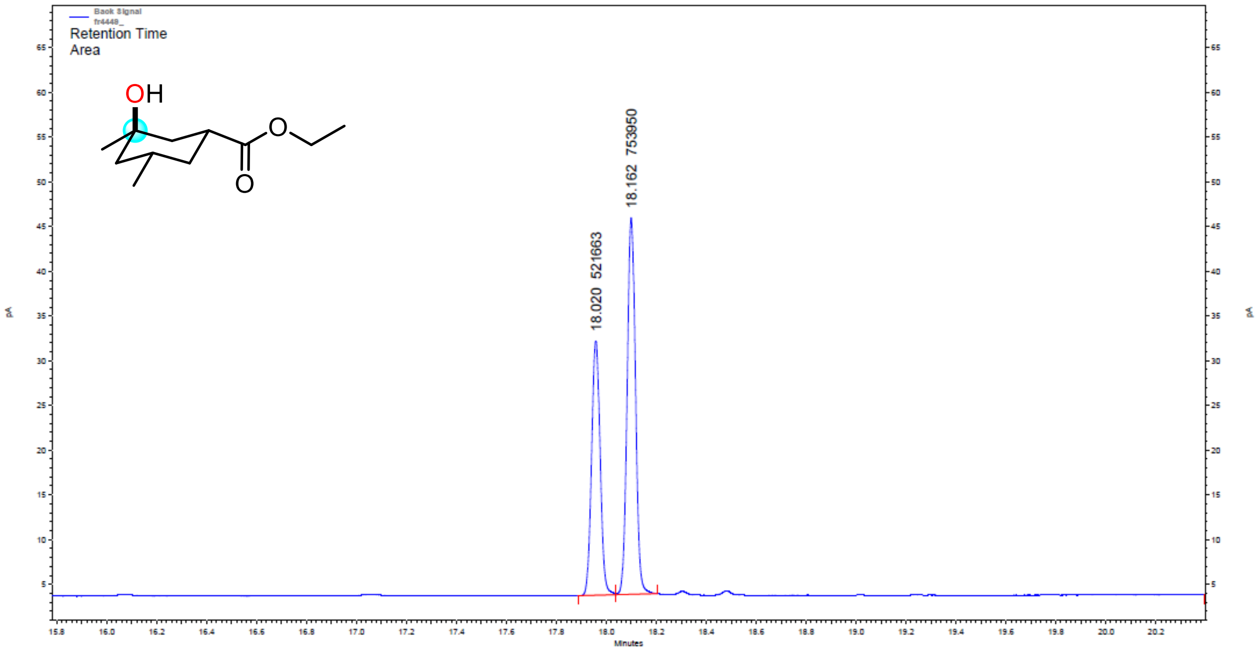

***Rac*-18b**

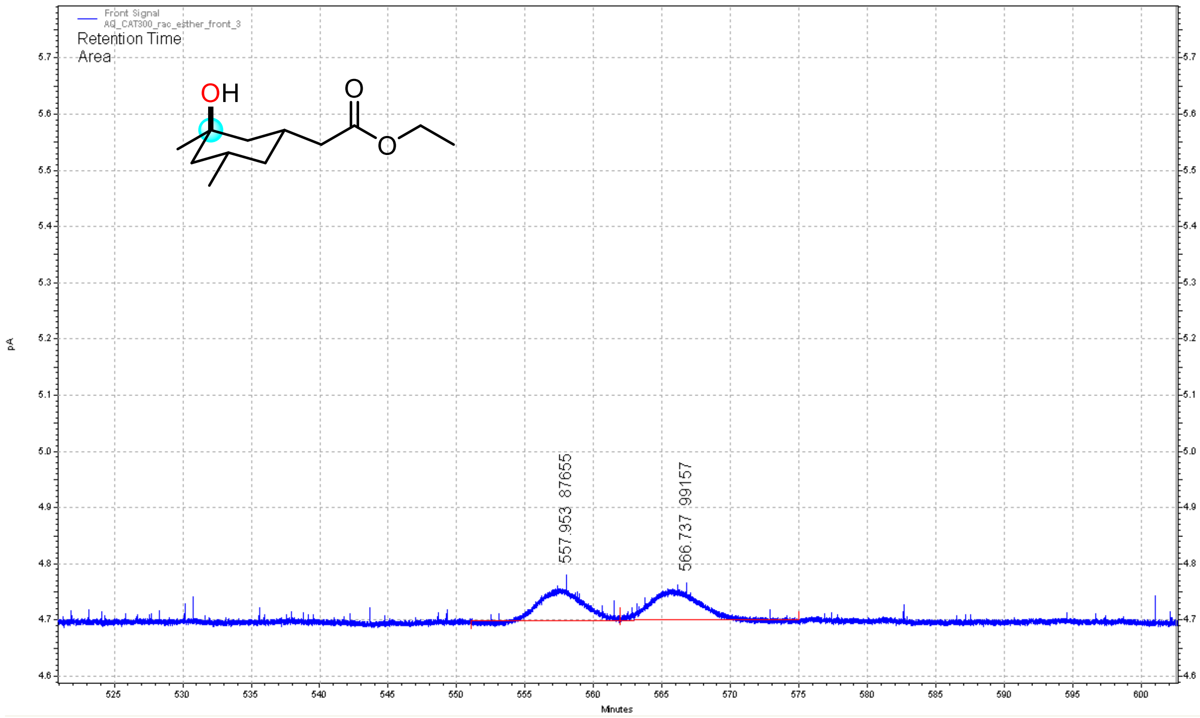

***(R,R)-Mn(TIPSpdp)*-18b**

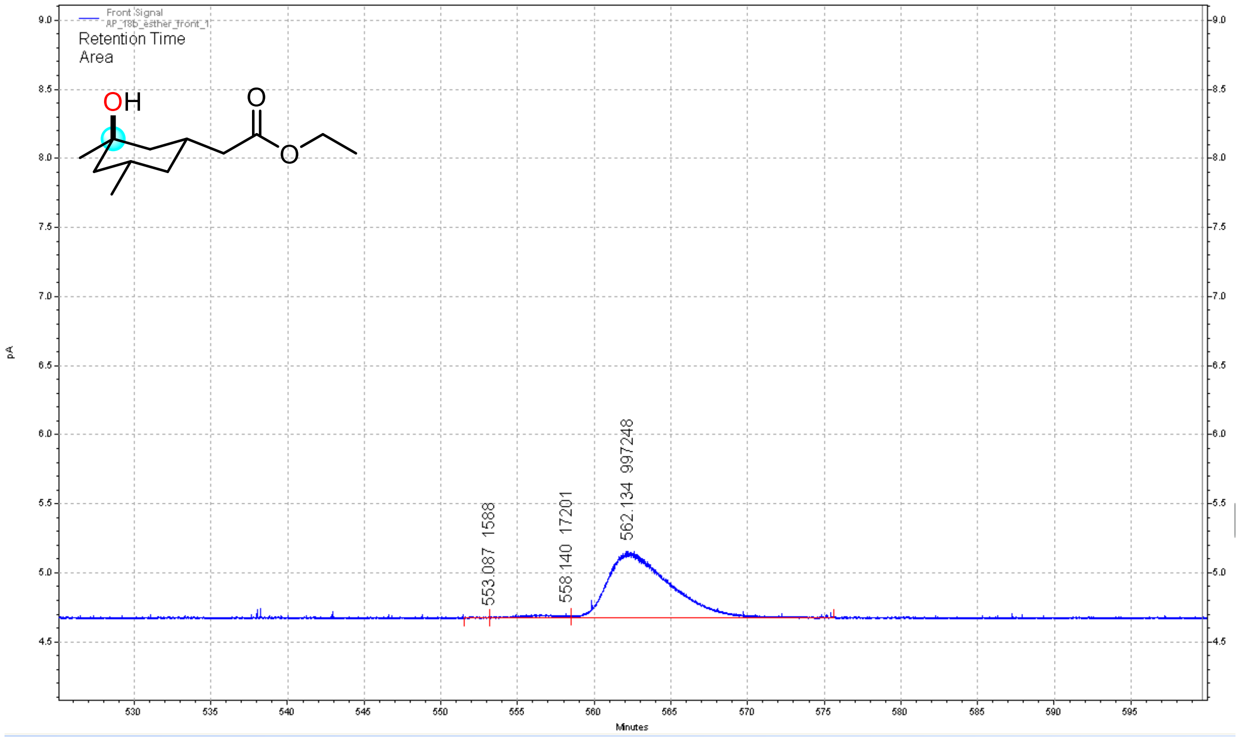

SFC separation conditions: Chiralpack IB-3 (100 × 4.6mm, 3µm), 350 nm, CO_2/IPA= 90:10, 1.5 mL/min; *Major rotamer*: r.t.(major) = 1.58 min, r.t.(minor) = 1.93 min. Minor rotamer: r.t.(major) = 2.29 min, r.t.(minor) = 2.70 min.

***Rac*-19b**[D] (Mixture of two rotamers)

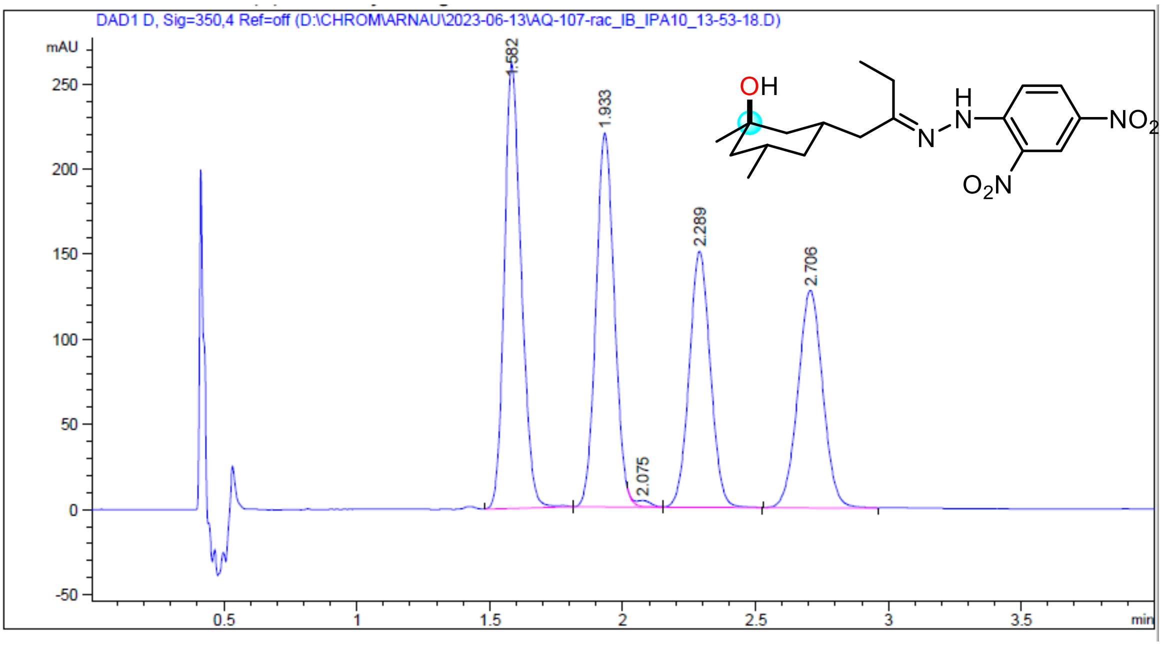

Peak #	RetTime [min]	Type	Width [min]	Area [mAU*s]	Height [mAU]	Area %
1	1.582	BV R	0.0671	1155.83313	261.42728	29.7123
2	1.933	BV R	0.0743	1059.56641	219.73148	27.2376
3	2.075	VB E	0.0585	13.04655	3.42234	0.3354
4	2.289	BB	0.0850	830.37836	150.23837	21.3460
5	2.706	BB	0.0999	831.26355	127.69347	21.3688

***(R,R)*-Mn(^TIPS^pdp)-19b**[D] (The ee was found to be the same (95% ee) in both rotamers)

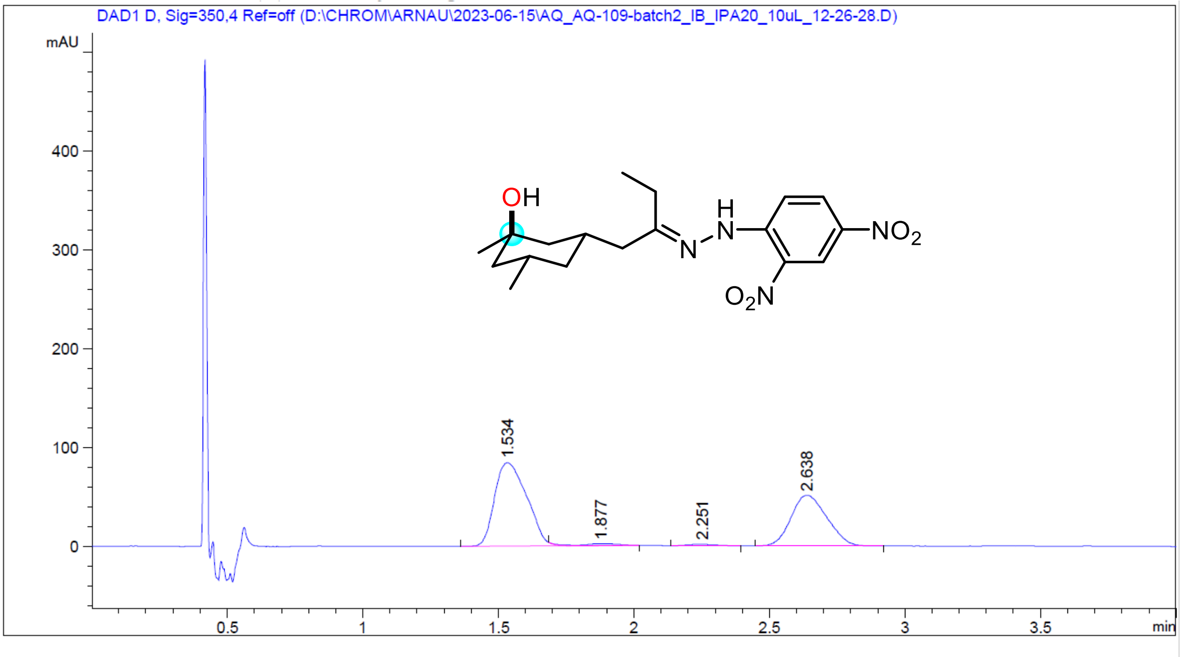

Peak #	RetTime [min]	Type	Width [min]	Area [mAU*s]	Height [mAU]	Area %
1	1.534	BV R	0.1291	693.70209	84.23105	57.9313
2	1.877	VB E	0.1060	17.80525	2.01679	1.4869
3	2.251	BB	0.0957	12.02214	1.52877	1.0040
4	2.638	BV R	0.1380	473.92758	51.44169	39.5778

SFC separation conditions: Chiralpack IG-3 (100 × 4.6mm, 3μm), 198.4 nm, CO_2/MeOH= 90:10, 1.5 mL/min; r.t.(minor) = 2.24 min, r.t.(major) = 2.60 min.

***Rac*-20b**

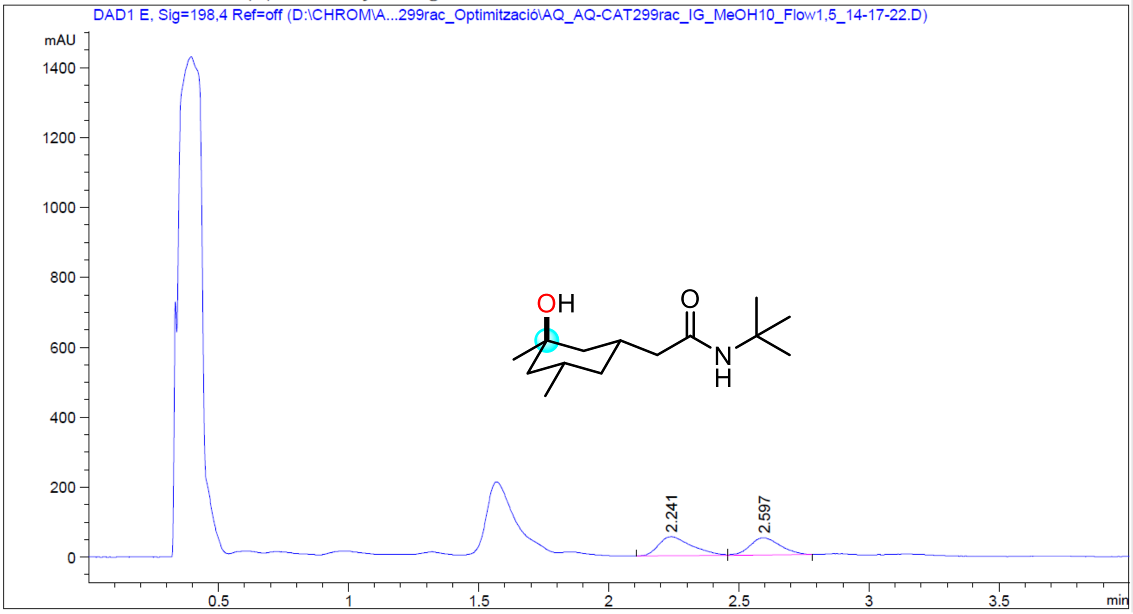

Peak #	RetTime [min]	Type	Width [min]	Area [mAU*s]	Height [mAU]	Area %
1	2.241	BV	0.1216	481.84277	54.81714	54.6287
2	2.597	VB	0.0987	400.18991	49.29714	45.3713

(R,R)-Mn(TIPSpdp)-20b

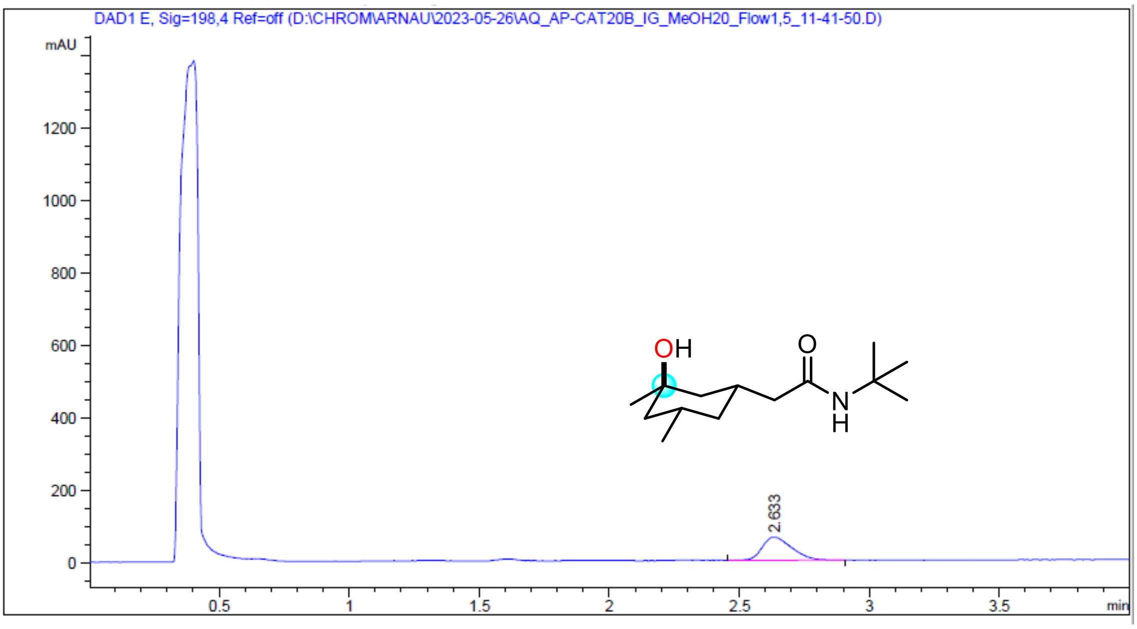

Peak #	RetTime [min]	Type	Width [min]	Area [mAU*s]	Height [mAU]	Area %
1	2.633	VV R	0.1143	515.75256	64.47321	100.0000

SFC separation conditions: Chiralpack IA-3 (100 × 4.6mm, 3µm), 220.4 nm, CO_2/MeOH= 80:20, 1.5 mL/min; r.t.(major) = 1.54 min, r.t.(minor) = 2.20 min.

***Rac*-21b**[D]

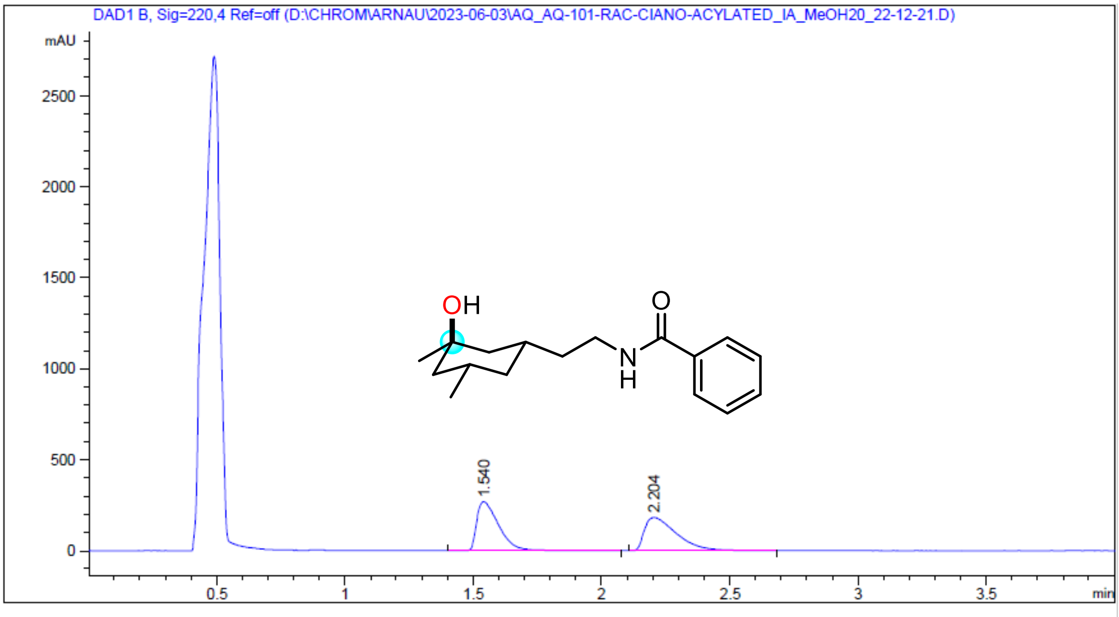

Peak #	RetTime [min]	Type	Width [min]	Area [mAU*s]	Height [mAU]	Area %
1	1.540	VV R	0.0931	1591.36743	268.63065	50.1807
2	2.204	BV R	0.1312	1579.90723	182.19939	49.8193

(R,R)-Mn(^{TIPS}pdp)-21b[D]

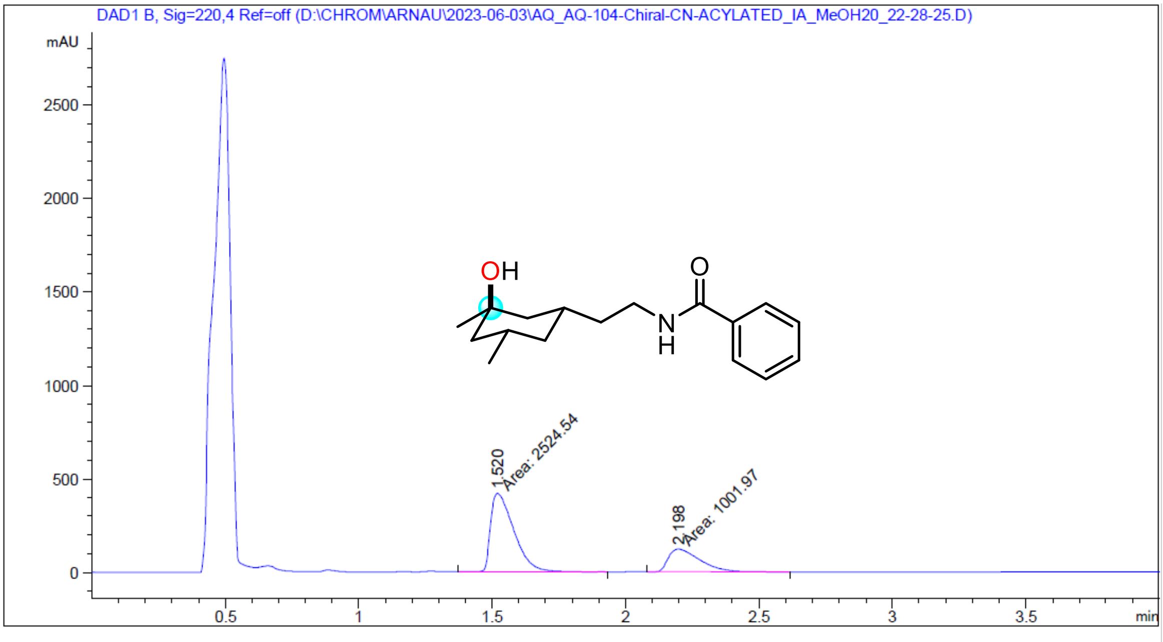

Peak #	RetTime [min]	Type	Width [min]	Area [mAU*s]	Height [mAU]	Area %
1	1.520	MM	0.1003	2524.53564	419.61105	71.5874
2	2.198	MM	0.1383	1001.97223	120.70780	28.4126

***Rac*-22b**

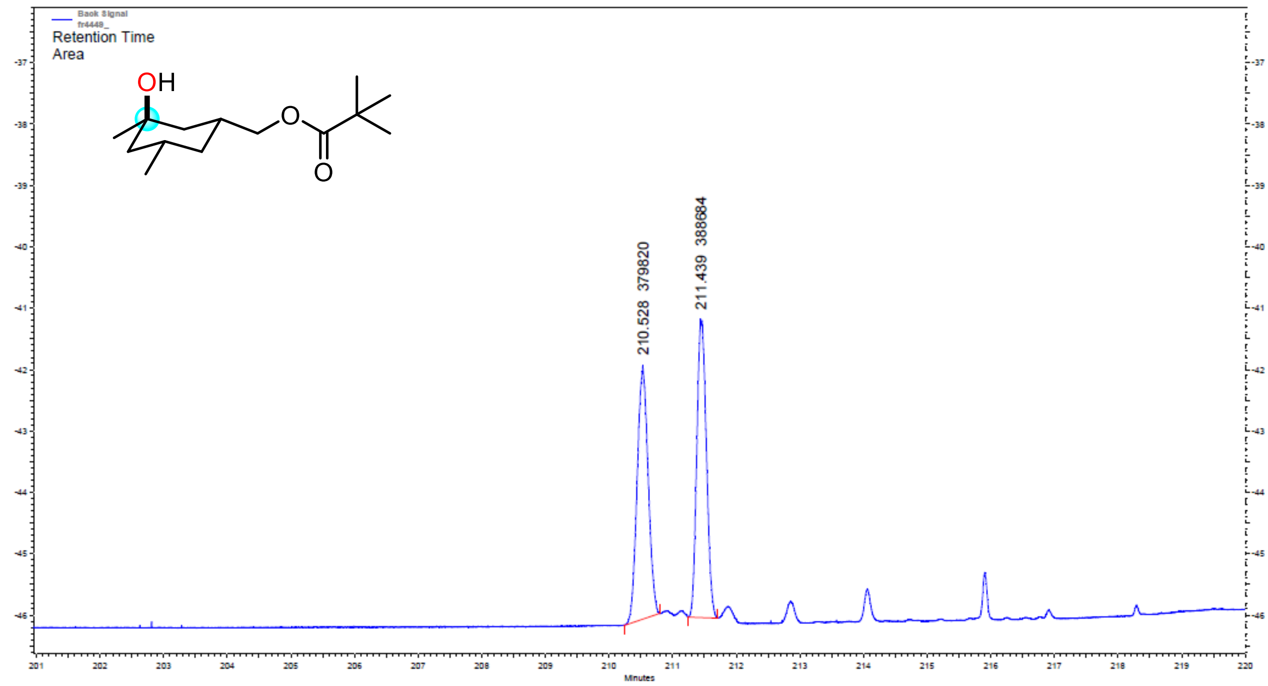

(R,R)-Mn(^{TIPS}pdp)-22b

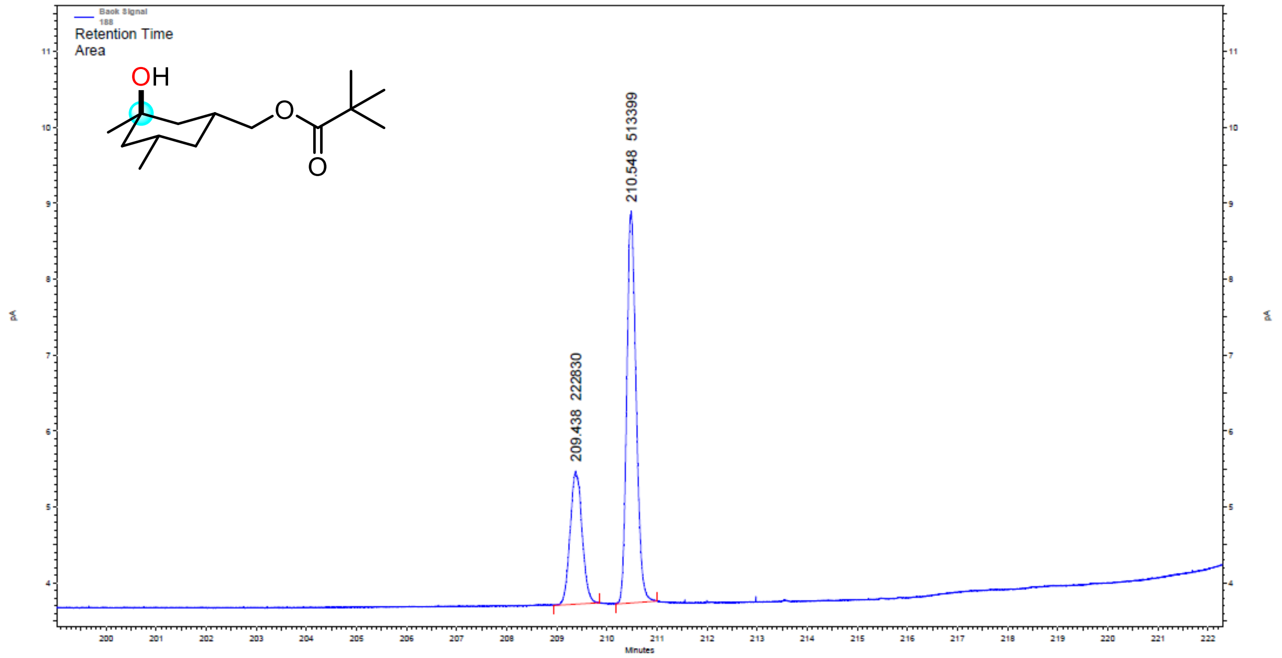

SFC separation conditions: Chiralpack IG-3 (100 × 4.6mm, 3µm), 35ºC, 220.4 nm, CO_2/ EtOH= 75:25, 1.2 mL/min; r.t.(*major*) = 2.179 min, r.t.(*minor*) = 2.402 min.

***Rac*-23b**

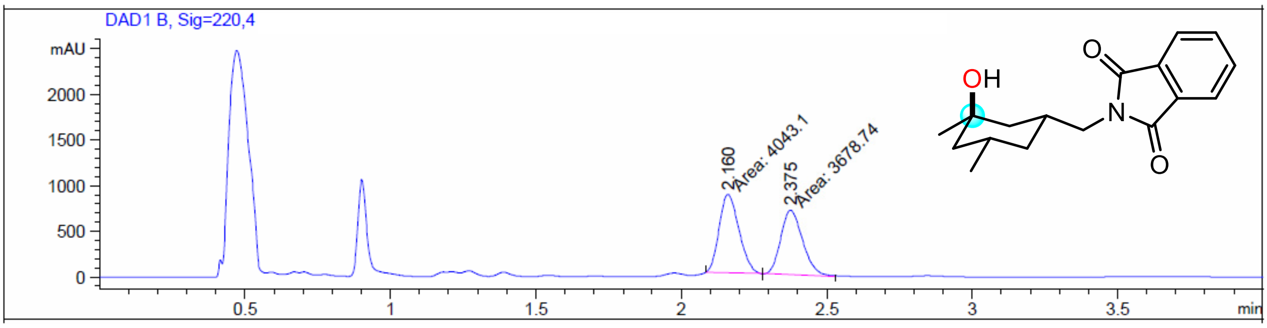

Peak #	RetTime [min]	Type	Width [min]	Area [mAU*s]	Height [mAU]	Area %
1	2.160	MM	0.0786	4043.10327	857.43341	52.3593
2	2.375	FM	0.0871	3678.73682	703.58752	47.6407

***(R,R)-Mn(TIPSpdp)*-23b**

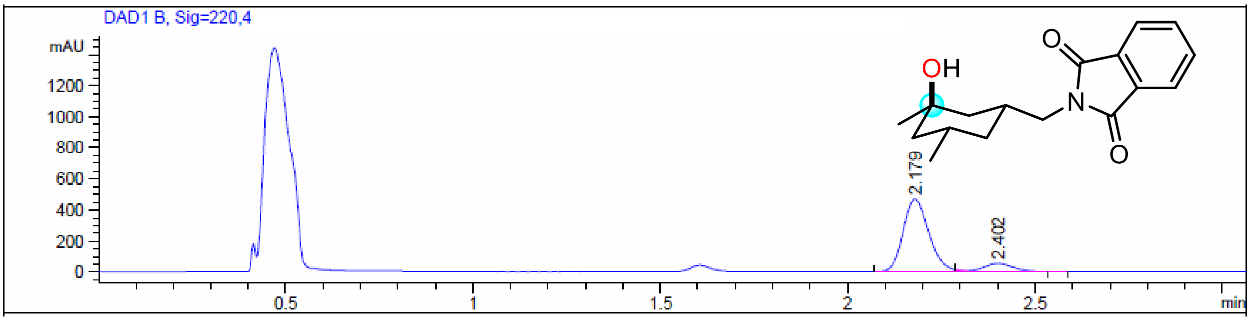

Peak #	RetTime [min]	Type	Width [min]	Area [mAU*s]	Height [mAU]	Area %
1	2.179	BV R	0.0736	2218.35791	465.62137	88.7804
2	2.402	VV E	0.0831	280.34460	51.41187	11.2196

Rac-24b

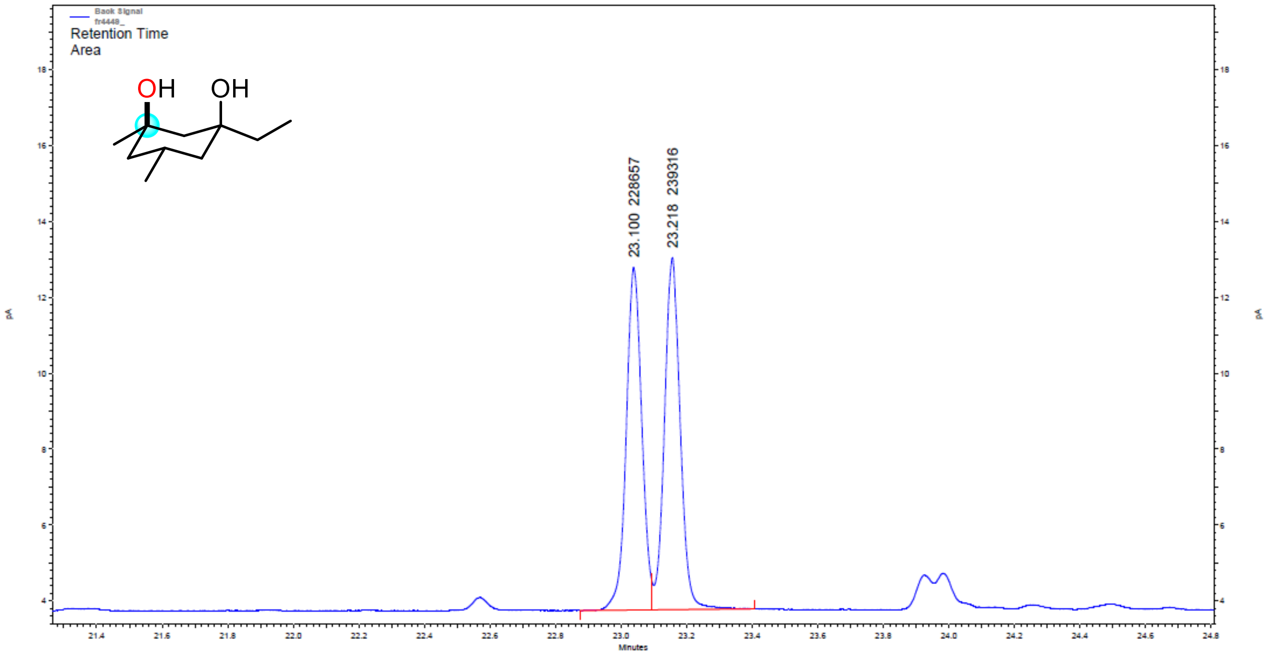

(R,R)-Mn(^{TIPS}pdp)-24b

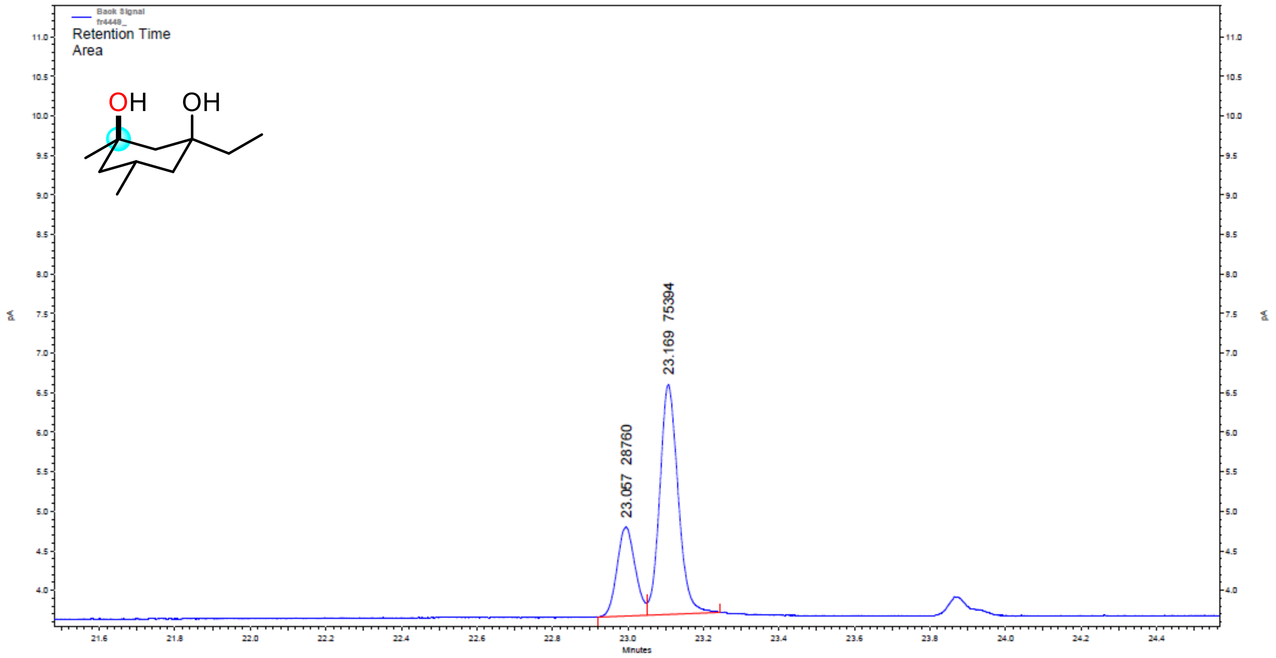

***Rac*-25b**

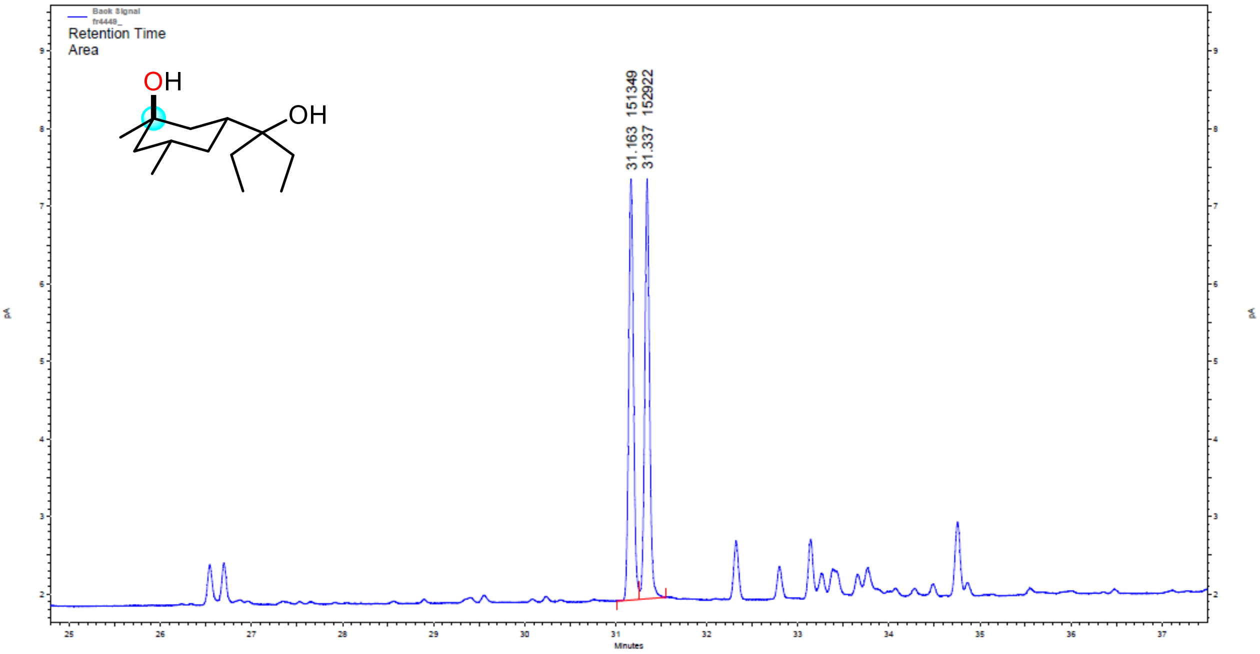

***(R,R)-Mn(TIPSpdp)*-25b**

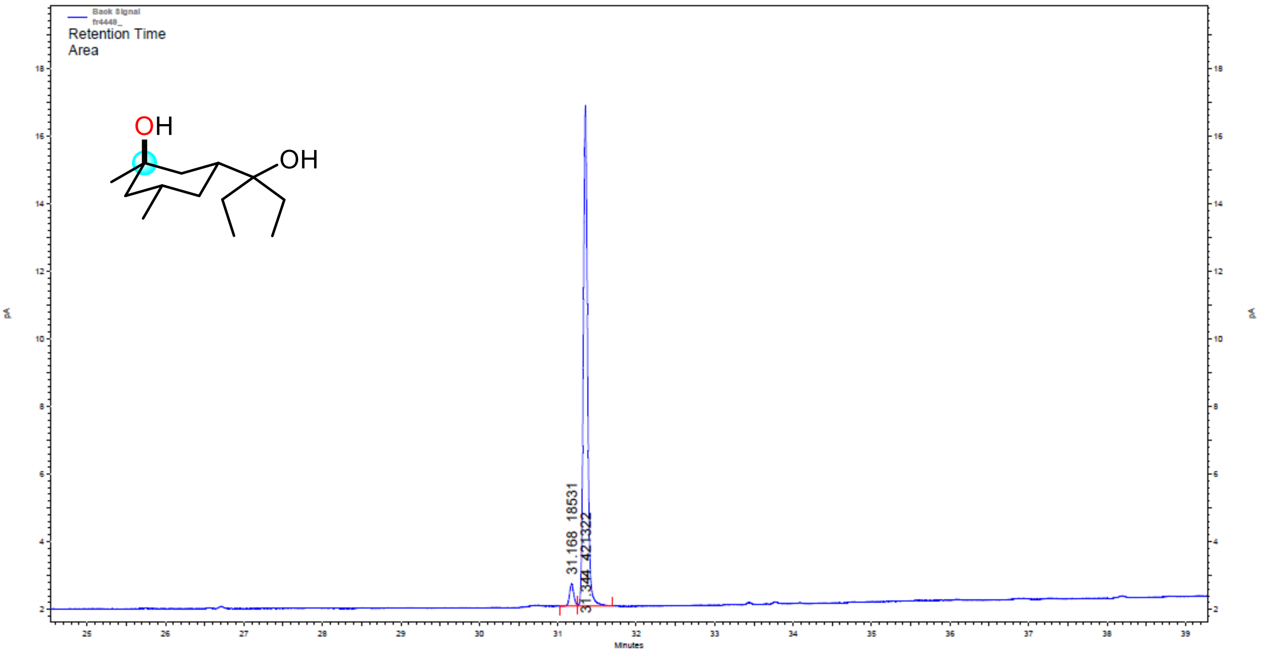

***Rac*-26b**

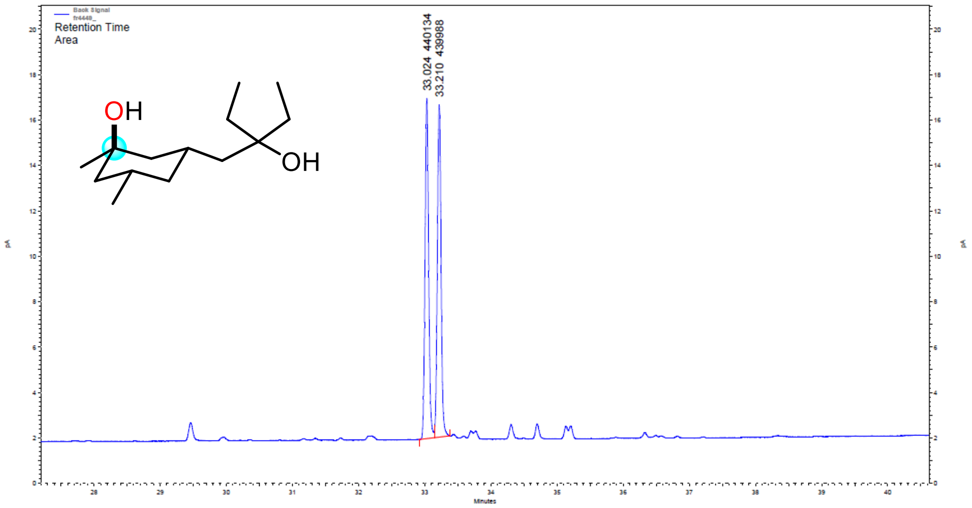

***(R,R)-Mn(^{TIPS}pdp)*-26b** Method B

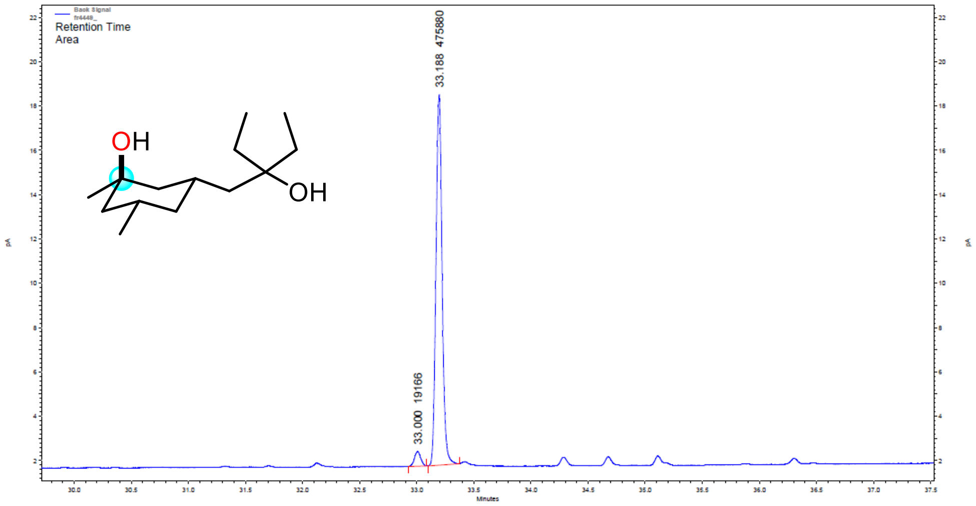

***(R,R)-Mn(^{TIPS}pdp)*-26b** Method A

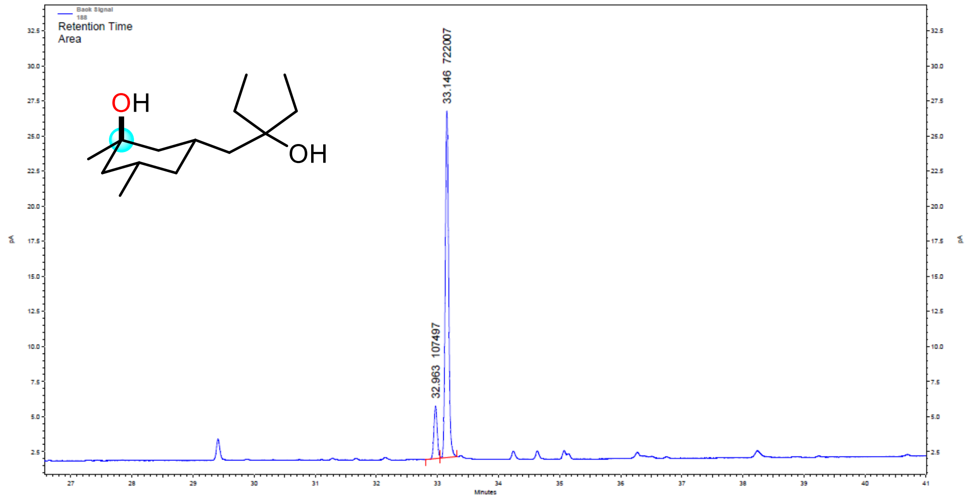

Rac-27b

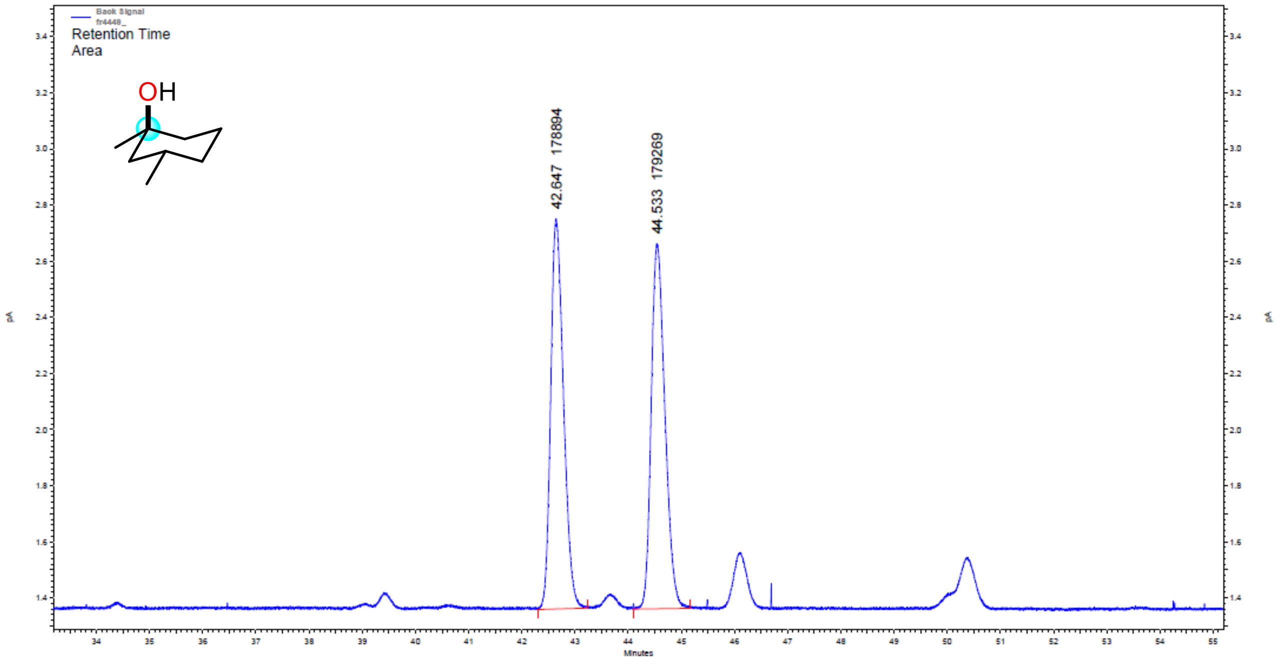

***(R,R)*-Mn(*^{TIPS}*pdp)*-27b**

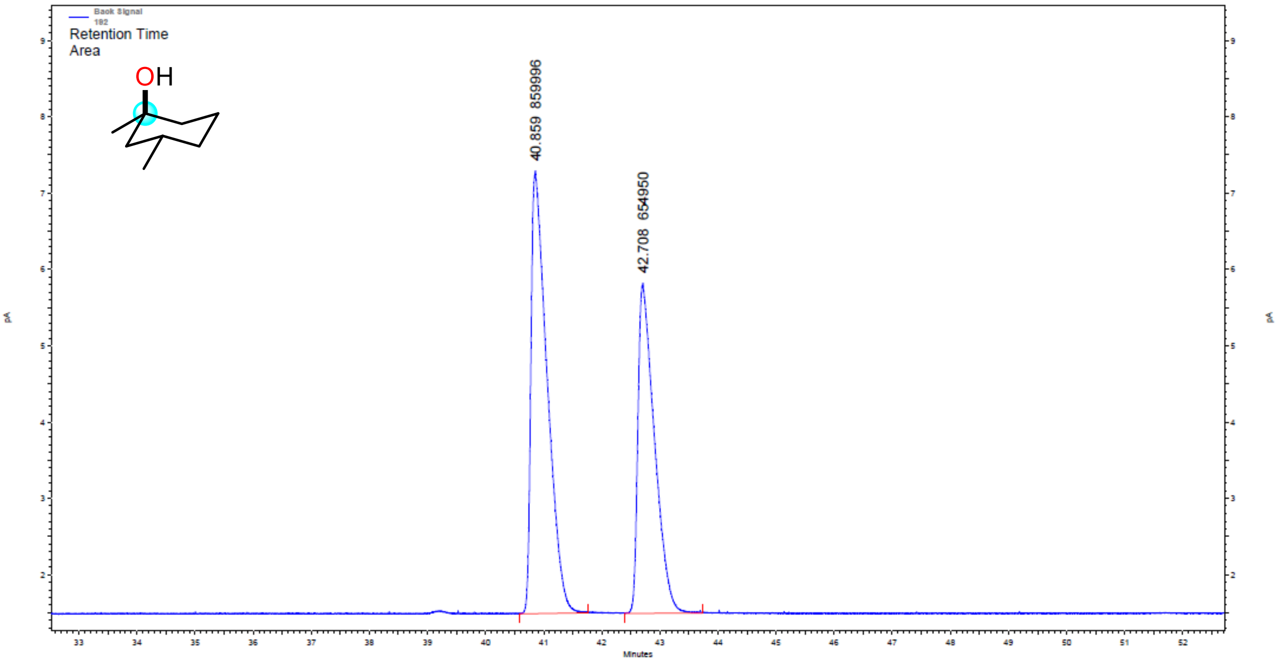

***Rac*-28b**

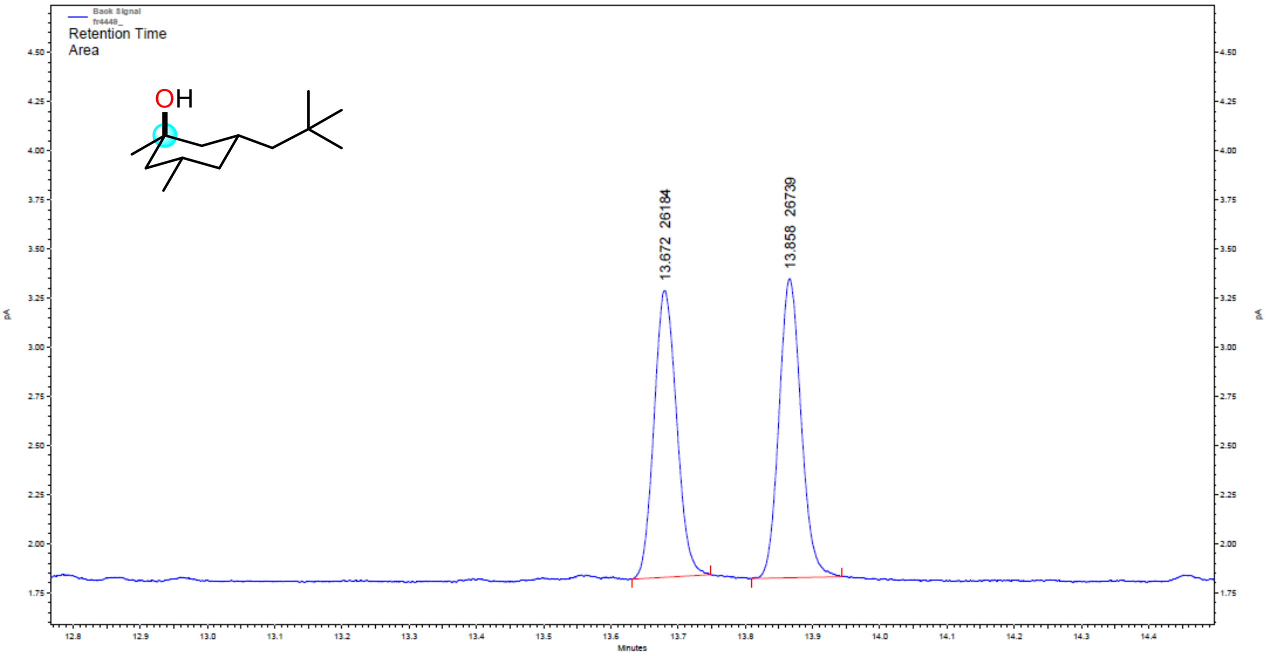

***(R,R)-Mn(TIPSpdp)*-28b**

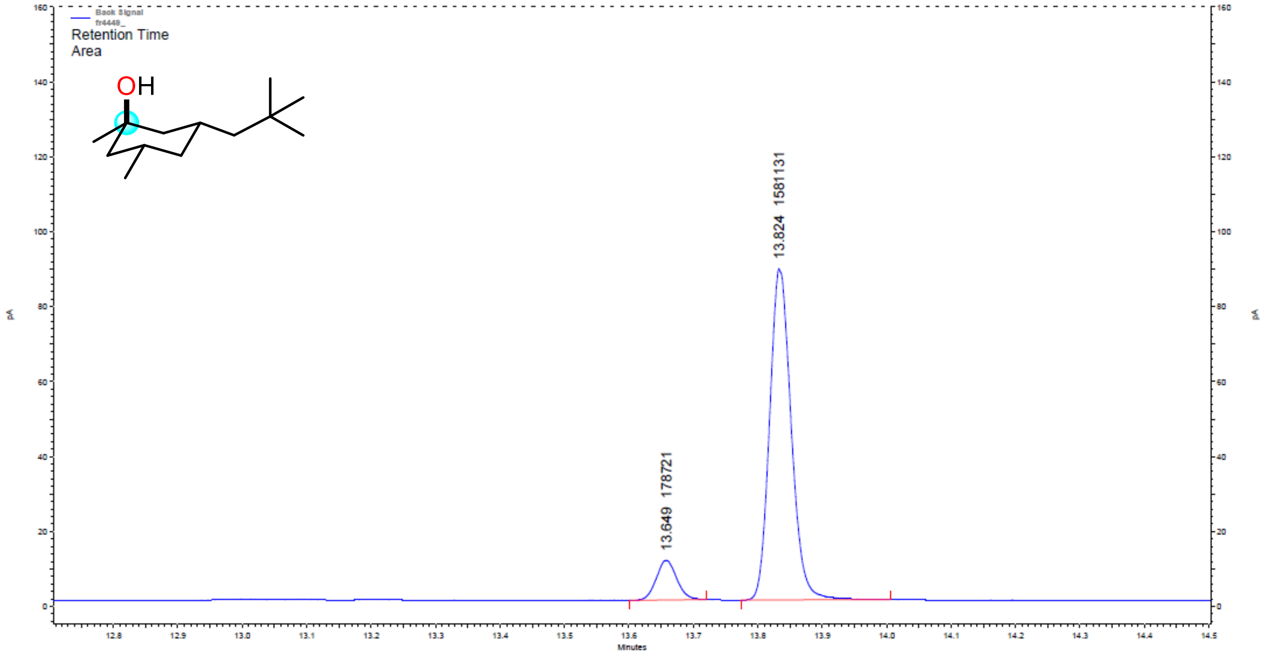

Rac-30c and 30d

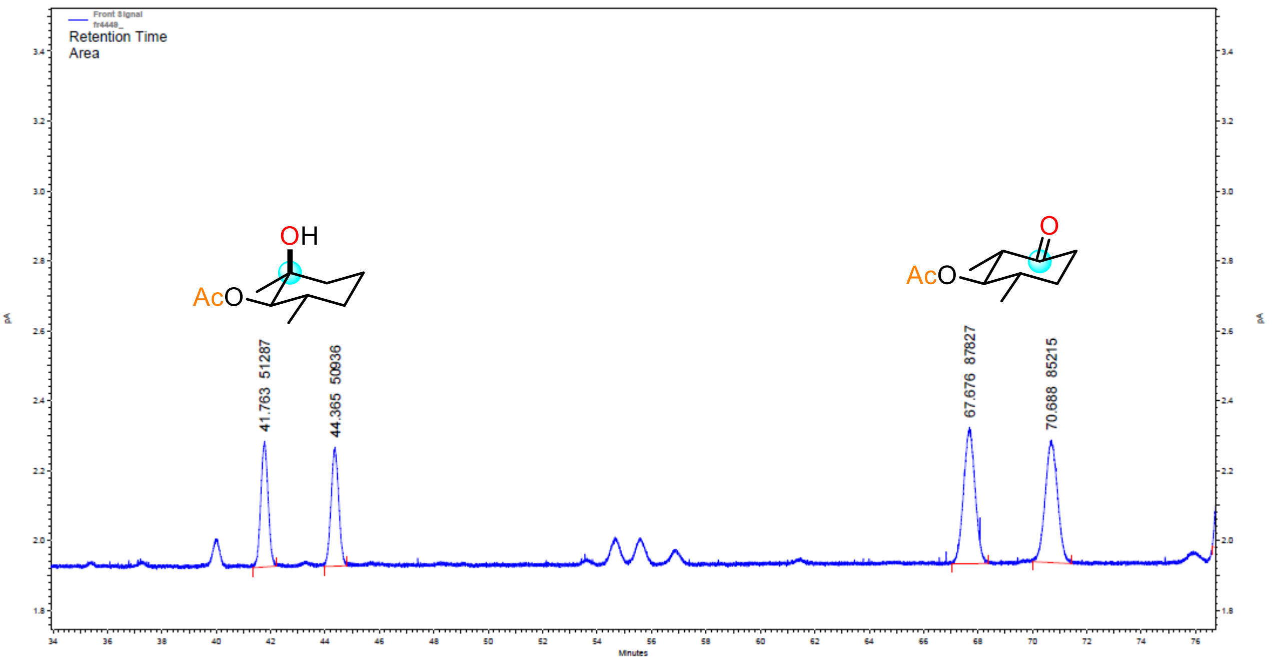

(R,R)-Mn(***TIPS pdp)*** **-30c and 30d**

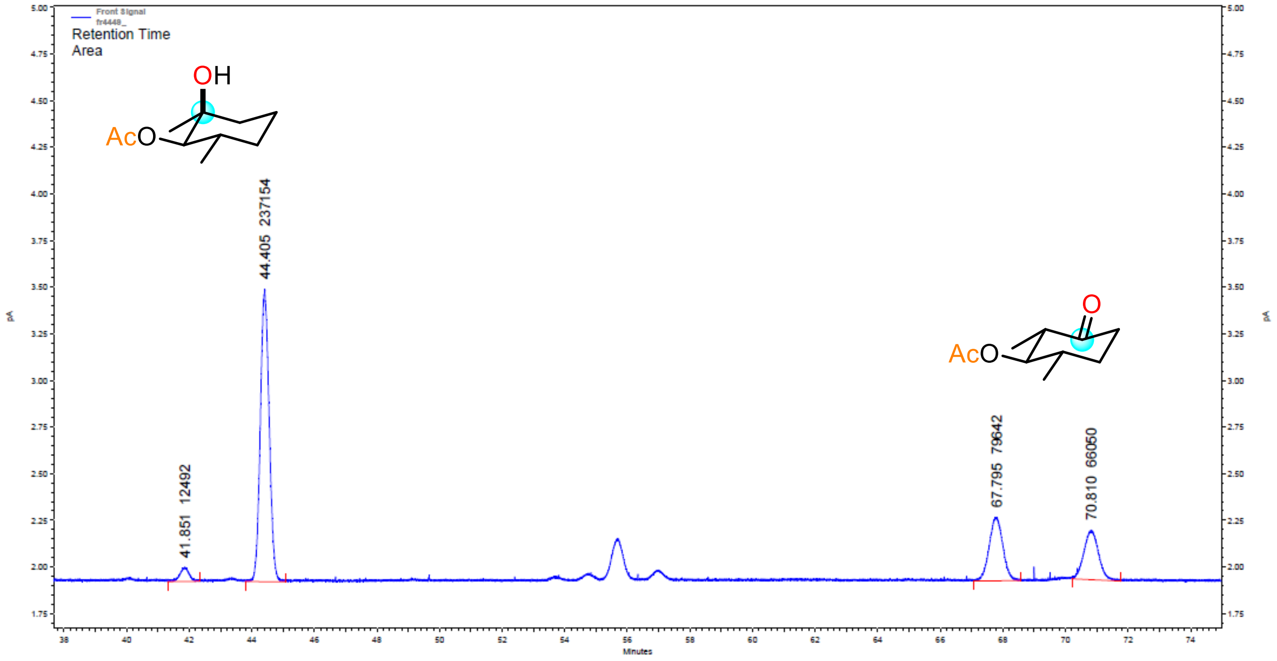

***Rac*-31b**

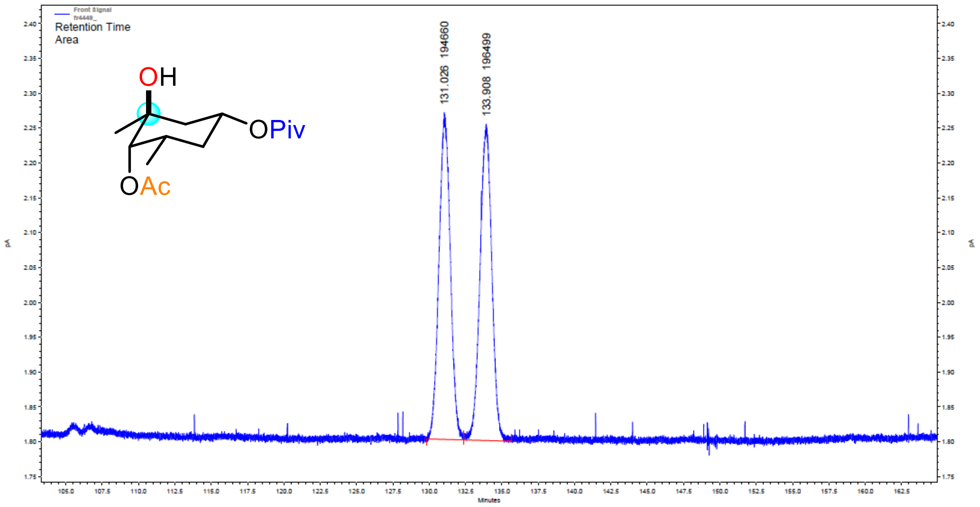

***(R,R)-Mn(^{TIPS}pdp)*-31b** Method B

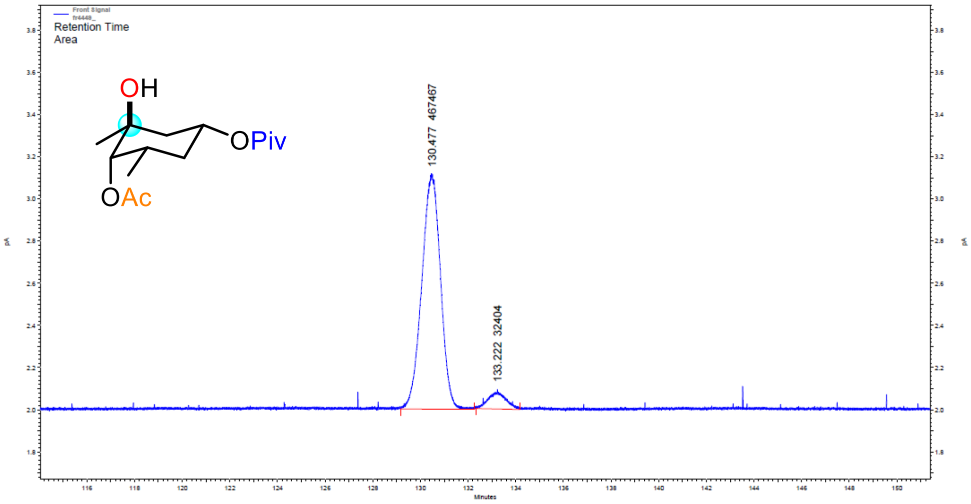

***(R,R)-Mn(^{TIPS}pdp)*-31b** Method A

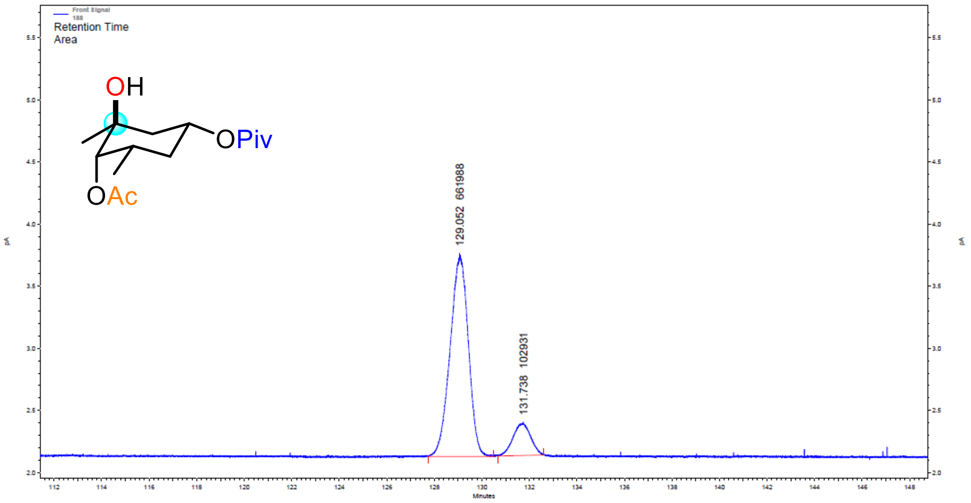

***Rac*-6e**

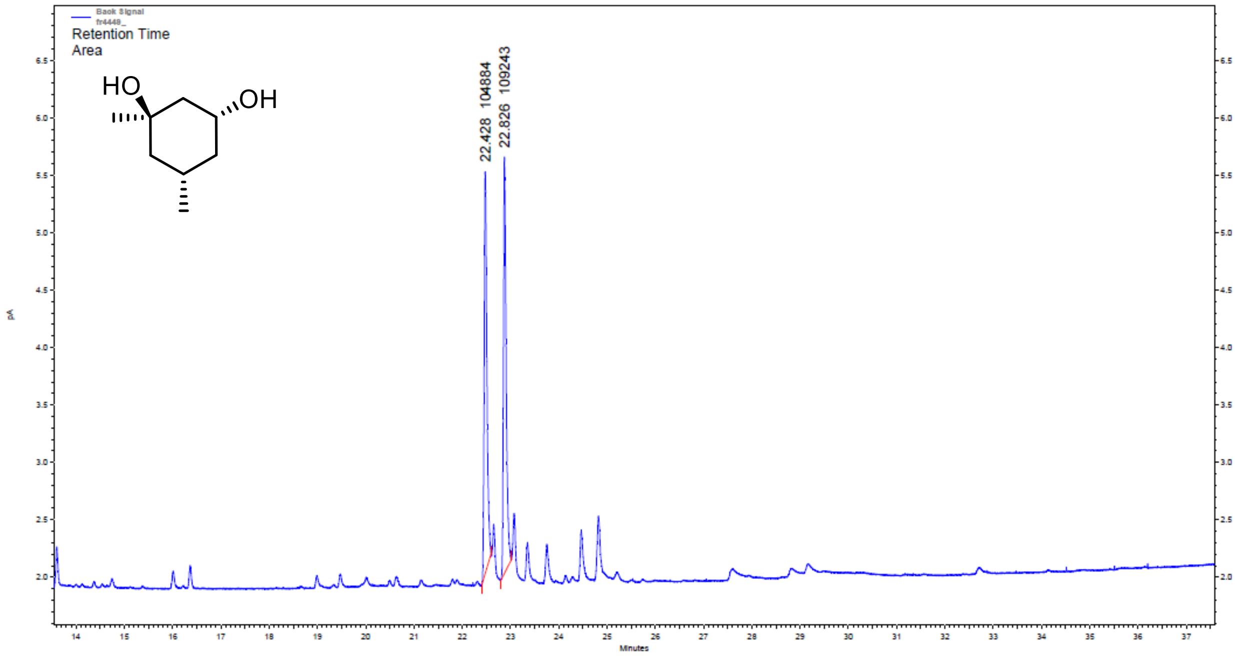
Retention Time
Area
HO
OH
22.428 104884
22.826 109243
pA
Minutes

***ee*-6e**

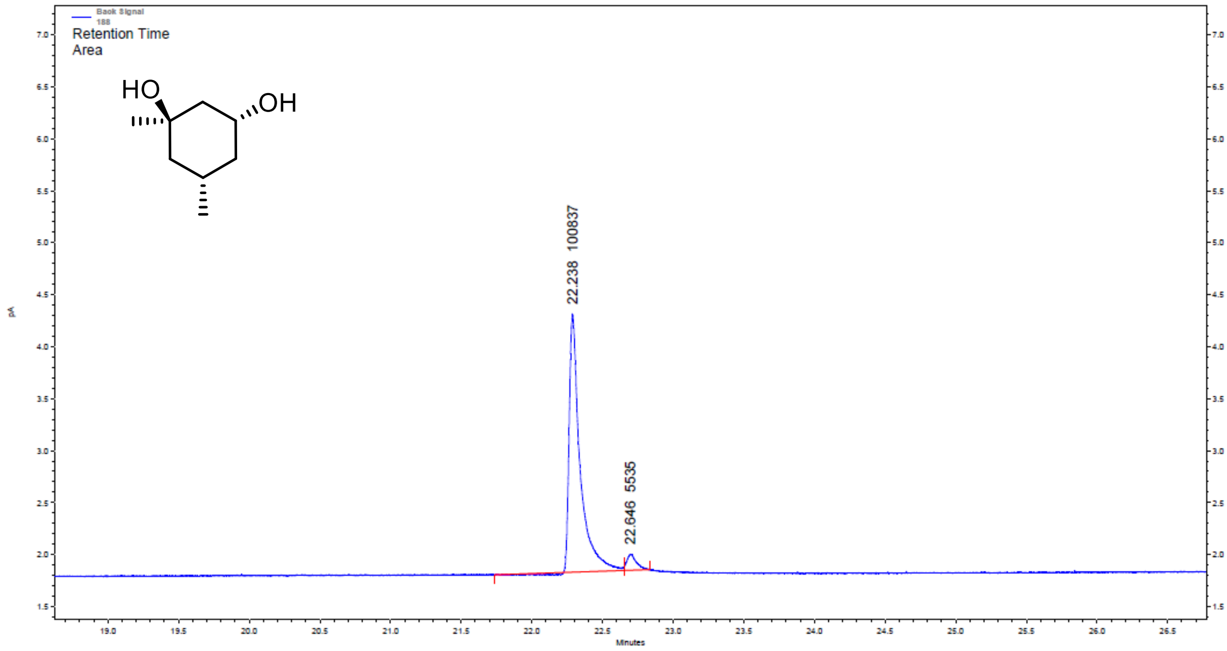
Retention Time
Area
HO
OH
22.238 100837
22.646 5535
pA
Minutes

***Rac*-6c**

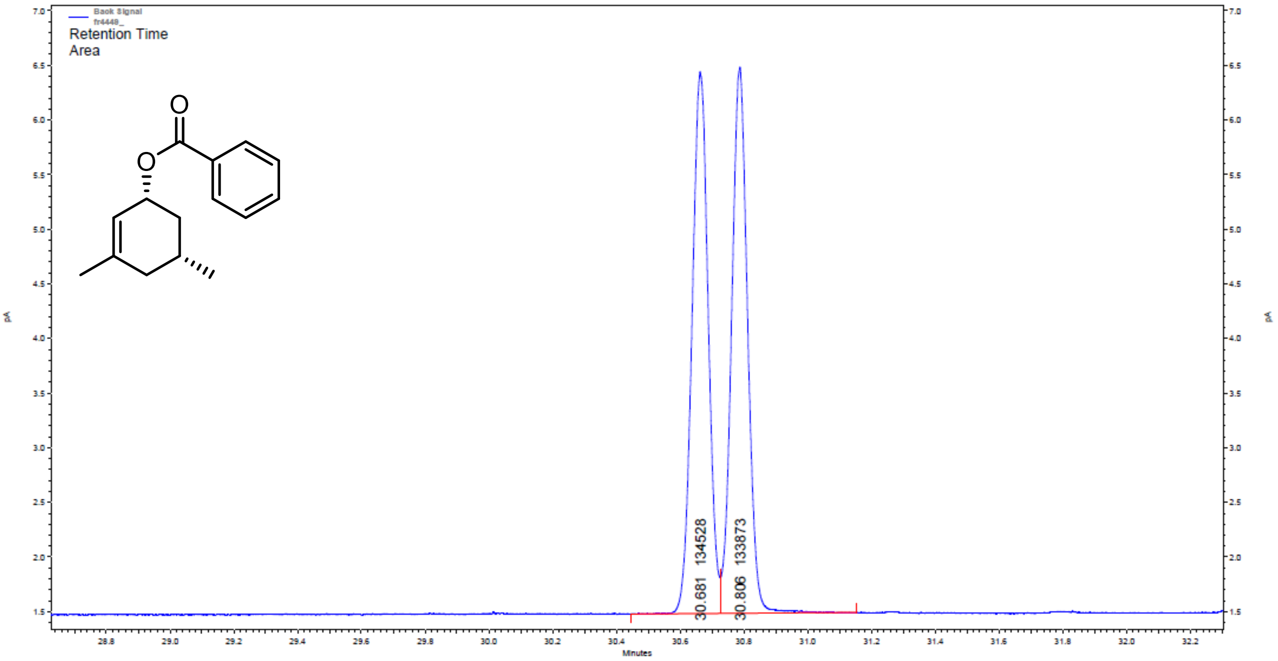
Back Signal
fr4449_
Retention Time
Area
O
O
134528
133873
30.681
30.806
pA
Minutes

***ee*-6c**

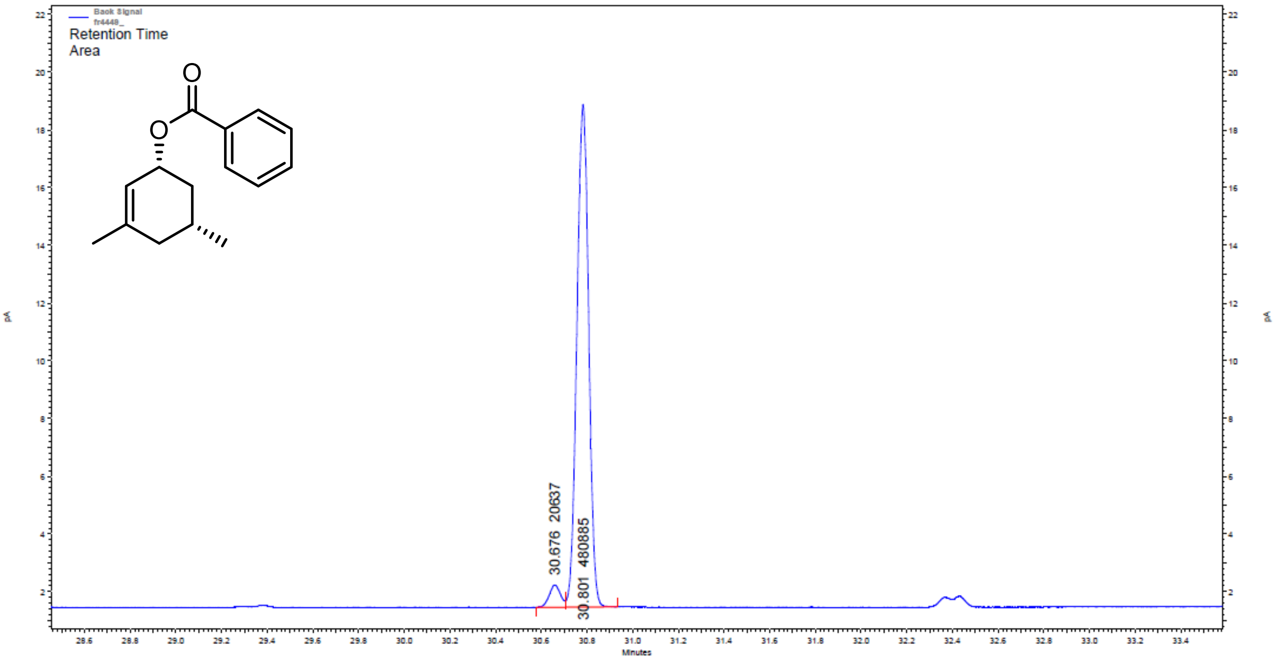
Back Signal
fr4449_
Retention Time
Area
O
O
30.676 20637
30.801 480885
pA
Minutes

The enantiomeric excess of **16c** was obtained by a comparison with gc traces of **DMM-16c**.

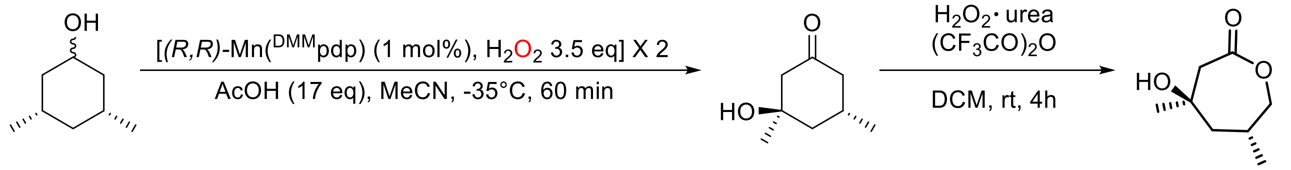

DMM-16b **50%** (13% ee) **DMM-16c** 12% ee

DMM-16b was obtained following oxidation protocol A with 17 eq. of acetic acid (AcOH), 1 mol% of *(R,R)*-Mn(^{DMM}pdp) and 3.5 eq of H_2O_2, after 30 min to the reaction mixture were added other 1 mol% of the catalyst and re-exposed to 3.5 equivalent of H_2O_2. Then **DMM-16c** was obtained from **DMM-16b** using Baeyer-Villiger oxidation as for **16c**.

***DMM*-16c**

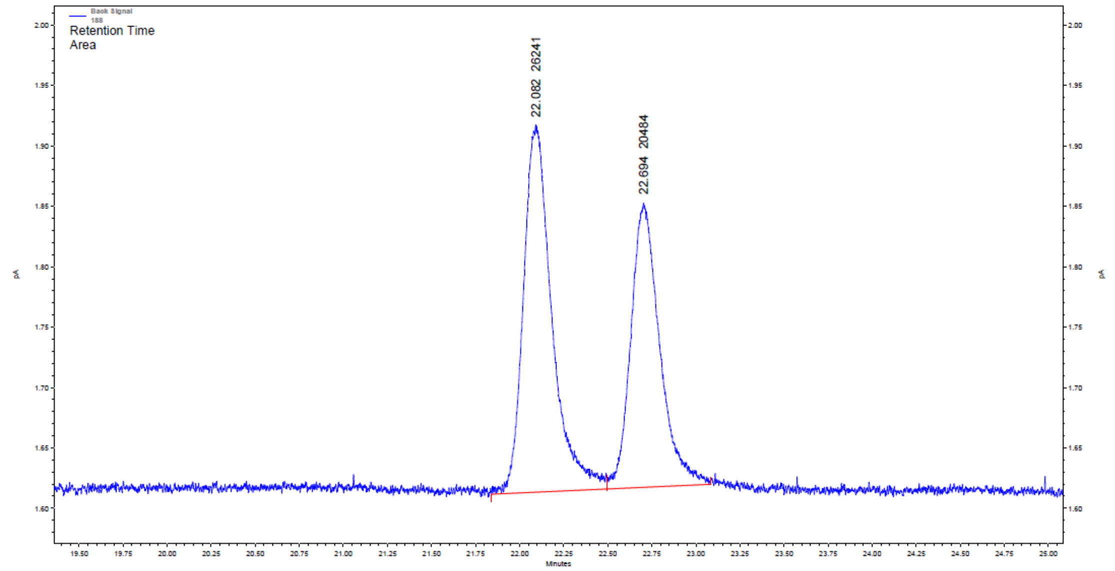

***ee*-16c**

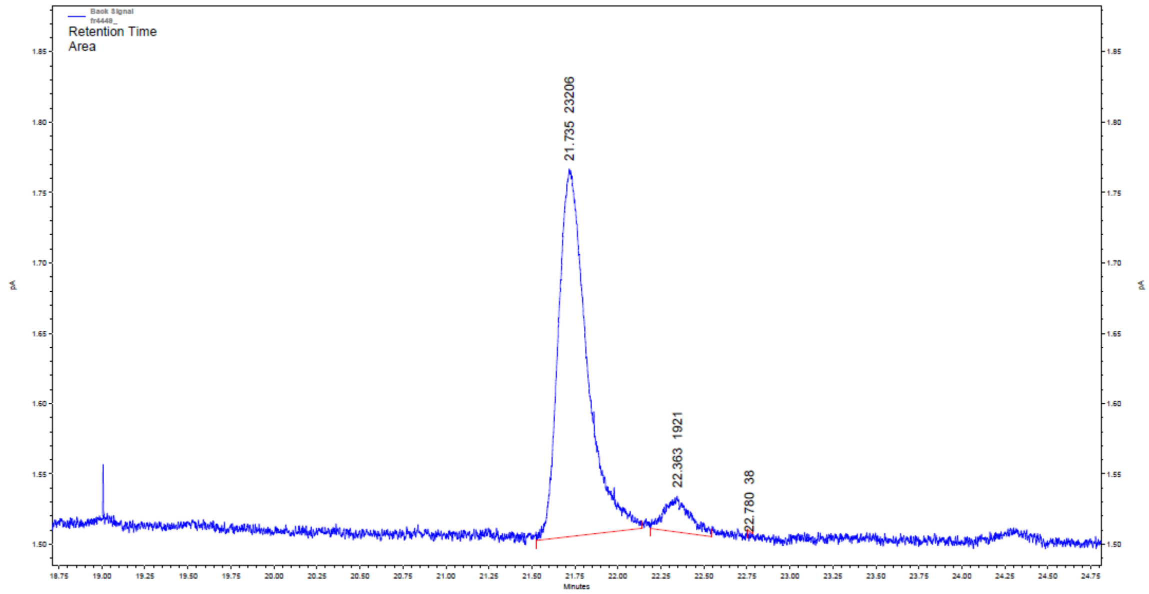

1.9 Computational Approaches

Electronic structure calculations. All the high-level energy and optimization calculations were performed with Gaussian 16 a03.[24] The systems were described with UM06-L density functional,[25] with the D3 dispersion correction[26] and adding solvation corrections (acetonitrile) through the Solvation Model based on Density (SMD).[27] 6-31G* was used as basis set for atoms (C, O, N, H and Si atoms)[16] and the Def2SVP pseudopotential for the central Mn atom.[28] A triplet electronic state (S=1) was set in all calculations, with a positive charge of 2. This specific level of calculations and electronic state were shown to be correct for accurately describing a similar Mn-based system with a related reaction.[29] Single-point electronic energy calculation of the reactant complex (RC) and transition states (TS) were performed by increasing the basis set quality to Def2TZVPP.[30]

Computational pipeline developed based on CREST software. The developed computational pipeline is based on the combination of conformer-rotamer ensemble sampling tool (CREST),[31]geometry-based clusterization procedures,[32] SP energy calculations and geometry optimizations at the UM06-L-D3 level of theory (see Figure S2). The pipeline starts with the evaluation of the conformational flexibility with CREST, which provided 1000-2000 structures within the 10 kcal/mol range at the semiempirical extended tight binding theory (xTB) level of theory (GFN2-xTB/ALPB(ACN)), with triplet electronic state (S=1) and a positive charge of 2.[33] Some modifications were introduced in the default CREST protocol: the length of the metadynamics simulations was reduced to 5-6 ps, and some constraints in the angles and distances were imposed. Optimization with xTB produces N-Mn bonds with a large difference in distance between xTB with respect to UM06-L-D3, in addition to that, xTB favors the anti conformation of the carbonyl group of the coligand and the Mn=O, as opposed to UM06-L-D3. For these reasons, these bond distances and angles were frozen at the equilibrium distances obtained for UM06-L-D3 for all optimizations run in this work. Also, the angles and distances related to the HAT hydrogen atom were also frozen to find specific conformations closer to the RC and HAT TSs. The 1000-2000 structures obtained via CREST were then ranked according to xTB relative energies, and those conformations within the 0-2 or 0-4 kcal/mol (depending on the case) were then clusterized using the Butina clustering from RDKit software based on the root-mean-square deviation (RMSD) matrix.[34] The centroid structure from each cluster (60-150 structures) were then subjected to SP energy calculations at the UM06-

L-D3/6-31G(d)(Mn-Def2SVP)/SMD(ACN) level of theory. Those structures presenting relative energies between 0-2 kcal/mol (10-15 structures, depending on the case) were then clustered using the same methodology as before, and the lowest in energy conformations (1-5 structures) were then fully optimized at the DFT level. SP energy calculations at UM06-L-D3/Def2TZVPP/SMD(ACN)//UM06-L-D3/6-31g(d)(Mn-Def2SVP)/SMD(ACN) were finally performed.

This procedure was applied for the free-catalyst, the reactant complex (RC) and HAT transition states (TS) for both C-3 and C-5 for the model substrate **18a**. As starting structures, we located several RC and TS corresponding to substrate **18a** for both C-3 and C-5 asymmetric hydroxylation, at the DFT level. These optimized structures were then input to CREST for finding additional lower in energy conformations at the semiempirical xTB level of theory, which provided more than 1000 structures within the 10 kcal/mol threshold. These structures were then further refined using the described protocol (Figure S2). The final TS and RC of substrate **18a** was then used as a template to generate C-3 and C-5 inputs for **27a** TS, **28a** RC, and **29a** RC. The RC for **27a** was directly located from the IRC calculation of the analytical vibrational frequency calculations of the HAT TS.

The TS geometries for both substrates **18a** and **27a** for C-3 and C-5 were then further analyzed by monitoring the non-covalent interactions established with the NCIplot using the atomic pre-computed spherically averaged LDA densities to compute the reduced density gradients.[35,36]

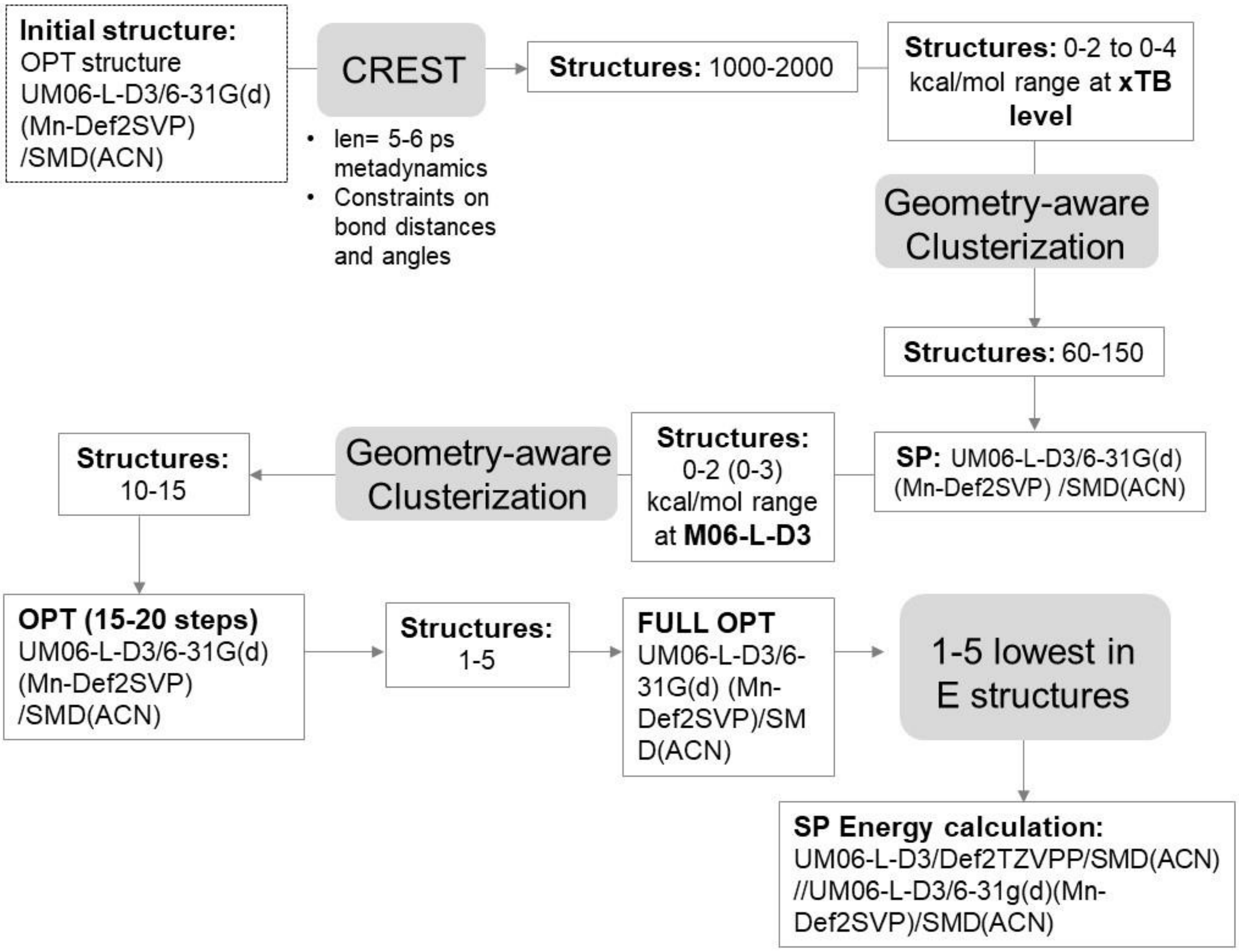

Figure S2. *Computational pipeline used based on the combination of the CREST program, geometry-based clusterization procedure, single point (SP) energy calculations and geometry optimizations at the UM06-L-D3 level of theory.*

Active site volume calculations. The free volumes for the substrate to fit in the Mn=O catalyst were computed with the software *MORFEUS*[37] and the steric maps were computed with the software *SambVca 2.1.*[38] The parameters used to compute the free volume and the steric maps was as follows: sphere center = O^{Mn}; z axis = equatorial plane (i.e., xy plane) formed by the atoms (Mn , O^{Phth}, $N^{pyridine}$, $N^{pyridine}$, $N^{pyrrolidine}$); excluded atom = O^{Mn} ; xz-plane = O^{Phth}(coordinated to Mn) and equatorial plane $N^{pyrrolidine}$; radius = 7; include hydrogens = True.

1.10 Computational Analyses

Rationalization of the conformational flexibility and preorganization of the Mn-catalyst

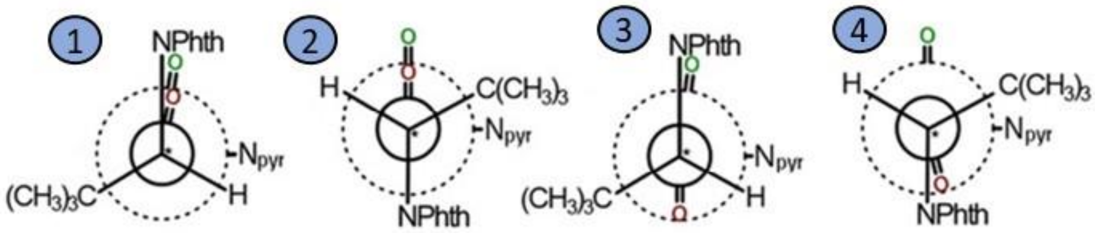

Figure S3. *Newman projections through the Cα-CO2 bond of the Mn-bound Phth-Tle-O ligand.*

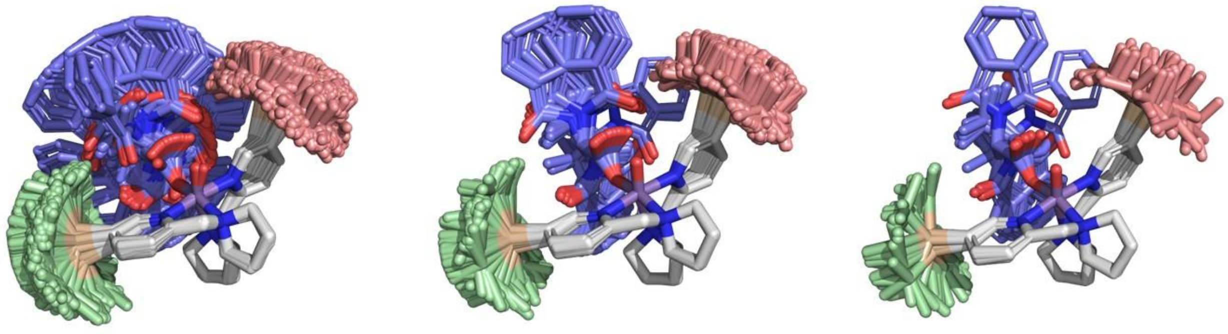

Figure S4. *Generated CREST ensembles for the free catalyst: the left image corresponds to the first 1000 structures generated from CREST at the xTB level, the middle figure shows the reduced number of conformations based on xTB relative energies and after applying the first clusterization protocol (136 structures), and the right panel shows the 14 lowest in energy conformations at the DFT level found in the second clusterization step.*

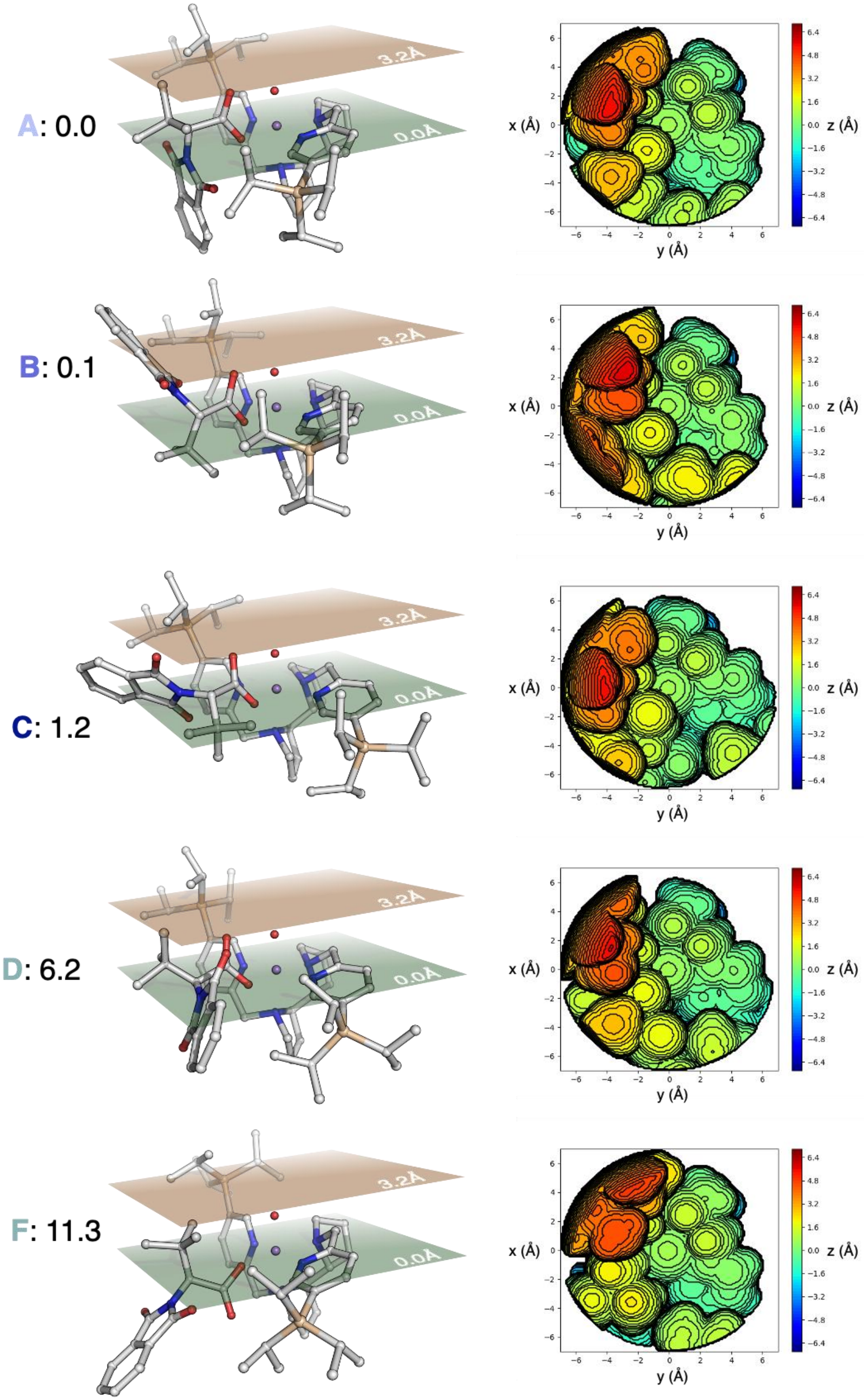
A: 0.0
B: 0.1
C: 1.2
D: 6.2
F: 11.3
3.2Å
0.0Å
x (Å)
y (Å)
z (Å)
6.4
4.8
3.2
1.6
0.0
−1.6
−3.2
−4.8
−6.4

Figure S5. *Volume calculations of the A-F conformations of the free catalyst: (A) the lowest in energy conformation in which the tert-butyl group of the coligand is situated close to the carbonyl group, (B) that is only 0.1 kcal/mol higher in energy and instead presents the phthalimide group located close to the carbonyl, (C) at 1.2 kcal/mol in which the Phth is establishing C-H·· π interactions with the TIPS^R group, (D) at 6.2 kcal/mol where the Phth is establishing C-H·· π interactions with the TIPS,^{L} and (F) at 11.3 kcal/mol that presents the carbonyl group of the coligand in anti with respect to the Mn=O.*

Table S10. *Relative energies between the conformations for the catalyst in the absence of any substrate. Details of electronic method are described in the method section above. All energies are expressed in kcal/mol.*

Conformation cat.	UM06-L-D3 rel. energy
A	0.0
B	0.1
C	1.2
D	6.2

Rationalization of the origins of the enantioselectivity in the catalyst substrate complexes and transitions states

Table S11. *Relative energy between the different optimized HAT transition states for substrates **18a** and **27a**. Details of electronic method described in the method section. Energies in kcal/mol.*

Substrate	HAT position	UM06-L-D3 rel. energy
18a	C3	0.0
	C5	3.4
	C3 (catalyst in conformation 4)	7.6
27a	C3	0.1
	C5	0.0

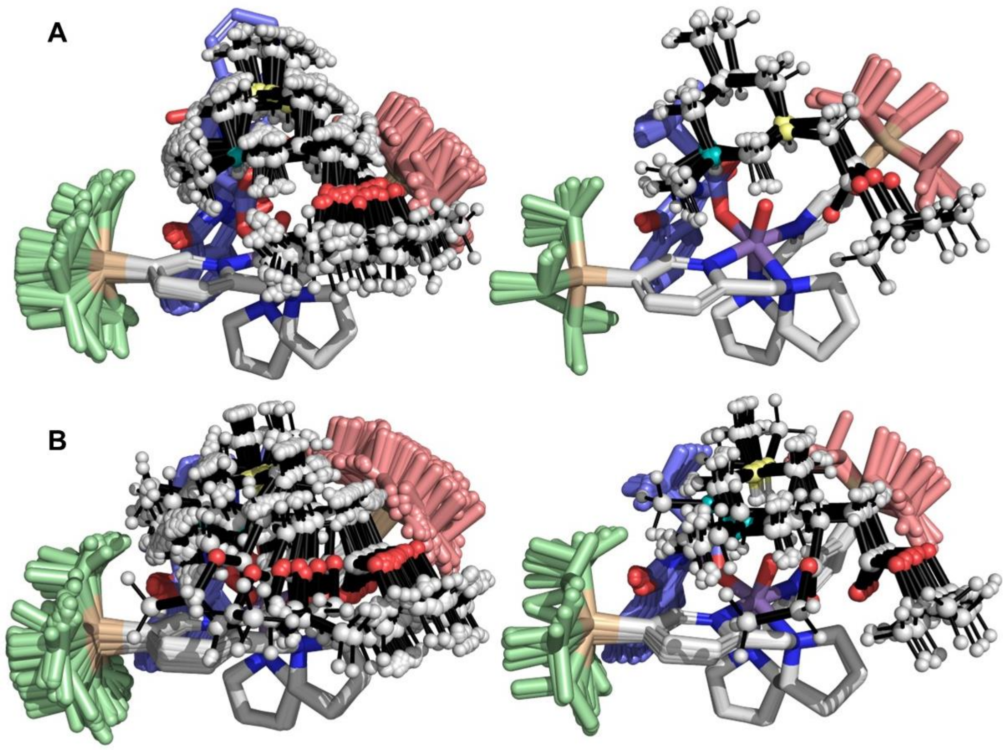

Figure S6. *CREST ensembles for the reactant complexes of substrate* ***18a*** *for (A) C-3 and (B) C-5. Right image conformations are obtained from the first clusterization step of the protocol. Left image conformations are obtained from the second clusterization step of the protocol.*

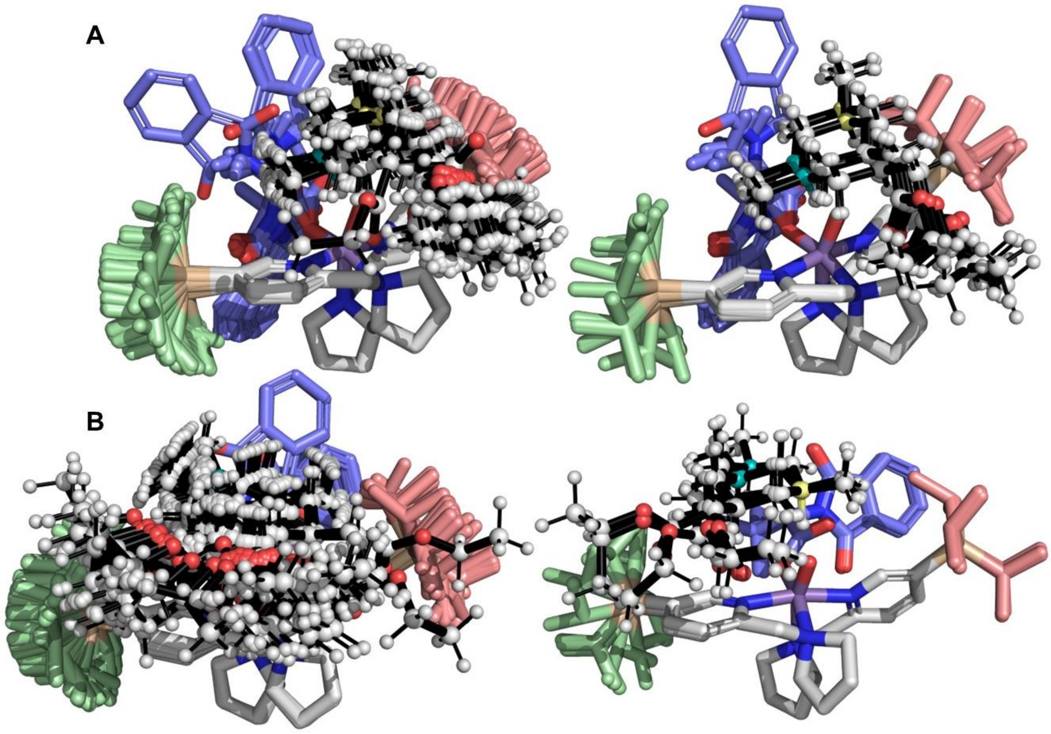

Figure S7. *CREST ensembles for the HAT transition states of substrate* ***18a*** *for (A) C-3 and (B) C-5. Right image conformations are obtained from the first clusterization step of the protocol. Left image conformations are obtained from the second clusterization step of the protocol.*

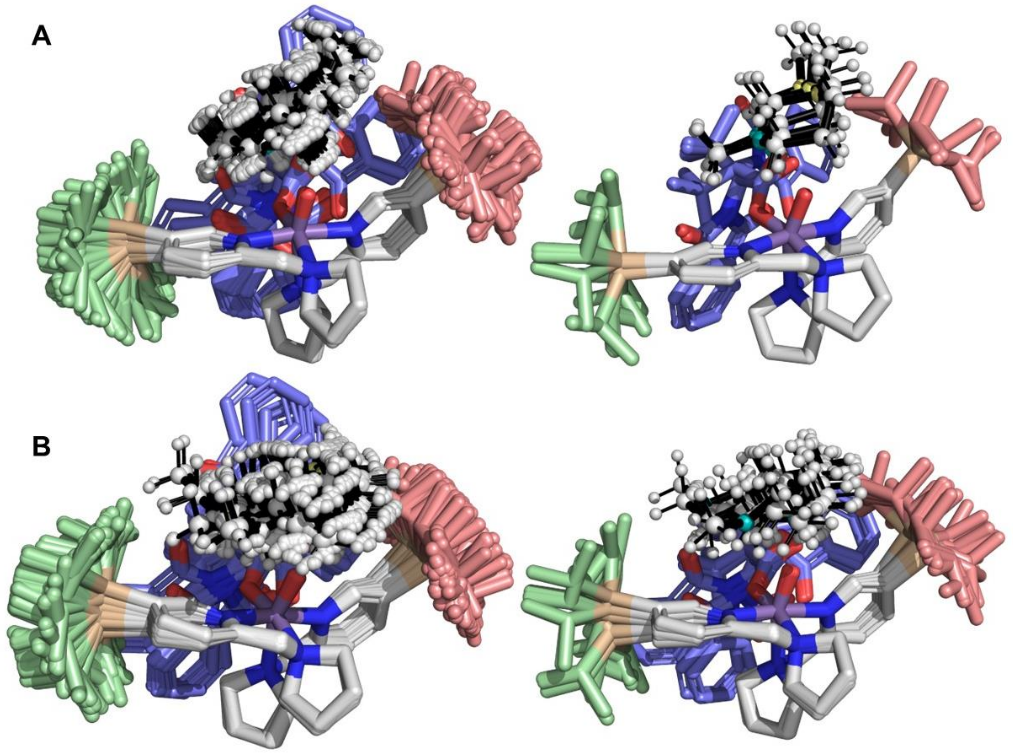

Figure S8. *CREST ensembles for the HAT transition states of substrate* ***27a*** *for (A) C-3 and (B) C-5. Right image conformations are obtained from the first clusterization step of the protocol. Left image conformations are obtained from the second clusterization step of the protocol.*

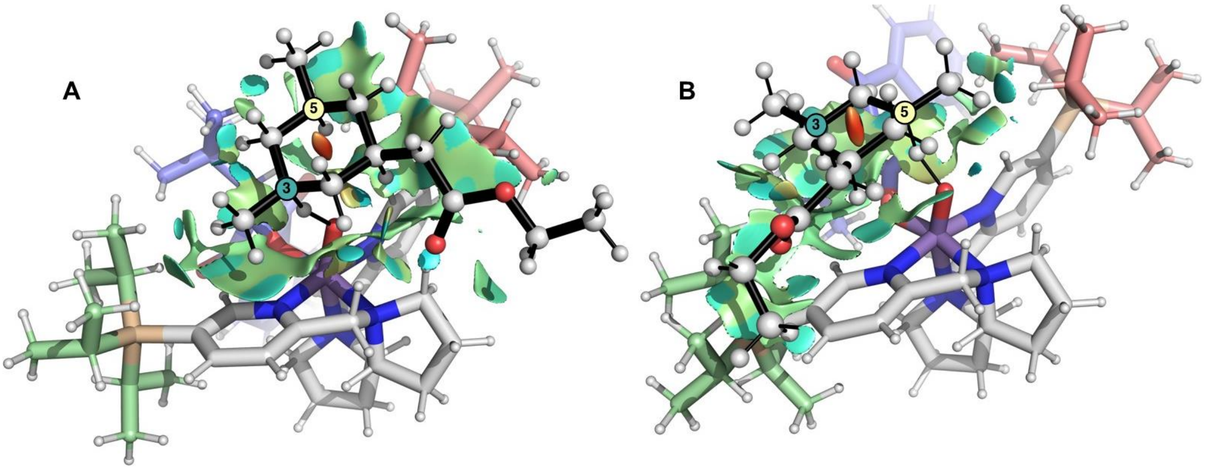

Figure S9. *Non-covalent interactions obtained by NCIPLOT for the HAT transition state of* ***18a*** *for (A) C-3, and (B) C-5.*

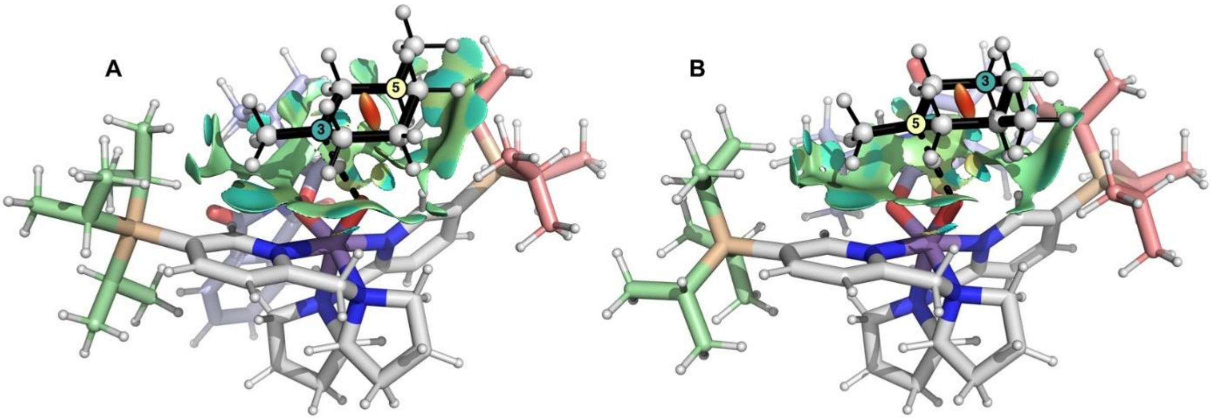

Figure S10. *Non-covalent interactions obtained by NCIPLOT for the HAT transition state of* ***27a*** *for (A) C-3, and (B) C-5.*

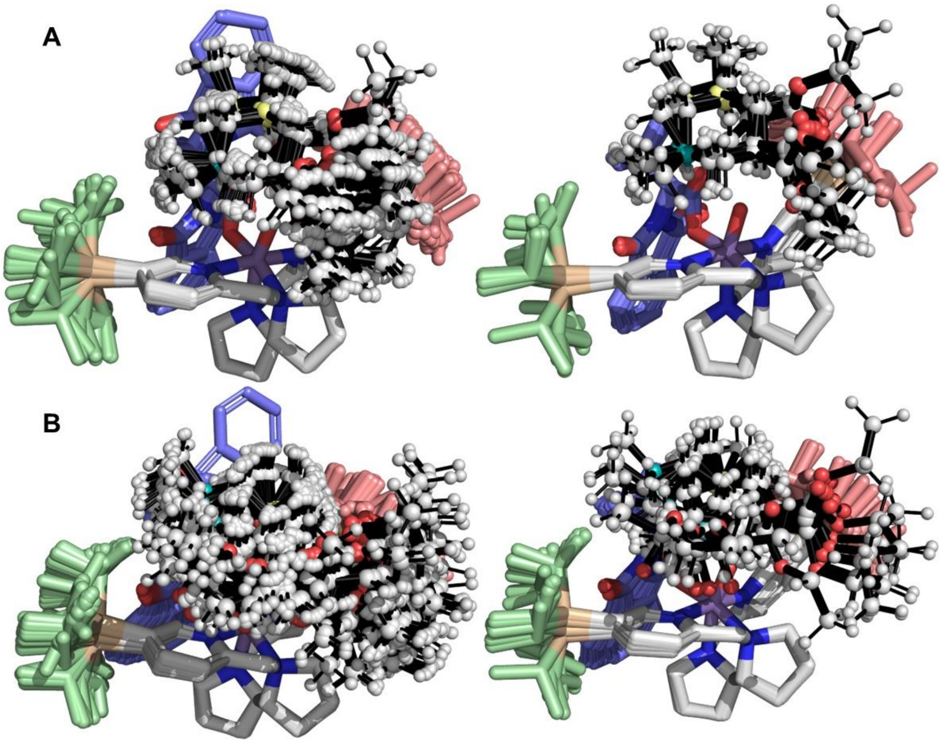

Figure S11. *CREST ensembles for the reactant complexes of substrates* ***17a*** *for (A) C-3 and (B) C-5. Right image conformations are obtained from the first clusterization step of the protocol. Left image conformations are obtained from the second clusterization step of the protocol.*

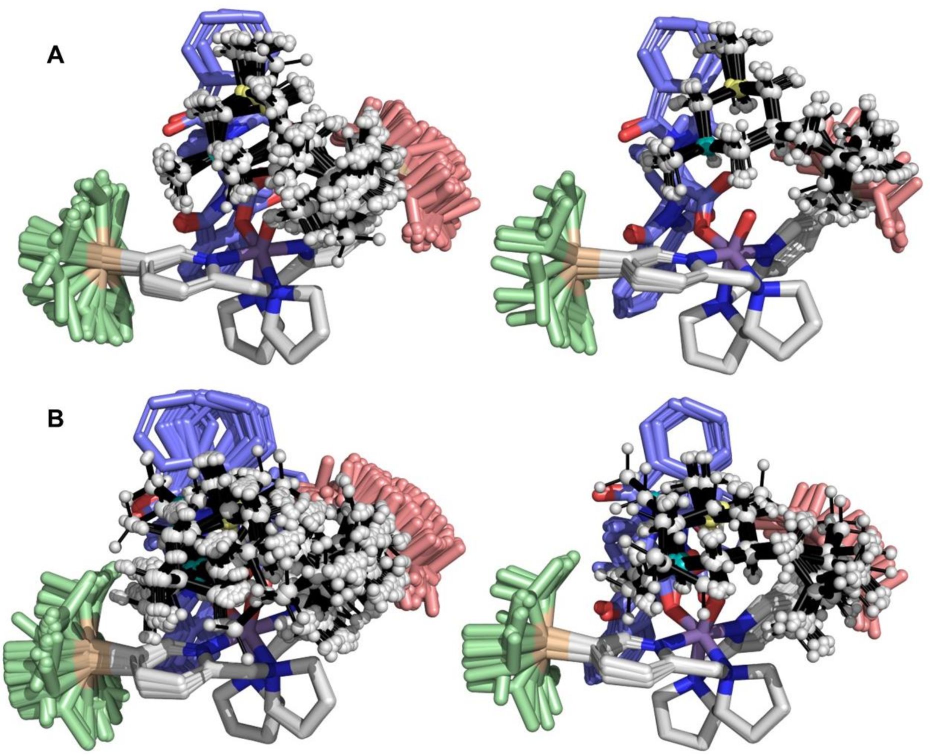

Figure S12. *CREST ensembles for the reactant complexes of substrates* ***28a*** *for (A) C-3 and (B) C-5. Right image conformations are obtained from the first clusterization step of the protocol. Left image conformations are obtained from the second clusterization step of the protocol.*

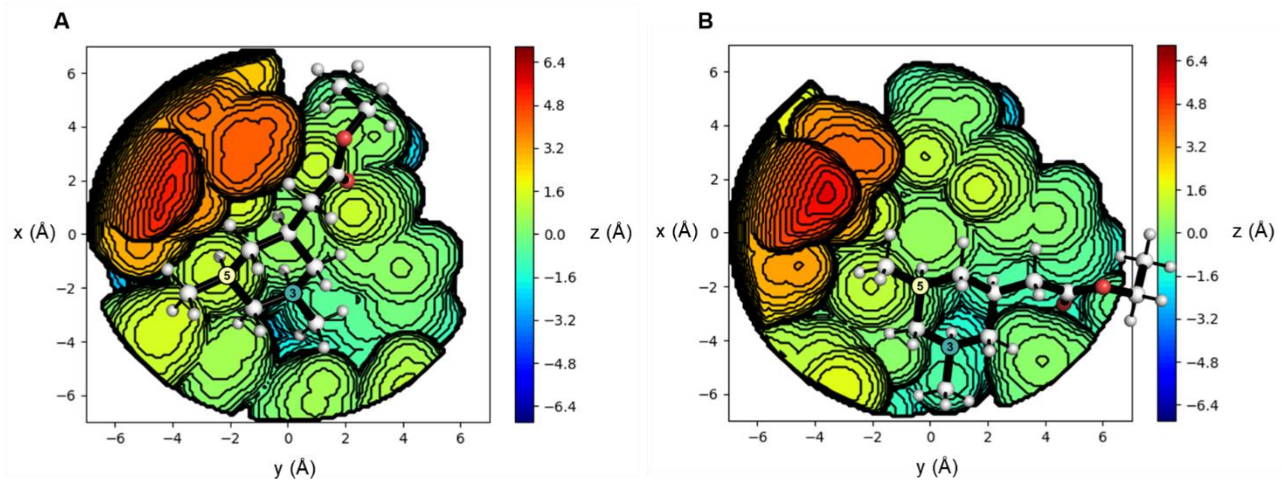

Figure S13. *Steric maps of the free catalyst for the HAT transition state superimposed with* ***18a*** *for (A) C-3 hydroxylation with the catalyst in conformation A, and (B) C-5 hydroxylation with the catalyst in conformation C.*

1.11 References

(1) Mahoney, W. S.; Stryker, J. M. Hydride-Mediated Homogeneous Catalysis. Catalytic Reduction of .Alpha.,.Beta.-Unsaturated Ketones Using $[(Ph_3P)CuH]_6$ and H_2. *J. Am. Chem. Soc.* **1989**, *111* (24), 8818–8823. https://doi.org/10.1021/ja00206a008.

(2) Naruse, Y.; Yamamoto, H. Asymmetrization of Meso-Cyclic Ketones Using Homochiral Acetal Templates. *Tetrahedron* **1988**, *44* (19), 6021–6029. https://doi.org/10.1016/S0040-4020(01)89790-8.

(3) Elumalai, V.; Hansen, J. H. A Scalable and Green One-Minute Synthesis of Substituted Phenols. *RSC Adv.* **2020**, *10* (66), 40582–40587. https://doi.org/10.1039/d0ra08580d.

(4) Schäfer, M.; Stünkel, T.; Daniliuc, C. G.; Gilmour, R. Regio- and Enantioselective Intermolecular Aminofluorination of Alkenes via Iodine(I)/Iodine(III) Catalysis. *Angew. Chemie - Int. Ed.* **2022**, *61* (32). https://doi.org/10.1002/anie.202205508.

(5) Diemer, V.; Chaumeil, H.; Defoin, A.; Fort, A.; Boeglin, A.; Carré, C. Syntheses of Sterically Hindered Pyridinium Phenoxides as Model Compounds in Nonlinear Optics. *European J. Org. Chem.* **2006**, No. 12, 2727–2738. https://doi.org/10.1002/ejoc.200600030.

(6) Wollenburg, M.; Heusler, A.; Bergander, K.; Glorius, F. Trans-Selective and Switchable Arene Hydrogenation of Phenol Derivatives. *ACS Catal.* **2020**, *10* (19), 11365–11370. https://doi.org/10.1021/acscatal.0c03423.

(7) Wu, W.; Cao, W.; Hu, L.; Su, Z.; Liu, X.; Feng, X. Asymmetric Baeyer-Villiger Oxidation: Classical and Parallel Kinetic Resolution of 3-Substituted Cyclohexanones and Desymmetrization of: Meso -Disubstituted Cycloketones. *Chem. Sci.* **2019**, *10* (29), 7003–7008. https://doi.org/10.1039/c9sc01563a.

(8) Lambert, K. M.; Stempel, Z. D.; Wiberg, K. B.; Bailey, W. F. Experimental Demonstration of a Sizeable Nonclassical CH···G Hydrogen Bond in Cyclohexane Derivatives: Stabilization of an Axial Cyano Group. *Org. Lett.* **2017**, *19* (23), 6408–6411. https://doi.org/10.1021/acs.orglett.7b03287.

(9) Chowdhury, L.; Croft, C. J.; Goel, S.; Zaman, N.; Tai, A. C. S.; Walch, E. M.; Smith, K.; Page, A.; Shea, K. M.; Hall, C. D.; Jishkariani, D.; Pillai, G. G.; Hall, A. C. Differential Potency of 2,6-Dimethylcyclohexanol Isomers for Positive Modulation of GABAA Receptor Currents. *J. Pharmacol. Exp. Ther.* **2016**, *357* (3), 570–579. https://doi.org/10.1124/jpet.115.228890.

(10) Mihovilovic, M. D.; Rudroff, F.; Grötzl, B.; Stanetty, P. Microbial Baeyer-Villiger Oxidation of

Prochiral Polysubstituted Cyclohexanones by Recombinant Whole-Cells Expressing Two Bacterial Monooxygenases. *European J. Org. Chem.* **2005**, No. 5, 809–816. https://doi.org/10.1002/ejoc.200400676.

(11) Milan, M.; Bietti, M.; Costas, M. Highly Enantioselective Oxidation of Nonactivated Aliphatic C-H Bonds with Hydrogen Peroxide Catalyzed by Manganese Complexes. *ACS Cent. Sci.* **2017**, *3* (3), 196–204. https://doi.org/10.1021/acscentsci.6b00368.

(12) Cussó, O.; Garcia-Bosch, I.; Font, D.; Ribas, X.; Lloret-Fillol, J.; Costas, M. Highly Stereoselective Epoxidation with H_2O_2 Catalyzed by Electron-Rich Aminopyridine Manganese Catalysts. *Org. Lett.* **2013**, *15* (24), 6158–6161. https://doi.org/10.1021/ol403018x.

(13) Cianfanelli, M.; Olivo, G.; Milan, M.; Klein Gebbink, R. J. M.; Ribas, X.; Bietti, M.; Costas, M. Enantioselective C-H Lactonization of Unactivated Methylenes Directed by Carboxylic Acids. *J. Am. Chem. Soc.* **2020**, *142* (3), 1584–1593. https://doi.org/10.1021/jacs.9b12239.

(14) Ottenbacher, R. V.; Bryliakov, K. P.; Talsi, E. P. Non-Heme Manganese Complexes Catalyzed Asymmetric Epoxidation of Olefins by Peracetic Acid and Hydrogen Peroxide. *Adv. Synth. Catal.* **2011**, *353* (6), 885–889. https://doi.org/10.1002/adsc.201100030.

(15) Font, D.; Canta, M.; Milan, M.; Cussó, O.; Ribas, X.; Klein Gebbink, R. J. M.; Costas, M. Readily Accessible Bulky Iron Catalysts Exhibiting Site Selectivity in the Oxidation of Steroidal Substrates. *Angew. Chemie* **2016**, *128* (19), 5870–5873. https://doi.org/10.1002/ange.201600785.

(16) Guo, H.; Zhang, L. W.; Zhou, H.; Meng, W.; Ao, Y. F.; Wang, D. X.; Wang, Q. Q. Substrate-Induced Dimerization Assembly of Chiral Macrocycle Catalysts toward Cooperative Asymmetric Catalysis. *Angew. Chemie - Int. Ed.* **2020**, *59* (7), 2623–2627. https://doi.org/10.1002/anie.201910399.

(17) Vicens, L.; Bietti, M.; Costas, M. General Access to Modified α-Amino Acids by Bioinspired Stereoselective γ-C–H Bond Lactonization. *Angew. Chemie* **2021**, *133* (9), 4790–4796. https://doi.org/10.1002/ange.202007899.

(18) Milan, M.; Bietti, M.; Costas, M. Aliphatic C-H Bond Oxidation with Hydrogen Peroxide Catalyzed by Manganese Complexes: Directing Selectivity through Torsional Effects. *Org. Lett.* **2018**, *20* (9), 2720–2723. https://doi.org/10.1021/acs.orglett.8b00929.

(19) Escudero-Adán, E. C.; Benet-Buchholz, J.; Ballester, P. The Use of Mo Kα Radiation in the Assignment of the Absolute Configuration of Light-Atom Molecules; The Importance of

High-Resolution Data. *Acta Crystallogr. Sect. B Struct. Sci. Cryst. Eng. Mater.* **2014**, *70* (4), 660–668. https://doi.org/10.1107/S2052520614014498.

(20) Hooft, R. W. W.; Straver, L. H.; Spek, A. L. Determination of Absolute Structure Using Bayesian Statistics on Bijvoet Differences. *J. Appl. Crystallogr.* **2008**, *41* (1), 96–103. https://doi.org/10.1107/S0021889807059870.

(21) Parsons, S.; Flack, H. D.; Wagner, T. Use of Intensity Quotients and Differences in Absolute Structure Refinement. *Acta Crystallogr. Sect. B Struct. Sci. Cryst. Eng. Mater.* **2013**, *69* (3), 249–259. https://doi.org/10.1107/S2052519213010014.

(22) Spek, A. L. Structure Validation in Chemical Crystallography. *Acta Crystallogr. Sect. D Biol. Crystallogr.* **2009**, *65* (2), 148–155. https://doi.org/10.1107/S090744490804362X.

(23) Senda, Y.; Ishiyama, J.; Imaizumi, S. Carbon-13 Pulse Fourier Transform NMR Spectra of Substituted 1-Methylcyclohexanols. *Tetrahedron* **1975**, *31* (13–14), 1601–1605. https://doi.org/10.1016/0040-4020(75)87020-7.

(24) Frisch, M. J.; Trucks, G. W.; Schlegel, H. B.; Scuseria, G. E.; Robb, M. a.; Cheeseman, J. R.; Scalmani, G.; Barone, V.; Petersson, G. a.; Nakatsuji, H.; Li, X.; Caricato, M.; Marenich, a. V.; Bloino, J.; Janesko, B. G.; Gomperts, R.; Mennucci, B.; Hratchian, H. P.; Ortiz, J. V.; Izmaylov, a. F.; Sonnenberg, J. L.; Williams; Ding, F.; Lipparini, F.; Egidi, F.; Goings, J.; Peng, B.; Petrone, A.; Henderson, T.; Ranasinghe, D.; Zakrzewski, V. G.; Gao, J.; Rega, N.; Zheng, G.; Liang, W.; Hada, M.; Ehara, M.; Toyota, K.; Fukuda, R.; Hasegawa, J.; Ishida, M.; Nakajima, T.; Honda, Y.; Kitao, O.; Nakai, H.; Vreven, T.; Throssell, K.; Montgomery Jr., J. a.; Peralta, J. E.; Ogliaro, F.; Bearpark, M. J.; Heyd, J. J.; Brothers, E. N.; Kudin, K. N.; Staroverov, V. N.; Keith, T. a.; Kobayashi, R.; Normand, J.; Raghavachari, K.; Rendell, a. P.; Burant, J. C.; Iyengar, S. S.; Tomasi, J.; Cossi, M.; Millam, J. M.; Klene, M.; Adamo, C.; Cammi, R.; Ochterski, J. W.; Martin, R. L.; Morokuma, K.; Farkas, O.; Foresman, J. B.; Fox, D. J. G16_C01. 2016, p Gaussian 16, Revision C.01, Gaussian, Inc., Wallin.

(25) Zhao, Y.; Truhlar, D. G. A New Local Density Functional for Main-Group Thermochemistry, Transition Metal Bonding, Thermochemical Kinetics, and Noncovalent Interactions. *J. Chem. Phys.* **2006**, *125* (19). https://doi.org/10.1063/1.2370993.

(26) Grimme, S.; Antony, J.; Ehrlich, S.; Krieg, H. A Consistent and Accurate Ab Initio Parametrization of Density Functional Dispersion Correction (DFT-D) for the 94 Elements H-Pu. *J. Chem. Phys.* **2010**, *132* (15). https://doi.org/10.1063/1.3382344.

(27) Marenich, A. V.; Cramer, C. J.; Truhlar, D. G. Universal Solvation Model Based on Solute

Electron Density and on a Continuum Model of the Solvent Defined by the Bulk Dielectric Constant and Atomic Surface Tensions. *J. Phys. Chem. B* **2009**, *113* (18), 6378–6396. https://doi.org/10.1021/jp810292n.

(28) Schäfer, A.; Horn, H.; Ahlrichs, R. Fully Optimized Contracted Gaussian Basis Sets for Atoms Li to Kr. *J. Chem. Phys.* **1992**, *97* (4), 2571–2577. https://doi.org/10.1063/1.463096.

(29) Call, A.; Cianfanelli, M.; Besalú-Sala, P.; Olivo, G.; Palone, A.; Vicens, L.; Ribas, X.; Luis, J. M.; Bietti, M.; Costas, M. Carboxylic Acid Directed γ-Lactonization of Unactivated Primary C-H Bonds Catalyzed by Mn Complexes: Application to Stereoselective Natural Product Diversification. *J. Am. Chem. Soc.* **2022**, *144* (42), 19542–19558. https://doi.org/10.1021/jacs.2c08620.

(30) Schäfer, A.; Huber, C.; Ahlrichs, R. Fully Optimized Contracted Gaussian Basis Sets of Triple Zeta Valence Quality for Atoms Li to Kr. *J. Chem. Phys.* **1994**, *100* (8), 5829–5835. https://doi.org/10.1063/1.467146.

(31) Pracht, P.; Bohle, F.; Grimme, S. Automated Exploration of the Low-Energy Chemical Space with Fast Quantum Chemical Methods. *Phys. Chem. Chem. Phys.* **2020**, *22* (14), 7169–7192. https://doi.org/10.1039/c9cp06869d.

(32) Butina, D. Unsupervised Data Base Clustering Based on Daylight's Fingerprint and Tanimoto Similarity: A Fast and Automated Way to Cluster Small and Large Data Sets. *J. Chem. Inf. Comput. Sci.* **1999**, *39* (4), 747–750. https://doi.org/10.1021/ci9803381.

(33) Bannwarth, C.; Caldeweyher, E.; Ehlert, S.; Hansen, A.; Pracht, P.; Seibert, J.; Spicher, S.; Grimme, S. Extended Tight-Binding Quantum Chemistry Methods. *Wiley Interdiscip. Rev. Comput. Mol. Sci.* **2021**, *11* (2), 1–49. https://doi.org/10.1002/wcms.1493.

(34) Landrum, G.; Tosco, P.; Kelley, B.; Ric; sriniker; gedeck; Vianello, R.; NadineSchneider; Kawashima, E.; Dalke, A.; N, D.; Cosgrove, D.; Jones, G.; Cole, B.; Swain, M.; Turk, S.; AlexanderSavelyev; Vaucher, A.; Wójcikowski, M.; Take, I.; Probst, D.; Ujihara, K.; Scalfani, V. F.; Godin, G.; Pahl, A.; Berenger, F.; JLVarjo; strets123; JP; DoliathGavid. Rdkit/Rdkit: 2022_03_3 (Q1 2022) [RDKit: Open-Source Cheminformatics. https://rdkit.org]. **2022**. https://doi.org/10.5281/ZENODO.6605135.

(35) Boto, R. A.; Peccati, F.; Laplaza, R.; Quan, C.; Carbone, A.; Piquemal, J. P.; Maday, Y.; Contreras-García, J. NCIPLOT4: Fast, Robust, and Quantitative Analysis of Noncovalent Interactions. *J. Chem. Theory Comput.* **2020**, *16* (7), 4150–4158. https://doi.org/10.1021/acs.jctc.0c00063.

(36) Laplaza, R.; Peccati, F.; A. Boto, R.; Quan, C.; Carbone, A.; Piquemal, J. P.; Maday, Y.; Contreras-García, J. NCIPLOT and the Analysis of Noncovalent Interactions Using the Reduced Density Gradient. *Wiley Interdiscip. Rev. Comput. Mol. Sci.* **2021**, *11* (2), 1–18. https://doi.org/10.1002/wcms.1497.

(37) Jorner, K.; Turcani, L. Kjelljorner/Morfeus: V0.7.2 [MORFEUS: Molecular Features for Machine Learning. https://github.com/kjelljorner/morfeus]. **2022**. https://doi.org/10.5281/ZENODO.7017599.

(38) Falivene, L.; Cao, Z.; Petta, A.; Serra, L.; Poater, A.; Oliva, R.; Scarano, V.; Cavallo, L. Towards the Online Computer-Aided Design of Catalytic Pockets. *Nat. Chem.* **2019**, *11* (10), 872–879. https://doi.org/10.1038/s41557-019-0319-5.
